# Targeting asparagine and cysteine in SARS-CoV-2 variants and human pro-inflammatory mediators to alleviate COVID-19 severity; a cross-section and in-silico study

**DOI:** 10.1038/s41598-025-19359-y

**Published:** 2025-11-03

**Authors:** Hewida H. Fadel, Hadeer Adel EL-Esseily, Mohammed Abd EL-Rahman Ahmed, Mohammed Ahmed Khamis Mohamed, Mohamed Nabil Roushdy, Amr ElSherif, Kareem Mahamoud Gharbeya, Hadeel Said Abdelsalam

**Affiliations:** 1 Department of Medical Laboratory Technology, Faculty of Applied Health Sciences Technology, Pharos University, Alexandria, Egypt; 2 Department of Health Care, Faculty of Computer and Data Science, Alexandria University, Alexandria, Egypt; 3 Department of Clinical Pathology, Alexandria Armed Forces Hospital, Military Medical Academy, Alexandria, Egypt; 4 Pulmonary Consultant at Alexandria Armed Forces Hospital, Alexandria, Egypt; 5 Department of Molecular and diagnostic microbiology, Alexandria Armed Forces Hospital, Alexandria, Egypt; 6 Department of Hematopathology, Military Medical Academy, Alexandria Armed Forces Hospital Alexandria, Alexandria, Egypt; 7 Department of Critical Care Consultant, Alexandria Armed Forces Hospital, Alexandria, Egypt; 8 Department of Medical Laboratory Technology, Faculty of Applied Health Sciences Technology, Pharos University, Alexandria, Egypt

**Keywords:** SARS-CoV-2, COVID-19, Interleukin-17, Asparaginase, Cysteine, Phytotherapies, Vaccine-adjuvants, Natural medicine, Biochemistry, Chemical biology, Computational biology and bioinformatics, Immunology, Biomarkers, Diseases, Health care, Medical research, Molecular medicine

## Abstract

To date, COVID-19 continues to pose a global health challenge, with substantial morbidity, mortality, and long-term post-COVID-19 complications threatening public health resilience. During the early pandemic, the IL-6 inhibitor (tocilizumab) was the widely used approved immunotherapy for critically ill patients; however, a subset of ICU cases exhibited normal interleukin-6 (IL-6) levels and failed to respond. We hypothesized that interleukin-17 (IL-17), which acts synergistically with IL-6, contributes to cytokine storm progression and severe inflammation. Our study uniquely integrates a clinical cross-sectional analysis with advanced in-silico modelling, directly linking patient-derived biomarker, radiological, and statistical data to molecular-level mechanisms of COVID-19 severity. Serum IL-17 was significantly elevated in critical versus moderate COVID-19 cases, with a threshold of 187.9 ng/mL predicting poor outcomes by ROC analysis. Logistic regression identified age and monocytes as independent predictors of severity, supporting a combined biomarker approach for improving the prognosis and clinical outcomes. Radiological findings, including ground-glass opacities and consolidations, alongside hematological abnormalities, were more frequent in critical cases. Computational docking revealed key amino acid residues—particularly asparagine (Asn) and cysteine (Cys)—as structural determinants shared by SARS-CoV-2 spike protein and human inflammatory mediators (IL-17R, IL-6R, CD41/CD61, CD47/SIRP). Asparaginase (ASNase) targeted critical residues such as the invariant gate residue “Asn343” and Cys213 of spike protein, Asn240 of IL-17R, and Asn136 of IL-6R. Several phytochemicals, including phytic acid and amygdalin, as well as synthetic agents such as candesartan, remdesivir, and enalapril, were found to preferentially bind to cysteine (Cys) residues—and, to a lesser extent, asparagine (Asn) residues—within key binding interfaces, in addition to targeting B-cell epitopes. This conserved residue preference supports the rationale for a dual-action therapeutic strategy in which asparaginase (ASNase) is combined with selected plant-derived ligands to simultaneously disrupt viral entry mechanisms and attenuate the inflammatory signalling. This dual-perspective approach not only identified IL-17 and IL-6 as independent severity predictors but also revealed conserved Asn and Cys motifs as critical therapeutic targets, leading to novel strategies—such as ASNase, synthetic agents and phytochemical combinations—for simultaneously blocking viral entry and modulating hyperinflammatory pathways. These findings warrant rigorous experimental and clinical validation to facilitate translation into effective therapeutic interventions.

**Supplementary Information:**

The online version contains supplementary material available at 10.1038/s41598-025-19359-y.

## Introduction

Extensive evidence indicates that the current generation has experienced Lifelong exposure to coronaviruses, with a notable rise in haematological and immune-related disorders since the 1960s. Successive coronavirus outbreaks—severe acute respiratory syndrome (SARS) in 2002 and Middle East respiratory syndrome (MERS) in 2012—preceded the emergence of the global COVID-19 Pandemic in late 2019, which was declared a pandemic by the World Health Organization (WHO) in March 2020. COVID-19 has since exerted a profound global impact, with 777,950,273 confirmed cases and 7,096,650 deaths reported worldwide, despite the administration of over 13.6 billion vaccine doses^1,2^.

In Egypt, 68,311 confirmed cases and 2,953 deaths were recorded by July 2020. By August 2020, critical cases had become rare in Alexandria and nationwide, as reflected in WHO reports [https://data.who.int/dashboards/covid19/data]. Currently, eight SARS-CoV-2 variants are reported by WHO, with JN.1 designated as a variant of interest due to its association with a ~ 10% rise in hospital admissions, while seven other variants remain under monitoring. Alarmingly, the most recent variant has shown increased severity in children and adolescents, necessitating admission to paediatric intensive care units^3^.

Beyond the acute phase, post-COVID-19 syndrome continues to impose a significant burden on healthcare systems, with persistent immune dysregulation leading to autoimmune conditions (e.g., rheumatoid arthritis, psoriasis), vascular complications, and psychological disorders among adolescents, including depression, loneliness, and anxiety^4,5^. According to the latest WHO epidemiological update, COVID-19 remains an ongoing global health threat, with hundreds of thousands of new cases and thousands of deaths recorded monthly—an increase of 28% in January 2025 compared to December 2024 [https://www.who.int/publications/m/item/covid-19-epidemiological-update-edition-177].

Collectively, these observations underscore that COVID-19 remains a persistent global health challenge, with profound clinical, societal, and economic repercussions. This reality demands unwavering surveillance and the accelerated development of innovative, targeted therapeutic strategies to mitigate both acute disease and its long-term sequelae.

Earlier research has highlighted COVID-19’s detrimental impact on multiple pillars of the Global Sustainable Development Goals (SDGs), particularly those related to health, education, and the economy, with recent evidence indicating that the pandemic has hindered progress toward all SDGs^6–8^. The severity and mortality associated with COVID-19 have been linked to haematological and autoimmune disorders, multi-organ dysfunction, pulmonary embolism, and thrombosis^9^. During the initial pandemic wave, the only approved immunotherapy for critically ill patients was the IL-6 inhibitor tocilizumab (Actemra)^10^; however, clinical observations revealed that some ICU patients exhibited normal IL-6 levels and did not respond to treatment, ultimately succumbing to the disease. This prompted us to investigate the role of IL-17, a cytokine that acts synergistically with IL-6, in COVID-19 pathogenesis. Concurrently, we noted that N-glycosylation on **Asparagine (Asn)** residues and disulfide bonds between **Cysteine (Cys)** residues are critical structural features governing the stability and function of the SARS-CoV-2 spike protein (S)^11^ and pro-inflammatory mediators including IL-6R^12^, IL-17R^13^, CD41/CD61^14^ and CD47/SIRP^15^ as well as mediating the interactions with host inflammatory mediators. These observations provided the basis for integrating clinical cytokine profiling with in-silico molecular docking, aiming to elucidate key molecular mechanisms and identify novel therapeutic targets.

The SARS-CoV-2 spike protein has a trimeric structure. Each monomer consists of two functional subunits: S1, which mediates the binding of the virus to human receptors such as hACE2, and S2, which facilitates fusion with the host cell membrane and subsequent cell invasion. The spike protein is characterized by extensive N- and O-linked glycosylation. These glycan residues play a key role in the binding affinity to host receptors and in immune evasion. Strong evidences revealed that the crucial role of N-glycans on Asn in controlling the conformational dynamic changes of spike protein “up” and “down”, subsequently, its binding affinity with hACE2. In addition, N-glycans at Asn343 form a shield that blocks approximately 40% of the protein surface from antibodies cloned from convalescent individuals, thereby protecting viral epitopes from neutralization^16,17^.

In response to stimuli, auto-immune disease or viral infection, Interleukin-17 (IL-17) binds to its receptor (IL-17R) initiating the inflammatory signaling pathway in fibroblasts, stromal, epithelial and endothelial cells, as well as on monocytes and platelets^18^. Previous studies have emphasized the essential role of integrins and integrin-associated proteins aIIBb3 and CD47/SIRP, respectively, in the regulation of the immune response. The former mediates many cellular functions including cell adhesion, proliferation, differentiation, migration and apoptosis^19^. CD47 is found either as a transmembrane protein on erythrocytes, thymocytes, T and B cells, monocytes, neutrophils as well as nerve cells or soluble form in plasma. It plays a crucial role in protecting RBC from elimination by macrophages, controlling RBCs agglutination and regulating the innate immune checkpoint^20^.

These events underscore the urgent need to evaluate the efficacy of safer natural agents that can help regulate the immune response by targeting elevated pro-inflammatory proteins. Our primary objective was to explore safe, precision therapies targeting key residues in the SARS-CoV-2 spike protein and human inflammatory mediators, with the aim of rescuing critically ill COVID-19 patients. To achieve our objective, we first assessed the levels of IL-17 and routine inflammatory markers, including IL-6, CRP, ferritin, and D-dimer, along with liver and kidney function tests, hematological parameters, and clinical findings. Bioinformatics has become an effective tool for exploring disease progression mechanisms and designing novel precision-targeted therapies, including drugs, immunotherapies, and vaccines, through drug screening, molecular docking, and epitope prediction. An in-silico study was also performed to examine the interactions between the SARS-CoV-2 spike protein (S) and human hemoglobin (Hb), as well as pro-inflammatory mediators such as IL-6R, IL-17R, CD41/CD61, and CD47/SIRPα. Then, we screened many large and small molecules to select therapies targeting key residues of receptors that lie at the binding interface with their ligand and/or B-cell epitopes. Biological therapies are the most recent revolution in the treatment and prevention of communicable and non-communicable diseases. They are based on engineering monoclonal antibodies which target specific antigenic epitopes on cell surface receptors or soluble ligands. Secukinumab, a monoclonal antibody to IL-17, was approved to treat immune disease such as Psoriasis^21^. Herein, we aimed to explore therapies which can efficiently interference with the binding of SARSCoV-2 spike protein from binding with human cell receptors that intermediate SARS-CoV-2-induced cytokine storm and inflammation. The current work bridges clinical observations with mechanistic insights for therapeutic discovery through a cross-sectional analysis of hematological derangements, biomarker dynamics, and chest imaging in COVID-19 patients (severe vs. moderate), coupled with in-silico modelling of SARS-CoV-2-driven inflammation and drug-target interactions. Thus, molecular docking was conducted for 28 therapeutic compounds [27 small molecules and one polypeptide (L-asparaginase, **ASNase**)] to the selected inflammatory mediators. Among these, sixteen small molecules are phytotherapies and four were synthetic. Most of the selected therapies, such as quercetin, apigenin, kaempferol, coumarin, and ferulic acid, are active ingredients of *Solanum nigrum*^22^.

## Materials and methods

### Patients and sampling

This study included 85 patients diagnosed with COVID-19 via real-time PCR at the Armed Forces Hospital in Alexandria, Egypt, between October 13, 2020, and August 31, 2021. The principle of RT-PCR is designed to cover 2 targets (N1 and N2) of the Nucleocapsid gene of SARS-CoV-2 detected with the same fluorescence channel. Based on the WHO Clinical Progression Scale (WHO, 2020), patients were categorized into two groups by CT and clinical Manifestations at admission. Group 1 included 47 moderate cases (WHO scores 4–5) in regular wards of chest department who had diagnosed with pneumonia but did not require oxygen supply (score 4) or required low-flow oxygen (SpO_2_ < 90%, score 5). Group 2 included 38 patients with severe illness (WHO scores 6–7) who referral to intensive care unit (ICU) with severe pneumonia, SPO2 < 85%, required high-flow oxygen, non-invasive ventilation (score 6), or mechanical ventilation (score 7) and had comorbidities.

To examine the validity, clinical relevance of our findings and ensure that the severe illness of COVID-19 persist, we examined the registered clinical and radiological findings from 20 COVID-19 patients admitted to different clinics and hospitals in Alexandria from October 2024 to January 2025. The study was reviewed and approved by the institutional review board of Pharos University in Alexandria, and all methods were performed in accordance with the ethical standards of the declaration of Helsinki. All patients provided written informed consent which was approved by the Ethics Committee of Pharos University in Alexandria (0420233263065). All methods followed the Declaration of Helsinki. Blood samples were collected for hematological and biochemical investigations including routine pro-inflammatory biomarkers, liver function and kidney function. All analysis was performed in technical duplicates to ensure reproducibility.

### Inclusion and exclusion criteria

The study included all confirmed COVID-19 patients who were diagnosed by RT-PCR and met the criteria of moderate and severe criteria [WHO/National guidelines]. We excluded: (1) suspected cases without RT-PCR confirmation, (2) patients with missing critical data (e.g., baseline CT imaging or laboratory results), (3) inadequate blood samples (< 5 mL) or samples failing quality control (hemolysis index > 50 mg/dL or visible clotting), and (4) individuals with contraindications to essential procedures (e.g., contrast allergy).

### Quantitative determination of interleukin-17 (IL-17) by ELISA

To evaluate the level of Interleukin-17 (IL-17) in the serum samples of COVID-19 patients, we used a double-antibody sandwich enzyme-linked immunosorbent assay (ELISA), according to the manufacturer’s instructions (SunRed, China, **cat. # 201-12-0143**). Briefly, 40 µL of the sample was added to each well, followed by 10 µL of IL-17 antibody and 50 µL of streptavidin-HRP. Plates were incubated at 37 °C for 60 min. Then, the wells were washed and 50 µl of chromogen solution A and 50 µl of chromogen solution B were added. After washing, we mixed, incubated for 10 min at 37 °C in the dark. Finally, added stop solution was added to each well and the optical density (OD) was measured at 450 nm within 15 min after the addition of stop solution.

### Measurement of routine biomarkers

We assessed the serum levels of routine pro-inflammatory markers including IL-6 using a chemiluminescence immunoassay (CLIA), according to the manufacturer’s instructions (Snibe, China). In addition, C-reactive protein (CRP), ferritin, D-dimer levels were assessed. On the basis of the reference ranges of routine markers, we analysed the data of biochemical markers including liver function including alanine transaminase (ALT) and aspartate transaminase (AST) as well as kidney function including urea and creatinine in COVID-19 patients according to the clinical guidelines of laboratory tests^23^.

### Microscopic examination of haematological parameters

Venous blood samples were collected from all patients on K2- EDTA (potassium salt of ethylene-diamine-tetra acetic acid) sterile vacutainers. Complete blood counts (CBCs) were performed on a Sysmex Xs I 500. Blood film smears were performed and stained with Leishman stain to differentiate and identify white blood cells. The microscopic examination of the blood films was performed in accordance with the established guidelines^24^.

### In silico study

#### Protein–protein interactions between SARS-CoV 2 and blood proteins

The first step in studying proteins was downloaded the PDB and FASTA forms from Protein Data Bank. There are many database sites include Protein Data Bank in Europe, Protein Data Bank Japan and Research Collaboratory for Structural Bioinformatics Protein Data Bank. All data banks have the same contents. We used PDBJ because of the easier presentation of structure and function of key residues that relevant to our objective^25^. Because mutations among SARS-CoV-2 variants continues, we analysed the alignment of 14 spike protein sequences of SARS-CoV-2 Spike protein (S) sequences published since 2016 to date using Clustal Omega program^26^ to verify that the selected therapies will have the same efficacy against a wide range of SARS-CoV variants. To explore novel mechanism of SARS-CoV-2-induced severe illness in COVID-19 patients, we studied the protein-protein-interaction (PPIs) between the SARS-CoV-2 S protein (pdb:7bnn) and hemoglobin (pdb: 1bz0) as well as inflammatory mediators including IL-6R (PDB: 1n26) and IL-6/IL-6R (PDB:1p9m), unliganded IL-17R (PDB:5n9b), IL-17 A/IL17RA (pdb:4hsa), IL-17 F/IL17RA (pdb:3jvf) IL-17AF/IL17RA (pdb:5nan), integrin αIIbβ3 (pdb:3fcs) and the human thrombospondin receptor CD47/SIRP (PDB:2jjs). Next, we studied the molecular docking of L-ASNase (PDB:1o7j) and the selected inflammatory mediators. To ensure the validity of the molecular docking, we compared our data with the structural of the selected proteins that published on the protein data bank. As IL-17 is the main target in our study, we compared our data with docking of Secukinumab Fab, a monoclonal antibody to IL-17 (pdb:6wir). To optimize the protein structure, Spdbv was used for energy minimization of the proteins^27^. We conducted the molecular docking of proteins via ClusPro, a clustering program used to evaluate the protein-protein interactions (PPIs), and PyMOL was used to visualize PPIs^28^. The Swiss model was used to remodel IL-17R^29^. To evaluate the accuracy of docking, root mean standard deviation (RMSD) was determined via TM-align.The binding affinity (ΔG) Kcal/mol and the dissociation constant (Kd) values were extracted by Prodigy^30^. Analysis of the drug docking of twenty-seven small molecules including sixteen natural therapies and ten synthetic therapies, of these eight were approved including remdesivir, dexamethasone, candesartan, enalapril, aminopterin, calcitriol, nitazoxanide, quinapril and molsidomine, in addition to the unapproved ones bromopyruvate and Zinc carried out on the studied proteins. We used iGemodock for drug docking^31^. Additionally, we determined the B-cell epitopes of the examined proteins that is very important for investigating their antigenicity against infected cells^32^.

### Statistical analysis

Statistical analysis was performed using IBM SPSS statistics, version 25.0 (IBM Corp., Armonk, NY, USA). A *t* test was used to compare the means of variables between COVID-19 patients who experienced severe illness and those who experienced moderate symptoms. Also, the Z test was used for analytic comparisons. All the quantitative data are presented as the mean ± SD. Categorical variables were assessed using the Chi-square test. Standard curve for calculating the concentration of IL-17 was generated from the Arigo Biolaboratories website (https://www.arigobio.cn/ELISA-calculator). The paired comparison of receiver operating characteristics (ROC) curve was performed via MedCalc version 23.0.8^33^. ROC curve was constructed to discriminate COVID-19 patients who experienced severe illness and those who experienced moderate symptoms to establish the sensitivity-specificity relationship. The ROC curve data provided the accuracy of each test with area under the curve (AUC) measurements and Cohen’s Kappa coefficient (K). The optimum cut-off values of all variables as well as the novel biomarker (IL-17) were determined and expressed as AUC. AUC values are reported with the 95% confidence intervals (CI) for specificity, sensitivity, positive predictive values and negative predictive values (PPV and NPV) and cut-off value. The odd ratio (OR) was used to calculate the strength of association between different confounding variables and clinical outcomes. *P* values ≤ 0.05 were considered statistically significant.

To assess the independent effects of haematological indices, inflammatory biomarkers, and hepatic/renal function on the clinical outcomes (disease severity), we performed logistic regression analysis adjusted for prespecified confounders including age. Assumptions (linearity, normality, homoscedasticity) were verified using residual plots and Shapiro-Wilk tests. A weighted least squares logistic regression was performed to identify predictors of disease severity (severe vs. moderate). Weights were assigned using the inverse of the squared standard deviation (1/SD²) to minimize heteroscedasticity. Multicollinearity was assessed using variance inflation factor (VIF), and model fit was evaluated through the coefficient of determination (R²) and adjusted R². The model employed forward selection. Variables with *P* < 0.1 were retained, and model fit was assessed using likelihood ratio tests, Nagelkerke’s *R*², and the Hosmer-Lemeshow test. Discrimination was evaluated via AUC-ROC analysis.

For in-Silico study, IGEMDOCK provides an empirical binding score, with more negative values indicating stronger predicted binding. The statistical analysis was applied to compare between the binding energy of natural vs. synthetic compounds to the various studied proteins. Binding energy (score) differences between natural and synthetic small molecules compounds were analyzed using the Mann-Whitney U test (two-tailed, α = 0.05) due to non-normality (Shapiro-Wilk *p* < 0.05) and unequal sample sizes. The effect size was estimated via Hodges-Lehmann median differences and rank-biserial correlation (r). Mean values with 95% confidence intervals (CI) were computed for each group. To aid in pharmacological interpretation, estimated binding free energies (ΔG) were derived from docking scores using the empirical relationship ΔG ≈ 0.1 × docking score. This facilitated relative potency comparisons. Predicted IC_50_ values were then estimated from ΔG using the formula:

IC50 = e ^ΔG/RT^ [in Molar units]^34^.

where *R* = 0.001987 kcal/(mol·K) (gas constant) and T = 298.15 K (standard temperature).

We converted M to nM: IC50 (nM) = IC50 (M) * 1e^9^.

Then, we calculated pIC50 = -log10(IC50) to have a more linear scale^35^.

## Results

### Severity and persistence of SARS-CoV-2

**Fig. 1:**
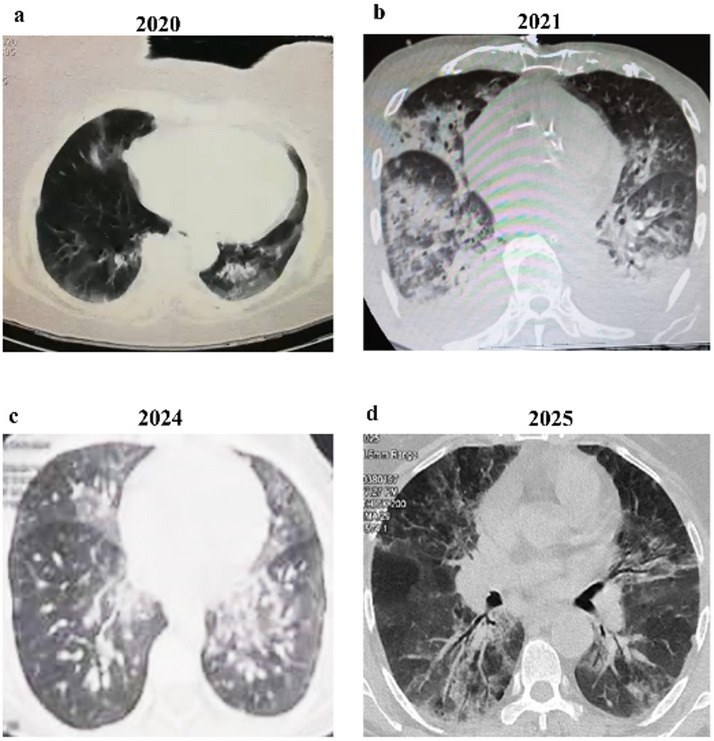
CT image showing GGO and consolidation (**a**), diffused bilateral GGO and consolidation (**b**), pulmonary lesions with a crazy paving pattern (**c**), tree-in-bud and bronchial wall thickening (**d**).

The diagnosis of COVID-19 patients was based on real-time-PCR of swab samples obtained from patients with symptoms plus chest CT scans and clinical Manifestations. On the basis of the CO-RAD classification, 85 COVID-19 patients from 2020 to 2021 were categorized into 47 moderate and 38 severe cases with CO-RAD6 rating where all of them had positive PCR results in addition to the typical CT findings of the chest from 2020 to 2021 whereas those from the end of 2024 to Jan. 2025 (Fig. 1) included diffused ground glass opacity (GGO), consolidations, broncho vascular interstitial thickening with air bronchogram. During the earlier wave, six out of forty-seven COVID-19 patients who were presented with moderate symptoms were outpatients while forty-one patients were admitted to regular wards while thirty out of thirty-eight COVID-19 patients who suffered from severe illness admitted internal chest unit while eight patients presented with many comorbidities. Of them, three patients were admitted to the cardiovascular intensive care unit, two were in oncology unit, one was in the brain and nervous unit, one was in hemodialysis unit and one in surgical unit. These patients suffered from fever, severe pneumonia, dry cough, low SPO2 (< 85%) and needed ventilation. One COVID-19 patient had a positive PCR result for 55 days and five deaths were recorded. During the first wave of COVID-19, the mean age of COVID-19 patients with severe illness was significantly higher (71.16 ± 9.52) than that of those with moderate symptoms (57.08 ± 12.8) years ± SD, *p* = 0.0001 (Table 1). An elevated level of D-dimer more accurately predicted severe illness in COVID-19 patients with a high specificity of 86.96, *p* = 0.0352 (Fig. S1). An elevated level of creatinine greater than 1.3 predicted the severe illness in COVID-19 patients with a high sensitivity of 76.60%, *p* = 0.0038 and significantly increased the risk factor for severe illness in COVID-19 patients 3.6-fold (Fig. S2).

**Table 1:** Comparison between the mean levels of bio- and haematological markers in moderate and severe cases of COVID-19 patients.

Clinical- pathological parameters	Moderate (*n* = 47) (mean ± SD)	Severe (*n* = 38) (mean ± SD)	*p* value
IL-17(ng/ml)	188.234 ± 61.206	279.5 ± 210.245	0.004*
IL-6(pg/ml)	58.627 ± 83.085	486.02 ± 1048.1	0.006*
Age (years)	57.08 ± 12.8	71.16 ± 9.52	0.0001*
Haemoglobin (g/dl)	12.1 ± 2.3	10.94 ± 2.54	0.030*
RBCs (×10^6^/mm^3^)	4.42 ± 0.97	3.88 ± 0.84	0.008*
Hct (PCV) (vol %)	34.94 ± 7.22	31.18 ± 7.20	0.019*
RDW (fl.)	12.1 ± 2.303	14.77 ± 4.22	0.048*
MCV(fl.)	79.38 ± 6.16	80.79 ± 7.82	0.356
MCH (pg)	27.638 ± 2.883	28.42 ± 4.03	0.316
MCHC (g/dl)	34.83 ± 1.98	35.18 ± 3.42	0.551
Leukocytes (×10^3^/mm^3^)	10.54 ± 6.02	13.4 ± 7.73	0.046*
lymphocytes(/µl)	1033.43 ± 729.2	957.84 ± 530.57	0.594
Neutrophils (/µl)	8940.21 ± 5739.5	11892.3 ± 7597.44	0.0444*
Monocytes (/µl)	407.389 ± 308.4	559.07 ± 395.7	0.050*
Platelets (×10^3^/mm^3^)	245.17 ± 145.71	206.89 ± 140.45	0.2245
MLR(%)	0.504 ± 0.404	0.724 ± 0.689	0.070
NLR(%)	12.302 ± 11.64	16.84 ± 12.6	0.0887
PLR(%)	331.1 ± 275.9	296.45 ± 299.47	0.5810
CRP (mg/L)	133.043 ± 153.5	134.68 ± 139.48	0.9595
Ferritin(ng/ml)	943.568 ± 929.3	945.9 ± 826.58	0.9904
D-dimer (µg/ml)	1.77 ± 2.403	2.63 ± 2.55	0.1142
ALT(U/L)	37.02 ± 32.204	46.815 ± 54.3	0.3046
AST(U/L)	43.78 ± 45.11	53.105 ± 52.17	0.3796
Urea (mg/dl)	86.89 ± 97.76	109.65 ± 90.73	0.2737
Creatinine (mg/dl)	1.43 ± 1.28	2.77 ± 3.12	0.0088*

*Statistically significant at *p* ≤ 0.05.

In the recent wave (2024–2025), no deaths were detected but severe illness including fever, severe pneumonia and bone pain was observed in all twenty current COVID-19 patients. Their CT revealed GGO and consolidation but no need to hospitalization. Two children were admitted to the paediatric intensive care unit and one elderly patient needed mechanical ventilation in the ICU. We observed that the recent wave attacked children, adults and elderly individuals, while aging was the highest risk factor for severe illness during the first wave where patients who were older than 57 years had a 12-fold greater risk for severe illness, *p* = 0.0002.

### Hematological anomalies associated with COVID-19

The mean levels of hemoglobin, RBCs count and Hct were significantly lower in COVID-19 patients with severe illness compared to those with moderate symptoms (Table 1). Additionally, decreasing the mean RBCs count and Hct to less than 3.3 × 10^6^/mm^3^ and 27.2 vol% predicted the worst clinical outcomes in COVID-19 patients with a high specificity 89.4% and high specificity 93.6%, *p* = 0.0047 and 0.0390, respectively, (Table 2, Fig. S3). Additionally, decreases in hemoglobin, RBCs count and Hct were more frequent in severe COVID-19 patients than in moderate patients and significantly increased the risk factor of severe complications (6.8-, 4.9- and 8.5-fold, *p* = 0.0057, 0.0062 and 0.0017, respectively) (Table 3).

**Table 2:** Discrimination between COVID-19 patients who suffered from severe illness and moderate cases.

Variables	AUC	*p*	95% C.I	Cut off	Sensitivity	Specificity	PPV	NPV
IL-17 (ng/ml)	0.685	0.0022*	0.575 to 0.781	187.9	63.16	76.60	68.5709	72.0071
IL-6 (pg/ml)	0.653	0.0117*	0.542 to 0.753	48.3	55.26	72.34	61.0853	67.2975
Age	0.789	< 0.0001*	0.687 to 0.870	57	92.11	51.06	59.6578	89.1733
Haemoglobin (g/dl)	0.607	0.089	0.493 to 0.709	9.1	31.6	93.6	79.5466	63.5235
RBCs (×10^6^/mm^3^)	0.669	0.004*	0.557 to 0.766	3.3	36.8	89.4	73.1216	64.2944
Hct (PCV) (vol %)	0.629	0.038*	0.517 to 0.731	27.2	36.8	93.6	81.9395	65.3562
RDW (fl.)	0.505	0.933	0.404 to 0.624	13.5	31.6	59.6	38.0315	52.5640
MCV(fl.)	0.565	0.312	0.449 to 0.669	85	34.2	91.5	75.9532	63.8976
MCH (pg)	0.566	0.300	0.462 to 0.681	30	26.3	91.49	70.8462	61.2459
MCHC (g/dl)	0.518	0.770	0.398 to 0.619	36	89.5	21.3	47.1741	72.0048
leukocytes (×10^3^/mm^3^**)**	0.624	0.050*	0.512 to 0.727	13.7	44.7	87.2	73.3530	66.7669
Lymphocytes(/µl)	0.501	0.982	0.393 to 0.614	601	26.32	61.70	35.0627	51.5923
Neutrophils (/µl)	0.635	0.030*	0.513 to 0.727	11,174	50.00	85.11	72.5154	68.4188
Monocytes (/µl)	0.617	0.0617	0.506 to 0.721	250	78.9	42.6	51.9175	72.0097
Platelets (×10^3^/mm^3^**)**	0.579	0.213	0.467 to 0.686	182	57.89	63.83	55.7039	65.8609
NLR(%)	0.606	0.093	0.484 to 0.701	14.66	52.63	72.34	59.9201	66.0282
MLR(%)	0.604	0.097	0.494 to 0.711	0.434	63.2	66.0	59.3143	69.5006
PLR(%)	0.572	0.256	0.460 to 0.679	135.85	42.1	80.9	63.3398	63.9965
CRP (mg/L)	0.543	0.4970	0.431 to 0.651	20	97.37	19.15	48.6194	90.2602
Ferritin (ng/ml)	0.517	0.7902	0.405 to 0.627	1013	39.47	71.74	52.3216	60.1345
D-dimer (µg/ml)	0.630	0.0352*	0.518 to 0.733	2.7	42.11	86.96	76.7196	66.4181
ALT (U/L)	0.500	1.0000	0.390 to 0.610	73	18.42	95.74	77.2592	59.8979
AST (U/L)	0.587	0.1774	0.474 to 0.692	33	65.79	59.57	56.1126	68.9075
Urea (mg/dl)	0.591	0.1552	0.479 to 0.696	76	57.89	63.83	55.7039	65.8609
Creatinine (mg/dl)	0.674	0.0038*	0.564 to 0.772	1.3	52.63	76.60	63.8622	67.2997

*AUC* Area Under a Curve, *p value* Probability value, *CI* Confidence Intervals, *NPV* Negative predictive value, *PPV* Positive predictive value.

*Statistically significant at *p* ≤ 0.05.

**Table 3:** Association between the Clinical- pathological characteristic features and severity of COVID-19 patients (*n* = 85).

Clinical- pathological features	No. of cases *N* = 85	Moderate (*n* = 47)	Severe (*n* = 38)	Odd Ratio (OR)	95%CI	Z	*p* value
IL-17 (ng/ml) ≤ 187.9 > 187.9	50 35	36 11	14 24	5.6104	2.1837 to 14.4145	3.582	0.0003
IL-6 (pg/ml) ≤ 48.3 > 48.3	51 34	34 13	17 21	3.2308	1.3082–7.9791	2.542	0.0110
Age (years) ≤ 57 > 57	27 58	24 23	3 35	12.1739	3.2832–45.1408	3.738	0.0002
Hemoglobin (g/dl) ≤ 9.1 > 9.1	15 70	3 44	12 26	6.7692	1.7463–26.2398	2.766	0.0057
RBCs(×10^6^/mm^3^**)** ≤ 3.3 > 3.3	19 66	5 42	14 24	4.9000	1.5708–15.2852	2.738	0.0062
Hct (PCV) (vol %) ≤ 27.2 > 27.2	17 68	3 44	14 24	8.5556	2.2346–32.7559	3.134	0.0017
Leukocytes(×10^3^/mm^3^**)** ≤ 13.5 > 13.5	60 25	39 8	21 17	3.9464	1.4609–10.6611	2.708	0.0068
Monocytes (/µl) ≤ 249 > 249	28 57	20 27	8 30	2.7778	1.0521–7.3341	2.062	0.0392
Neutrophils(/µl) ≤ 11,174 > 11,174	59 26	40 7	19 19	5.7143	2.0517–15.9153	3.335	0.0009
Lymphopenia No lymphopenia	55 30	26 21	29 9	2.6026	1.0132–6.6852	1.987	0.0469
Leucocytosis No leucocytosis	28 57	10 37	18 20	3.3300	1.2940–8.5696	2.494	0.0126
D-dimer (µg/ml) ≤ 2.7 > 2.7	64 21	41 6	23 15	4.4565	1.5200-13.0665	2.723	0.0065
Creatinine (mg/dl) ≤ 1.3 > 1.3	52 33	35 12	17 21	3.6029	1.4418–9.0035	2.743	0.0061

Data are presented as counts (percentages) or odds ratios (ORs) with 95% confidence intervals (CIs). Statistical significance was assessed via two-tailed z-tests (*p* ≤ 0.05 considered significant).

**Fig. 2:**
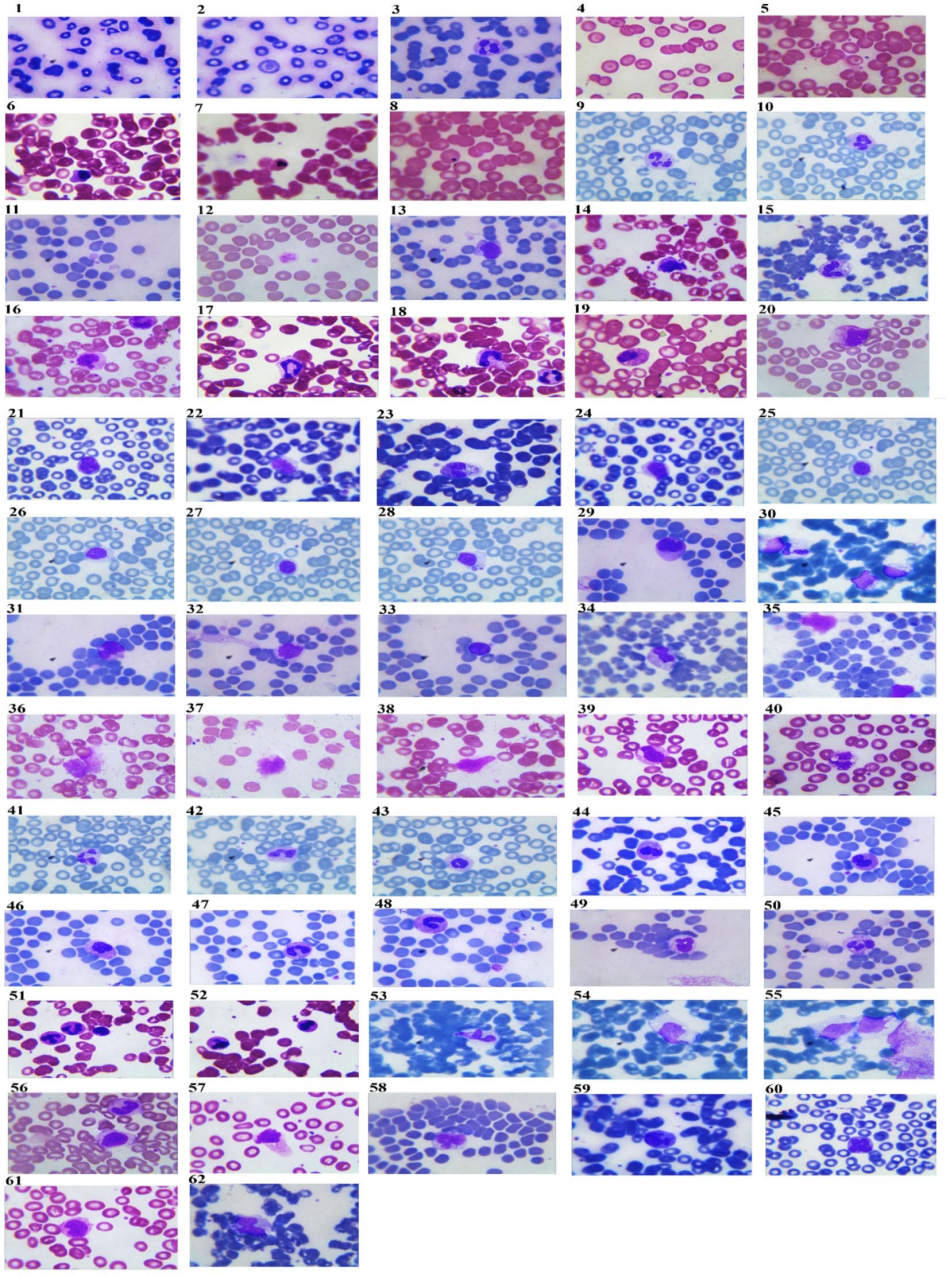
Blood smear from COVID-19 patients showed teardrop cell (1), teardrop and target cell (2), echinocyte, RBCs rouleaux (3), target cells and elliptocyte (4), target cells (5), RBCs rouleaux and agglutination (6,7), Thrombocytopenia (8–10), thrombocytopenia with macrothrombocyte (11,12), plasmacytoid lymphocyte with acanthocytes RBCs (13), acanthocytes RBCs, elliptocytes and rouleaux RBCs with atypical lymphocytes (14), acanthocytes RBCs with vacuolated neutrophil (15), blister cell with atypical neutrophil (16), rouleaux RBCs with neutrophilic Toxic granules and thrombocytopenia (17), RBCs clumps and neutrophil with toxic granules (18), rouleaux RBCs and atypical neutrophile with toxic granules (19), elliptocytes with atypical lymphocyte (20), atypical lymphocyte (21–24), elliptocytes and stimulated lymphocytes (25–28), large granular lymphocyte with indented nuclei (29), reactive large granular lymphocyte (30), atypical lymphocytes (31), large atypical lymphocyte (32), atypical lymphocyte with thrombocytopenia (33), RBCs agglutination and monocyte with cytoplasmic vacuole (34), atypical monocyte (35), smudge cells with schistocytes (36,37) smudge cell with bite cell (38), vacuolated neutrophil with thrombocytopenia (39), vacuolated neutrophil (40), elliptocytes and thrombocytopenia with normal neutrophil (41), acanthocytes RBCs and hyposegmented neutrophil (42), hyposegmented neutrophil (43), rouleaux RBCs and acanthocytes RBCs with hyposegmented neutrophil (44), hyposegmented neutrophil (45), pseudo-pleger-Huet neutrophil with thrombocytopenia (46), binucleated neutrophil (47), staff cell with giant platelet (48), atypical neutrophil (49), vacuolated neutrophil with toxic granules (50), RBCs rouleaux and atypical neutrophil with toxic granules (51), RBCs clumps with atypical neutrophil (52), RBCs agglutination with atypical monocytes (53–55), tear-drop cell with normal monocyte (56), elliptocytes with vacuolated monocyte (57), activated monocytes (58), rouleaux RBCs with activated monocyte (59), elliptocytes with activated monocyte (60), atypical monocyte (61), acanthocyte RBCs with atypical monocyte (62).

Microscopic examination of blood films revealed that many blood cells anomalies such as teardrop with fragmented RBCs, teardrop, elliptocytes, acanthocytes, target cells, RBCs rouleaux and RBCs aggregation were more frequent in COVID-19 patients who suffered from severe illness. Concurrently, we found that RDW increased significantly *p* = 0.048 in severe vs. moderate cases. Although there was no significant difference in either the absolute count of platelet or PLR between the critical and moderate cases of COVID-19 patients, thrombocytopenia, macrothrombocytes with toxic granules were more frequent in COVID-19 patients who suffered from severe illness. Additionally, many morphological abnormalities of lymphocytes such as reactive large granular with indented nuclei lymphocyte, stimulated lymphocyte, atypical, plasmacytoid and smudge cells were observed. Additionally, atypical and vacuolated monocytes were noticed. Moreover, numerous neutrophil abnormalities were observed including left shift which appeared as increasing of immature neutrophils such as neutrophil staff cell, giant, binucleated and vacuolated neutrophils with thrombocytopenia and toxic granules. In addition, pseudo-pleger-Huet neutrophil was high frequent among severe cases (Fig. 2).

The data of blood indices revealed a significant increase in the absolute values of total leukocytes, neutrophils and monocytes in critical cases of COVID-19 patients without significant change in the mean level of lymphocytes compared to those with moderate symptoms (Table 1). An absolute value of total leukocyte and neutrophil counts greater than 13.7 cells (×10^3^/mm^3^) and 11,174 cell (/µl) predict poor prognosis in COVID-19 patients with moderate sensitivity (44.74% and 50.00%) and high specificity (87.23% and 85.11%), *p* = 0.0488 and 0.030, respectively. A significant increase in the absolute monocytes count above 249 (cells/µl), *p* = 0.05, predicted severe illness in COVID-19 patients with high sensitivity 78.95% and moderate specificity of 42.55%, (Table 2, Fig S4).

### Therapeutic strategies to block SARS-CoV-2-S-Hemoglobin binding

Bioinformatic analysis provided us with many possible mechanisms of the observed blood anomalies as well as the efficacy of the selected therapies in targeting either the spike protein and/or human pro-inflammatory mediators. First of all, the data of the alignment of fourteen spike proteins (S) that were published since 2016 to date revealed that Asn and Cys are among the most conserved residues (Supplementary appendix 1). Particularly, the identity percent between the SARS-CoV-2 Spike protein variant D614G (7bnn) which released on data bank on 3rd February 2021 and Spike protein of SARS-CoV-2 JN.1 (8 × 4 h) which released on data bank on 3rd July 2024 was 94.51%, Asn and Cys are highly conserved. (Supplementary Notepad 1 and Supplementary Figure W1).

**Fig. 3:**
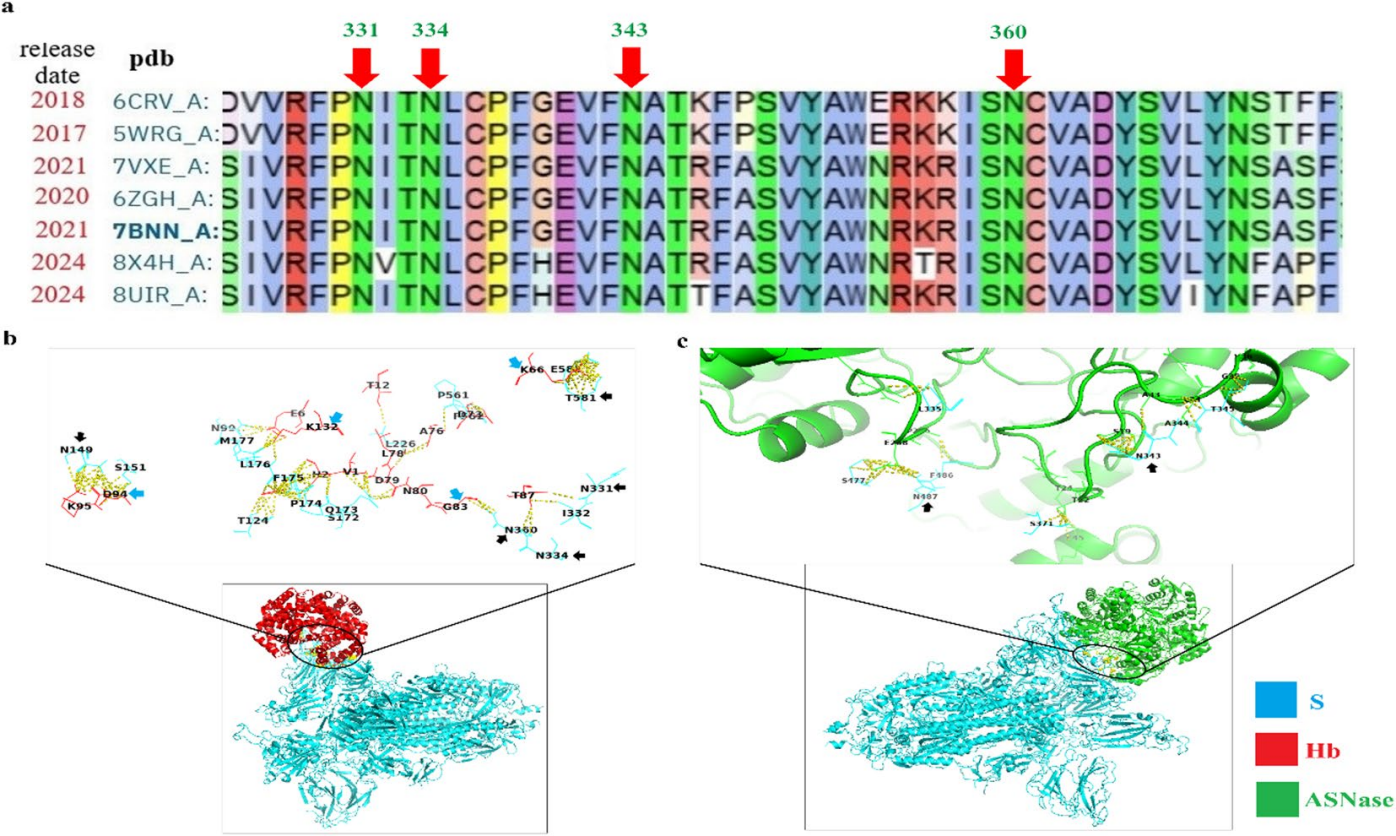
(**a**) alignment of 7 sequences of spike protein of SARS-CoV variants showing the highly conserved Asn residues N331 N334 N343 N360, (**b**) 2D image (upper) showing the key residues N149 N331 N334 N360 T581 of SARS-CoV-2 S (7bnn) target critical ones of Hb K66 G83 D94 K132 and 3D image (bottom) showing the binding interface between S and Hb, (**c**) 2D (upper) showing ASNase targets key glycan residues N343 and N487 of SARS-CoV-2 S (7bnn) and 3D image (lower) showing the binding interface between ASNase and S.

We noted that SARS-CoV-2 spike protein targeted critical residues of Hb (**K66 K132 G83 D94**) via its highly infectivity tools the highly conserved **glycosylated Asn N149 N331 N334 N360** and **T581**. Spike protein (7bnn) exhibited high affinity with ΔG equal − 33.0 kcal mol^−1^, Kd (5.9e-25 M) towards Hb (1bz0) (Supplementary Table W1) causing disruption of Hb structural disruption as RMSD was 6.46 Å. Subsequently, Spike protein enables to disrupt the Hb architecture as RMSD between 2 and 3 indicated conserved structure architecture. Surprisingly, a promising finding was that L-asparaginase (**ASNase**) can target key residues of the RBD, especially, invariant **Asn** **N343** and **N487** **(**Fig. 3). Also, ASNase targeted S371. ASNase exhibited high affinity towards SARS-CoV-2 spike protein (pdb:7bnn) where ΔG equal − 25.0 kcal mol^−1^, Kd (4.6e-19 M) (Supplementary Table W1) and RMSD was 7.26 Å, therefore, ASNase enables to disrupt the Spike protein architecture.

**Fig. 4:**
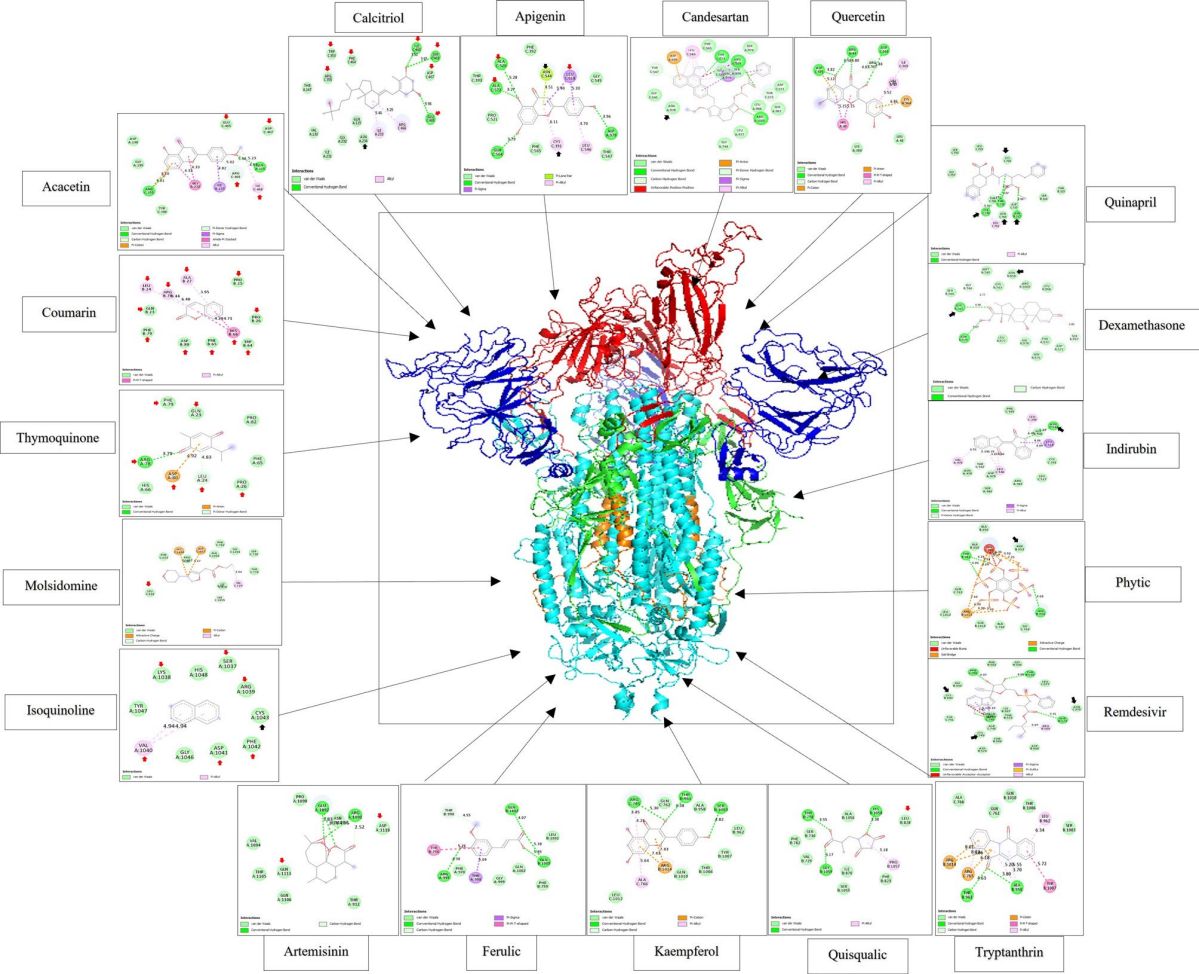
Drugs docking of SARS CoV-2 Spike protein (S) showing the key target amino acids residues. Quinapril, indirubin, apigenin, calcitriol target the key residues maintaining S architecture and function including (**Asn317**,** Cys738**,** Asn760**,** Asn764**), (**Cys391**,** Asn544**,** Asn978**), (**Cys391**,** Asn544**), (**Asn234**), and (**Asn 953**), respectively, (black arrow). Apigenin, calcitriol, acacetin coumarin, thymoquinone, artemisinin, isoquinoline and quisqualic bind to residues that lie within the predicted B-cell epitopes of S (red arrow).

Another novel finding was that the screened small molecules compounds are efficiently targeted the head of SARS-CoV-2 S (RBM), its trunk and tail as well as they targeted important B-cell epitopes (Fig. 4). These effects may subsequently interfere with the binding of the spike RBM to its receptor on blood cells. We observed that tryptanthrin, kaempferol, indirubin, calcitriol, quercetin, quinapril, apigenin, acacetin and isoquinolone had high affinities for SAR-CoV-2 S. Specifically, quinapril, indirubin, apigenin, calcitriol and phytic acid can destabilize the S architecture via targeting the key residues in S including (**Asn317**,** Cys738**,** Asn760**,** Asn764**), (**Cys391**,** Asn544**,** Asn978**), (**Cys391**,** Asn544**), (**Asn234**), and (**Asn953**), respectively. Additionally, apigenin, calcitriol, acacetin coumarin, thymoquinone, artemisinin, isoquinoline and quisqualic bind to residues that lie within the B-cell epitopes of the spike protein.

### The role of IL-17 and IL-6 in the pathogenesis of SARS-CoV-2-Induced cytokine storm

**Fig. 5:**
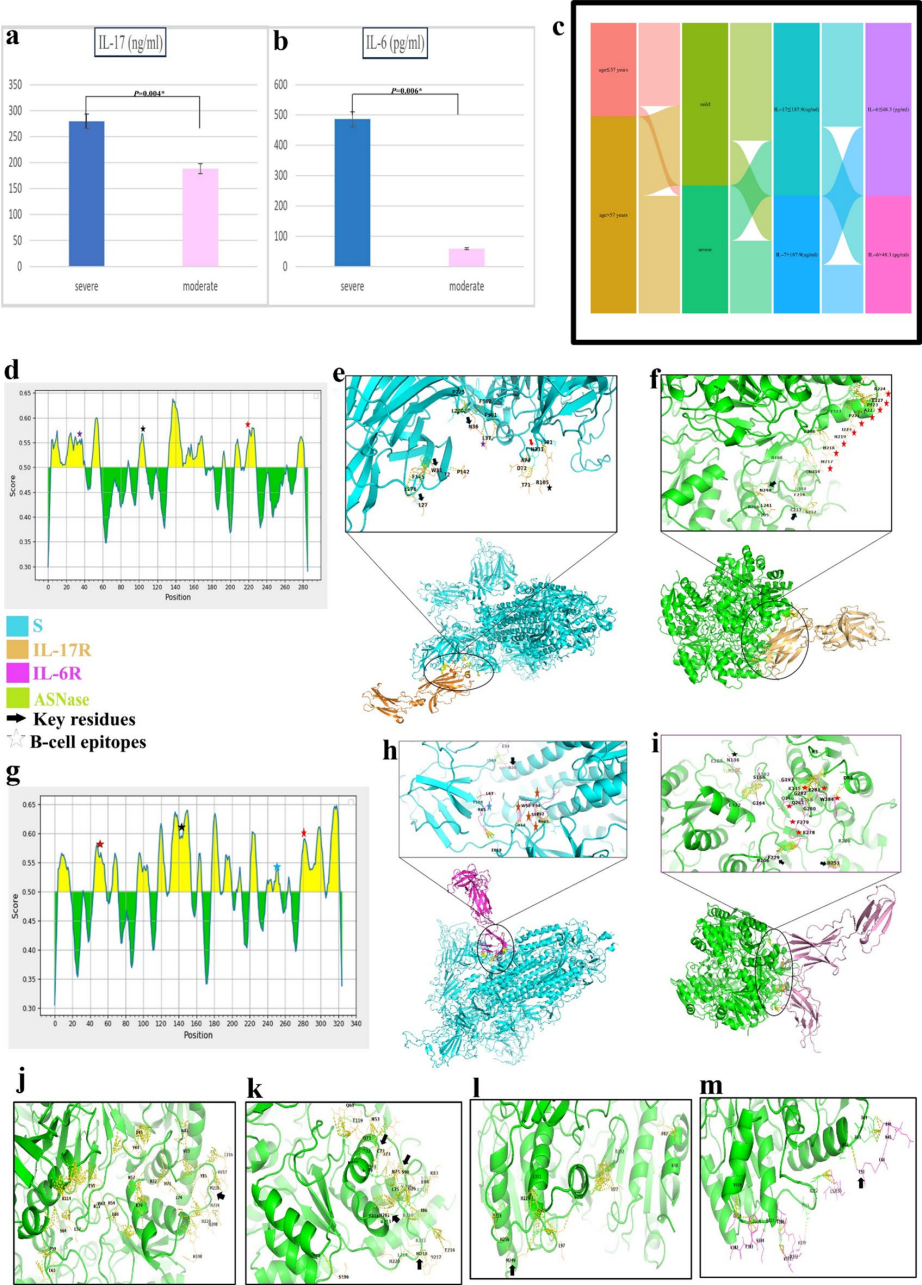
(**a**) Histogram chart compare between the mean level of serum IL-17 among severe and moderate cases of COVID-19 patients, (**b**) Histogram chart compare between the mean level of serum IL-6 among severe and moderate cases of COVID-19 patients, (**c**) Alluvial plot depicting the pattern of COVID-19 severity in relationship to age, serum levels of IL-17 and IL-6, (**d**): graph showing the peak of the B-cell epitopes on IL-17R (5n9b), (**e**) 3D image showing PPIs between SARS-CoV-2 S and IL-17R (bottom) focusing on the binding interface between SARS-CoV-2 S and IL-17R (top), (**f**) 3D image showing PPIs between ASNase and IL-17R (bottom) focusing on the binding interface between ASNase and IL-17R (top), (**g**) graph showing the peak of the predicted B-cell epitopes on IL-6R (1n26), (**h**) 3D image showing PPIs between SARS-CoV-2 S and IL-6R (bottom) focusing on the binding interface between SARS-CoV-2 S and IL-6R (top), (**i**) 3D image showing PPIs between ASNase and IL-6R (bottom) focusing on the binding interface between ASNase and IL-6R (top), (**j**) 3D image showing PPIs between ASNase and IL-17 A/IL-17RA (4hsa), (**k**) 3D image showing PPIs between ASNase and IL-17 F/IL-17RA (3jvf), (**l**) 3D image showing PPIs between ASNase and IL-17AF/IL-17RA (5nan), (**m**) 3D image showing PPIs between ASNase and IL-6/IL-6R (1p9m).

The mean levels of serum IL-17 and IL-6 were increased significantly in COVID-19 patients who experienced severe illness (279.5 ± 210.245 ng/ml and 486.02 ± 1048.1 pg/ml) compared to those who experienced moderate symptoms (188.234 ± 61.206 ng/ml and 58.627 ± 83.085 pg/ml), *p* = 0.004 and *p* = 0.006, respectively, (Fig. 5a-c). The minimum detectable limit of IL-17 is 0.012 ng/ml with intra-assay and inter-assay less than 8% and 10%, respectively. The minimum detectable limit of IL-6 is 15 pg/ml with intra-assay and inter-assay less than 5% and < 10%, respectively. The ROC curve showed that an elevated IL-17 level greater than 187.9 ng/mL predicted poor clinical outcomes in COVID-19 patients with higher probability of disease progression and severe complications (AUC = 0.685, 95% CI = 0.575 to 0.781, *p* = 0.0022 with a sensitivity of 63.16%, specificity of 76.60%, PPV of 68.5709%, NPV of 72.0071%). Also, ROC curve showed that an elevated level of IL-6 greater than 48.3 pg/ml predicted poor clinical outcomes in COVID-19 patients with higher probability of disease progression and severe complications (AUC = 0.653, 95% CI = 0.542 to 0.753, *p* = 0.0117 with a sensitivity of 55.26%, specificity of 72.34%, PPV of 61.0853%, NPV of 67.2975%). The regression analysis of IL-17 to predict the severity of disease showed that the regression equation is *y* = 188.2340 + 91.2660 *x*, where *y* represents IL-17 and *x* is the severity of disease. The model yielded a coefficient of determination (R²) of 0.0617, indicating a weak association. The residual standard deviation was 1.5562. Both intercept and slope were statistically significant (*p* < 0.0001) and (*p* = 0.0218), respectively, suggesting a modest but significant increase in IL-17 levels with increasing disease severity. However, the residuals violated the assumption of normality (*p* < 0.0001; Chi-squared = 71.828, df = 9). The F-ratio for the model was 5.4660, indicating the overall regression model was statistically significant.

Regression analysis was performed to assess the relationship between IL-6 levels and disease severity. The resulting regression equation was: y = 58.6277 + 427.3901x, where *y* represents IL-6 and *x* is the disease severity. The model showed a coefficient of determination (R²) = 0.0723, indicating a weak explanatory power. The residual standard deviation was 1.5074. Both intercept and slope were statistically significant (*p* < 0.0001) and (*p* = 0.0128), respectively. However, the residuals did not follow a normal distribution (*p* < 0.0001; Chi-squared = 133.033, df = 5), and the F-ratio for the regression model was 6.474.

### Logistic regression analysis for the studied markers

The study included 85 patients (severe: 38 [44.7%]; non-severe: 47 [55.3%]). While univariate analyses linked IL-6 and IL-17 to severity (both *P* < 0.05), these associations were nonsignificant (*P* > 0.1) in the adjusted model, likely due to confounding by age/monocytes or collinearity with other biomarkers (Supplementary Table R1).

A logistic regression model was constructed to identify independent predictors of disease severity in a cohort of 85 patients, of whom 44.7% (*n* = 38) had severe disease. Among the candidate biomarkers, age and monocyte count emerged as significant predictors. Age showed a strong, independent association with severity, with each additional year increasing the odds of severe disease by 14% (adjusted OR: 1.14; 95% CI: 1.07–1.20; *P* < 0.001). Monocyte count also contributed independently to severity prediction (adjusted OR: 1.002 per unit increase; 95% CI: 1.001–1.004; *P* = 0.013). The final model demonstrated excellent discrimination with an area under the ROC curve (AUC) of 0.86 (95% CI: 0.77–0.92), and accounted for 45% of the variance in disease severity as indicated by Nagelkerke’s R². The model fit was statistically significant (Likelihood ratio χ² = 35.20, *P* < 0.001), and calibration was marginally acceptable (Hosmer–Lemeshow χ² = 15.56, *P* = 0.049). Inflammatory biomarkers (CRP, IL-6), hematologic indices (NLR, PLR), and organ function markers (ALT, creatinine) did not significantly contribute to the model (*P* > 0.1 for all), supporting the model’s clinical applicability despite its modest explanatory power for unmeasured confounders.

The current data showed that IL-17 has the same potential of IL-6 in distinguishing between severe and moderate cases where AUC of IL-17 and IL-6 showed insignificant difference. Additionally, the specificity of IL-17 test was higher than that of IL-6 test (76.60% vs. 72.34%) while both exhibited moderate sensitivity (63.16% and 55.26%), respectively, (Table 2, Fig. S1).

There is no correlation between IL-17 and IL-6 where the correlation coefficient *r* = −0.052, 95% Confidence interval for *r* = −0.2626 to 0.1625. Therefore, assessment of serum level of IL-17 test may be a useful prognostic marker as well as help in improving the clinical outcomes of COVID-19 patients via the addition of targeted immunomodulatory therapy to the treatment protocol. Additionally, elevated levels of IL-17 and IL-6 significantly increased the risk factor of severe illness in COVID-19 patients by approximately 5.6- and 3.2-fold (*p* = 0.0003 and *p* = 0.011, respectively).

### Targeted therapies counteract PPIs between SARS-CoV-2 and interleukins signals

To identify potential novel inhibitors of the IL-17 and IL-6 signalling pathways, we first conducted a bioinformatic analysis of the protein–protein interactions (PPIs) between the SARS-CoV-2 spike (S) protein and IL-17R and IL-6R and then examined the drug docking of twenty-seven drugs against IL-17R and IL-6R either alone or in complex with their ligands. SARS-CoV-2 S (pdb:7bnn) had had high affinity for IL-6R (1n26) followed by IL-17R (5n9b).

**T581** and **N331** of **SARS-CoV-2 S** lie within the binding site with **IL-17R** and target critical residues of IL-17R particularly **L37 R105** which are highly predicted B-cell epitopes and **L27 W31** which lie within one of the three crucial sites of binding interface with IL-17 (Fig. 5d, e). Another superior result was that SARS-CoV-2 S targeted **N36** of both IL-17R and IL-6R which is one of N-glycosylation sites for both. On the other hand, ASNase docked IL-17R with high affinity and very low ΔG equal − 26.1 and Kd 7.6e-20 M compared to that of IL-6R ΔG (−8.5 Kcal mol^−1^)/Kd (5.4e-07 M), (**Supplementary Table W1**). As shown, the binding energy between ASNase and IL-17R is much lower than that between Spike protein and IL-17R, indicating a higher binding affinity of ASNase to IL-17R compared to the spike protein. ASNase binds to critical residues of IL-17R including **C213** and **N240** that lie at the binding interface with its ligand IL-17 (Fig. 5f).

**SARS-CoV-2 S** docked many residues lie within the B-cell epitopes of IL-6R (1n26) including **P52 S53 R54 W55** and **R65**, (Fig. 5g, h). While ASNase binds efficiently to many residues of IL-6R, especially, ** N136 G164 S166 F229 Y230 R231** **R233** **D253** **R274** **E278 F279 G280** E283 **W284** which lie within the binding interface with IL-6 (Fig. 5i).

To assess the potential of ASNase in mitigating the cytokine storm associated with SARS-CoV-2 infection—or, more broadly, in autoimmune conditions characterized by interleukin receptors bound to their respective ligands—we performed molecular docking analyses of ASNase with various IL-17R and IL-6R ligand–receptor complex structures. We found that ASNase targeted many critical residues in IL-17 A/IL-17RA (4hsa) that lie within the binding interface between IL-17 and IL-17R, especially, **Pro59** and **Met218 (**Fig. 5j**)**. Additionally, ASNase binds to many critical residues especially **C72 S90 M218 N261** of IL-17 F/IL-17RA (3jvf) as well as Pro82 **Met249** of IL-17AF/IL-17RA (5nan) (Fig. 5k, l). Our data is in line with the published structure of IL-17 and their complex structure on the data bank. Surprisingly, Secukinumab binds to both unliganded IL-17R as well as the liganded forms at critical residues that lie within the binding interface with IL-17 such as M197 and Asn225 of IL-17R (5n9b), M249 H251 N292 of IL-17/IL-17R (5nan), and R42, R102 of IL-17 in complex form (3jvf) (Supplementary Figure W3-W6). Moreover, ASNase docked with IL-6/IL-6R (1p9m) by binding many residues especially, Cys50 (Fig. 5m).

Docking analysis revealed that apigenin, kaempferol, molsidomine, and ferulic acid exhibited the highest predicted affinities for IL-6R (1n26), followed by IL-17R (5n9b) (Supplementary Table R2). Several ligands—including acacetin, apigenin, amygdalin, calcitriol, ferulic acid, kaempferol, candesartan, remdesivir, and phytic acid—interacted with unliganded IL-17R (5n9b) via multiple amino acid residues, notably **Cysteine**. While amygdalin, calcitriol, ferulic acid, molsidomine, and phytic acid targeted **Asparagine** residues. Dexamethasone and Nitazoxnide binds to both Asn and Cys (Supplementary ppt.1).

Regarding IL-17 A/IL-17RA (4hsa), apigenin, coumarin, indirubin, isoquinoline, kaempferol, thymoquinone, and tryptanthrin bound key residues, particularly Trp31 of IL-17RA and Pro59 and Arg101 of IL-17. Also, remdesivir binds to Trp31 of IL-17RA and to Arg101 of IL-17 (Supplementary ppt.2). Acacetin, calcitriol, and ferulic acid targeted Cysteine residues which are critical for IL-17R stability, while molsidomine and quisqualic acid bound to (**Asp29**,** Ser30**) and **Cys259** of IL-17, respectively—interactions predicted to destabilize IL-17R structure, disrupt ligand binding, and impair downstream signalling. Aminopterin binds to Asn67 and Cys137 of IL-17AF/IL-17RA (5nan) and **Cys259**,** Cys263** of IL-17 F/IL-17RA (3jvf). Nitazoxanide and Remdesivir bind to (**Asn79**) and (**Asn79**,** Asn211 His212**). Enalapril binds to **Trp31**,** pro60**,** Arg102** IL-17 F/IL-17RA (3jvf). Dexamethasone binds to **Cys154, Cys259 and Cys263 of** IL-17 F/IL-17RA (3jvf). Of note, acacetin uniquely binds to **Asn261**, a residue essential for N-glycosylation of IL-17R, whereas calcitriol and remdesivir targeted **His212**, a key site for IL-17R subunit dimerization. These interaction patterns were similarly observed in docking to IL-17 F/IL-17RA (3jvf) and IL-17AF/IL-17RA (5nan), suggesting a shared mechanism of receptor modulation across IL-17 family complexes, as shown in Supplementary ppt.3 and Supplementary ppt.4, respectively.

Regarding to small molecules docking of IL-6R, the small molecules compounds exhibited more than one mechanism. Ferulic, kaempferol and quercetin targeted **Cys**, thus they can disrupt IL-6R the architecture (Supplementary ppt.5). Kaempferol, phytic, quercetin, quinapril and tryptanthrin bind to Many residues including while tryptanthrin binds to critical residues in domain 2 which lies at the binding interface, in addition to **Lys133 Gln135 Asn136** which lie within the highest predicted **B-cell epitope**s. Additionally, acacetin, apigenin, artemisinin, amygdalin, calcitriol, coumarin, indirubin, isoquinolone, molsidomine, quisqualic and thymoquinone bind to Many amino acid residues which Lie within B-cell epitope extending from 117 to 125. Acacetin, phytic, quercetin and Quinapril bind to many amino acids residues especially those lie within the B-cell epitopes, which extends from **Met250 to Val259**.

Except Zinc and ascorbate, all examined drugs showed high affinity towards IL-6/IL-6R (1p9m). Of these, kaempferol, quercetin and tryptanthrin bind to **Cys150** and **Cys160** whereas acacetin and remdesivir bind to **Asn135**, molsidomine binds to **Asn136**, aminopterin and apigenin bind to **Asn224**,** Trp249**,** Arg259**, candesartan, indirubin, nitazoxanide and enalapril bind to **Asn224** of IL-6R while thymoquinone and artemisinin binds to **Arg168** and **Cys50**, respectively, of IL-6 (Supplementary ppt.6).

### Impact of SARS-CoV-2 on integrin and integrin-associated protein

**Fig. 6:**
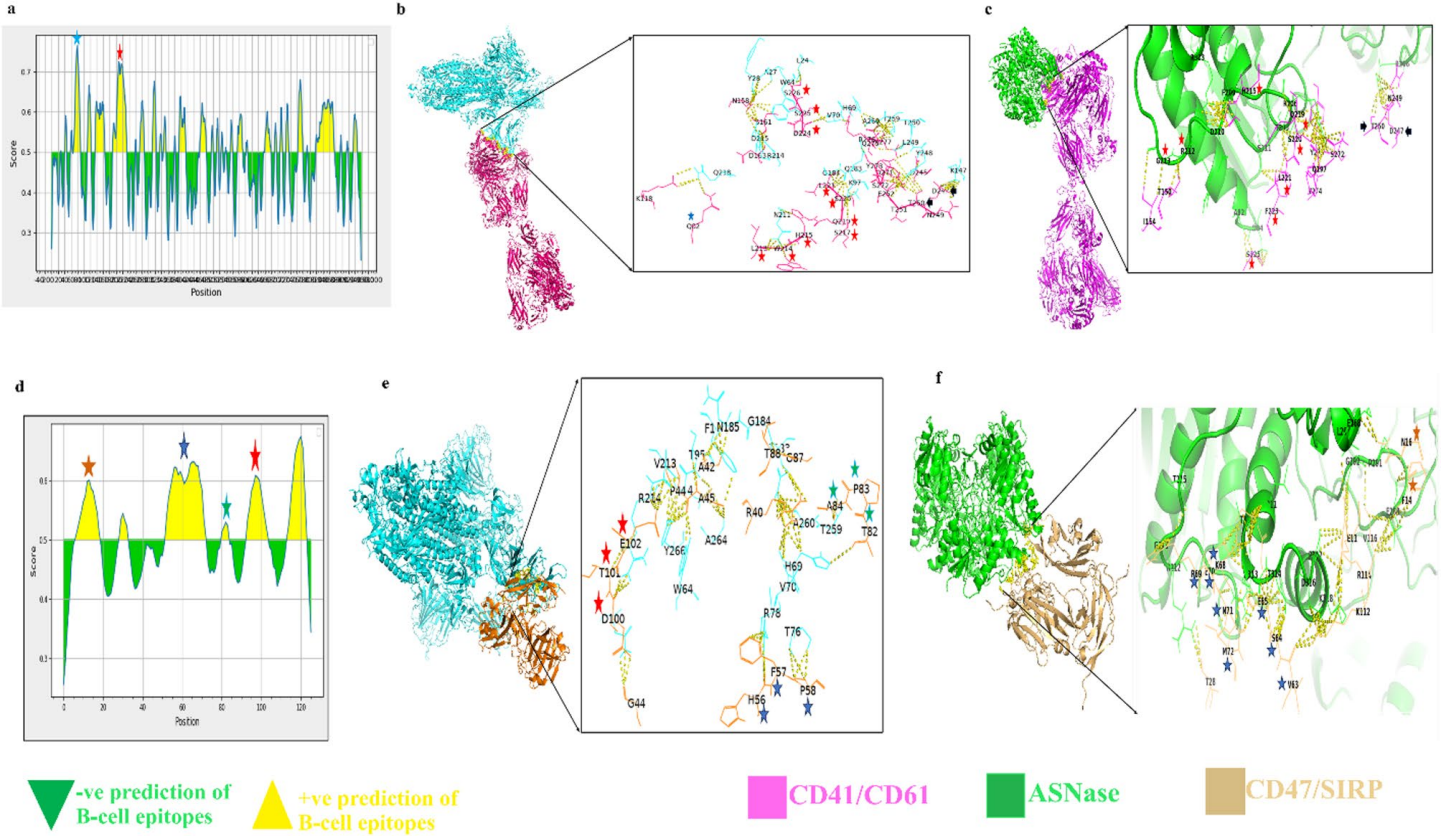
(**a**) Graph showing the analysis of B-cell epitopes of Human platelet glycoprotein αIIbβ3 (pdb:3fcs), (**b**) 3D image showing PPI between SARS-CoV-2 S (7bnn) and αIIbβ3 focusing on the critical residues that lie within the MIDAS and the predicted B-cell epitopes with the highest score including Q82 which lie within peptide extended from (D71 to R90), followed by the peptide extended (L213 W214 H215 S217 Q219 S220 L221 D224 S225 S226) which lie within that extended from Y190 to G233, (**c**) 3D image showing ASNase docking of αIIbβ3 via binding the critical residues that lie within the binding interface with ligand and the high scored predicted B-cell epitopes. (**d**) graph showing the peak of the predicted B-cell epitopes, (**e**) 3D image showing PPIs between SARS-CoV-2 S and human thrombospondin (CD47)/SIRP focusing on the residues with high predicted score to lie within the B-cell epitopes including H56 F57 P58 that have highly predicted scores 0.605, 0.622, 0.621, respectively, followed by T82 P83 A84 with average score 0.521 and D100 (0.6) T101 (0.577) E102 (0.544), (**f**) 3D image showing PPIs between ASNase and human thrombospondin receptor (CD47)/SIRP focusing on the residues that lie within the binding interface between CD74 and SIRP, B-cell epitopes and the key glycosylation site N16 of CD47.

SARS-CoV-2 S (pdb:7bnn) interacts with aIIBb3 (pdb:3fcs) via binding to **Asp247** and **T250** which lie within the metal-ion-dependent adhesion site (MIDAS) in addition to many residues which lie within B-cell epitopes including Q82 and the peptide extending between **Leu213** and **Ser226**, (Fig. 6a–c). Although the affinity of Spike protein overcame that of ASNase towards aIIBb3 where the ΔG(−27.0 kcal mol-1) and Kd (1.5e-20 M) for the former was lower than that for the latter ΔG(−19.9 kcal mol^−1^) and Kd (2.6e-15 M), (Supplementary Table W1). The superior finding was the efficacy of ASNase in docking integrin at the same residues that were targeted by SARS CoV-2 S with the exception of **Q82**. Thus, **ASNase** can compete the binding of SARS-CoV-2 S with aIIBb3. Regarding to small molecules, with the exception of ferulic and methionine sulfoximine, all the examined small molecule drugs can efficiently dock aIIBb3 via binding to **Cys** residues and/or B-cell epitopes, as shown in S7.

SARS-CoV-2 spike protein docked CD47/SIRP with higher affinity and very lower binding energy ΔG(−32.1 kcal mol-1) and Kd (2.7e-24 M) than that between ASNase and CD47/SIRP as ΔG(−29.8 kcal mol-1) and Kd (1.3e-22 M), (Supplementary Table W1). We also detected strong PPI between SARS-CoV-2 S and CD47/SIRP at many crucial residues, especially those lie within B-cell epitopes via binding to many residues especially, Arg69 and Met7**2** of SIRP, (Fig. 6, d-f). Amygdalin, phytic, quercetin, kaempferol, acacetin, calcitriol, quinapril, apigenin and quisqualic docked CD47/SIRP with high affinity (Supplementary table R2). As shown in S8, except zinc, ascorbate and methionine sulfoximine, 22 examined small molecule drugs targeted amino acid residues within the B-cell epitopes on SIRP that extended from **Tyr53** to **Thr73** which was the same epitope targeted by SARS-CoV-2 S. Regarding CD47, calcitriol, molsidomine and nitazoxanide bind to **Asn93**, calcitriol binds to **Glu106** while kaempferol binds to **Thr107**. Using B-cell epitope predictor showed that **Asn93**,** Glu106 and Thr107** lie within the B-cell epitopes on CD47.

### Statistical analysis of drug Docking studied proteins

**Fig. 7:**
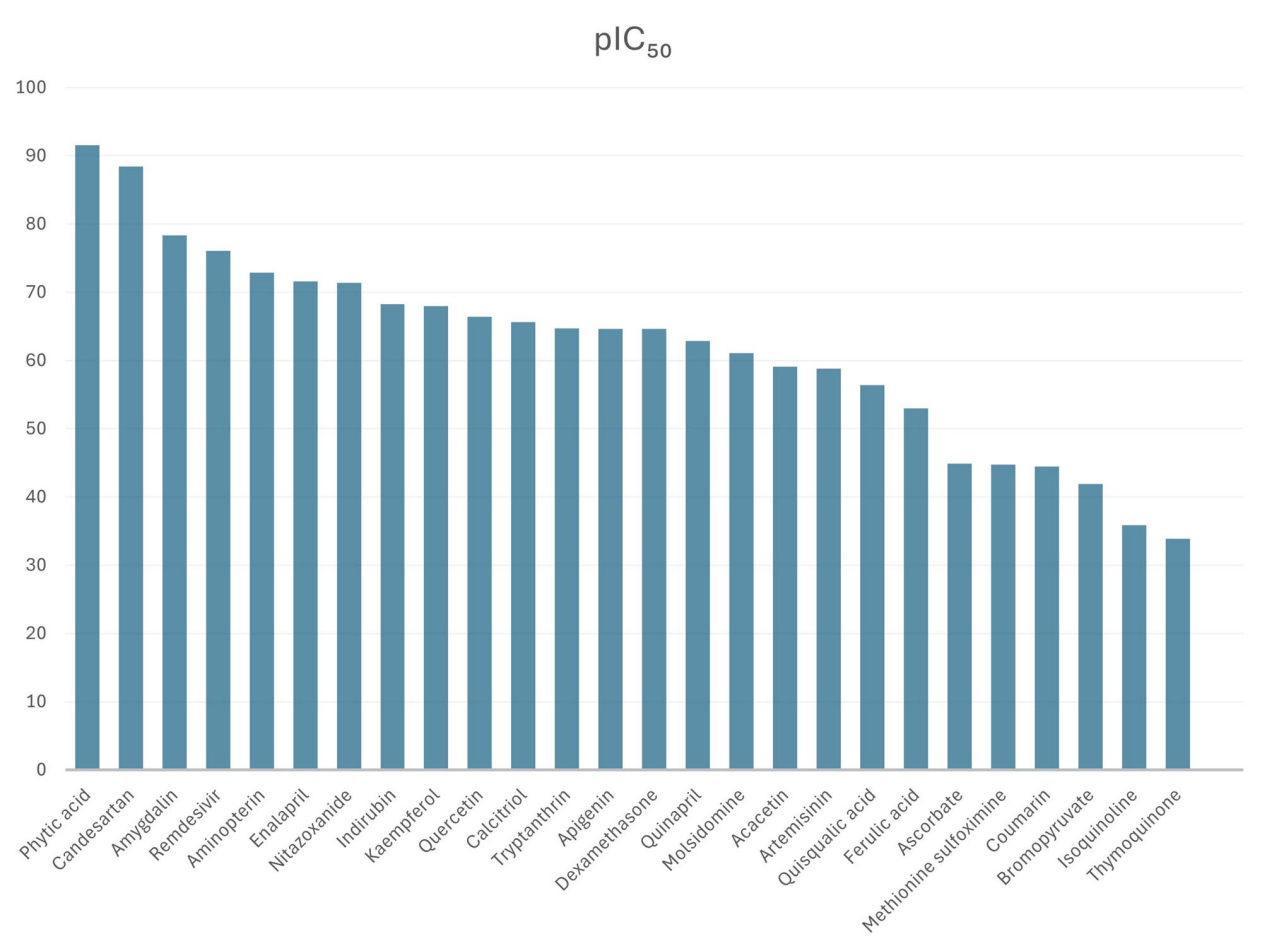
Predicted inhibitory potency (pIC_50_) of all tested drugs. Values were computed from docking-derived binding energies. Higher pIC_50_ values reflect high predicted potency and enhanced target inhibition.

Docking-derived binding free energy (ΔG) values varied substantially among the tested compounds, with natural agents exhibiting markedly more negative ΔG values than synthetic drugs, reflecting enhanced predicted binding stability and thermodynamic favourability (Supplementary table R2). Substantial heterogeneity was observed across targets (I² = 70%, τ² = 0.39), justifying the use of a random-effects model for meta-analysis. As shown in Fig. 7 and Supplementary table R3, comparison of the highest-affinity (lowest binding energy) of natural and synthetic ligands, phytic acid exhibited a significantly greater mean binding affinity than candesartan (ΔG = − 124.89 ± 13.57 kcal/mol; 95% CI: −3.0012 to − 0.8168; *p* = 0.0019) vs. (− 120.99 ± 7.4 kcal/mol) for candesartan. Both compounds ranked among the strongest predicted binders, corresponding to exceptionally low estimated IC_50_ values (2.8 × 10⁻⁸³ nM and 1.1 × 10⁻⁸⁰ nM, respectively) and pIC_50_ values higher than 88.

Comparative binding interface analysis across multiple targets—IL-17R, IL-6R, CD41/CD61, and CD47/SIRP—revealed that phytic acid preferentially targeted key residues, including Asn292 and Cys294 of IL-17R, Asp253, Gln255, His256, and His257 of IL-6R, Cys528, Cys674, and B-cell epitopes of CD41/CD61, and Gln552, Lys553, PCA1, and B-cell epitopes of CD47/SIRP. In contrast, candesartan primarily interacted with Cys185 and Cys294 (IL-17R); B-cell epitope regions (IL-6R); Cys674, Asn675, and B-cell epitopes (CD41/CD61); and additional B-cell epitope regions within CD47/SIRP. The second-ranking high-potency candidates, the natural compound amygdalin (ΔG = − 106.89 ± 14.11 kcal/mol; 95% CI: −10.1686 to 12.3486; *p* = 0.84; pIC_50_ = 78.36) and the synthetic agent remdesivir (ΔG = − 105.80 ± 7.4 kcal/mol; pIC_50_ = 76.09), demonstrated no statistically significant difference in predicted binding energy. Structural interaction mapping revealed that both ligands targeted key residues within the examined targets, most notably IL-17R (5n9b), where amygdalin predominantly interacted with Asn272, Cys276, Cys277, and Cys303, whereas remdesivir favoured contacts with Asn120, Cys185, Cys196, and Cys294. In contrast, the weakest predicted binder, bromopyruvate, exhibited a ΔG of − 57.15 ± 5.83 kcal/mol, corresponding to an IC_50_ of 1.3 × 10^−33^ nM (pIC_50_ = 41.90). Overall, natural products such as quercetin, apigenin, and indirubin demonstrated strong predicted binding affinities and high pIC_50_ values, comparable to or exceeding several clinically approved synthetic drugs (Supplementary table R4).

## Discussion

The present study underscores the ongoing persistence of COVID-19 and its profound impact on human life. To date, thousands of COVID-19 cases continue to be reported. While vaccination campaigns have significantly curtailed the spread of SARS-CoV-2 and reduced mortality rates, the post-COVID-19 era has extended its effects beyond public health, influencing education and the economy. Remote learning and work-from-home arrangements have become a defining lifestyle feature for Generation Z^36^. Although these shifts offer certain advantages, they may also foster social isolation and negatively influence productivity, particularly in manufacturing sectors such as agricultural supply chains, ultimately threatening food security^37^. The synergy between clinical phenotyping and in-silico modelling herein successfully deciphers the pathogenic mechanism linking SARS-CoV-2 spike protein interactions with haemoglobin and inflammatory dysregulation to clinical severity, directly translating these mechanistic insights into the rational discovery of promising dual-targeting anti-viral/anti-inflammatory therapeutics.

Although numerous studies have previously reported a strong association between dysregulated blood cell differentiation, cytokine storms, and the severity of COVID-19, no evidence has yet identified the shared factors between SARS-CoV-2 and the pro-inflammatory mediators asparagine (Asn) and cysteine (Cys), nor elucidated the extent to which existing therapies targeting these factors might alleviate disease severity. To date, many SARS-CoV-2 variants have affected different populations worldwide every year. Despite the high mutation rate of SARS-CoV-2, critical residues including Asn and Cys residues are highly conserved among them where the glycosylation of Asn serves as a shield that protects the virus from Ab and helps it to escape from immune cells^38,39^. The current data are in parallel line with the published sequences of SARS-CoV-2 S from earlier strain to current one, revealing that Asn and Cys are the highly conserved, key residues involved in the infectivity of SARS-CoV-2 variants and essential for Maintaining the spike protein architecture of earlier wave 2020 (7bnn), current waves 2024 (8×4h) and even in the oldest ones SARS-CoV S (5wrg, 6crv). Notably, both variants have the same mutation D614G which confer the virus more open conformation, in turn, increasing the viral infectivity causing higher viral loads in COVID-19 patients^40^. Our data highlight the persistence of SARS-CoV-2 infectivity tools where glycosylation of invariant N343 impairs the neutralization of virus by antibodies and helps the virus to evade from the immune cells. Additionally, Cys residues maintain the architecture structure of spike protein enabling the virus to invade the human cells^11^.

In contrast to the first COVID-19 wave where the mortality rate was very high among elderly critical cases of COVID-19, current wave of SARS-CoV-2 affected people of all ages including adults and children. Subsequently, this will have severe negative impact on the health and quality of life among the next generations and threat many SDGs. This finding supports that older age in the first wave was the main risk factor for a higher mortality rate than in the current wave where no deaths were recorded. The radiological findings including GGO, consolidations, broncho vascular interstitial thickening with air bronchogram lesions with a crazy paving, tree-in-bud indicated the damage of pulmonary tissues are still recorded among earlier and current wave. These spotlights on the urgent need to explore more efficient therapies to relieve the severe illness of COVID-19.

### Impact of SARS-CoV-2 on blood cells

In line with the findings of numerous studies, we detected many hematological abnormalities including leucocytosis, lymphopenia, neutrophilia, thrombocytopenia and monocytosis which are more frequent among elderly COVID-19 patients with severe illness. Our findings support that older age COVID-19 patients who suffer from severe illness already have immune disorders, increasing their vulnerability to severe complications such as pulmonary embolism, thrombosis, stroke and death. The current study identified increasing age and monocyte levels as significant independent predictors of disease severity, suggesting that innate immune cells may contribute to pathogenesis and could serve as early warning biomarkers. The robust AUC (0.858) confirms the model’s clinical utility for risk stratification. However, the moderate explanatory value (Nagelkerke’s *R*² = 0.45) highlights the influence of unmeasured factors such as comorbidities (e.g., diabetes), genetic predisposition, or epigenetic alterations. The specificity-sensitivity trade-off further emphasizes that the optimal probability threshold should be context-dependent: lower thresholds (high sensitivity) may be preferred in emergency settings to avoid missing severe cases, while higher thresholds (high specificity) could be used in ICU to confirm the critical cases. These findings align with established literature on immunosenescence while introducing novel insights into monocyte involvement, warranting future mechanistic studies to elucidate underlying pathways and validate these associations in larger, diverse cohorts.

Consistently, the role of platelet activation and contact with RBCs, WBCs and endothelial cells together with lymphopenia and neutrophilia have been implicated in the impairment of pulmonary microcirculation leading to thromboses, ischemic stroke, hemorrhagic stroke and multiorgan complications leading to death among the critical cases of COVID-19^9^. Additionally, malnourishment and multiple comorbidities in older patients are strongly associated with lymphopenia because of the presence of few lymphoid progenitors or a decrease in the proliferation together with a decrease in the survival capability, which predicts a high mortality rate among inpatients^41,42^. Additionally, leukocyte infiltration in the lung and other organs including the liver, heart, central nervous system and kidney is attributed to the severe complications in COVID-19 patients especially thrombus formation^9^. Moreover, we observed significant decreases in Haemoglobin, RBCs count and Hct in patients with severe illness vs. moderate cases and significantly increased the risk factor of severe complications significantly. Additionally, decreasing the mean RBCs count and Hct predicted the worst clinical outcomes in COVID-19 patients with high sensitivity and high specificity. This finding is in great agreement with many previously published reports in that these abnormalities were attributed to the expression of SARS-CoV-2 receptors on RBCs facilitating its infectivity and impairing RBCs differentiation, iron metabolism and redox homeostasis resulting in a lack of iron content and the degradation of RBCs^43^.

### Proposed mechanism of SARS-CoV-2 induced blood anomalies

The current data suggest a mechanism of SARS-CoV-2-induced blood cell anomalies. As reported, glycosylation of Asn residue of the SARS-CoV-2 spike protein provides a glycan coat that protects the virus from antibodies, conformational plasticity during fusion with the host cell and ACE2 recognition as well as a novel strategy for exploring novel vaccine. We found that SARS-CoV-2 S RBD used its Asn residues to adhere to critical residues of Hb including **H45 K61 G46 L96.** It was reported that these residues mediated the binding site with Heme^44^. Our data may explain the RBCs anomalies and hypoxia in COVID-19 as a consequence of the interaction between SARS-CoV-2 spike and hemoglobin causing disruption of the hemoglobin structure and function via binding to many crucial residues that consider with holding the iron atoms, maintenance the stability and integrity of hemoglobin leading to the observed fragmented RBCs, teardrop, schistocytes, target cells and elliptocytes. Thus, our data support evidence revealed that SARS-CoV-2 S interacts with Hb and provide a novel mechanism of this interaction.

### Therapeutic compounds directly targeting the SARS-CoV-2 Spike protein

Previous structural studies have characterized the SARS-CoV-2 Spike protein (S), with its receptor-binding domain (RBD) spanning residues 319–537 and containing two N-glycosylated asparagine (**N331**,** N343**) and two O-glycosylated sites (**T323**,** S325**)^45^. The current data revealed that N-glycans Asn are highly conserved among spike protein S of the selected 14 variants more than O-glycans. To date, no direct protein–protein interactions (PPIs) have been reported between Spike and the proteins examined in this study, except for recent evidence suggesting that **N331** and **N343** influence IL-6 gene expression rather than directly interacting with IL-6R^46,47^. The main finding was that ASNase, an antileukemia-approved therapy, exhibited high affinity for SARS-CoV-2 S. Surprisingly, we observed that ASNase molecules targeting SARS-CoV-2 S RBD at many critical amino acids, especially, **T345**,** S371** and **N487.** Sequence analysis of spike proteins from SARS-CoV-2 variants has revealed that N-glycosylated Asn residues are highly conserved, especially **N343**, **N234** and **N165** which support the RBD in the open conformation and act as a shield supporting the RBD. Of these, N343 is the invariant one of the twenty-two glycosylation sites conserved among all SARS-CoV-2 variants and considers as a “gate” that facilitate the conformational dynamic of RBD to “open” form, subsequently, facilitate its binding to the host cell receptor and invasion. Also, it was reported that mutation at **N343** significantly reduced the ability of binding of virus spike protein to host receptor^48^. N-glycans linked to Asn contribute to binding with hACE2, shielding the spike, protect the virus from neutralisation by human and monoclonal antibodies and regulating the conformational dynamics of the spike protein^49,50^. Moreover, S371 helps the virus to escape from the immune system by reducing the accessibility of epitopes to targeting antibodies^51^. In this context, our findings provide the first evidence that asparaginase (ASNase), an approved antileukemia therapy, exhibits high binding affinity for the Spike RBD, targeting multiple critical residues, particularly **N343**, **N487**,** T345 and S371**. These results suggest a previously unrecognized mechanism by which ASNase may disrupt Spike conformational dynamics and interfere with viral entry.

Thus, ASNase may confer a novel strategy to hinder both SARS-CoV-2 infectivity and its ability to evade from the immune cells. There are many sources of ASNase which were already approved for treatment of acute lymphoblastic leukaemia in children such as *Erwinia (USA) and E. Coli (Germany)*^52^. The former has shorter half-life and was administrated in case of patients who experienced allergic reaction to the latter one^53^. Also, Polyethylene-glycolated *E.Coli* derived ASNase has been approved because of its potent pharmacokinetic profile. However, many adverse effects of different formulations of ASNase such as Rylase and Oncaspar were reported including hypersensitivity, hyperglycaemia, pancreatitis, osteonecrosis, thromboembolism, neurotoxicity and Hepatic veno-occlusive disease^54^.

Apigenin and indirubin can destabilize the S architecture by targeting the key residues in S including **Cys391** and **Asn544**. The former forms one of the six disulfide bonds that stabilize the S architecture, hinder the accessibility of the vaccine to the SARS-CoV-2 S and impair the efficacy of the immune response, whereas the latter is one of the deamidation hotspot of S that enable it to escape from antibody recognition. Another superior finding, Phytic acid docked with the lowest binding energy in addition to target key Asn953, a key residue in S2 as reported by^55^. Additionally, we detected multiple binding sites between apigenin, indirubin, acacetin and calcitriol to key residues lying among the B-cell epitopes, whereas calcitriol binds to many amino residues of the RBM which lies within the binding interface with its receptor ACE2. In particular, calcitriol binds to **Asn234** of SARS-CoV-2 S. Many studies revealed that **N234** play a key role in stabilizing the up conformation of the RBD and facilitates the viral evasion of the Omicron variant from neutralization by antibodies^46^.

Our results indicate that apigenin and indirubin hold promise as safe immunomodulators in the management of critical COVID-19, owing to their capacity to directly disrupt the structural integrity of the SARS-CoV-2 spike (S) protein. In addition, apigenin—alongside acacetin, coumarin, thymoquinone, isoquinoline, quisqualic acid, and artemisinin—was found to recognize key B-cell epitopes of the S protein, highlighting their potential role in the formulation of plant-derived vaccines or vaccine adjuvants with enhanced safety profiles. The findings also reaffirm the significance of calcitriol, already integrated into certain COVID-19 treatment regimens, as a potential B-cell epitope–targeting compound. Moreover, we report novel mechanistic insights into quinapril, an antihypertensive medication, demonstrating its ability to destabilize the spike protein, thereby potentially improving host immune recognition and response.

Our findings provide further insight into the mechanisms by which ASNase, calcitriol, apigenin, and indirubin counteract the infectivity of a wide range of SARS-CoV-2 variants by targeting highly conserved residues. These mechanisms include disrupting the Spike architecture maintained by disulfide bonds between Cys residues, breaking the Spike Asn shield, and disrupting the Asn gate, which regulates the conformational dynamics of the RBD, thus locking it in the ‘down’ form. Altogether, these effects interfere with viral invasion of the host cell. These findings support the clinical relevance of incorporating ASNase and calcitriol into the treatment protocol for critical cases of COVID-19 to improve clinical outcomes.

### Implication of IL-17 and IL-6 in SARS-CoV-2 induced cytokine storm

The impact of the elevated levels of IL-6 and IL-17 on immune system were observed clearly. Many blood abnormalities were observed including lymphopenia, leucocytosis, neutrophilia, hypochromic microcytic anaemia, normochromic normocytic anaemia, thrombocytopenia and monocytosis significantly increase the risk factor of severe complications. In particular, thrombocytopenia and monocytosis were more prevalent in critical cases than in moderate cases. Although univariate analyses linked IL-6 and IL-17 to severity, these associations were nonsignificant in the adjusted model, likely due to confounding by age/monocytes or collinearity with other biomarkers. We propose that monocytes, which were independently associated with severity (aOR = 1.002, *p* = 0.013), may influence pathogenesis by: (1) amplifying Th17 responses via secretion of proinflammatory cytokines (e.g., IL-1β, IL-6), or (2) differentiating into tissue macrophages that modulate IL-17 activity. Additionally, a minor subset of CD16 + monocytes (< 5% of circulating monocytes) can directly produce IL-17 A under specific inflammatory conditions, such as rheumatoid arthritis. This aligns with prior work demonstrating that IL-6 overexpression - alongside TNF-α and IL-1β - correlates with severe-stage monocyte activation in COVID-19, though its independent predictive value was attenuated in comprehensive models.

The logistic regression analysis identified age and monocyte count as independent predictors of disease severity, reinforcing the well-established role of immunosenescence and innate immune cell dysregulation in the pathophysiology of severe disease. The strong association between advancing age and severity aligns with prior literature, suggesting that age-related changes in immune responsiveness and comorbid burden significantly modulate host vulnerability. Although the effect size of monocyte count was modest, its statistical significance underscores the potential role of monocyte-driven inflammation and antigen presentation in disease progression. Notably, other commonly implicated biomarkers—including pro-inflammatory cytokines (IL-6), acute-phase reactants (CRP), and hematologic ratios (NLR, PLR)—did not retain significance in the multivariable model, suggesting limited independent predictive value when adjusted for age and monocyte levels. The high discriminative capacity (AUC = 0.86) and explanatory power (R² = 0.45) of the model support its clinical utility in risk stratification, although further validation in larger, prospective cohorts is warranted to refine predictive algorithms and optimize biomarker integration into clinical practice. One explanation is that although monocytes are not classical IL-17 producers, their significant association with disease severity (aOR = 1.002, *p* = 0.013) may reflect either: (1) amplification of Th17 responses via proinflammatory cytokine secretion (IL-1β/IL-6), or (2) tissue-specific differentiation into macrophages that modulate IL-17 activity.

Additionally, many morphological abnormalities of lymphocytes such as reactive large granules with indented nuclei, lymphocyte, stimulated, atypical, plasmacytoid and smudge were more frequent in critical cases than in moderate cases. As reported, SARS-CoV-2-induced lymphocytes apoptosis, impairs the thymus and bone marrow and autoantibody destruction of infected lymphocytes^9^. Additionally, atypical and vacuolated monocytes were observed. Numerous neutrophil abnormalities were observed including left shift which appeared as an increase in immature neutrophil such as neutrophil staff cell, giant, binucleated and vacuolated neutrophils with thrombocytopenia and toxic granules. Moreover, the high frequency of pseudo-pleger-Huet neutrophil among severe cases indicated severe infection and myeloid stem cell disorders. Consistently, overwhelming evidence has been demonstrated that lymphopenia, neutrophilia, thrombocytopenia and monocytosis are the major risk factors for severe illness in COVID-19 patients, multiple organ disorders and mortality^9^. IL-6 inhibitors such as Actemra and Siltuximab were earlier approved immunotherapy for critical cases who showed elevated level of IL-6.

Our previous study showed that few cases did not have elevated level of IL-6, did not respond to IL-6 inhibitor “Actemra” and dead. This made us to hypothesize that other interleukins may be involved in the progression of severe complications of disease; thus, we aimed to measure the serum level of IL-17 in COVID-19 patients. The current work showed that ROC curve analysis of IL-6 and IL-17 showed the high specificity and moderate sensitivity of both tests in COVID-19 patients. Although a significant elevation of serum IL-6 and IL-17 levels were observed and predicted a poor clinical outcome in critical COVID-19 cases, there was neither correlation between their levels nor significant difference between their ROC-AUC. These valuable finding not only interpret the observed blood abnormalities but also emerge the role of IL-17 inhibitor in rescue the critical COVID-19 patients who have elevated level of IL-17.

Regression analysis revealed that while IL-6 and IL-17 levels were significantly associated with disease severity, their low R² values (0.0723 and 0.0617, respectively) indicate that each explains only a small fraction of severity variance. This is consistent with clinical observations that IL-6 alone is an inadequate prognostic marker and highlights the need for multi-marker models. The rejection of residual normality further suggests possible non-linear relationships or population heterogeneity. Collectively, these results support the potential of IL-17 as a complementary biomarker—acting synergistically or independently of IL-6—for early prediction of severe COVID-19 or post-COVID inflammatory syndromes. Further experimental and clinical validation is warranted to define its role within integrated prognostic frameworks.

Consistently, it has been demonstrated that IL-17 plays a crucial role in the induction of leukocytosis and neutrophilia in inflamed airways and is also implicated in atherosclerosis, a major lethal consequence in critical COVID-19 patients^59,60,^. In contrast to our finding, a previous study found that IL-6 and IL-17 are correlated and synergistically promote viral persistence by protecting infected cells from apoptosis and inducing tissue-infiltrating neutrophils to eliminate invading pathogens. These synergistic effects were carried out via genomic and non-genomic pathways where IL-6 activates the gene expression of IL-17 and vice versa. Inflammatory stimuli amplify the gene expression of both where IL-6 together with Il-17 enhances IL-6 expression via triggering the positive feedback loop. IL-6 promotes the expansion of Th17 cells, inhibits Tregs and triggers the activation phosphorylation of Signal Transducer and Activator of Transcription 3 (STAT3)/nuclear factor-kappa B (NF-κB) pathway. IL-17 together with IL-1β and TNFα induces the secretion of IL-6^64,65^. In this aspect, it was reported that IL-6 inhibitors can reduce the inflammatory process via blocking both IL-6 and IL-17 signal pathways. In contrast, Carbone et al., revealed that there is no correlation between IL-6 and IL-17 where IL-6 inhibitor, Actemra, significantly decreases IL-6 and slightly decreases IL-2 but did not influence the IL-17 level in rheumatoid arthritis. However, another study revealed that IL-6 and IL-17 independently inhibit T cell-mediated target cell destruction. It was recommended blocking of both IL-6 and IL-17 pathways to ameliorate chronic viral diseases and autoimmune diseases^68,69^. To explore the possible mechanism of SARS-CoV-2-induced blood disorders and inflammation by elevating IL-6 and IL-17, we carried out molecular docking to investigate PPIs between SARS-CoV-2 spike protein (S) and either unliganded IL-6R and IL-17R or complex forms IL-6/IL-6R and IL-17/IL-17R.

### Target therapies ameliorate PPIs SARS-CoV-2 induced cytokine storm

Bioinformatic analysis of PPIs between SARS-CoV-2 S, IL-17R (unliganded and complex form IL-17/IL-17R), IL-6R (unliganded and complex form IL-6/IL-6R), CD41/CD61 and CD47/SIRP was carried out. We subsequently examined the drug docking of twenty-seven drugs against the selected proteins.

On the basis of published data concerning the crystal structures of IL-6R^12^ and IL-17R^13^, Cys and Asn are crucial for maintaining their structure and function. There are four Cys and two Serine residues in IL-17R that are highly conserved and crucial for the formation of a cysteine knot fold in both to stabilize the interdomain linker region. Additionally, methionine (Met159, Met166 and Met218) and histidine (His212) plus Asn-linked glycosylation and Cys knots are essential for stabilizing IL-17RA^70^. The presence of T581 and N331 which mediate sialylated O-glycosylation and N-glycosylation of SARS-CoV-2 S within the binding site with unliganded IL-17R highlights a novel tool by which SARS-CoV-2 can induce inflammation^71^. Both residues are essential for CoV pathogenesis, interspecies transmission, infection, intercellular expansion and cell-cell spread. IL-6 and IL-17 either independently or synergistically promote viral persistence by protecting virus-infected cells from apoptosis and/or by inhibiting T cell-mediated target cell destruction^72^.

Another superior result was that SARS-CoV-2 S targeted N36 of both IL-17R and IL-6R which is one of the N-glycosylation sites for both. In addition, S targeted many other B-cell epitopes on both, in addition to those within the binding interface with their ligand. These PPIs may be a novel mechanism for modulating the serum levels of IL-6 and IL-17 and May cause the observed insignificant correlation by affecting their non-genomic signal pathways rather than the genomic pathway that was found by Korn et al., 2021^73^. Notably, the two most potent synthetic agents, calcitriol and remdesivir, were predicted to interact with His212, a critical residue mediating IL-17R subunit dimerization. This interaction may represent a previously unrecognized mechanism contributing to the therapeutic efficacy of these widely approved COVID-19 interventions, potentially through the modulation of IL-17–driven inflammatory signalling. In consistent, many recent studies revealed the beneficial of addition of remdesivir and vitamin D to the treatment protocol of immunocompromised COVID-19 patients^74,75^.

Also, these findings support the efficacy of an IL-6 inhibitor (Actemra) and suggest the urgent need to block IL-17/IL-17R signaling to relieve the cytokine storm in critical cases of COVID-19. Our data showed that ASNase can efficiently block IL-17R by binding to critical residues especially, C213 and N240 plus many B-cell epitopes. Thus, ASNase can inhibit the inflammatory action of IL-17R via many mechanisms; first, it binds to one arm of the disulfide bond which forms cysteine knots that stabilize the interdomain linker, second, it inhibits N-glycosylation on Asn; third, it blocks the extension of the peptide from M217 to E227 which lies within the highest predicted B-cell epitopes of IL-17R. Additionally, ASNase showed higher affinity to IL-17R (5n9b) and IL-17/IL-17R (5nan) compared to that of Secukinumab. Along with the anti-tumorigenic effect of ASNase, its immunosuppressive and anti-inflammatory effects by overcoming T-cell-mediated B-cell responses were well documented^76^.

As shown in Supplementary table R2, the phytotherapies had high affinities for all the studied forms of IL-17R, IL-6R and their complex structures. Additionally, most of the selected small molecules both phytotherapies such as apigenin, amygdalin, ferulic and kaempferol, phytic and calcitriol can block unliganded IL-17R (5n9b) by targeting many critical residues, especially, cysteine which maintains its structure through the formation of disulfide bonds. Additionally, phytotherapies such as amygdalin, ferulic and phytic and synthetic ones such as calcitriol and molsidomine bind to Asn, which plays a crucial role in the glycosylation of IL-17R. Additionally, ferulic binds to Thr121 which lie within an important peptide (NTNER) that lie at the binding interface with the ligand. Thus, these drugs can counteract the cytokine storm by blocking the binding of IL-17R to its ligand.

Collectively, these findings support the continued exploration and optimization of natural compounds as viable leads in multi-target therapeutic strategies, particularly in diseases driven by dysregulated cytokine signalling.

Many studies have revealed the role of the four intramolecular disulfide bonds between Cys residues and two free Cys residues in the homodimerization of IL-6R, Maintaining its architecture and signal transduction. In addition, the junction between domain 2 (D2) which consists of 4 loops (L1-L4) and D3 which consists of 3 loops (L5-L7), plays role in the folding of IL-6R and binding to its ligand^77,78^. Our data showed that **ASNase** can dock unliganded **IL-6R** via binding to many critical residues at the outer elbow forming the junction between D2 and D3 in addition to bind to Arg274 and Trp284 which together form the tryptophanearginine ladder “WSXWS”, a unique sequence motif that stabilizes its architecture. In addition, ASNase targeted many residues that lie within the highest predicted B-cell epitopes on IL-6R. With respect to IL-6/IL-6R (1p9m), kaempferol, quercetin, and tryptanthrin can block the IL-6/IL-6R signal pathway via binding to one of the five crucial disulfide bonds between **Cys150** and **Cys160**. Acacetin and remdesivir binds to **Asn135** while aminopterin and apigenin bind to **Asn224**,** Trp249**,** Arg259**, candesartan, indirubin, nitazoxanide and enalapril bind to **Asn224** of IL-6R. Dexamethasone binds to Asn60, Asn61 of IL-6 and to Asn136 of IL-6R. Of these, it has been demonstrated that **Asn135** is one of the nine glycosylated sites while non-glycosylated **Asn224** is crucial for the protein yield, while Trp249 and Arg259 lie within the binding interface between IL-6R beta (gp130) and IL-6. Thymoquinone and artemisinin binds to **Arg168** and **Cys50**, respectively, of IL-6. Overall, small-molecule drugs can block IL-6/IL-6R and IL-17/IL-17R signal pathways via targeting mainly **Cys** residues and to lesser extent **Asn** residues as well as the binding interface.

### Target therapies ameliorate SARS-CoV-2 induced coagulopathy

Integrins and integrin-associated proteins play pivotal roles in cell migration, an essential process of immune response. As reported, the integrins GPIIb/IIIa carry most of human platelet antigens (20 out of 33) that mediate the hemostasis and inflammation^79^. Together, GPIIb/IIIa, IL-6R, IL-17R and CD47/SIRP are together participate in regulation of the immune response^80^. In our previous work, we reported that elevated level of GPIIb/IIIa was associated with poor clinical outcomes in COVID-19 patients^81^. Considering the published structure of αIIβ3, **Asp247** and **Thr250** lie within the metal-ion-dependent adhesion site (MIDAS)^82^. The current data may help in explain SARS-CoV-2-induced coagulopathy via binding to **Asp247** and **Thr250** in addition to many residues that are predicted to lie within the B-cell epitopes of GPIIb/IIIa causing platelet clumping and RBCs fragments as shown in the blood films. It has been demonstrated that GGO and consolidation are the main manifestations of coagulopathy and pulmonary hemorrhage among critical cases of COVID-19 patients^83^.

The superior finding of the current study was that for ASNase which efficiently binds to αIIβ3 at the same amino acids that are targeted by SARS CoV-2 S, which include that lies within the binding site with MIDAS and that was predicted to lie within B-cell epitopes with the exception of Q82. Thus, ASNase can compete with SARS-CoV-2 S for binding to αIIβ3, subsequently, counteracting pulmonary epithelium damage and coagulopathy. Additionally, except for ferulic acid and methionine sulfoximine, all the examined small molecules drugs can efficiently docked aIIBb3 via binding to **Cys** residues in addition to many residues that lie within the highly predicted B-cell epitopes. Thus, they may ameliorate the severe illness such as coagulopathy, pulmonary embolism and thrombosis in patients with COVID-19 by blocking the B-cell adhesion to antigenic epitopes of αIIβ3 integrin. Our findings are in line with those of a previous study that indicated the efficacy of an αIIβ3 inhibitor in preventing the aggregation of platelets and thrombosis^84,85^.

Our data revealed that all the selected small molecule drugs docked IL-6R except ascorbate, methionine sulfoximine and bromopyruvate. Notably, M250–H256 peptide which forms L6 is the most frequent target of the selected small-molecules phytotherapies, including kaempferol, phytic, quercetin, tryptanthrin as well as the synthetic one quinapril, which is an antihypertensive drug. In addition, kaempferol and quercetin can block IL-6R signal via preventing the receptors homodimerization via targeting the free Cys285. Ferulic acid destabilizes the structure of IL-6R (1n26) via targeting Cys77 which mediates in the formation of intramolecular disulfide bonds. Quinapril also targets His261, which cantered the signalling transduction. Additionally, tryptanthrin binds to many residues especially Ser167 and Asn136. As reported, the former is essential for signalling pathway whereas the latter is an essential residue at the binding interface with the ligand. Moreover, with the exception of ferulic acid, the selected drugs docked to IL-6R at many B-cell epitopes.

The broad expression of IL-6, IL-17 and CD47/SIRP on erythrocytes, platelets, lymphohematopoietic cell, and endothelial and epithelial cells indicates their roles in cell differentiation, apoptosis, phagocytosis and cell-cell fusion, as well as many metabolic and vascular disorders. Thus, we performed an in-silico study to investigate the role of CD47/SIRP in the severity of COVID-19 and as a potential target in critical cases. A previous study demonstrated that Tocilizumab antagonizes the antiphagocytic effect of CD47 via the inhibition of IL-6. Additionally, it has been demonstrated that in response to acute inflammation, the interaction between TSP-1 and CD47/SIRP mediates the regulation of IL-17-induced cell adhesion. Additionally, a previous study demonstrated that elevated level of CD47 in COVID-19 patients was associated with immune evasion and impairing the immune recognition of virus-infected cells leading to the severe complications of COVID-19 including hypertension, vascular disease, lung fibrosis, myocardial injury, kidney injury and stroke^86^. Moreover, CD47/SIRP regulates B-cell migration which is essential for its differentiation and the production of specific antigen^87^. Blockade of the CD47/SIRPa interaction is a novel strategy for the treatment of atherosclerosis, cancer and autoimmune diseases^88^. On the basis of the published structure of CD47/SIRP, the folding of SIRP and its interaction with CD47 depends on the salt bridges between Asp100 of SIRP and both Lys96 of SIRP and Lys39 of CD47^15^.

Thus, we may suggest that, first, the PPI between SARS-CoV-2 and CD-47/SIRP may be a mechanism of RBCs deformability such as elliptocytes, teardrop and target cells; second, the binding of SARS-CoV-2 S to B-cell epitopes on CD47/SIRP may enhance the phagocytosis of RBCs. Together, these two mechanisms may explain the significant decrease in RBCs and the appearance of RBCs anomalies. On the other hand, ASNase efficiently docked to CD47/SIRP via binding to many residues especially those that lie within the binding interface including R69 and M72 of SIRP as Hatherley et al., reported^15^. In addition, ASNase binds to multiple residues that lie within the predicted B-cell epitopes on SIRP as well as Asn16 which is one of the five most potent glycosylation sites of CD47. Also, small molecules drugs including amygdalin, aminopterin, calcitriol and molsidomine, nitazoxanide and remdesivir bind to another one of these glycosylation sites (Asn93) of CD47. The small molecule drugs amygdalin, apigenin, kaempferol, phytic, quinapril, quisqualic, and thymoquinone bind to the pyroglutamate acid (PCA1) of CD47, thus exerting synergistic effects with ASNase via interference with the binding of CD47 with SIRP. PCA1 lies at the N-terminus of CD47, which intermediates its binding with SIRPα^89^. Thus, combination of ASNase with small molecules may enhance the phagocytosis of infected cells. This finding is in line with Kelleni’s protocol who recommended the involvement of nitazoxanide to improve clinical outcomes and reduce the mortality rate of critical cases of COVID-19^90^. Thus, we may suggest that a combination of ASNase and the selected small-molecule drugs that target critical residues in CD47/SIRP prevents stroke in critical cases of COVID-19 via interference with their binding. Consistently, many studies have indicated the efficacy of CD47 inhibitors as promising therapeutic strategies against immune disorders and stroke^91^.

These findings support the emphasize the clinical relevance of integrating the most potent synthetic anti-angina drugs, quinapril and molsidomine, in the treatment protocol of critical cases of COVID-19 patients to improve the clinical outcomes and reduce the mortality rate. Although both drugs were approved since 1980 s, rare evidence proposed their clinical relevance in ameliorating the severe illness in critical cases or reduce the mortality rate which were mainly related to platelets and endothelial dysfunction resulting in atherosclerosis, pulmonary embolism and stroke. Recent study elucidated its anti-angina mechanism to ameliorate atherosclerosis and stroke via exerting antioxidant and anti-inflammatory effects by counteracting the inflammation pathway and necrosis via increasing B cell/lymphoma 2 (BCL-2), lowered caspase-3 and IL-6. Also, recent experimental study emerges a novel inhalation formulation to improve the efficacy of molsidomine in reducing the acute pulmonary hypertension^92,93^. The current finding of insignificant difference in the binding affinity of natural and synthetic compounds in binding with integrins and associated proteins together with excellent safety and superior inhibitory potency of natural compounds, may highlight the urgent need to further study to explore novel formula of natural compounds to improve their bioavailability. This may alleviate the endothelial and platelets dysfunction in COVID-19 patients.

In summary, most of the small-molecule drugs in our study targeted mainly Cys, whereas few targeted Asn or other critical residues such as Met, Pro and Arg. Notably, phytic acid emerged as a highly promising multi-target inhibitor, demonstrating superior predicted binding affinity, potency, and safety profiles against all target proteins implicated in SARS-CoV-2-induced severe inflammation and hematological dysregulation. While bioavailability presents a potential challenge for phytic acid, the established high bioavailability of Calcium Phytate formulations offers a viable delivery strategy^94^. Furthermore, our findings corroborate the clinical role of vitamin D supplementation in improving COVID-19 outcomes. In contrast, Zinc failed to dock any of the proteins examined, suggesting that its potential effects may arise from mechanisms independent of direct protein interaction, warranting further investigation.

We used the crystal structure of ASNase from Erwinia chrysanthemi (PDB: 1o7j), which was approved 50 years ago for the treatment of children with lymphoblastic leukemia^95^. Further studies are planned to examine the efficacy, pharmacodynamics, and safety of herbal sources of ASNase, such as Solanum nigrum. In particular, therapies targeting asparagine (Asn) directly may be more efficient than those targeting asparagine synthetase, especially given the low cell permeability of Bisabosqual A, a therapy that targets Asn synthetase, hinders its anticancer activity^96^. Taken together, numerous studies have revealed that the bioactive components of Solanum nigrum (e.g. quercetin, apigenin, kaempferol, coumarin and ferulic acid)^97,98^ as well as amygdalin^99^ thymoquinone^100^, tryptanthrin^101^ ameliorate inflammation in COVID-19 patients, however, the ability of these compounds to target B-cell epitopes of both SARS-CoV-2 S and proinflammatory mediators as a possible mechanism to counteract the cytokine storm has not been revealed. Additionally, extensive evidence has confirmed their safety. Many clinical studies revealed that thymoquinone exerts anti-metabolic syndrome, hypoglycemic, antioxidant, immunomodulatory, neuroprotective, hypolipidemic, and cardioprotective effects, with no observed side effects at various doses^102^. It has been demonstrated that the immunomodulatory effect of thymoquinone is attributed to its ability to significantly decrease IL-6 and TNF-α levels. In this context, we propose a novel mechanism for its immunomodulatory activity by targeting B-cell epitopes on unliganded IL-6R and IL-17R, and impairing ligand binding by blocking the receptor–ligand interface.

We recommend the use of medicinal plants for preventing and treating immune disorders related to SARS-CoV-2 infection or autoimmune diseases. Our results support previous studies that have demonstrated the efficacy of natural medicines compared to synthetic ones in reducing the severity of COVID-19 and other inflammatory diseases^103^. Phytotherapies possess unique characteristics and superior features. First, they do not exhibit significant adverse effects like synthetic drugs^104^. Second, in line with numerous studies, we suggest that the complex and intersected signaling pathways which regulate the vital processes in the human body require combination therapy, rather than monotherapy, to effectively address the multiple dysregulations associated with disease. Consistent with this, extensive research has shown that combination therapies are more effective than monotherapies in treating conditions such as cancer, metabolic disorders, and autoimmune diseases^105^. Plants as a reservoir of numerous natural flavonoids, carotenoids, saponins and polyphenolic can exert the beneficial effects safely compared to synthetic drugs which of course can exert the same action effectively but with adverse effects^106^. To date, most synthetic therapies have adverse effects even Aspirin, the oldest anti-platelet, analgesic, antipyretic and anti-inflammatory therapy may cause gastrointestinal injury with either plain or enteric-coated formulations^107^. On the other hand, our data demonstrated the efficacy of apigenin and its methylated derivative, acacetin, as effective anti-inflammatory agents. They function by blocking the binding sites and B-cell epitopes of unliganded IL-6R and IL-17R, as well as the interfaces between these receptors and their ligands in the active IL-6/IL-6R and IL-17/IL-17R complexes. Thus, they may add benefits in ameliorates the inflammatory diseases. Additionally, they are effectively targeting B-cell epitopes on integrin (3fcs) and CD47/SIRP (2jjs), in turn, they may safely counteract the platelet activation in coagulopathy diseases either in COVID-19, post-COVID-19 syndrome and vascular diseases. Particularly, an increasing number of studies are revealing the long-term negative impacts of SARS-CoV-2 on the health of future generations. This supports the hypothesis proposed by Nature editors that the COVID-19 Pandemic has Made achieving the SDGs by 2030 out of reach, especially, those related to children and adults, who are central to this agenda^108,109^.

From previous, it is clear that SARS-CoV-2 variants and post-COVID-19 syndrome continue to pose significant threats to global health. Therefore, scientific research on therapies and vaccines should progress forwards towards exploring more target precision therapy that can inhibit not only viral infectivity but also its inflammatory complications. We have to be ready for future outbreaks and address the lasting damage caused by earlier waves. In this context, our goal was to target both challenges with a single, unified strategy. In this context, our goal was to target two threats with a single strategy. To achieve this objective, we followed two parallel approaches: one involved analyzing the biochemical and haematological data and their association with the clinical and radiological findings; the other involved an in-silico study including molecular docking to investigate the PPIs between PPIs between SARS-CoV-2 and both human proteins and pro-inflammatory mediators as well as perform drug docking of small molecules to both the SARS-CoV-2 spike protein and pro-inflammatory mediators. The former provides real insight into the impact of SARS-CoV-2 on human health, while the latter is a simulation of the infectivity mechanism at the molecular level, which may explain the progression and severity of the disease, as well as the long-term persistence of post-COVID-19 syndrome. We hypothesized that incorporation of an in-silico study provided deep insight into the mechanisms underlying SARS-CoV-2-induced blood anomalies, immune disorders, multiple organ disorders and post-COVID-19 syndrome.

Referring to drug docking, we analysed the bioinformatic data from two perspectives. First, we examined whether the selected therapies targeted proteins at key amino acid residues involved in the binding interface between ligand and receptor or affected the protein structure. Second, we assessed whether the PPIs influenced the B-cell epitopes on the selected proteins. Both analyses offer potential foundations for further clinical studies to evaluate their pharmacokinetic, pharmacodynamic, and clinical relevance against wide range of viruses’ species beyond SARS-CoV-2. Especially, many recent studies have revealed that N-glycosylation on Asn residues are essential for many viruses such as HCV^110^, HIV^111^, Epstein-Barr virus^112^ to invade the host cell and become a potential target to immunotherapy and vaccines. Our findings support the hypothesis that there is an urgent need to incorporate safe medicinal plants into the formulation of immunotherapy. These therapies may effectively target both the spike proteins of SARS-CoV-2 variants and human inflammatory mediators, thereby minimizing the adverse effects associated with synthetic drugs.

### Application of drug docking in ameliorating the severity of COVID-19

The predicted IC50 values, derived from scaled iGEMDock binding energies, align with established structure-activity relationships. While statistical significance was not reached (*P* > 0.05) in comparisons between natural and synthetic compounds across all target proteins, the consistent trend of stronger predicted binding affinities for natural compounds—evidenced by uniformly negative ΔΔG values—suggests a potential generalizable advantage. Natural compounds exhibited more favorable ΔG values and lower predicted IC50 than synthetic analogs. Notably, phytic acid and candesartan emerged as top binders, with the lowest predicted IC50 values, implicating their high-affinity interactions with inflammatory and coagulopathy pathway targets. This is structurally rationalized by their polyanionic (phytic acid) and polycyclic (candesartan) motifs, which enhance target engagement. In contrast, dexamethasone, a synthetic drug, showed moderate affinity, indicating a need for optimization, while natural flavonoids like quercetin may require scaffold derivatization to improve potency. Discrepancies between predicted and experimental IC50 values, likely due to unmodeled solvation effects, entropic penalties, or target flexibility, underscore the necessity of experimental validation. Nevertheless, these computational results provide a strategic framework for prioritizing candidates in further mechanistic and preclinical studies.

Notably, kaempferol, apigenin, tryptanthrin, quercetin and indirubin also demonstrated strong predicted affinities and high pIC_50_ values, suggesting substantial inhibitory potential, possibly reflecting their polyphenolic scaffolds and multiple hydrogen bond donors/acceptors that can adapt to diverse binding pockets. Ferulic acid, ascorbate, isoquinoline, and thymoquinone fell into a moderate potency range, with binding energies suggesting weaker but still potentially relevant interactions; these ligands may benefit from structural optimization or derivatization to enhance binding thermodynamics. Quisqualic acid, while smaller and more polar, displayed better-than-expected binding, possibly due to favorable electrostatic complementarity with the target’s active site.

The broad range of binding affinities observed among the diverse natural products underscores the role of scaffold complexity, functional group diversity, and conformational adaptability in optimizing receptor engagement. Phytic acid and amygdalin the top-ranked natural compounds, exhibited high affinity towards receptors, particularly at functionally relevant sites within IL-17R, IL-6R, CD41/CD61, and CD47/SIRP, suggesting a strong potential for multi-target modulation. Collectively, these trends underscore the potential of natural scaffolds as promising lead structures for poly-pharmacological strategies, provided that their pharmacokinetic limitations are addressed through rational design and formulation.

Similarly, synthetic agents such as candesartan, remdesivir, dexamethasone, and enalapril ranked among the top predicted binders, with notable interactions at key residues, reinforcing their potential as lead candidates in rational drug design. Consistent with these computational findings, recent clinical evidence has demonstrated that enalapril and candesartan are associated with reduced COVID-19 severity and mortality, particularly in patients with multiple comorbidities, including diabetes mellitus, hypertension, and cardiovascular disease^91^.

Finally, our findings highlight the relevance of uniquely integrating a clinical cross-sectional investigation with advanced in-silico modelling, providing a dual-perspective approach that links real-world patient data with molecular-level mechanistic insights. By combining clinical biomarker profiling, radiological assessment, and statistical modelling with computational docking of key inflammatory mediators and SARS-CoV-2 targets, we were able to bridge the gap between observed disease patterns and their underlying molecular drivers. This integrative strategy not only enabled the identification of IL-17, alongside IL-6, as an independent predictor of COVID-19 severity but also uncovered structural motifs—particularly Asparagine (Asn) and cysteine (Cys) residues—shared between viral proteins and host inflammatory mediators. Such findings reveal previously uncharacterized mechanisms of immune dysregulation, cytokine amplification, and host–virus protein–protein interactions. Importantly, the dual approach facilitated the proposal of novel therapeutic avenues, including the use of Asparaginase (ASNase) and selected phytochemicals to simultaneously target viral entry and hyperinflammatory pathways. These mechanistic and therapeutic insights, emerging from the synergy between clinical and computational domains, represent a significant advance in our understanding of COVID-19 pathophysiology and highlight the broader potential of combining cross-sectional clinical data with in-silico screening for accelerating the discovery of precision therapies in emerging infectious and inflammatory diseases. These findings warrant experimental validation, including biophysical binding assays and in vitro functional studies, to confirm the computational predictions and to assess pharmacokinetic and safety profiles.

The selected small natural and synthetic ligands as well as the polypeptide drugs may serve as promising scaffolds for developing effective therapeutics and vaccines with minimal adverse effects. The present study underscores the pivotal role of specific amino acid residues—particularly Asn and Cys—positioned at the ligand–receptor interface, frequently residing within B-cell epitopes and contributing to structural integrity. These residues represent common molecular determinants shared by SARS-CoV-2 variants and human pro-inflammatory mediators. Our findings provide mechanistic insight into SARS-CoV-2 infection and host specificity, identifying key protein–protein interactions (PPIs) interface binding sites as potential therapeutic targets. This work opens avenues for further experimental and clinical investigations into plant-derived compounds as modulators of immune defense, with potential to disrupt viral entry via spike protein neutralization, interfere with pathogenic PPIs involving blood proteins and cytokines, and inhibit inflammatory ligand–receptor binding. Such strategies may not only mitigate acute COVID-19 severity but also reduce the burden of post-COVID-19 syndromes, including autoimmune, vascular, and haematological complications, thereby safeguarding public health in future pandemics.

## Conclusion

Our findings identify elevated IL-17 and IL-6 levels as key inflammatory biomarkers directly predictive of COVID-19 severity and clinical outcomes. This predictive value persists independent of confounding factors like age and monocyte count, which robustly stratify severity risk. Bridging these clinical observations with in-silico analysis elucidates the mechanistic link between SARS-CoV-2 spike protein interactions, haemoglobin dysfunction, and inflammatory dysregulation driven by mediators like IL-17 and IL-6. This unified understanding facilitates the discovery of novel dual-targeting therapeutic candidates. Significantly, natural compounds demonstrated superior predicted binding affinity, potency, and safety profiles compared to synthetic agents. Consequently, a therapeutic combination incorporating ASNase, calcitriol, and promising natural compounds (e.g., acacetin, apigenin, amygdalin, coumarin, phytic acid, kaempferol, ferulic acid, indirubin, quercetin, tryptanthrin, thymoquinone) holds significant potential for mitigating cytokine storms in critical COVID-19, post-COVID syndrome, and autoimmune conditions like rheumatoid arthritis and psoriasis.

### Translational implications of our findings for clinical medicine

Our study highlights translational opportunities to optimize therapeutic strategies for severe COVID-19 and post-COVID-19 syndromes, particularly in addressing the escalating burden of associated autoimmune pathologies. We advocate for targeted pharmacology research to develop dual-action therapeutics or treatment protocol—combining natural and synthetic agents—that selectively target Asparagine (Asn) and Cysteine (Cys) residues. Such an approach could enhance therapeutic precision, mitigate the risks of polypharmacy, excessive immunosuppression, and iatrogenic complications linked to conventional broad-spectrum anti-inflammatory regimens.

### Limitation of our study

Our study acknowledges several limitations. Primarily, the relatively modest sample size—attributable to Egypt’s unique 2020 epidemiological context characterized by a dramatically reduced incidence of severe COVID-19 requiring hospitalization. By mid-2020, ICU admissions had become exceedingly rare, with most cases managed outpatient; notably, only two paediatric ICU admissions were included. Secondly, lack of data on important comorbidities (e.g., diabetes mellitus, dyslipidemia, autoimmune diseases), which may have influenced the observed associations. These limitations highlight the need for future studies to prospectively integrate multi-omics data with clinical comorbidities when designing targeted therapies. Thirdly, the observational cross-sectional design constrains causal inference and may introduce residual confounding despite statistical adjustments. Finally, while in-silico analyses identified promising therapeutic targets (specifically Asn, Cys, critical residues, and B-cell epitopes) and candidate agents (ASNase, calcitriol, natural compounds), these predictive findings necessitate experimental validation of binding mechanisms and clinical assessment of therapeutic efficacy.

## Further study

Future studies will validate the logistic regression model in larger, independent cohorts to confirm the clinical relevance of the identified biomarkers. Prospective investigations should also examine interactions with comorbidities—particularly glycemic metrics—given molecular docking evidence that glycosylation modulates SARS-CoV-2 cell entry.

In parallel, experimental studies will be designed to evaluate pharmacokinetic parameters (e.g., bioavailability and half-life) and toxicity profiles of the studied drugs. This integrated computational-experimental approach will establish a critical bridge between in-silico predictions and translational relevance, along with investigation of key ADMET (Absorption, Distribution, Metabolism, Excretion, and Toxicity) properties essential for drug development.

## Supplementary Information

Below is the link to the electronic supplementary material.

Supplementary Material 1

Supplementary Material 2

Supplementary Material 2

Supplementary Material 4

Supplementary Material 5

Supplementary Material 6

Supplementary Material 7

Supplementary Material 8

Supplementary Material 9

Supplementary Material 10

Supplementary Material 11

Supplementary Material 12

Supplementary Material 13

Supplementary Material 14

Supplementary Material 15

Supplementary Material 16

Supplementary Material 17

## Data Availability

Data is provided within the manuscript and supplementary information files.

## Abbreviations

IL-17: Interleukin-17
IL-17R: Interleukin-17 receptor
IL-6: Interleukin-6
IL-6R: Interleukin-6 receptor
MLR: Monocytes to lymphocytes ratio
NLR: Neutrophil to lymphocytes ratio
PLR: Platelets to lymphocytes ration
RT-PCR: Real time polymerase chain reaction
CT: Computed tomography
SPO2: Oxygen saturation
ICU: Intensive care unit
CLIA: Chemiluminescence immunoassay
CRP: C-reactive protein
ALT: Alanine transaminase
AST: Aspartate transaminase
K2- EDTA: Potassium salt of ethylene-diamine-tetra acetic acid
CBCs: Complete blood counts
AUC: Area under curve-receiver
CI: Confidence intervals
OR: Odd ratio
PPV: Positive predictive value
NPV: Negative predictive value
PPIs: Protein-protein interaction
Asn/N: Asparagine
ASNase: Asparaginase
Cys/C: Cysteine
Pro/P: Proline
Leu/L: Leucine
His/H: Histidine
Met/M: Methionine
Gln/Q: Glutamine
Asp/D: Aspartic
Tyr/Y: Tyrosine
Phe/F: Phenylalanine
RMSD: Root mean standard deviation
Kd: Dissociation constant
