# Supplementary material for: Targeting asparagine and cysteine in SARS-CoV-2 variants and human pro-inflammatory mediators to alleviate COVID-19 severity; a cross-section and in-silico study
Source: Sci Rep. 2025 Nov 3;15:38445. doi: 10.1038/s41598-025-19359-y (PMC12583749; doi:10.1038/s41598-025-19359-y)
Supplement: Supplementary file 8 — Supplementary Material 8 [file 41598_2025_19359_MOESM8_ESM.pptx]

## Slide 1
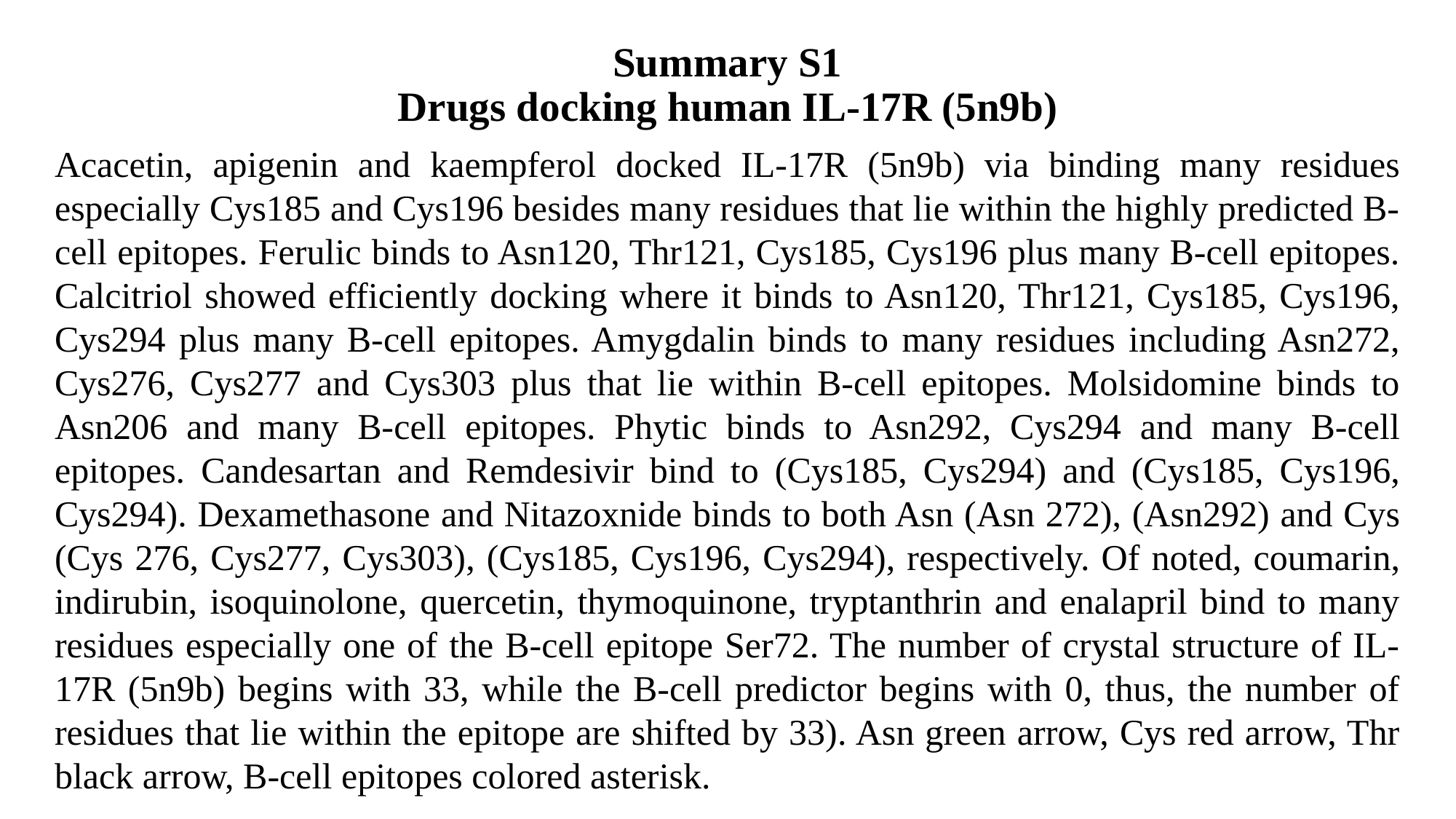

Summary S1
Drugs docking human IL-17R (5n9b)
Acacetin, apigenin and kaempferol docked IL-17R (5n9b) via binding many residues especially Cys185 and Cys196 besides many residues that lie within the highly predicted B-cell epitopes. Ferulic binds to Asn120, Thr121, Cys185, Cys196 plus many B-cell epitopes. Calcitriol showed efficiently docking where it binds to Asn120, Thr121, Cys185, Cys196, Cys294 plus many B-cell epitopes. Amygdalin binds to many residues including Asn272, Cys276, Cys277 and Cys303 plus that lie within B-cell epitopes. Molsidomine binds to Asn206 and many B-cell epitopes. Phytic binds to Asn292, Cys294 and many B-cell epitopes. Candesartan and Remdesivir bind to (Cys185, Cys294) and (Cys185, Cys196, Cys294). Dexamethasone and Nitazoxnide binds to both Asn (Asn 272), (Asn292) and Cys (Cys 276, Cys277, Cys303), (Cys185, Cys196, Cys294), respectively. Of noted, coumarin, indirubin, isoquinolone, quercetin, thymoquinone, tryptanthrin and enalapril bind to many residues especially one of the B-cell epitope Ser72. The number of crystal structure of IL-17R (5n9b) begins with 33, while the B-cell predictor begins with 0, thus, the number of residues that lie within the epitope are shifted by 33). Asn green arrow, Cys red arrow, Thr black arrow, B-cell epitopes colored asterisk.

## Slide 2
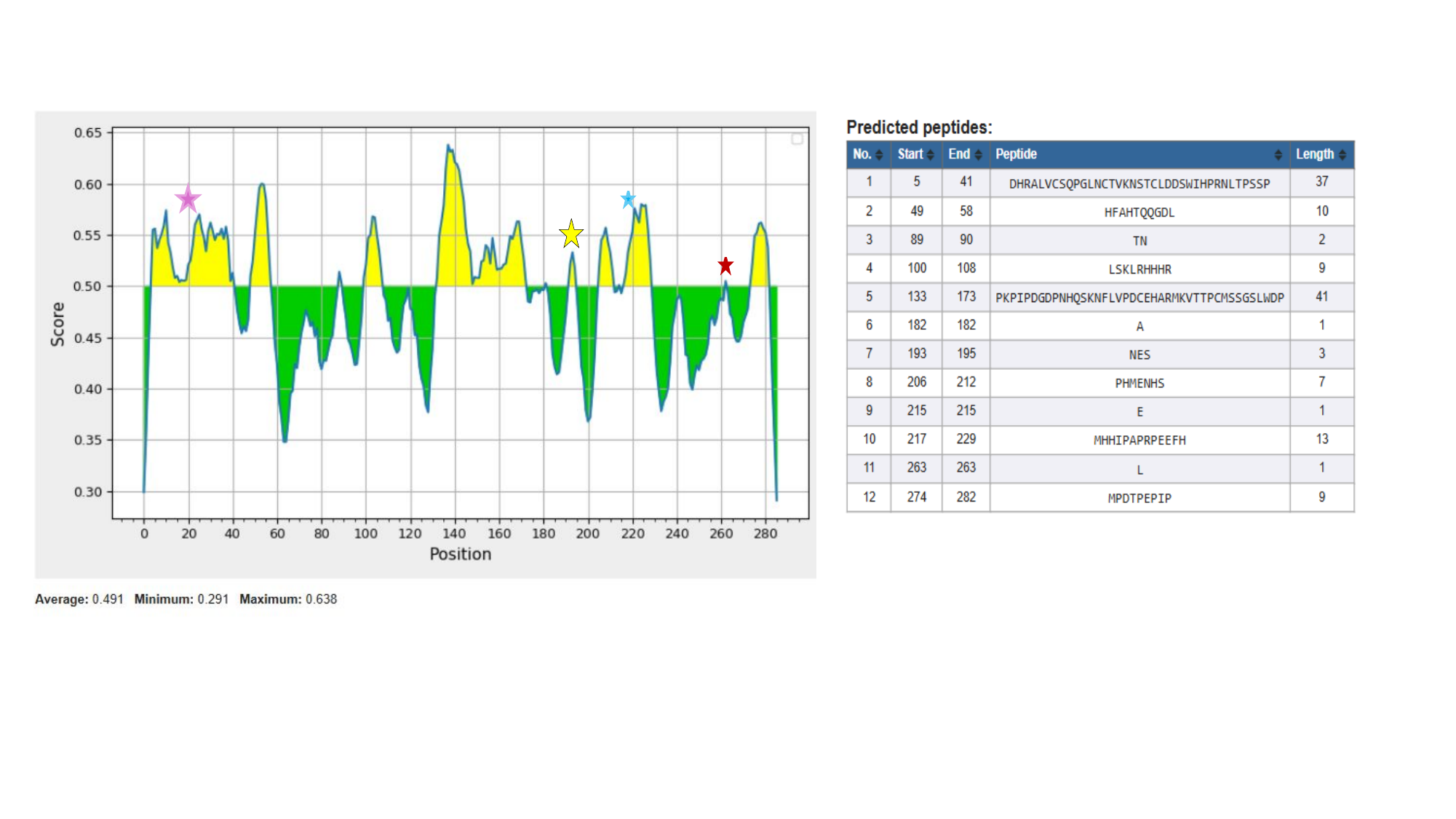

## Slide 3
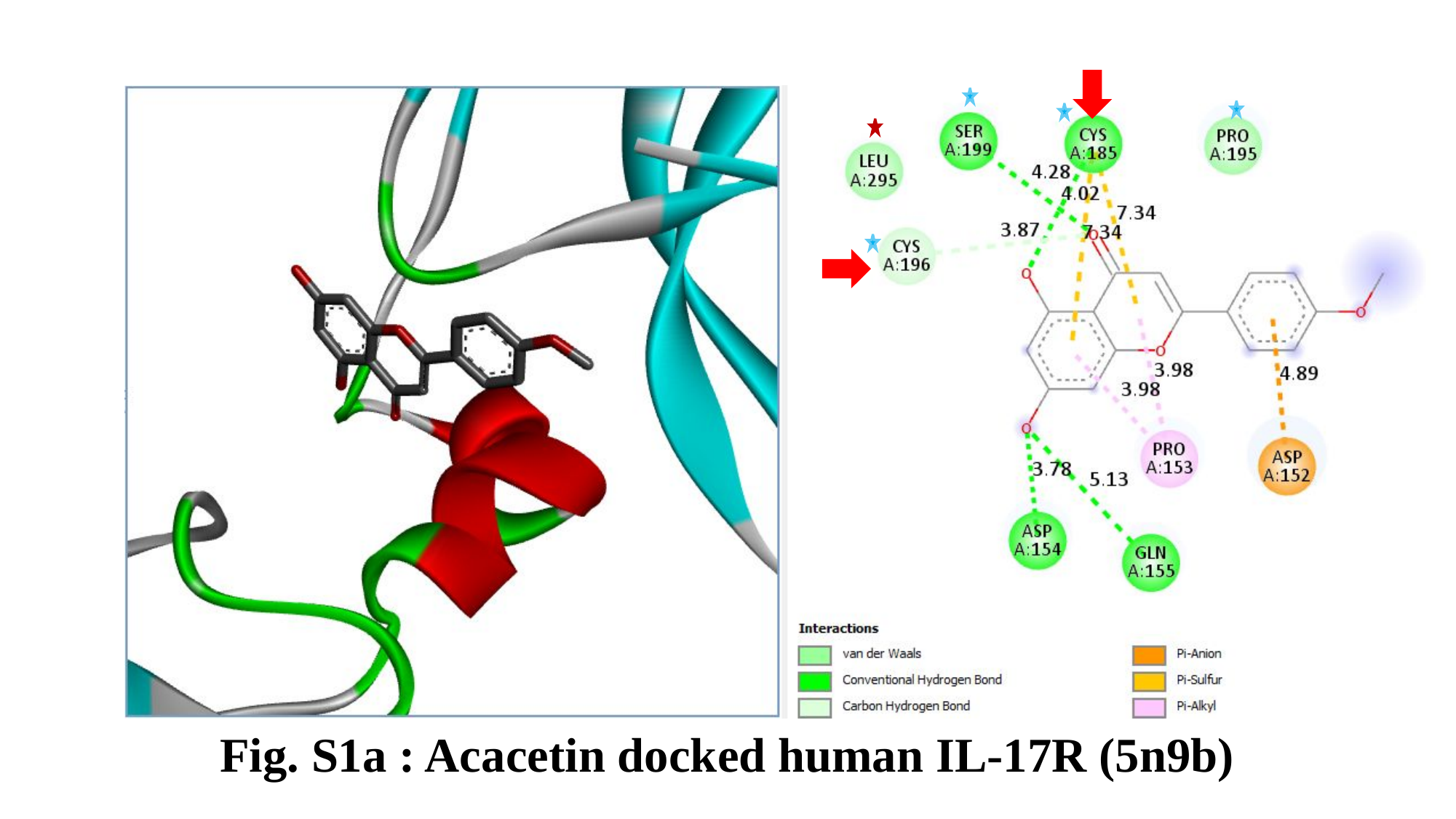

Fig. S1a : Acacetin docked human IL-17R (5n9b)

## Slide 4
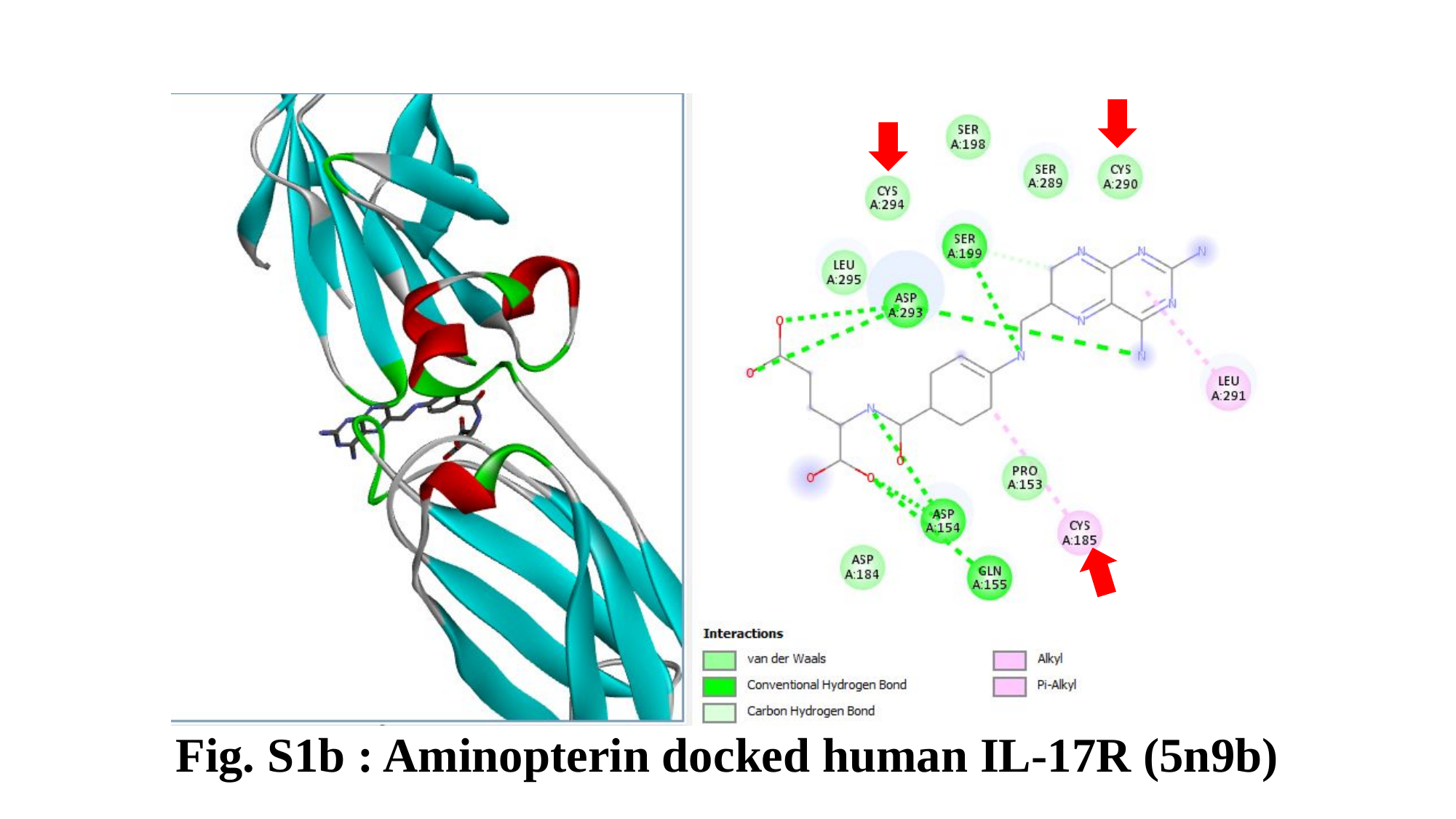

Fig. S1b : Aminopterin docked human IL-17R (5n9b)

## Slide 5
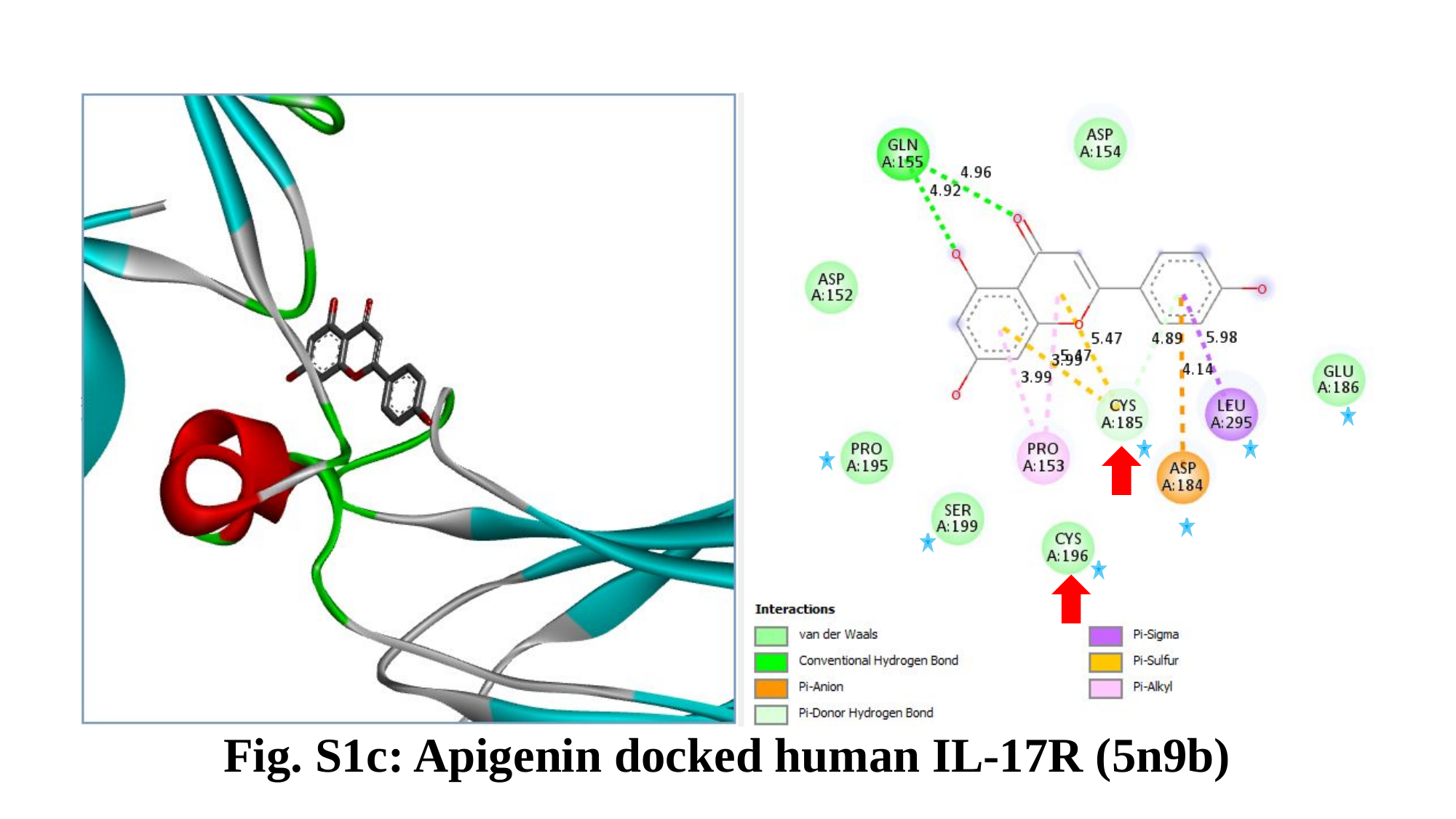

Fig. S1c: Apigenin docked human IL-17R (5n9b)

## Slide 6
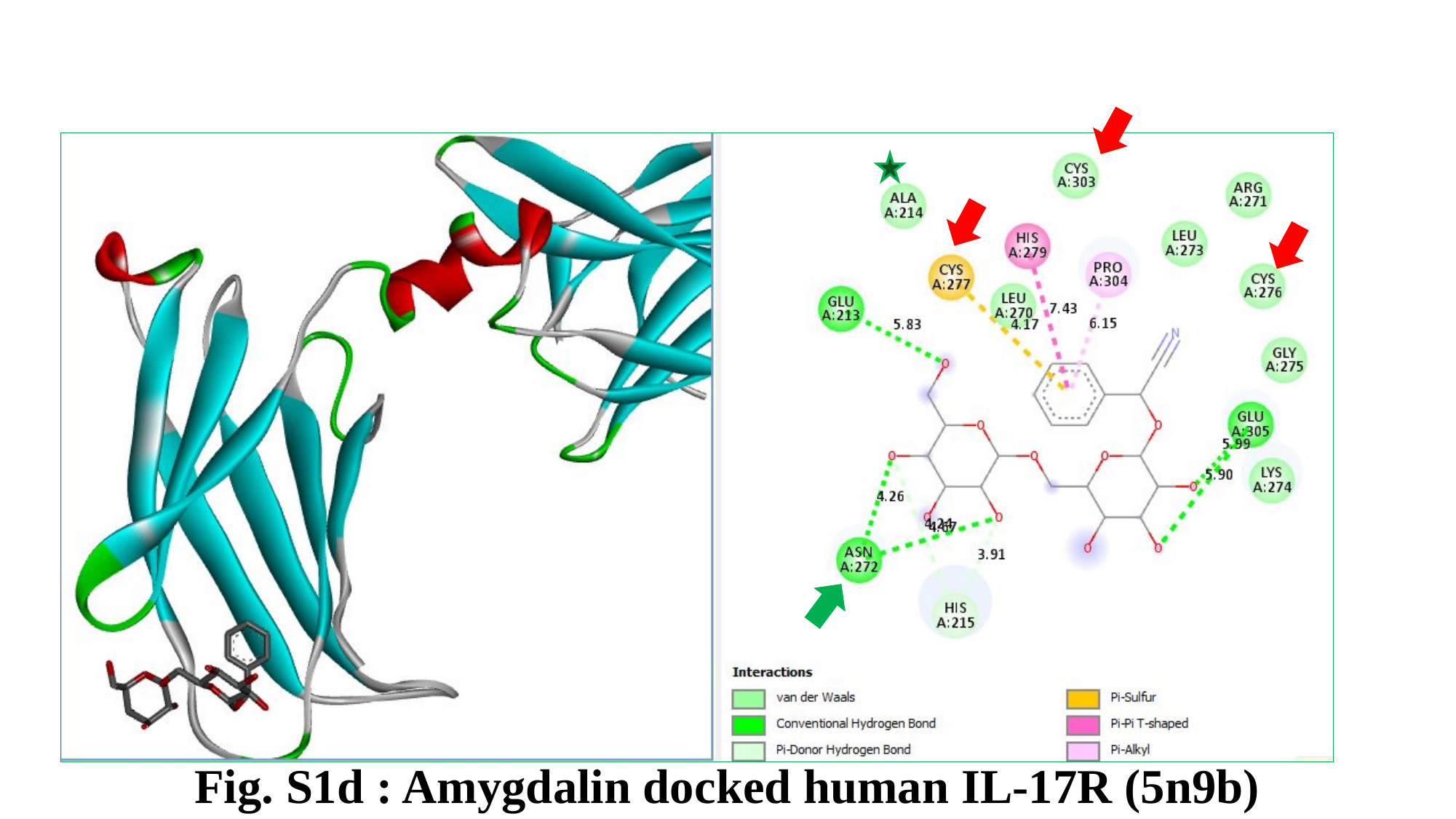

Fig. S1d : Amygdalin docked human IL-17R (5n9b)

## Slide 7
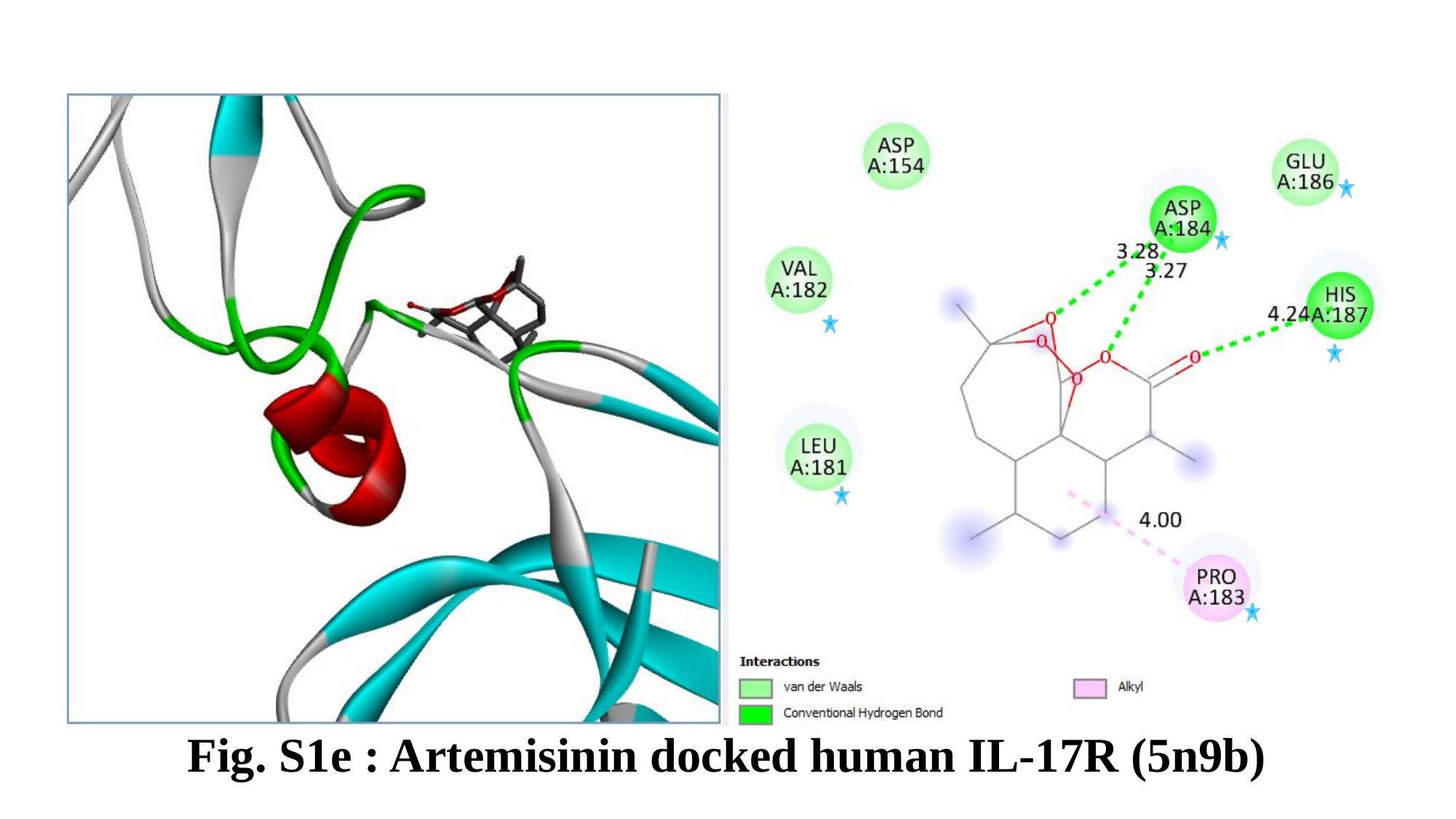

Fig. S1e : Artemisinin docked human IL-17R (5n9b)

## Slide 8
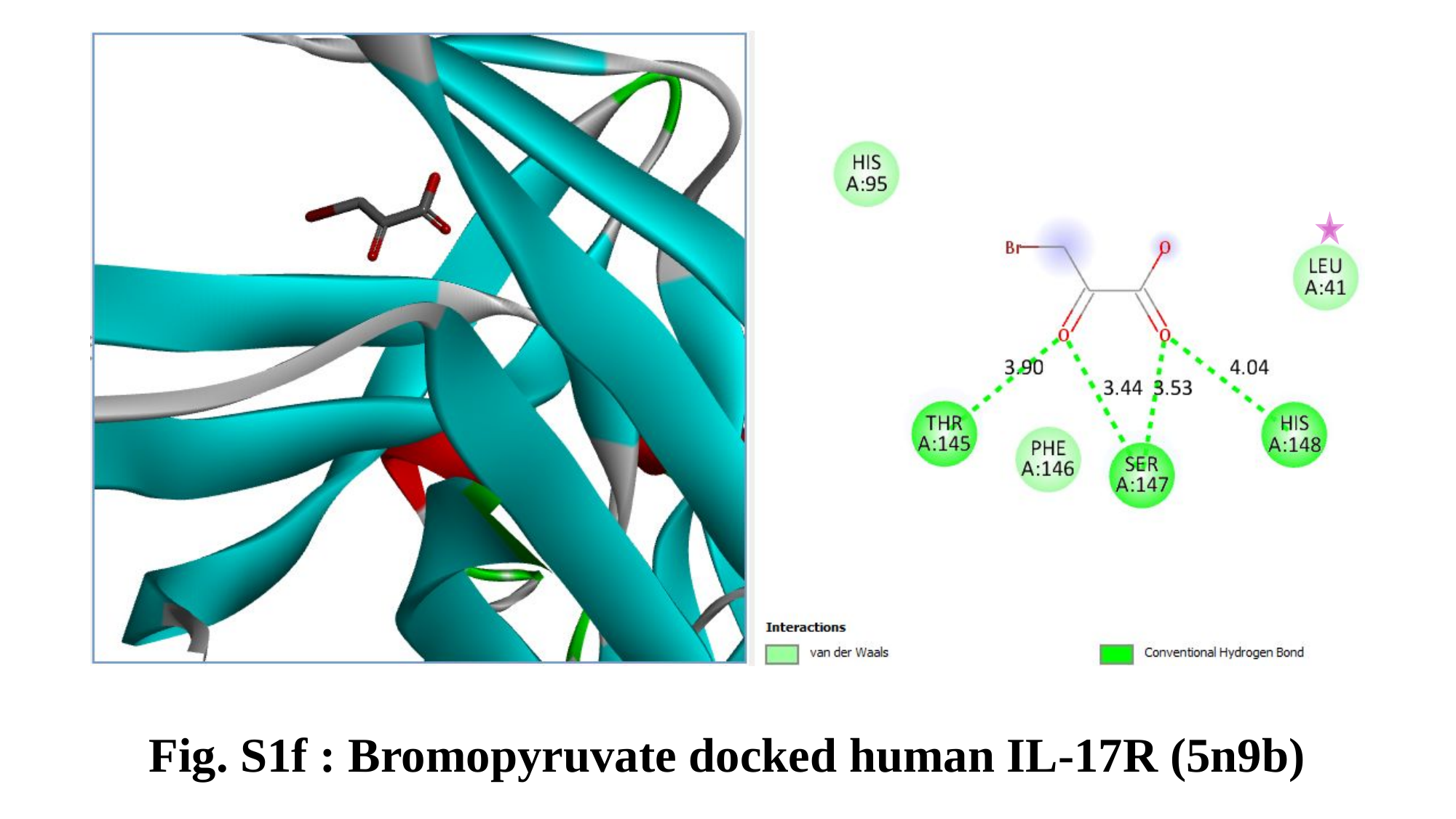

Fig. S1f : Bromopyruvate docked human IL-17R (5n9b)

## Slide 9
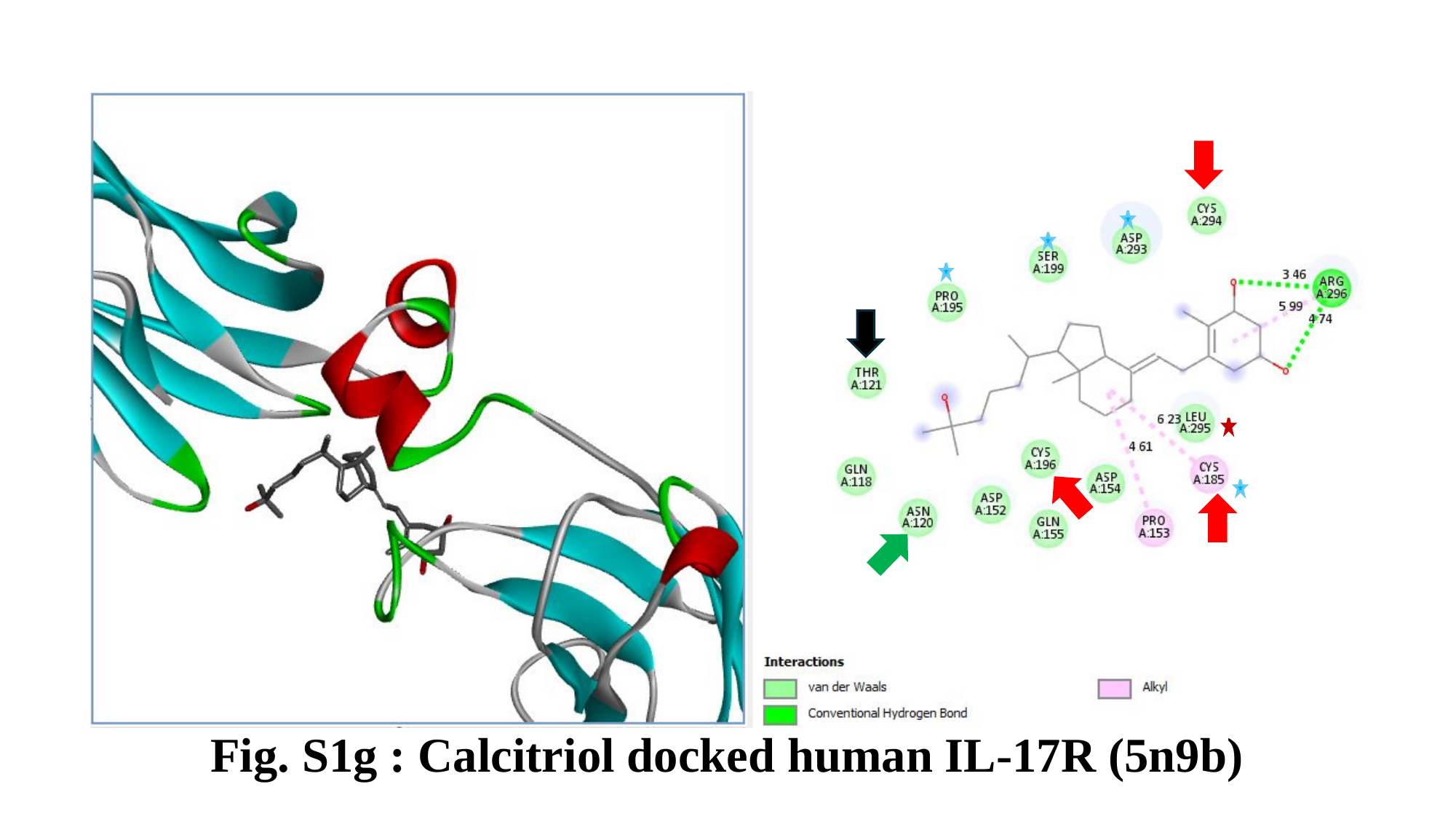

Fig. S1g : Calcitriol docked human IL-17R (5n9b)

## Slide 10
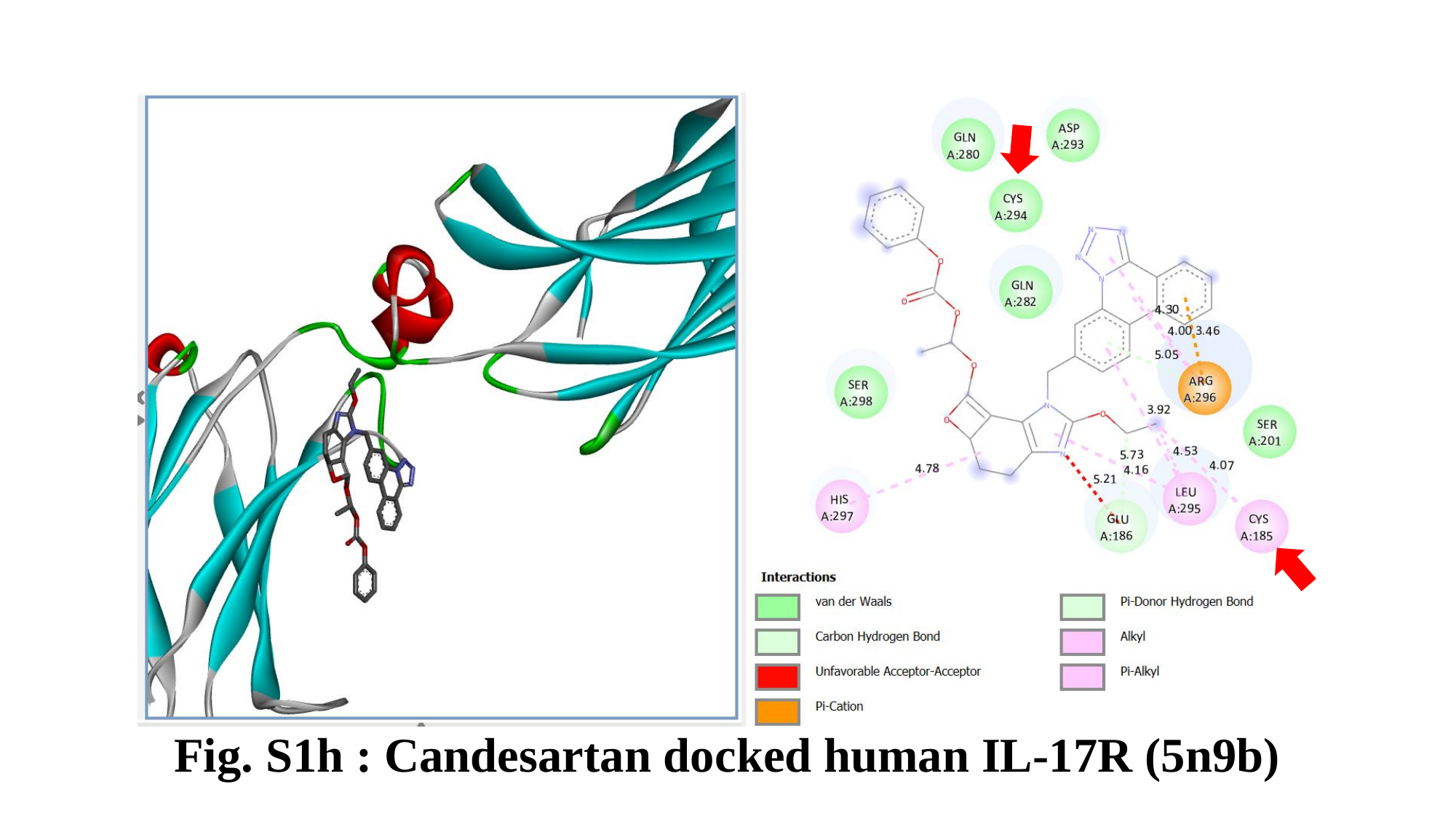

Fig. S1h : Candesartan docked human IL-17R (5n9b)

## Slide 11
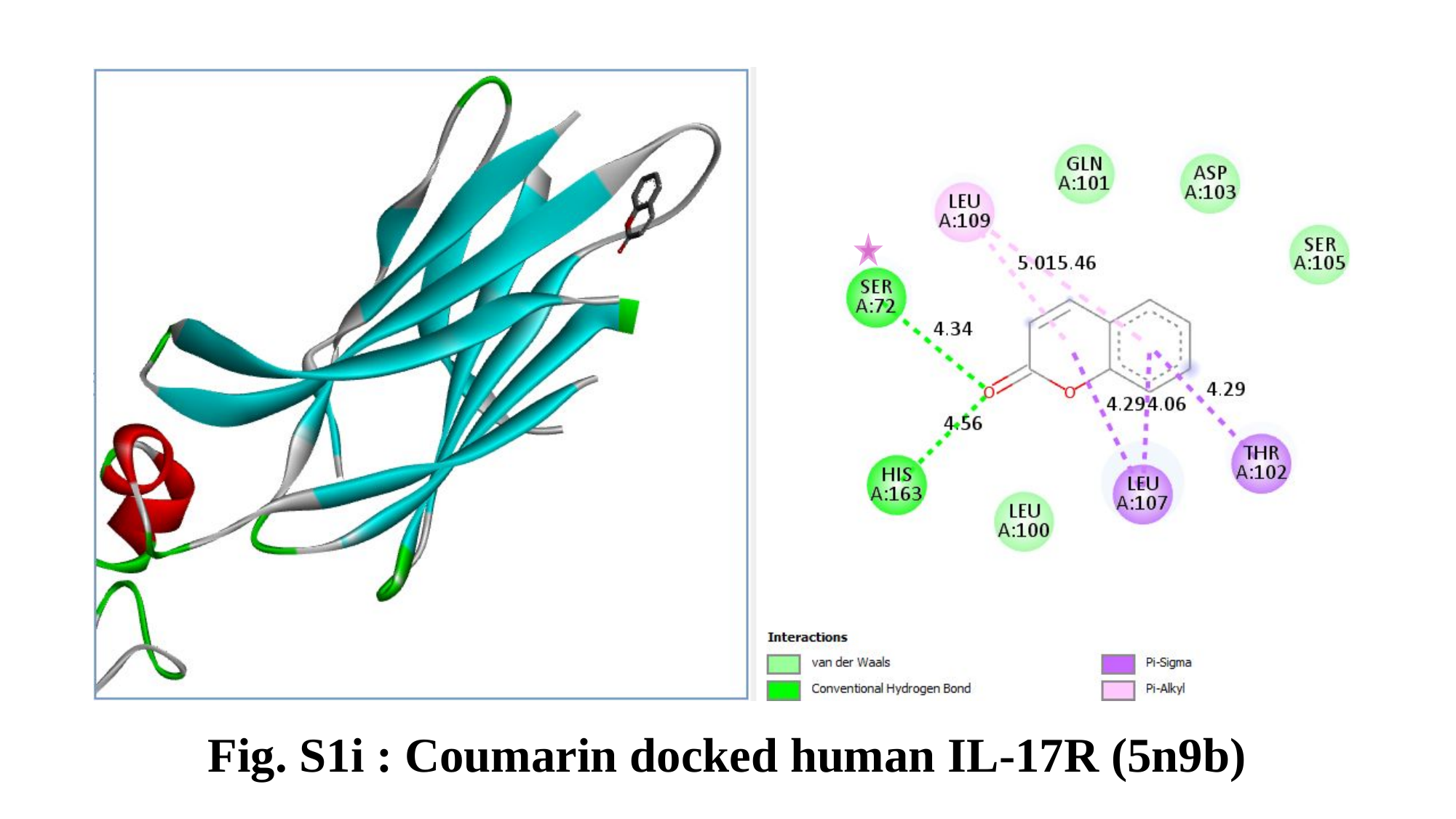

Fig. S1i : Coumarin docked human IL-17R (5n9b)

## Slide 12
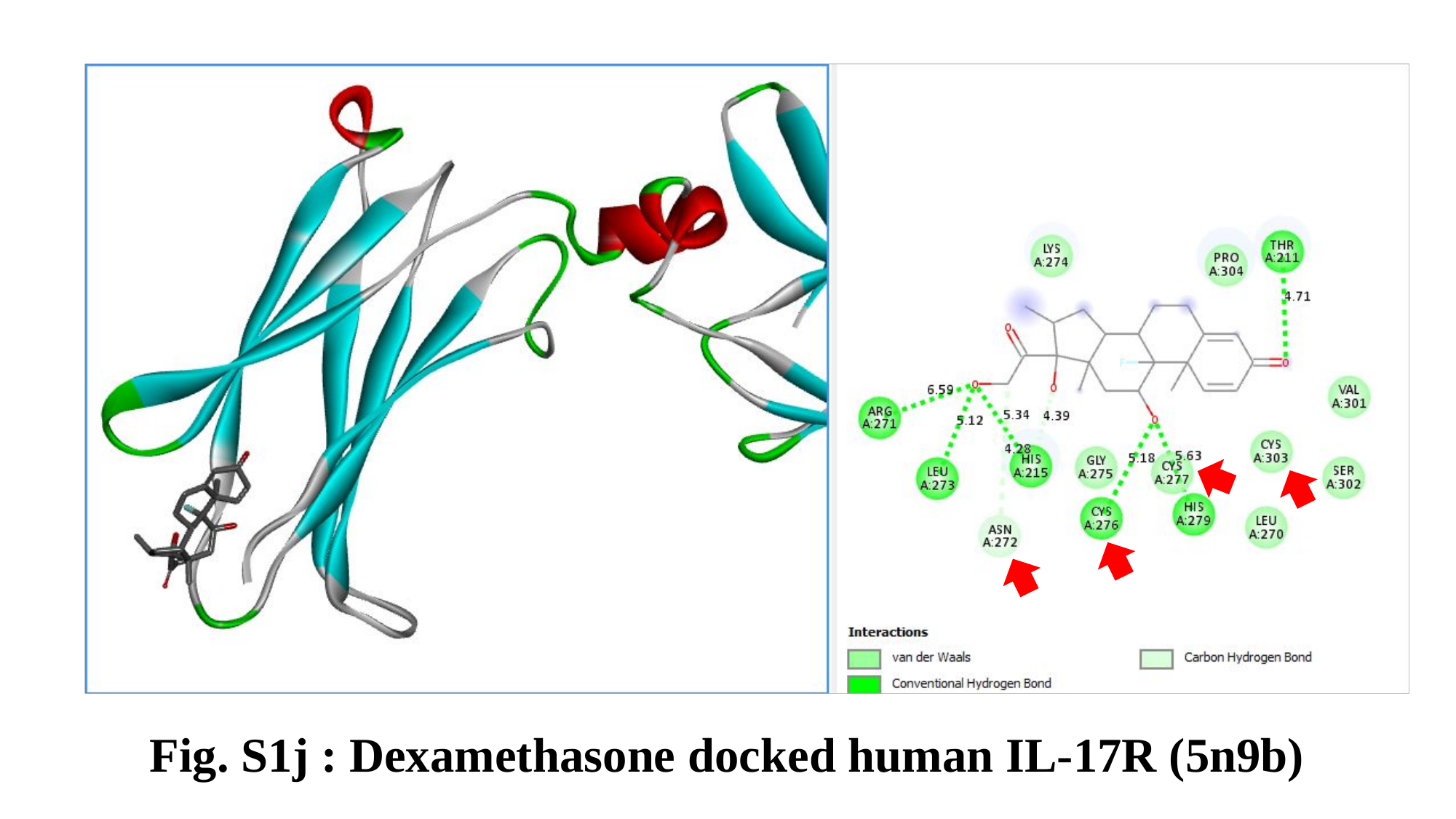

Fig. S1j : Dexamethasone docked human IL-17R (5n9b)

## Slide 13
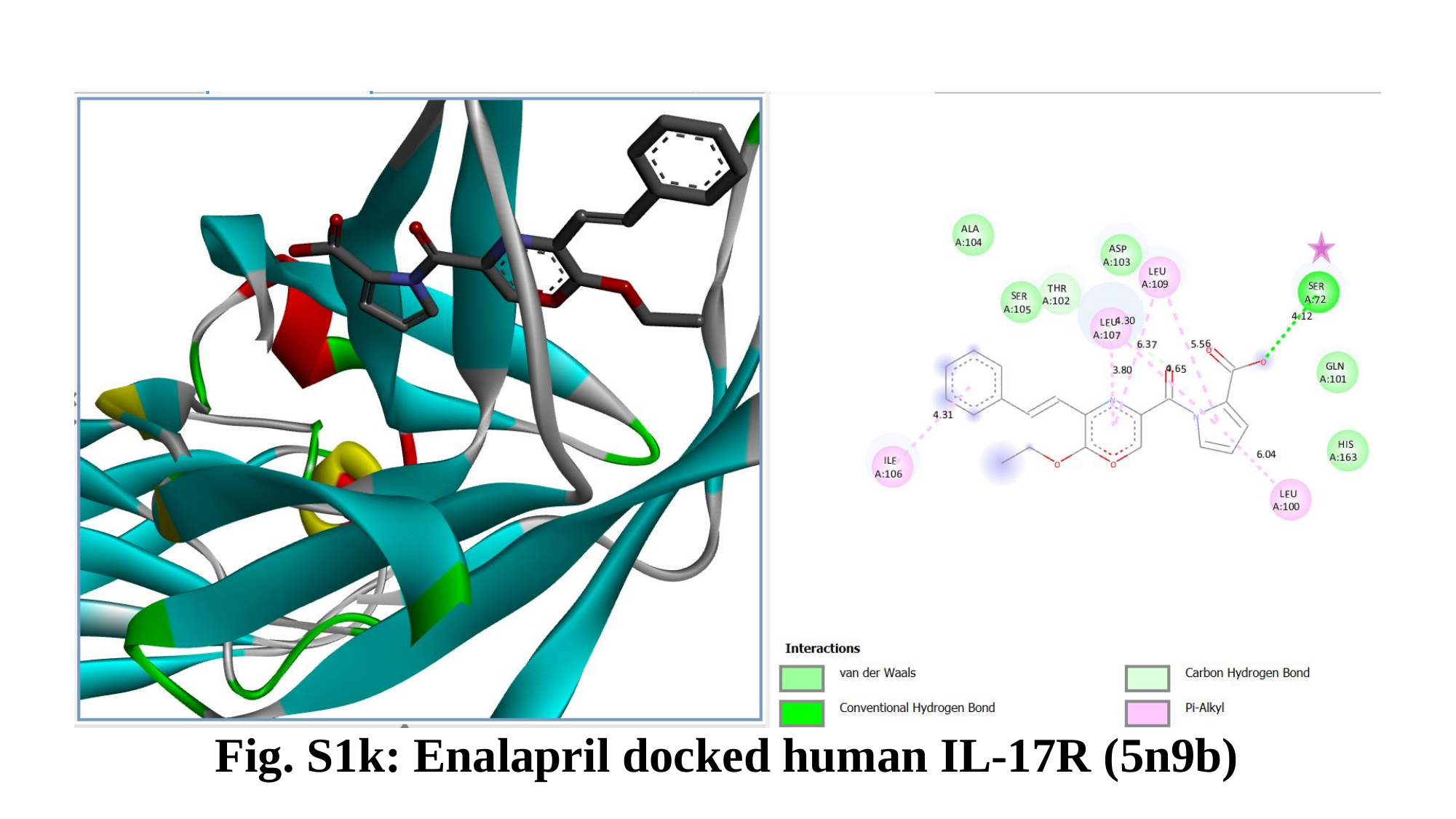

Fig. S1k: Enalapril docked human IL-17R (5n9b)

## Slide 14
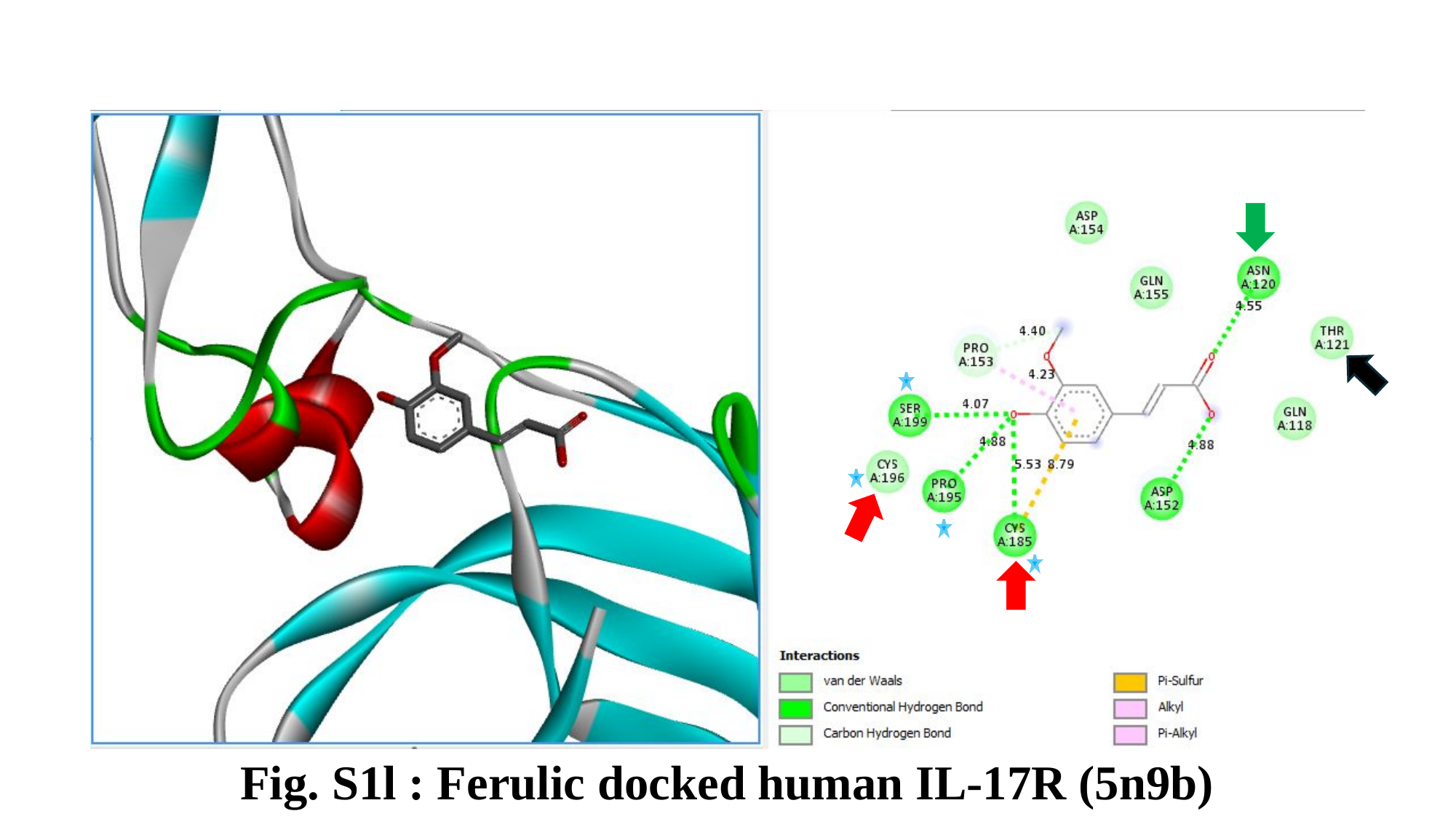

Fig. S1l : Ferulic docked human IL-17R (5n9b)

## Slide 15
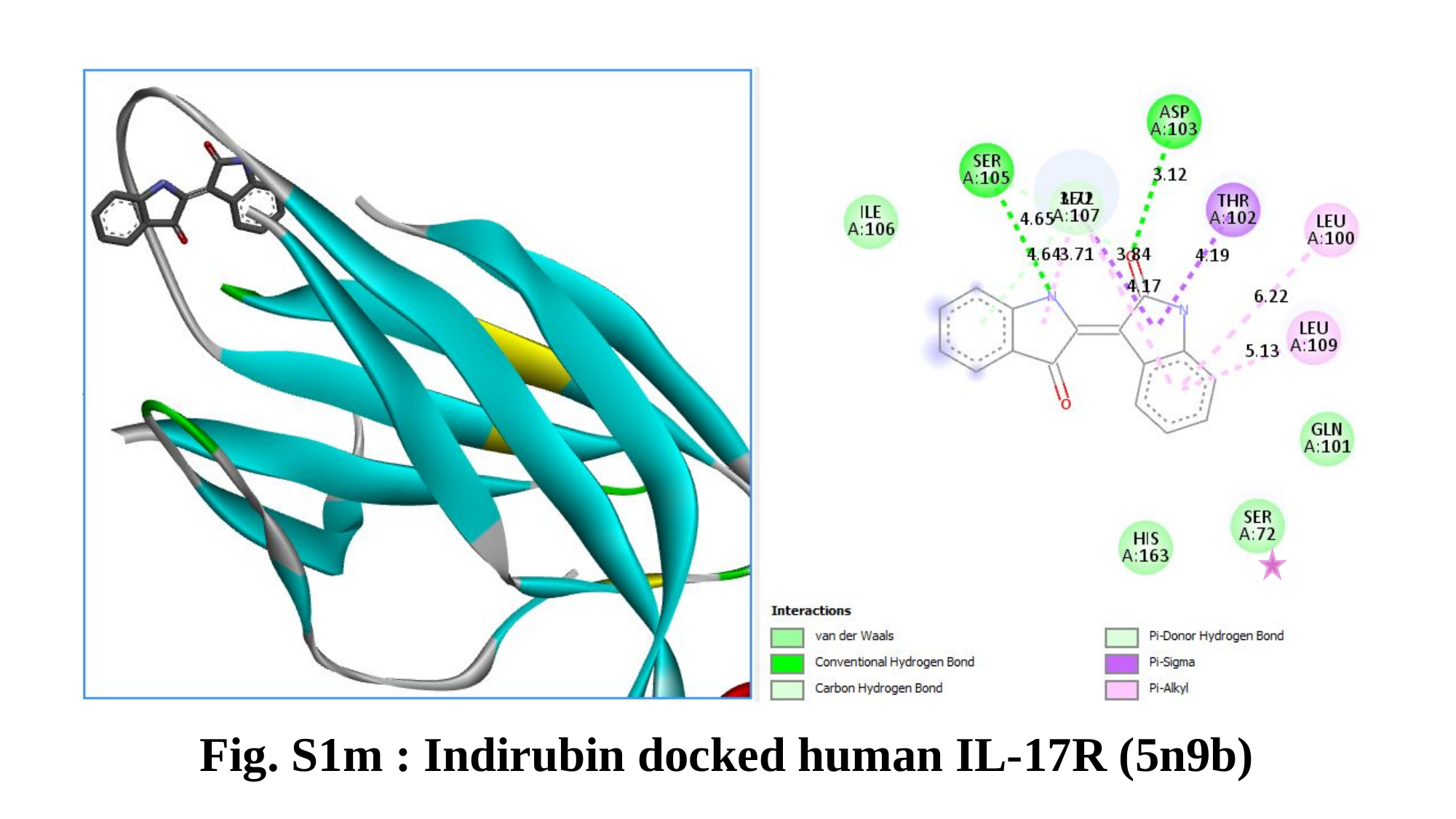

Fig. S1m : Indirubin docked human IL-17R (5n9b)

## Slide 16
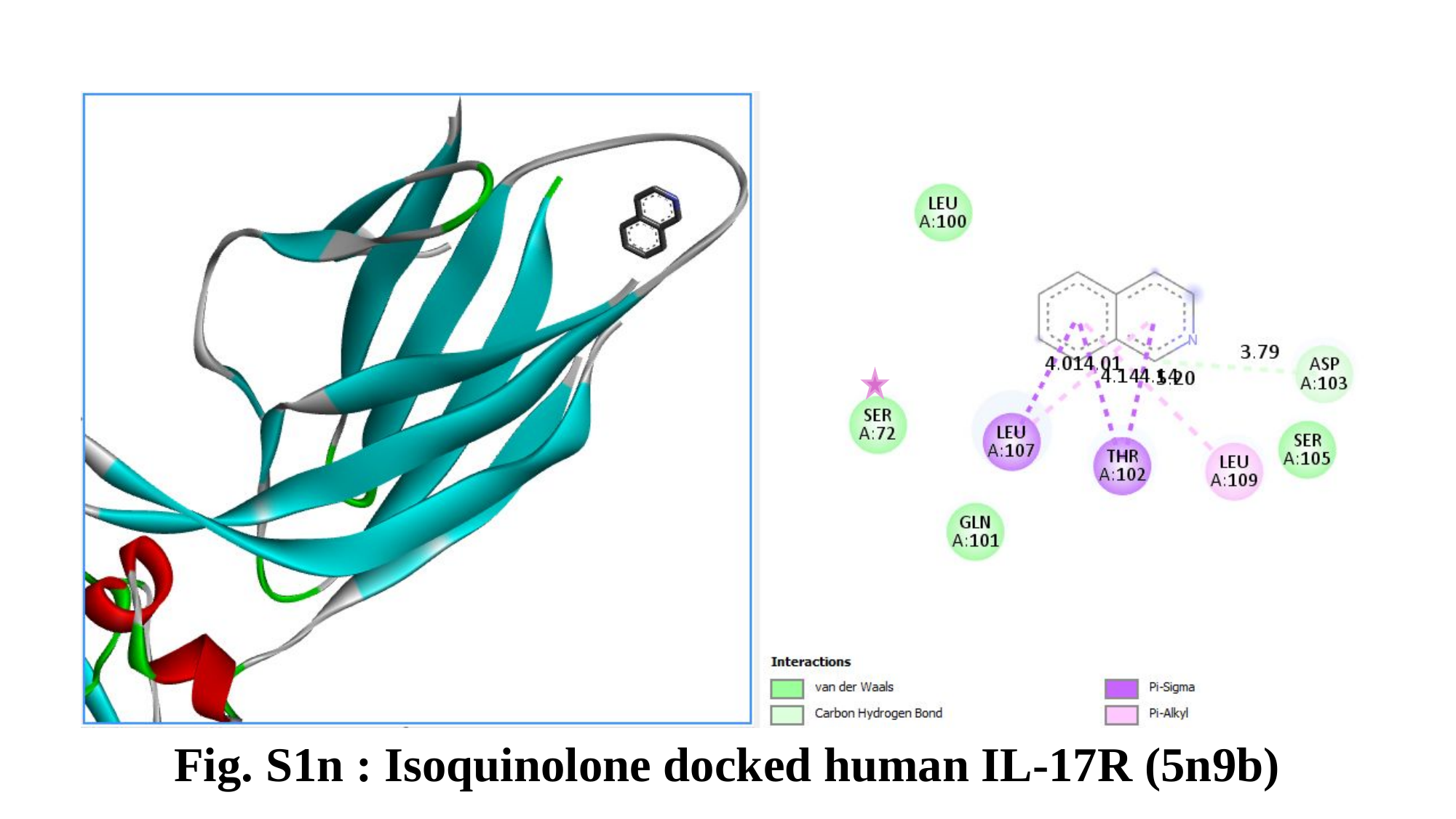

Fig. S1n : Isoquinolone docked human IL-17R (5n9b)

## Slide 17
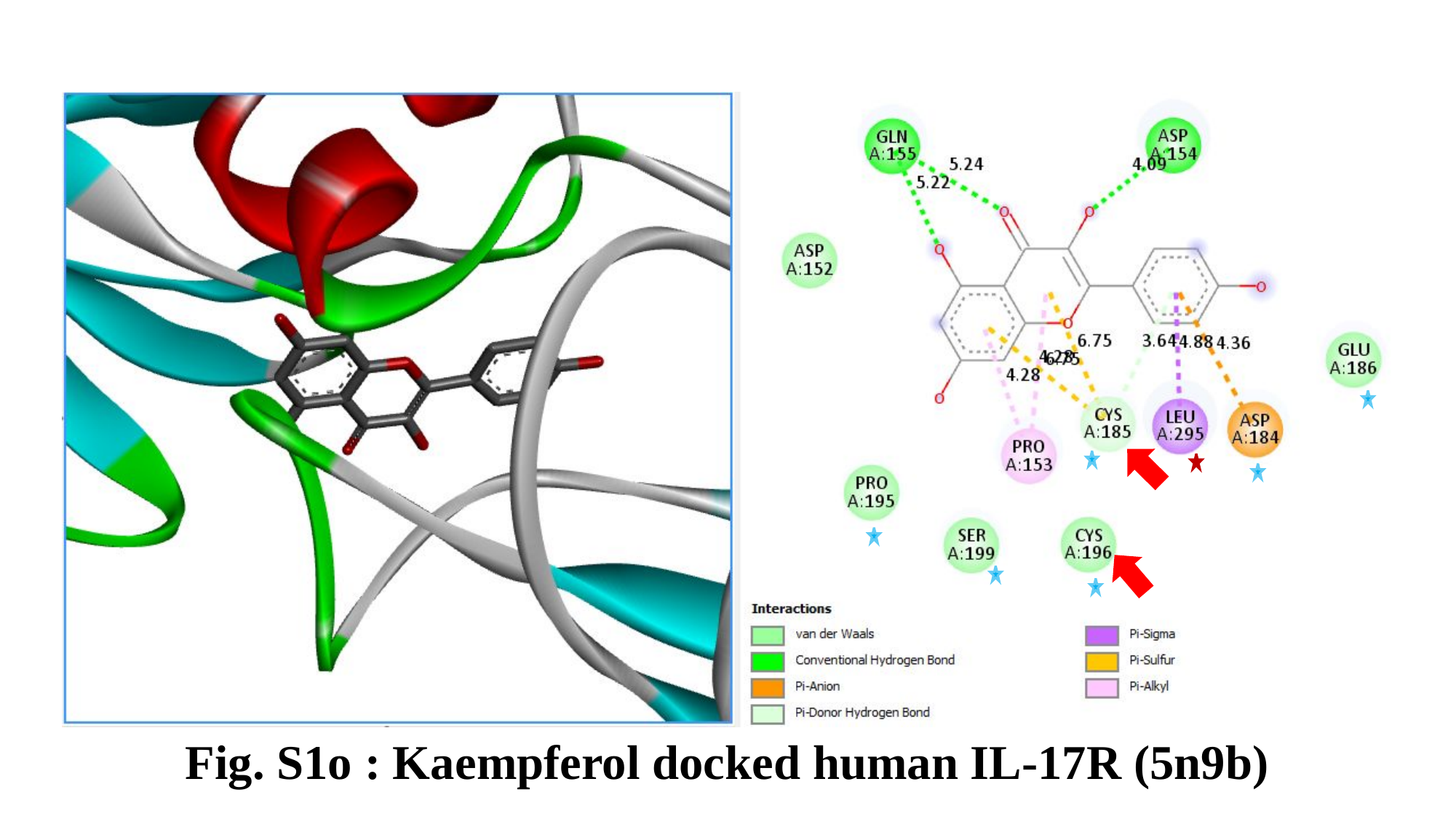

Fig. S1o : Kaempferol docked human IL-17R (5n9b)

## Slide 18
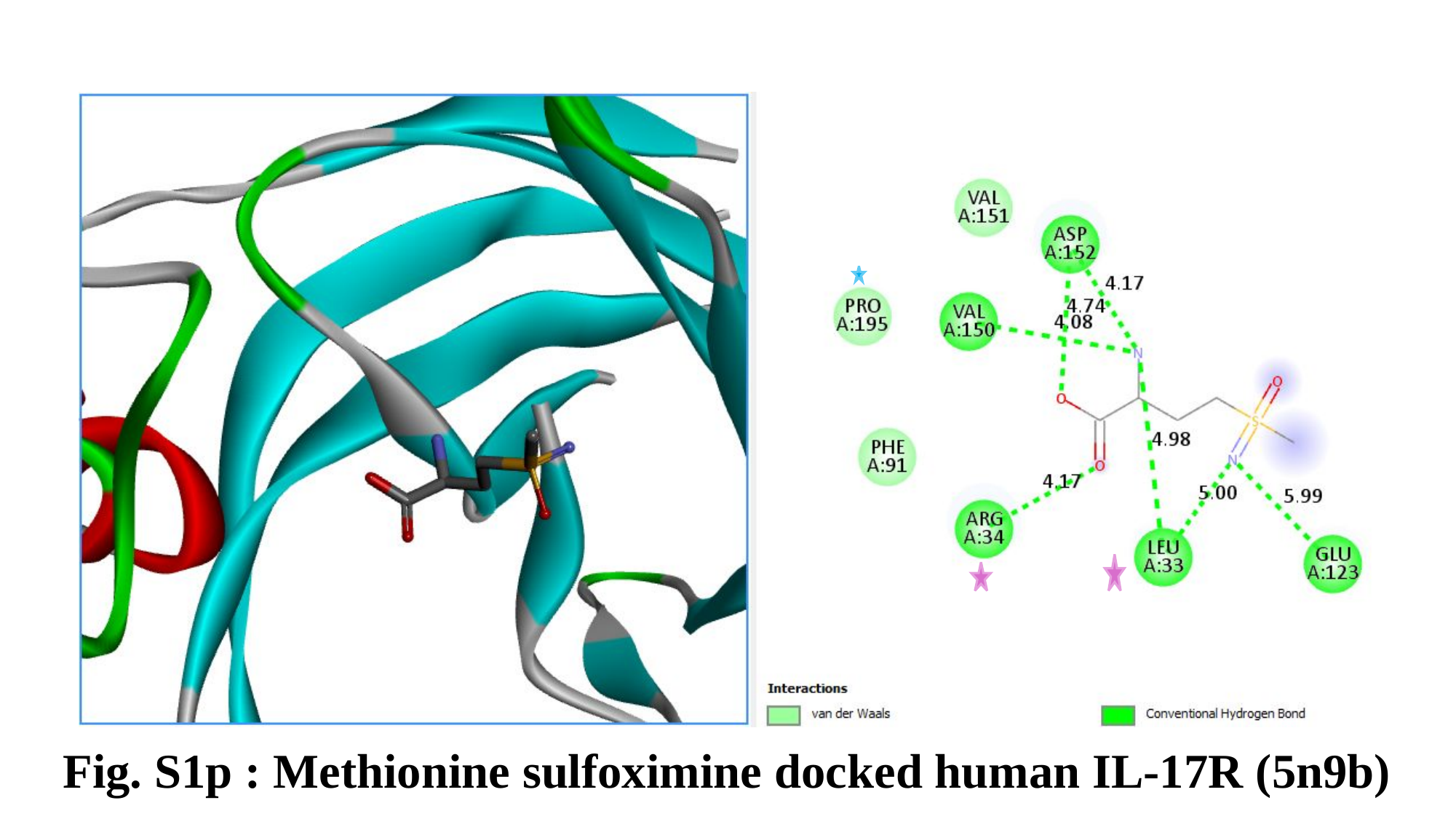

Fig. S1p : Methionine sulfoximine docked human IL-17R (5n9b)

## Slide 19
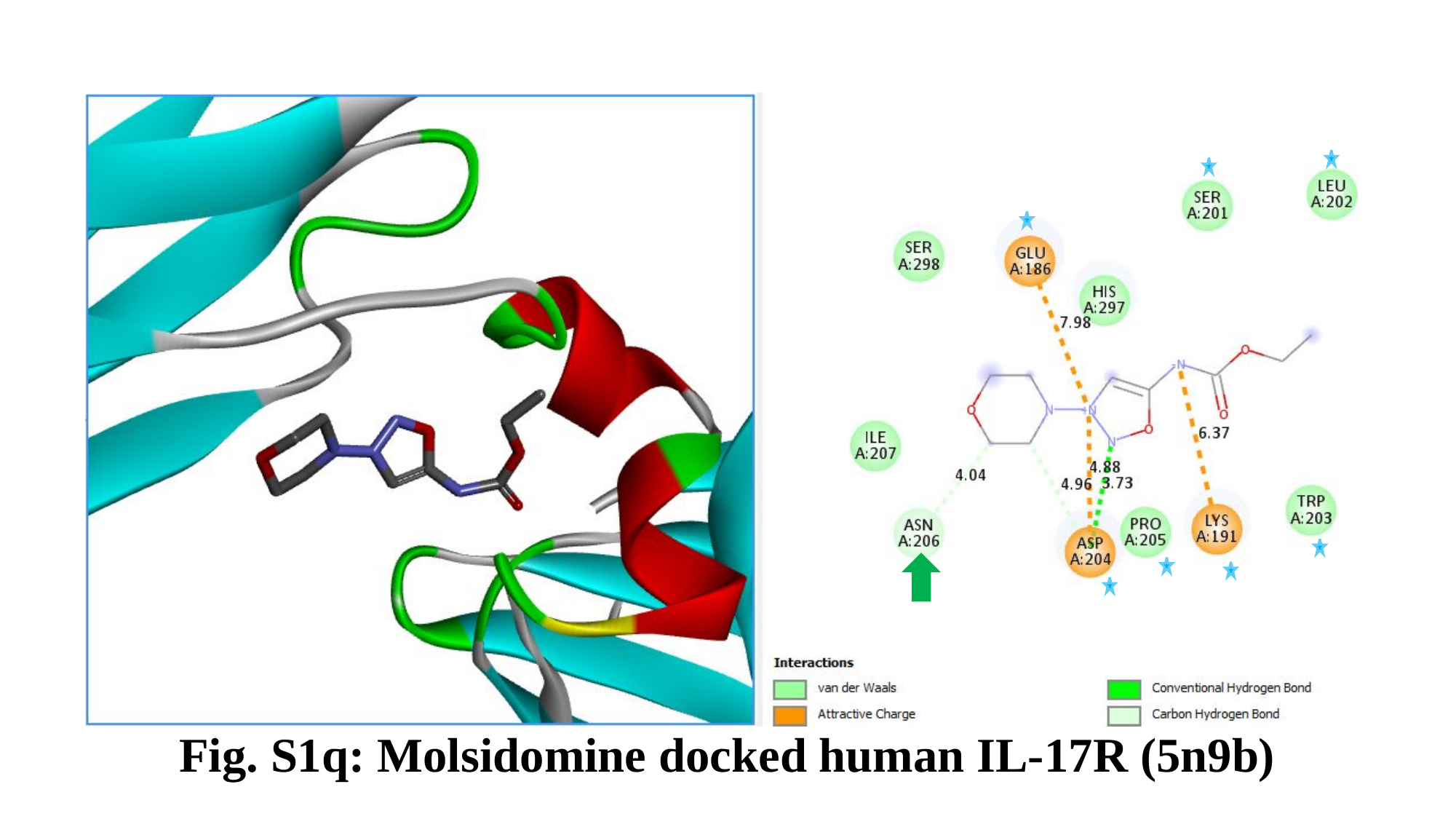

Fig. S1q: Molsidomine docked human IL-17R (5n9b)

## Slide 20
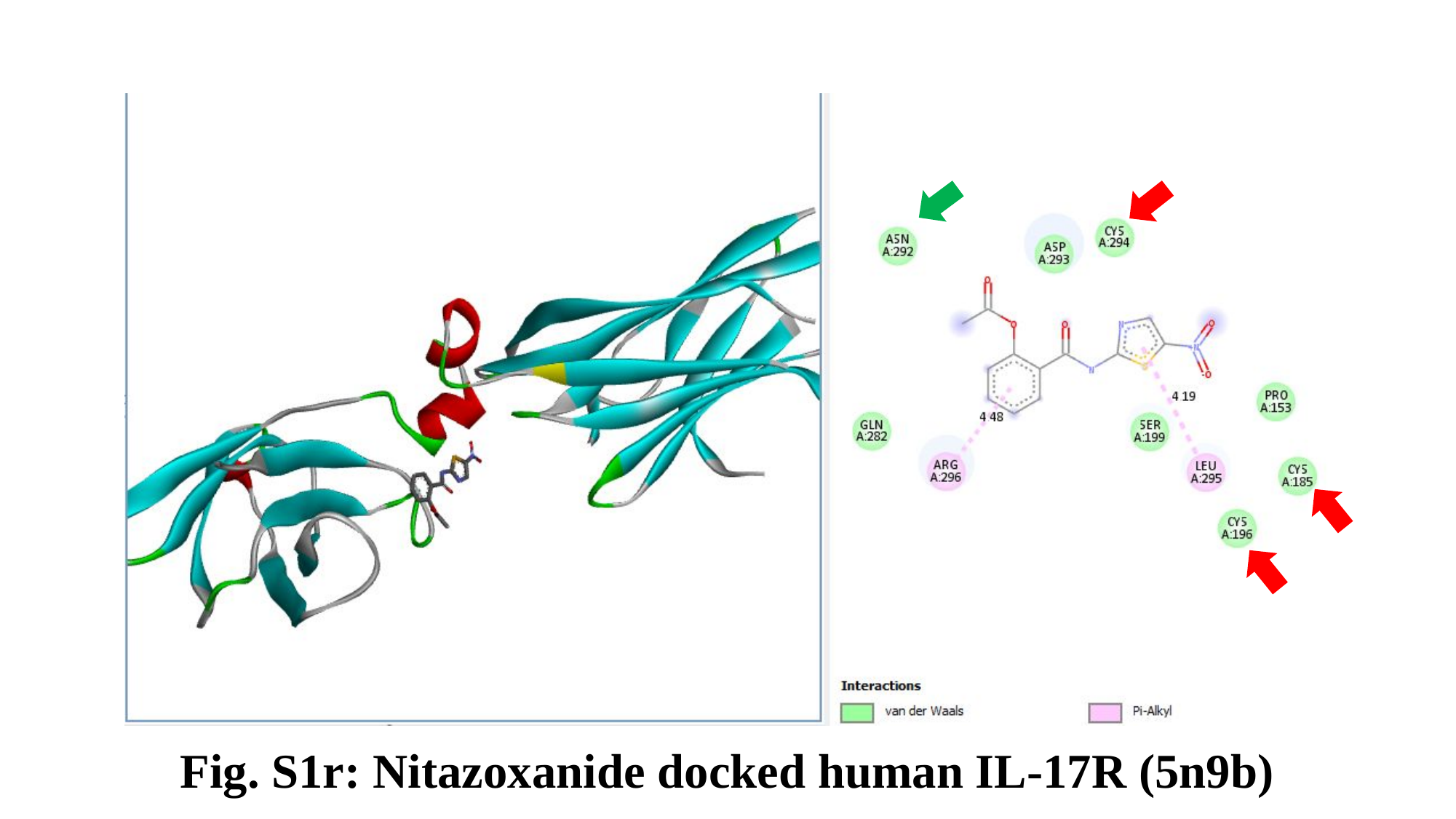

Fig. S1r: Nitazoxanide docked human IL-17R (5n9b)

## Slide 21
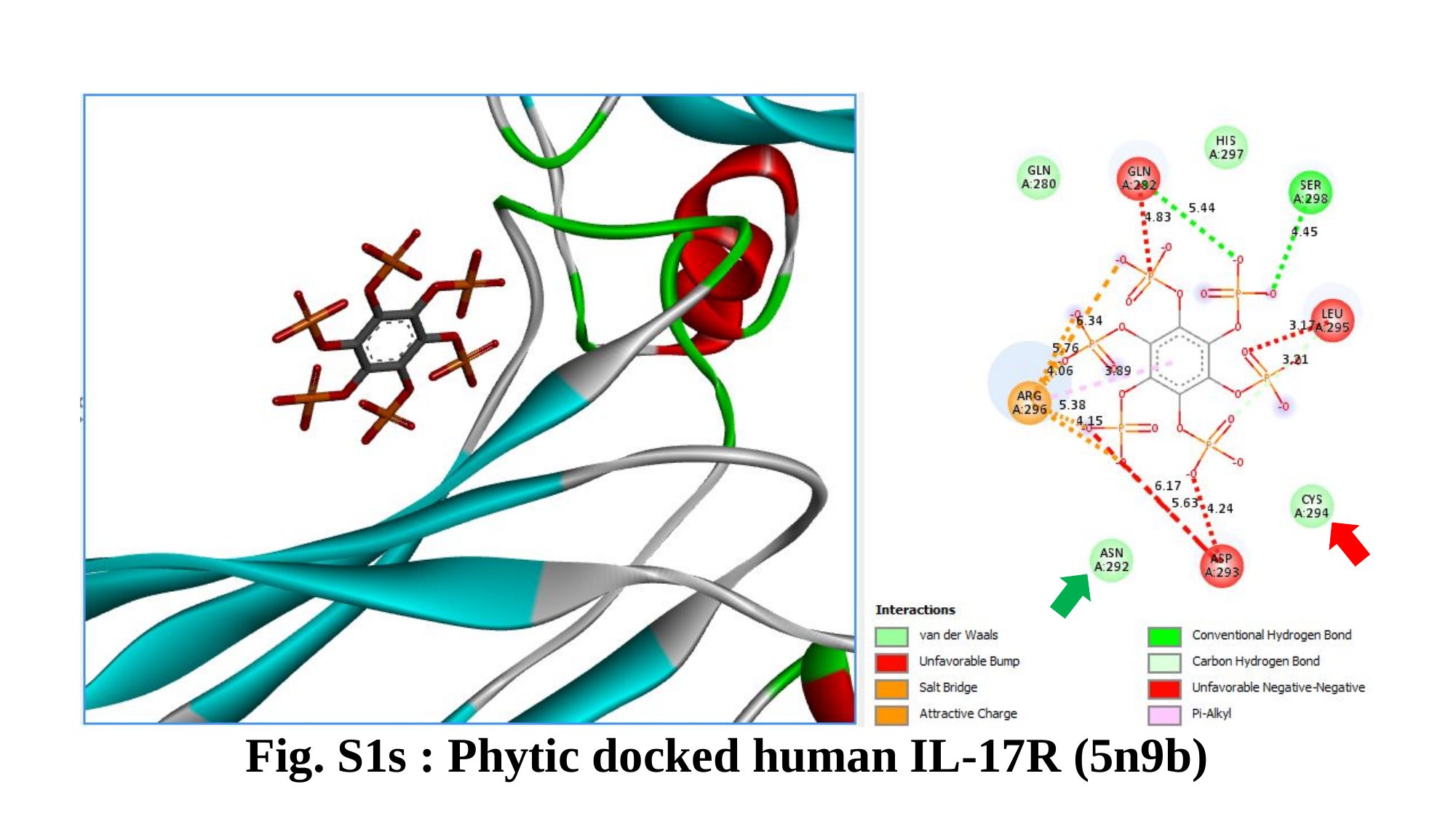

Fig. S1s : Phytic docked human IL-17R (5n9b)

## Slide 22
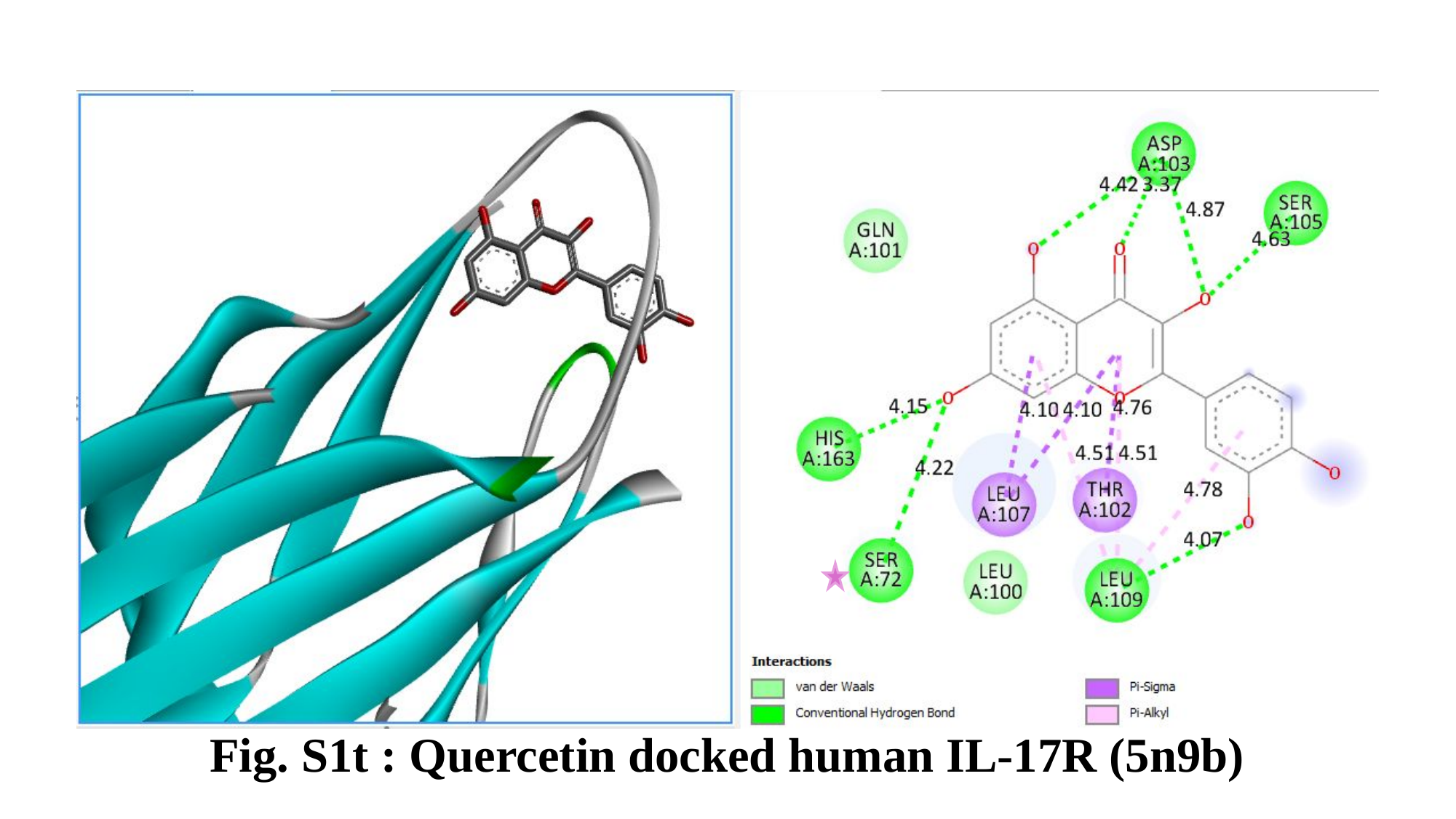

Fig. S1t : Quercetin docked human IL-17R (5n9b)

## Slide 23
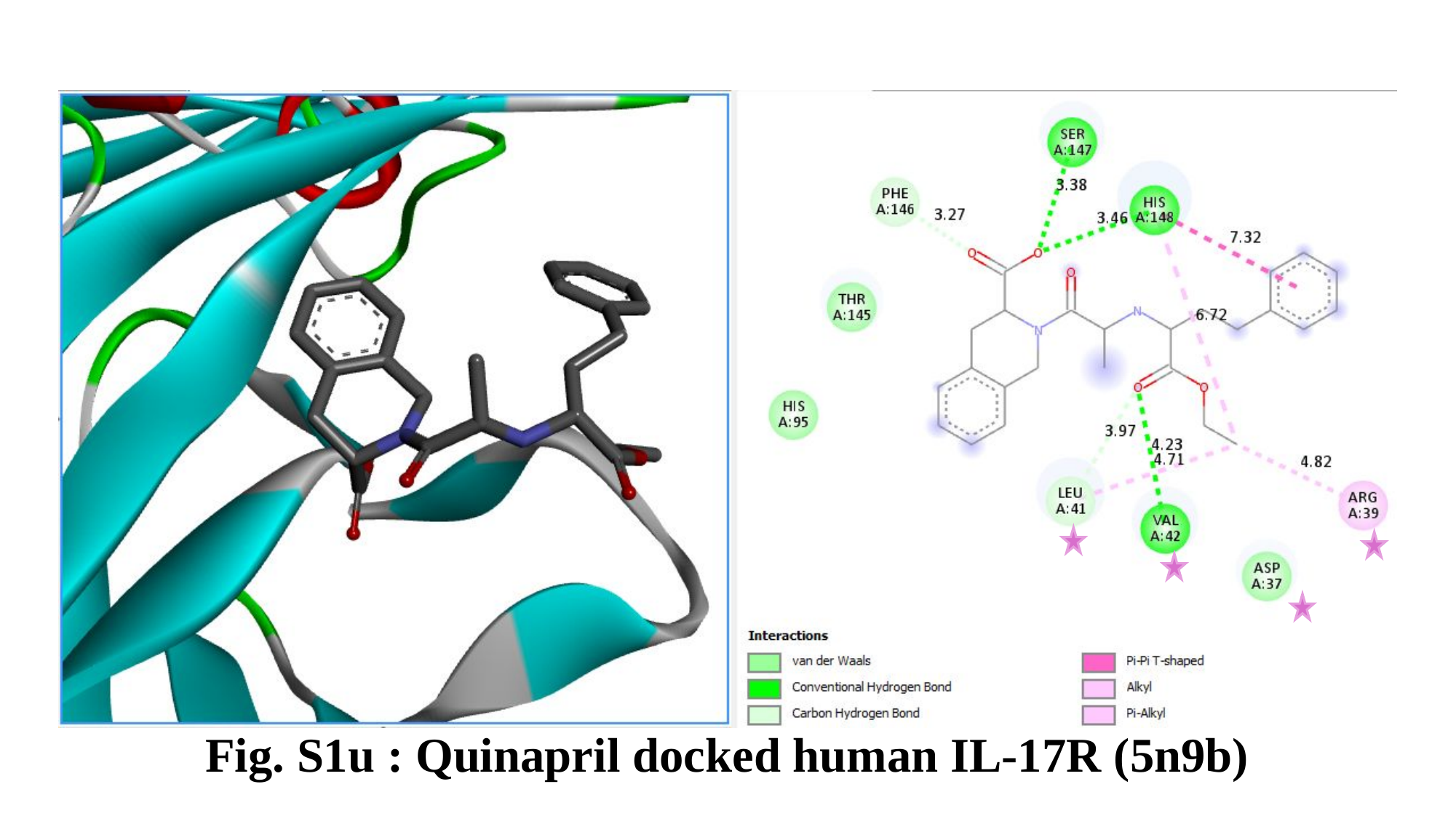

Fig. S1u : Quinapril docked human IL-17R (5n9b)

## Slide 24
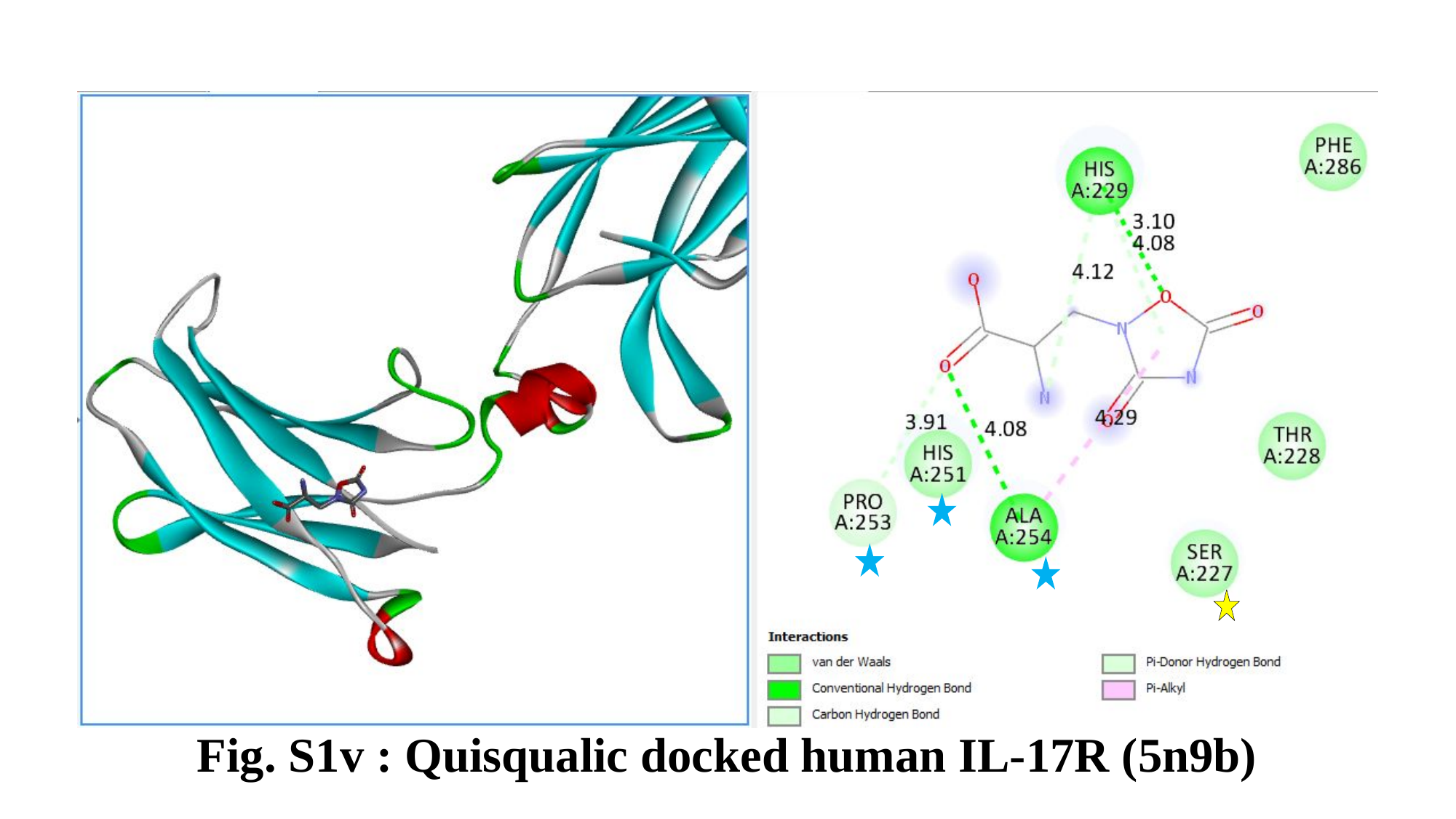

Fig. S1v : Quisqualic docked human IL-17R (5n9b)

## Slide 25
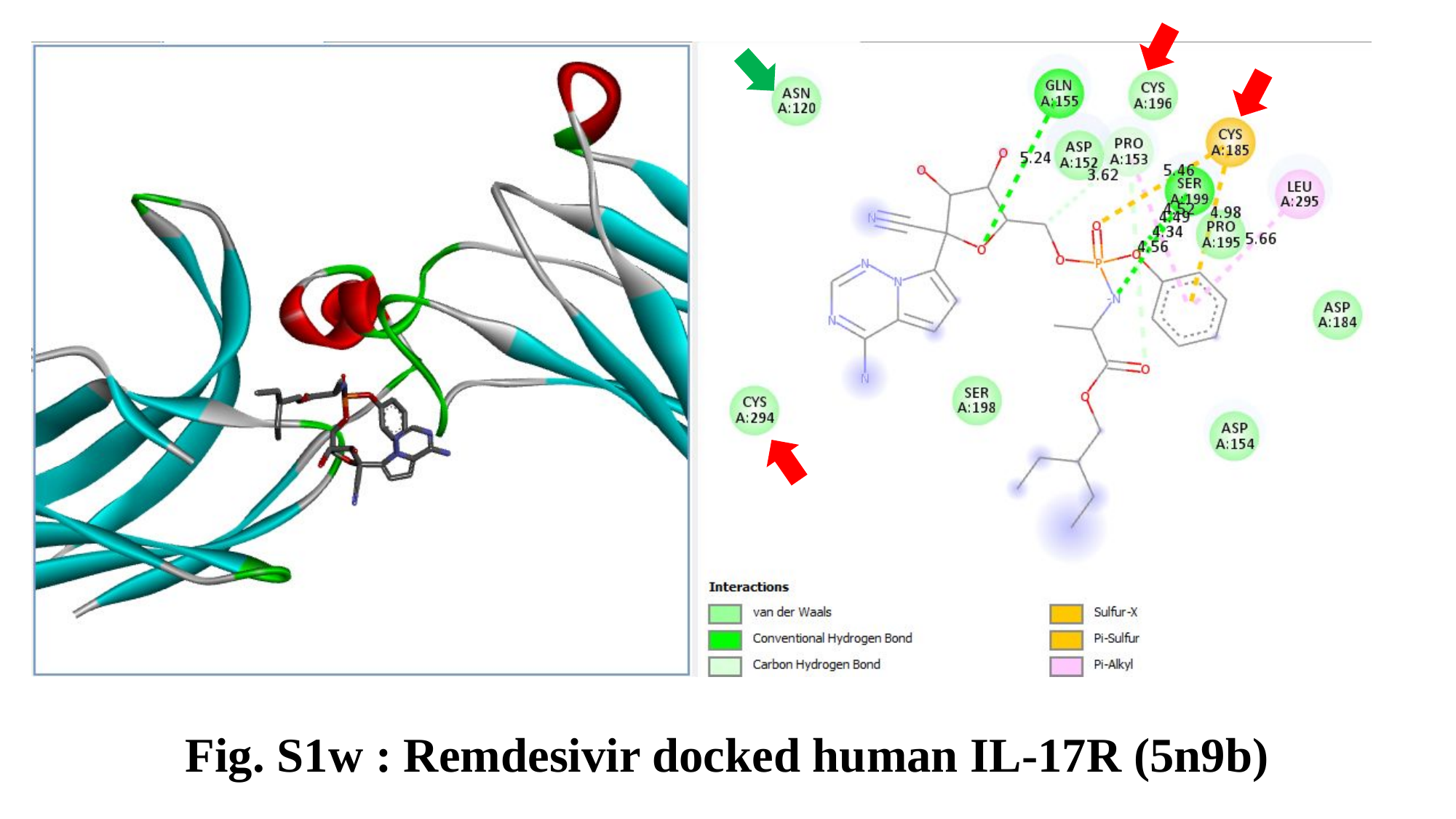

Fig. S1w : Remdesivir docked human IL-17R (5n9b)

## Slide 26
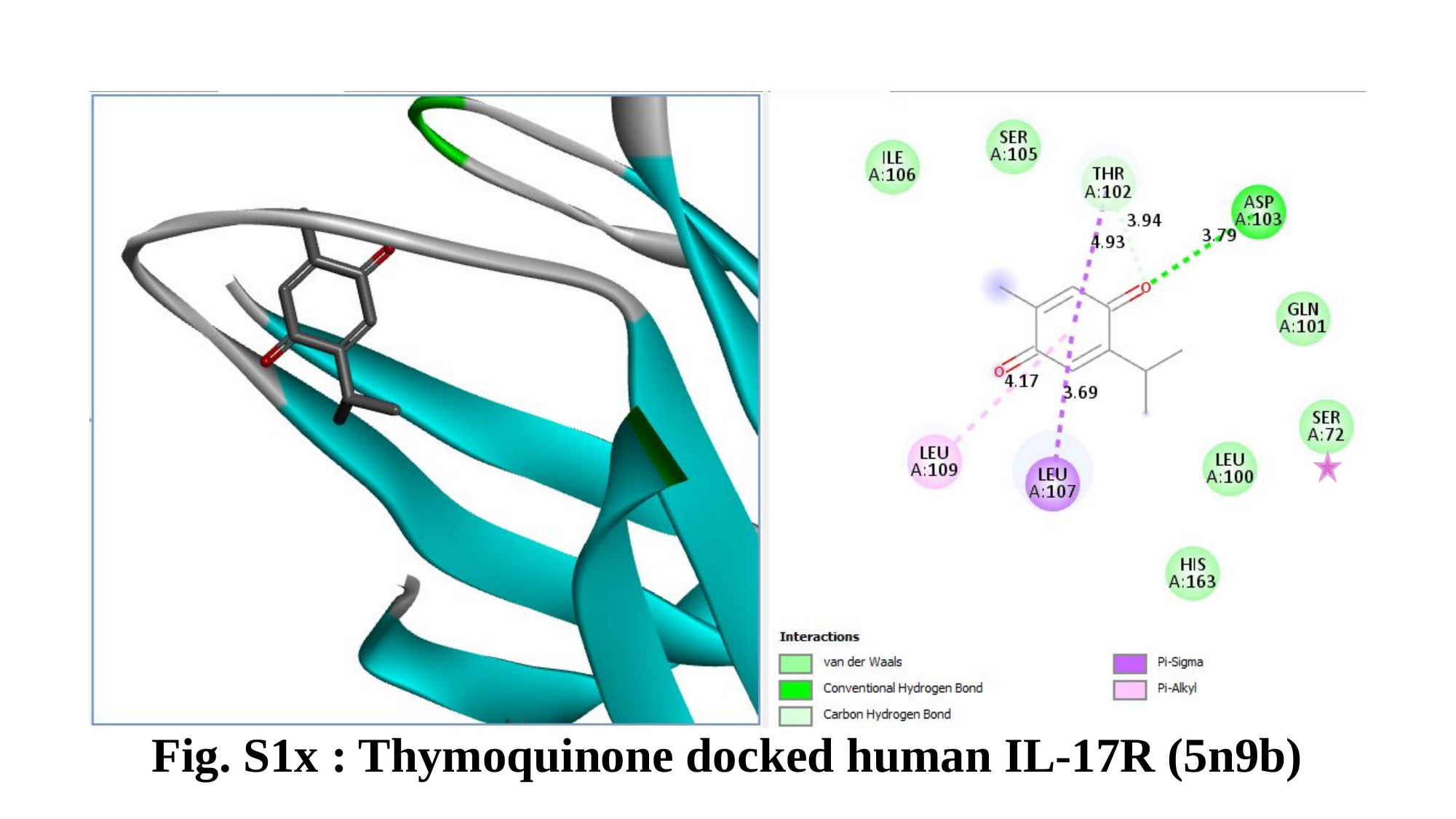

Fig. S1x : Thymoquinone docked human IL-17R (5n9b)

## Slide 27
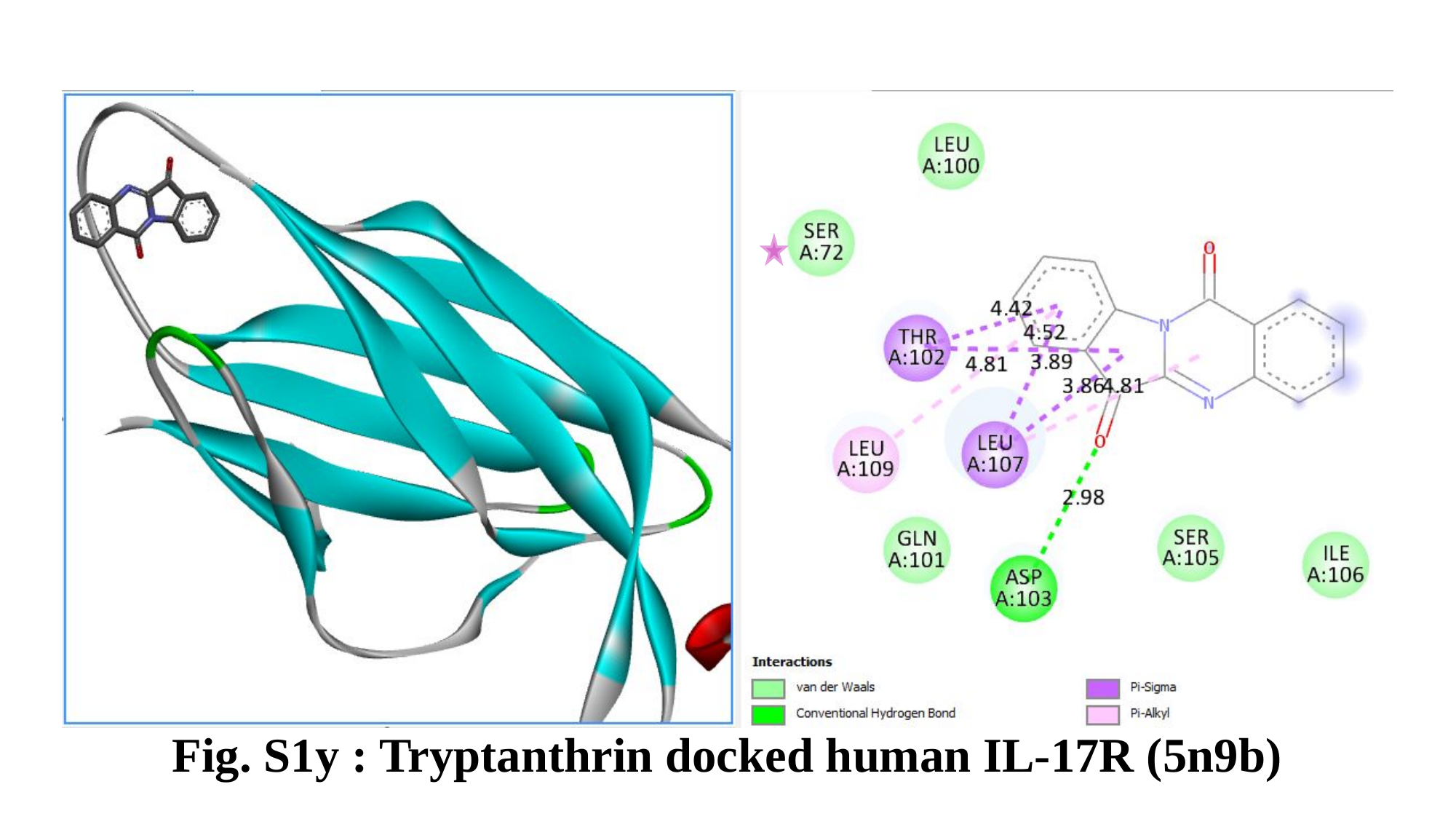

Fig. S1y : Tryptanthrin docked human IL-17R (5n9b)
