# Supplementary material for: Targeting asparagine and cysteine in SARS-CoV-2 variants and human pro-inflammatory mediators to alleviate COVID-19 severity; a cross-section and in-silico study
Source: Sci Rep. 2025 Nov 3;15:38445. doi: 10.1038/s41598-025-19359-y (PMC12583749; doi:10.1038/s41598-025-19359-y)
Supplement: Supplementary file 9 — Supplementary Material 9 [file 41598_2025_19359_MOESM9_ESM.pptx]

## Slide 1
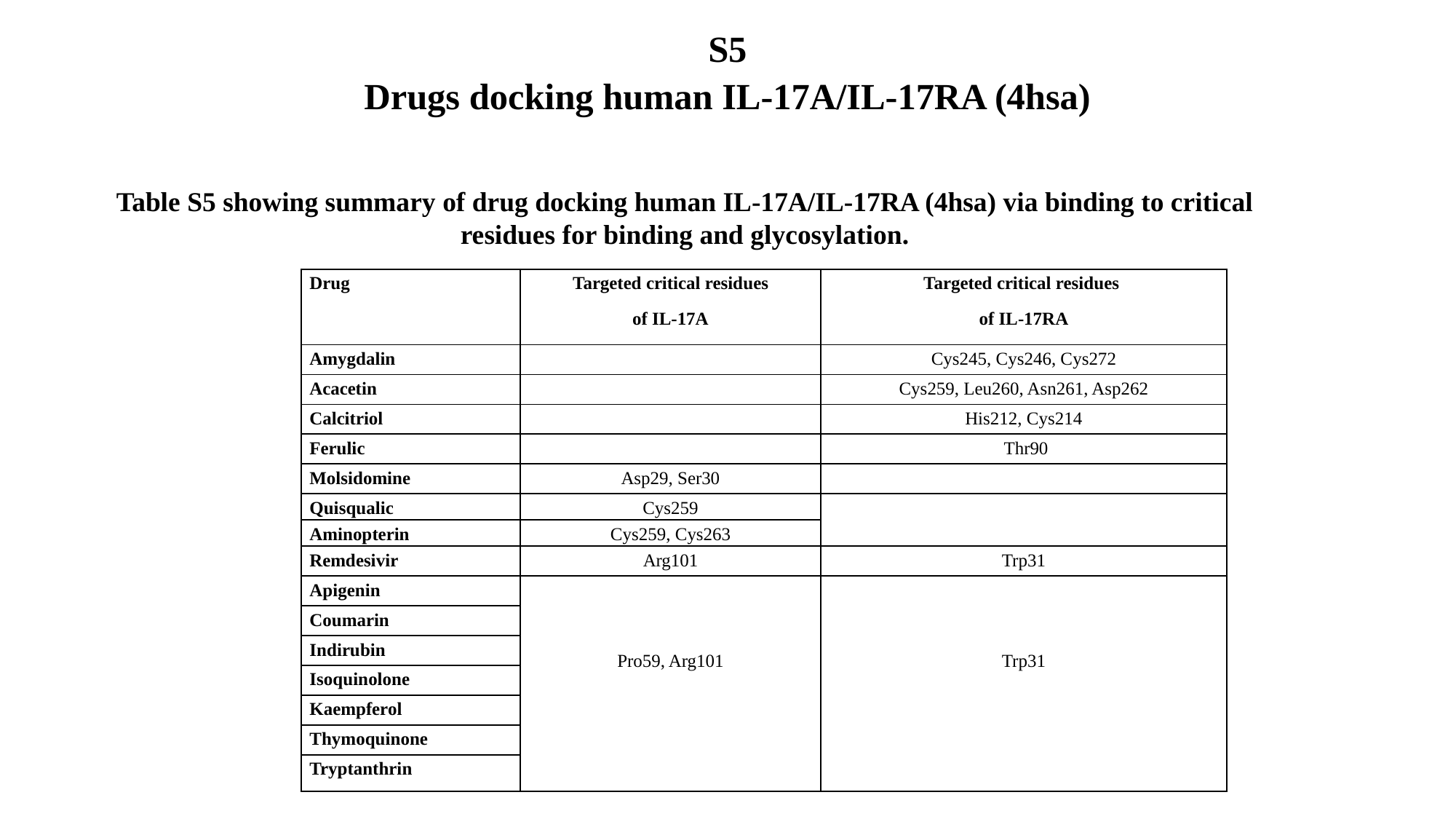

S5
Drugs docking human IL-17A/IL-17RA (4hsa)
Table S5 showing summary of drug docking human IL-17A/IL-17RA (4hsa) via binding to critical residues for binding and glycosylation.
| Drug | Targeted critical residues of IL-17A | Targeted critical residues of IL-17RA |
| --- | --- | --- |
| Amygdalin | | Cys245, Cys246, Cys272 |
| Acacetin | | Cys259, Leu260, Asn261, Asp262 |
| Calcitriol | | His212, Cys214 |
| Ferulic | | Thr90 |
| Molsidomine | Asp29, Ser30 | |
| Quisqualic | Cys259 | |
| Aminopterin | Cys259, Cys263 | |
| Remdesivir | Arg101 | Trp31 |
| Apigenin | Pro59, Arg101 | Trp31 |
| Coumarin | | |
| Indirubin | | |
| Isoquinolone | | |
| Kaempferol | | |
| Thymoquinone | | |
| Tryptanthrin | | |

## Slide 2
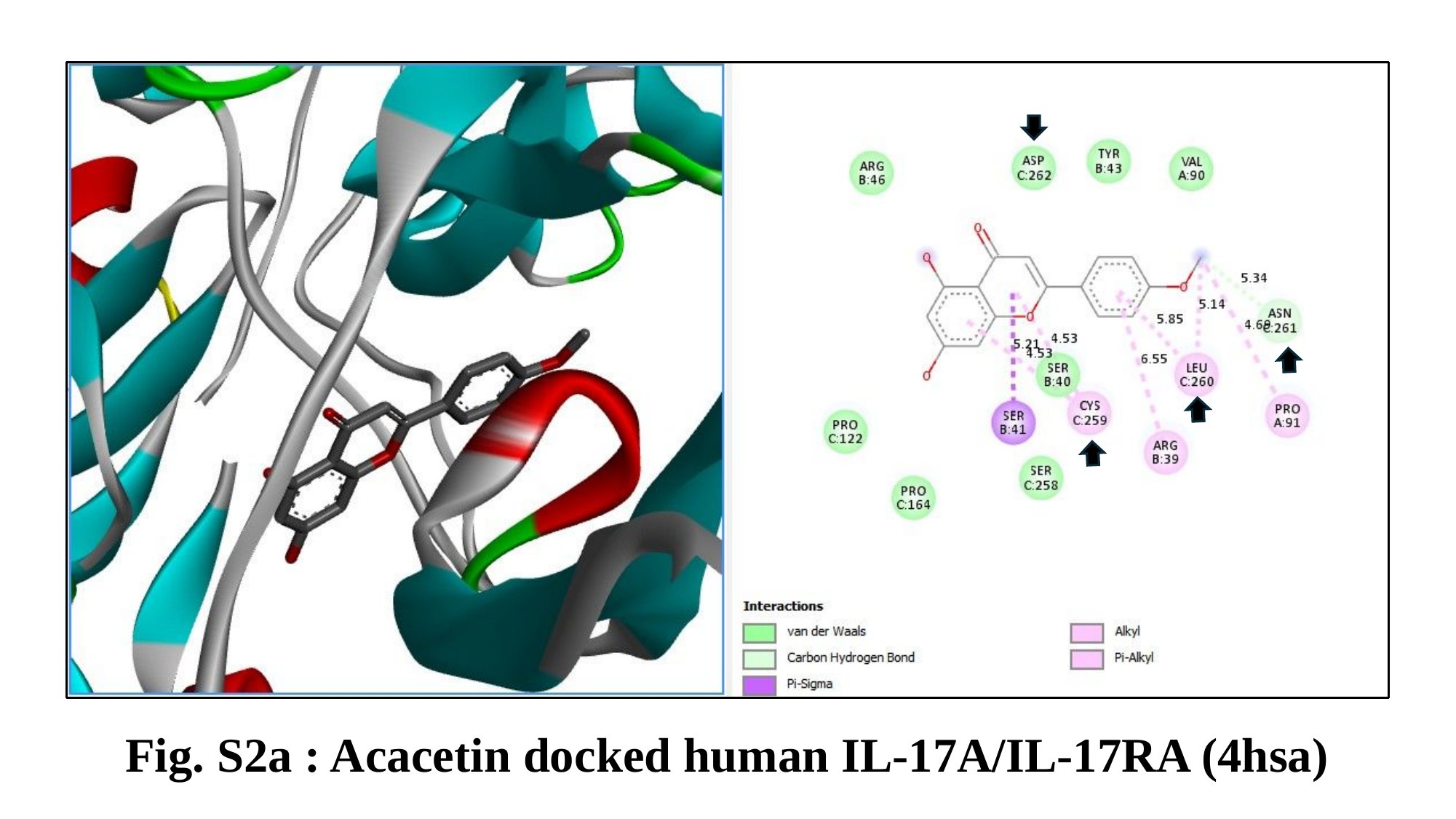

Fig. S2a : Acacetin docked human IL-17A/IL-17RA (4hsa)

## Slide 3
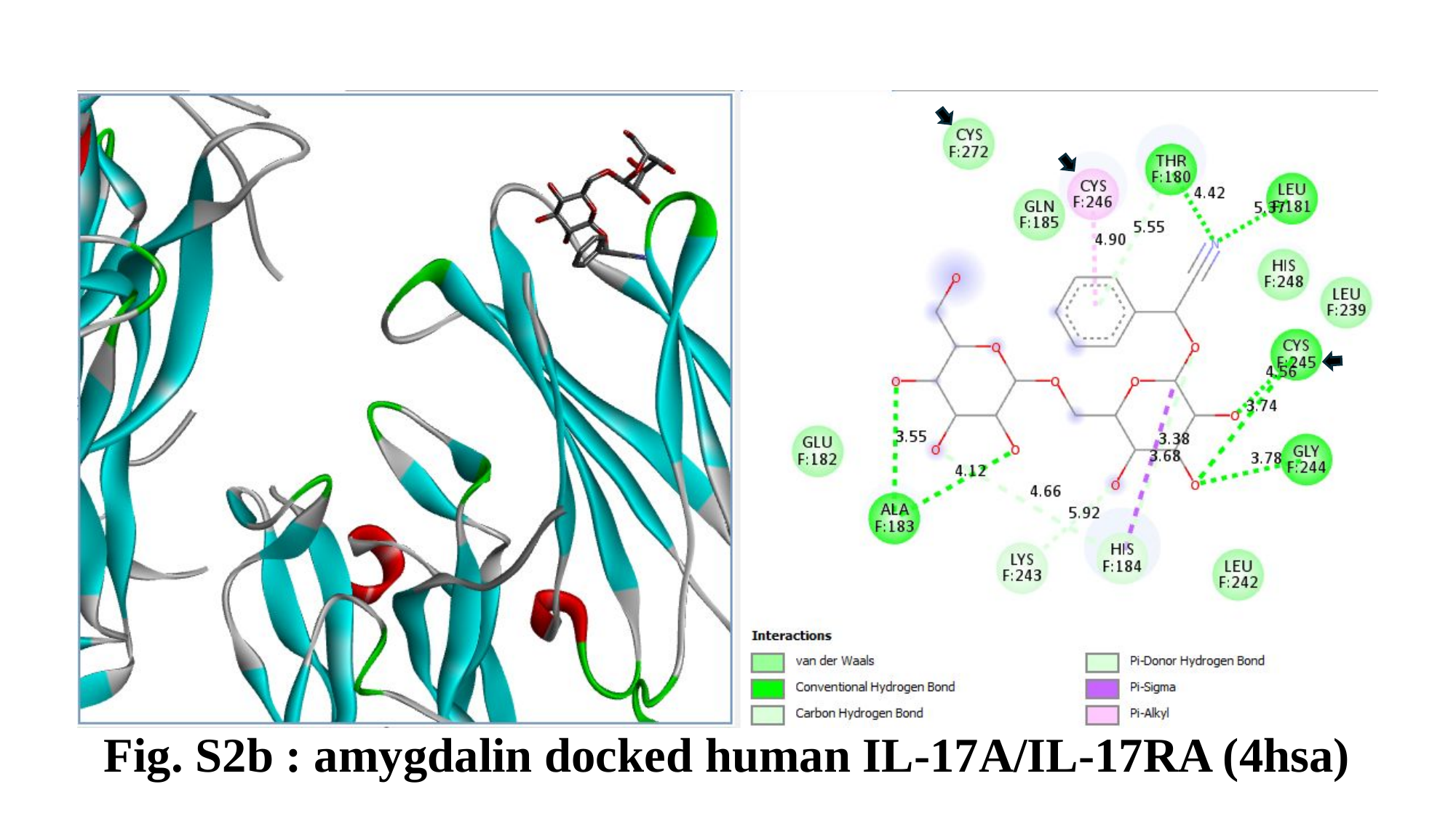

Fig. S2b : amygdalin docked human IL-17A/IL-17RA (4hsa)

## Slide 4
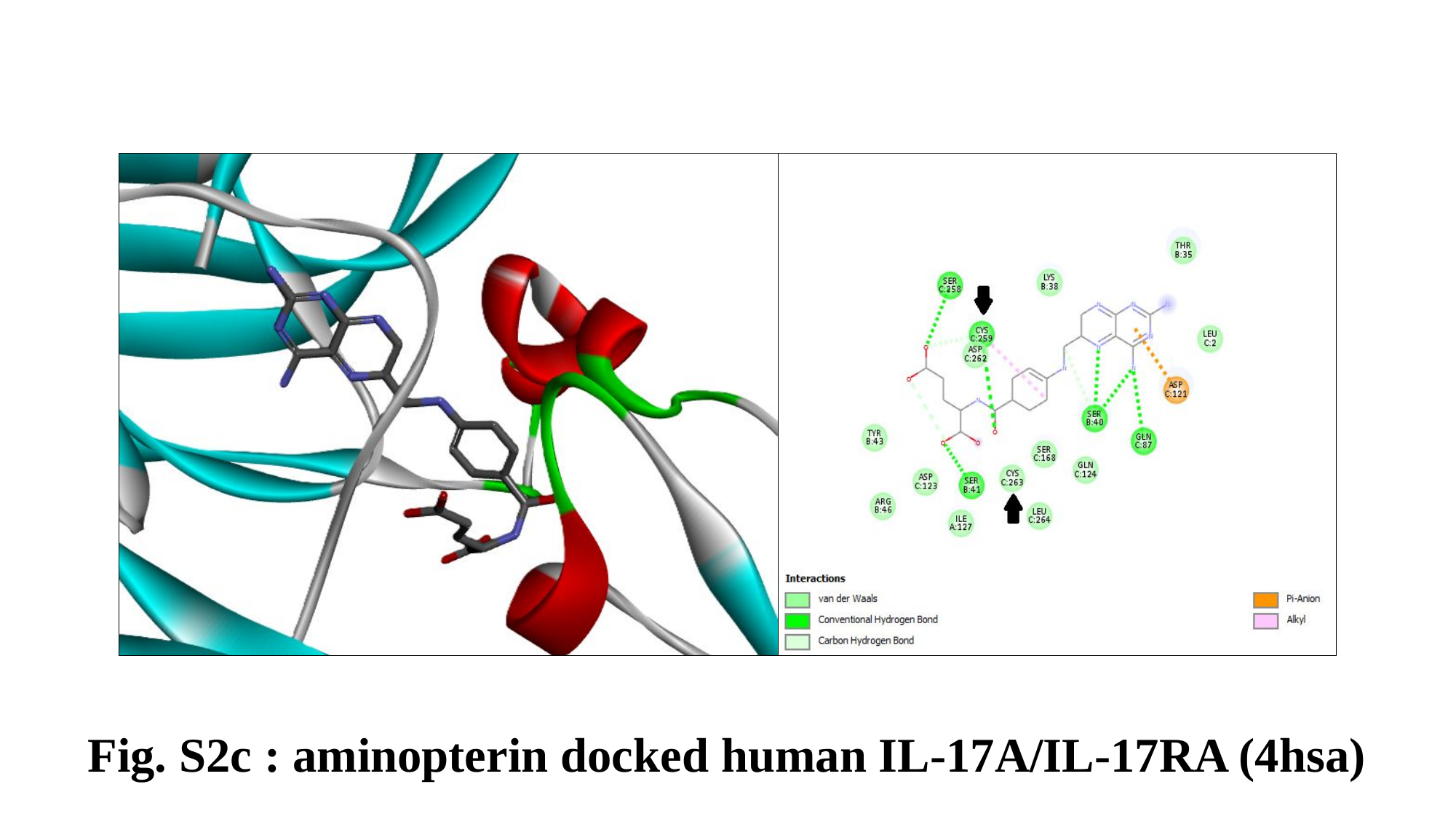

Fig. S2c : aminopterin docked human IL-17A/IL-17RA (4hsa)

## Slide 5
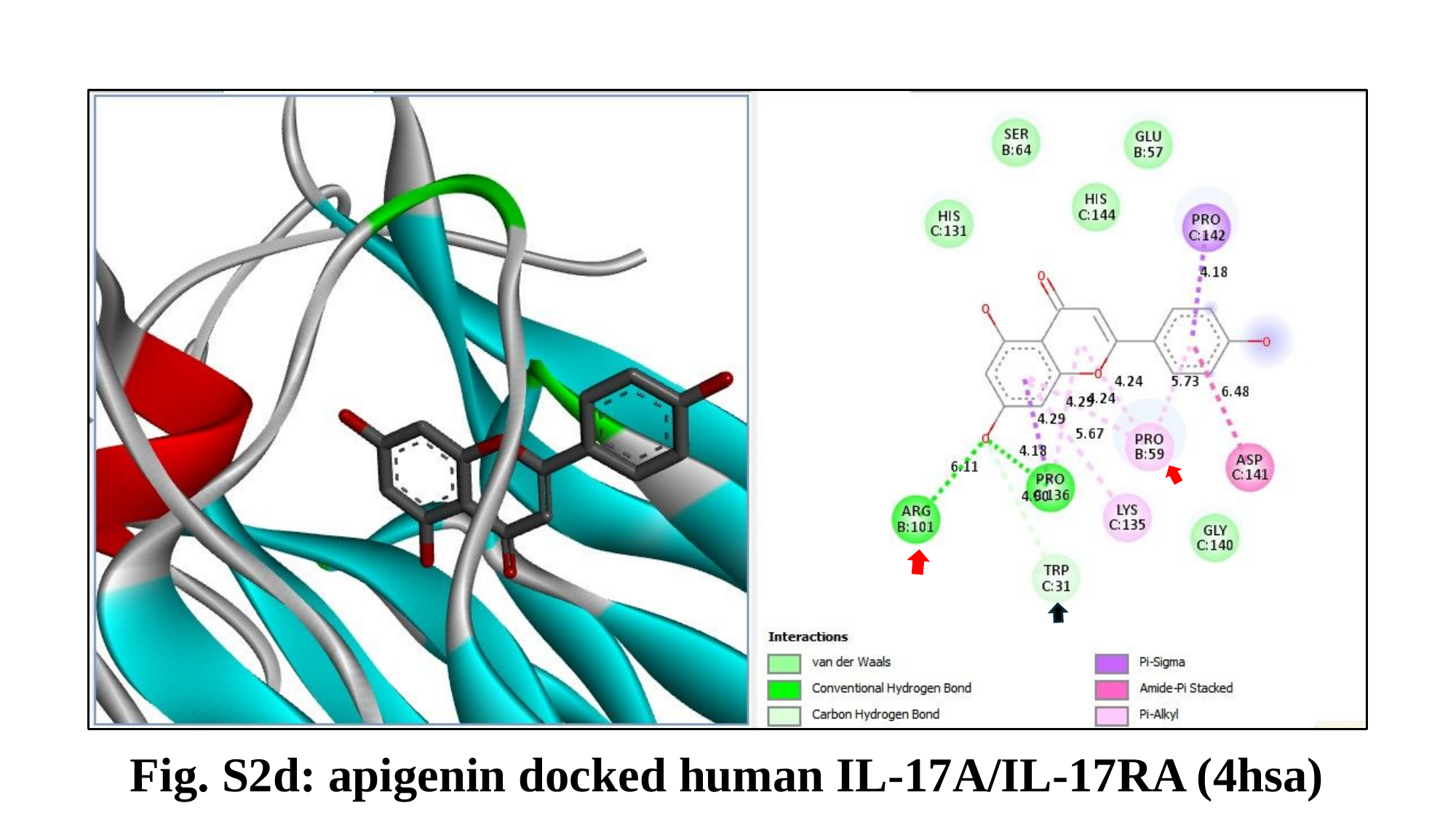

Fig. S2d: apigenin docked human IL-17A/IL-17RA (4hsa)

## Slide 6
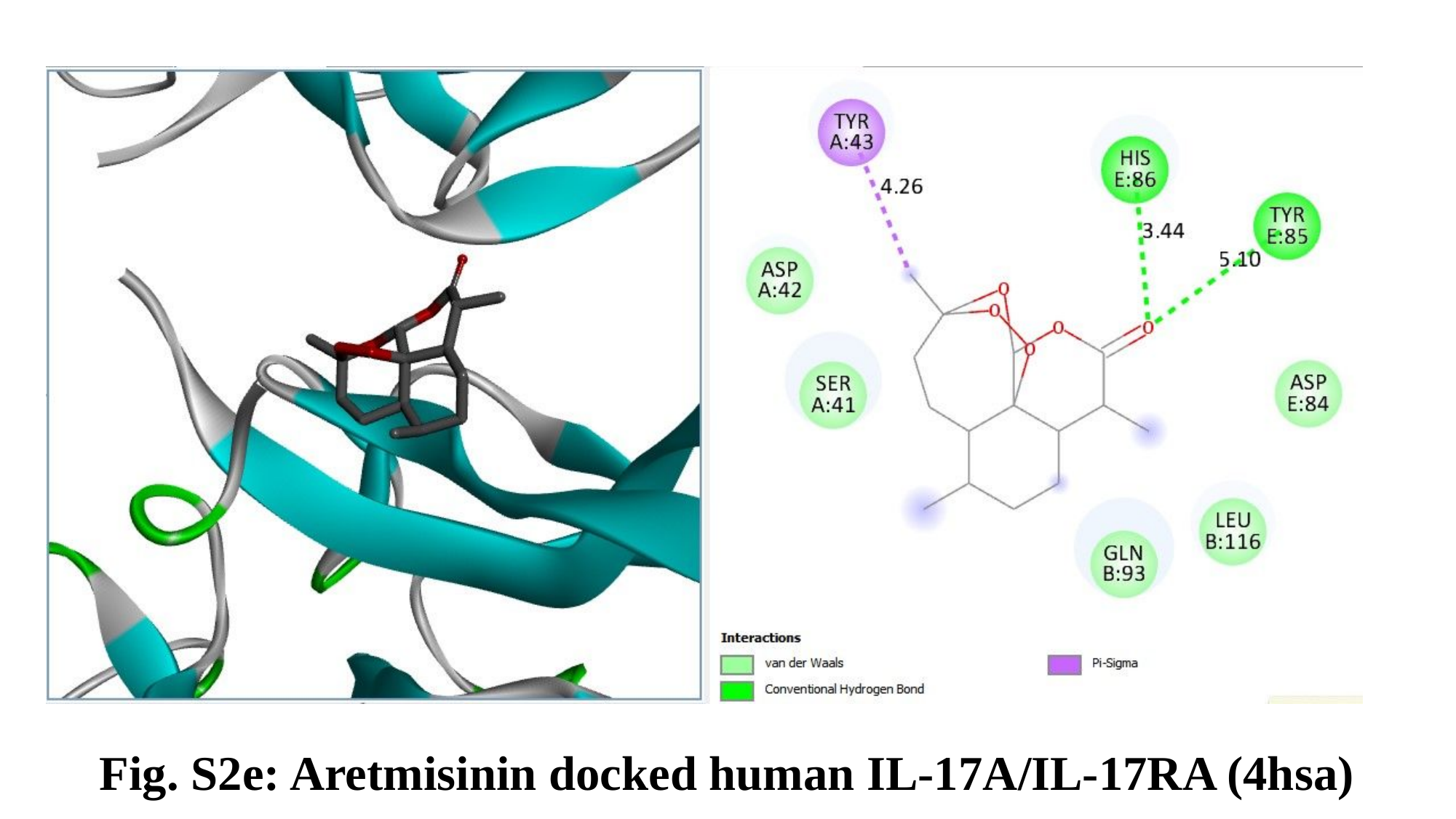

Fig. S2e: Aretmisinin docked human IL-17A/IL-17RA (4hsa)

## Slide 7
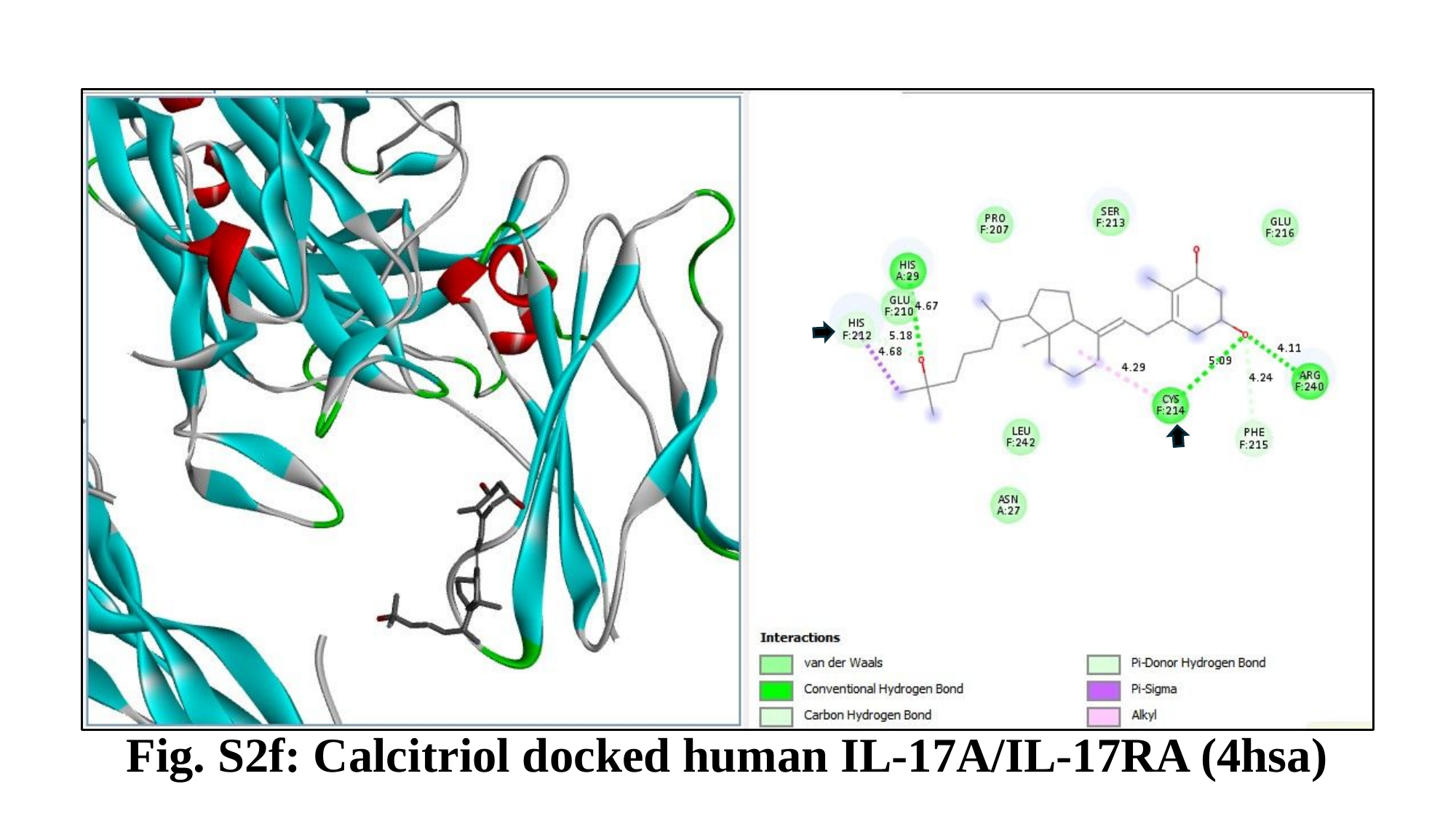

Fig. S2f: Calcitriol docked human IL-17A/IL-17RA (4hsa)

## Slide 8
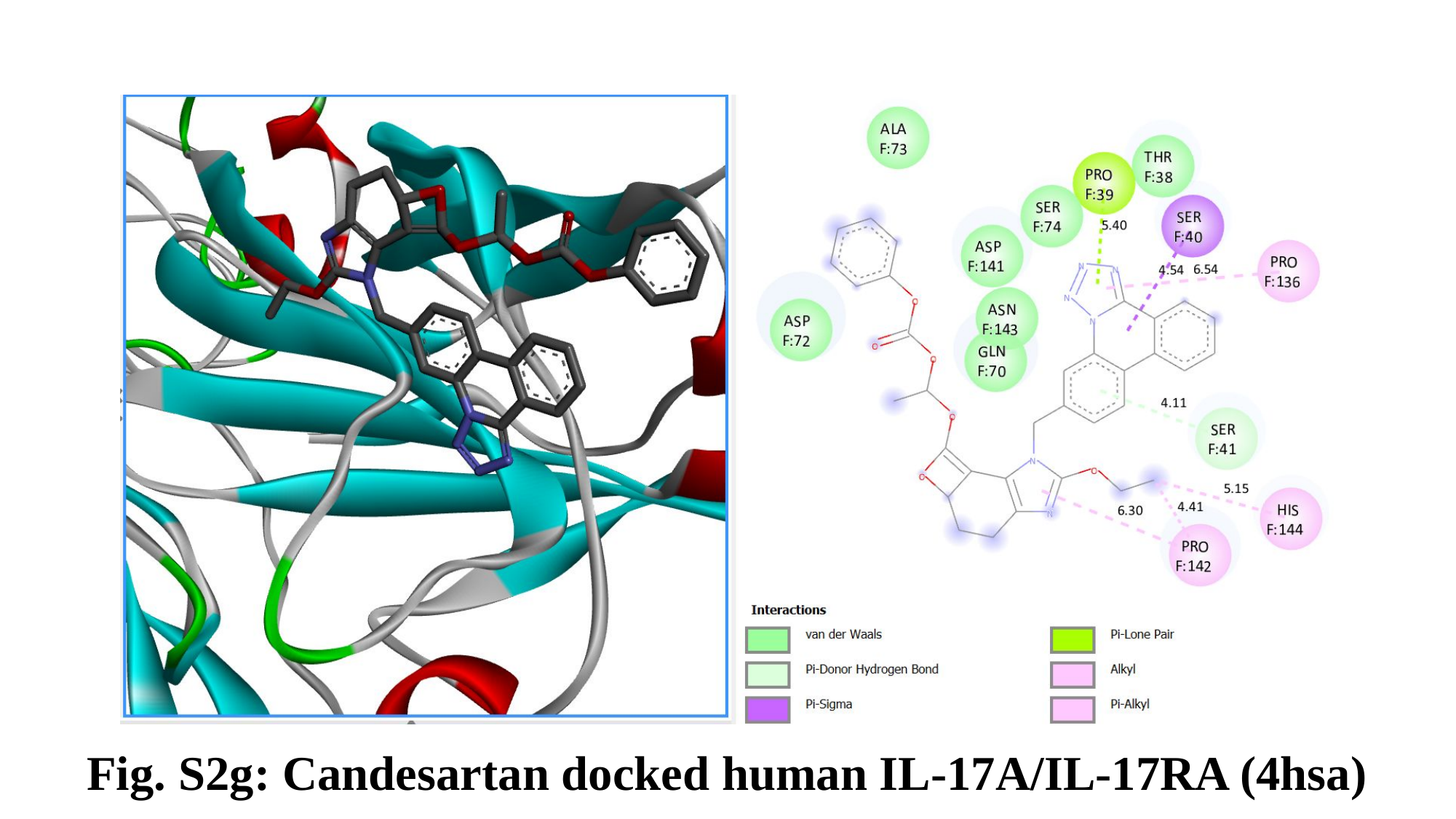

Fig. S2g: Candesartan docked human IL-17A/IL-17RA (4hsa)

## Slide 9
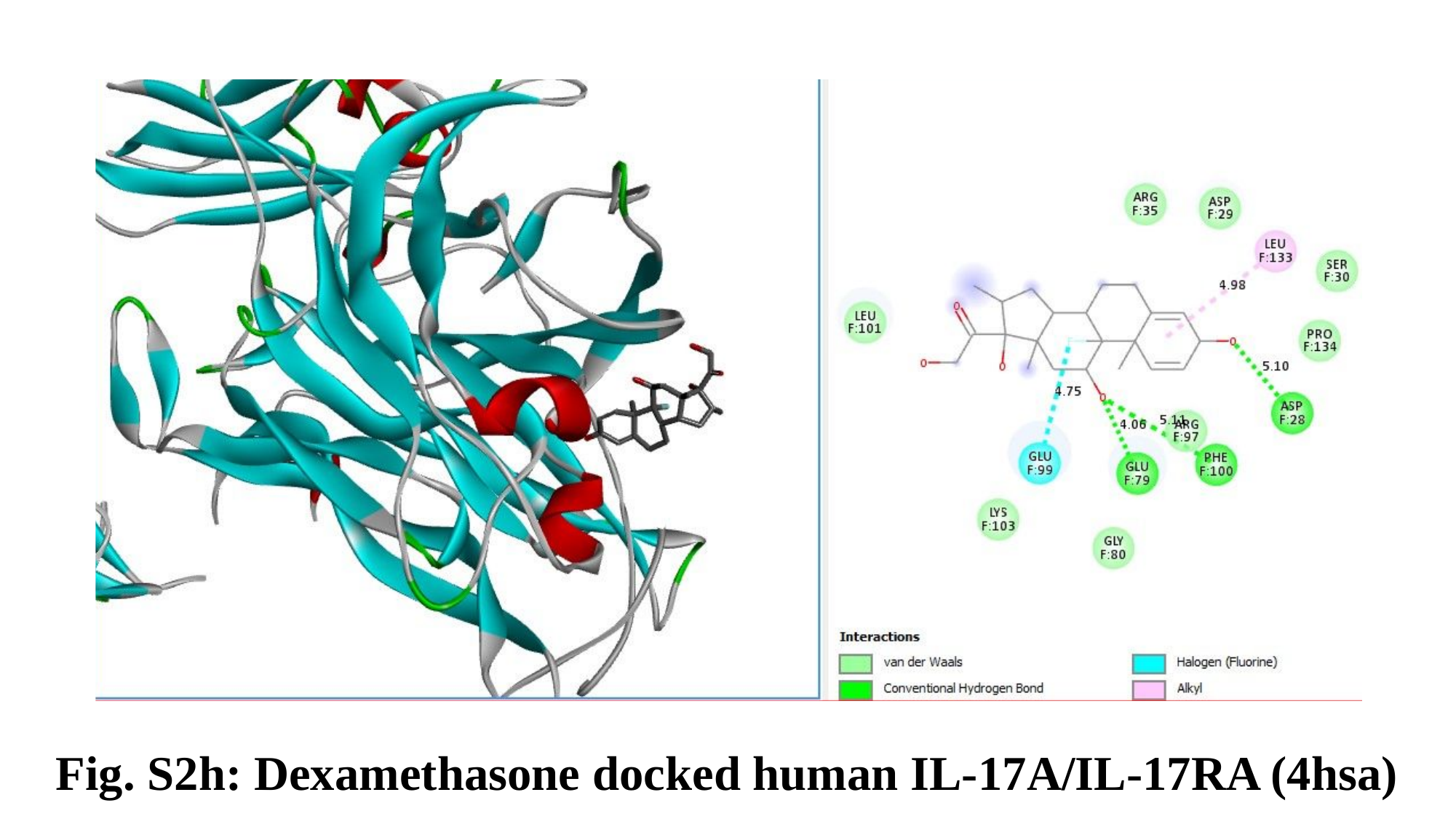

Fig. S2h: Dexamethasone docked human IL-17A/IL-17RA (4hsa)

## Slide 10
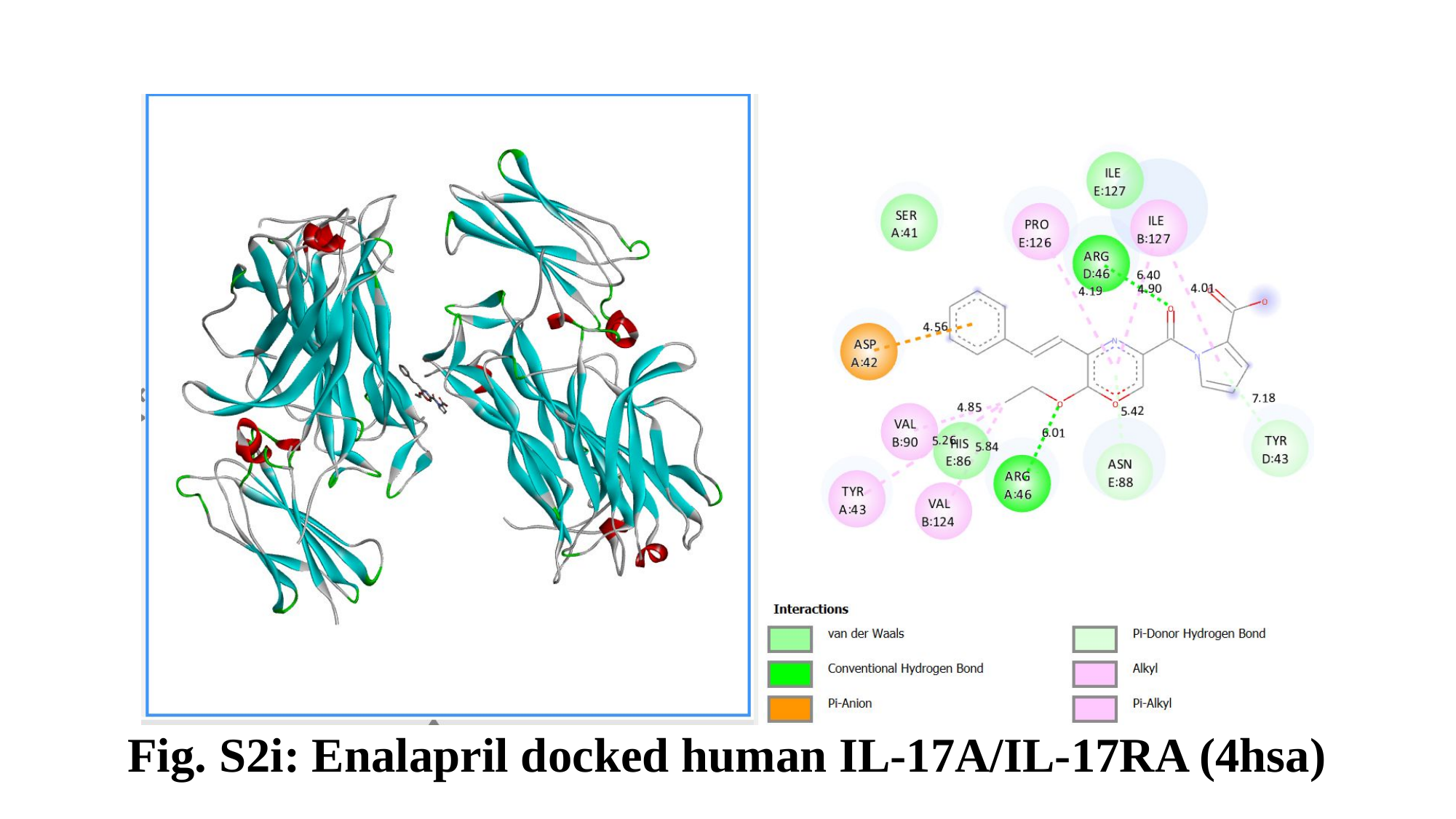

Fig. S2i: Enalapril docked human IL-17A/IL-17RA (4hsa)

## Slide 11
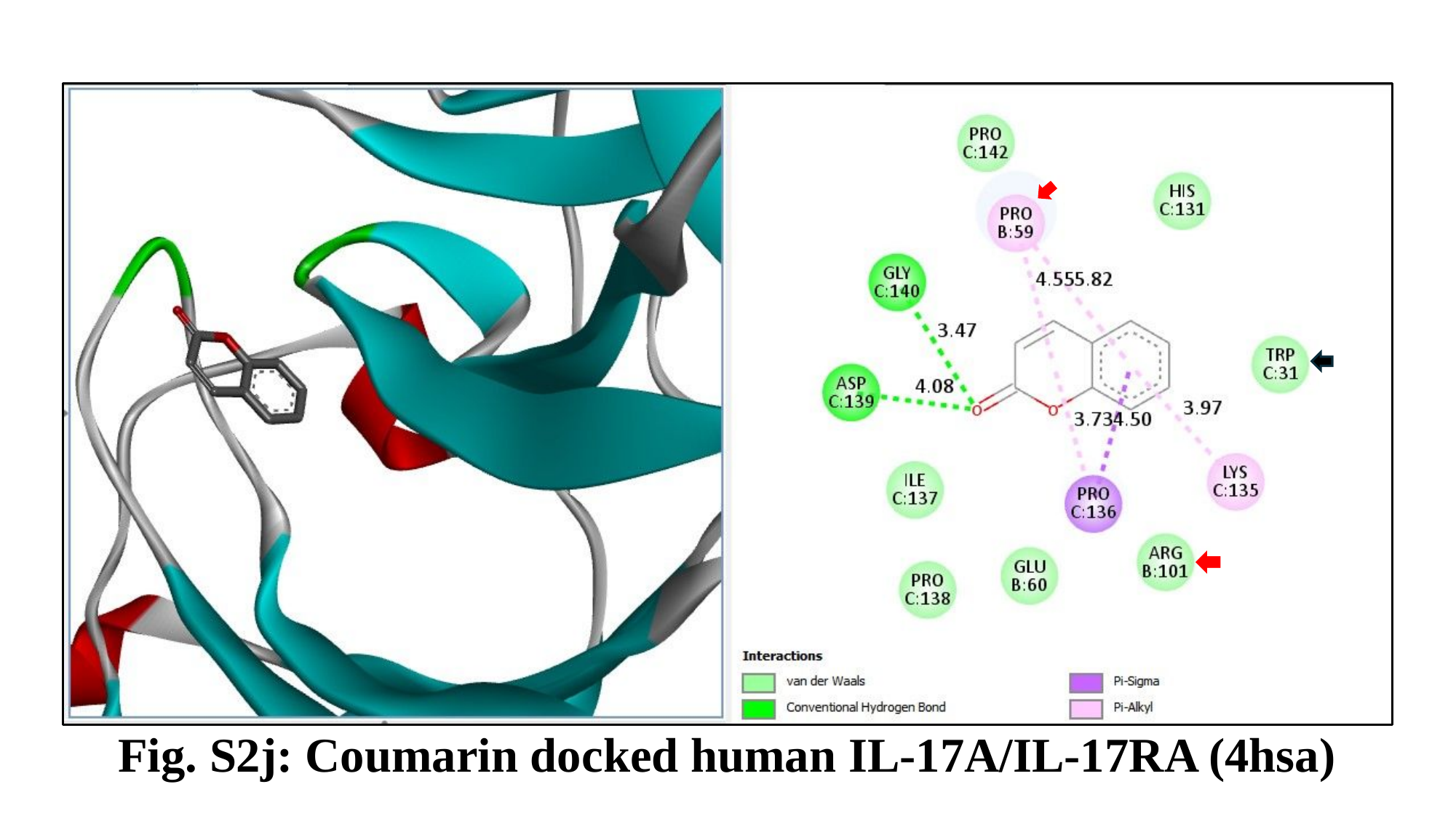

Fig. S2j: Coumarin docked human IL-17A/IL-17RA (4hsa)

## Slide 12
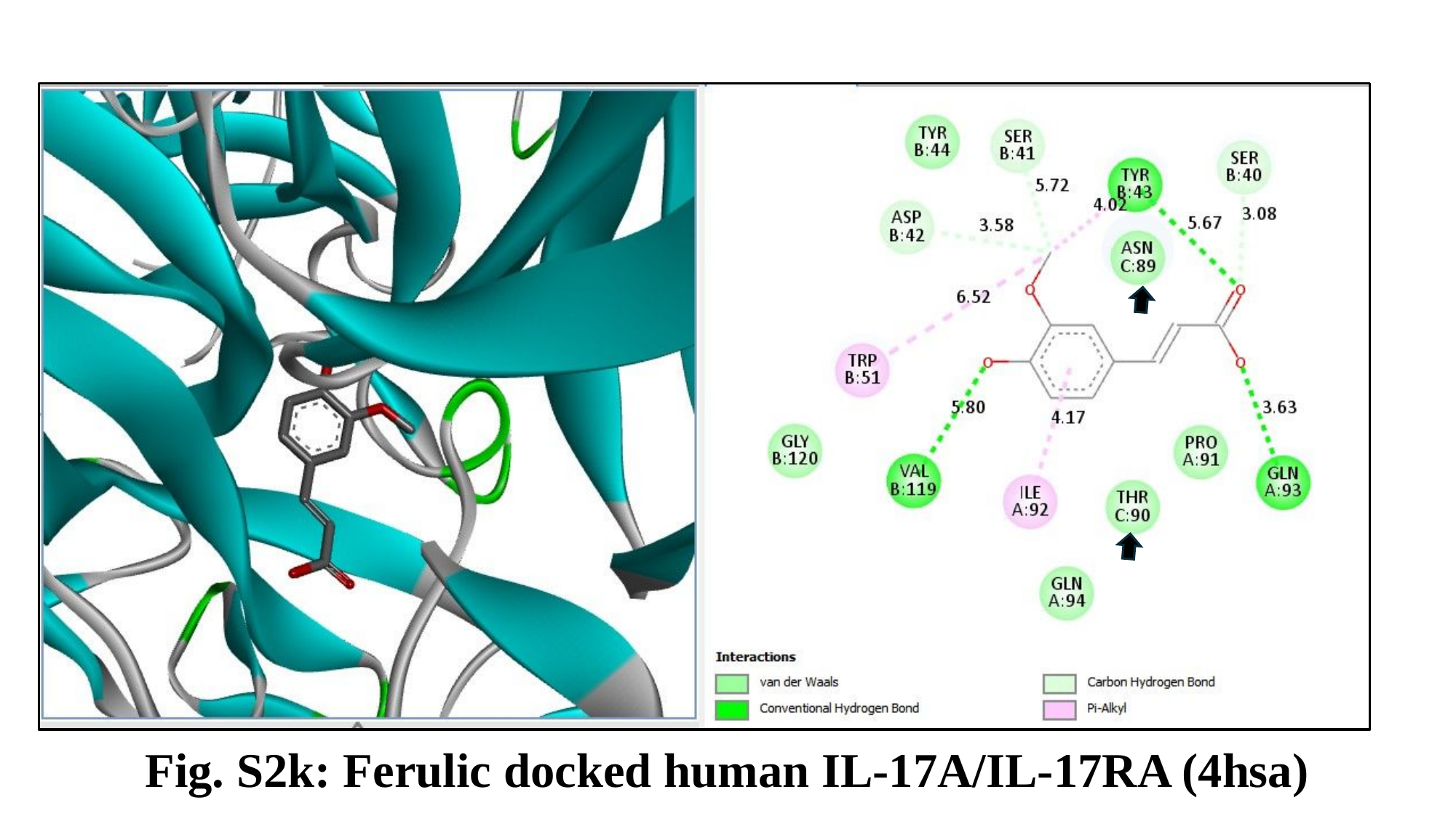

Fig. S2k: Ferulic docked human IL-17A/IL-17RA (4hsa)

## Slide 13
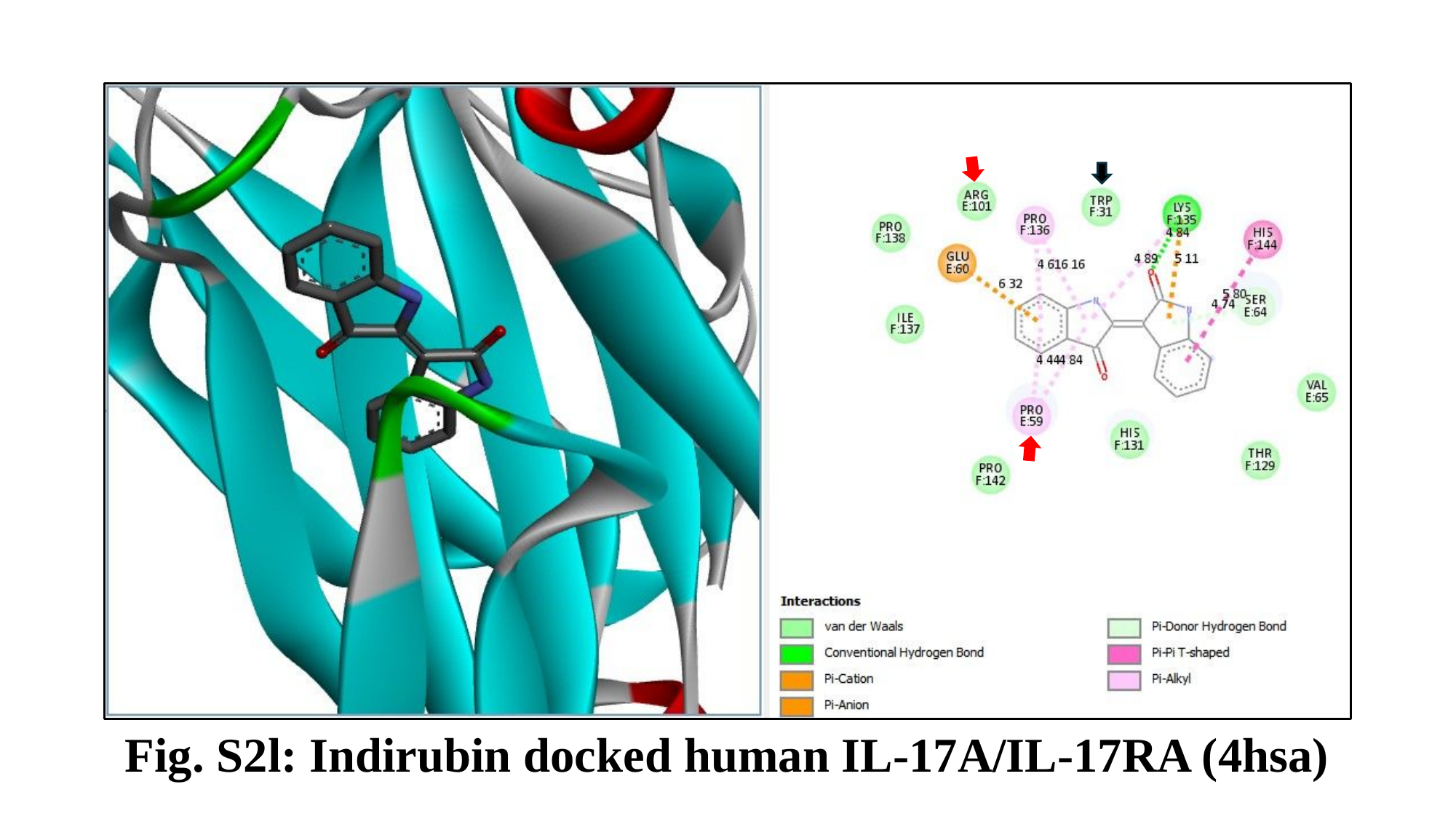

Fig. S2l: Indirubin docked human IL-17A/IL-17RA (4hsa)

## Slide 14
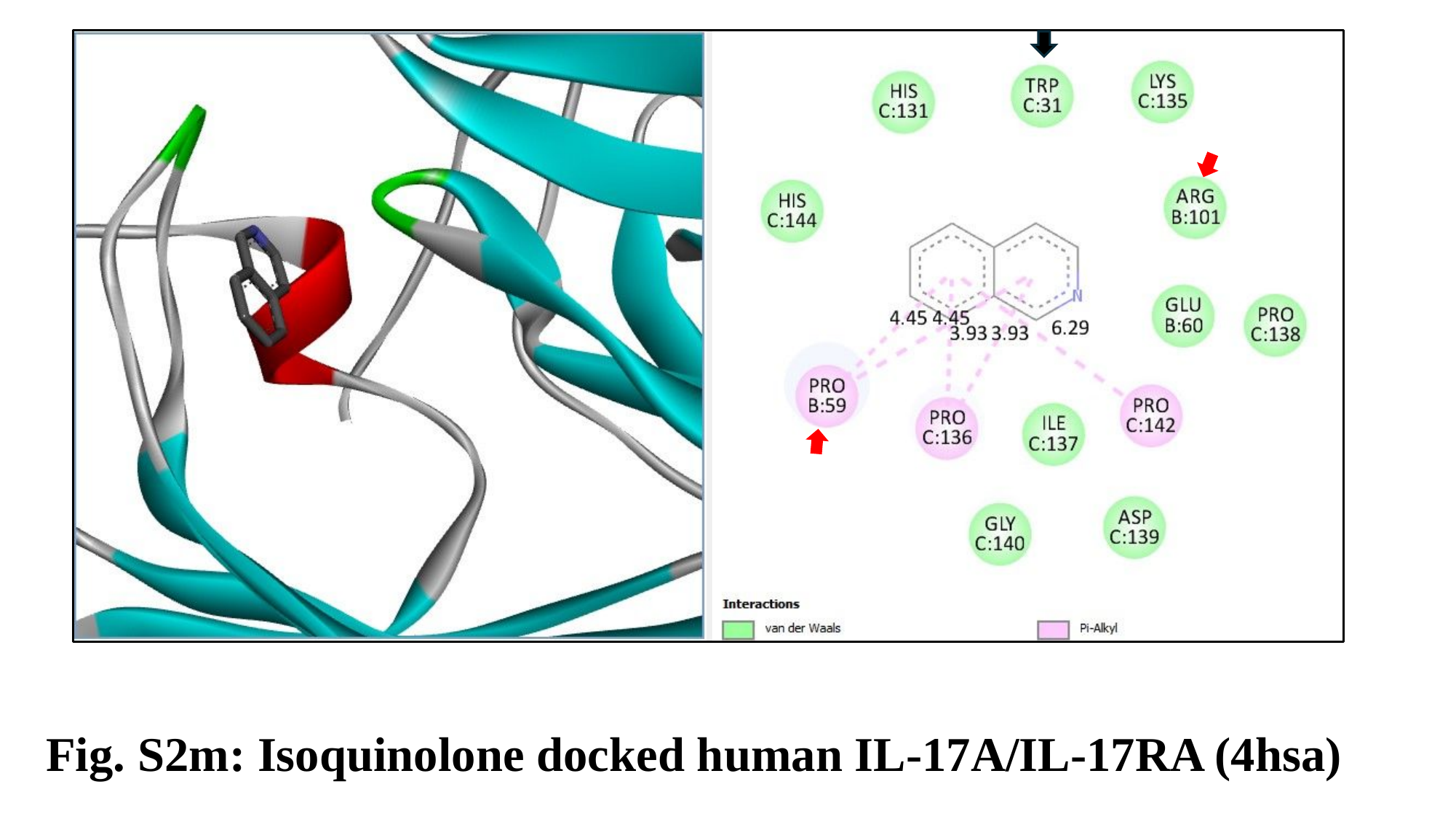

Fig. S2m: Isoquinolone docked human IL-17A/IL-17RA (4hsa)

## Slide 15
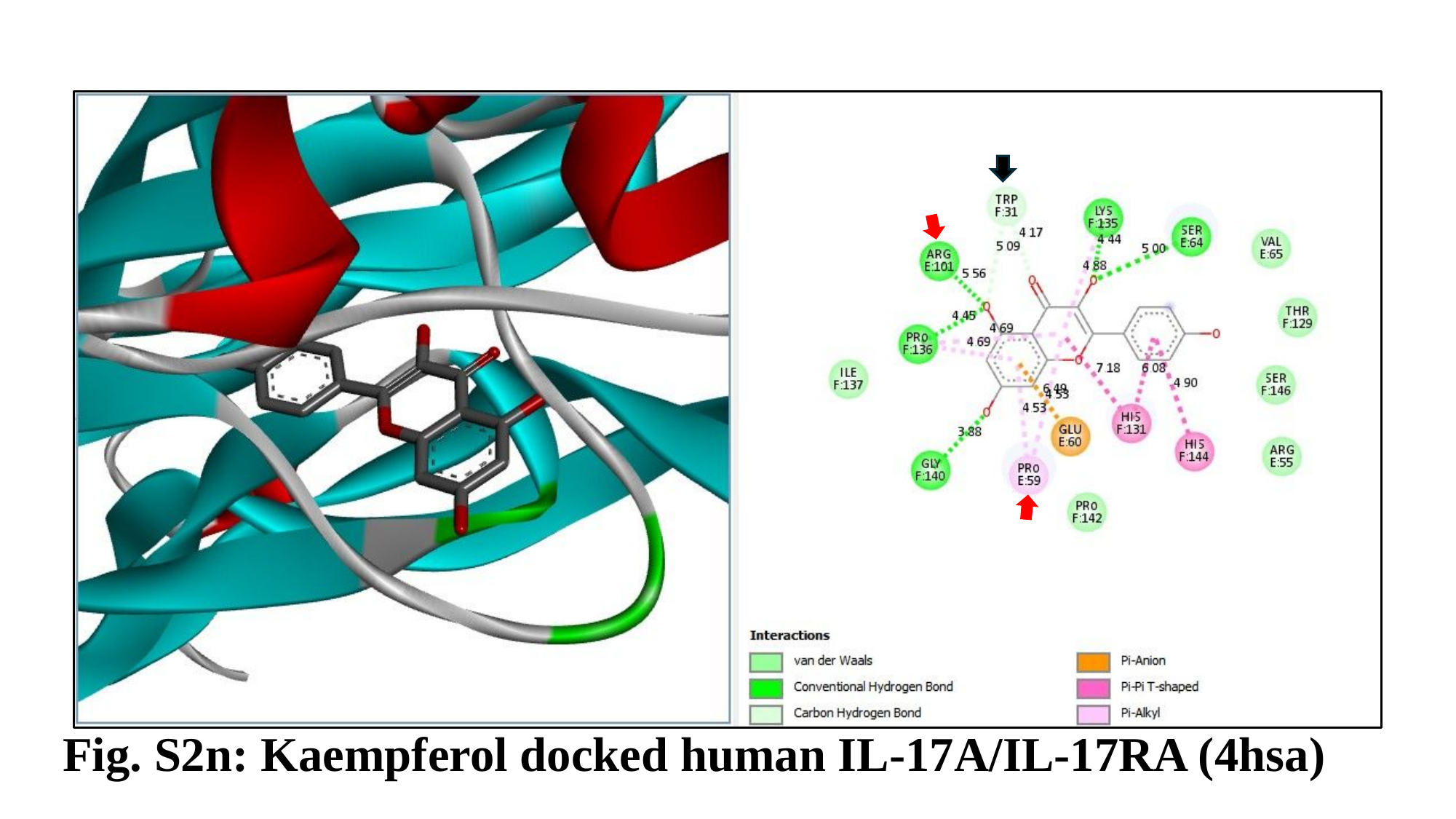

Fig. S2n: Kaempferol docked human IL-17A/IL-17RA (4hsa)

## Slide 16
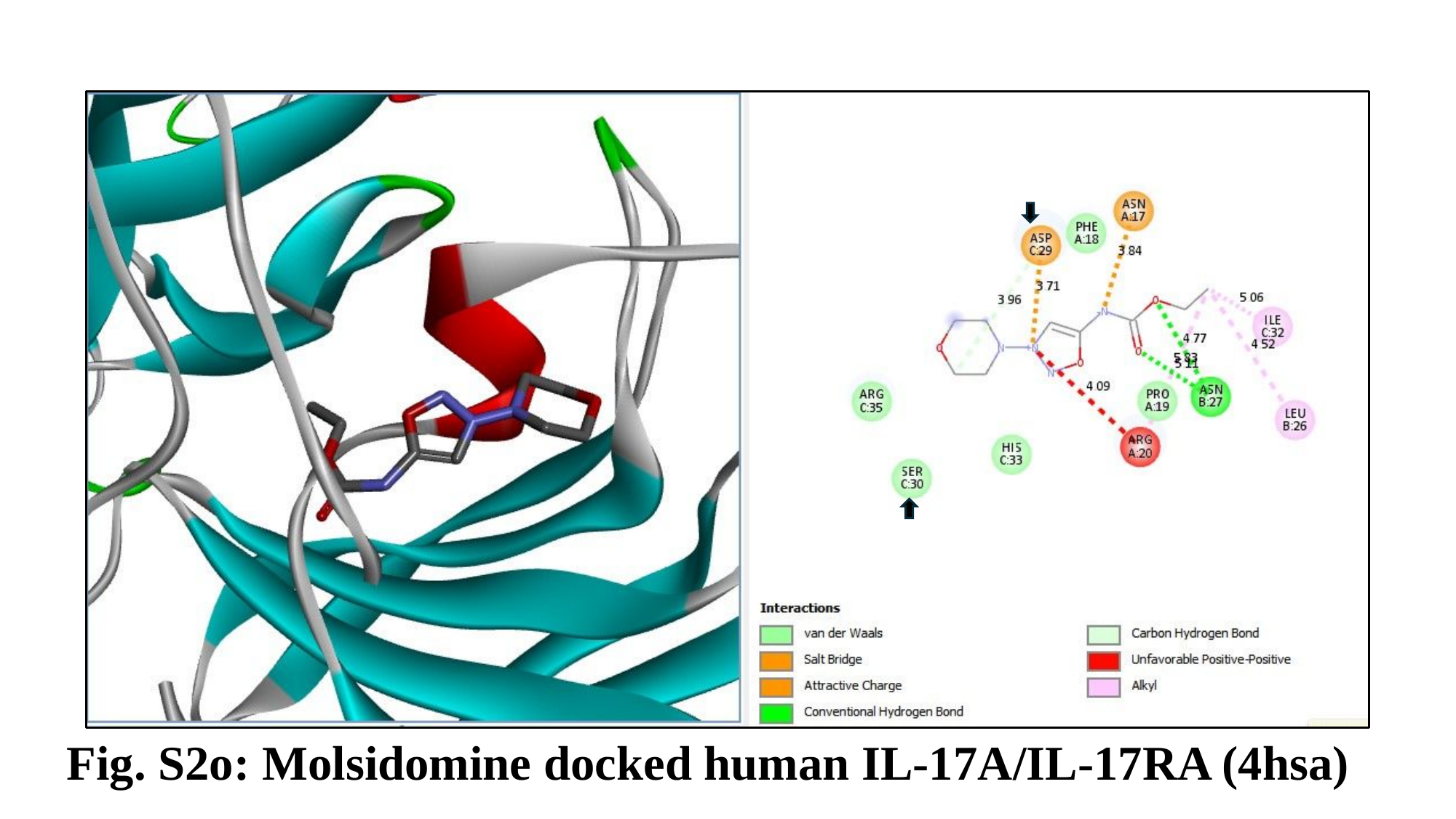

Fig. S2o: Molsidomine docked human IL-17A/IL-17RA (4hsa)

## Slide 17
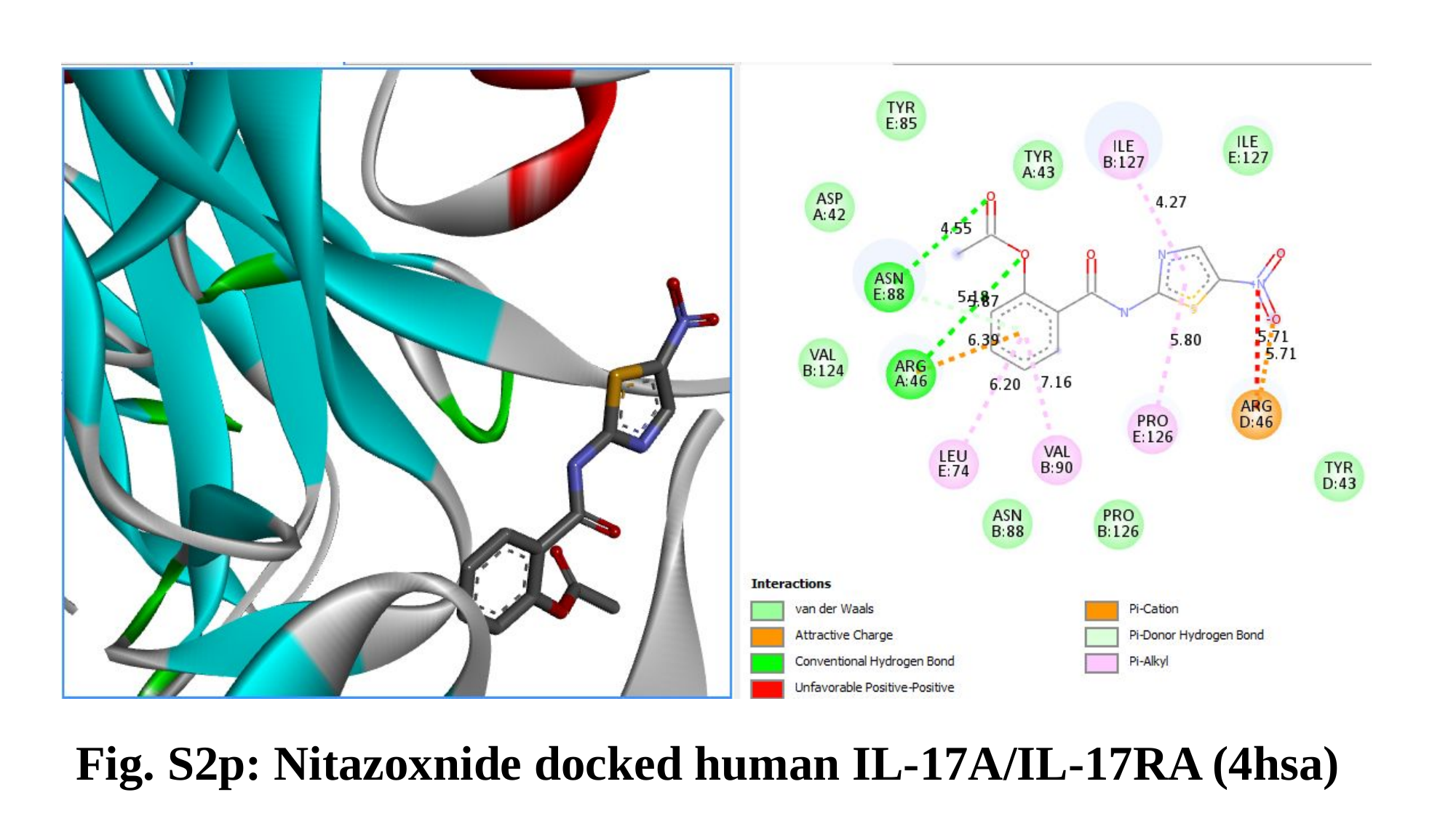

Fig. S2p: Nitazoxnide docked human IL-17A/IL-17RA (4hsa)

## Slide 18
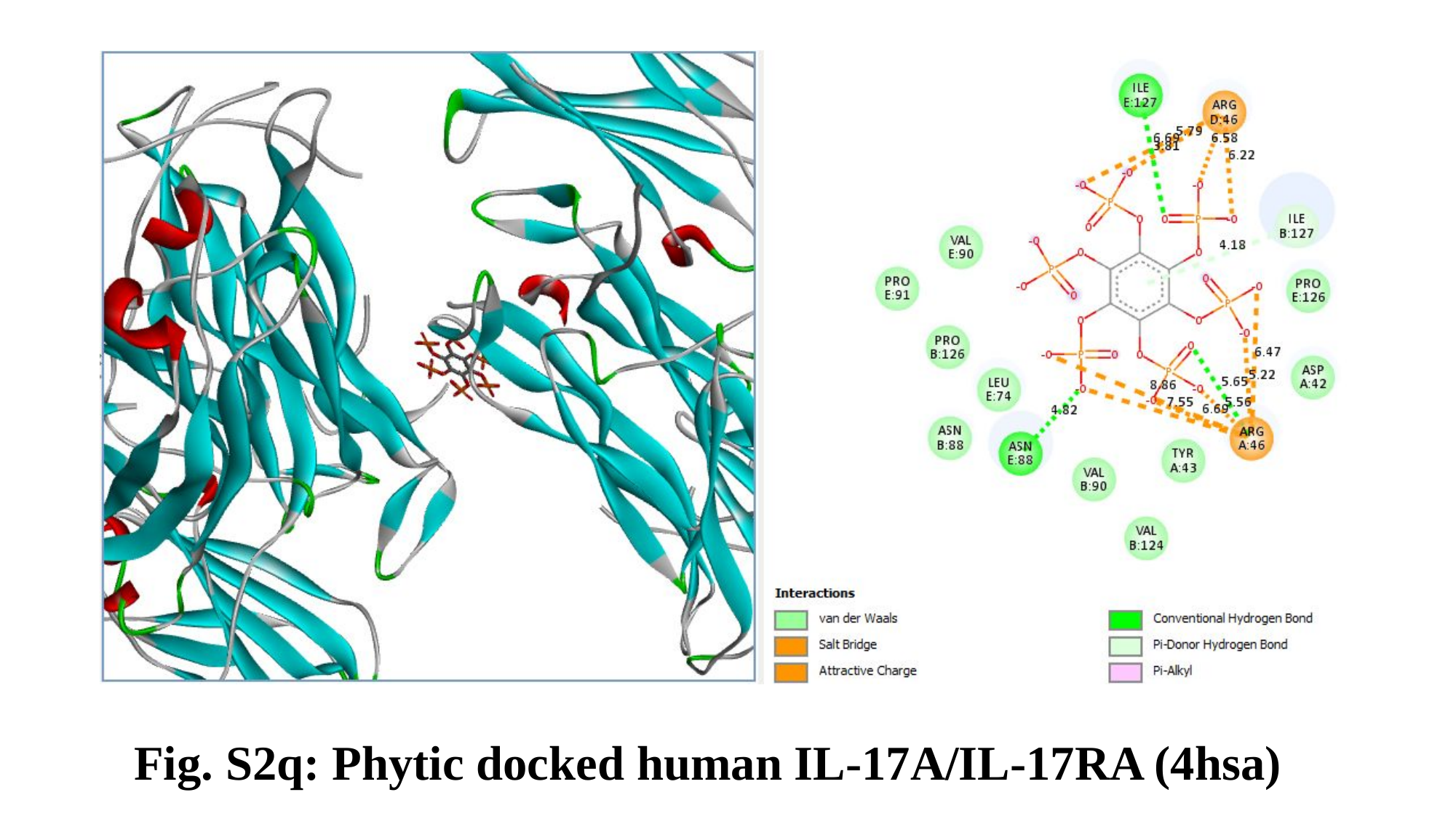

Fig. S2q: Phytic docked human IL-17A/IL-17RA (4hsa)

## Slide 19
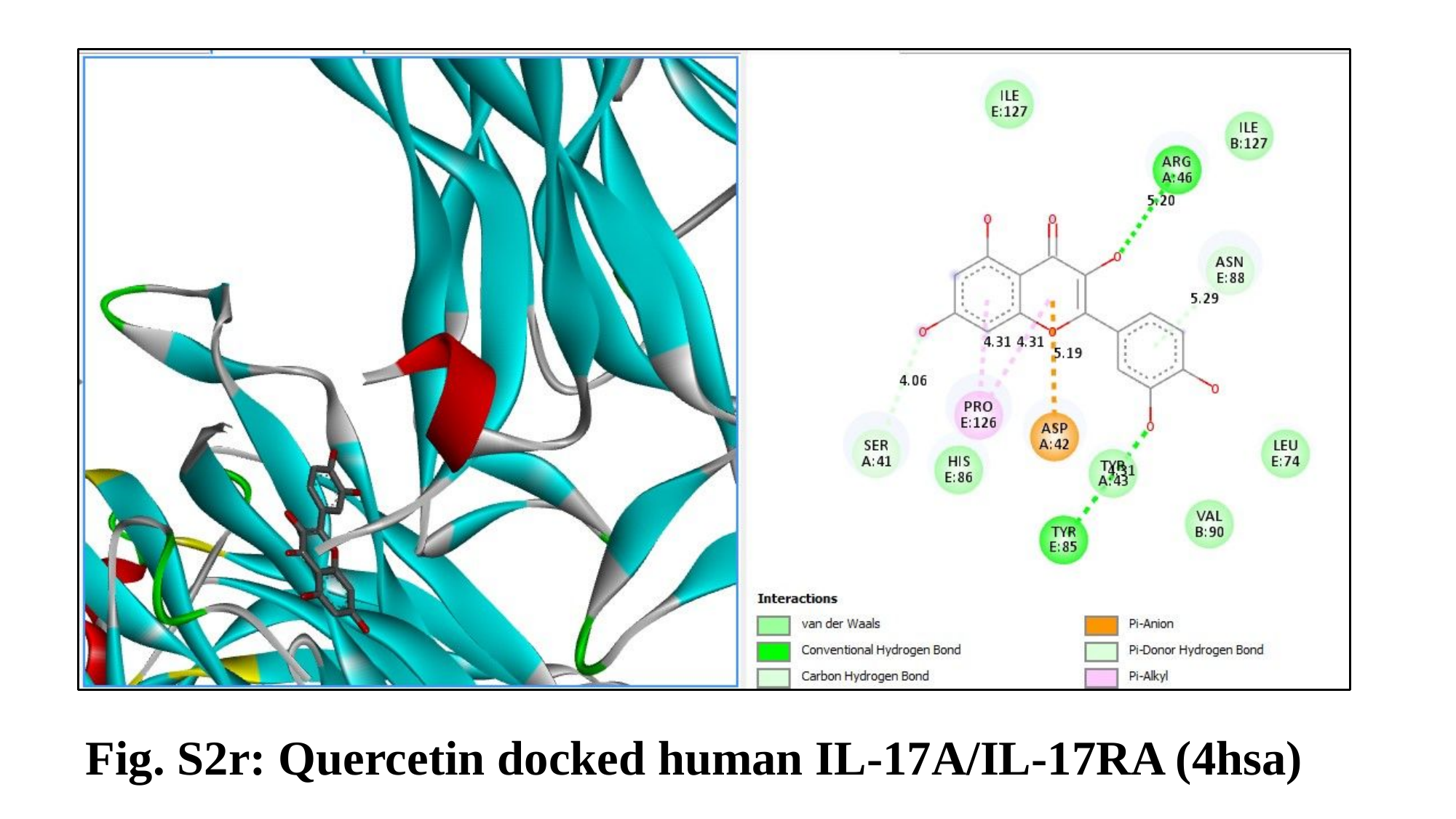

Fig. S2r: Quercetin docked human IL-17A/IL-17RA (4hsa)

## Slide 20
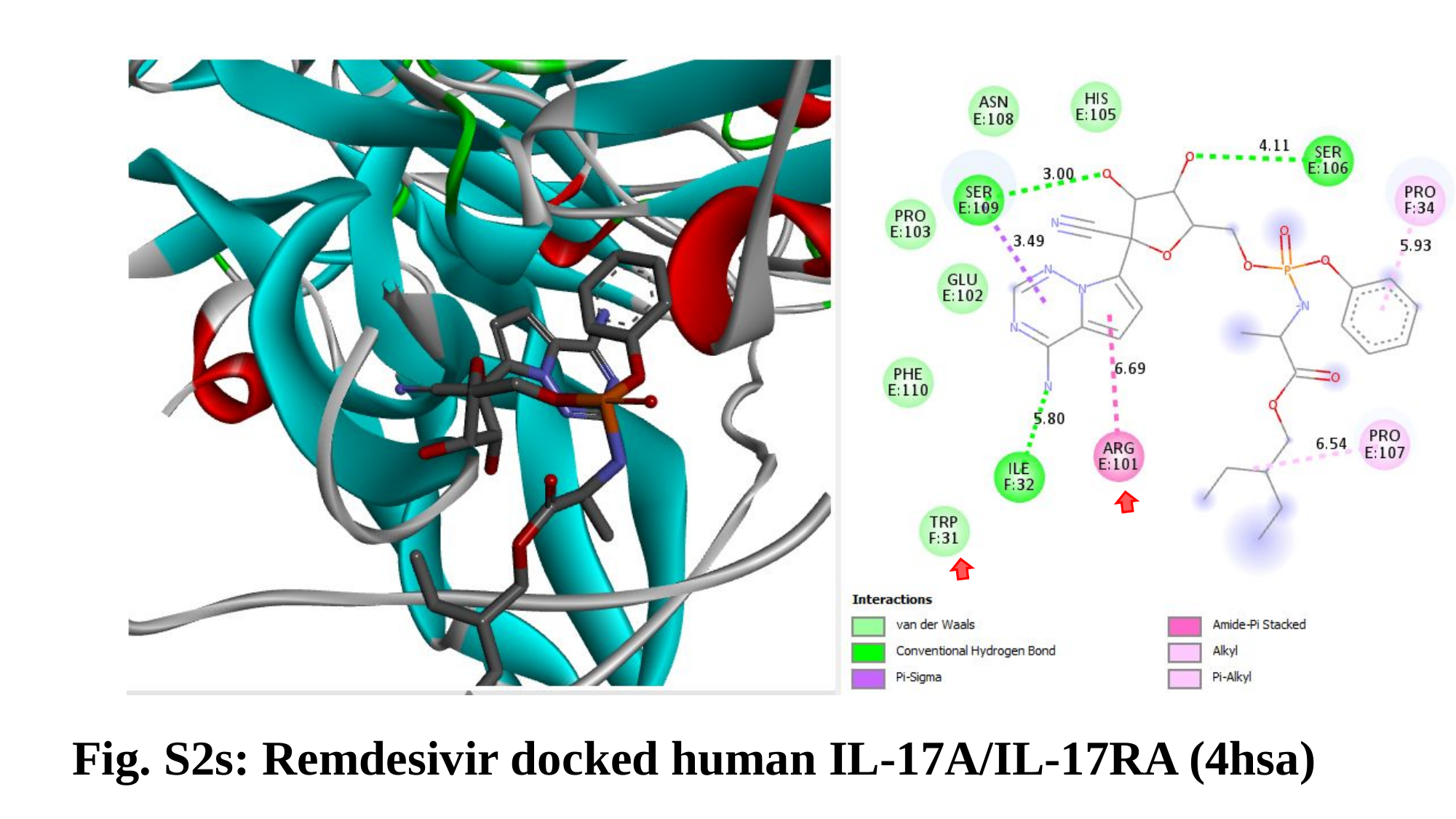

Fig. S2s: Remdesivir docked human IL-17A/IL-17RA (4hsa)

## Slide 21
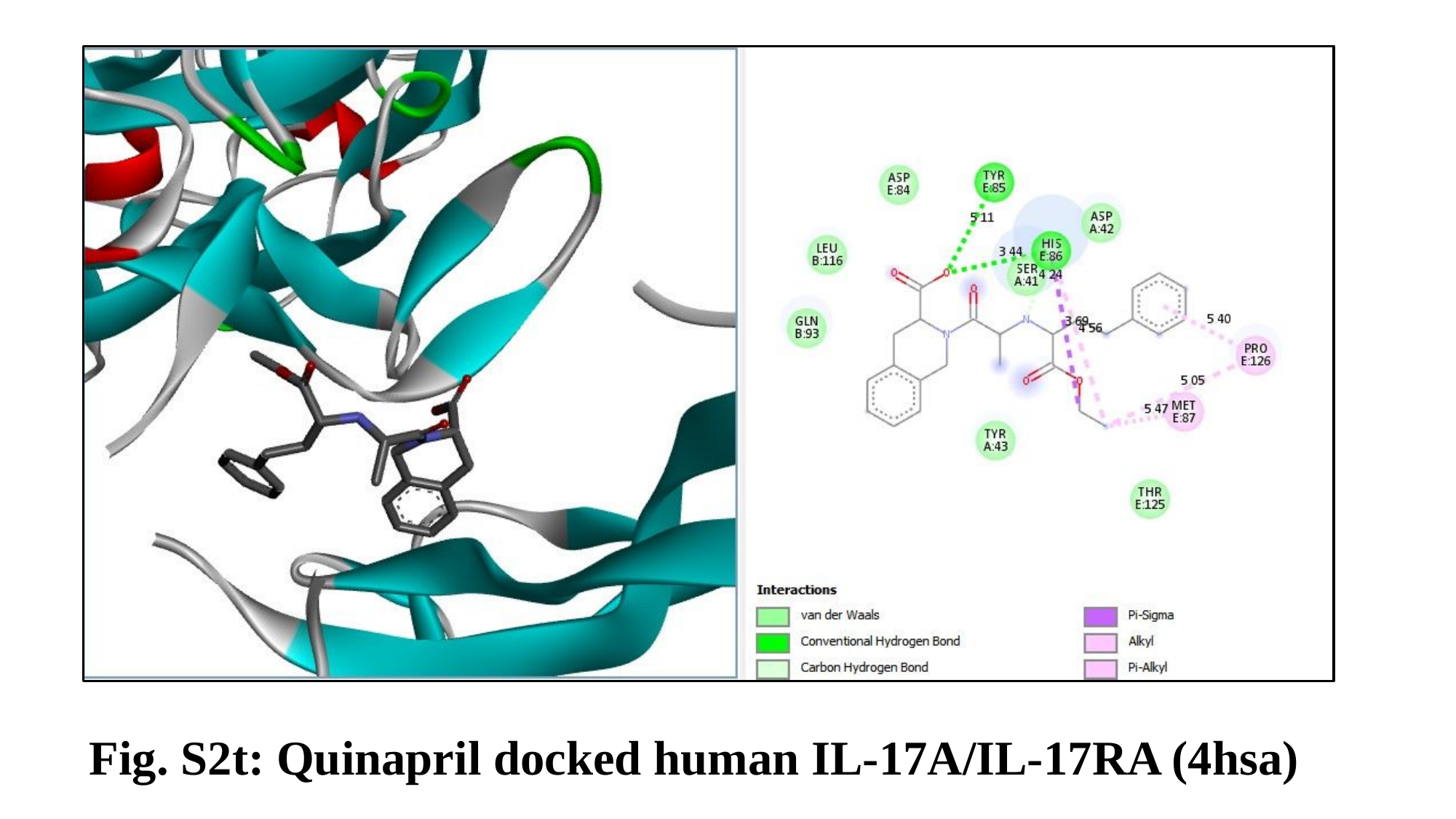

Fig. S2t: Quinapril docked human IL-17A/IL-17RA (4hsa)

## Slide 22
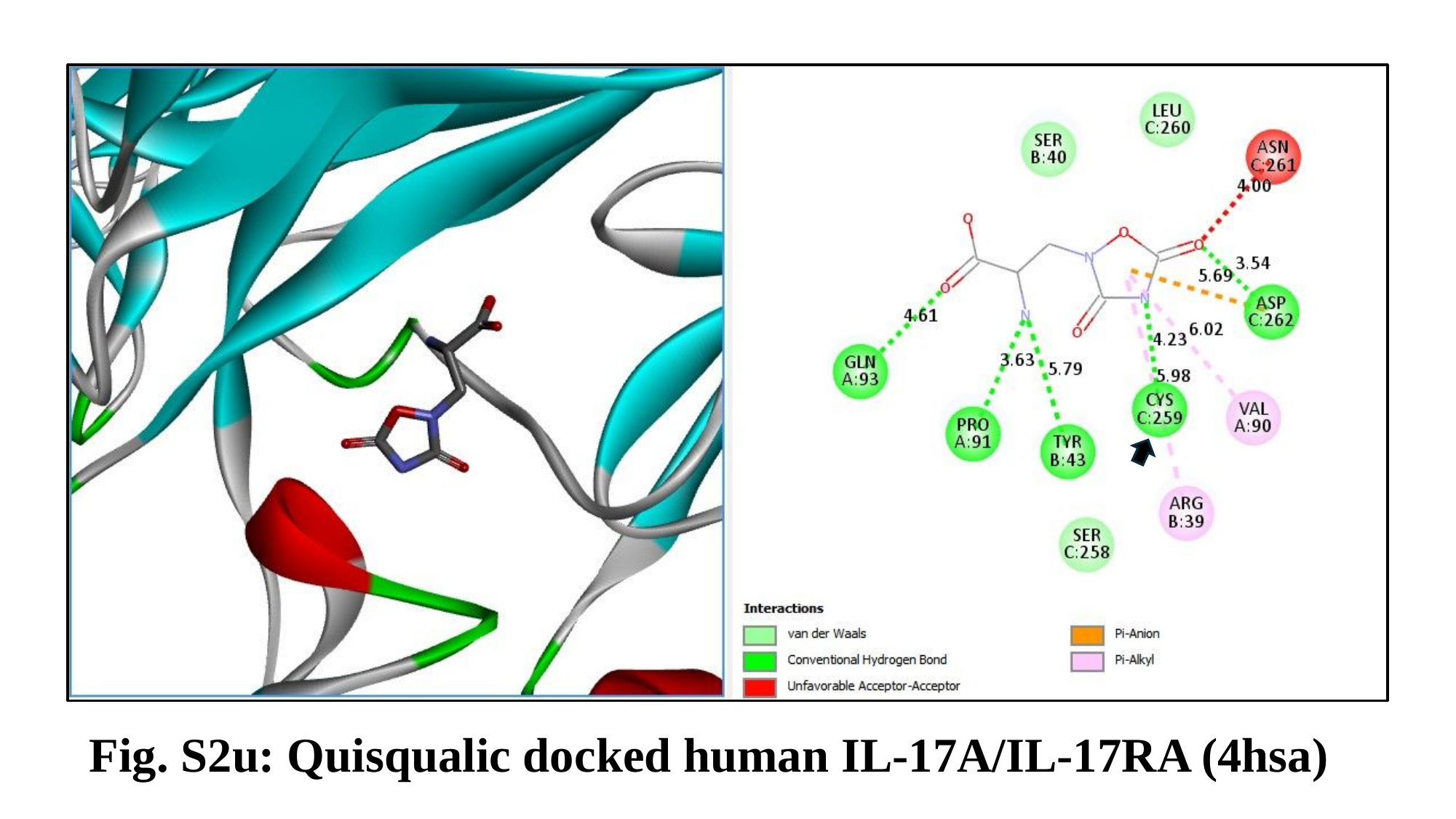

Fig. S2u: Quisqualic docked human IL-17A/IL-17RA (4hsa)

## Slide 23
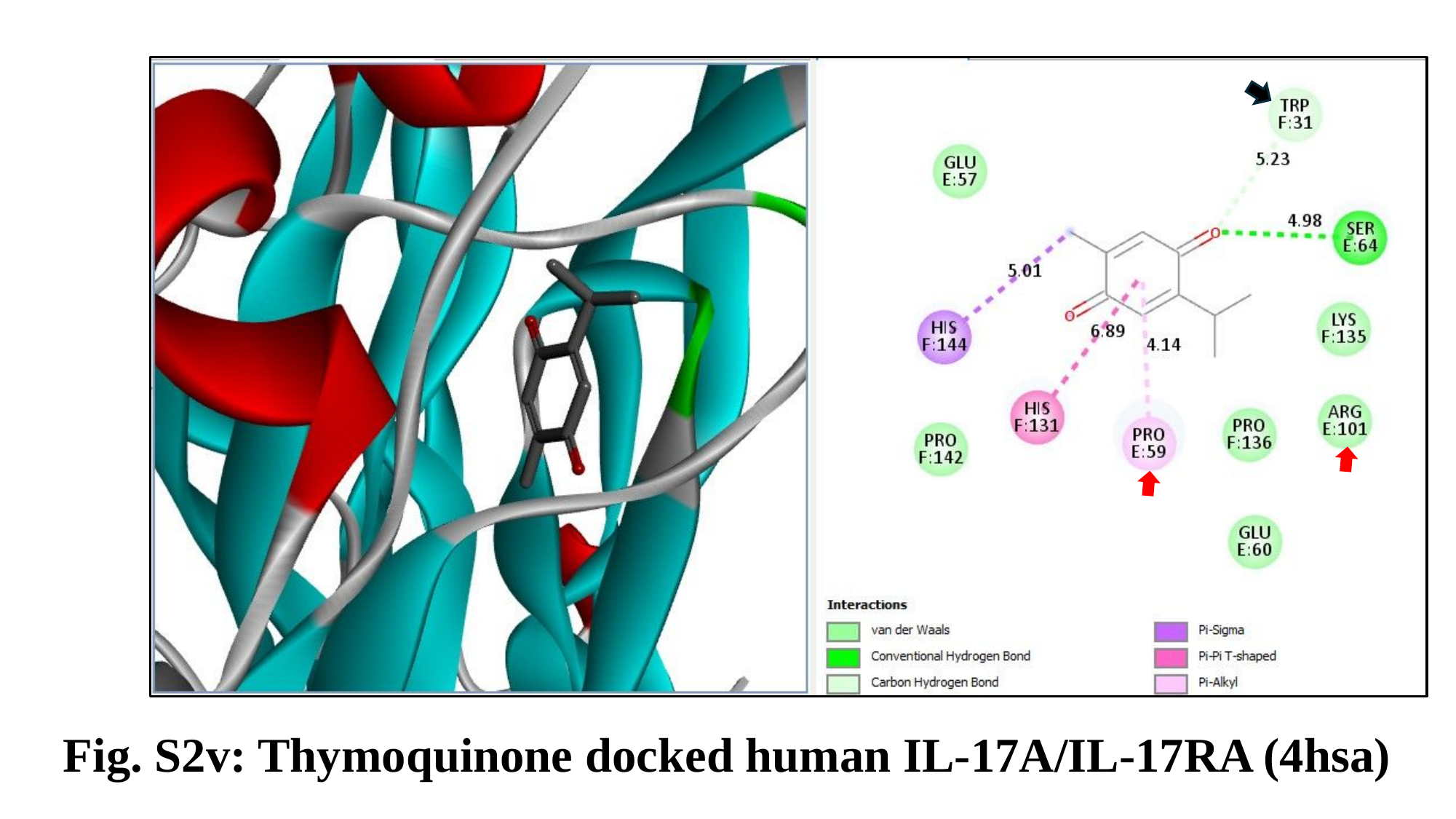

Fig. S2v: Thymoquinone docked human IL-17A/IL-17RA (4hsa)

## Slide 24
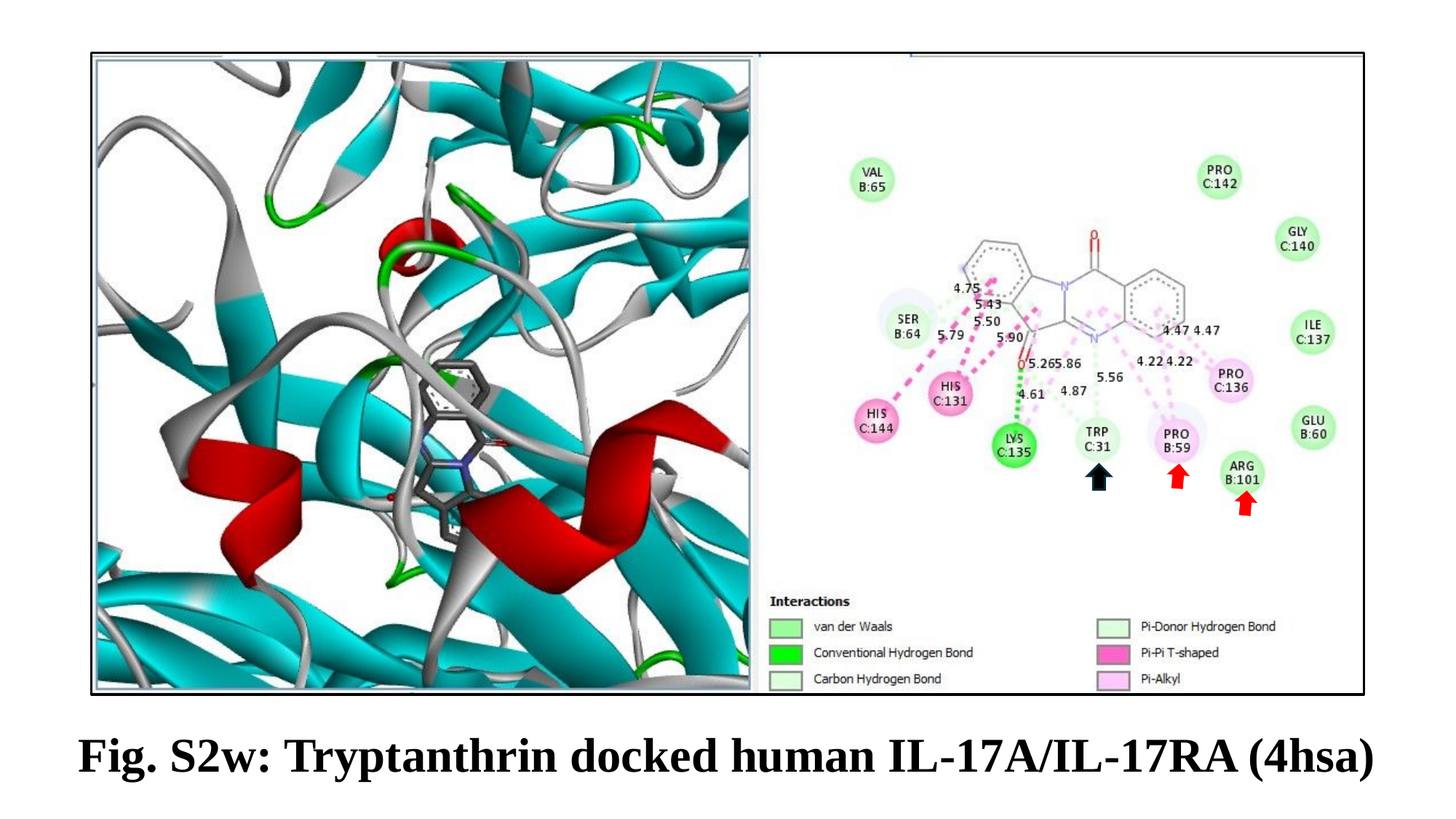

Fig. S2w: Tryptanthrin docked human IL-17A/IL-17RA (4hsa)
