# Supplementary material for: Targeting asparagine and cysteine in SARS-CoV-2 variants and human pro-inflammatory mediators to alleviate COVID-19 severity; a cross-section and in-silico study
Source: Sci Rep. 2025 Nov 3;15:38445. doi: 10.1038/s41598-025-19359-y (PMC12583749; doi:10.1038/s41598-025-19359-y)
Supplement: Supplementary file 10 — Supplementary Material 10 [file 41598_2025_19359_MOESM10_ESM.pptx]

## Slide 1
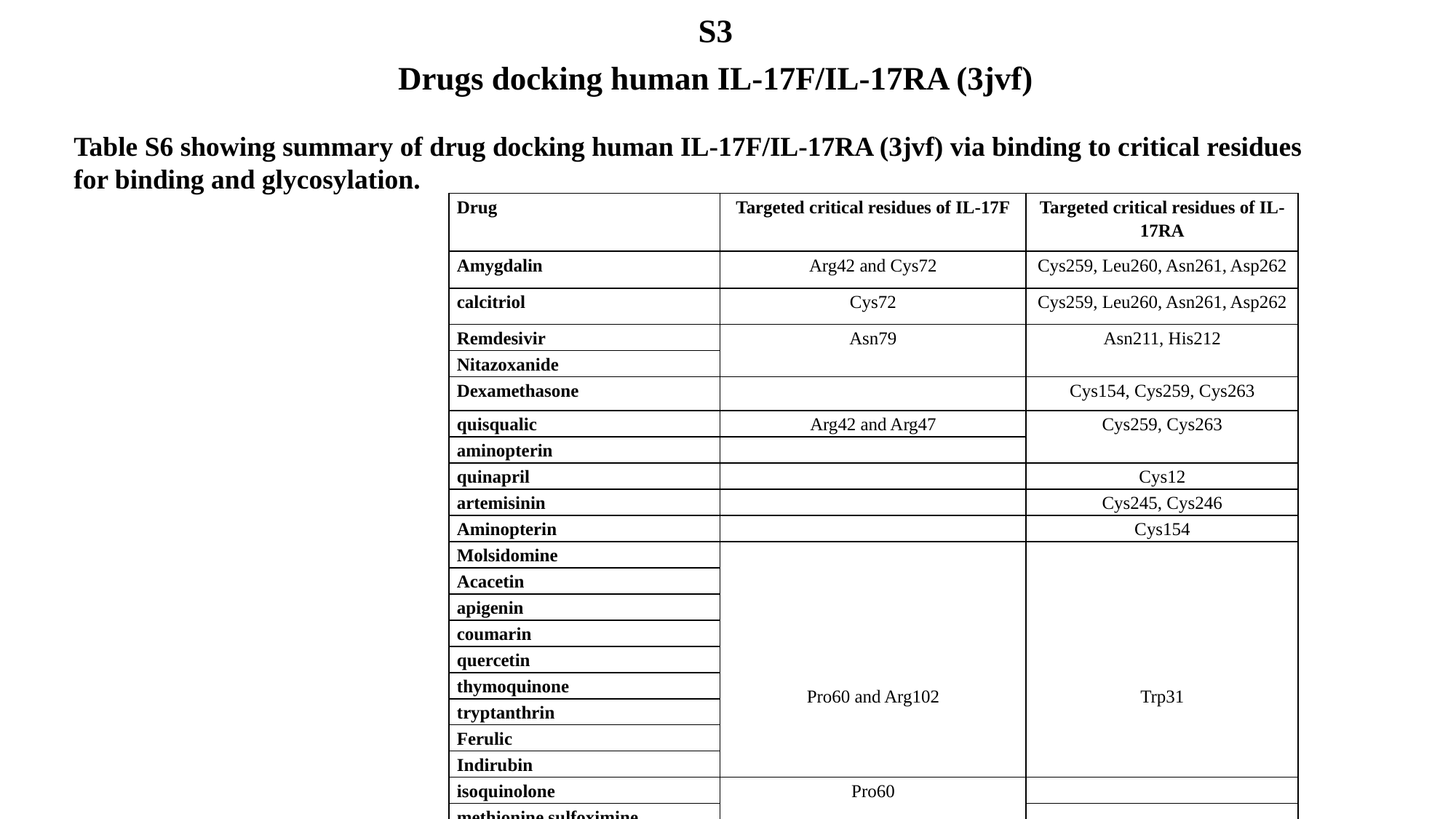

S3
Drugs docking human IL-17F/IL-17RA (3jvf)
Table S6 showing summary of drug docking human IL-17F/IL-17RA (3jvf) via binding to critical residues for binding and glycosylation.
| Drug | Targeted critical residues of IL-17F | Targeted critical residues of IL-17RA |
| --- | --- | --- |
| Amygdalin | Arg42 and Cys72 | Cys259, Leu260, Asn261, Asp262 |
| calcitriol | Cys72 | Cys259, Leu260, Asn261, Asp262 |
| Remdesivir | Asn79 | Asn211, His212 |
| Nitazoxanide | | |
| Dexamethasone | | Cys154, Cys259, Cys263 |
| quisqualic | Arg42 and Arg47 | Cys259, Cys263 |
| aminopterin | | |
| quinapril | | Cys12 |
| artemisinin | | Cys245, Cys246 |
| Aminopterin | | Cys154 |
| Molsidomine | Pro60 and Arg102 | Trp31 |
| Acacetin | | |
| apigenin | | |
| coumarin | | |
| quercetin | | |
| thymoquinone | | |
| tryptanthrin | | |
| Ferulic | | |
| Indirubin | | |
| isoquinolone | Pro60 | |
| methionine sulfoximine | | |

## Slide 2
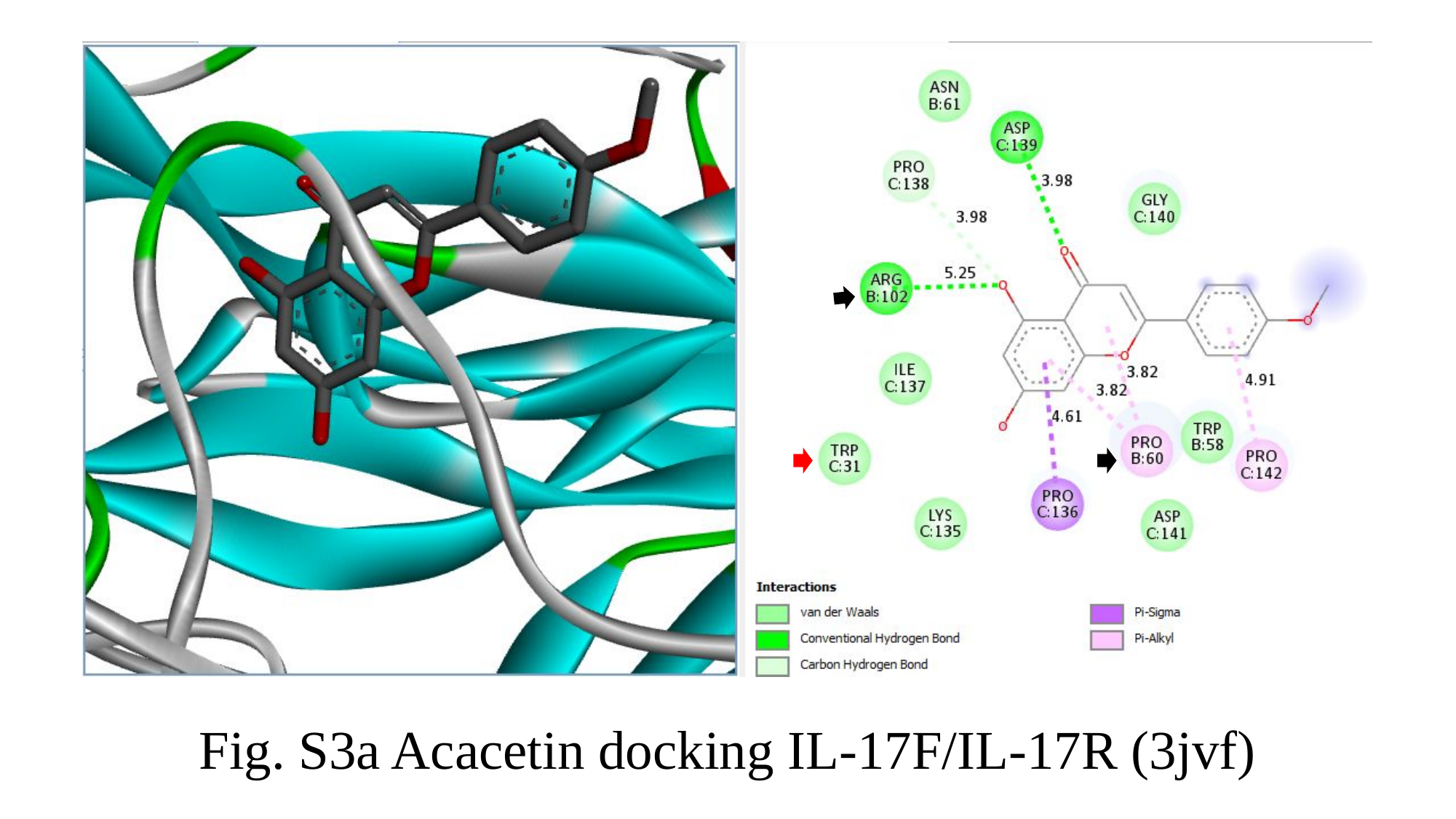

Fig. S3a Acacetin docking IL-17F/IL-17R (3jvf)

## Slide 3
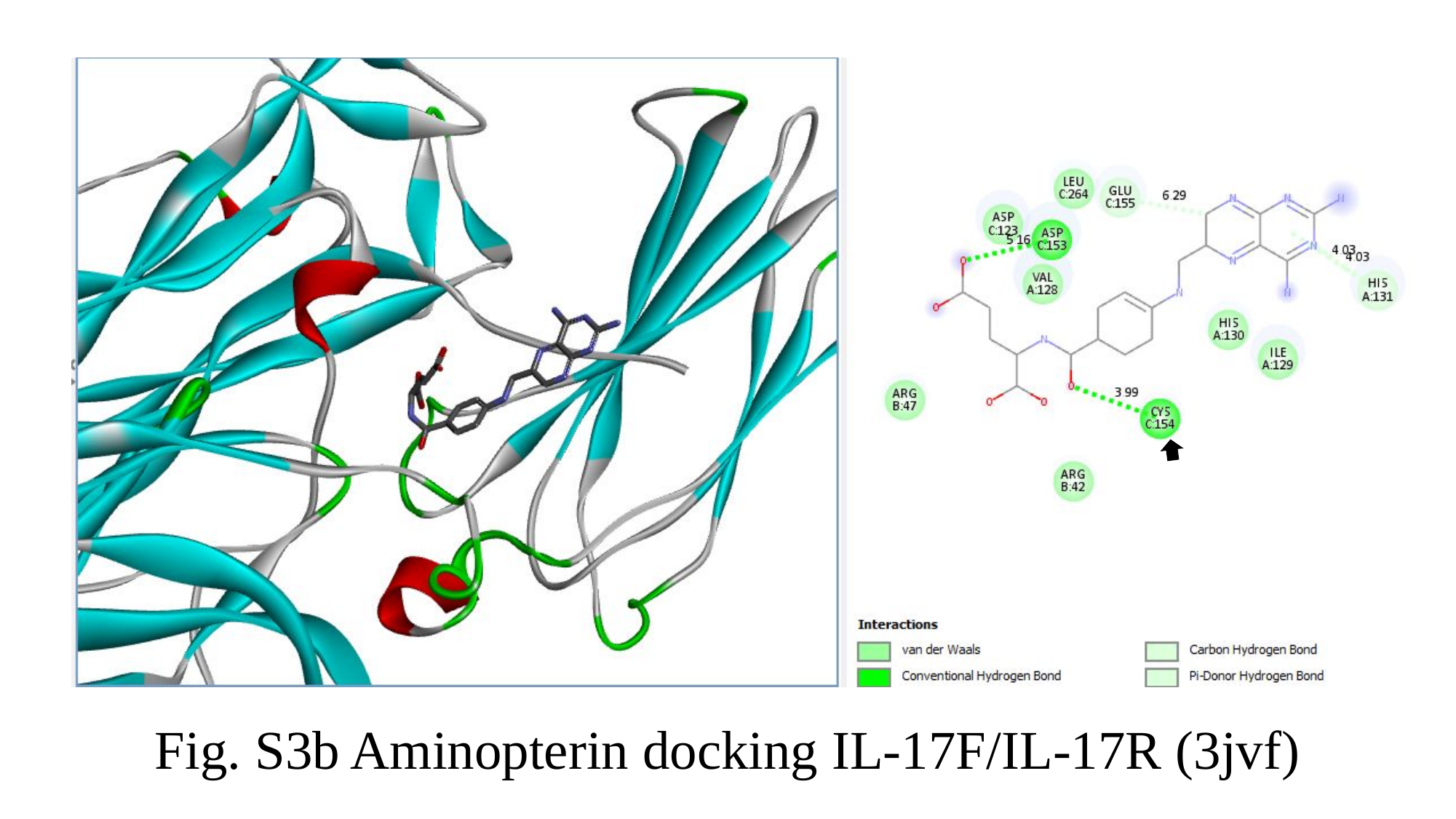

Fig. S3b Aminopterin docking IL-17F/IL-17R (3jvf)

## Slide 4
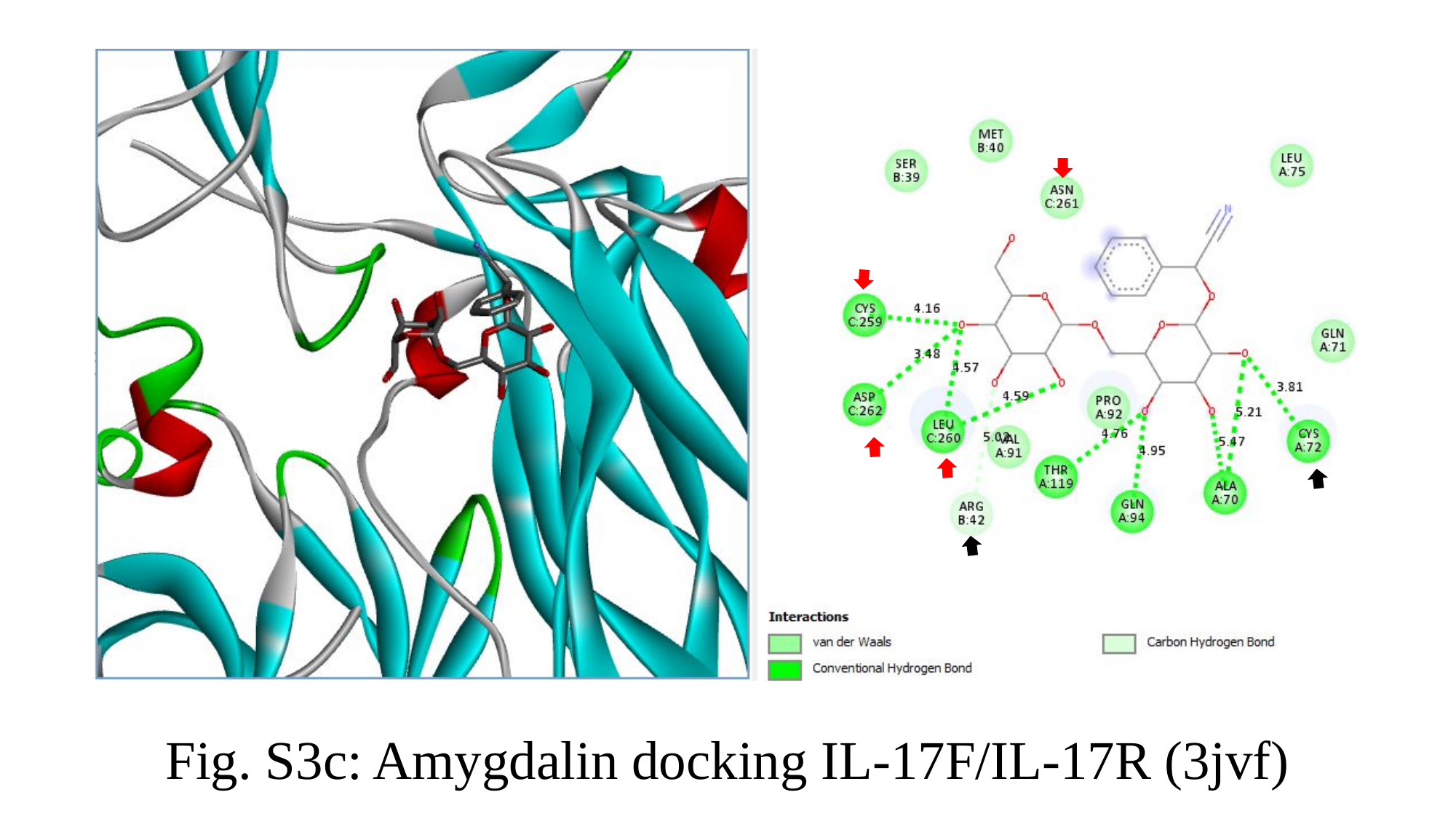

Fig. S3c: Amygdalin docking IL-17F/IL-17R (3jvf)

## Slide 5
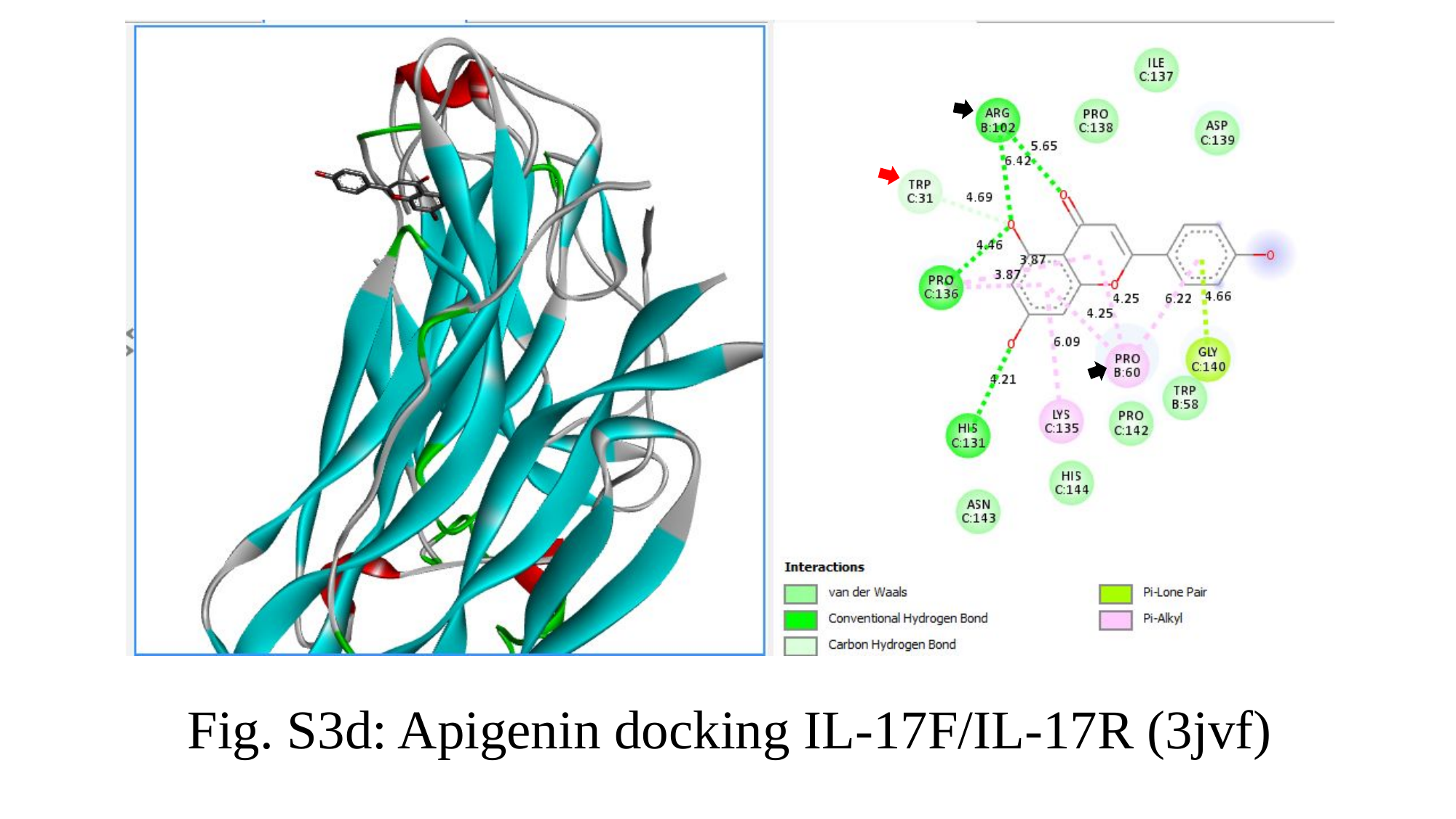

Fig. S3d: Apigenin docking IL-17F/IL-17R (3jvf)

## Slide 6
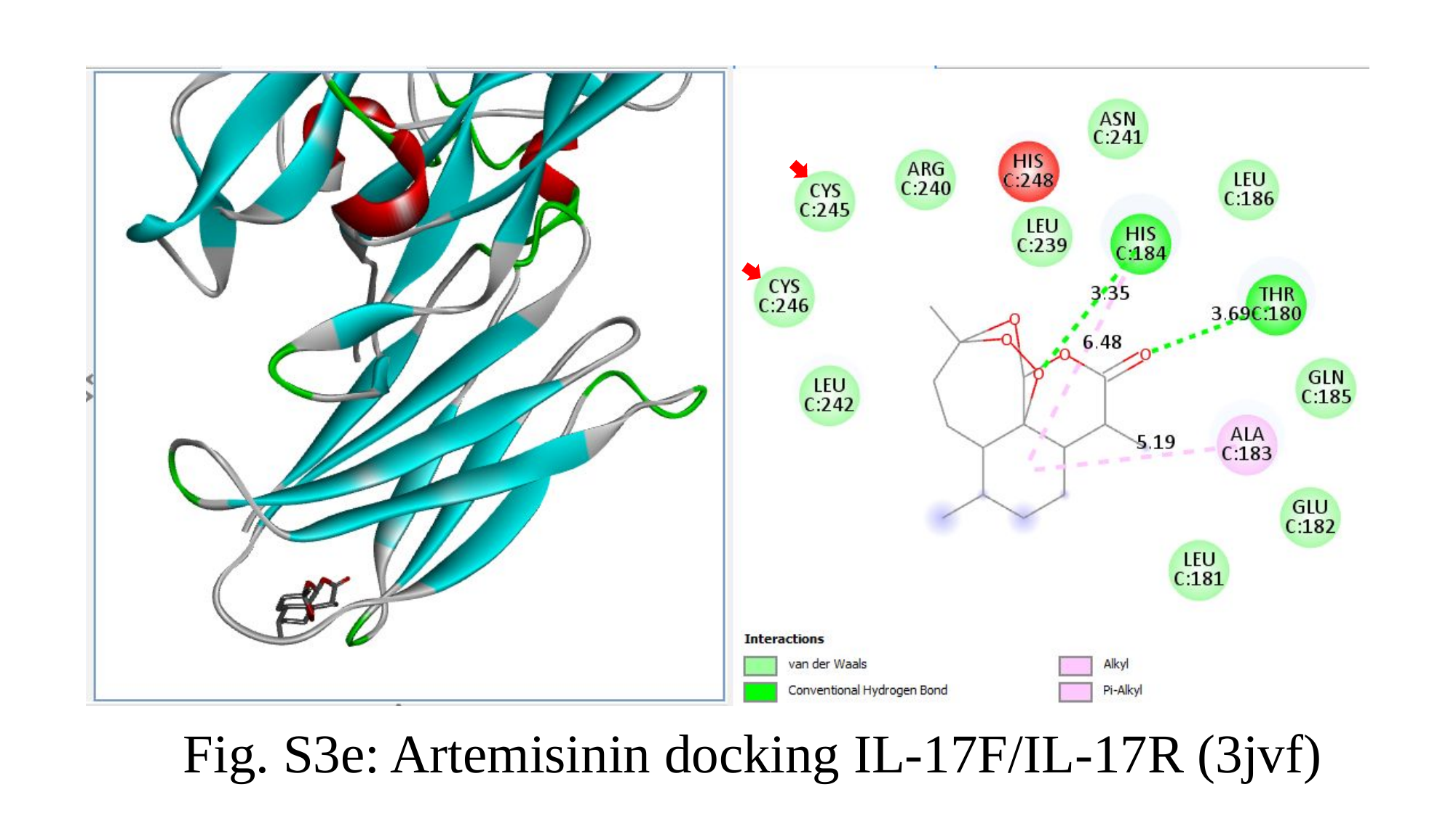

Fig. S3e: Artemisinin docking IL-17F/IL-17R (3jvf)

## Slide 7
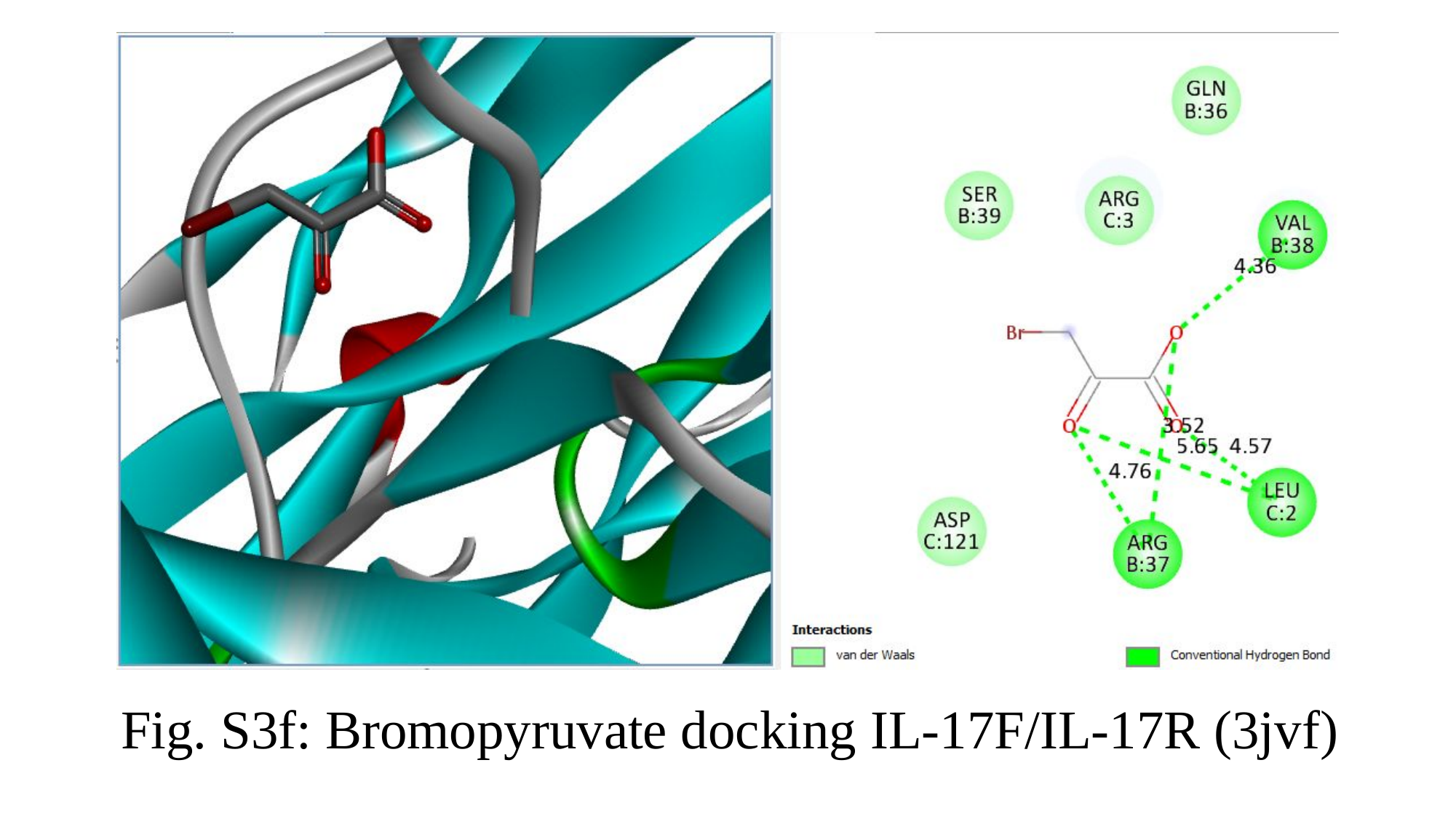

Fig. S3f: Bromopyruvate docking IL-17F/IL-17R (3jvf)

## Slide 8
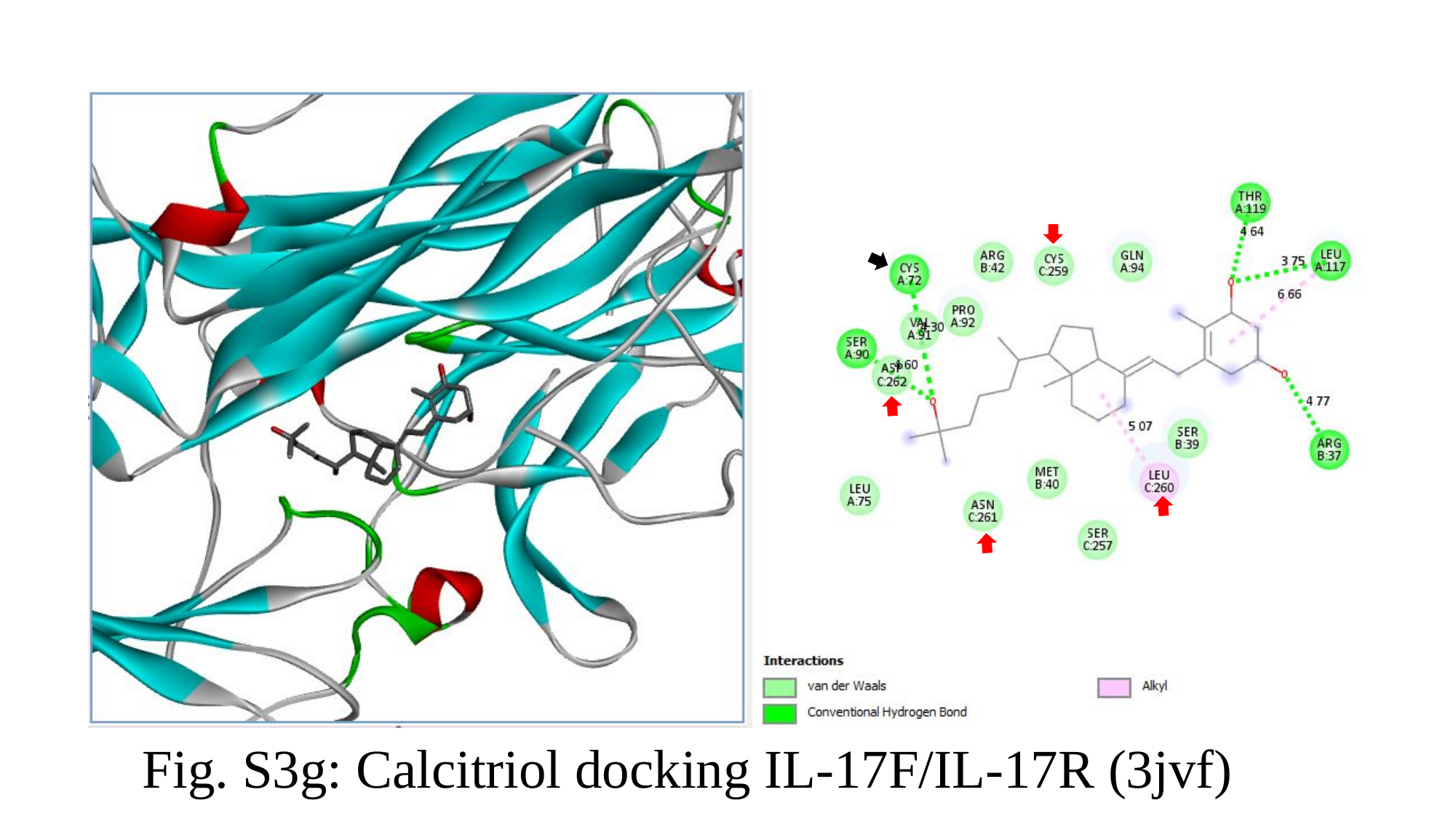

Fig. S3g: Calcitriol docking IL-17F/IL-17R (3jvf)

## Slide 9
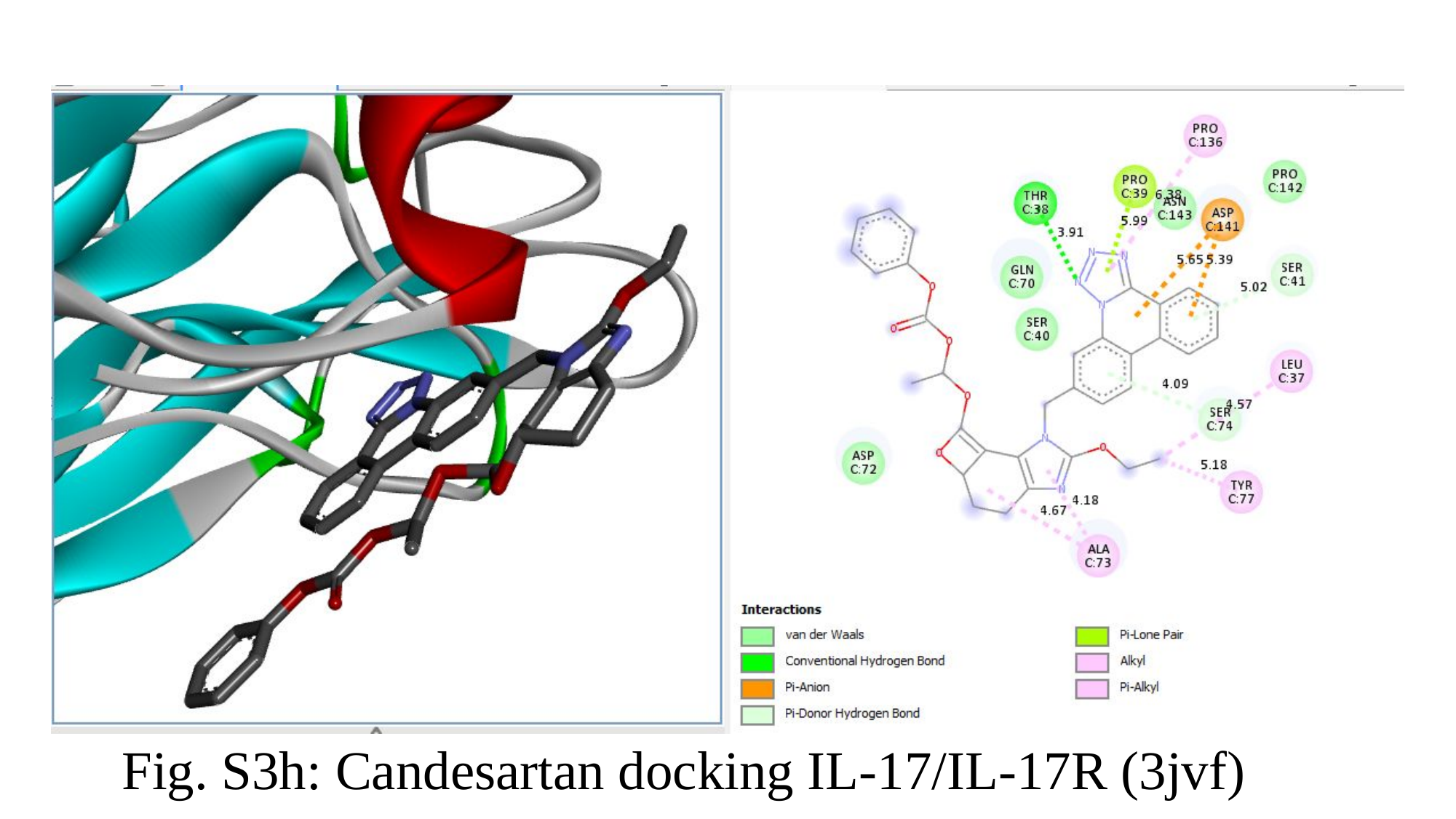

Fig. S3h: Candesartan docking IL-17/IL-17R (3jvf)

## Slide 10
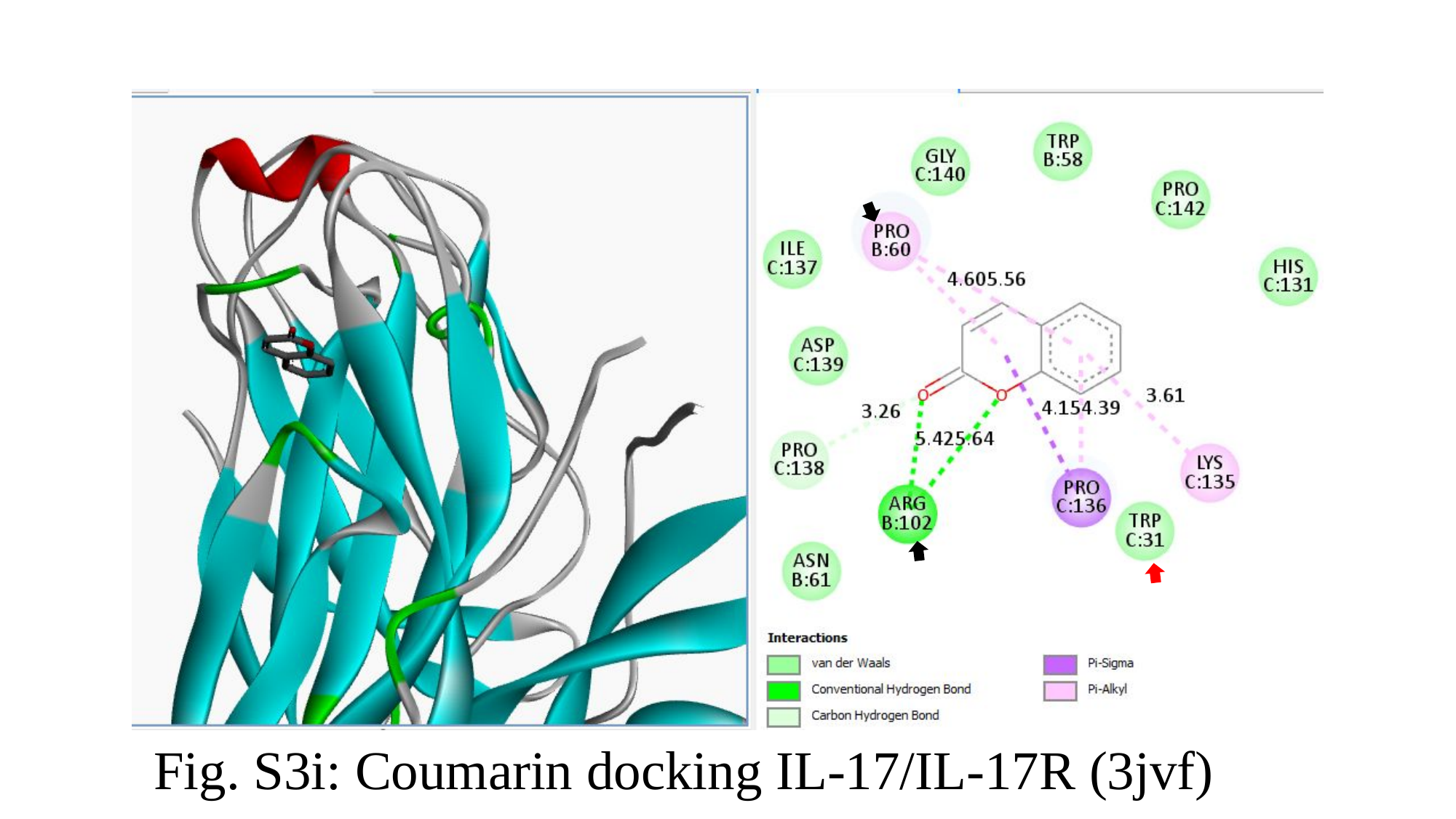

Fig. S3i: Coumarin docking IL-17/IL-17R (3jvf)

## Slide 11
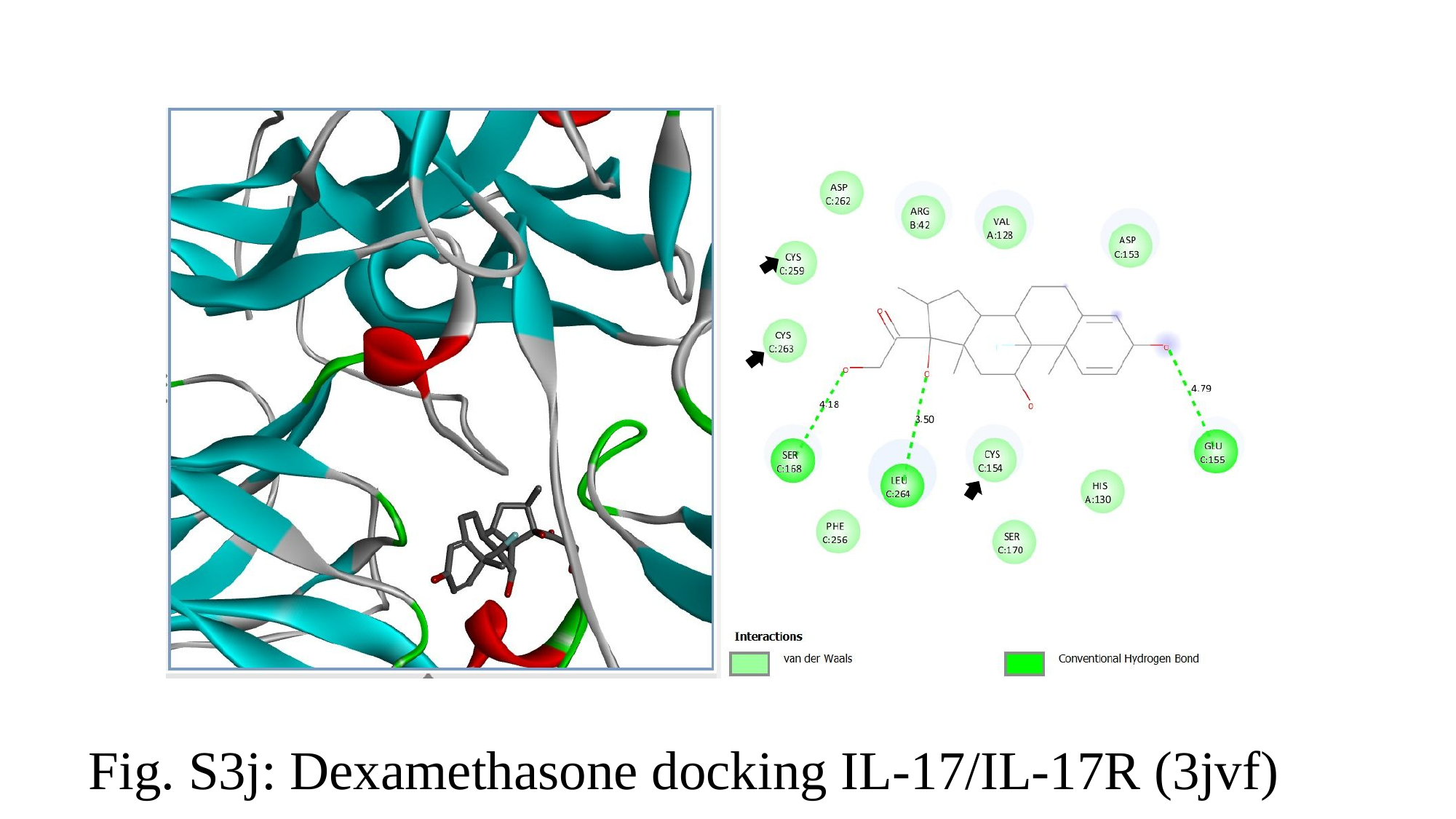

Fig. S3j: Dexamethasone docking IL-17/IL-17R (3jvf)

## Slide 12
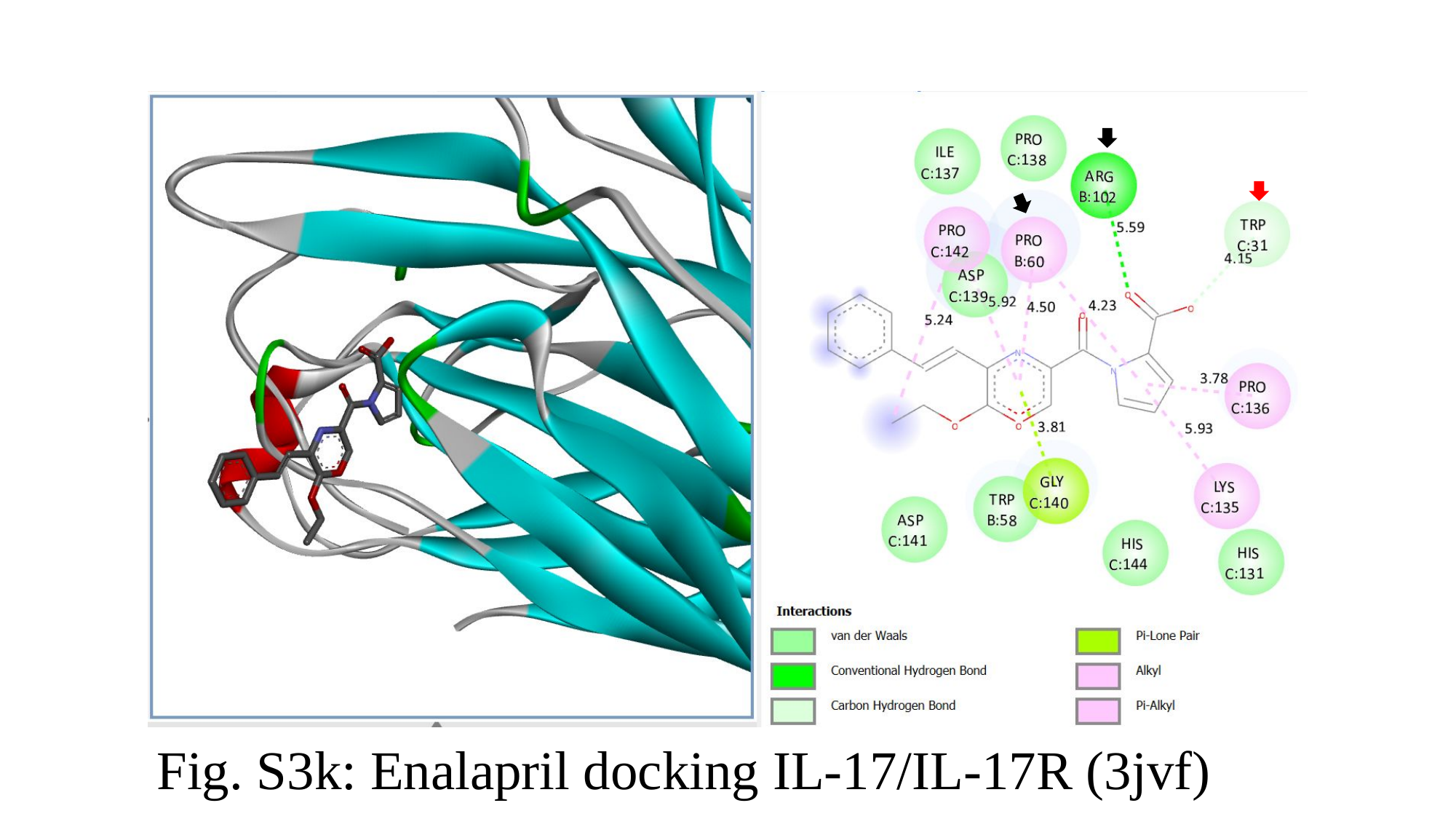

Fig. S3k: Enalapril docking IL-17/IL-17R (3jvf)

## Slide 13
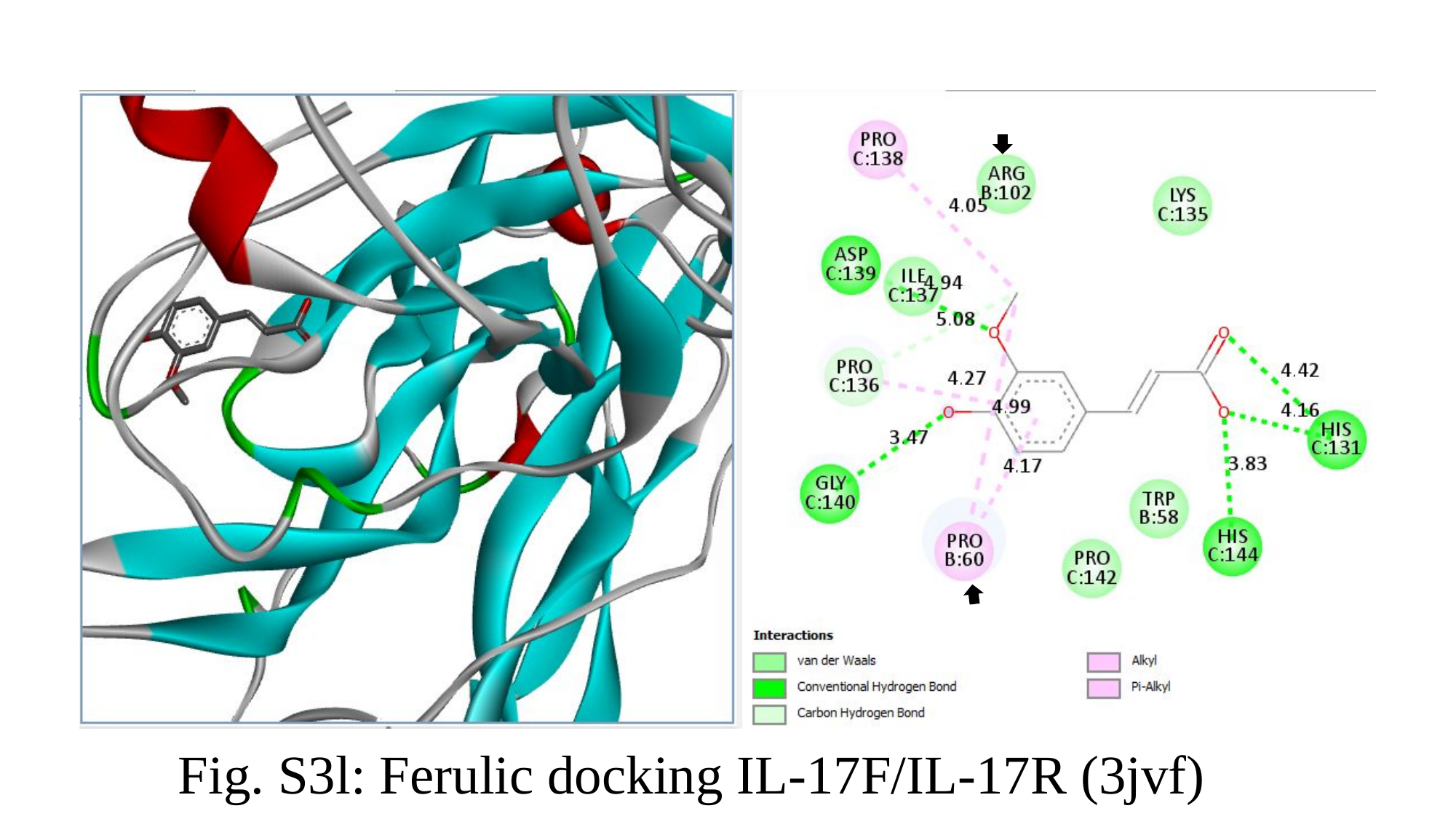

Fig. S3l: Ferulic docking IL-17F/IL-17R (3jvf)

## Slide 14
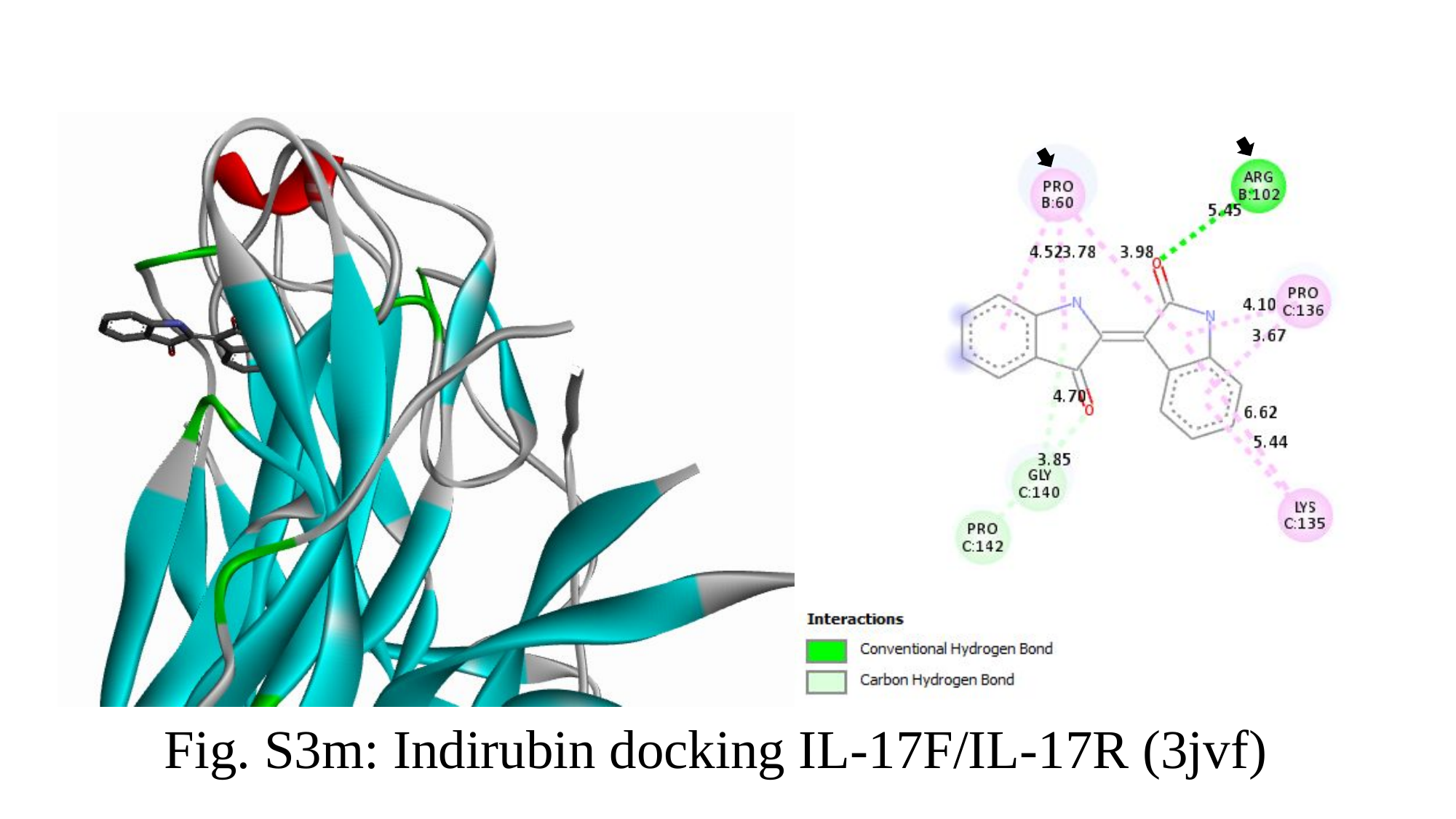

Fig. S3m: Indirubin docking IL-17F/IL-17R (3jvf)

## Slide 15
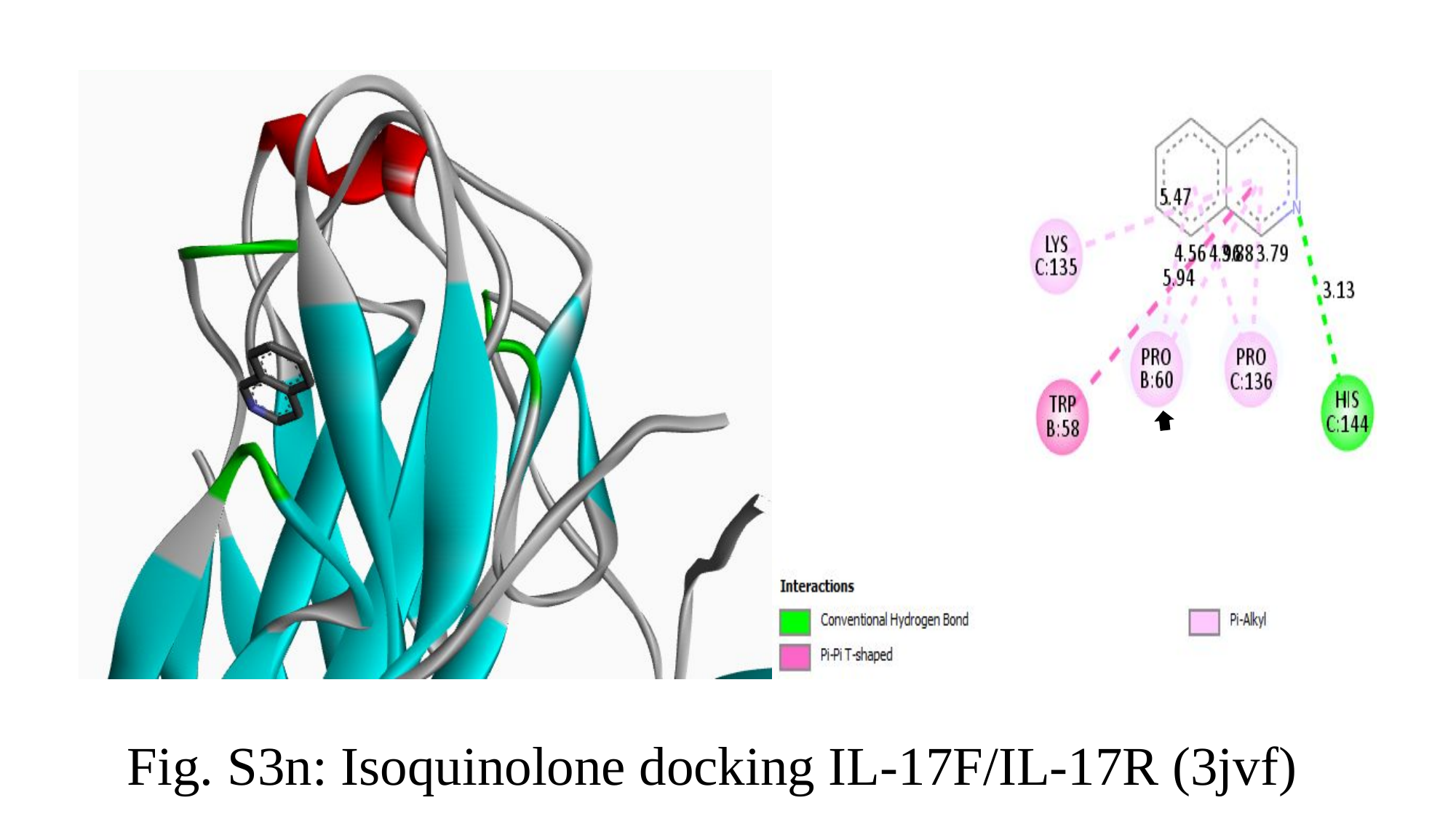

Fig. S3n: Isoquinolone docking IL-17F/IL-17R (3jvf)

## Slide 16
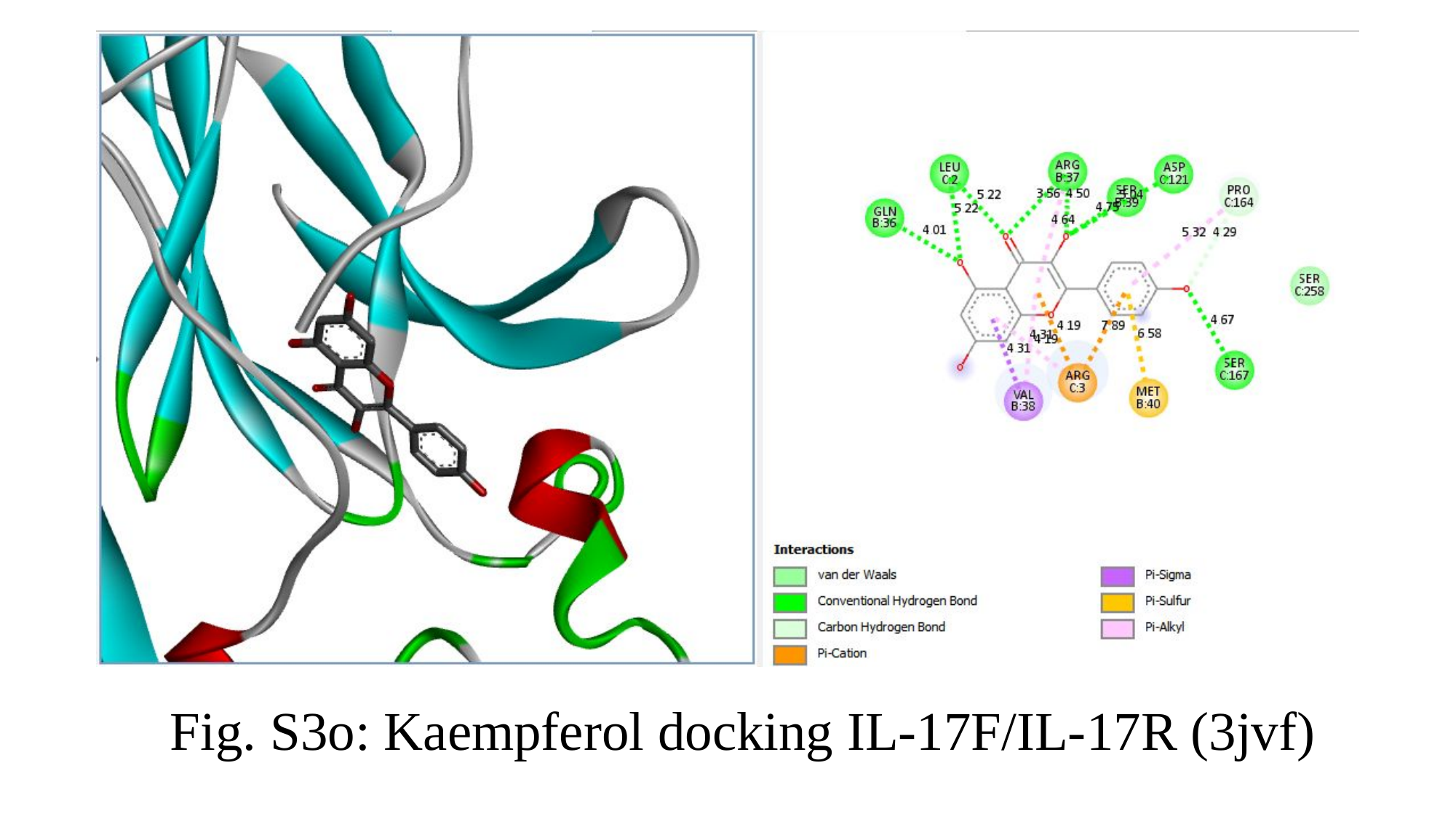

Fig. S3o: Kaempferol docking IL-17F/IL-17R (3jvf)

## Slide 17
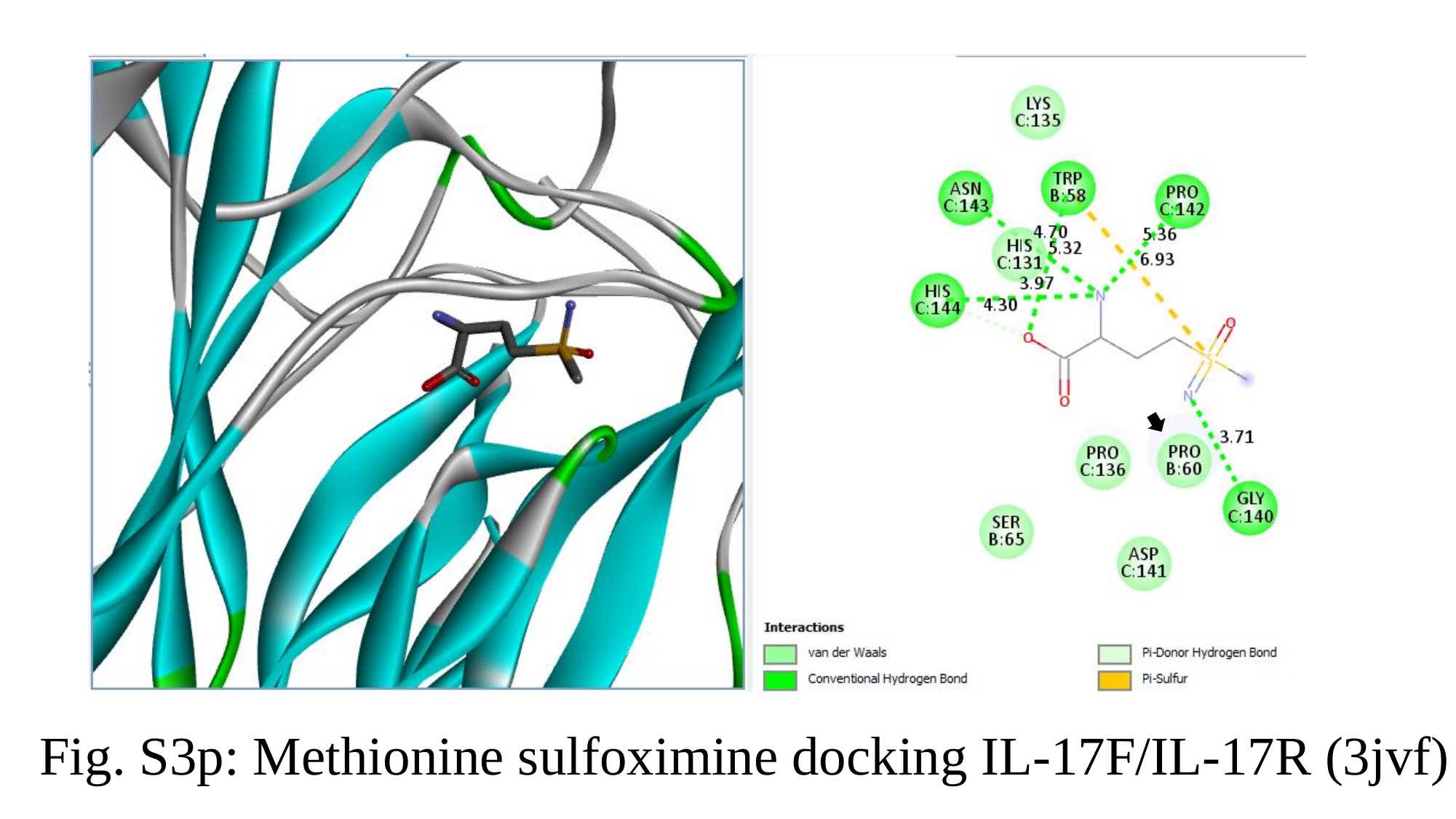

Fig. S3p: Methionine sulfoximine docking IL-17F/IL-17R (3jvf)

## Slide 18
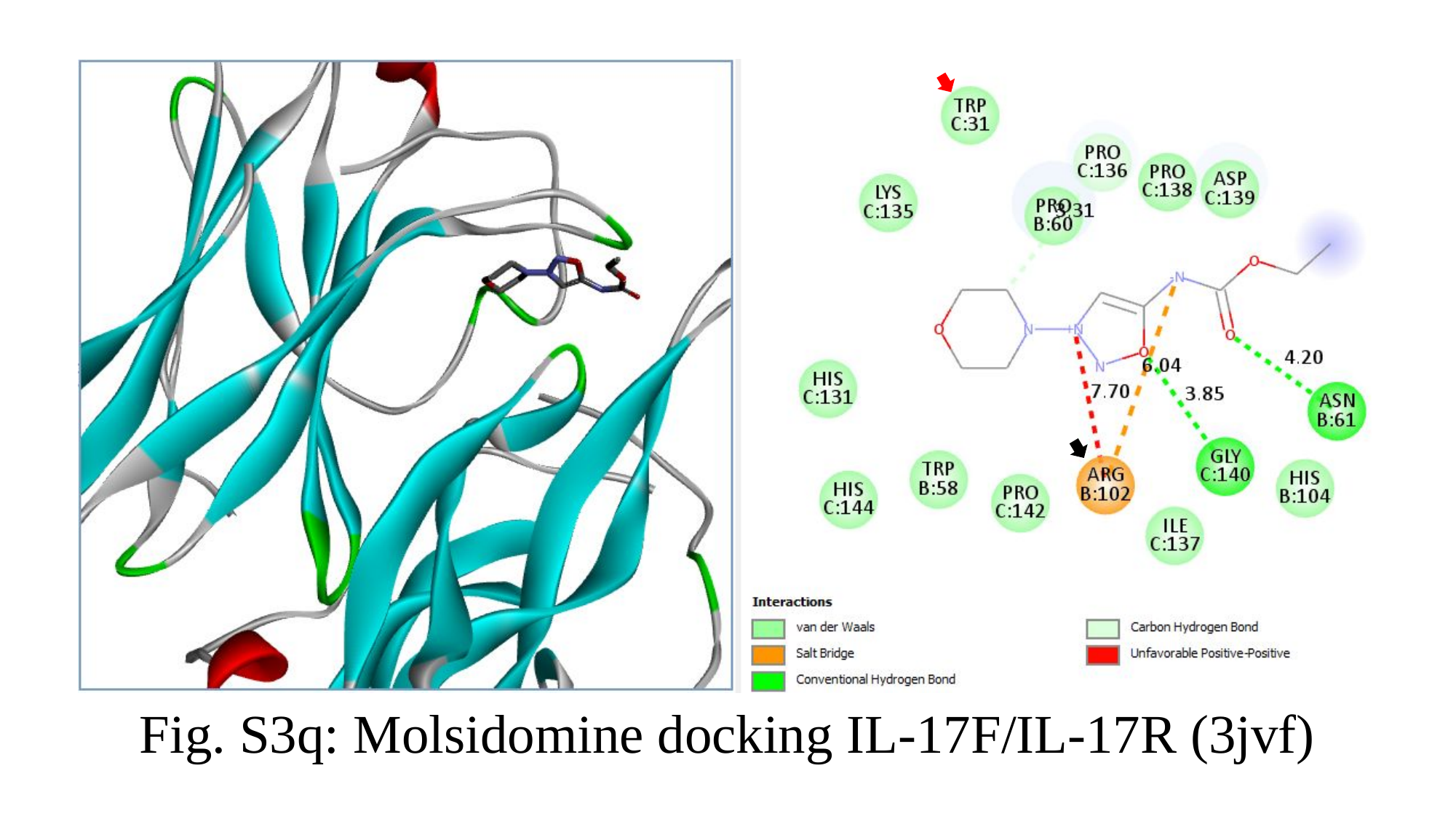

Fig. S3q: Molsidomine docking IL-17F/IL-17R (3jvf)

## Slide 19
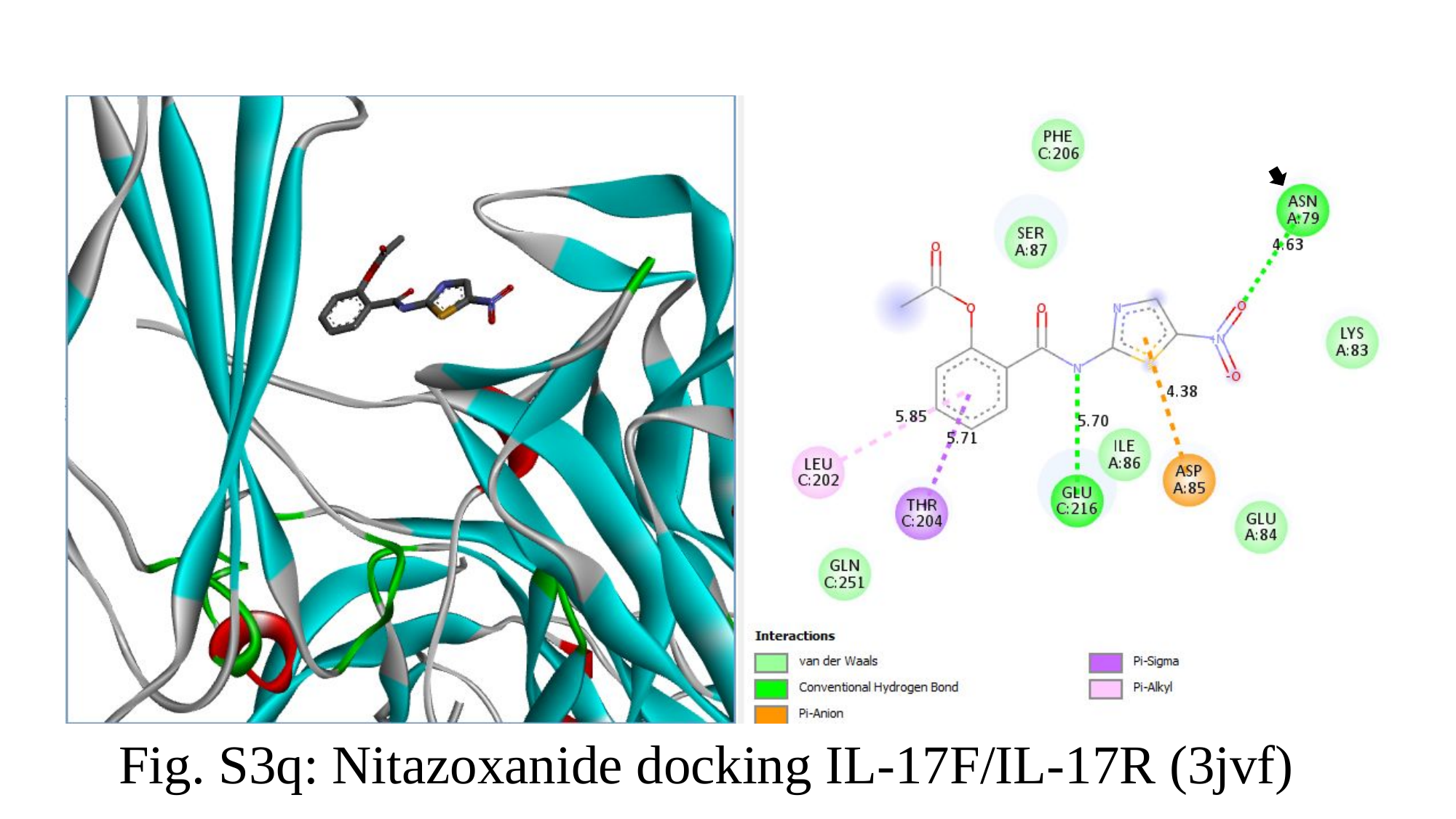

Fig. S3q: Nitazoxanide docking IL-17F/IL-17R (3jvf)

## Slide 20
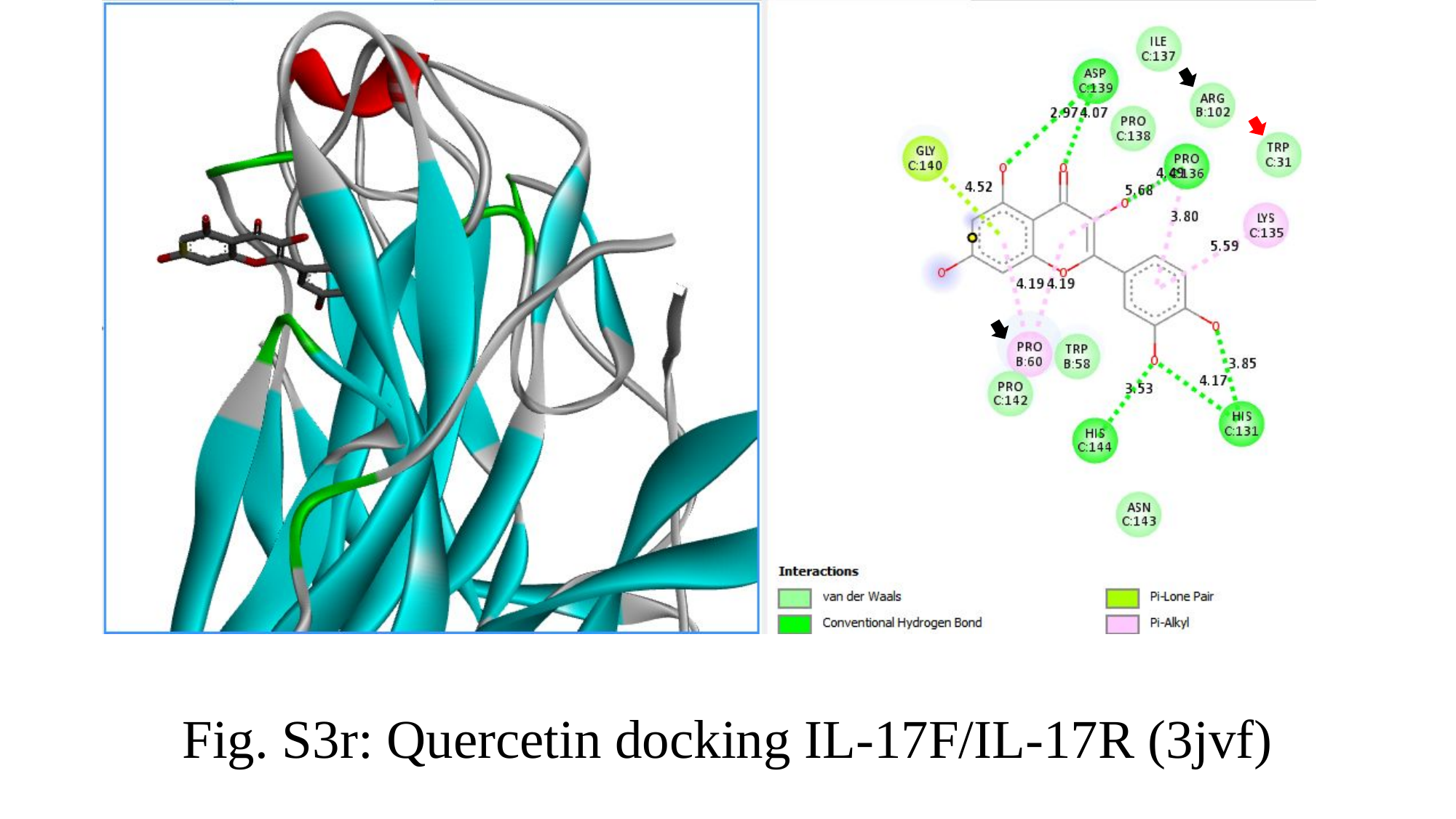

Fig. S3r: Quercetin docking IL-17F/IL-17R (3jvf)

## Slide 21
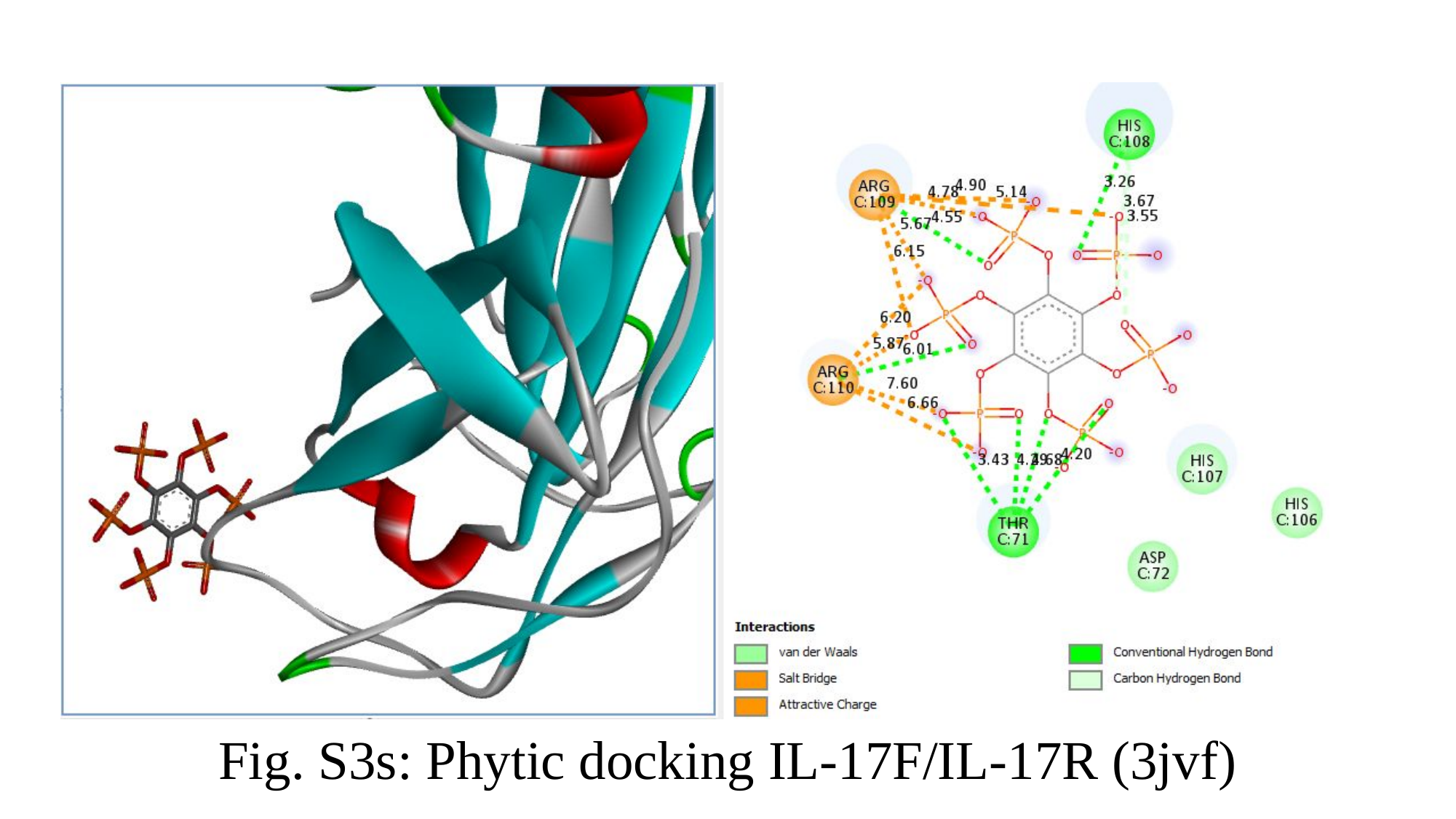

Fig. S3s: Phytic docking IL-17F/IL-17R (3jvf)

## Slide 22
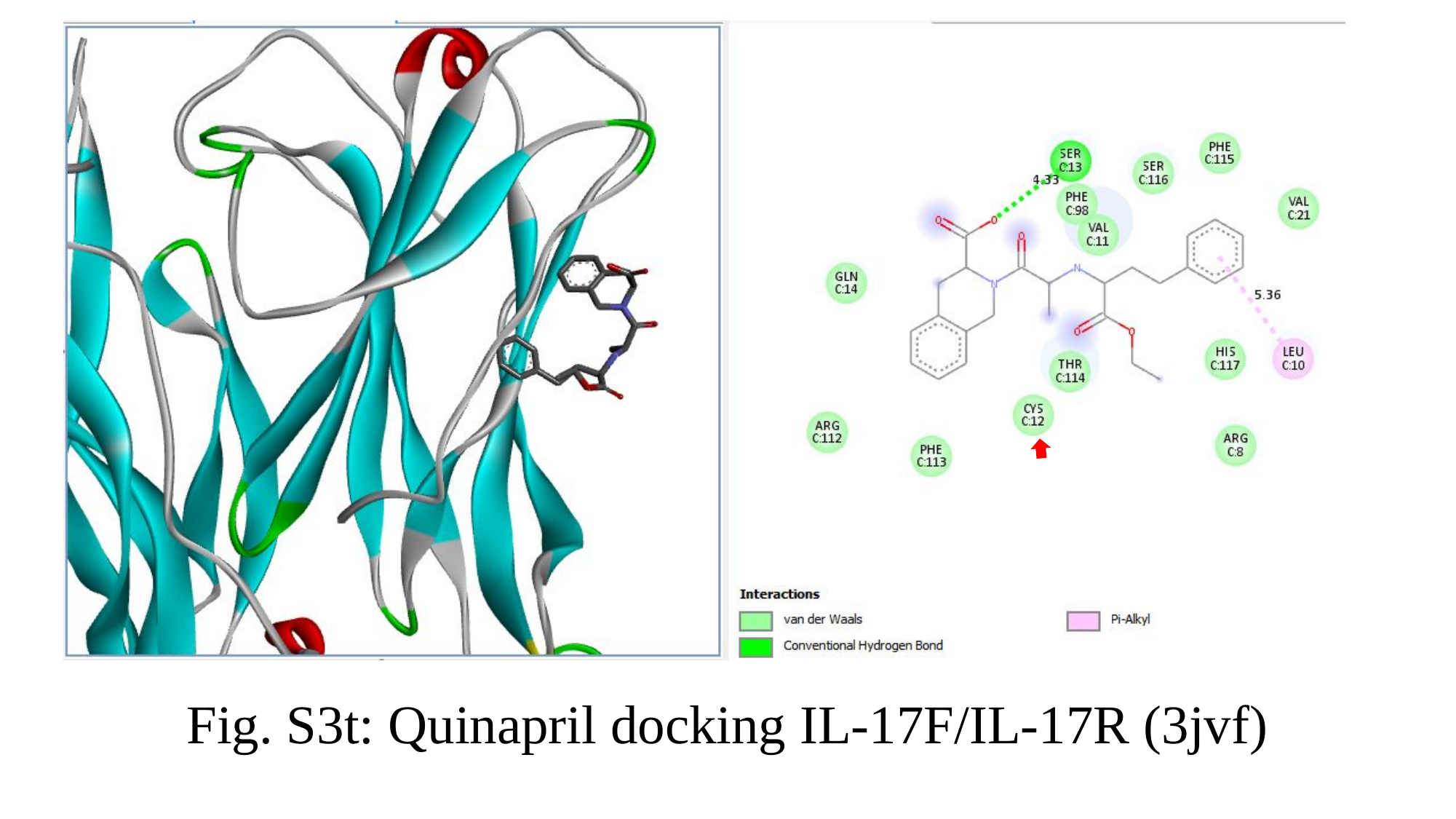

Fig. S3t: Quinapril docking IL-17F/IL-17R (3jvf)

## Slide 23
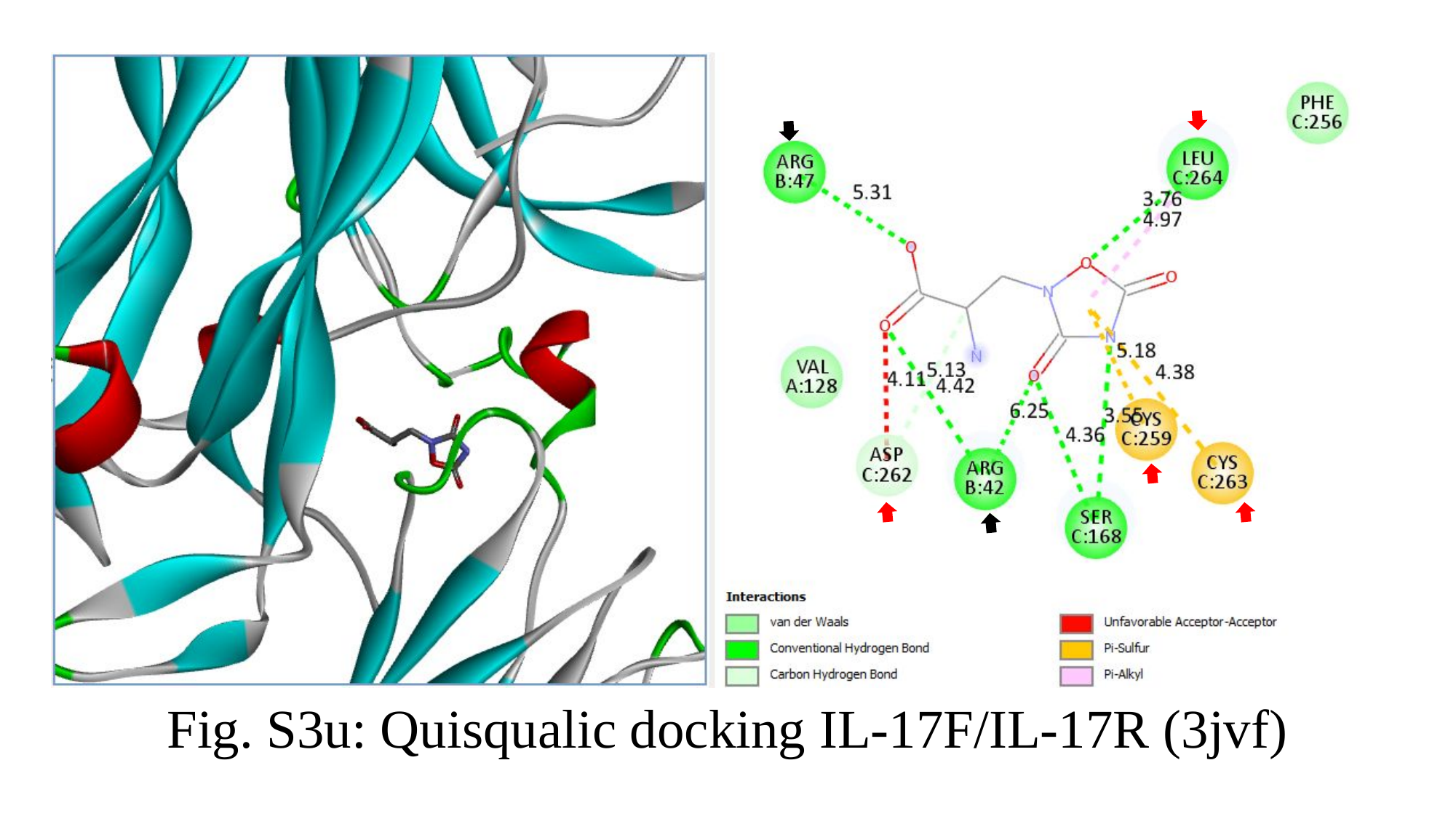

Fig. S3u: Quisqualic docking IL-17F/IL-17R (3jvf)

## Slide 24
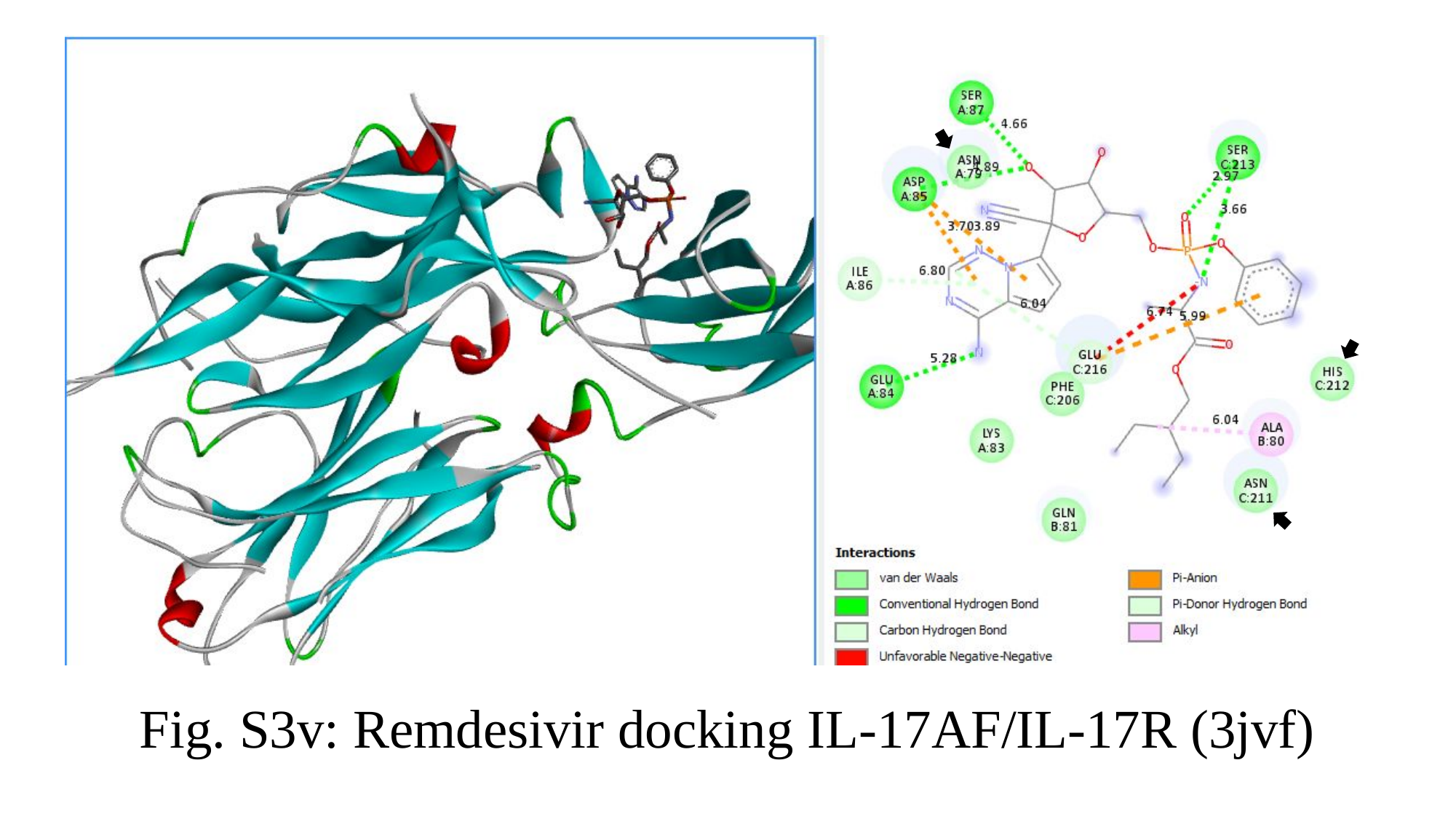

Fig. S3v: Remdesivir docking IL-17AF/IL-17R (3jvf)

## Slide 25
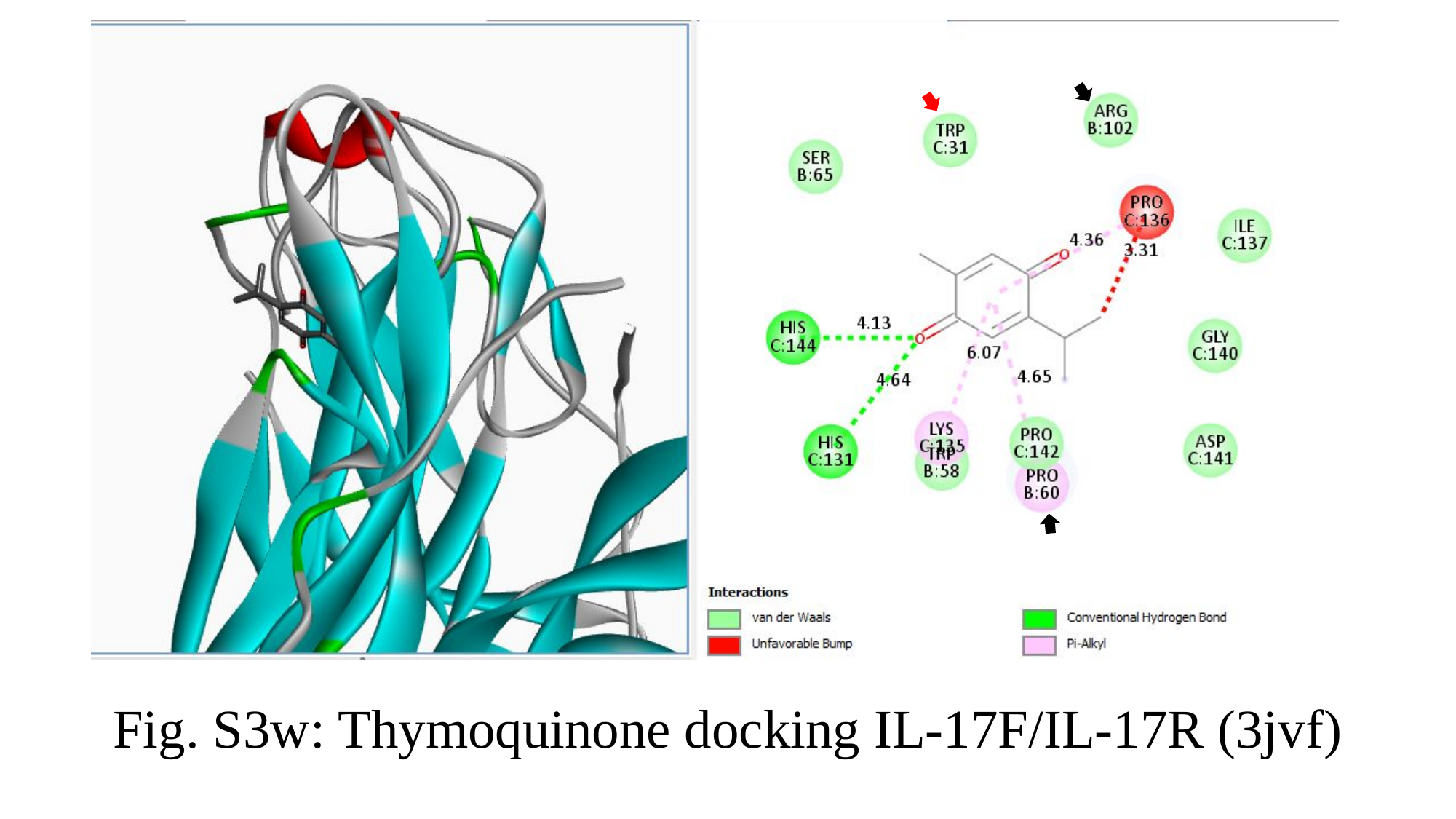

Fig. S3w: Thymoquinone docking IL-17F/IL-17R (3jvf)

## Slide 26
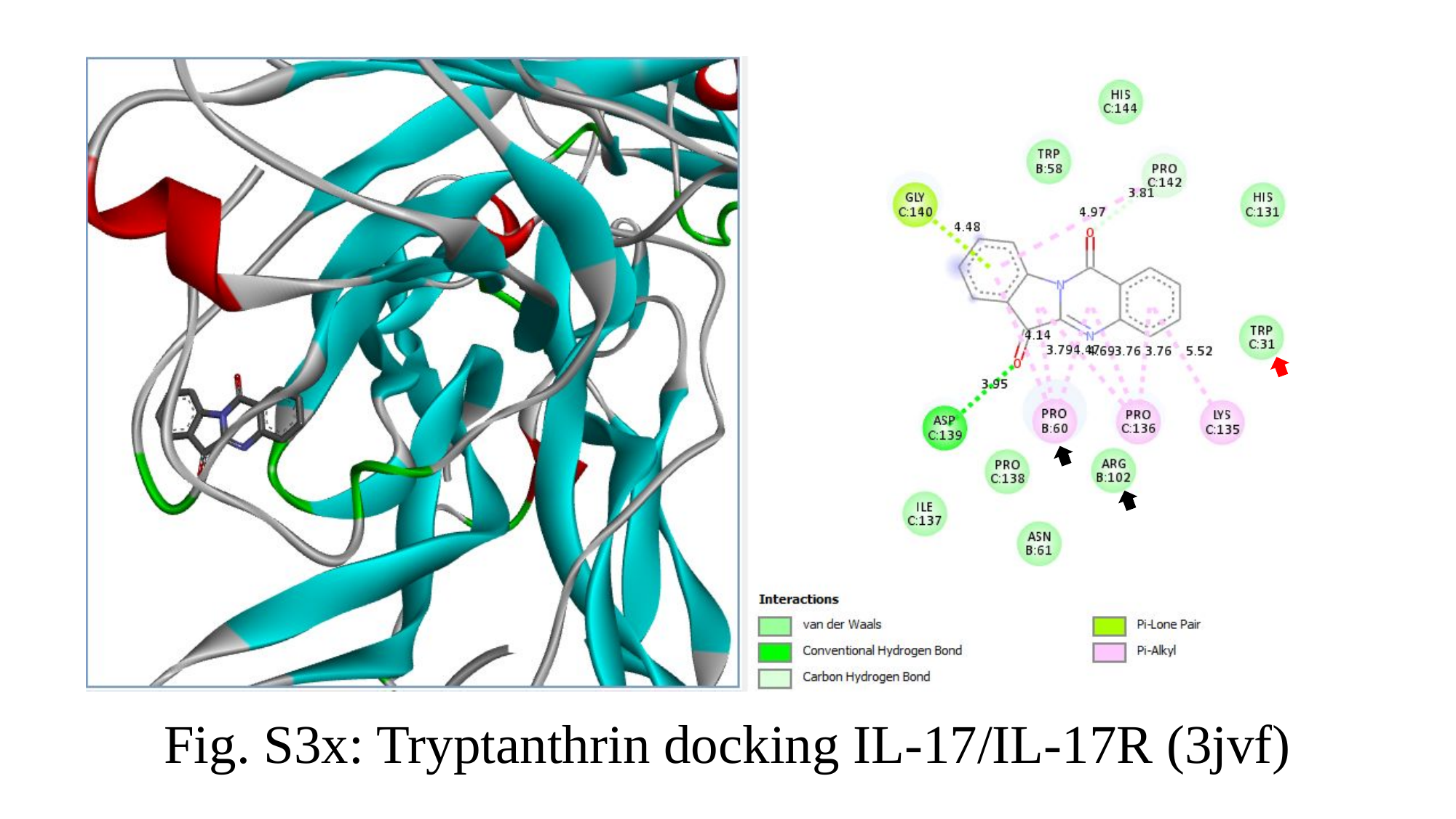

Fig. S3x: Tryptanthrin docking IL-17/IL-17R (3jvf)
