# Supplementary material for: Targeting asparagine and cysteine in SARS-CoV-2 variants and human pro-inflammatory mediators to alleviate COVID-19 severity; a cross-section and in-silico study
Source: Sci Rep. 2025 Nov 3;15:38445. doi: 10.1038/s41598-025-19359-y (PMC12583749; doi:10.1038/s41598-025-19359-y)
Supplement: Supplementary file 11 — Supplementary Material 11 [file 41598_2025_19359_MOESM11_ESM.pptx]

## Slide 1
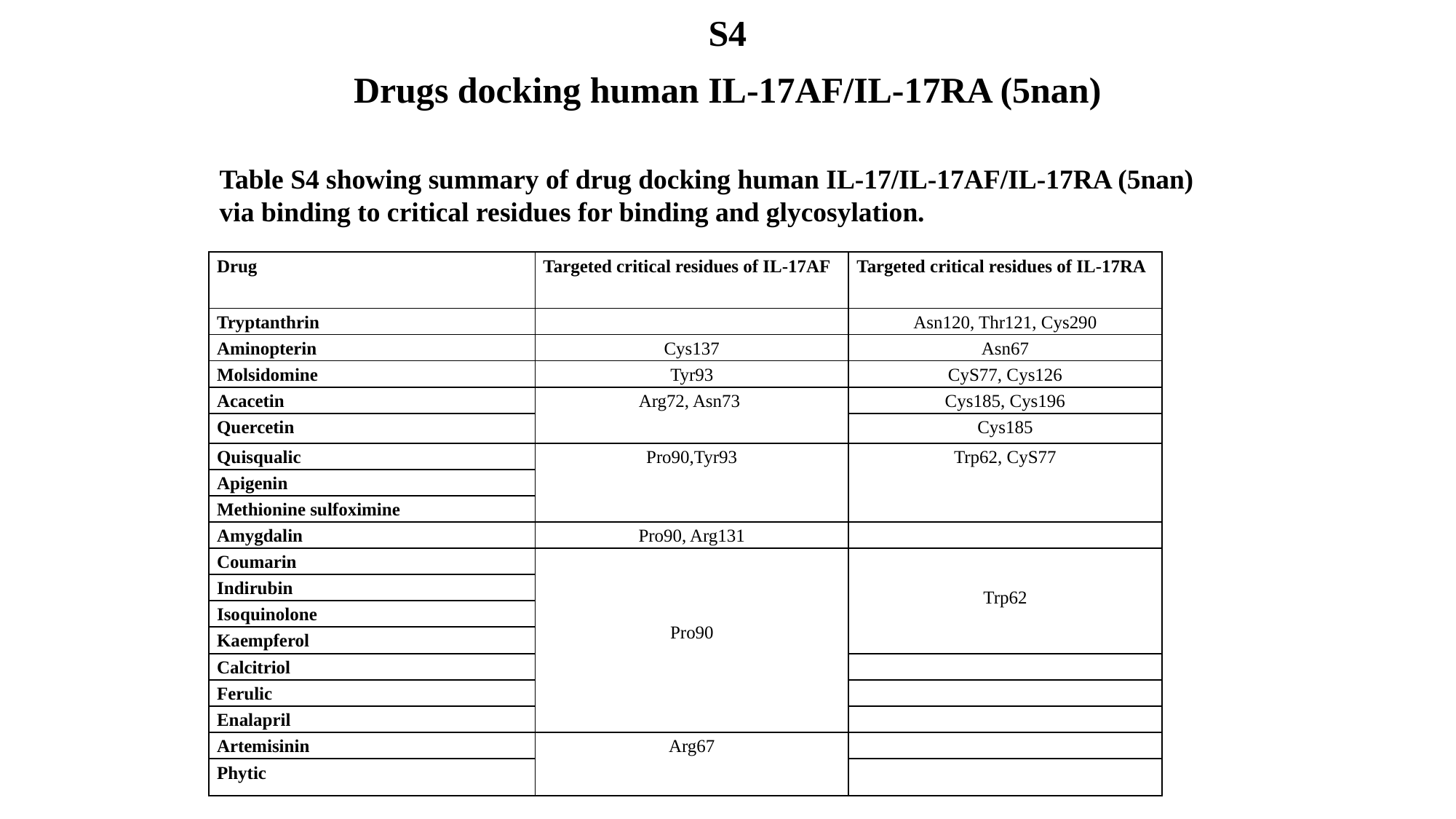

S4
Drugs docking human IL-17AF/IL-17RA (5nan)
Table S4 showing summary of drug docking human IL-17/IL-17AF/IL-17RA (5nan) via binding to critical residues for binding and glycosylation.
| Drug | Targeted critical residues of IL-17AF | Targeted critical residues of IL-17RA |
| --- | --- | --- |
| Tryptanthrin | | Asn120, Thr121, Cys290 |
| Aminopterin | Cys137 | Asn67 |
| Molsidomine | Tyr93 | CyS77, Cys126 |
| Acacetin | Arg72, Asn73 | Cys185, Cys196 |
| Quercetin | | Cys185 |
| Quisqualic | Pro90,Tyr93 | Trp62, CyS77 |
| Apigenin | | |
| Methionine sulfoximine | | |
| Amygdalin | Pro90, Arg131 | |
| Coumarin | Pro90 | Trp62 |
| Indirubin | | |
| Isoquinolone | | |
| Kaempferol | | |
| Calcitriol | | |
| Ferulic | | |
| Enalapril | | |
| Artemisinin | Arg67 | |
| Phytic | | |

## Slide 2
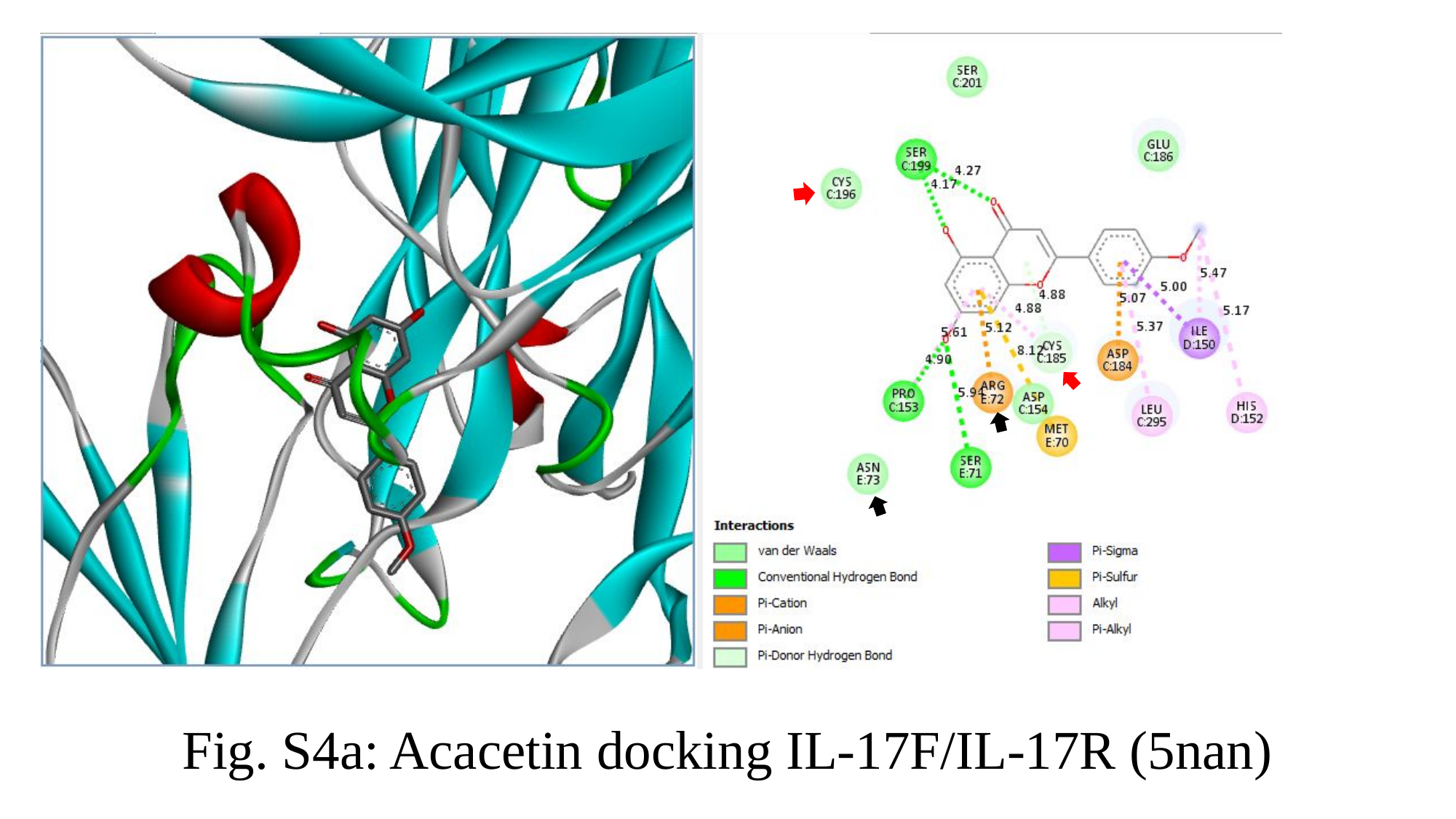

Fig. S4a: Acacetin docking IL-17F/IL-17R (5nan)

## Slide 3
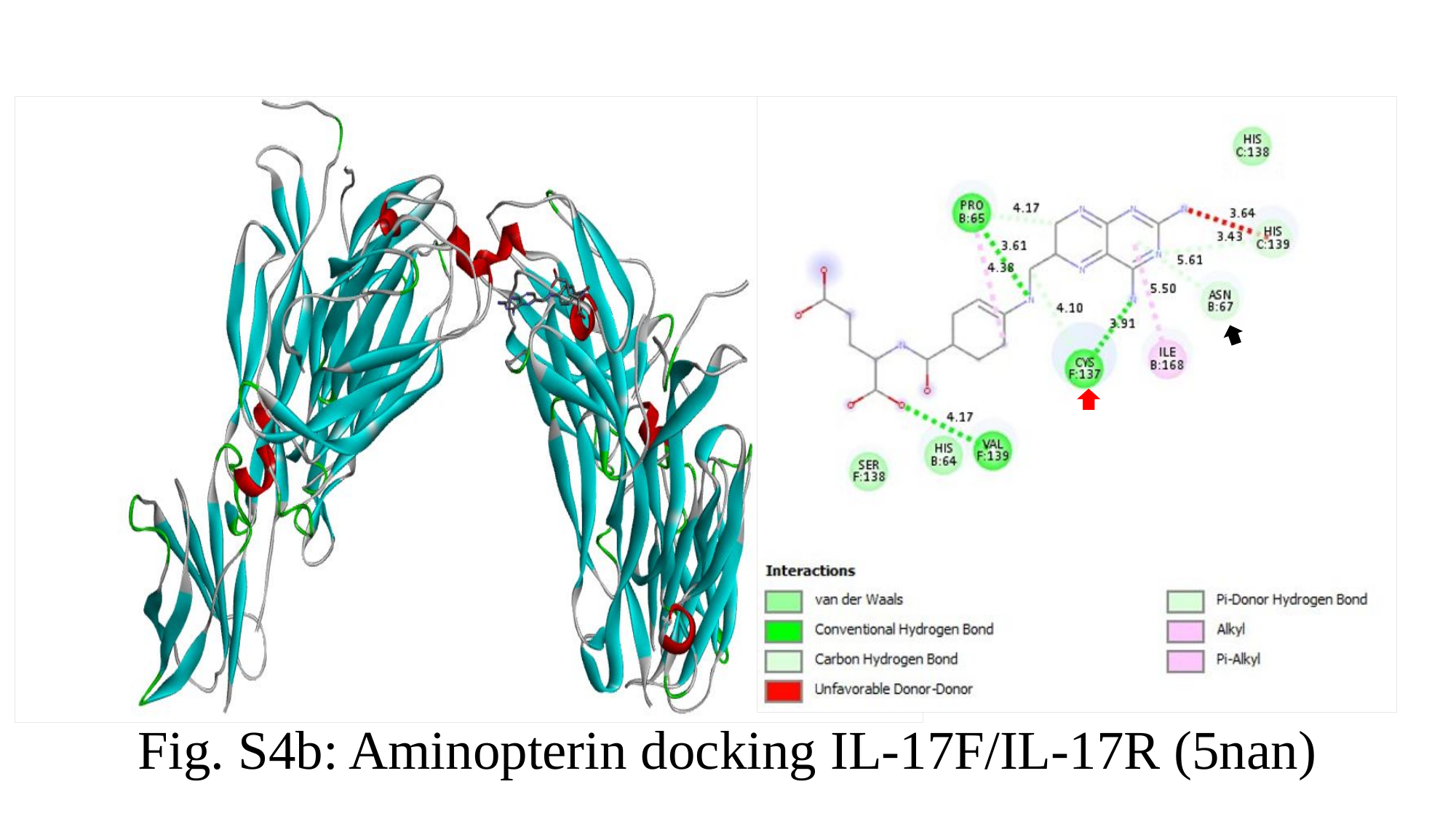

Fig. S4b: Aminopterin docking IL-17F/IL-17R (5nan)

## Slide 4
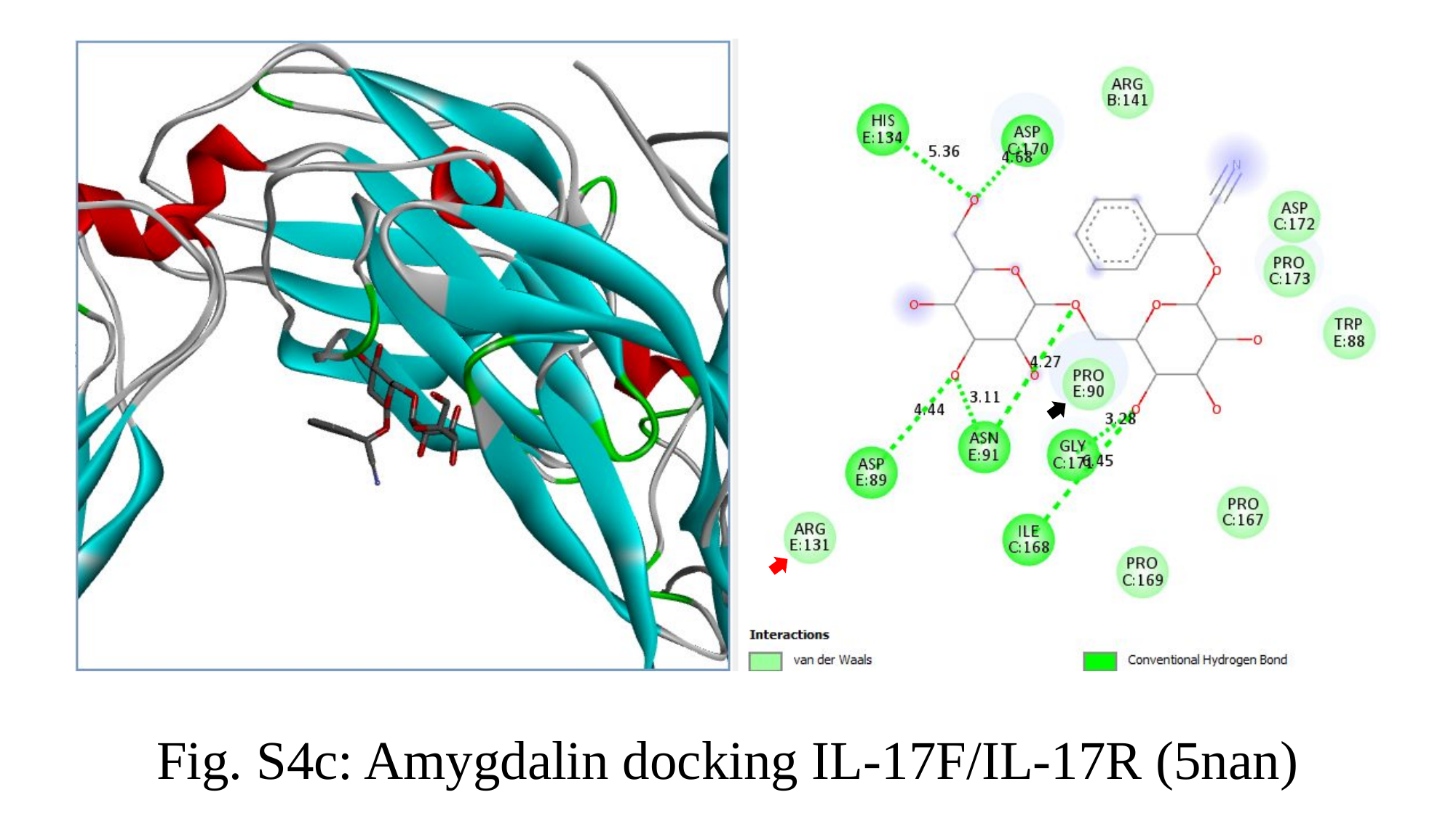

Fig. S4c: Amygdalin docking IL-17F/IL-17R (5nan)

## Slide 5
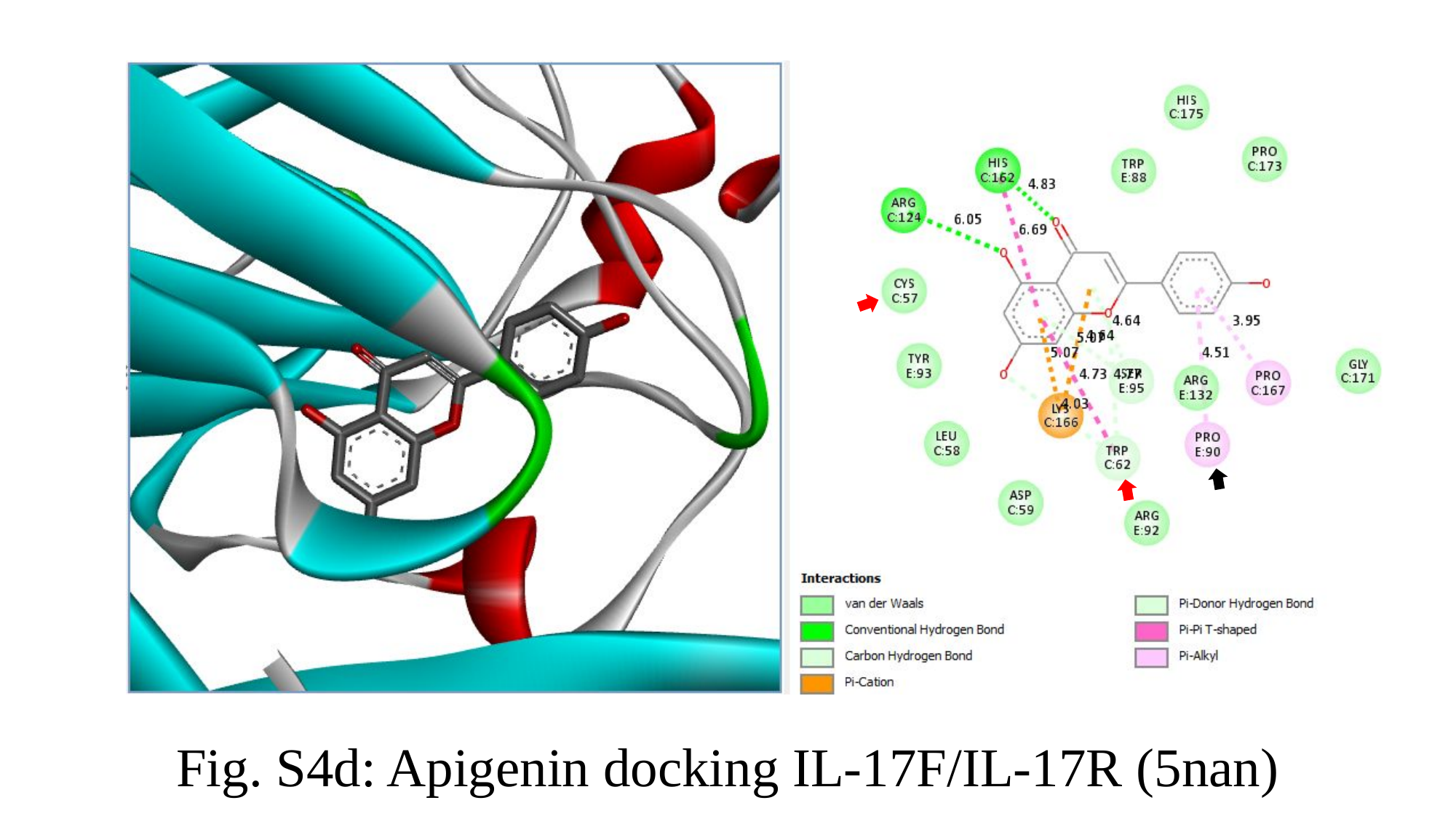

Fig. S4d: Apigenin docking IL-17F/IL-17R (5nan)

## Slide 6
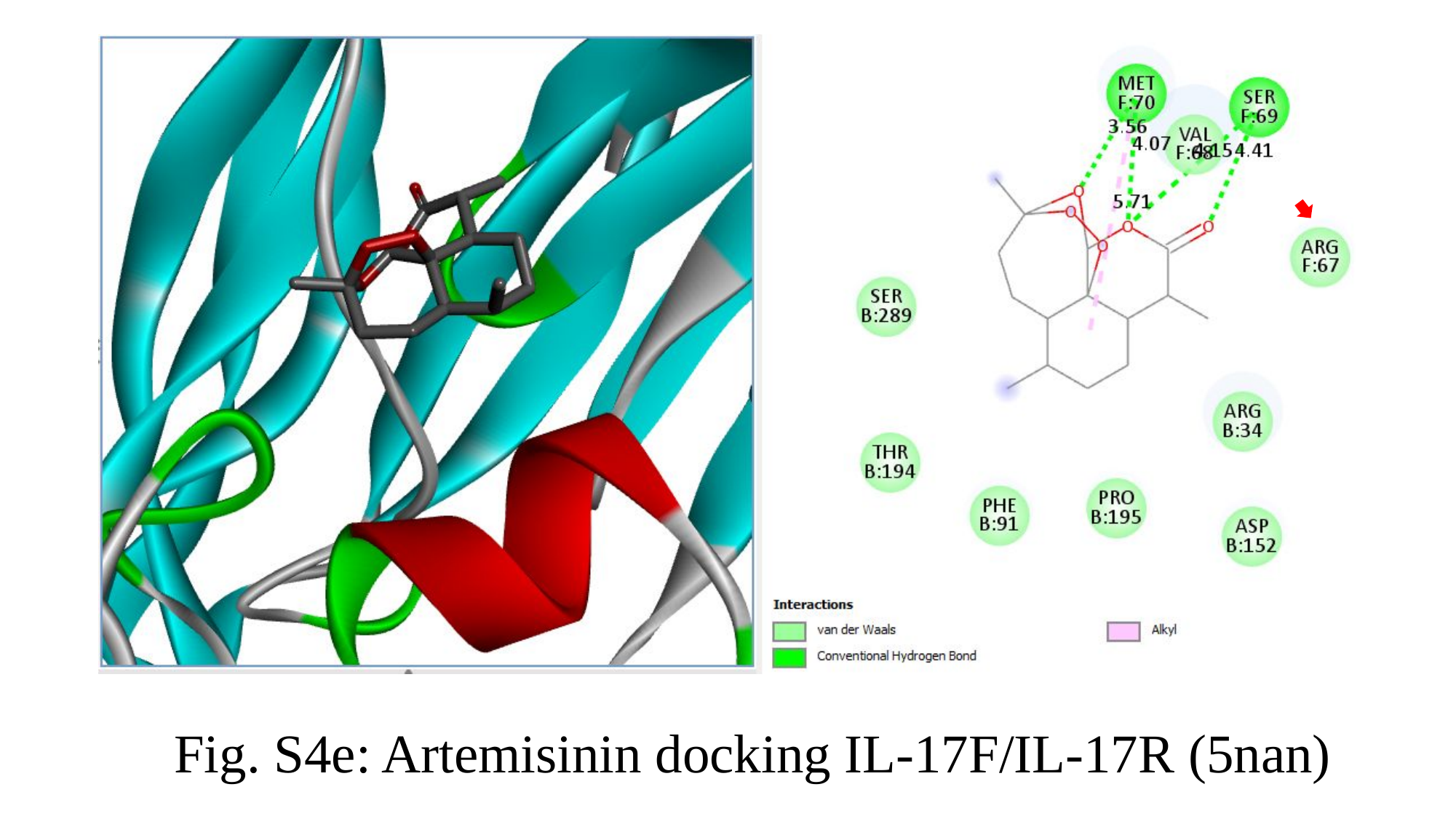

Fig. S4e: Artemisinin docking IL-17F/IL-17R (5nan)

## Slide 7
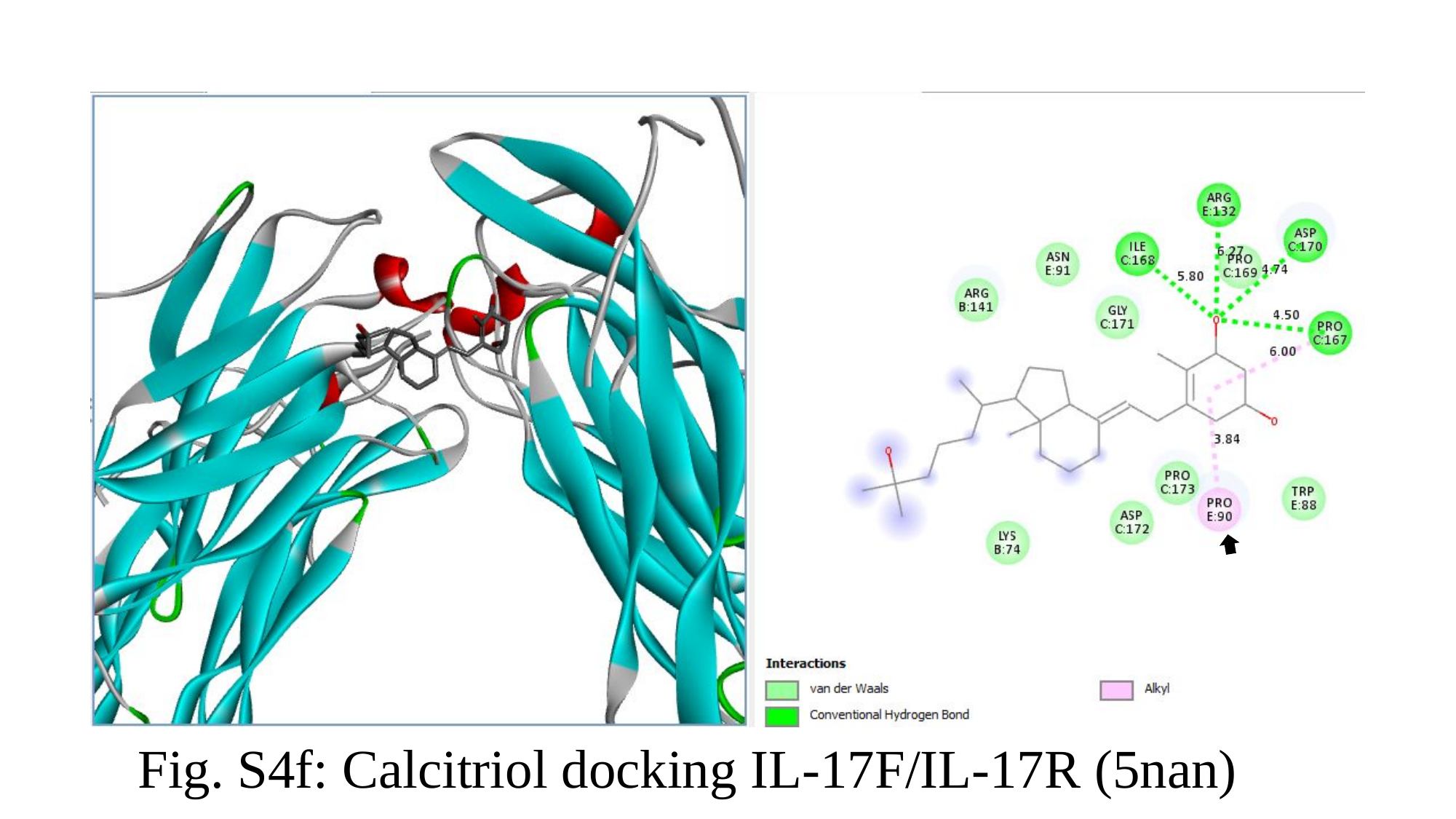

Fig. S4f: Calcitriol docking IL-17F/IL-17R (5nan)

## Slide 8
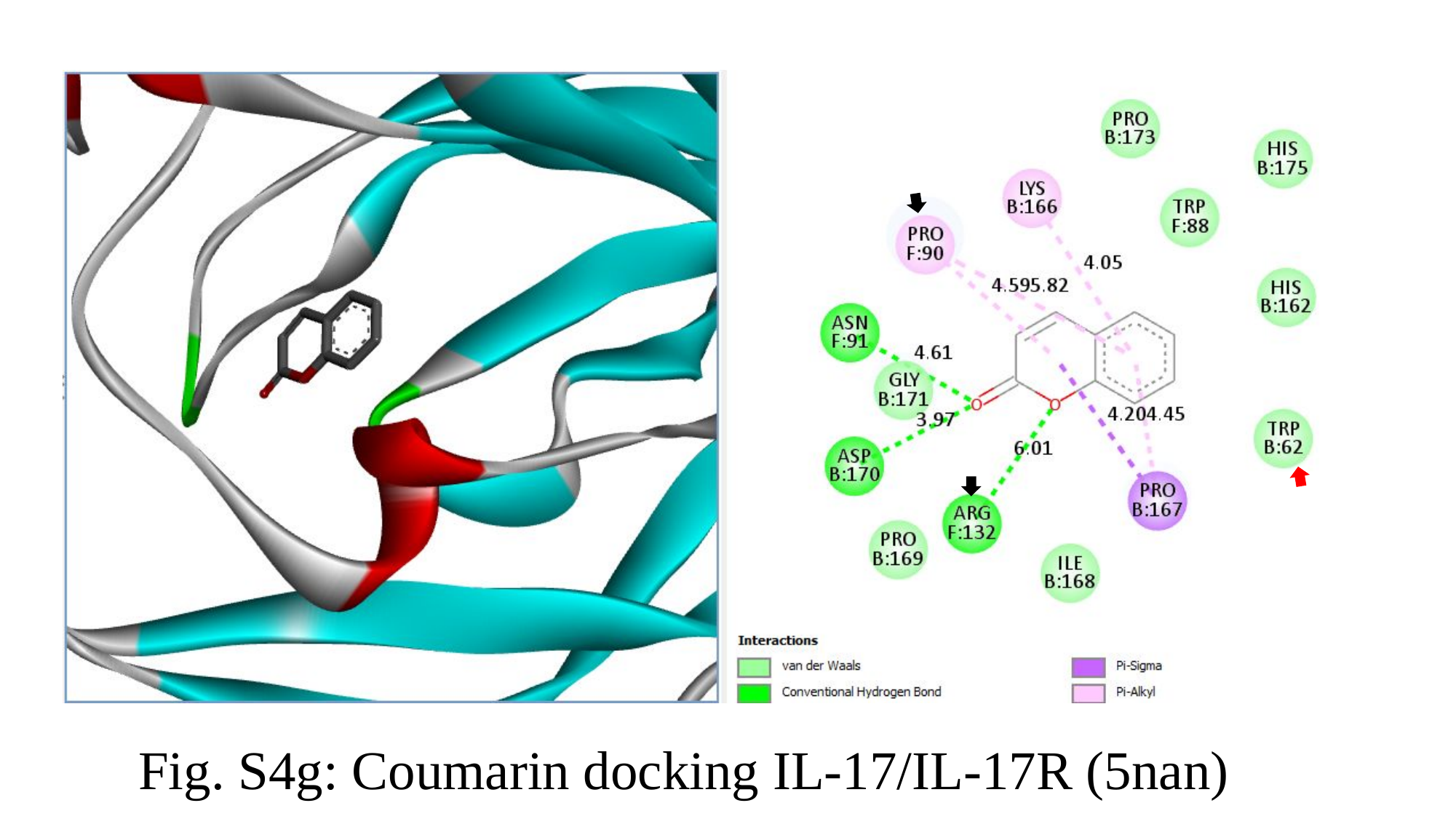

Fig. S4g: Coumarin docking IL-17/IL-17R (5nan)

## Slide 9
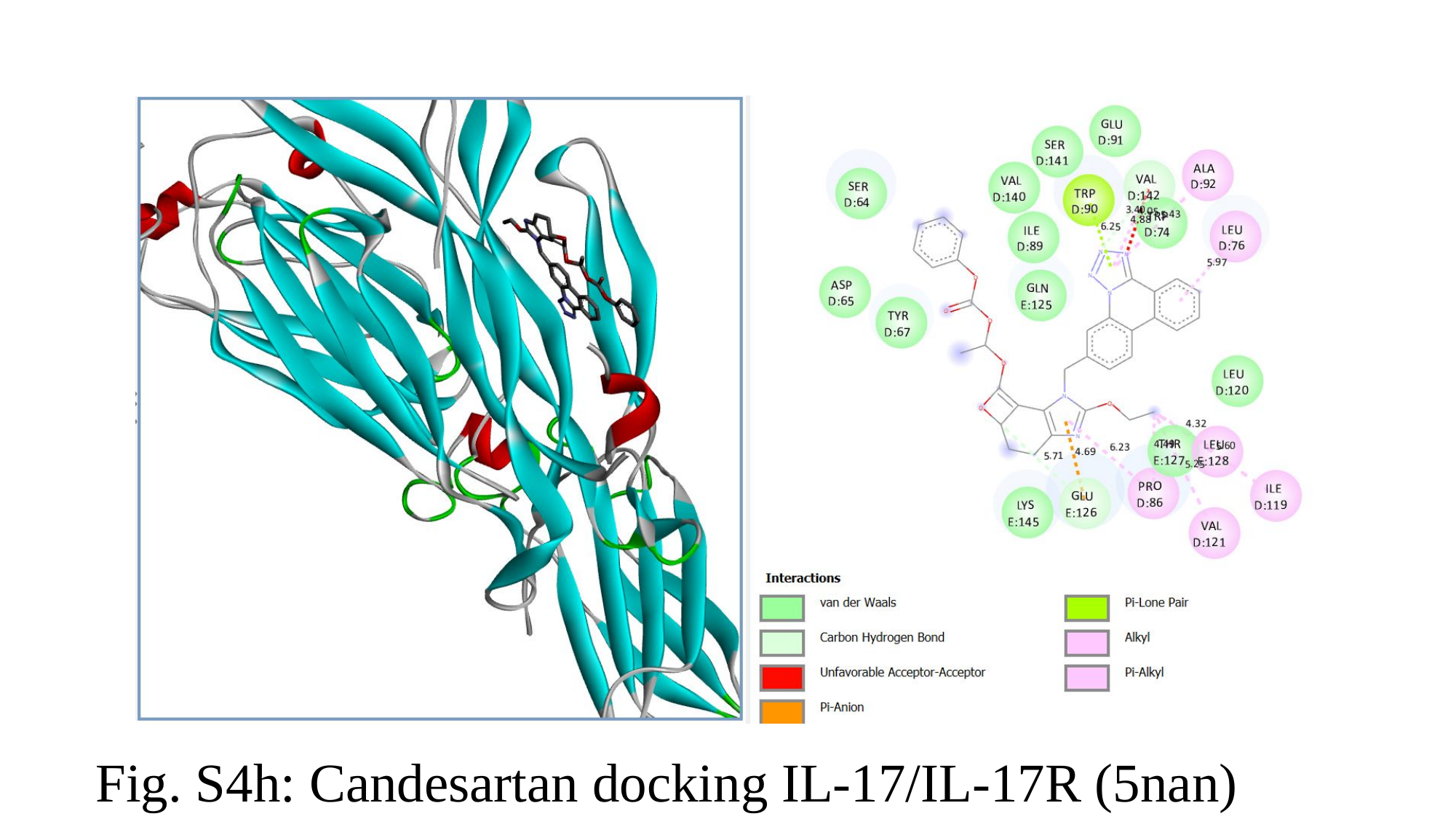

Fig. S4h: Candesartan docking IL-17/IL-17R (5nan)

## Slide 10
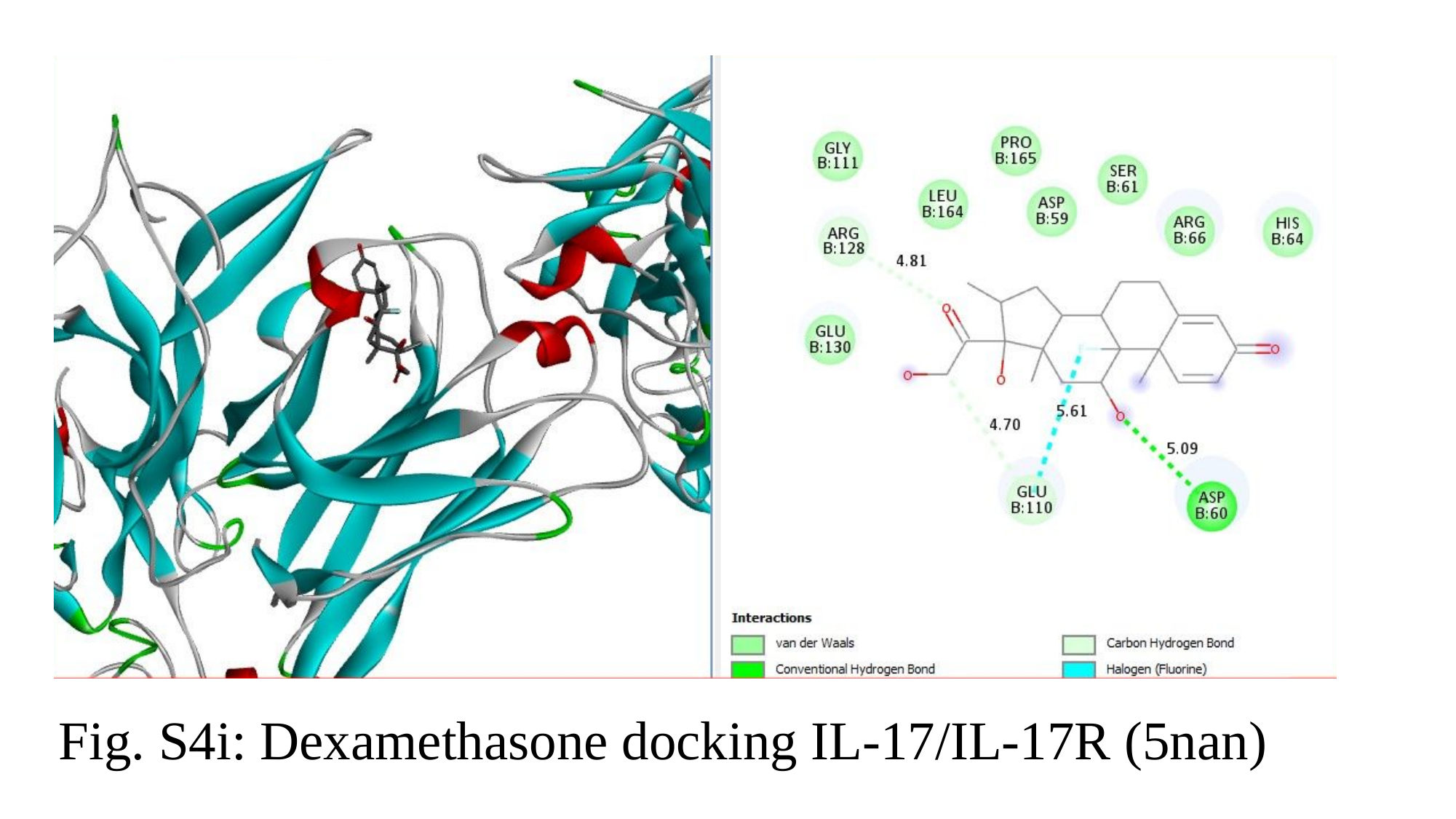

Fig. S4i: Dexamethasone docking IL-17/IL-17R (5nan)

## Slide 11
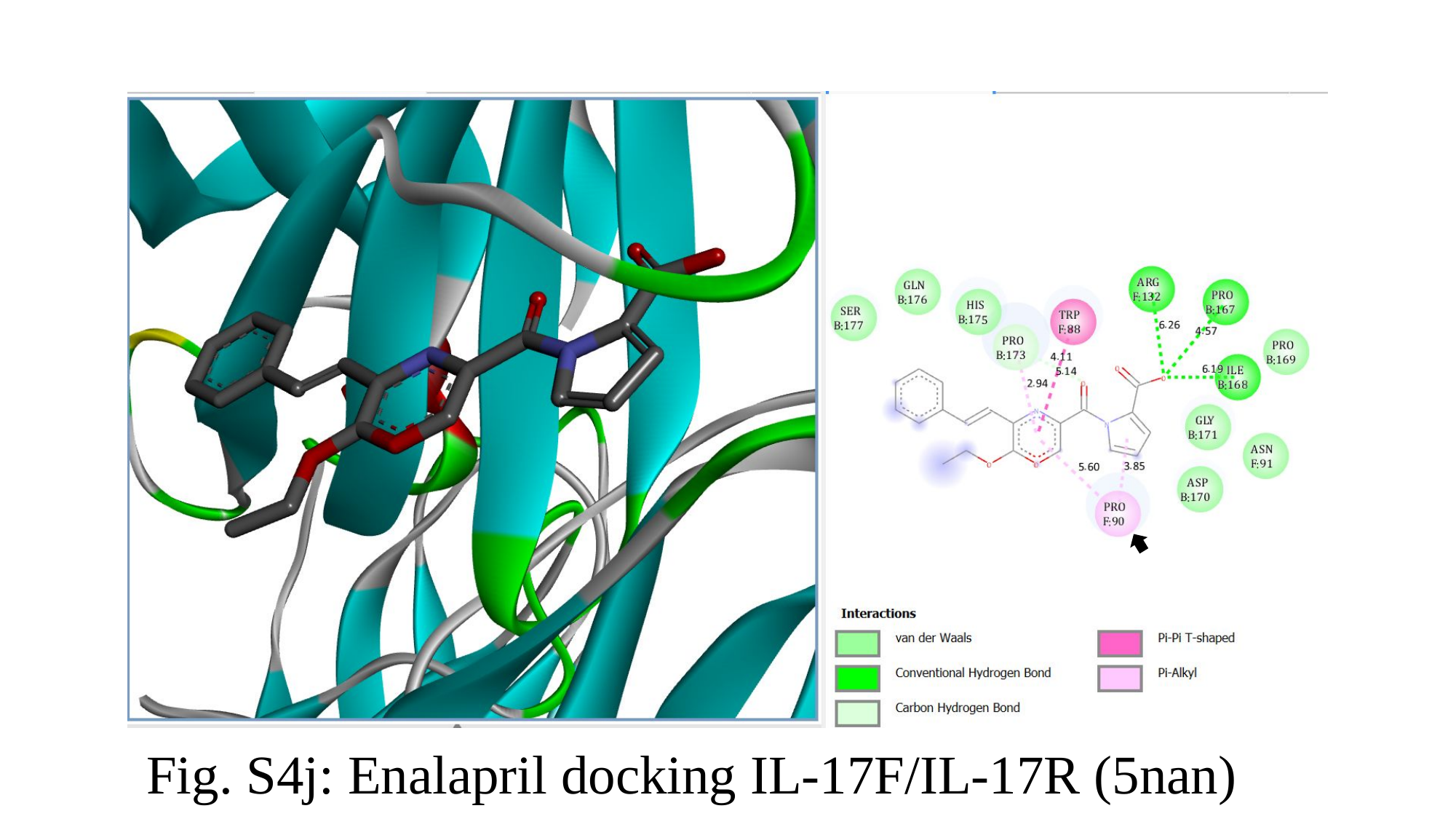

Fig. S4j: Enalapril docking IL-17F/IL-17R (5nan)

## Slide 12
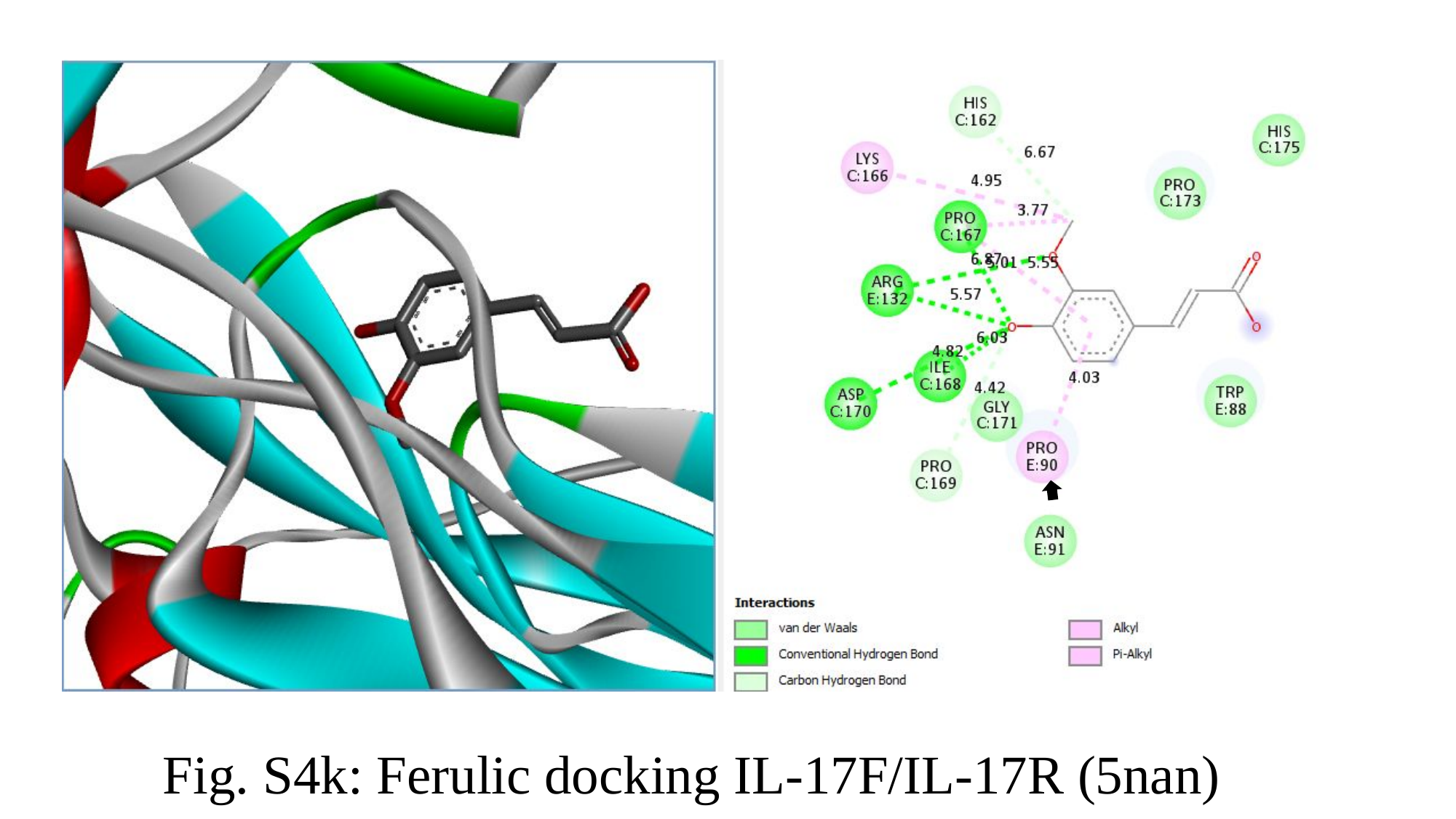

Fig. S4k: Ferulic docking IL-17F/IL-17R (5nan)

## Slide 13
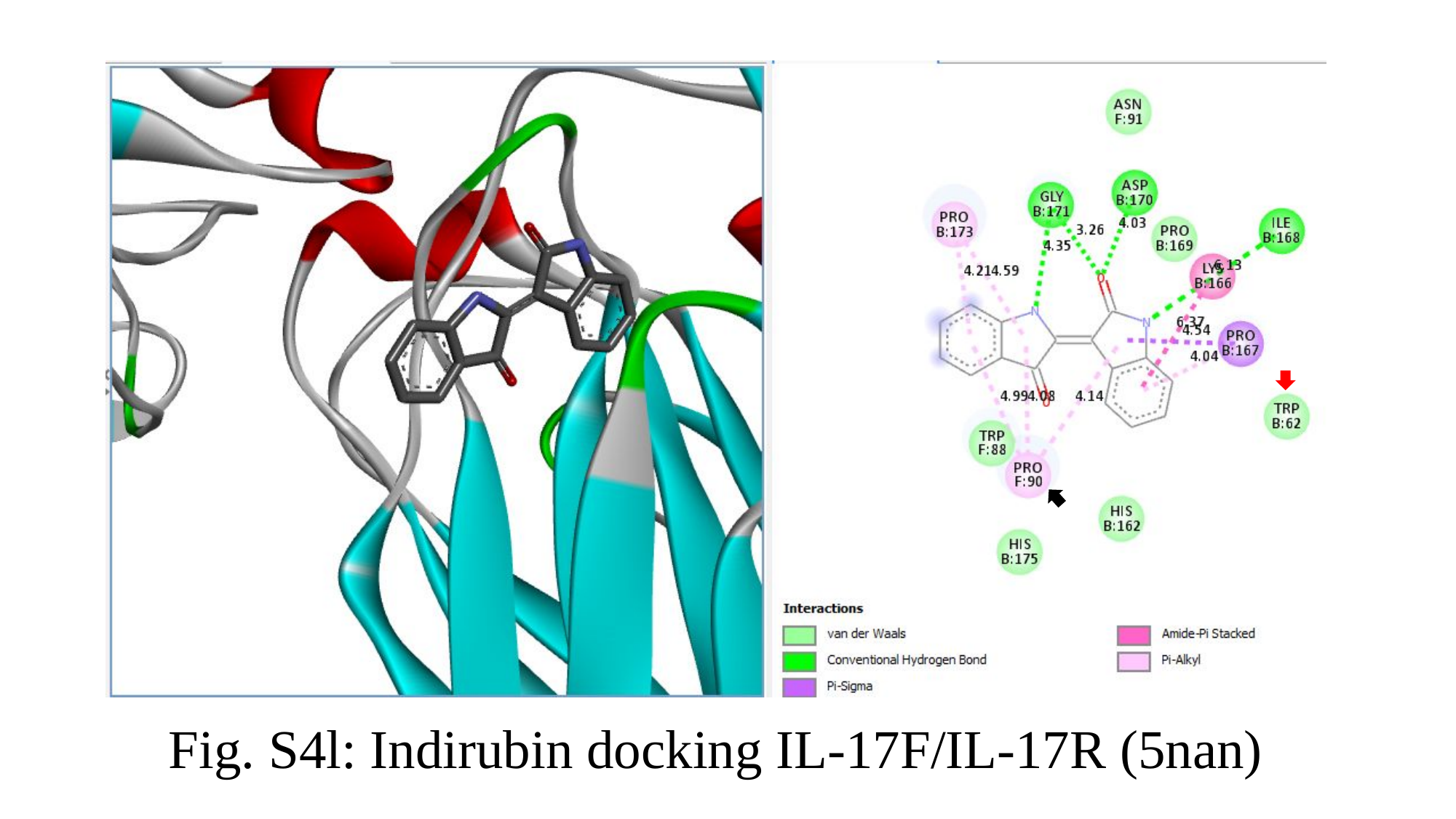

Fig. S4l: Indirubin docking IL-17F/IL-17R (5nan)

## Slide 14
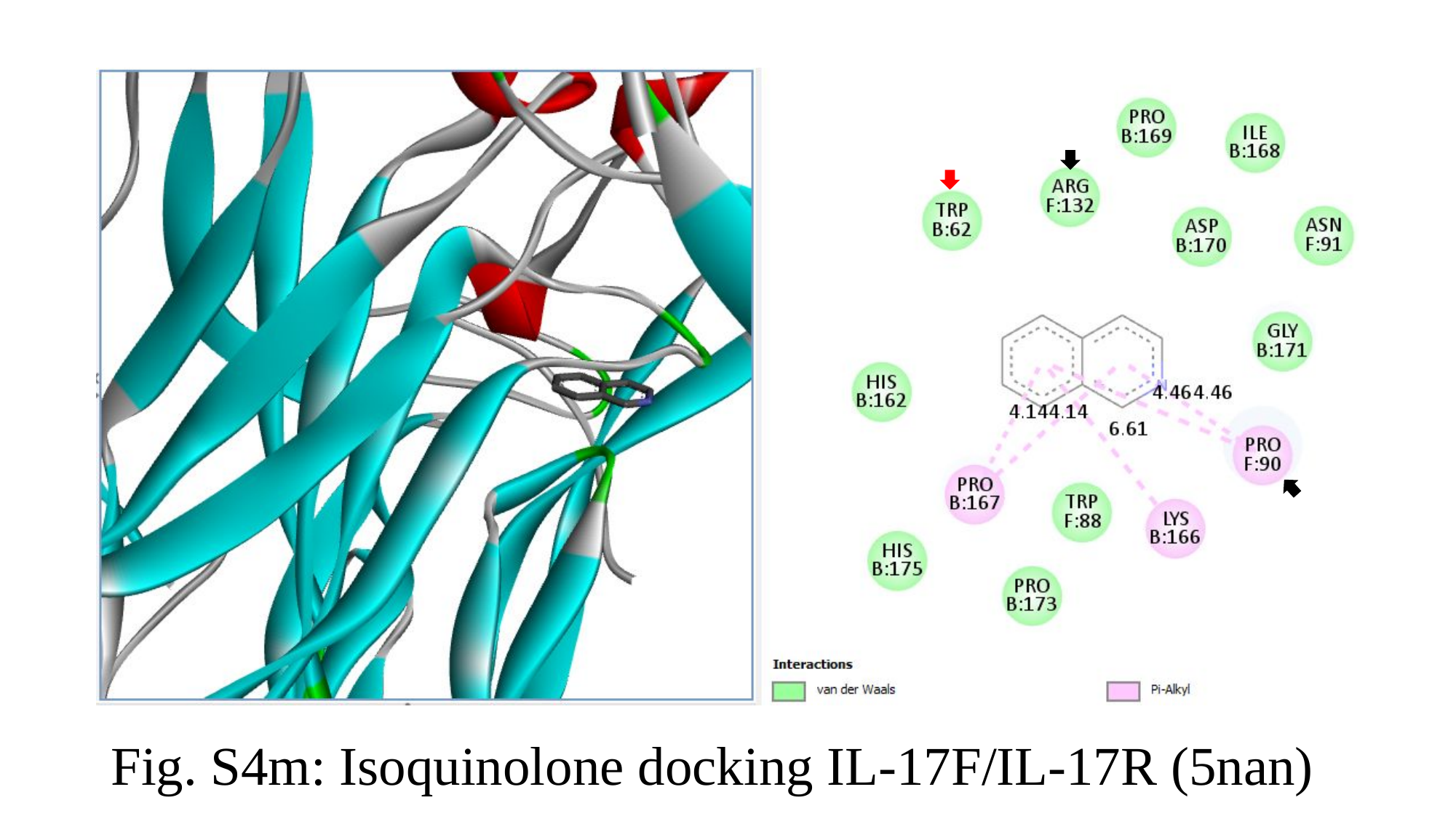

Fig. S4m: Isoquinolone docking IL-17F/IL-17R (5nan)

## Slide 15
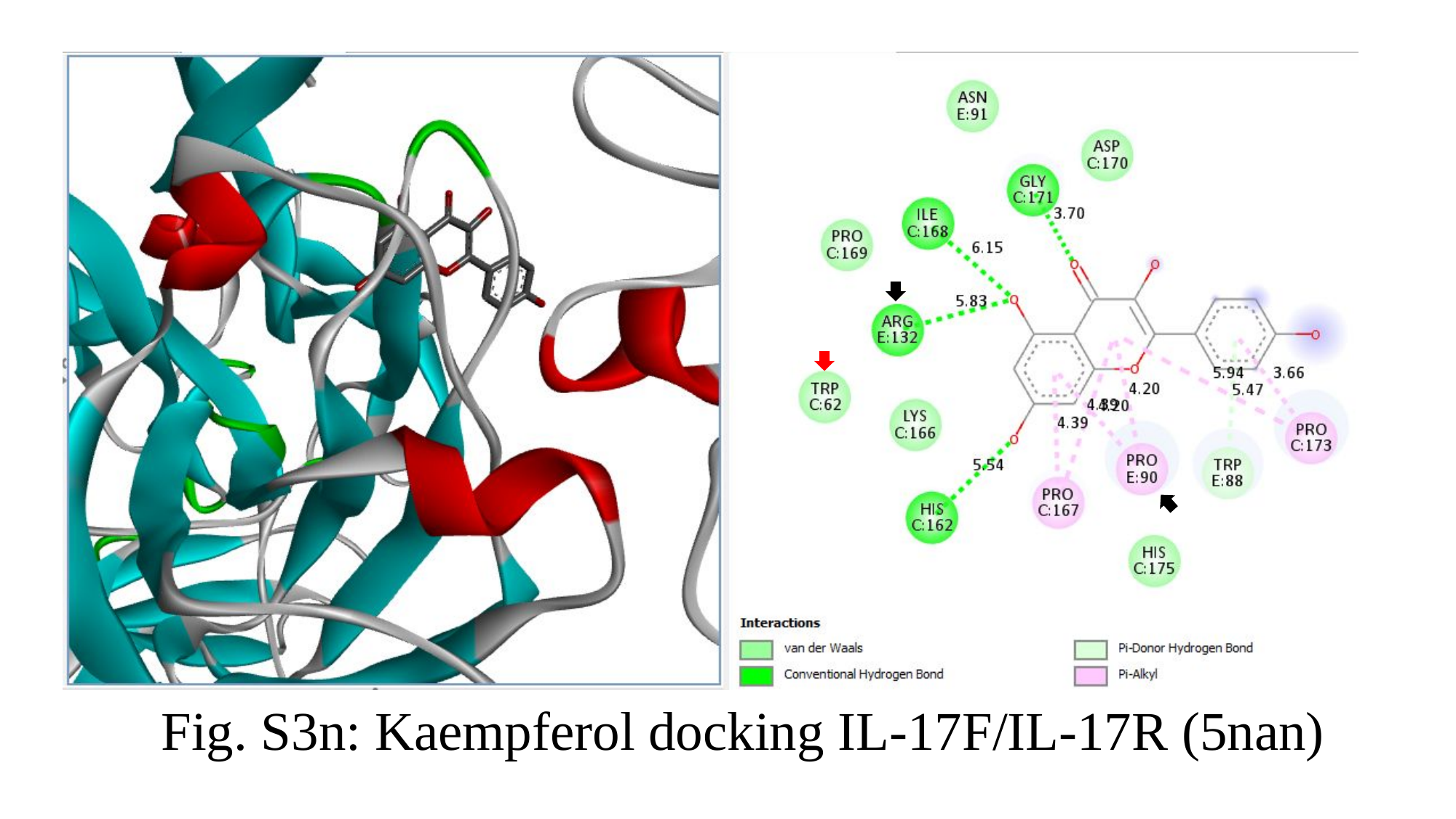

Fig. S3n: Kaempferol docking IL-17F/IL-17R (5nan)

## Slide 16
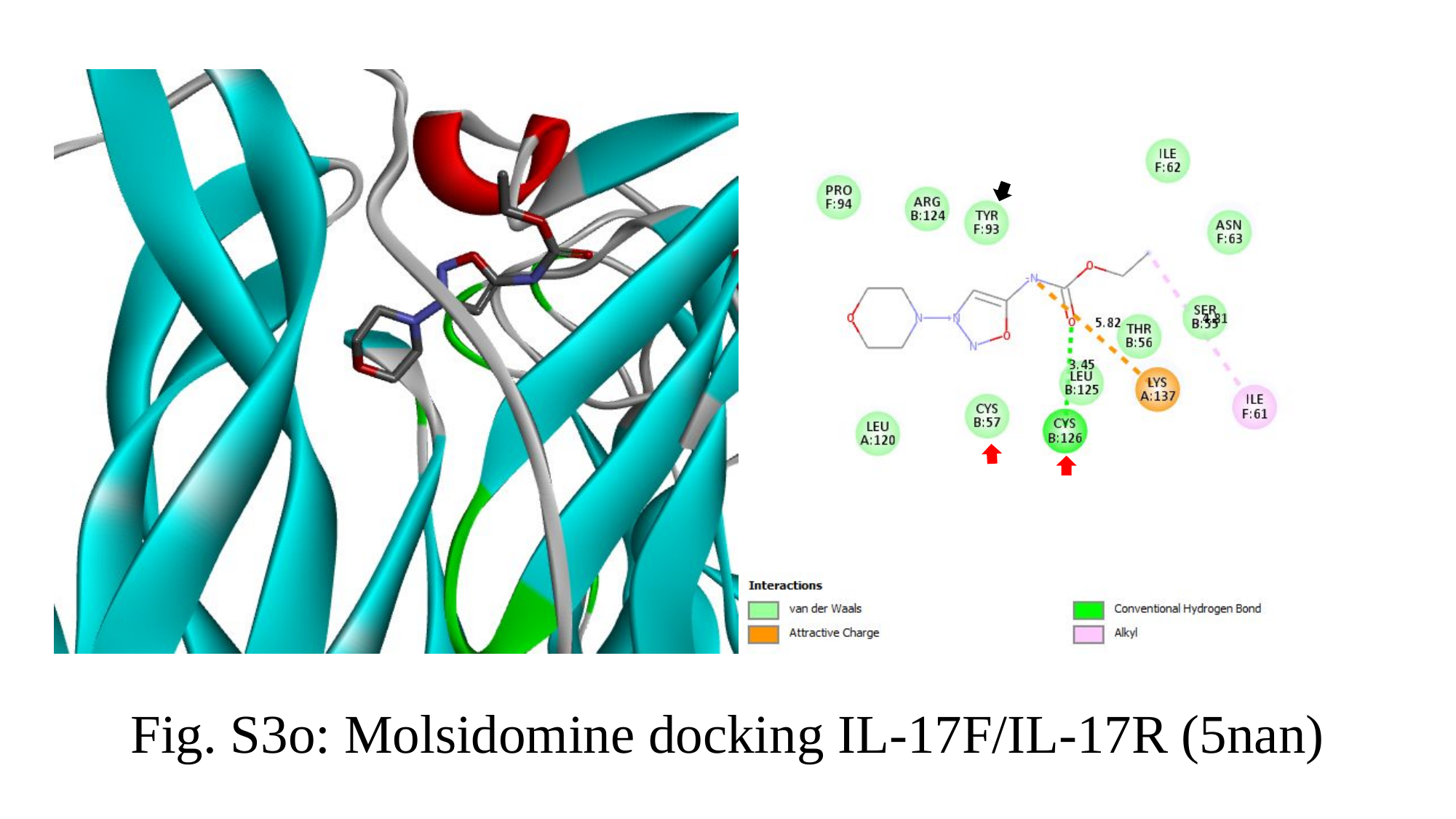

Fig. S3o: Molsidomine docking IL-17F/IL-17R (5nan)

## Slide 17
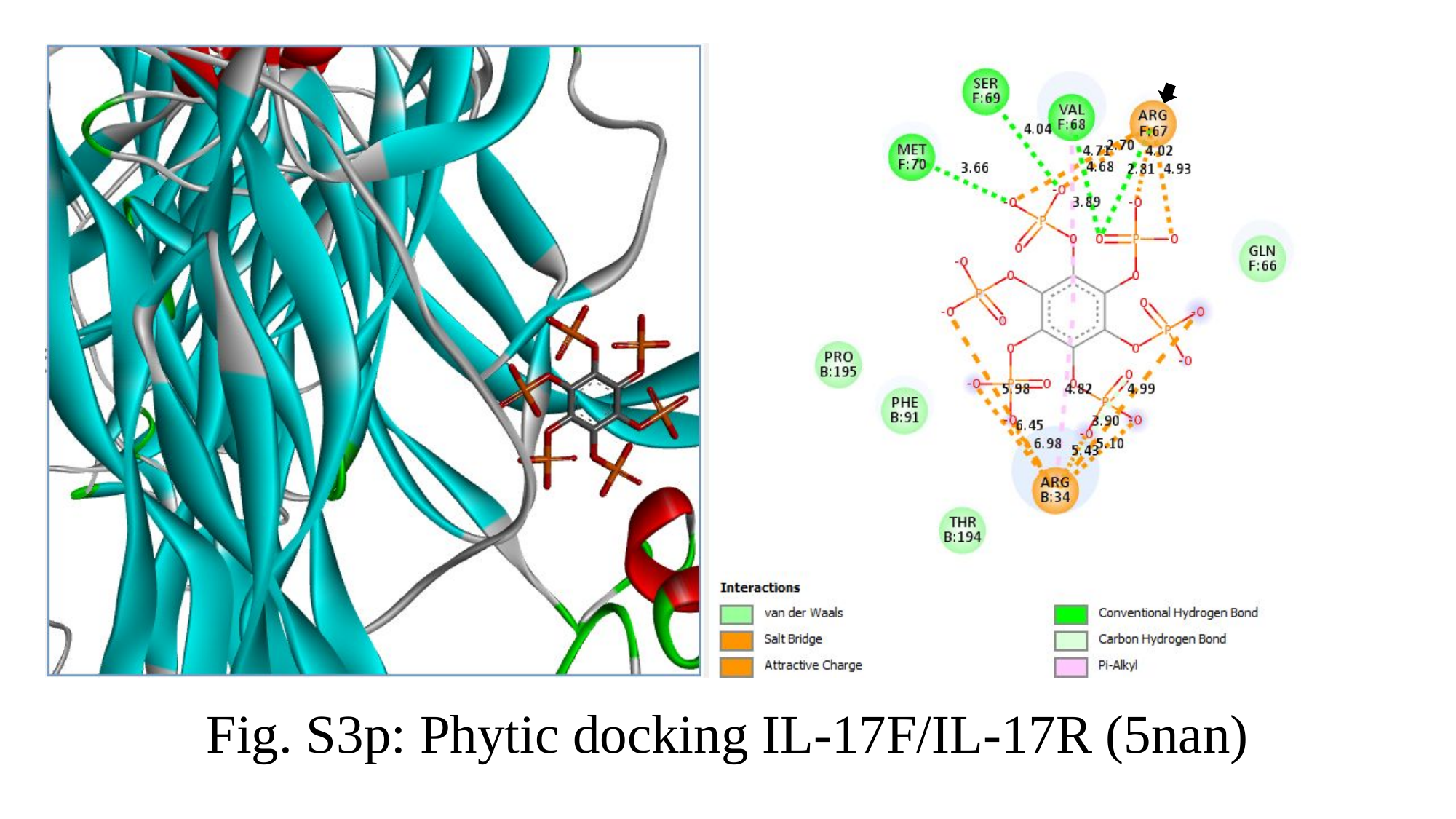

Fig. S3p: Phytic docking IL-17F/IL-17R (5nan)

## Slide 18
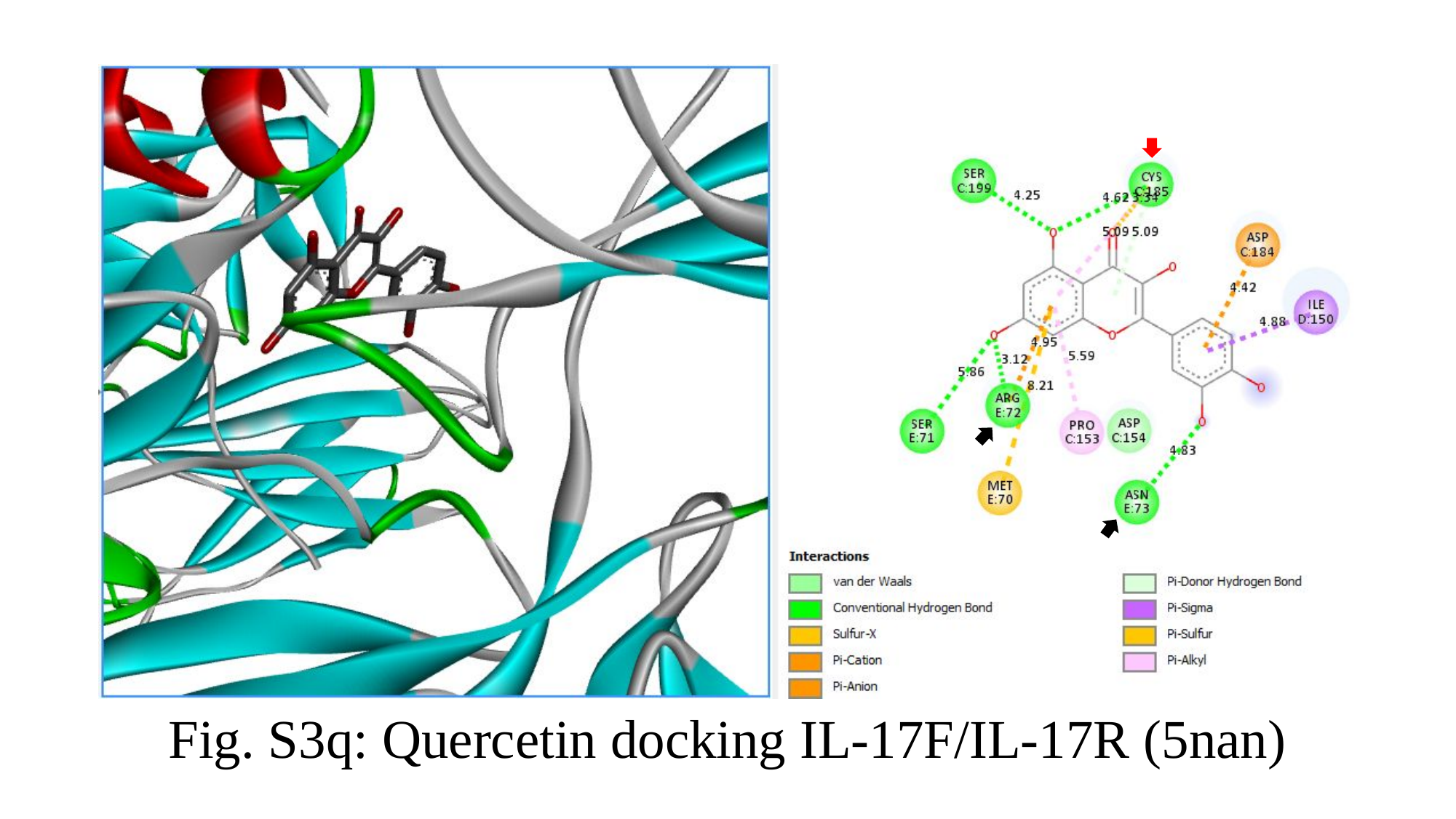

Fig. S3q: Quercetin docking IL-17F/IL-17R (5nan)

## Slide 19
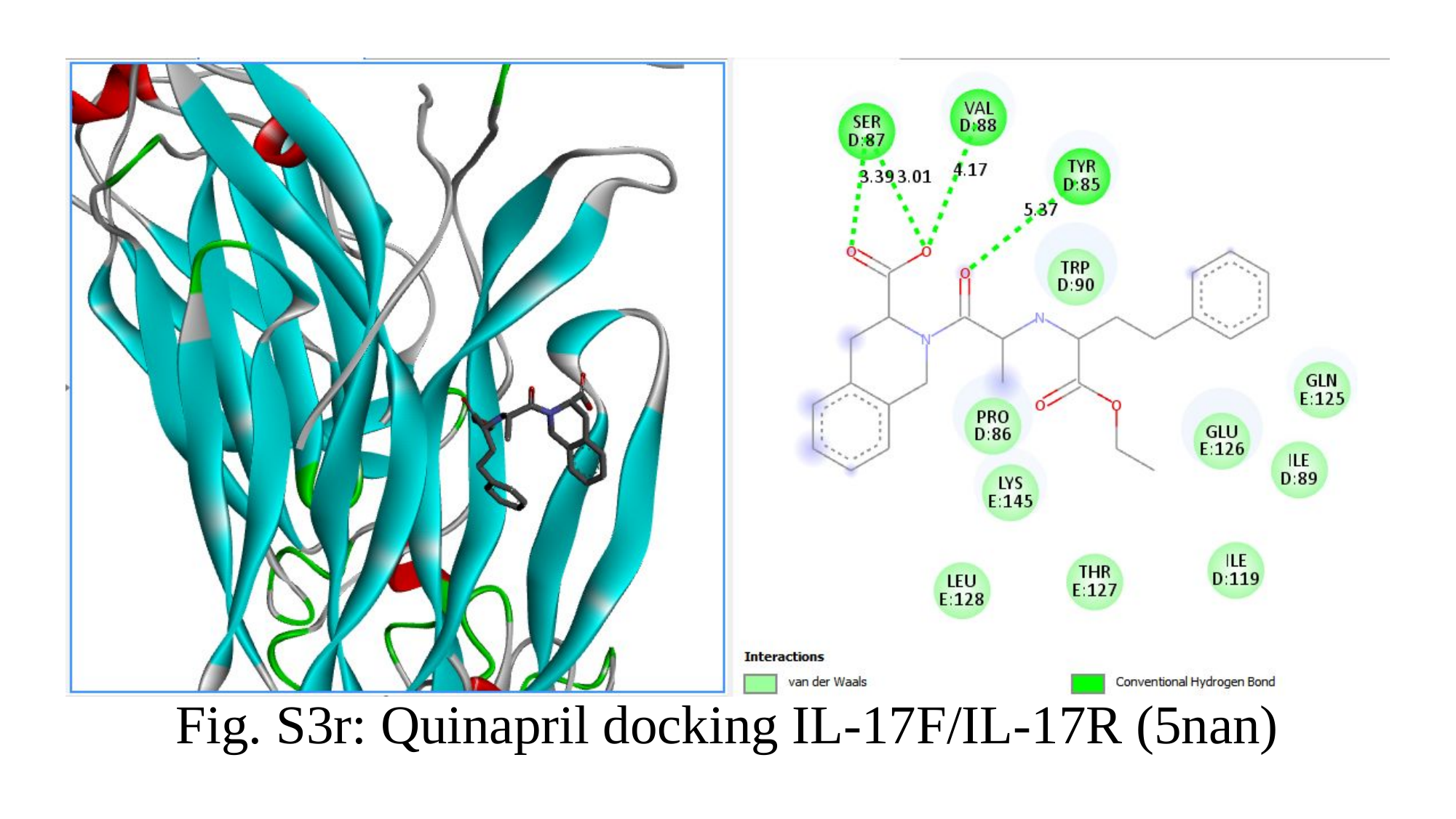

Fig. S3r: Quinapril docking IL-17F/IL-17R (5nan)

## Slide 20
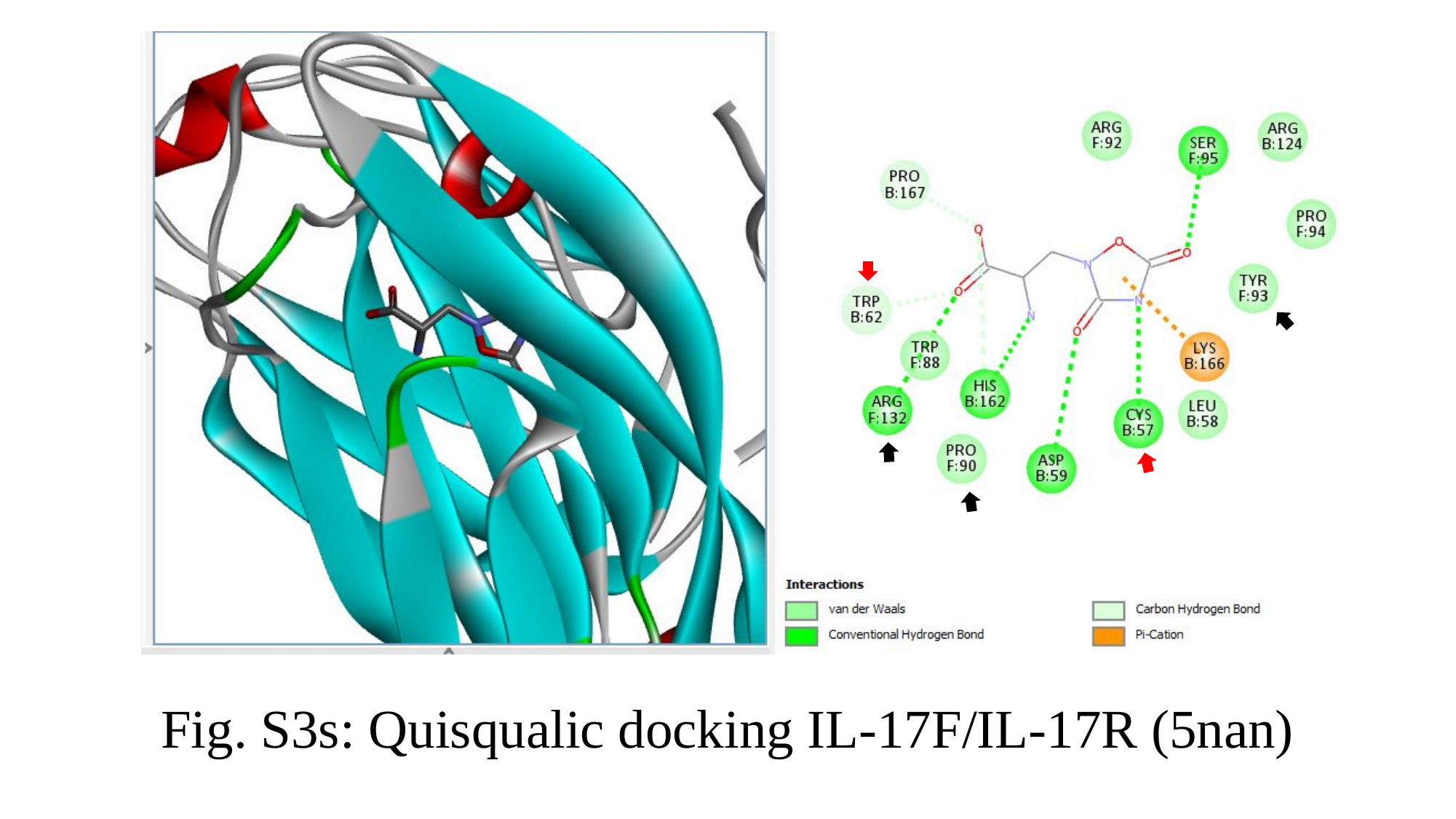

Fig. S3s: Quisqualic docking IL-17F/IL-17R (5nan)

## Slide 21
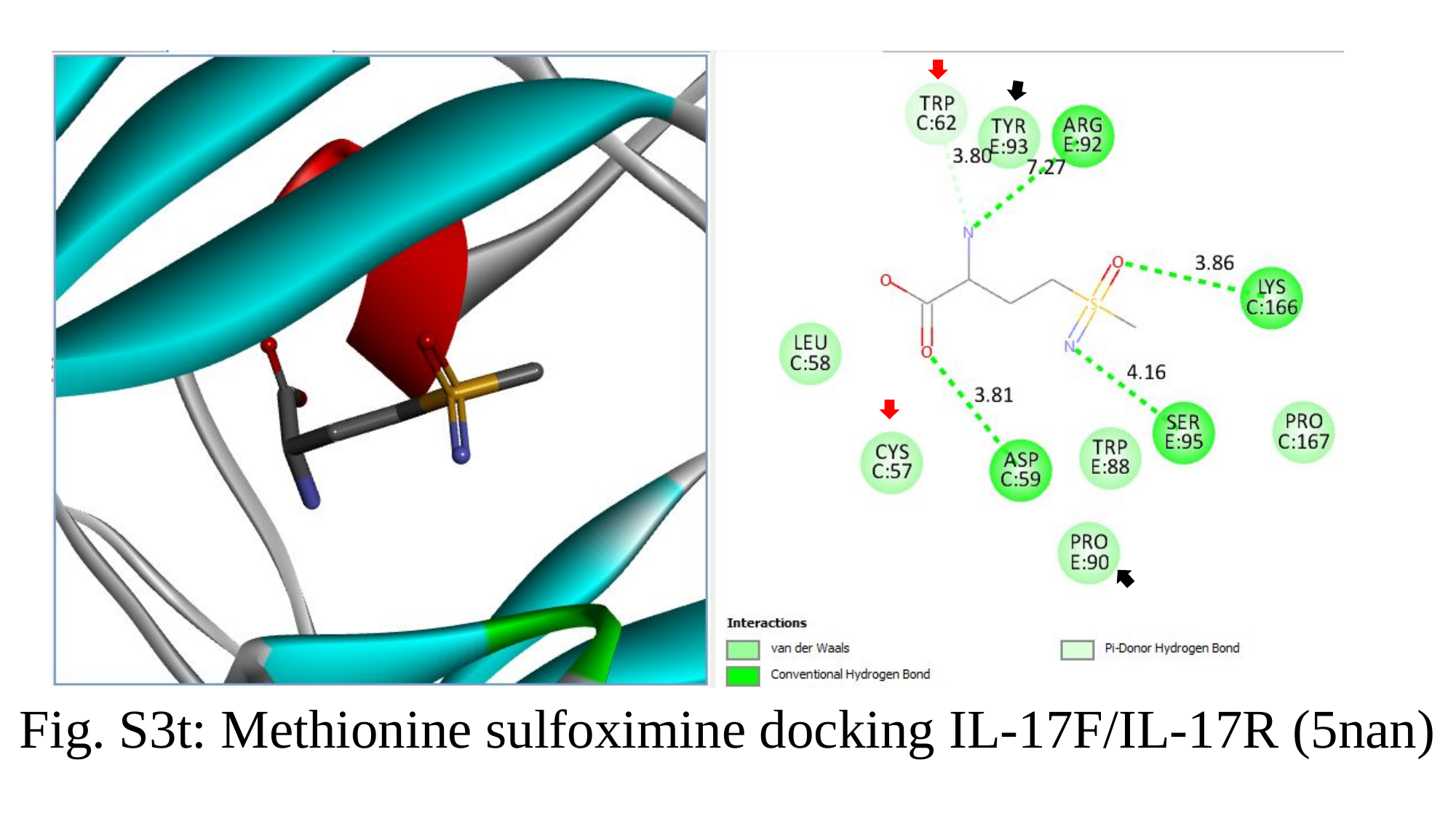

Fig. S3t: Methionine sulfoximine docking IL-17F/IL-17R (5nan)

## Slide 22
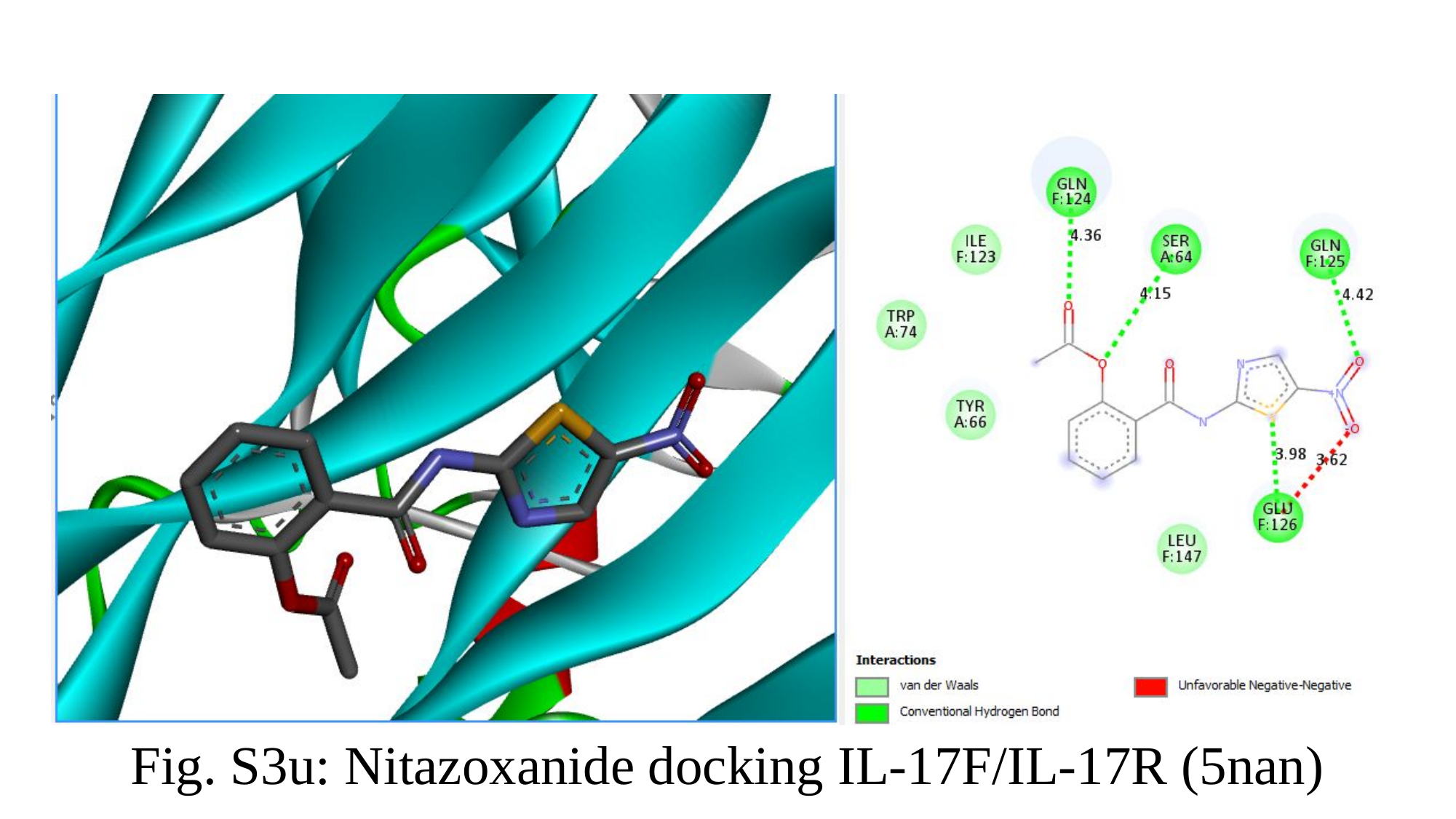

Fig. S3u: Nitazoxanide docking IL-17F/IL-17R (5nan)

## Slide 23
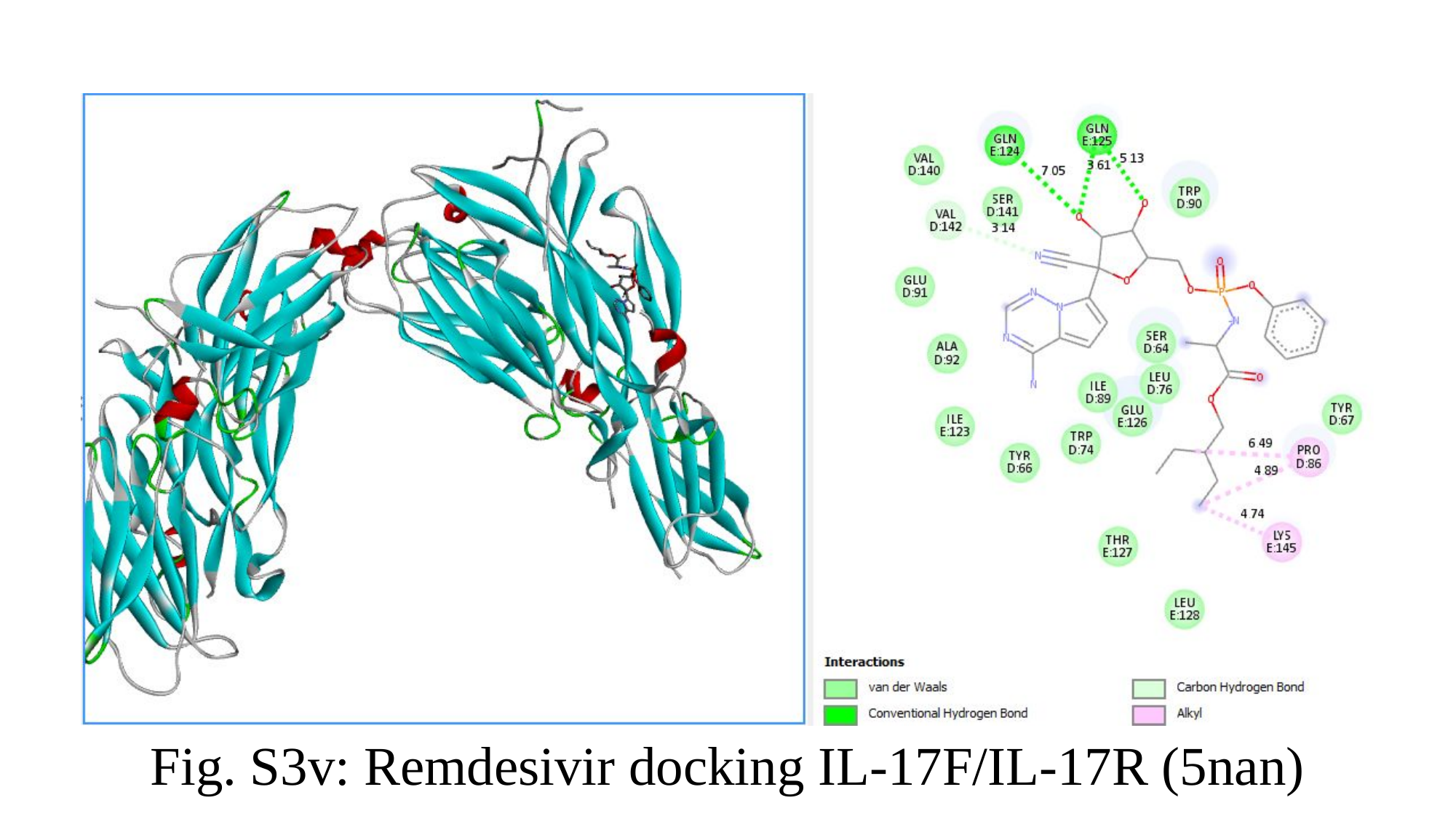

Fig. S3v: Remdesivir docking IL-17F/IL-17R (5nan)

## Slide 24
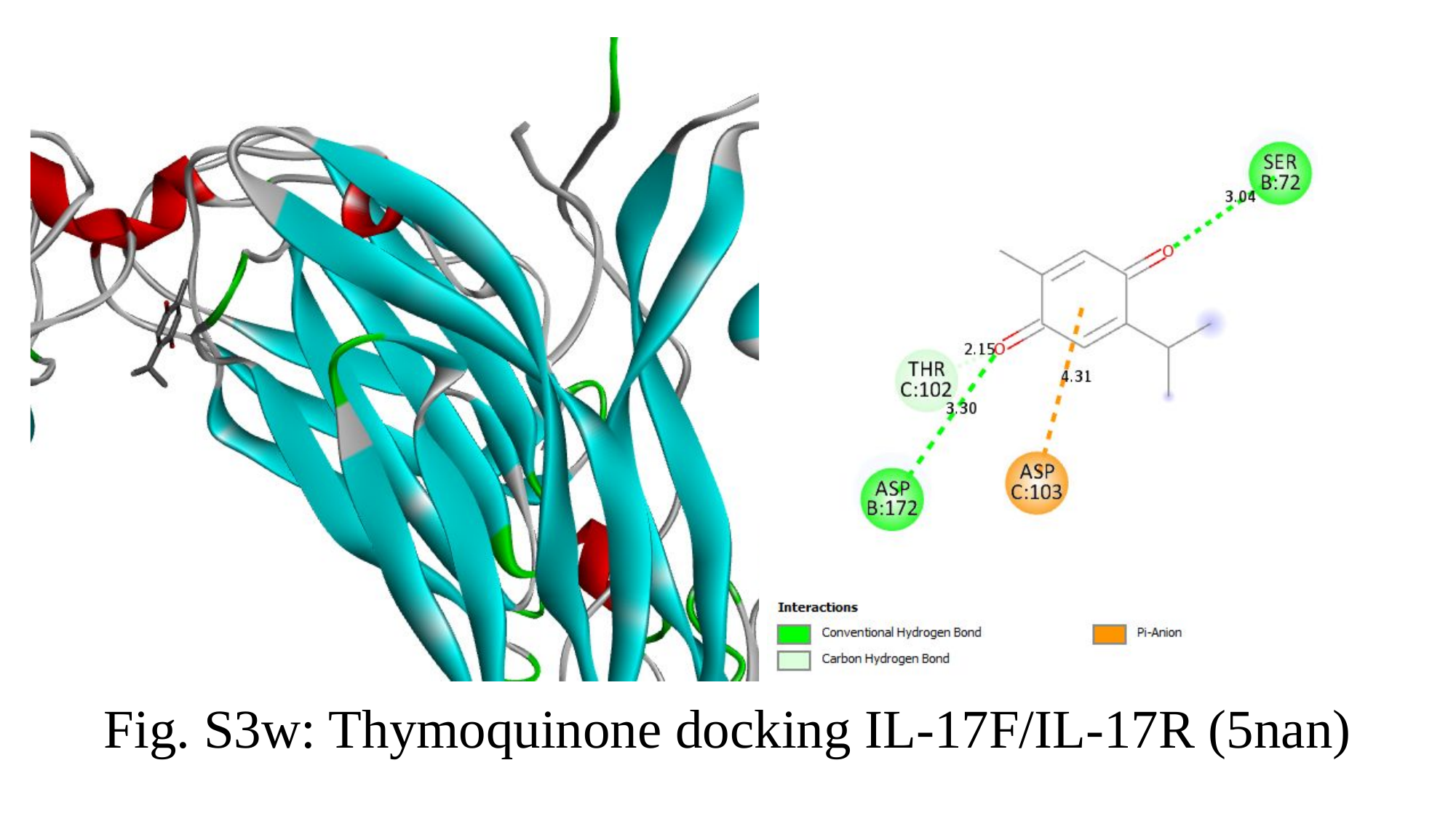

Fig. S3w: Thymoquinone docking IL-17F/IL-17R (5nan)

## Slide 25
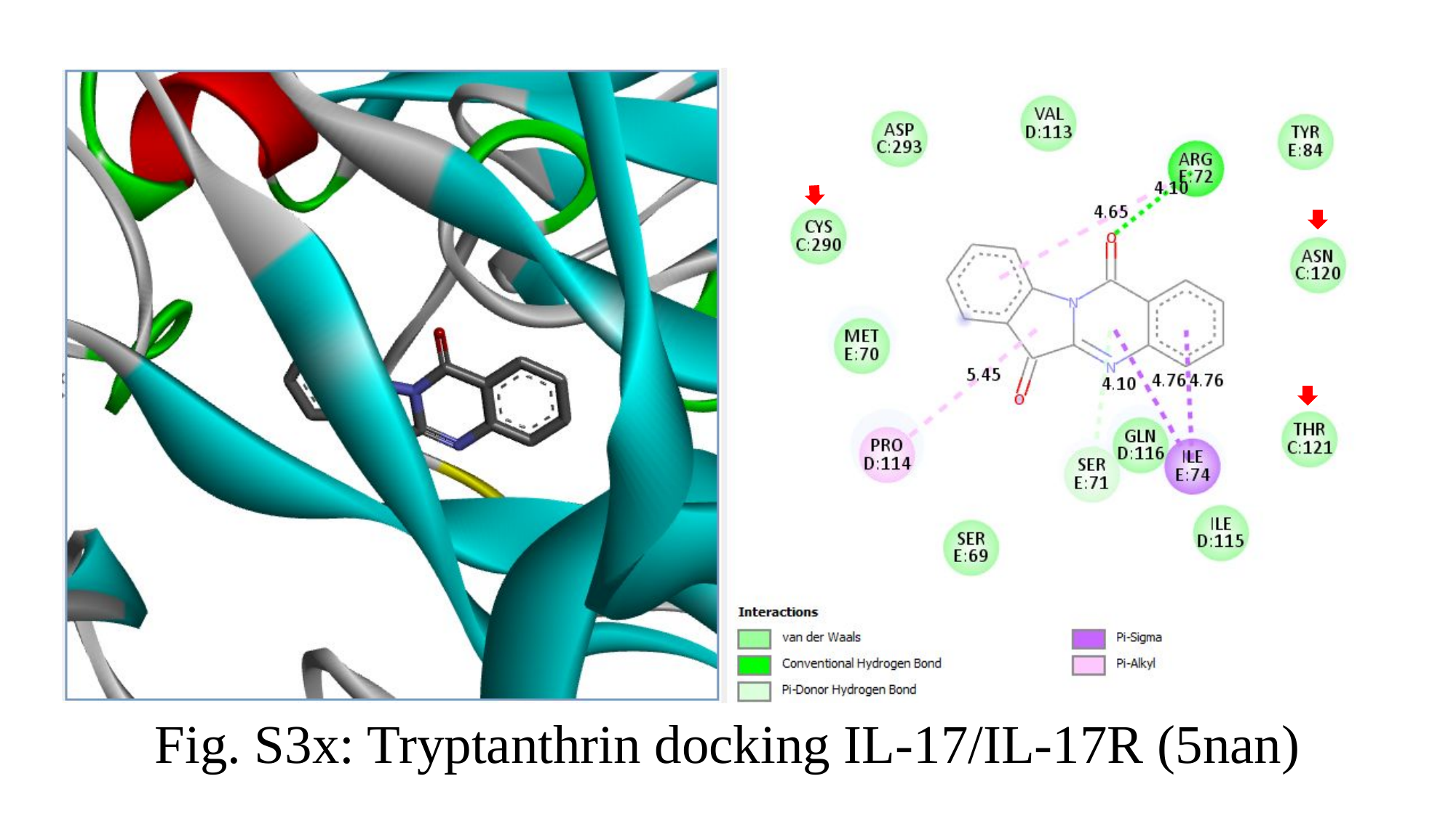

Fig. S3x: Tryptanthrin docking IL-17/IL-17R (5nan)
