# Supplementary material for: Targeting asparagine and cysteine in SARS-CoV-2 variants and human pro-inflammatory mediators to alleviate COVID-19 severity; a cross-section and in-silico study
Source: Sci Rep. 2025 Nov 3;15:38445. doi: 10.1038/s41598-025-19359-y (PMC12583749; doi:10.1038/s41598-025-19359-y)
Supplement: Supplementary file 12 — Supplementary Material 12 [file 41598_2025_19359_MOESM12_ESM.pptx]

## Slide 1
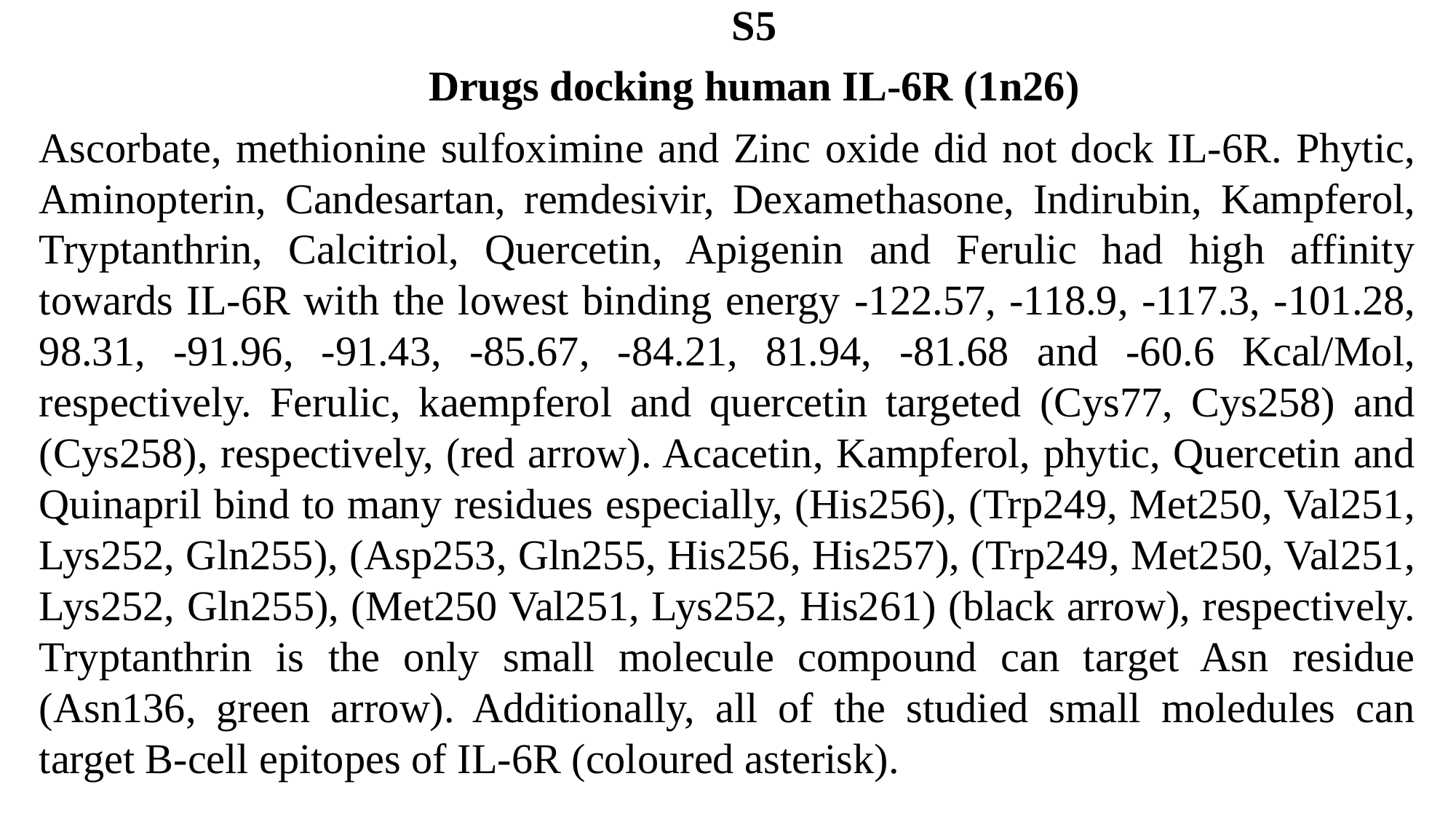

S5
Drugs docking human IL-6R (1n26)
Ascorbate, methionine sulfoximine and Zinc oxide did not dock IL-6R. Phytic, Aminopterin, Candesartan, remdesivir, Dexamethasone, Indirubin, Kampferol, Tryptanthrin, Calcitriol, Quercetin, Apigenin and Ferulic had high affinity towards IL-6R with the lowest binding energy -122.57, -118.9, -117.3, -101.28, 98.31, -91.96, -91.43, -85.67, -84.21, 81.94, -81.68 and -60.6 Kcal/Mol, respectively. Ferulic, kaempferol and quercetin targeted (Cys77, Cys258) and (Cys258), respectively, (red arrow). Acacetin, Kampferol, phytic, Quercetin and Quinapril bind to many residues especially, (His256), (Trp249, Met250, Val251, Lys252, Gln255), (Asp253, Gln255, His256, His257), (Trp249, Met250, Val251, Lys252, Gln255), (Met250 Val251, Lys252, His261) (black arrow), respectively. Tryptanthrin is the only small molecule compound can target Asn residue (Asn136, green arrow). Additionally, all of the studied small moledules can target B-cell epitopes of IL-6R (coloured asterisk).

## Slide 2
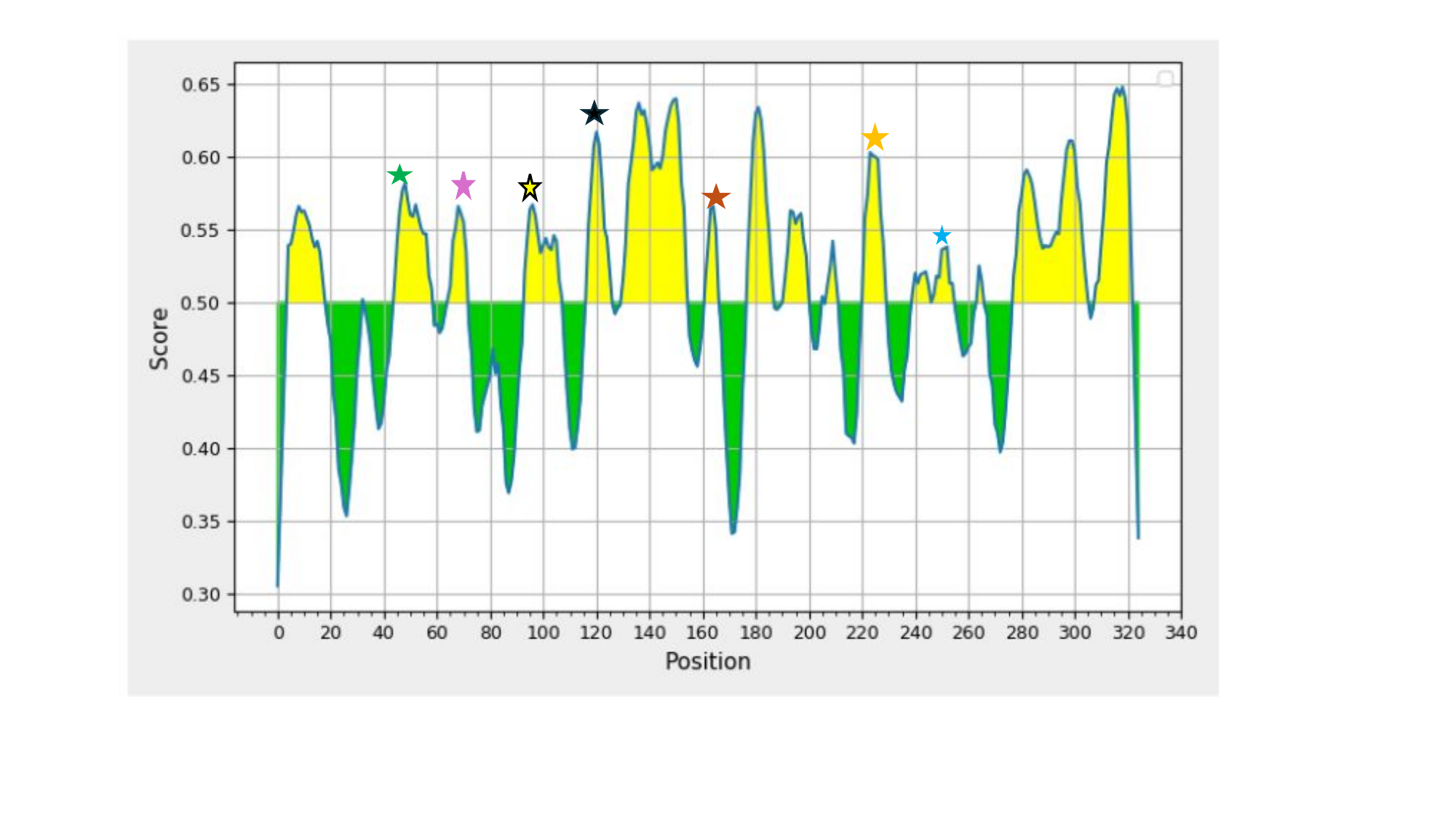

## Slide 3
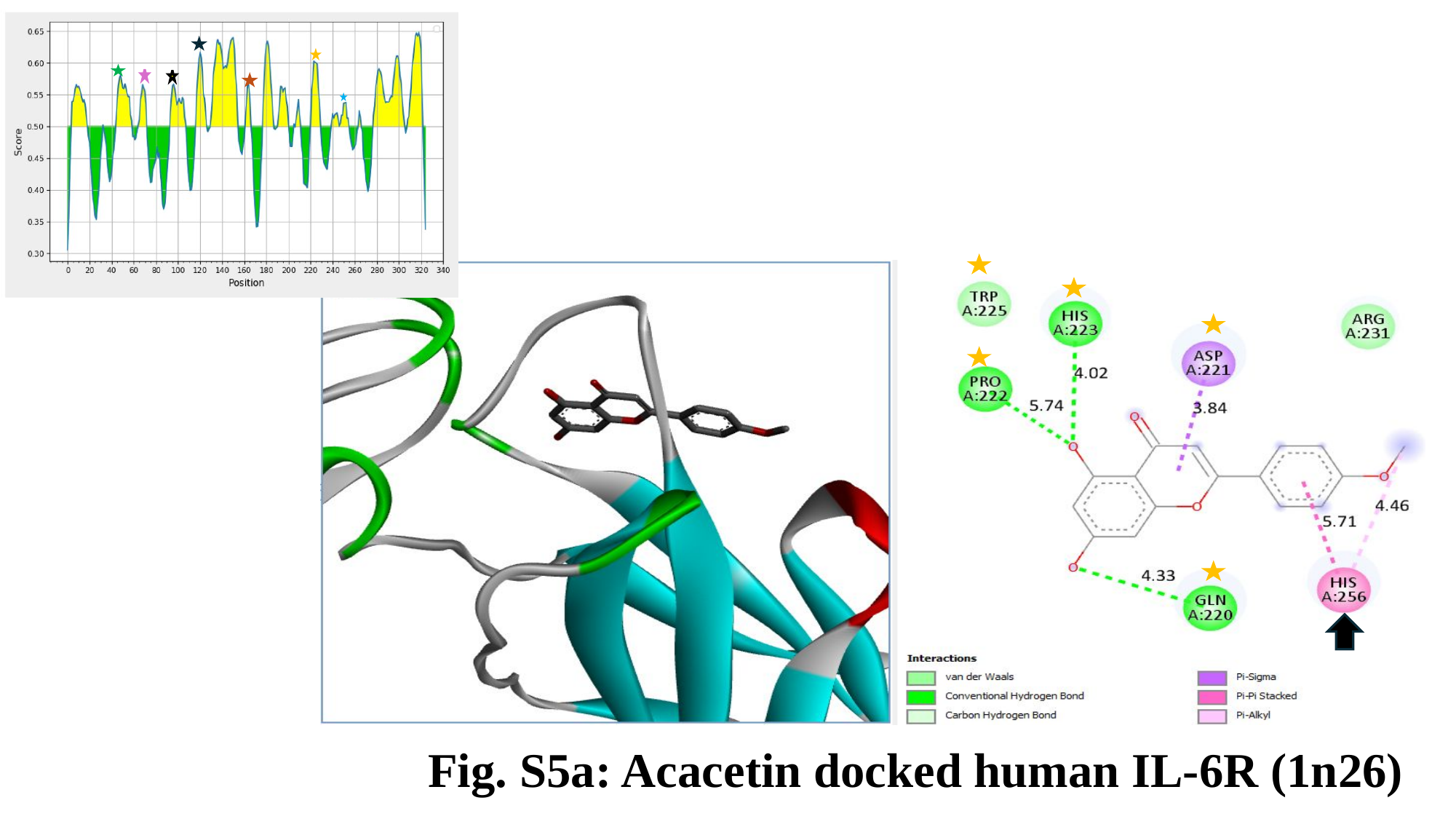

Fig. S5a: Acacetin docked human IL-6R (1n26)

## Slide 4
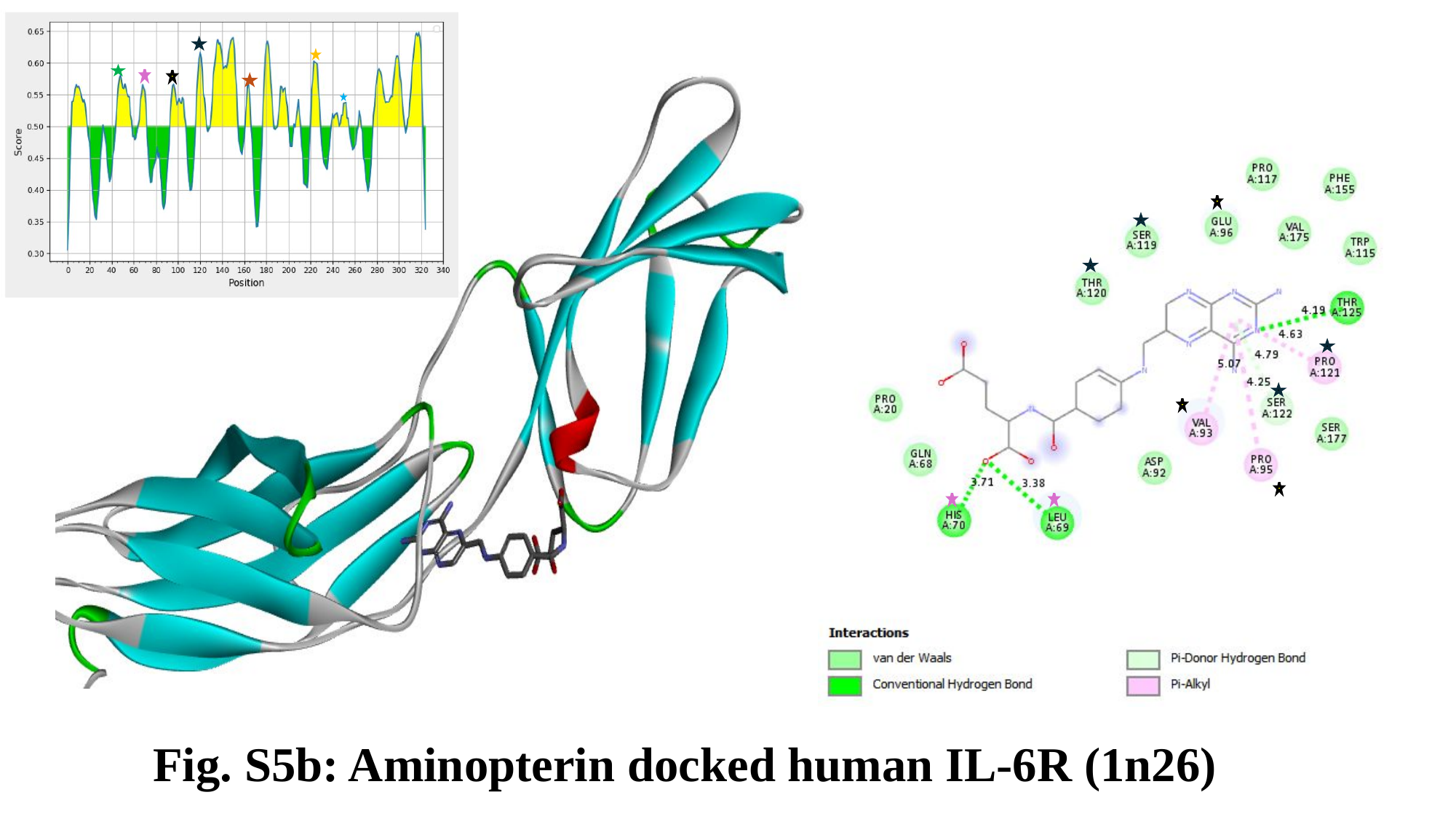

Fig. S5b: Aminopterin docked human IL-6R (1n26)

## Slide 5
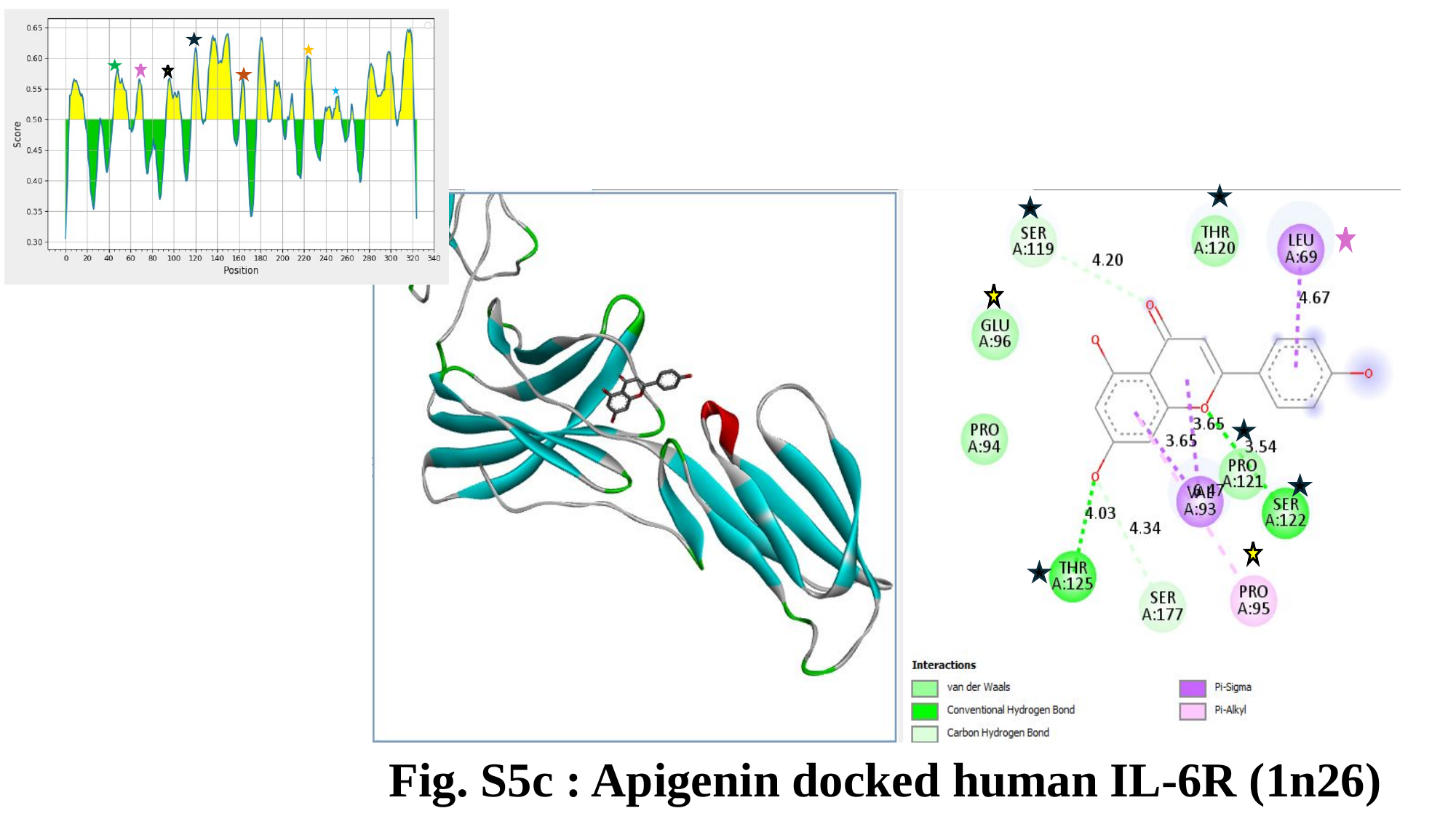

Fig. S5c : Apigenin docked human IL-6R (1n26)

## Slide 6
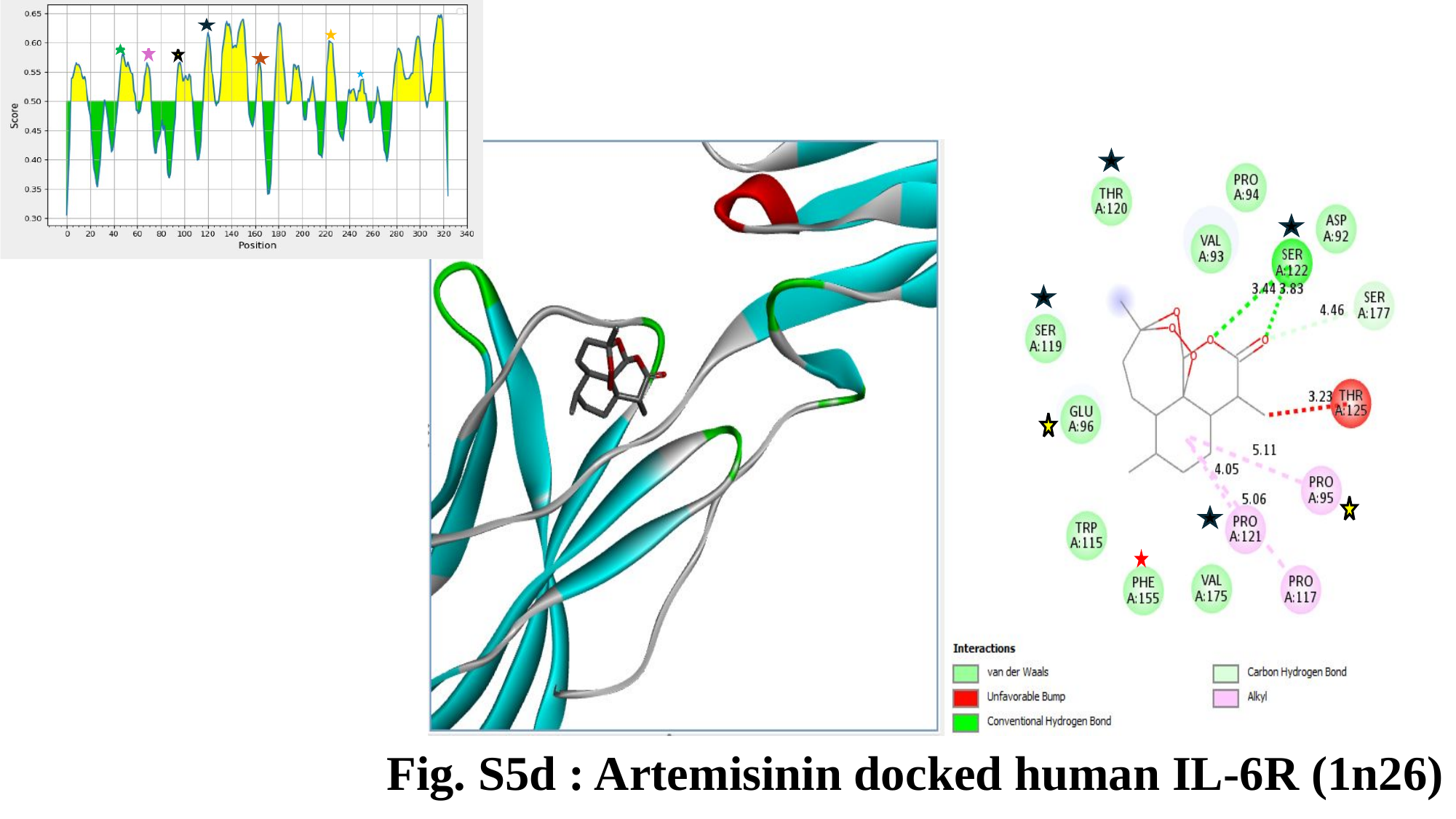

Fig. S5d : Artemisinin docked human IL-6R (1n26)

## Slide 7
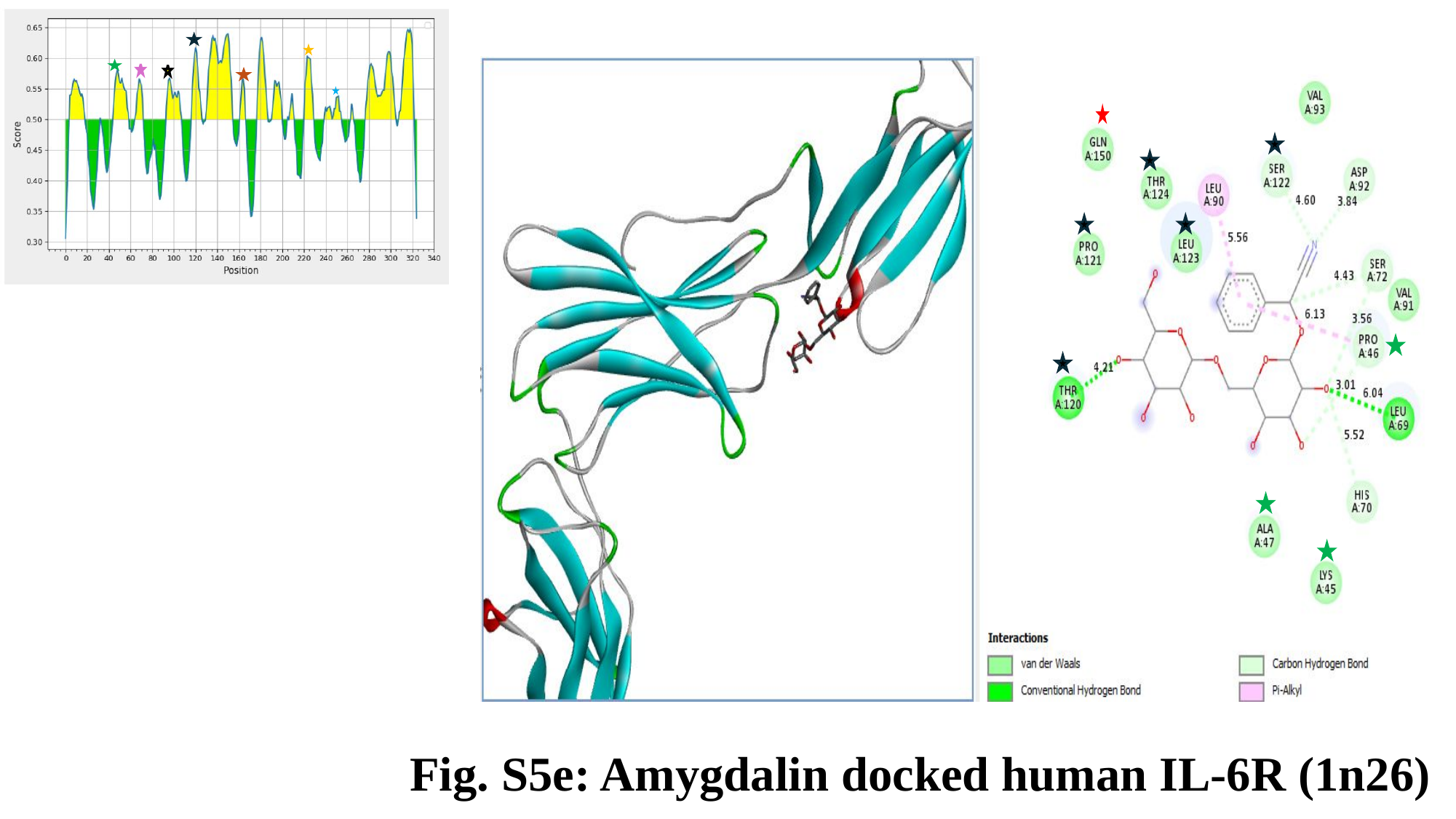

Fig. S5e: Amygdalin docked human IL-6R (1n26)

## Slide 8
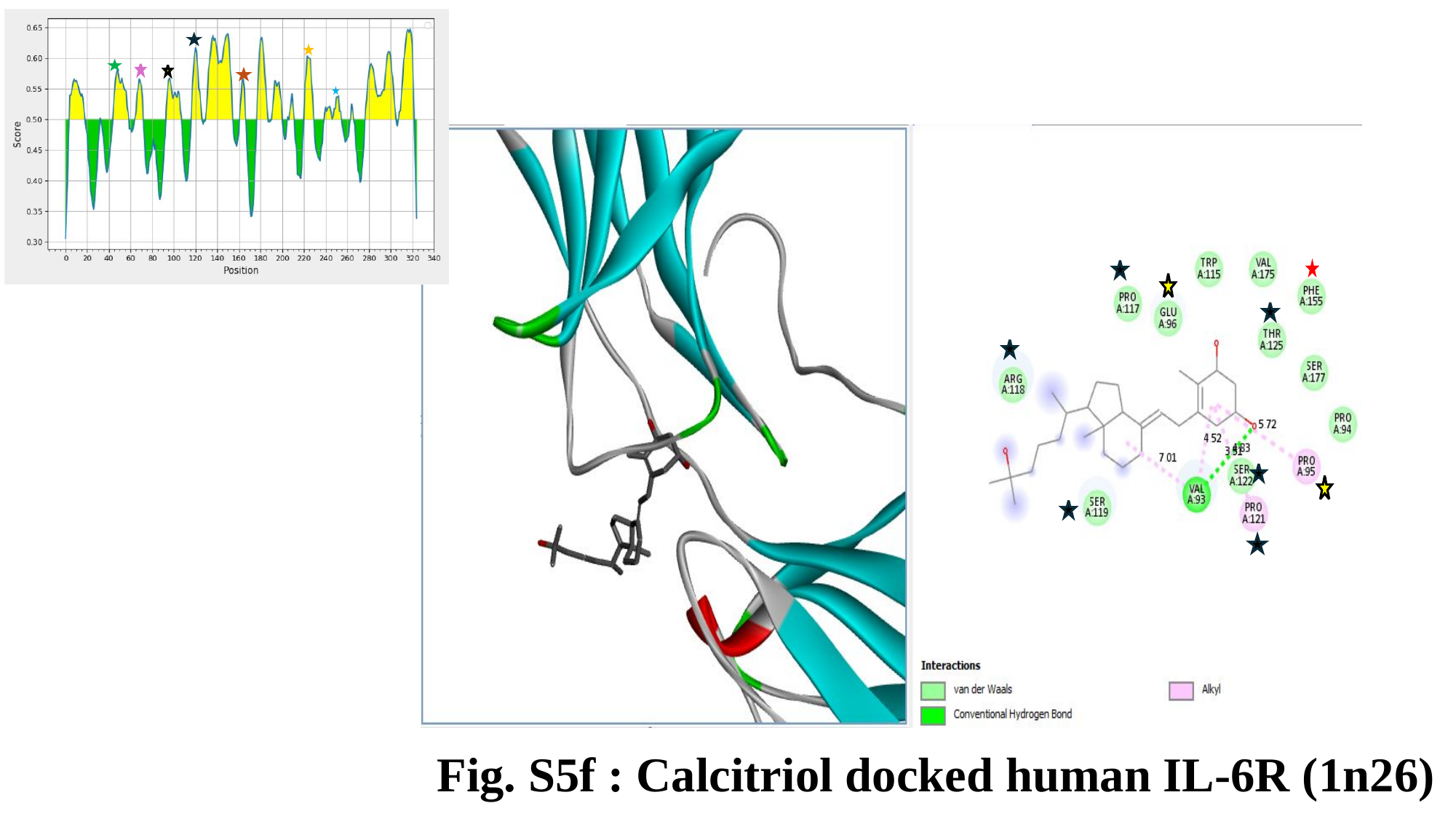

Fig. S5f : Calcitriol docked human IL-6R (1n26)

## Slide 9
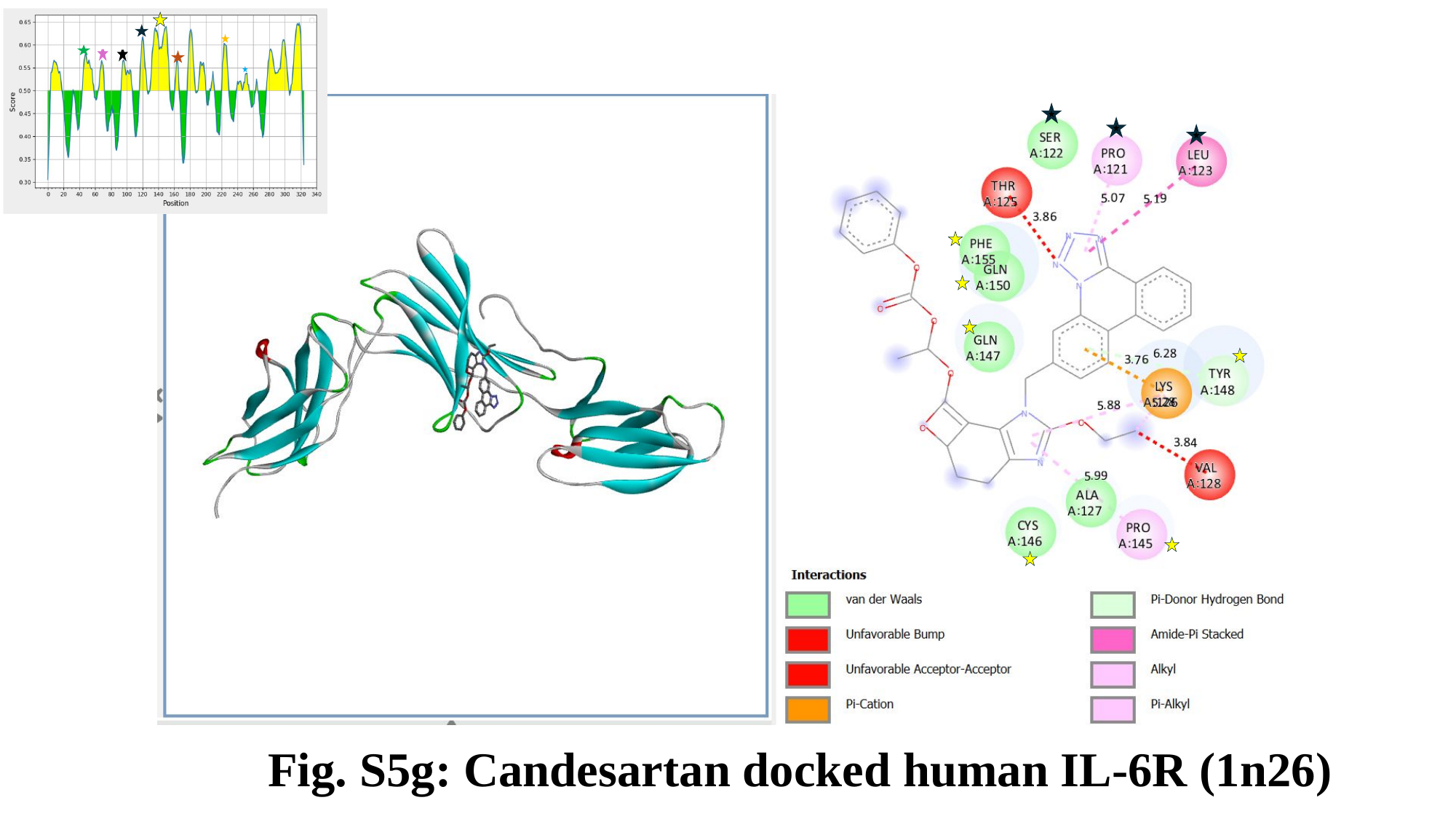

Fig. S5g: Candesartan docked human IL-6R (1n26)

## Slide 10
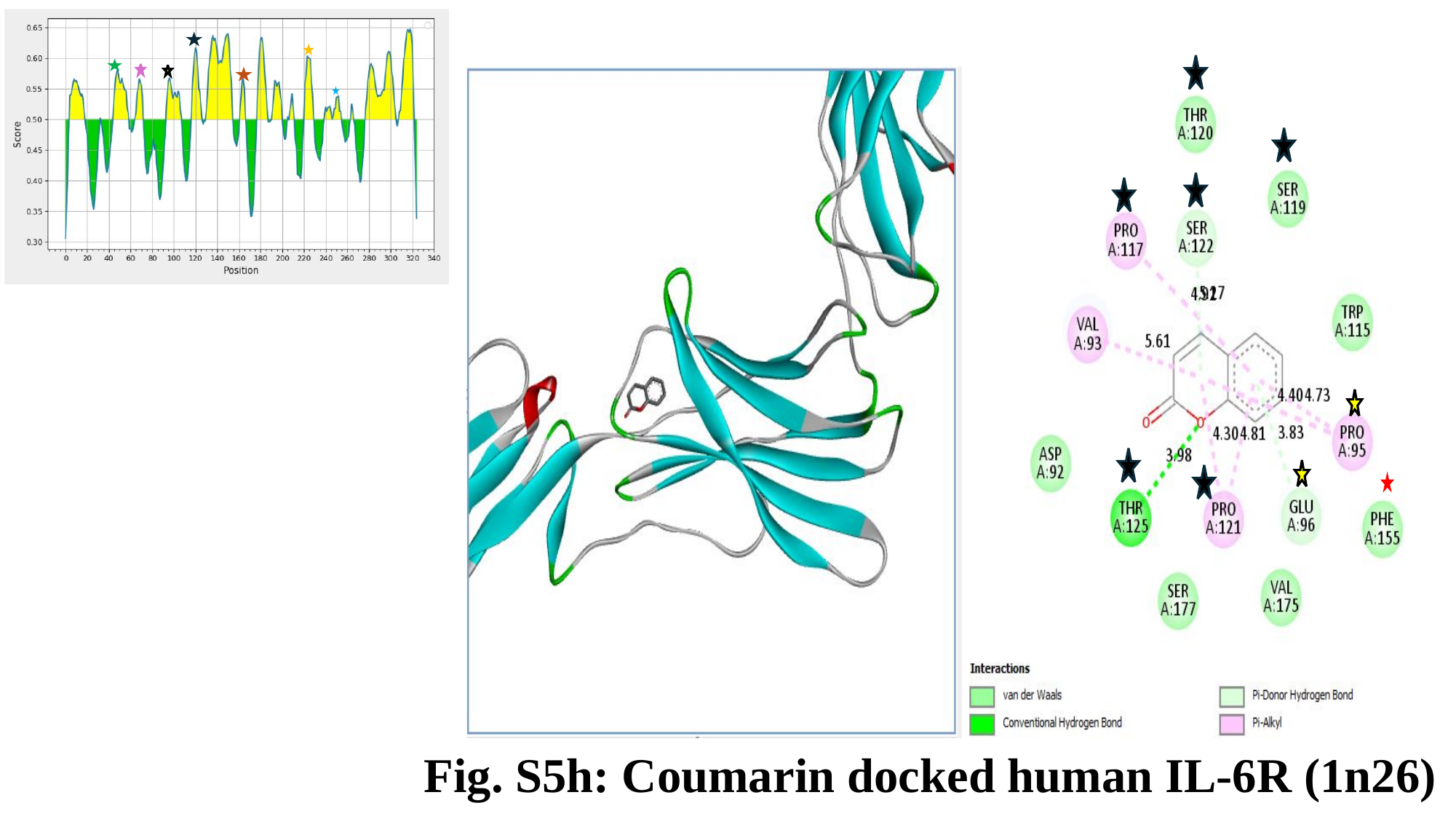

Fig. S5h: Coumarin docked human IL-6R (1n26)

## Slide 11
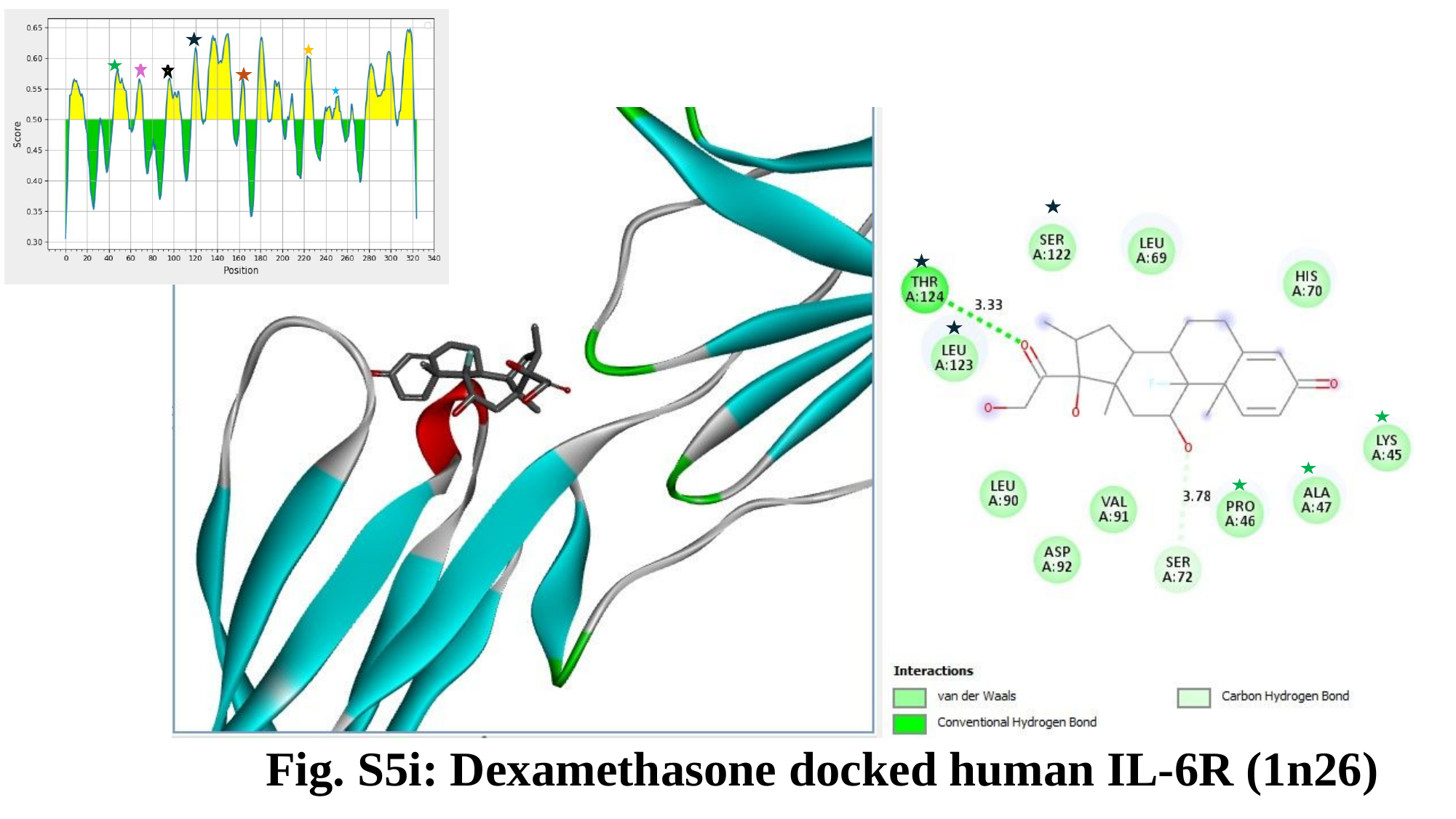

Fig. S5i: Dexamethasone docked human IL-6R (1n26)

## Slide 12
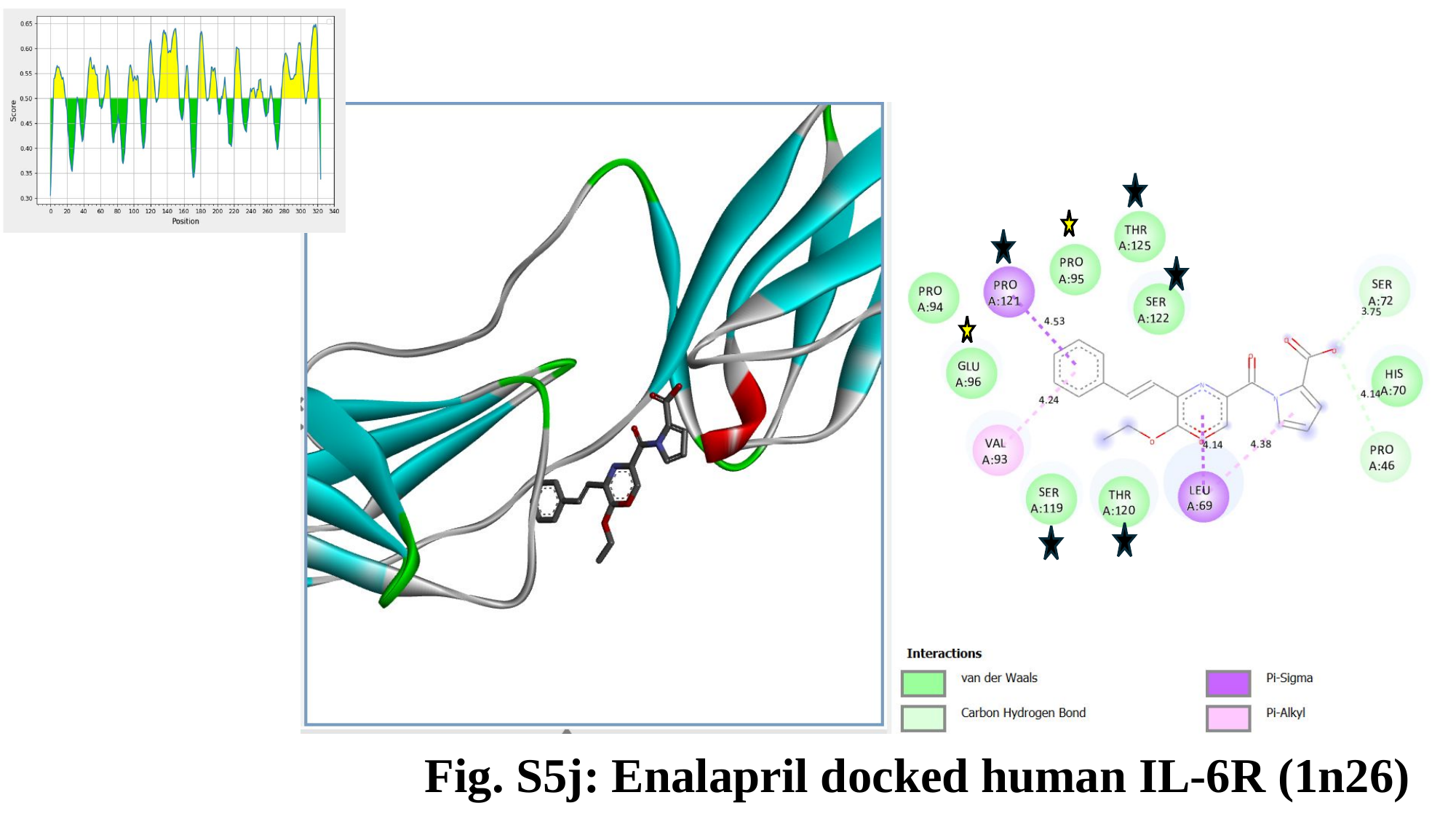

Fig. S5j: Enalapril docked human IL-6R (1n26)

## Slide 13
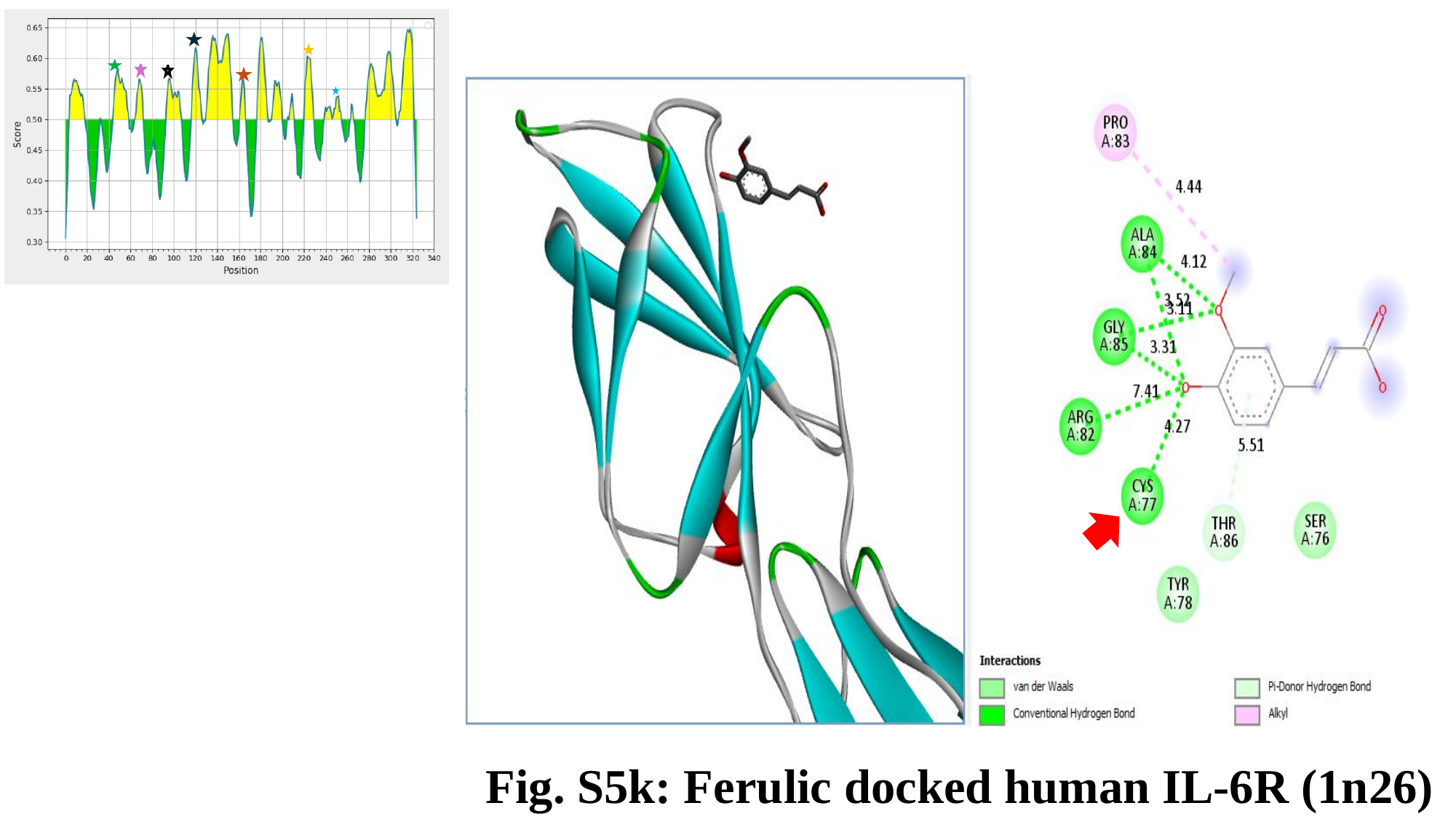

Fig. S5k: Ferulic docked human IL-6R (1n26)

## Slide 14
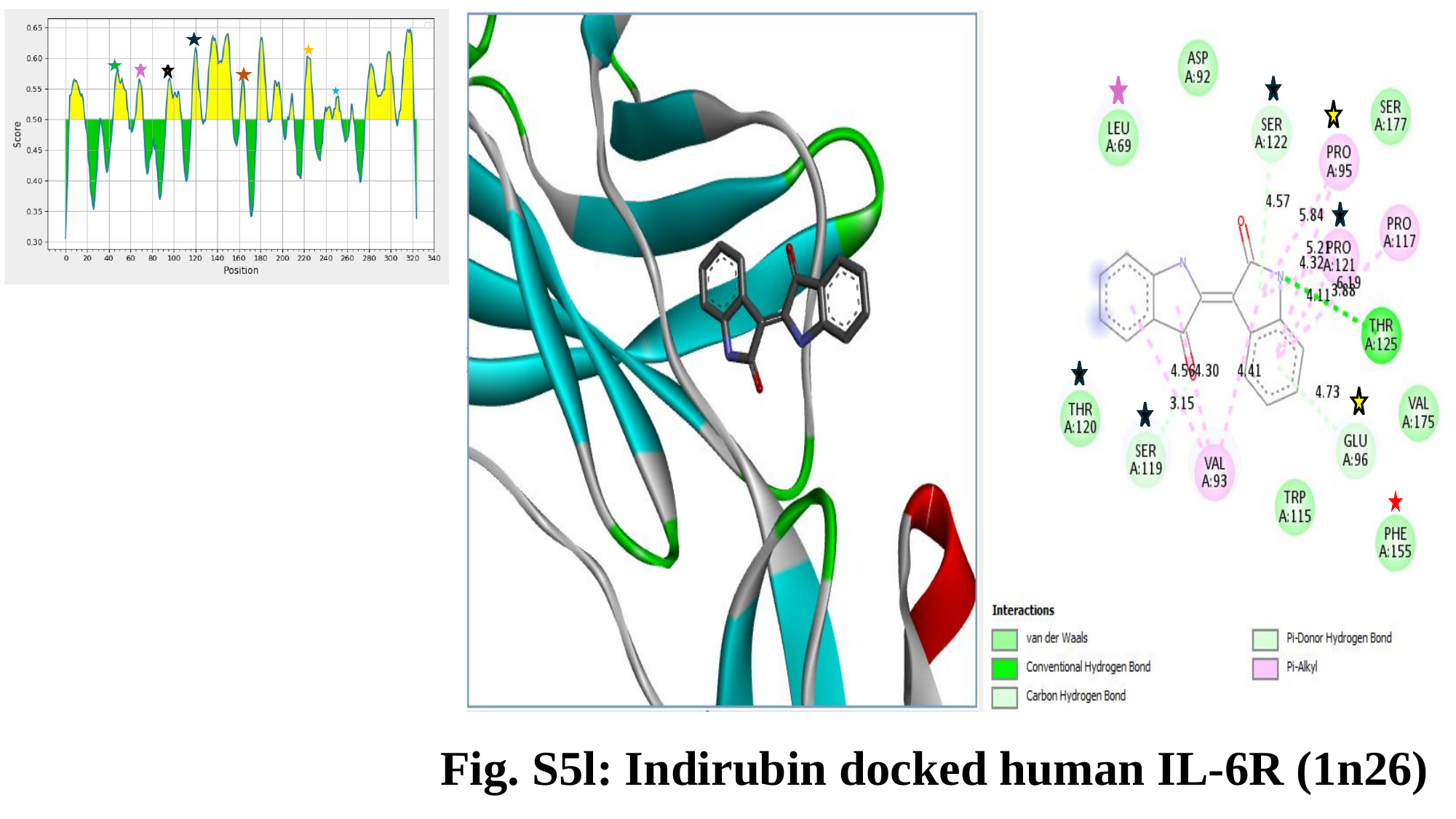

Fig. S5l: Indirubin docked human IL-6R (1n26)

## Slide 15
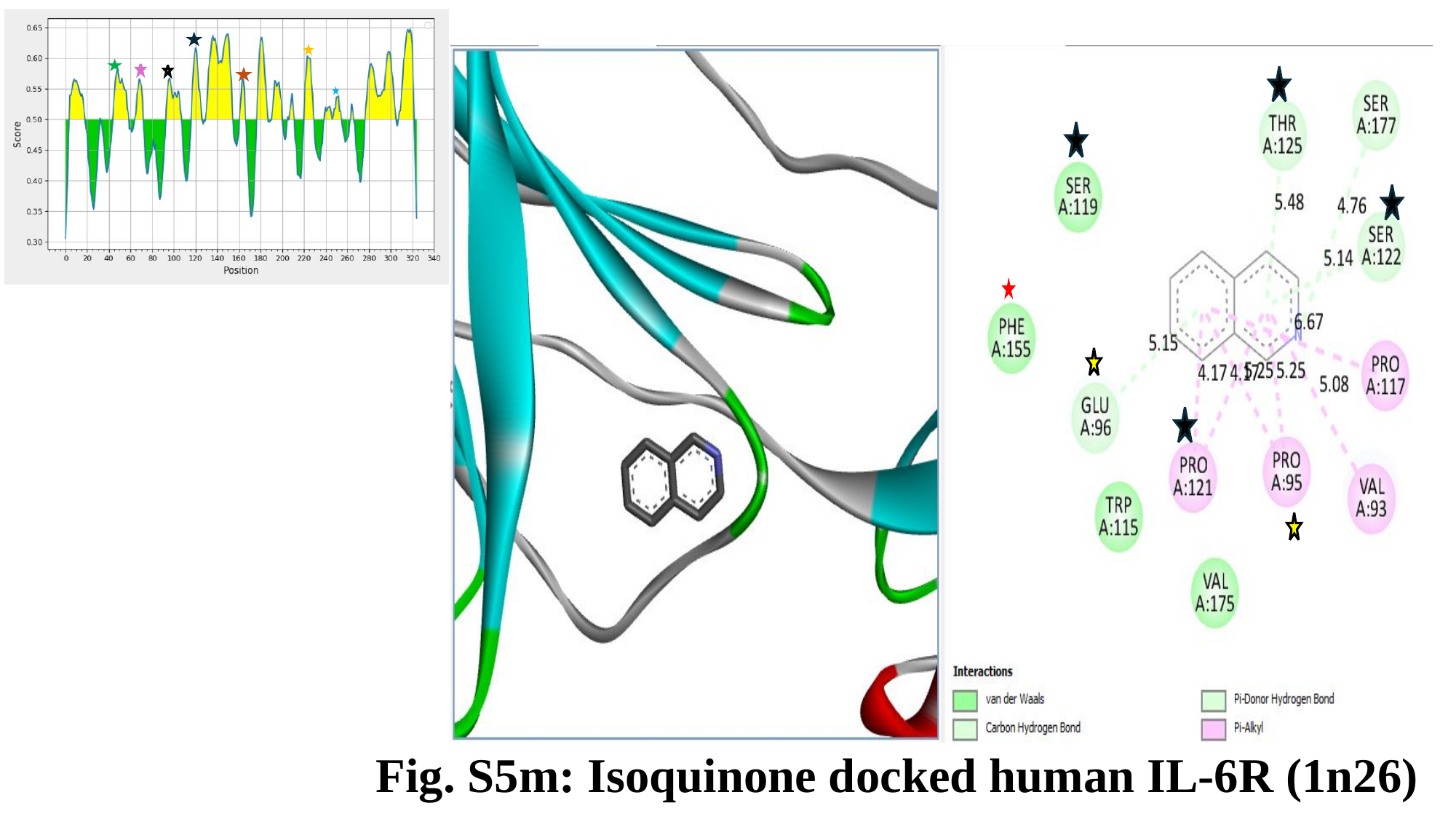

Fig. S5m: Isoquinone docked human IL-6R (1n26)

## Slide 16
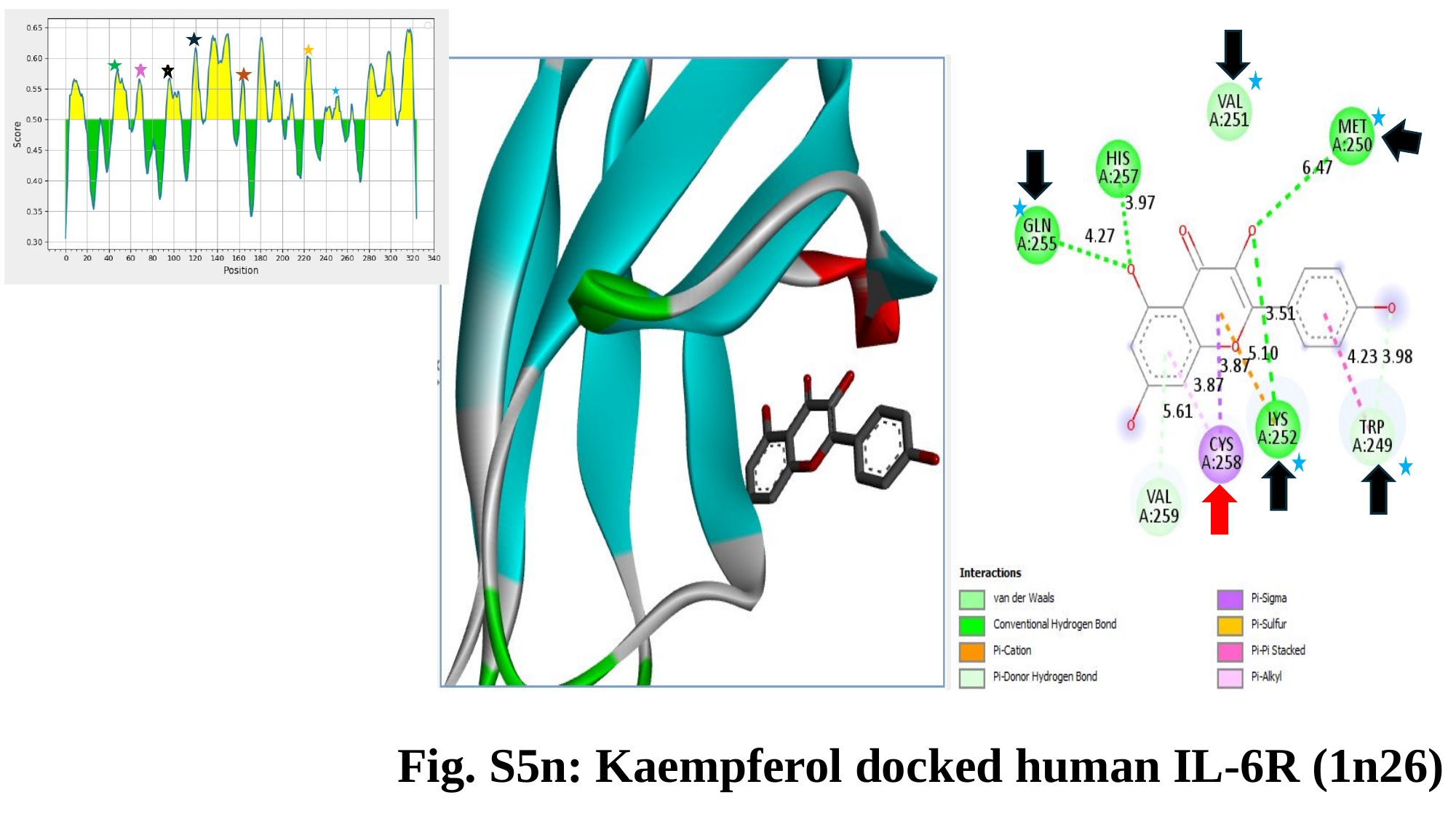

Fig. S5n: Kaempferol docked human IL-6R (1n26)

## Slide 17
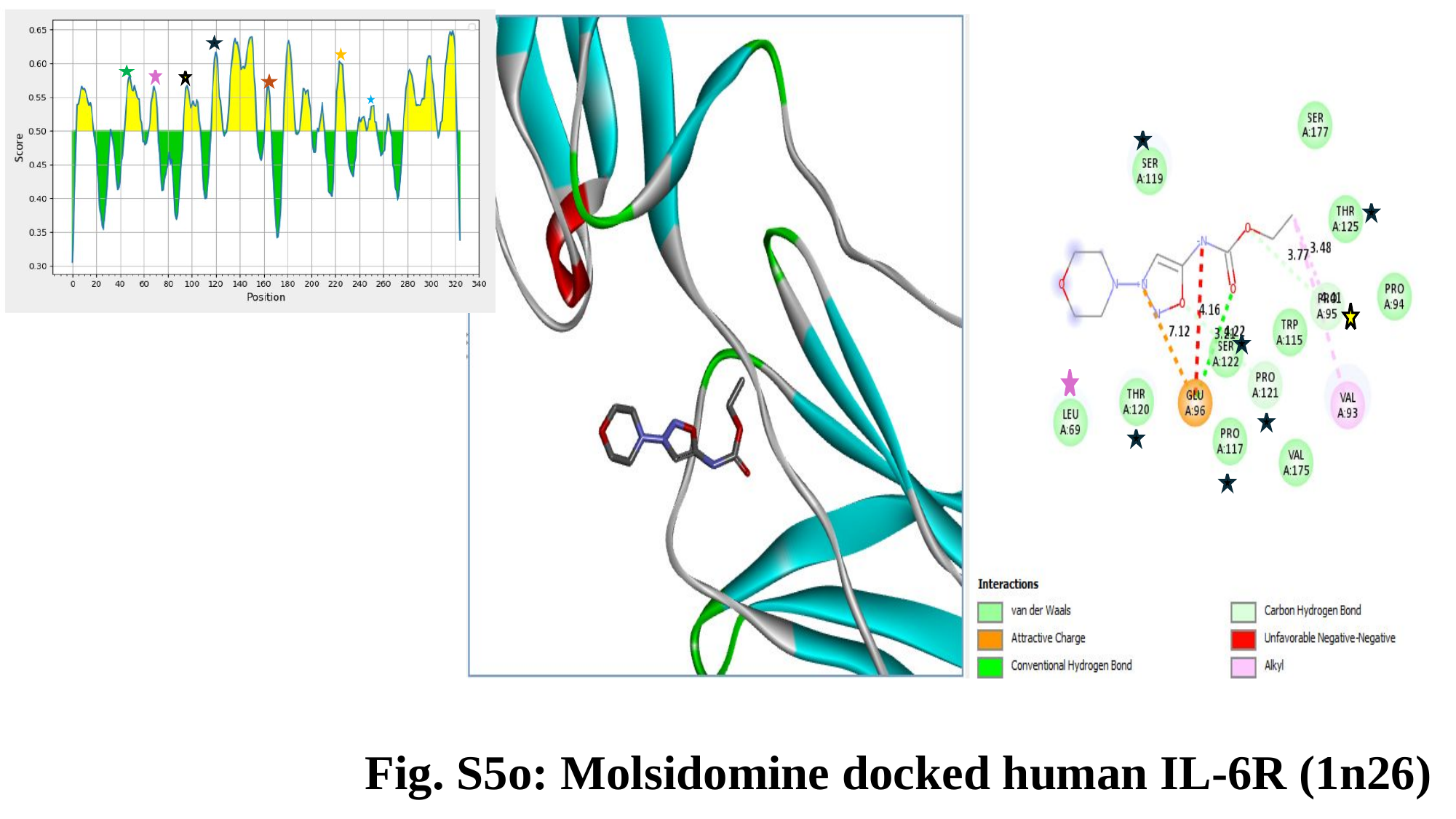

Fig. S5o: Molsidomine docked human IL-6R (1n26)

## Slide 18
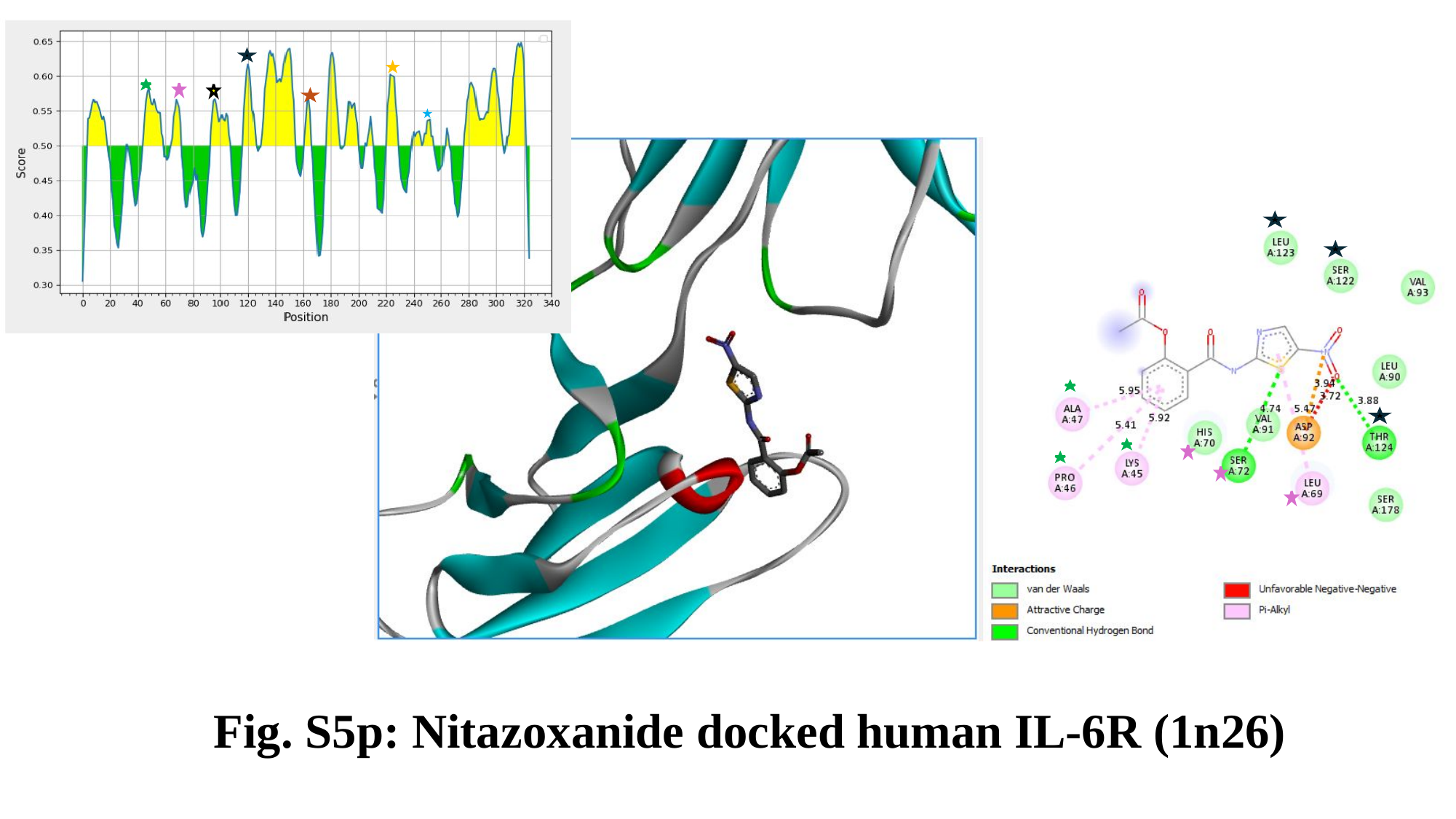

Fig. S5p: Nitazoxanide docked human IL-6R (1n26)

## Slide 19
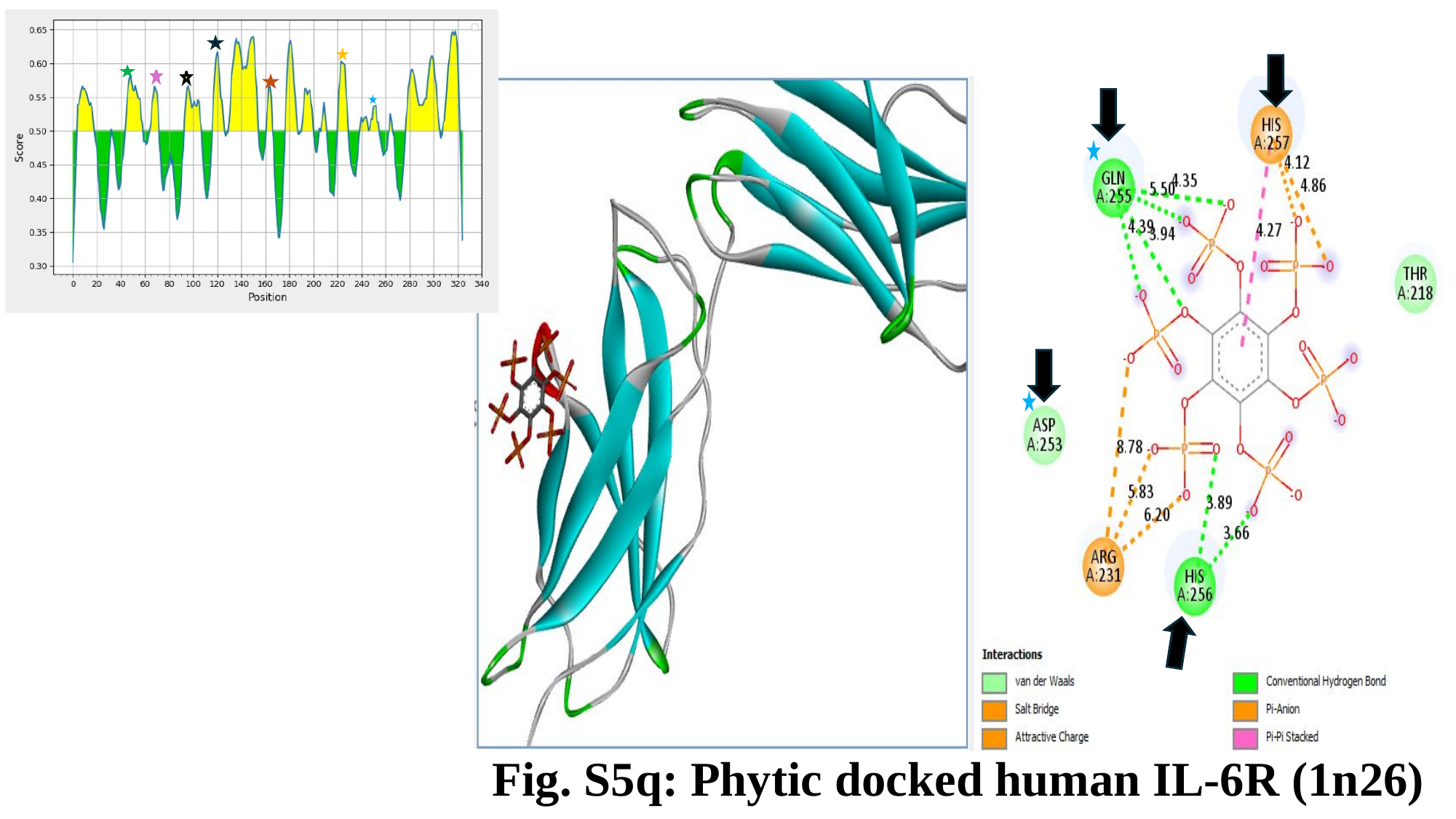

Fig. S5q: Phytic docked human IL-6R (1n26)

## Slide 20
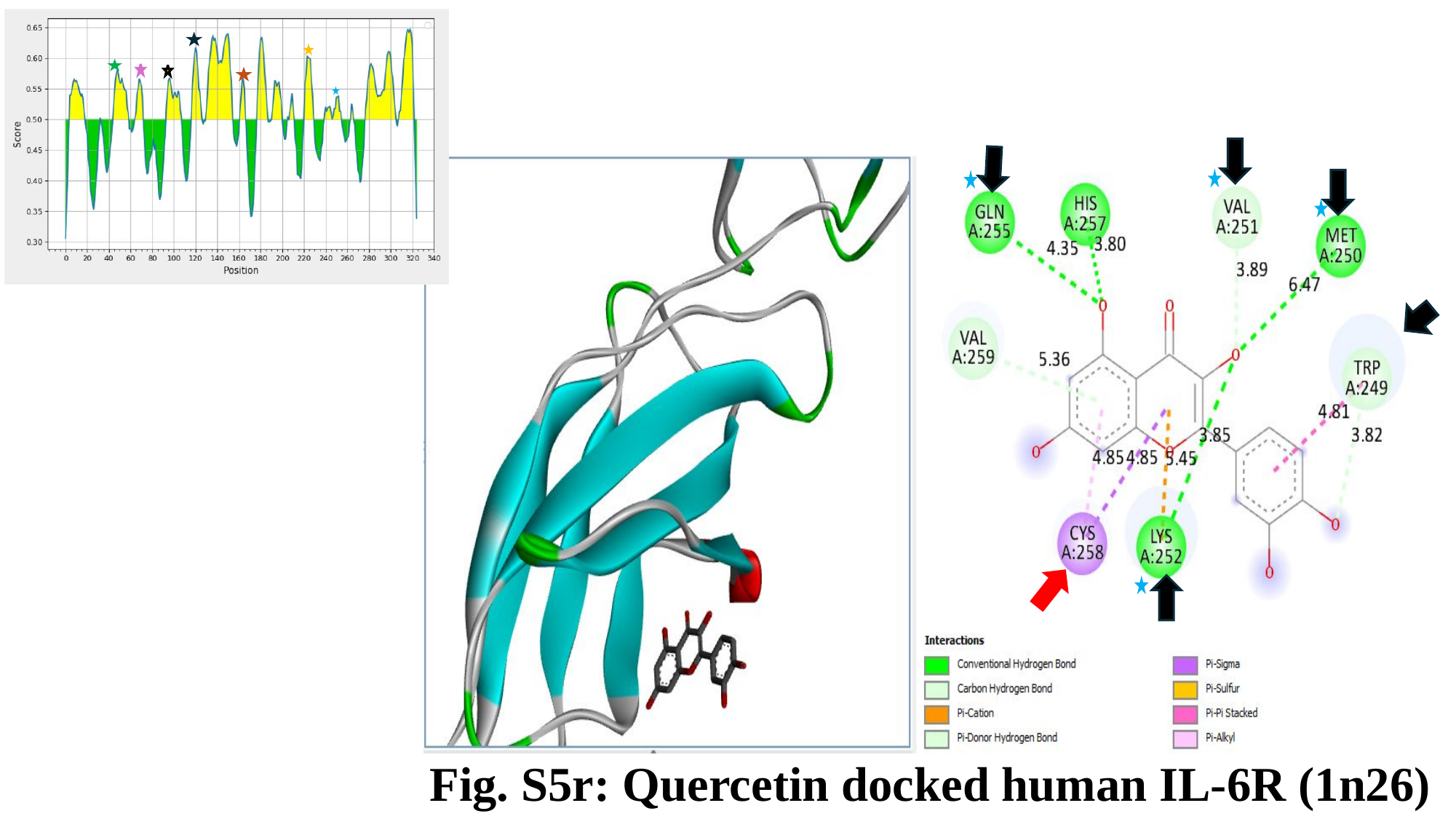

Fig. S5r: Quercetin docked human IL-6R (1n26)

## Slide 21
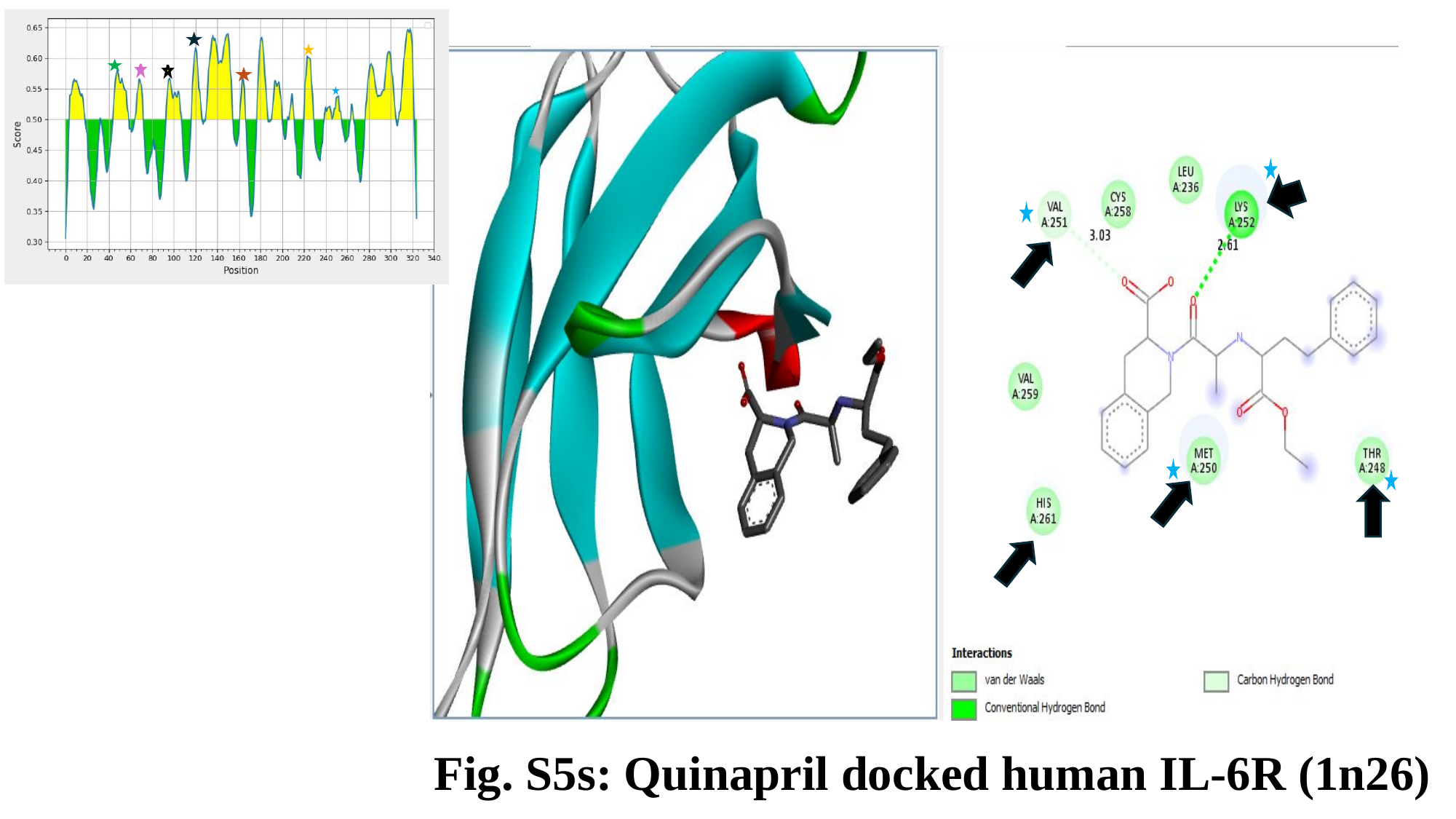

Fig. S5s: Quinapril docked human IL-6R (1n26)

## Slide 22
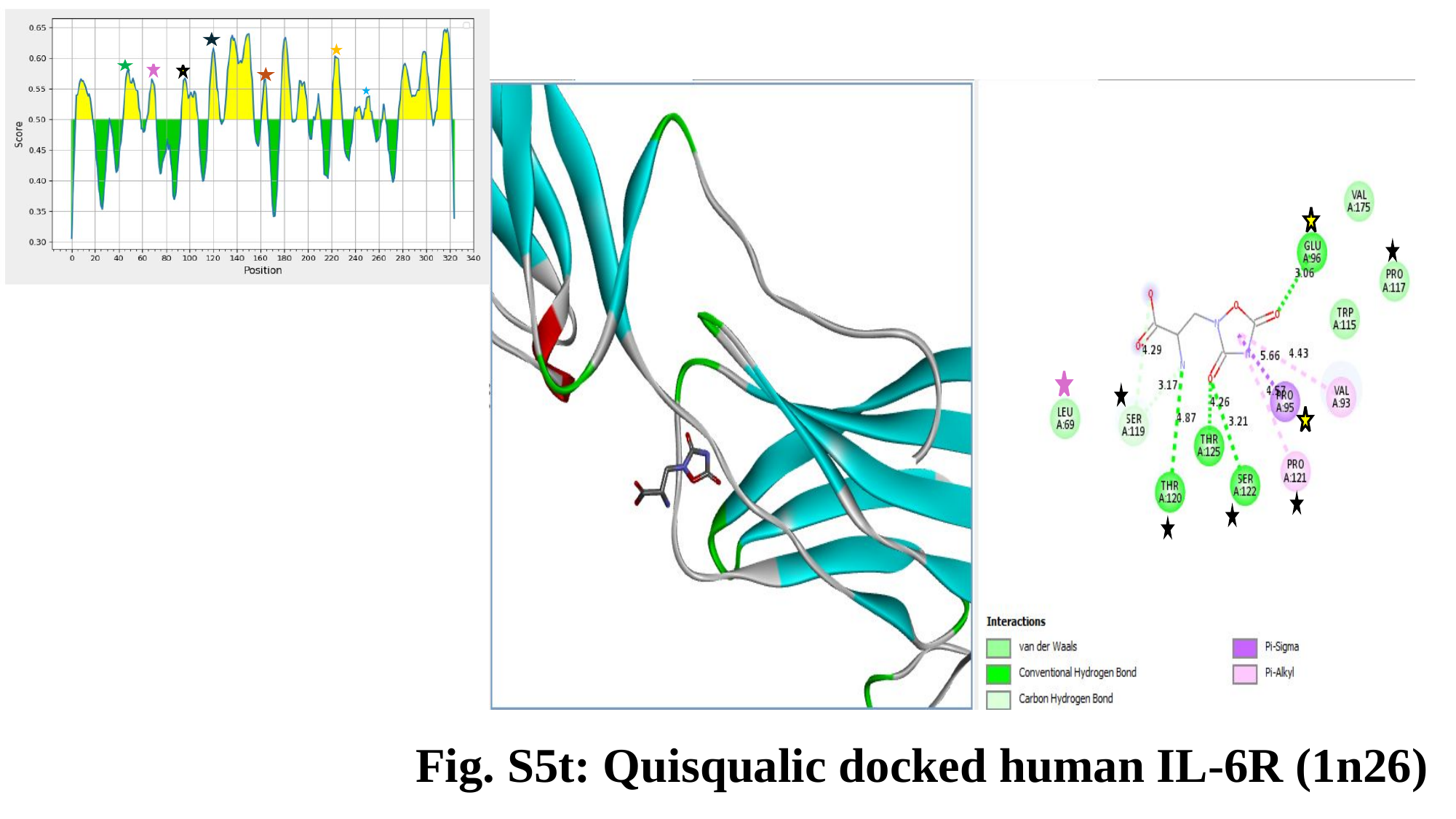

Fig. S5t: Quisqualic docked human IL-6R (1n26)

## Slide 23
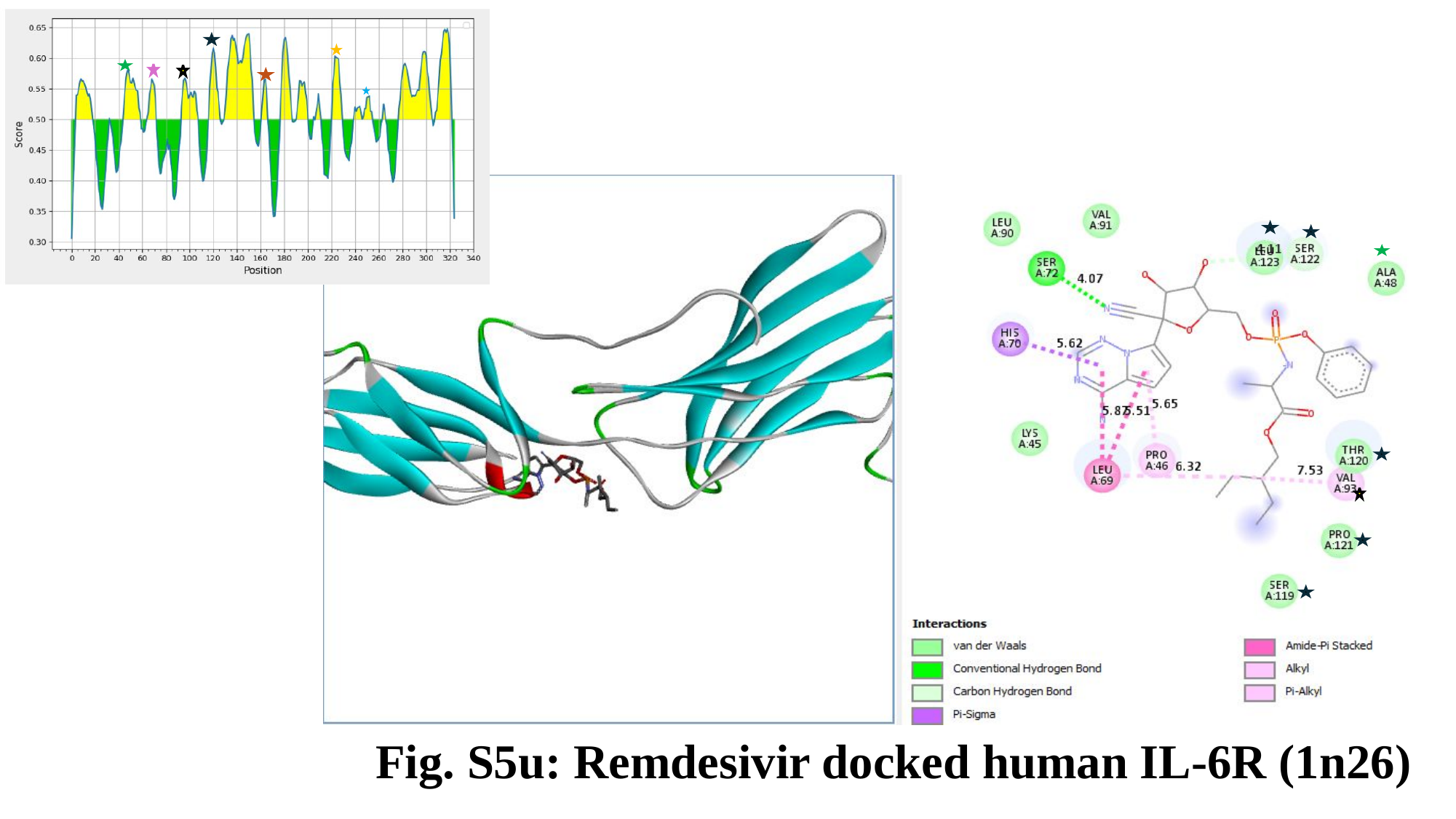

Fig. S5u: Remdesivir docked human IL-6R (1n26)

## Slide 24
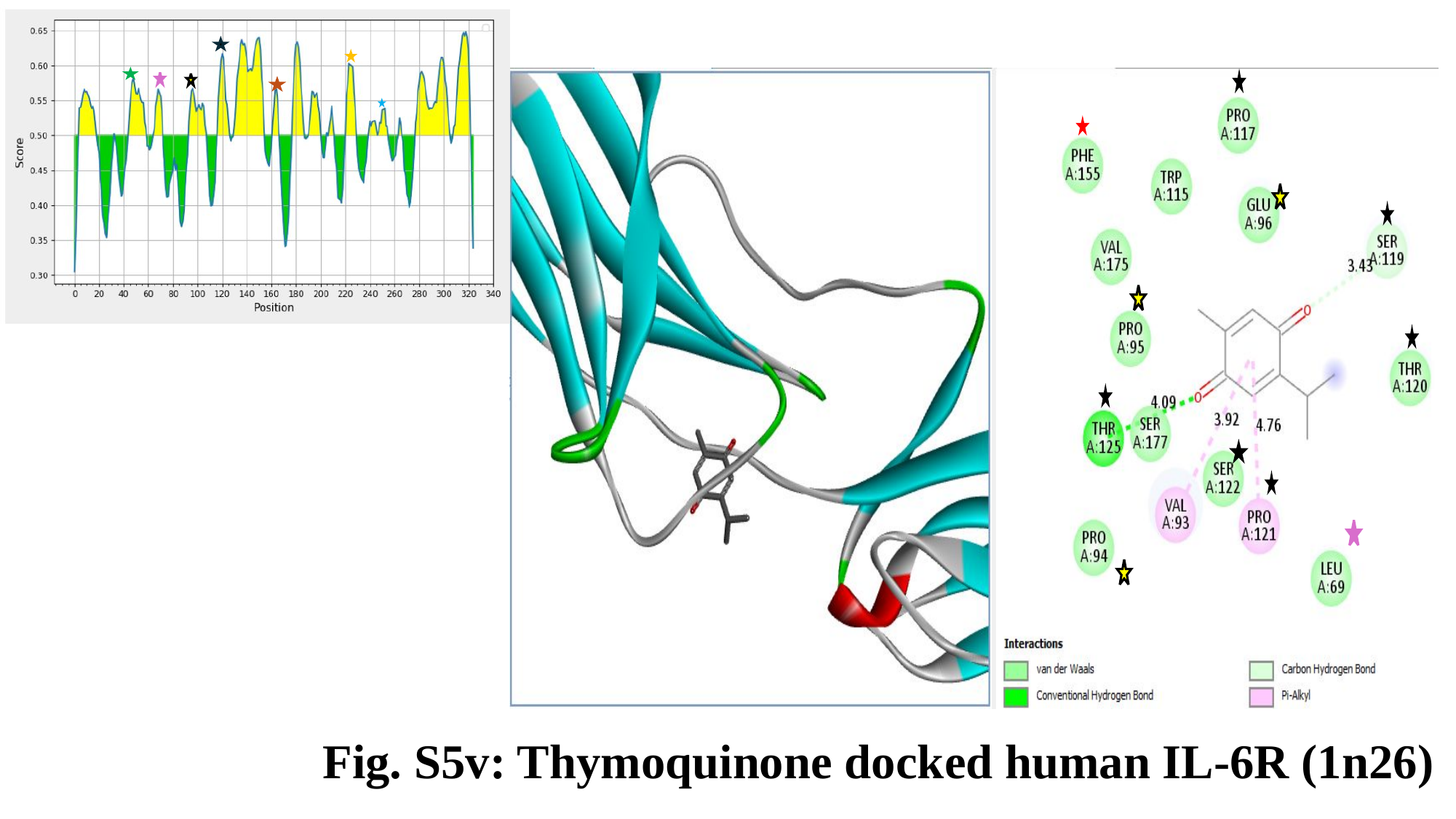

Fig. S5v: Thymoquinone docked human IL-6R (1n26)

## Slide 25
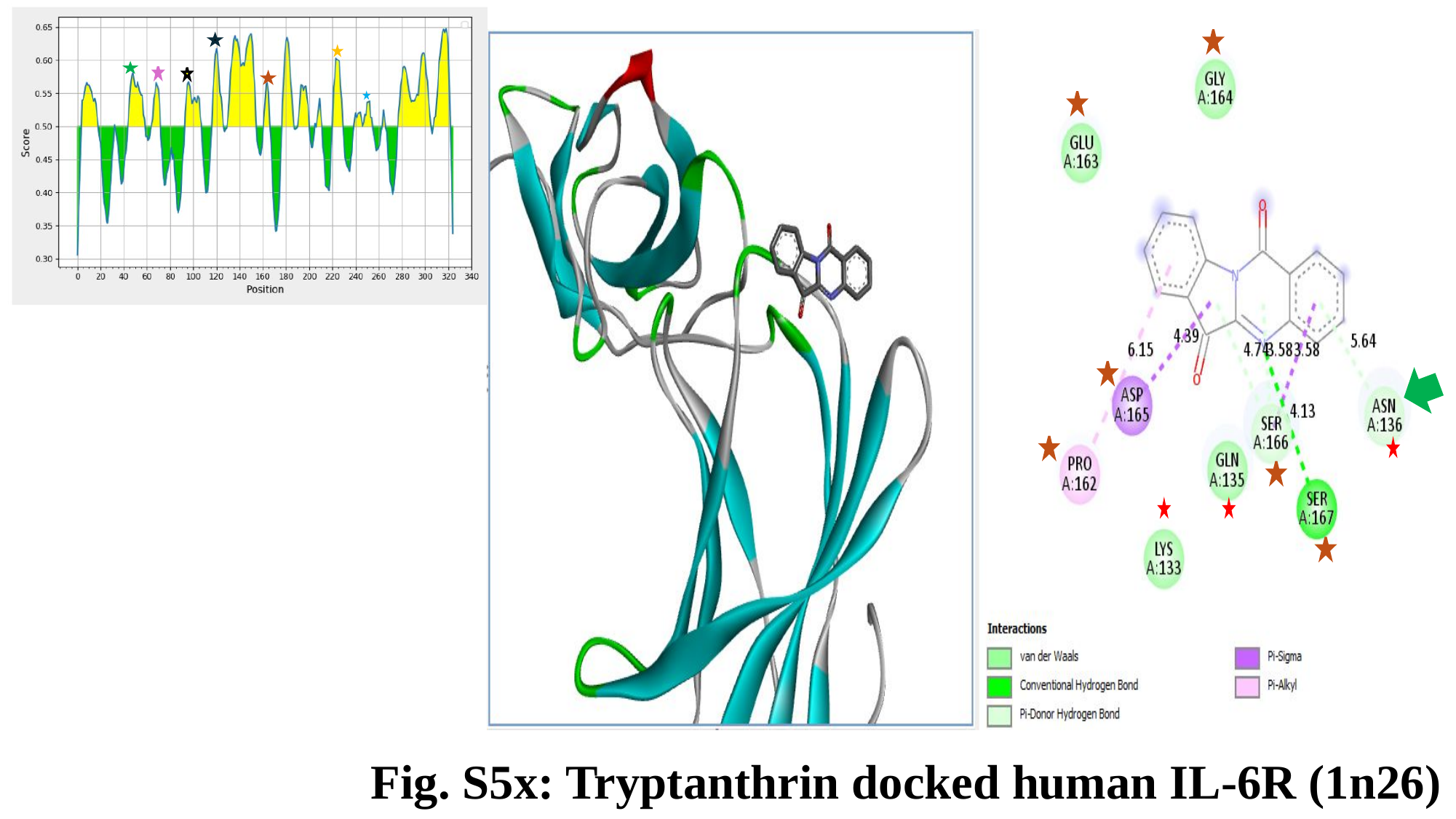

Fig. S5x: Tryptanthrin docked human IL-6R (1n26)
