# Supplementary material for: Targeting asparagine and cysteine in SARS-CoV-2 variants and human pro-inflammatory mediators to alleviate COVID-19 severity; a cross-section and in-silico study
Source: Sci Rep. 2025 Nov 3;15:38445. doi: 10.1038/s41598-025-19359-y (PMC12583749; doi:10.1038/s41598-025-19359-y)
Supplement: Supplementary file 13 — Supplementary Material 13 [file 41598_2025_19359_MOESM13_ESM.pptx]

## Slide 1
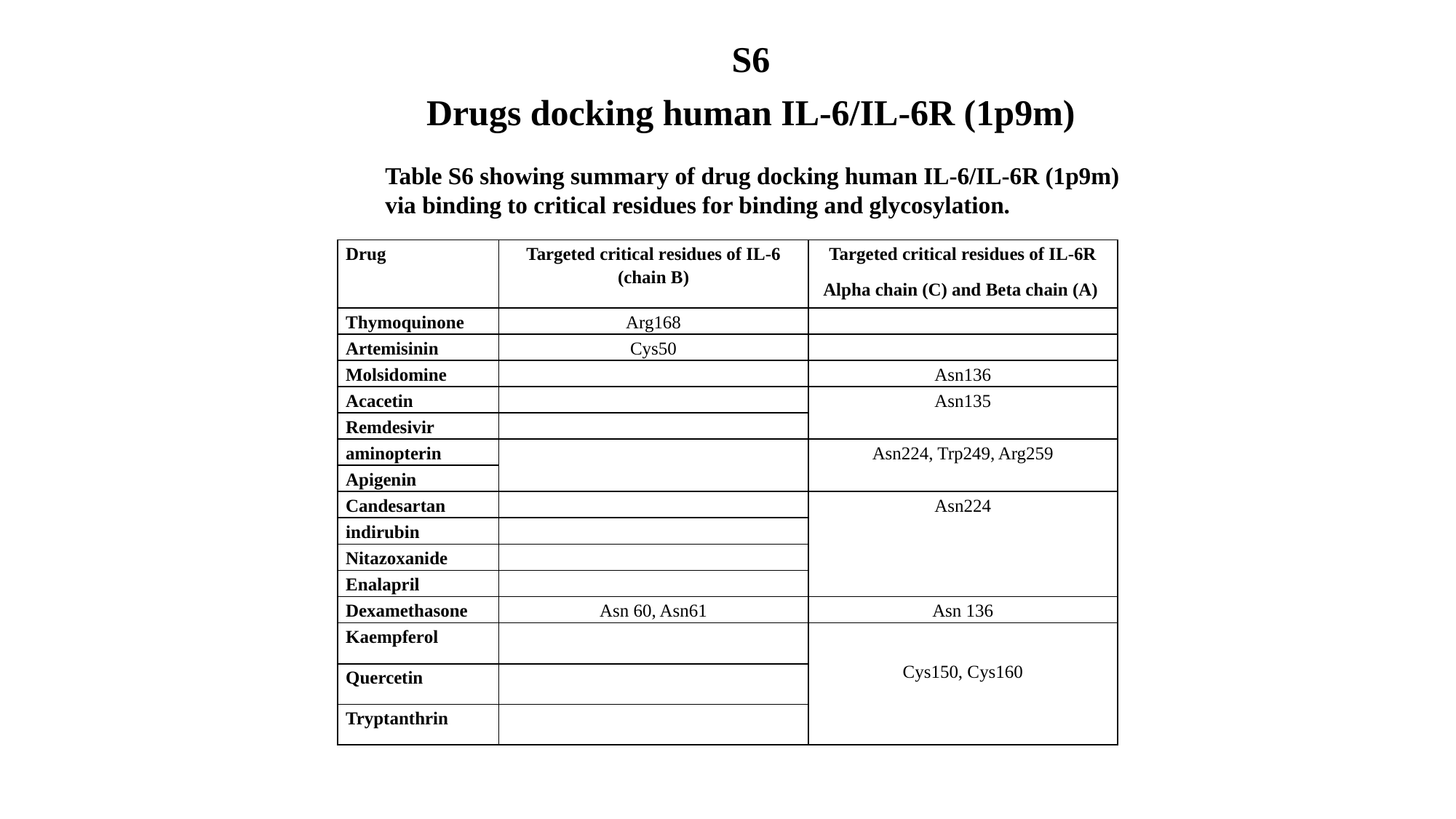

S6
Drugs docking human IL-6/IL-6R (1p9m)
Table S6 showing summary of drug docking human IL-6/IL-6R (1p9m)
via binding to critical residues for binding and glycosylation.
| Drug | Targeted critical residues of IL-6 (chain B) | Targeted critical residues of IL-6R Alpha chain (C) and Beta chain (A) |
| --- | --- | --- |
| Thymoquinone | Arg168 | |
| Artemisinin | Cys50 | |
| Molsidomine | | Asn136 |
| Acacetin | | Asn135 |
| Remdesivir | | |
| aminopterin | | Asn224, Trp249, Arg259 |
| Apigenin | | |
| Candesartan | | Asn224 |
| indirubin | | |
| Nitazoxanide | | |
| Enalapril | | |
| Dexamethasone | Asn 60, Asn61 | Asn 136 |
| Kaempferol | | Cys150, Cys160 |
| Quercetin | | |
| Tryptanthrin | | |

## Slide 2
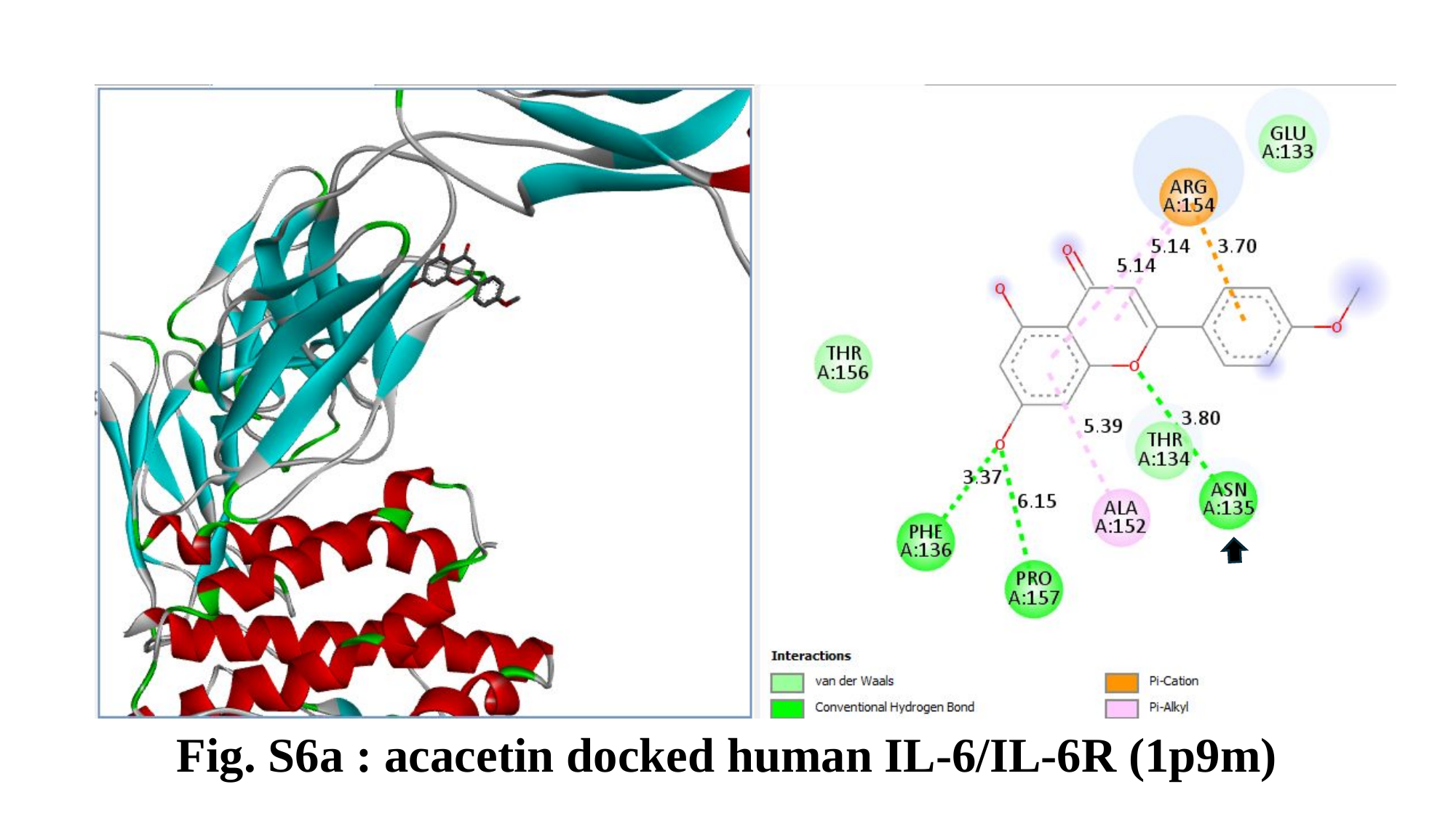

Fig. S6a : acacetin docked human IL-6/IL-6R (1p9m)

## Slide 3
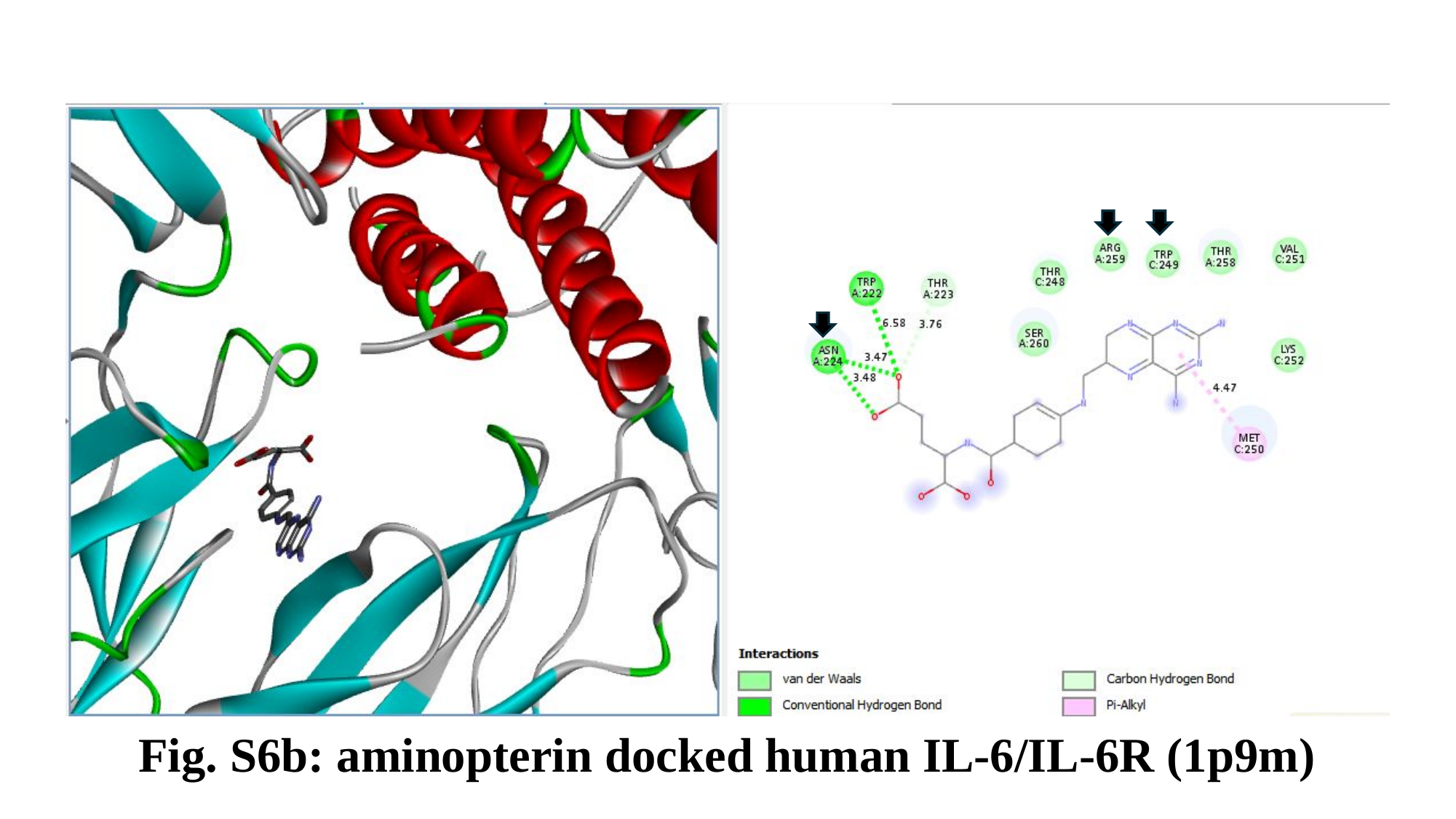

Fig. S6b: aminopterin docked human IL-6/IL-6R (1p9m)

## Slide 4
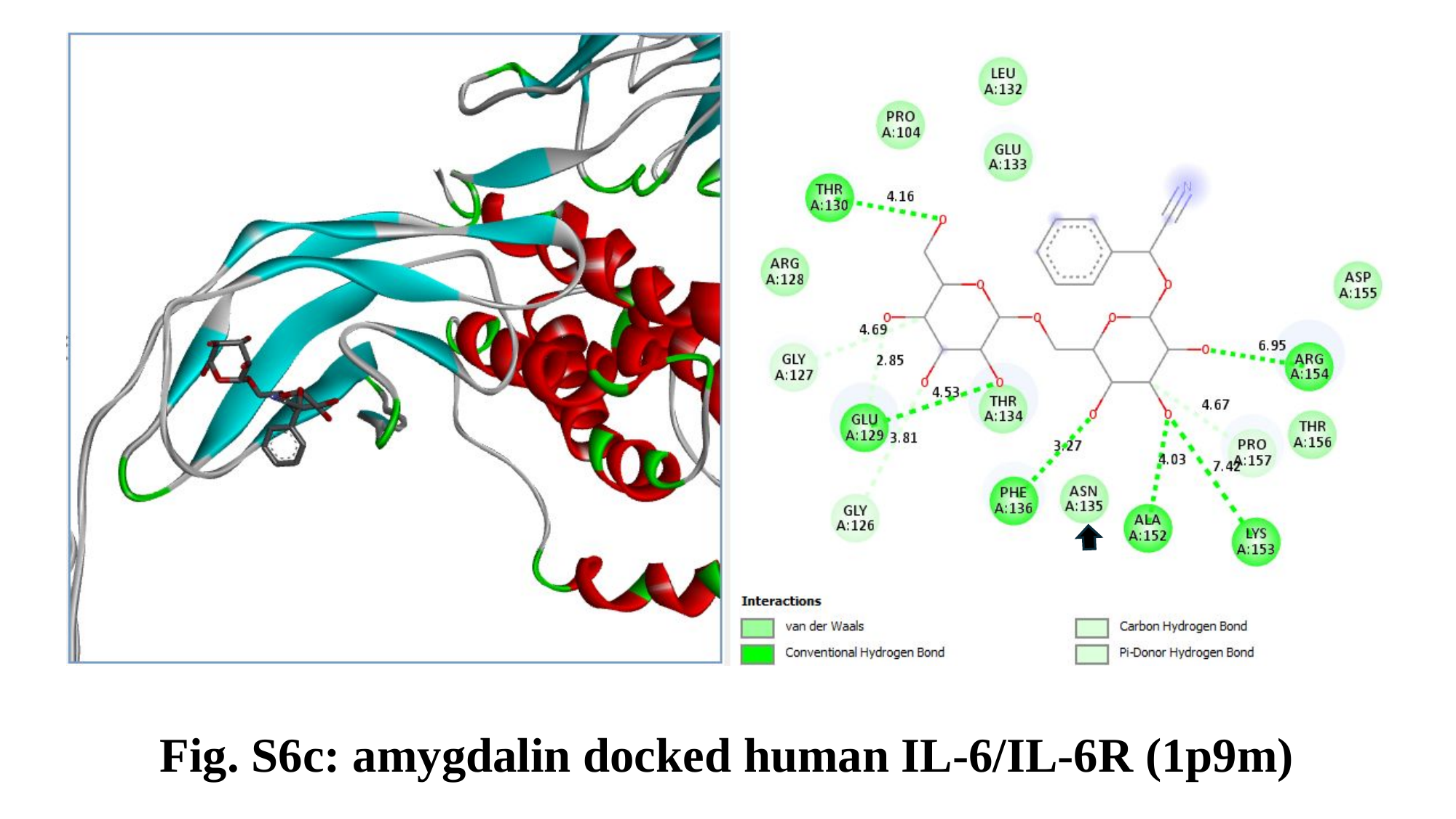

Fig. S6c: amygdalin docked human IL-6/IL-6R (1p9m)

## Slide 5
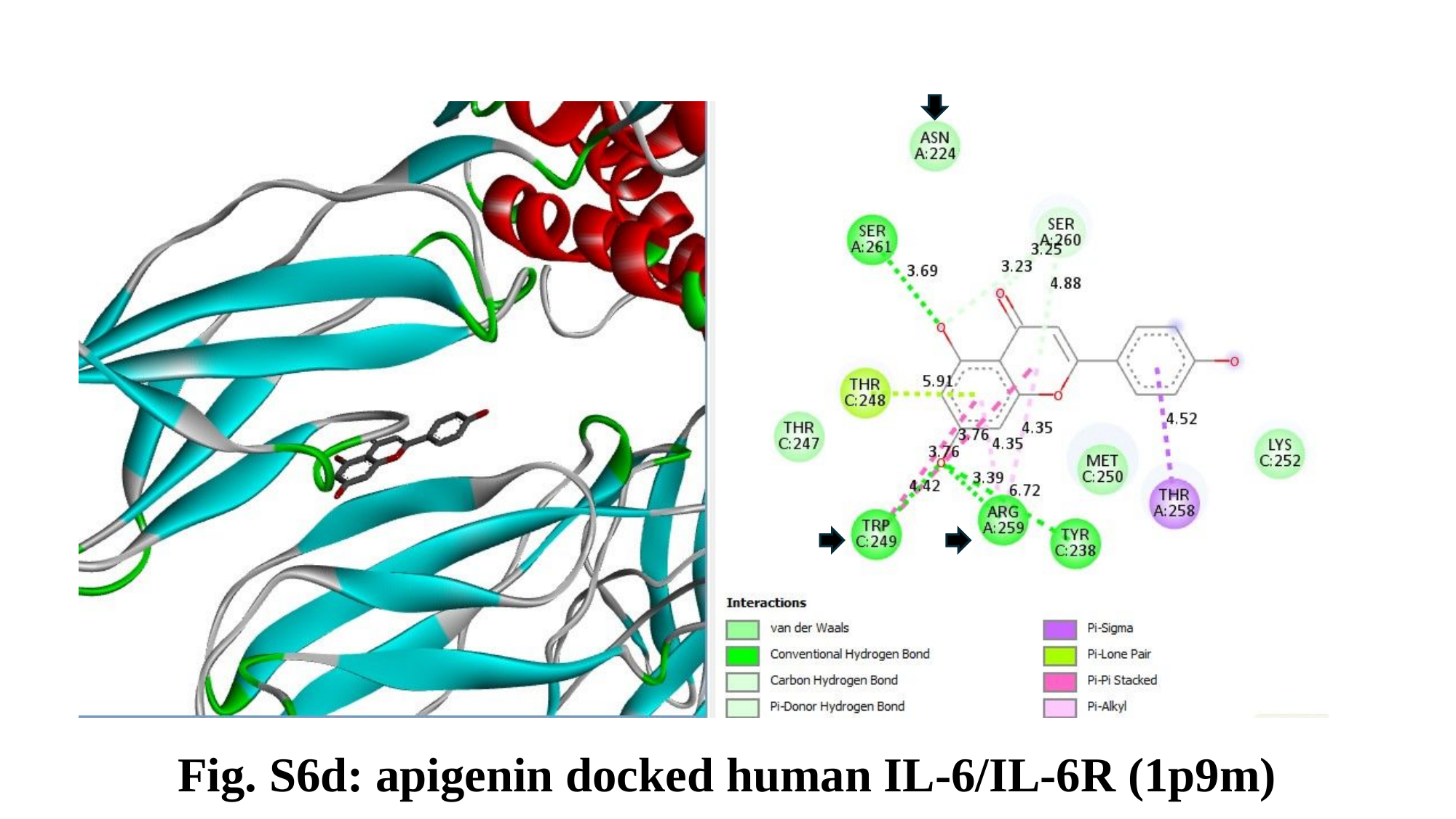

Fig. S6d: apigenin docked human IL-6/IL-6R (1p9m)

## Slide 6
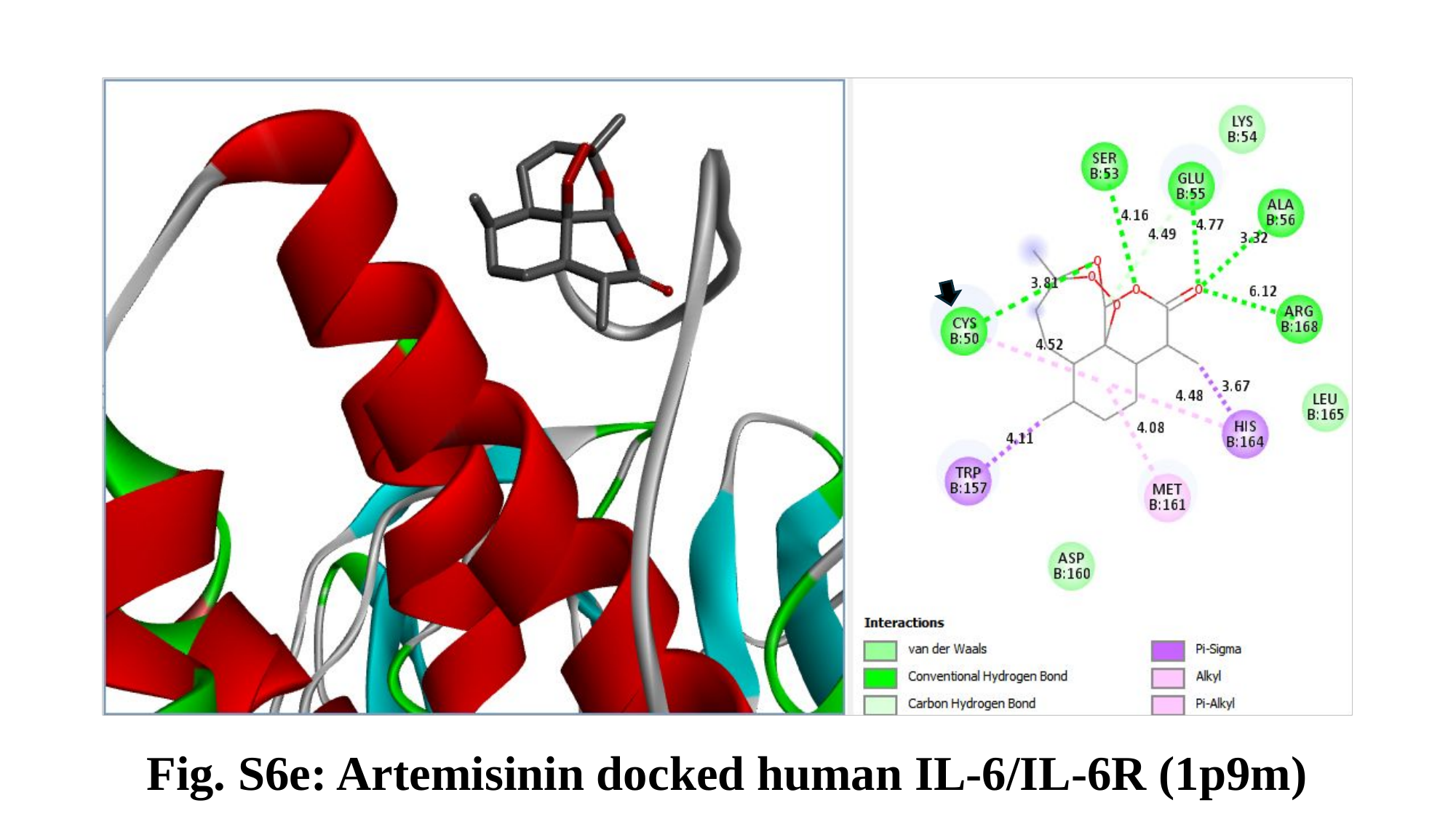

Fig. S6e: Artemisinin docked human IL-6/IL-6R (1p9m)

## Slide 7
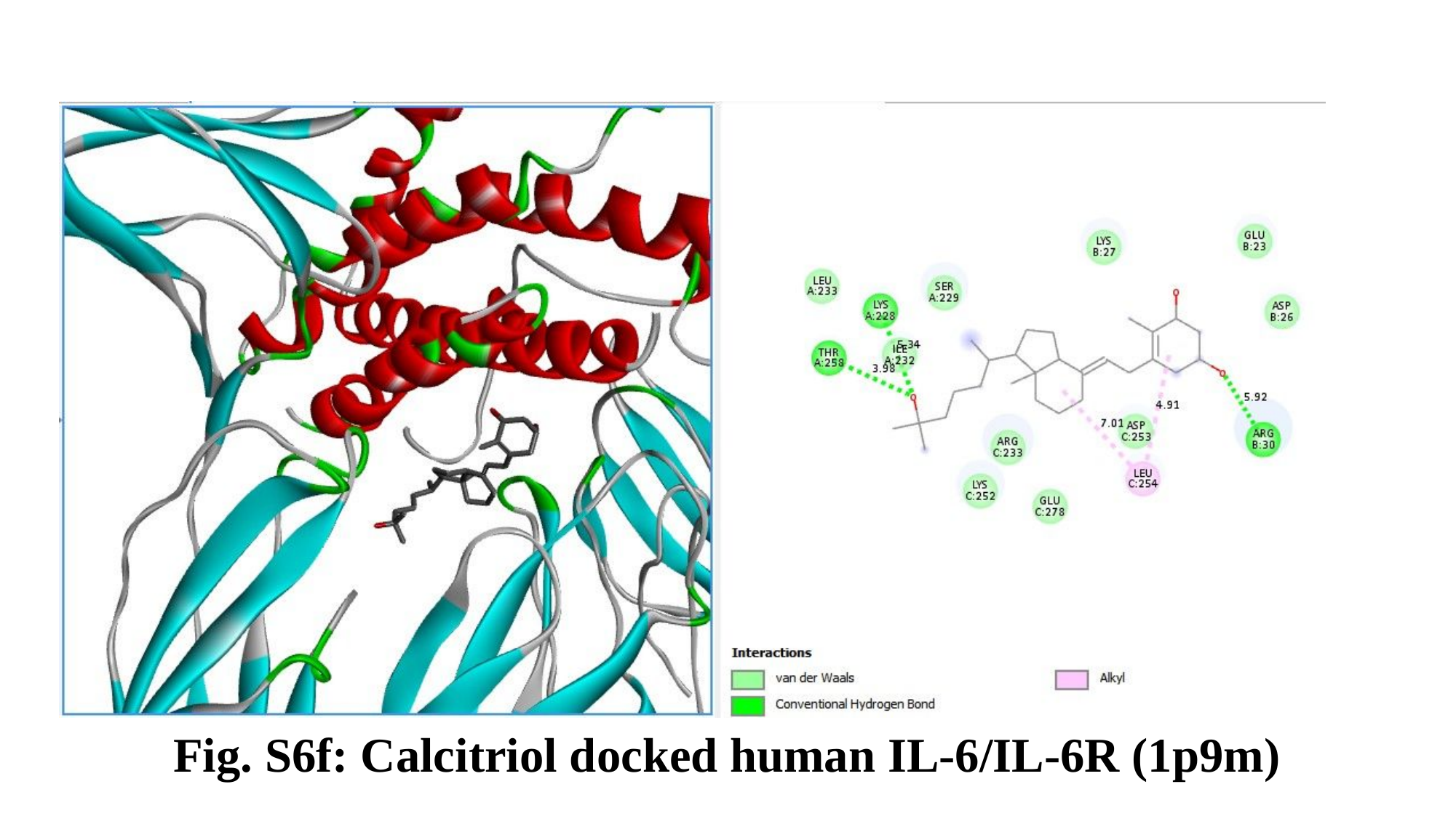

Fig. S6f: Calcitriol docked human IL-6/IL-6R (1p9m)

## Slide 8
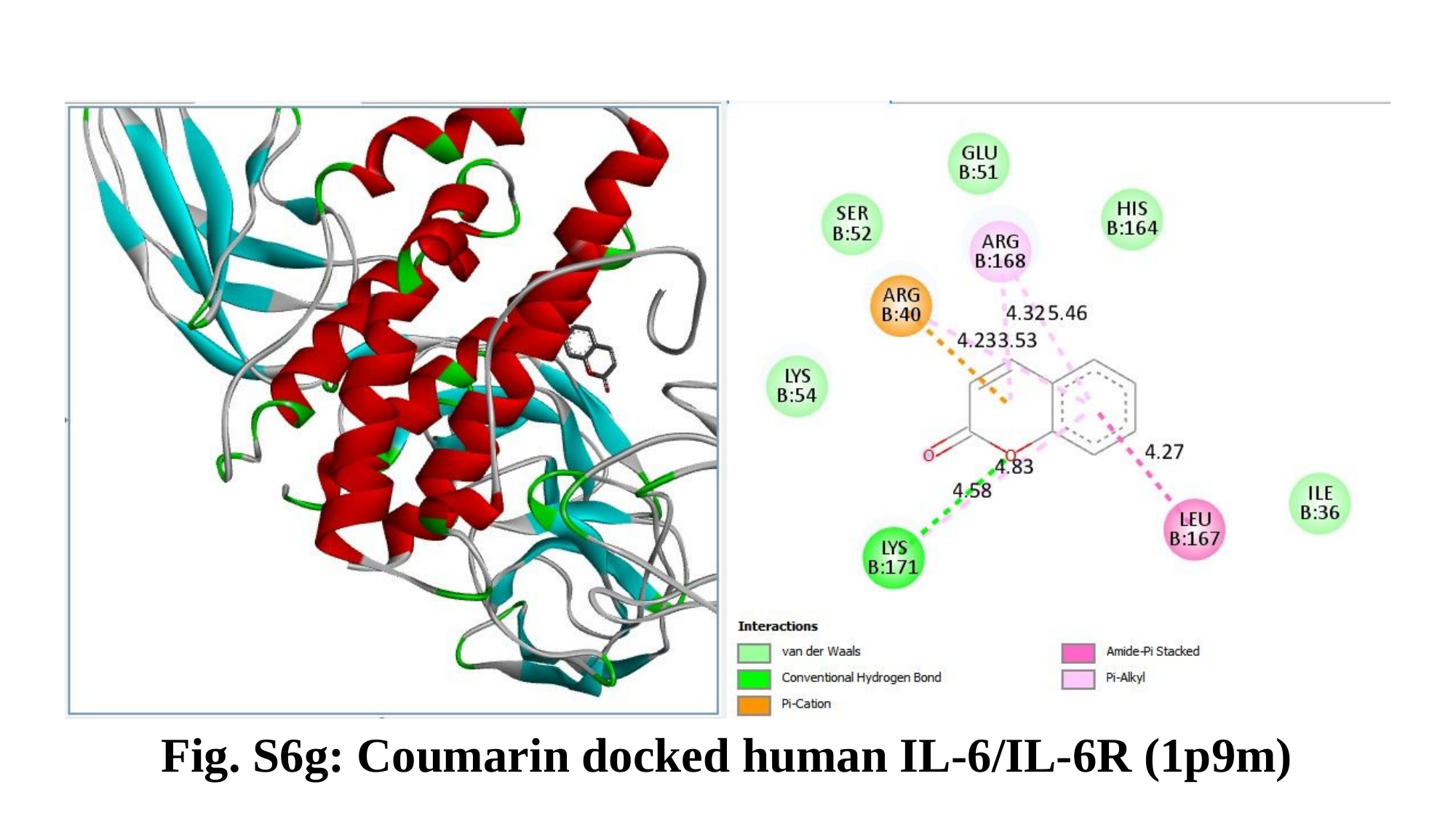

Fig. S6g: Coumarin docked human IL-6/IL-6R (1p9m)

## Slide 9
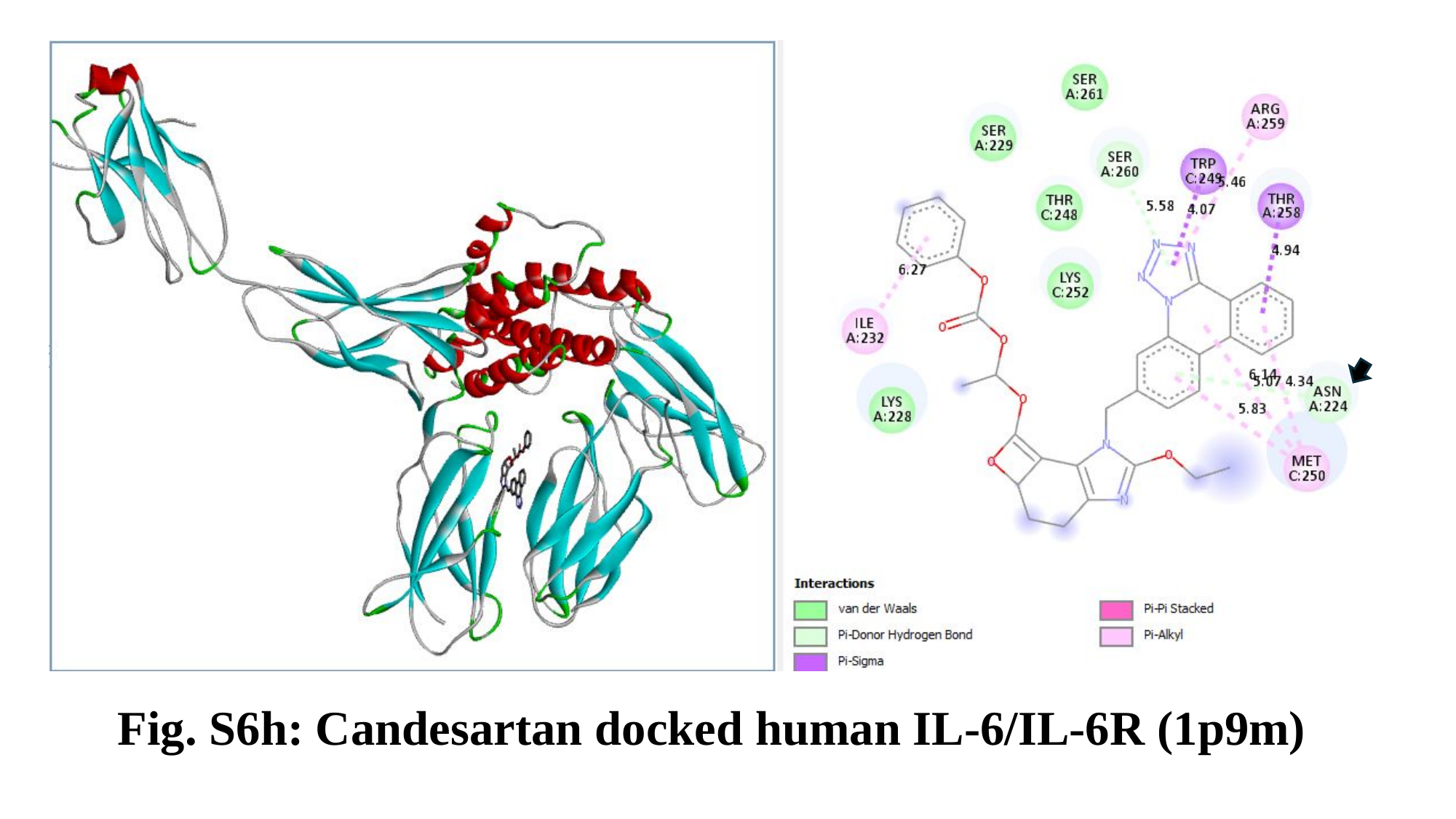

Fig. S6h: Candesartan docked human IL-6/IL-6R (1p9m)

## Slide 10
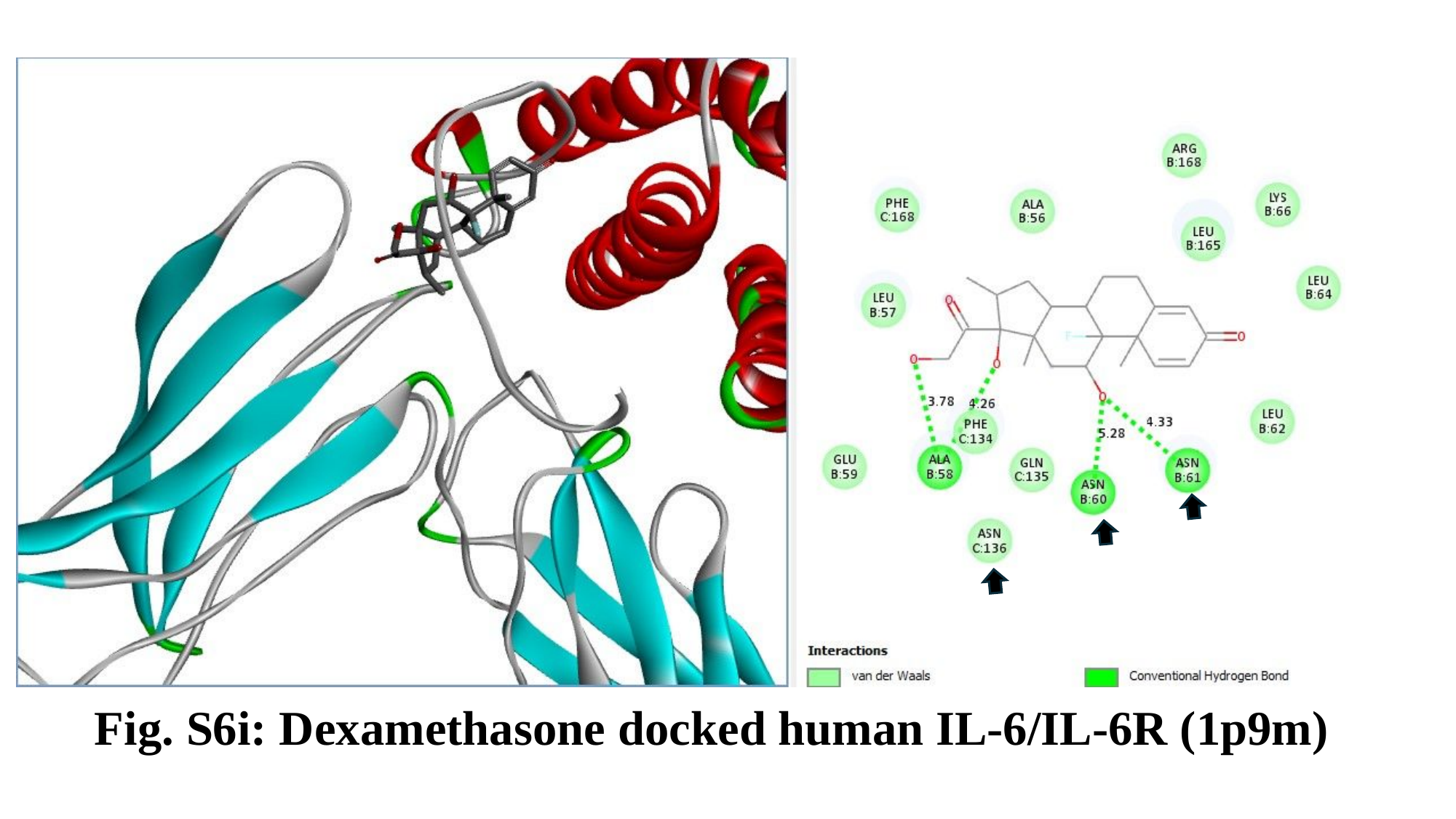

Fig. S6i: Dexamethasone docked human IL-6/IL-6R (1p9m)

## Slide 11
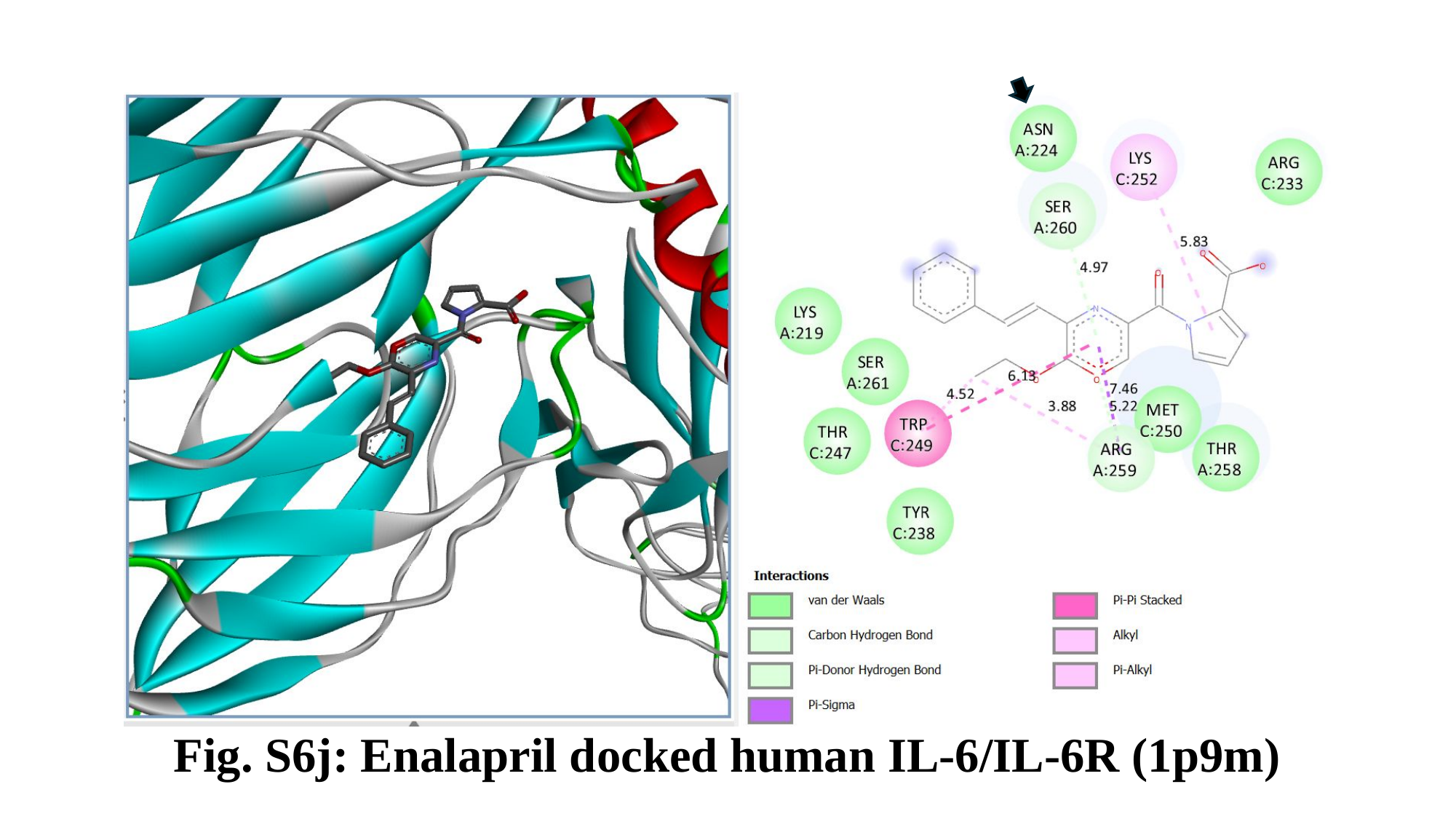

Fig. S6j: Enalapril docked human IL-6/IL-6R (1p9m)

## Slide 12
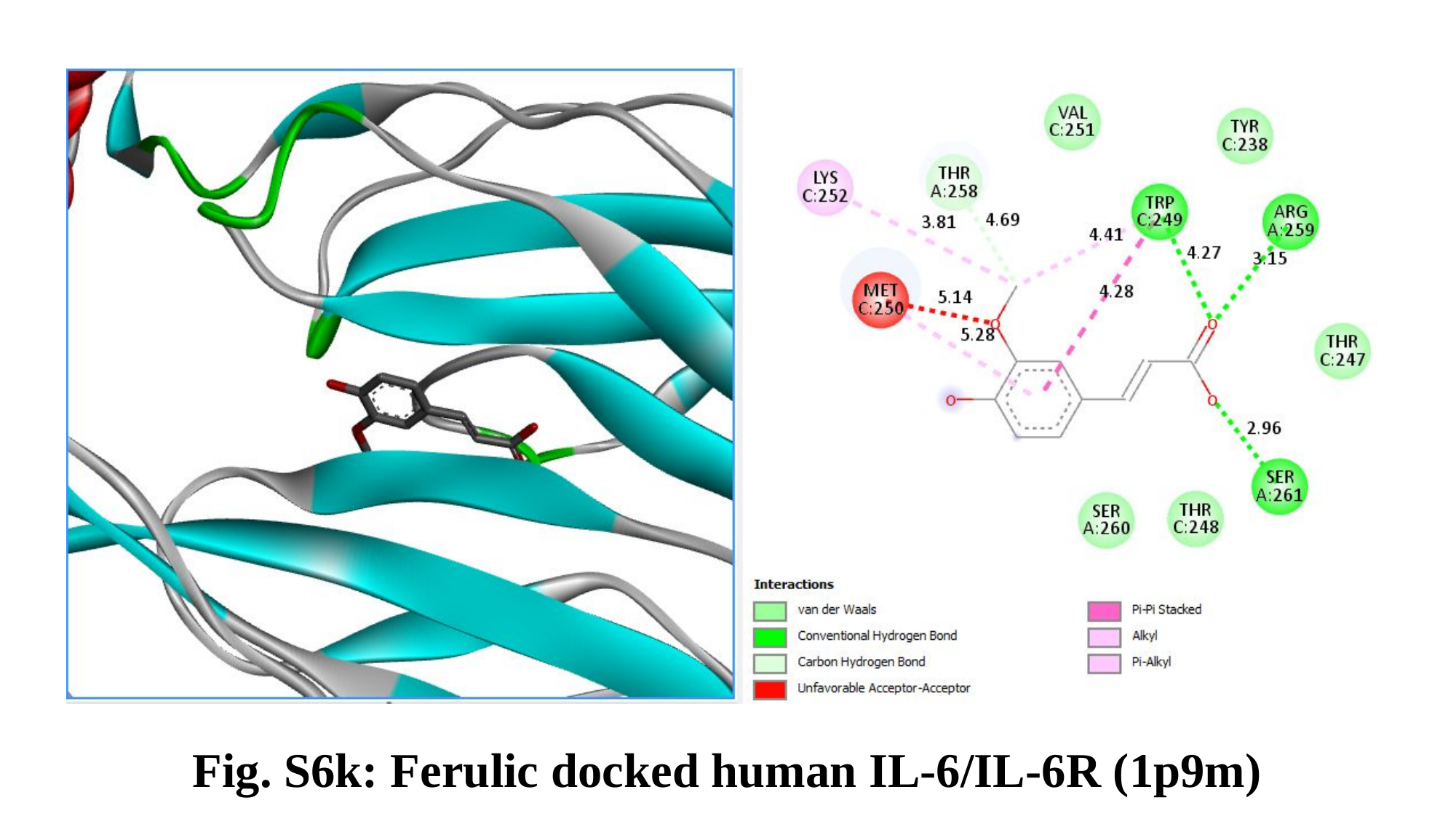

Fig. S6k: Ferulic docked human IL-6/IL-6R (1p9m)

## Slide 13
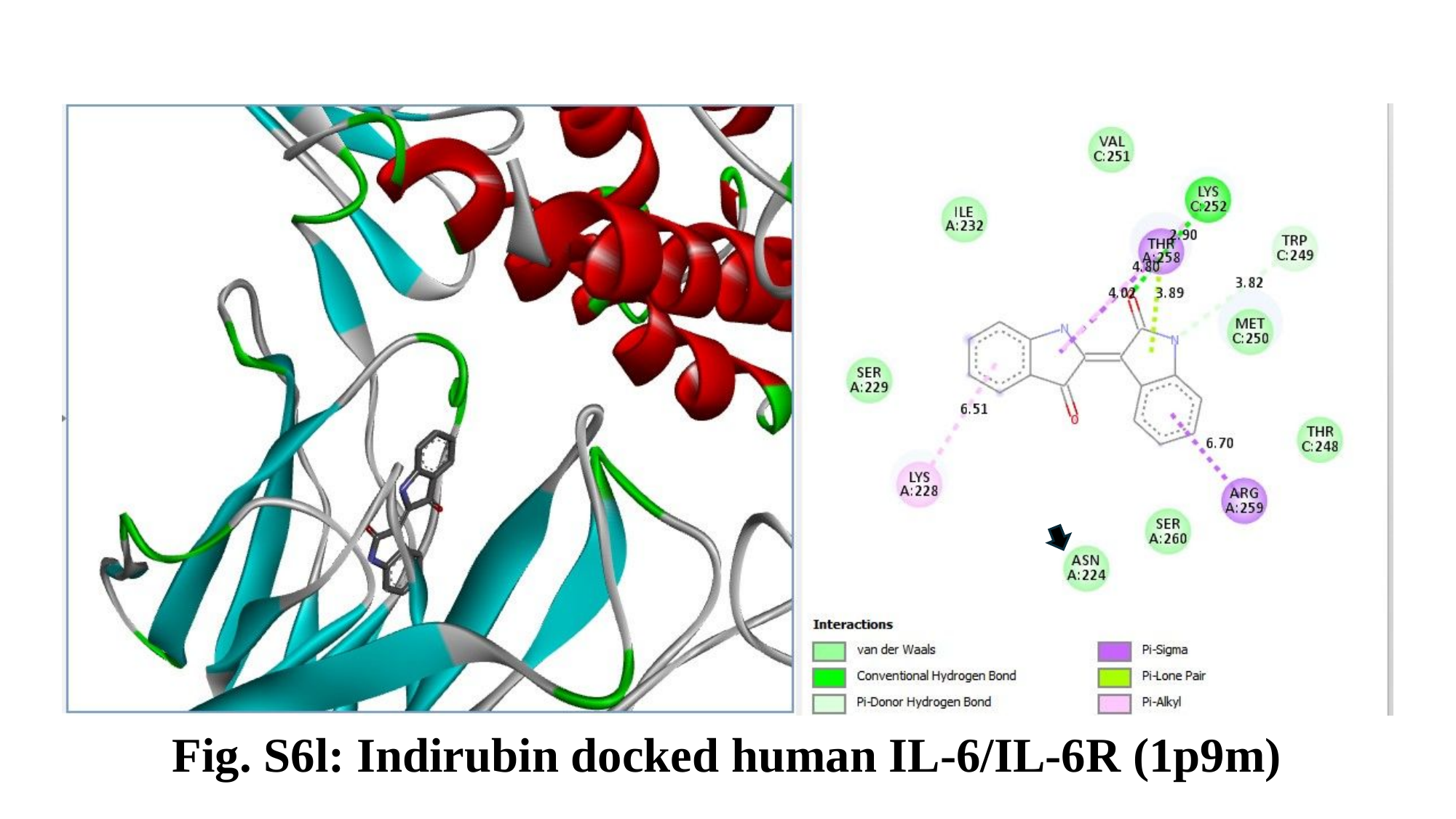

Fig. S6l: Indirubin docked human IL-6/IL-6R (1p9m)

## Slide 14
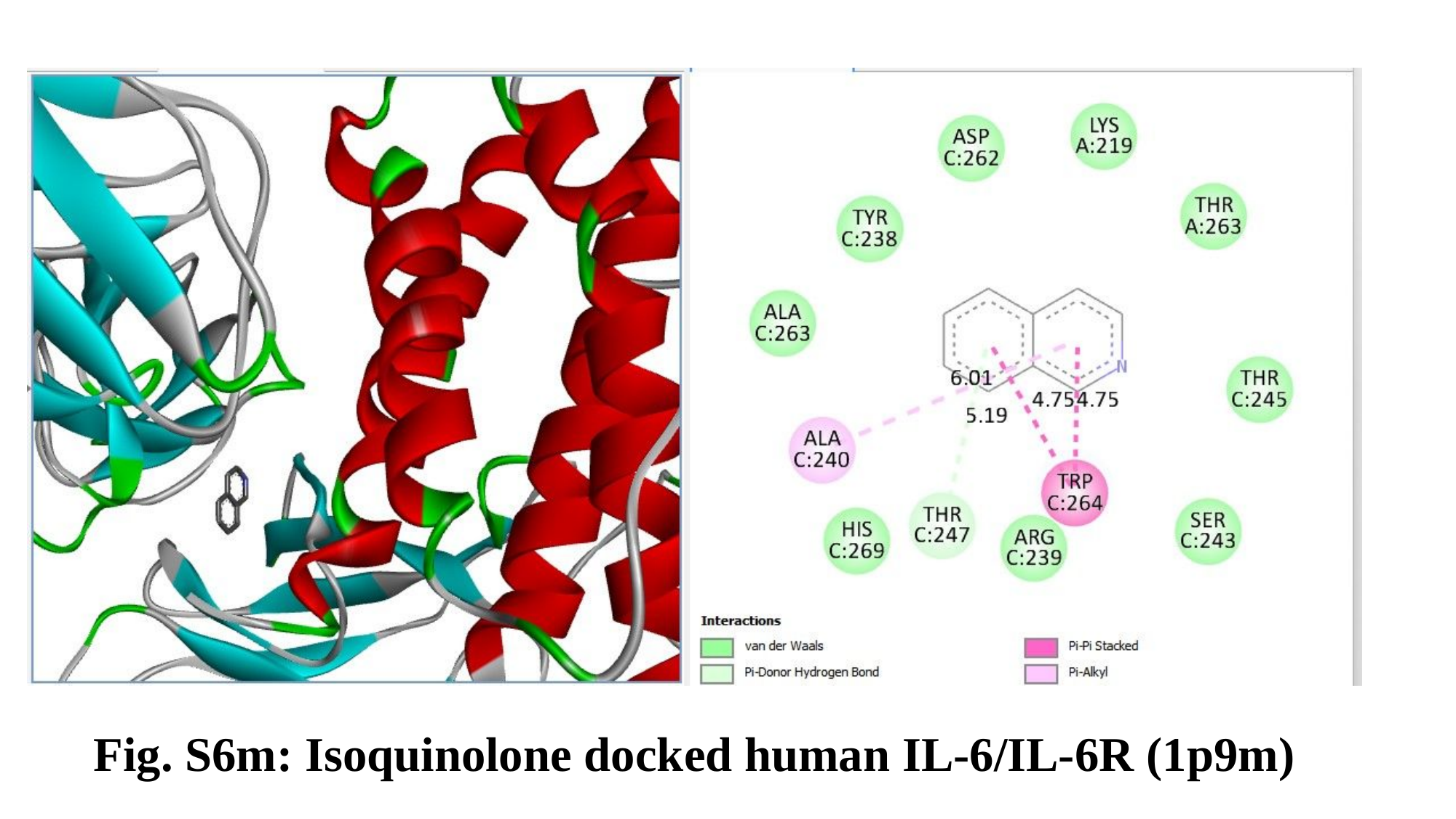

Fig. S6m: Isoquinolone docked human IL-6/IL-6R (1p9m)

## Slide 15
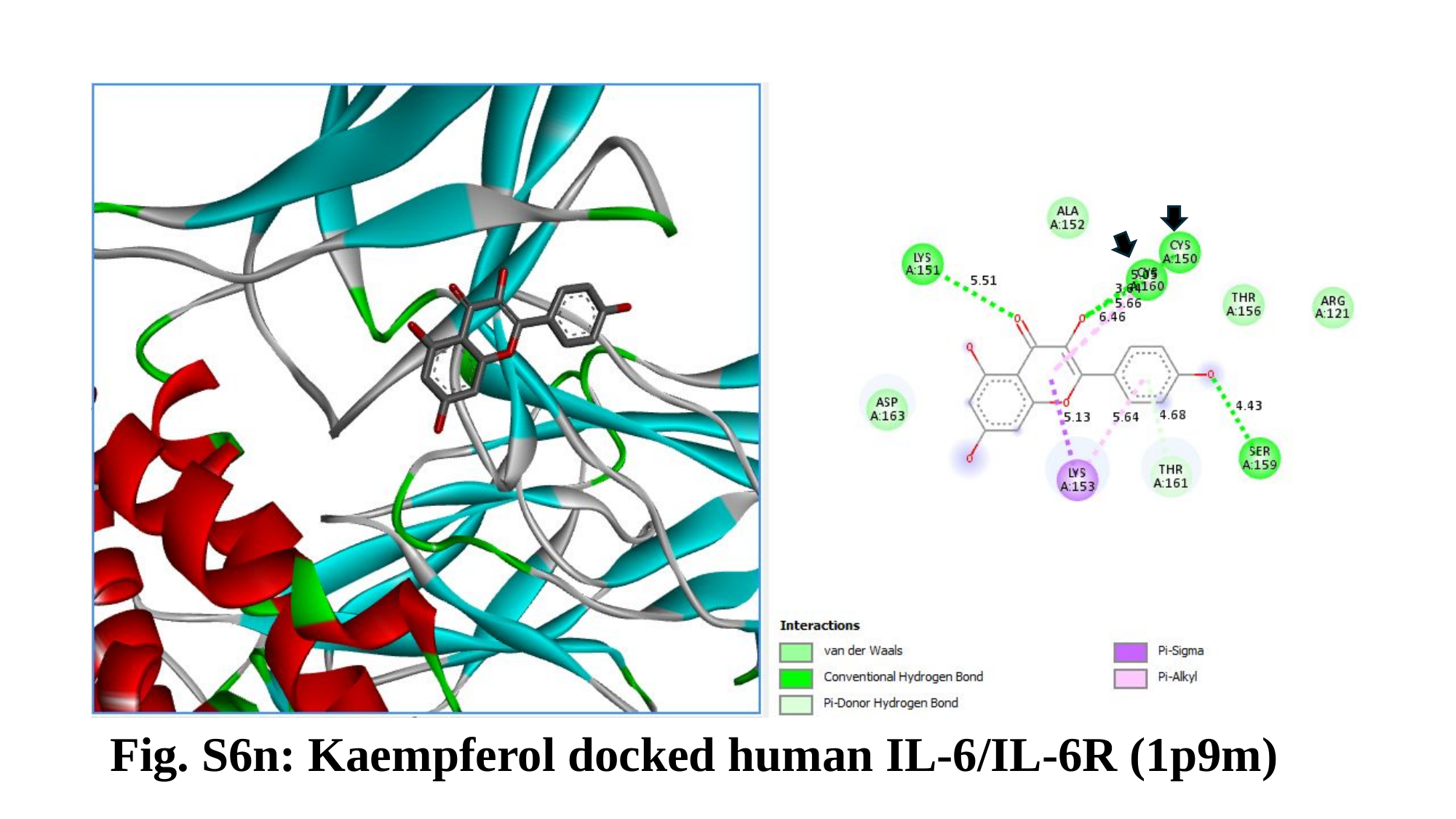

Fig. S6n: Kaempferol docked human IL-6/IL-6R (1p9m)

## Slide 16
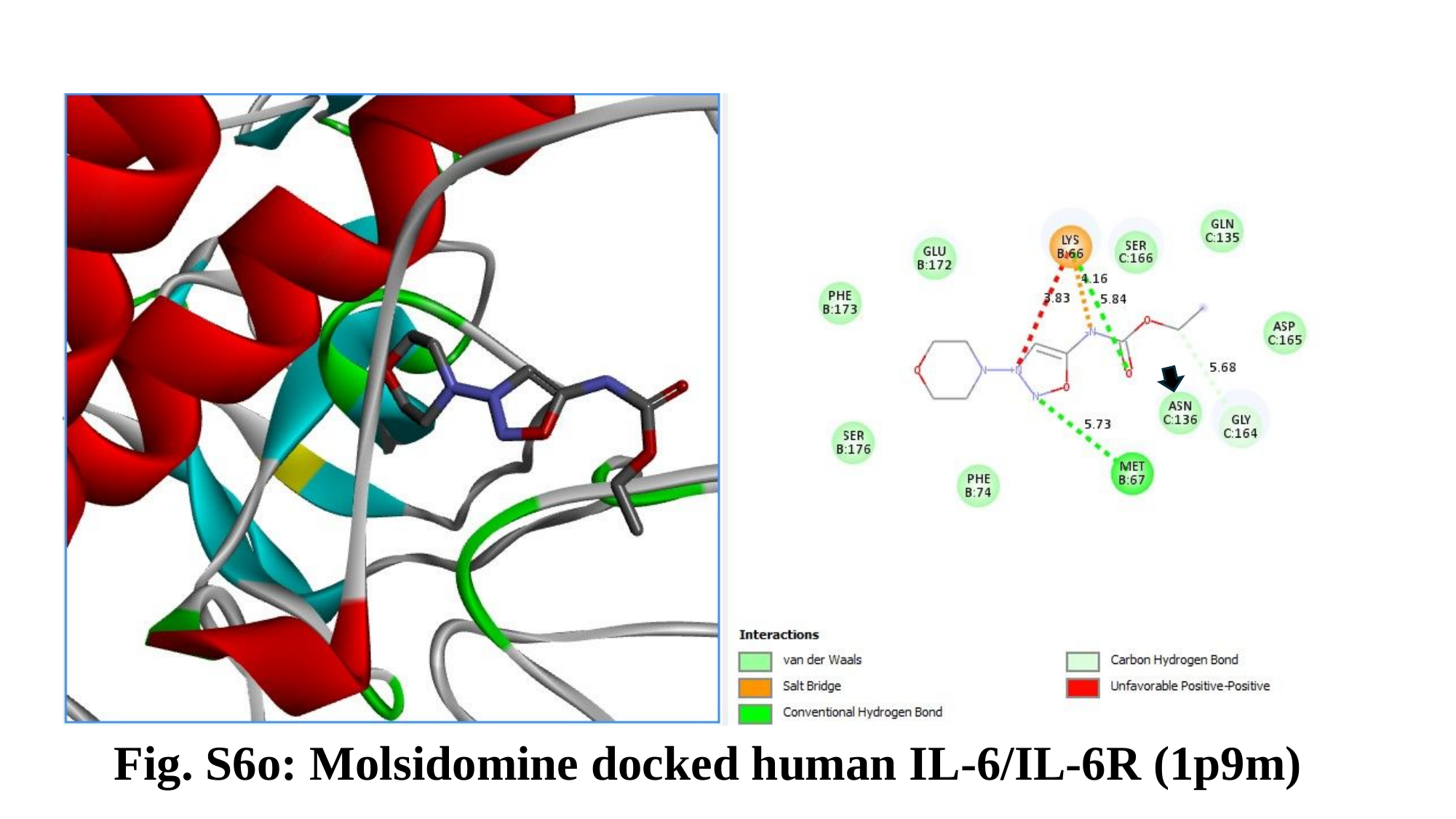

Fig. S6o: Molsidomine docked human IL-6/IL-6R (1p9m)

## Slide 17
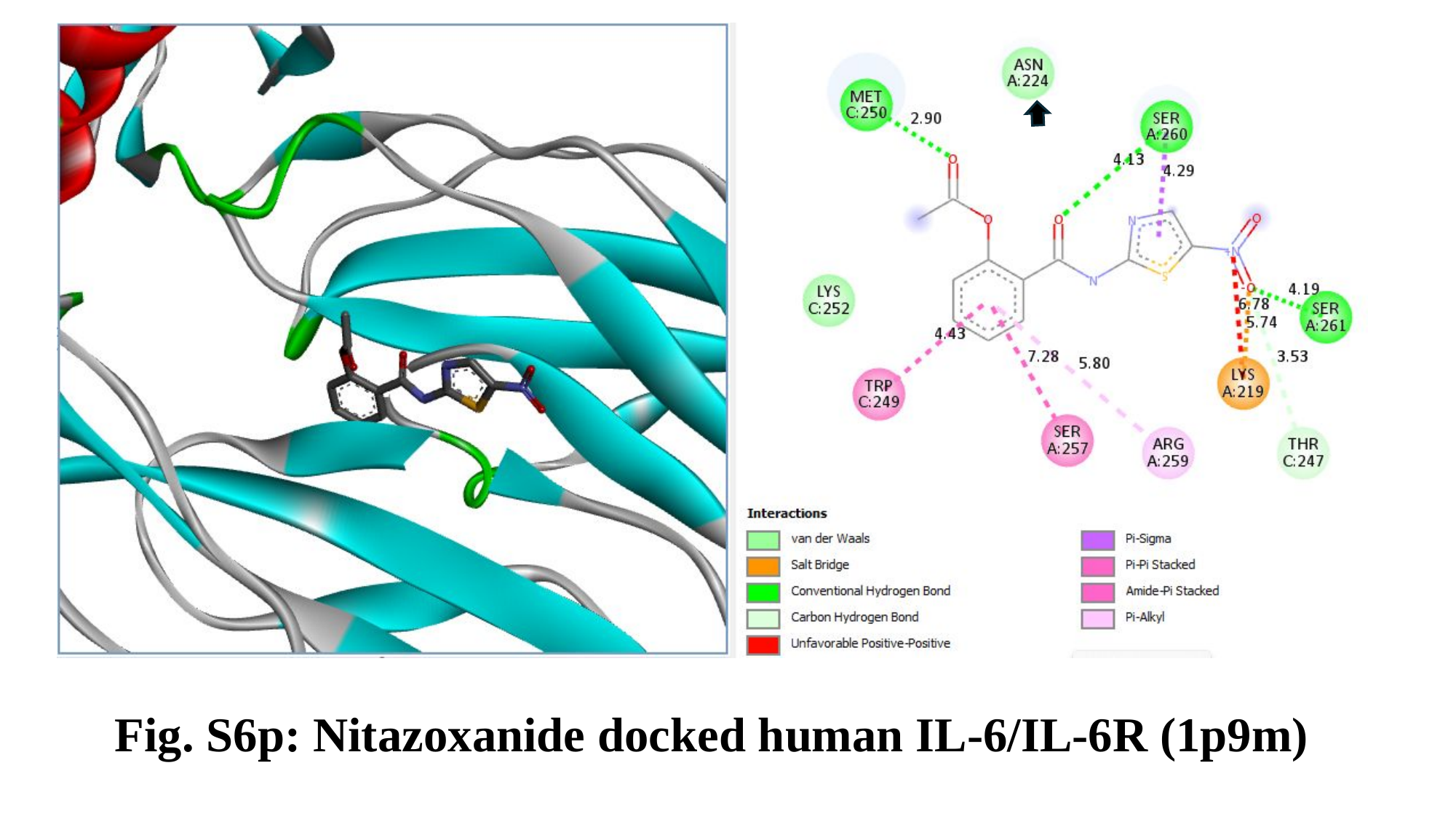

Fig. S6p: Nitazoxanide docked human IL-6/IL-6R (1p9m)

## Slide 18
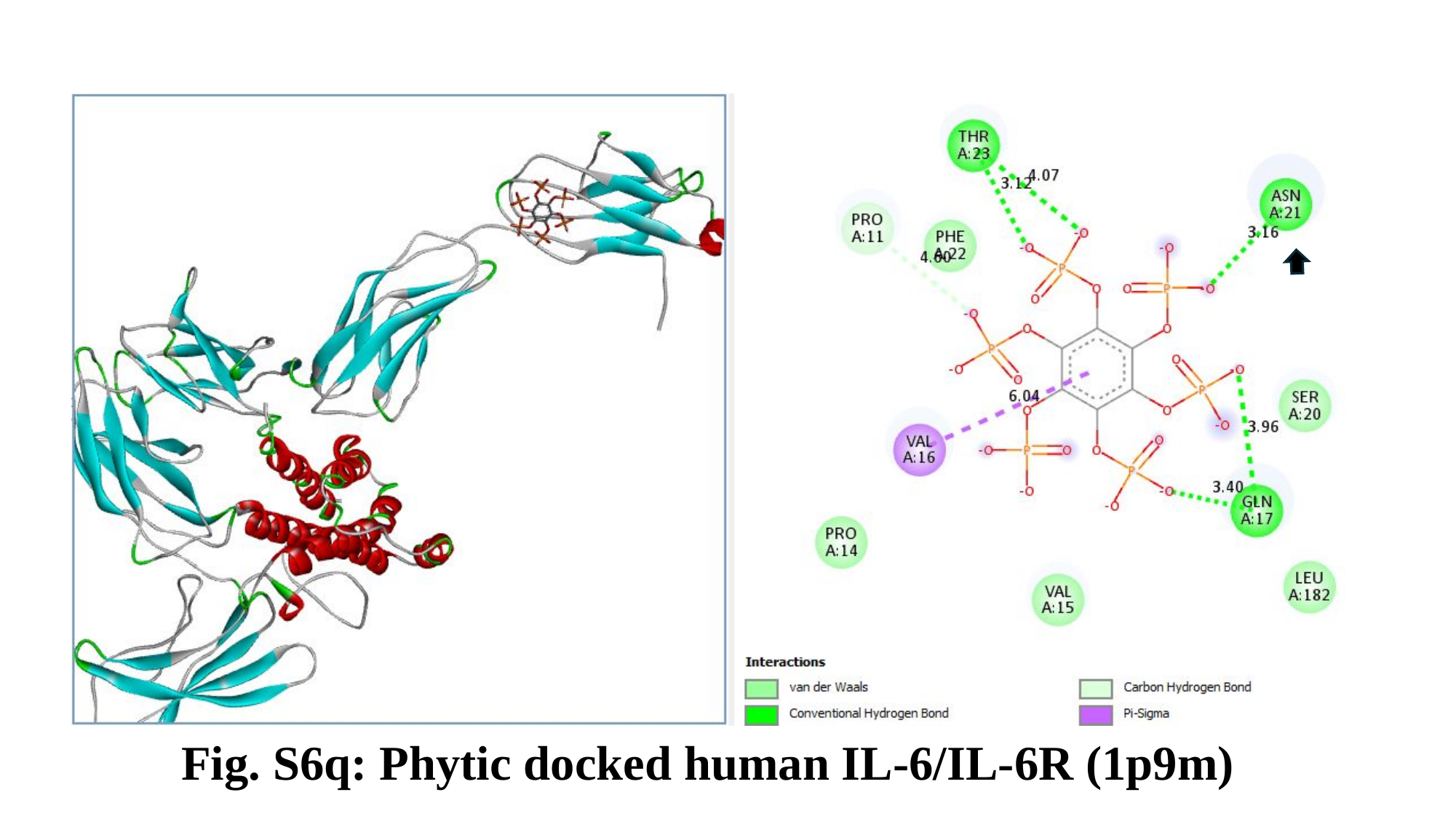

Fig. S6q: Phytic docked human IL-6/IL-6R (1p9m)

## Slide 19
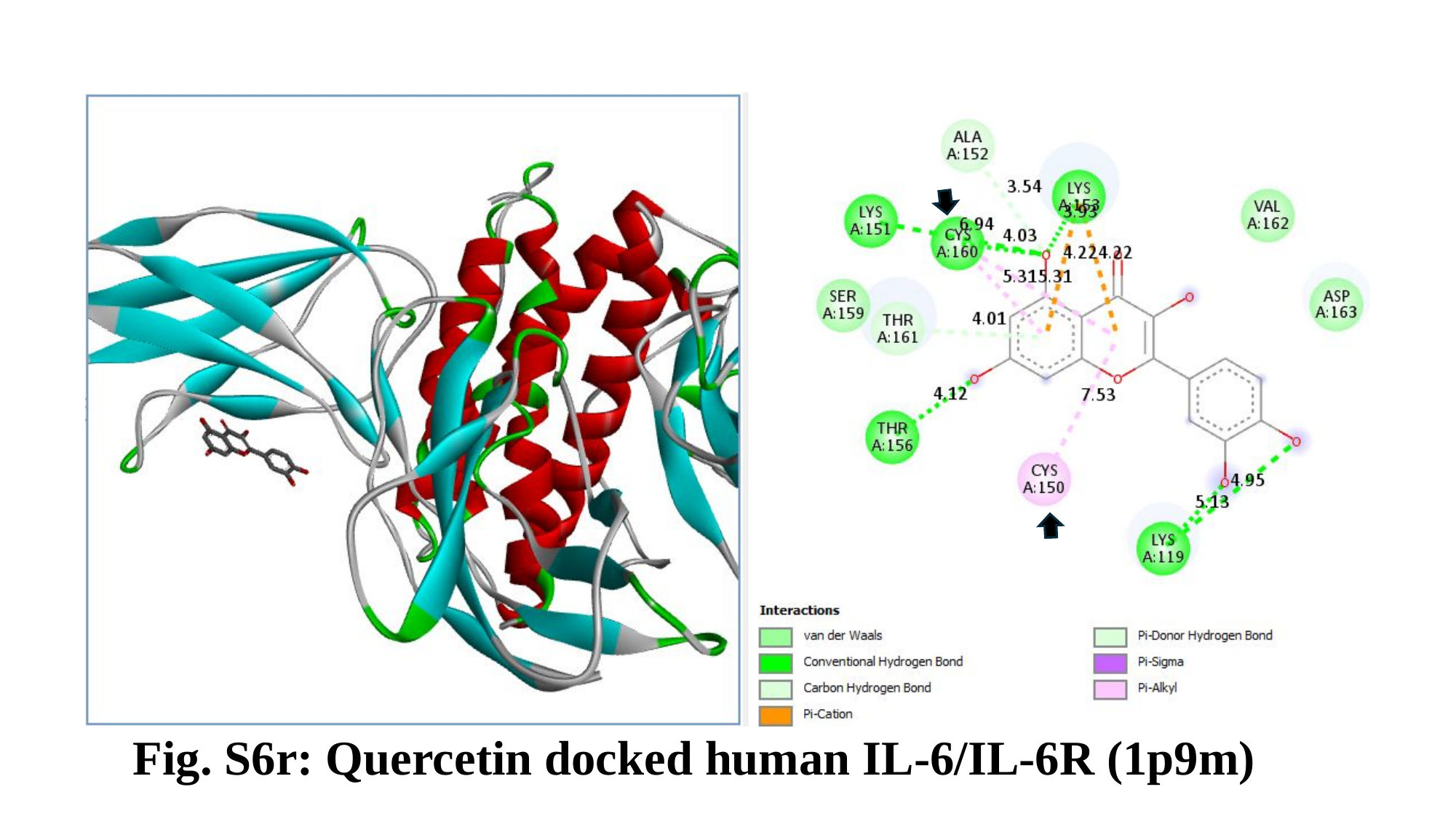

Fig. S6r: Quercetin docked human IL-6/IL-6R (1p9m)

## Slide 20
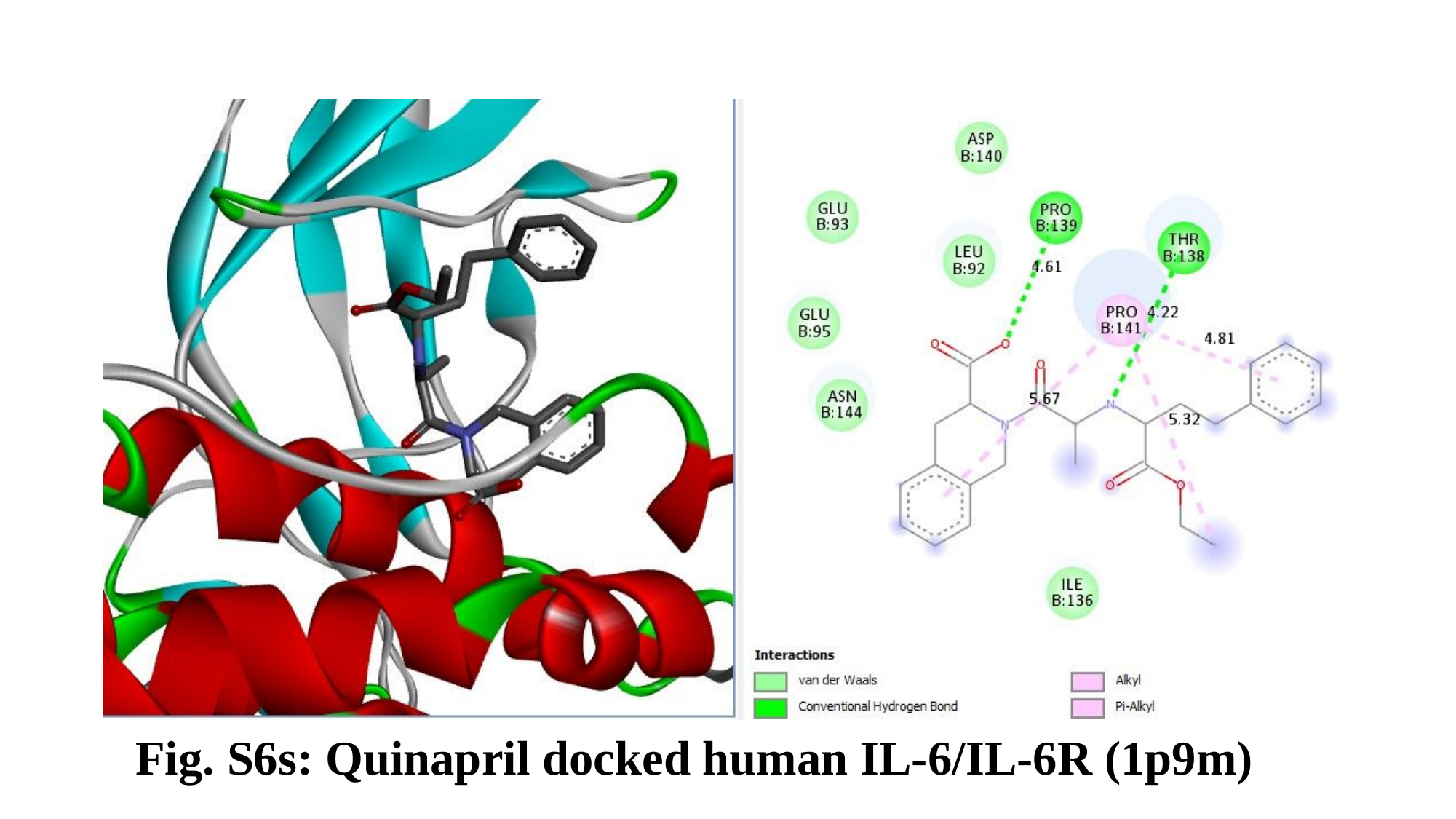

Fig. S6s: Quinapril docked human IL-6/IL-6R (1p9m)

## Slide 21
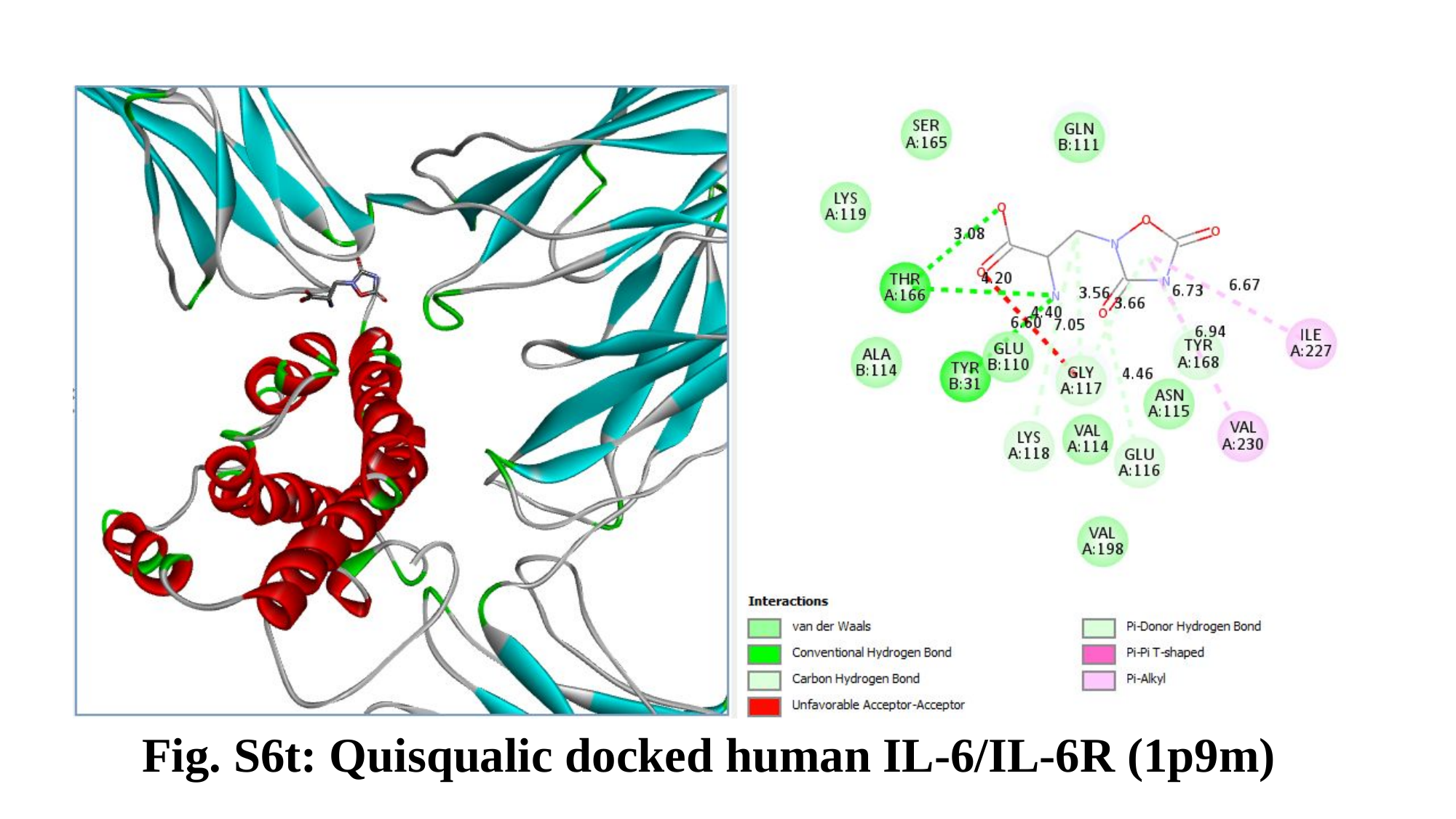

Fig. S6t: Quisqualic docked human IL-6/IL-6R (1p9m)

## Slide 22
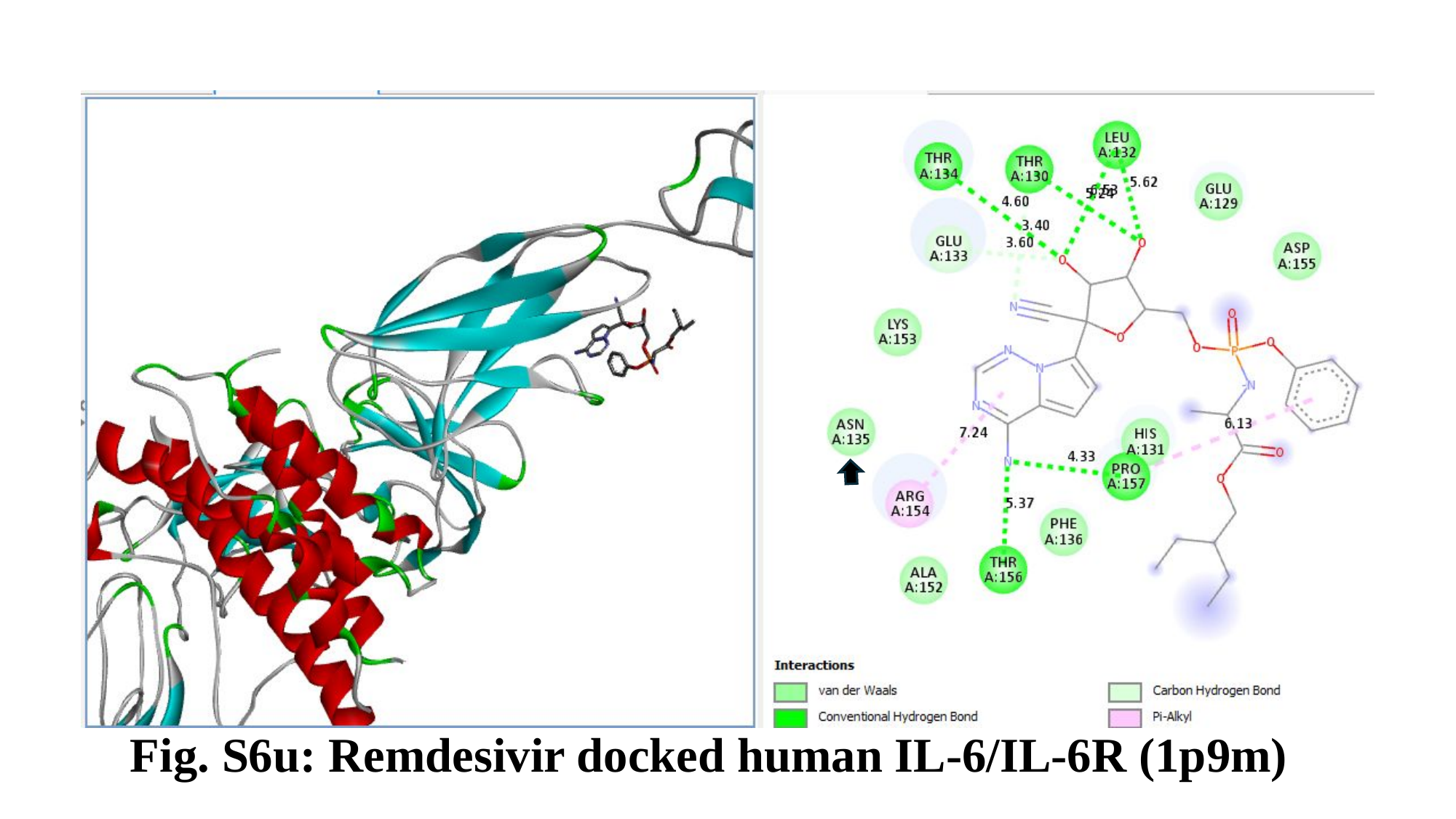

Fig. S6u: Remdesivir docked human IL-6/IL-6R (1p9m)

## Slide 23
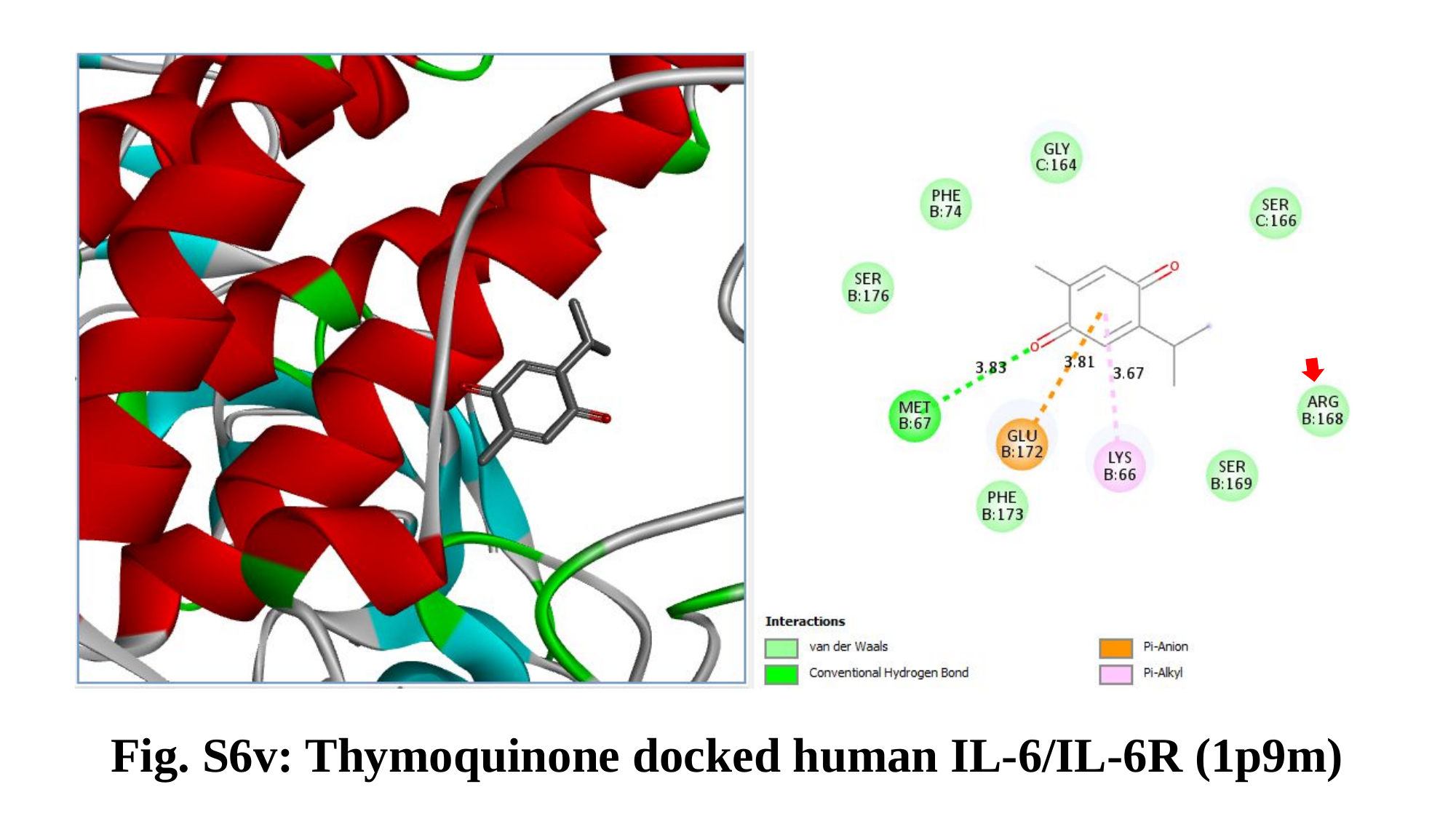

Fig. S6v: Thymoquinone docked human IL-6/IL-6R (1p9m)

## Slide 24
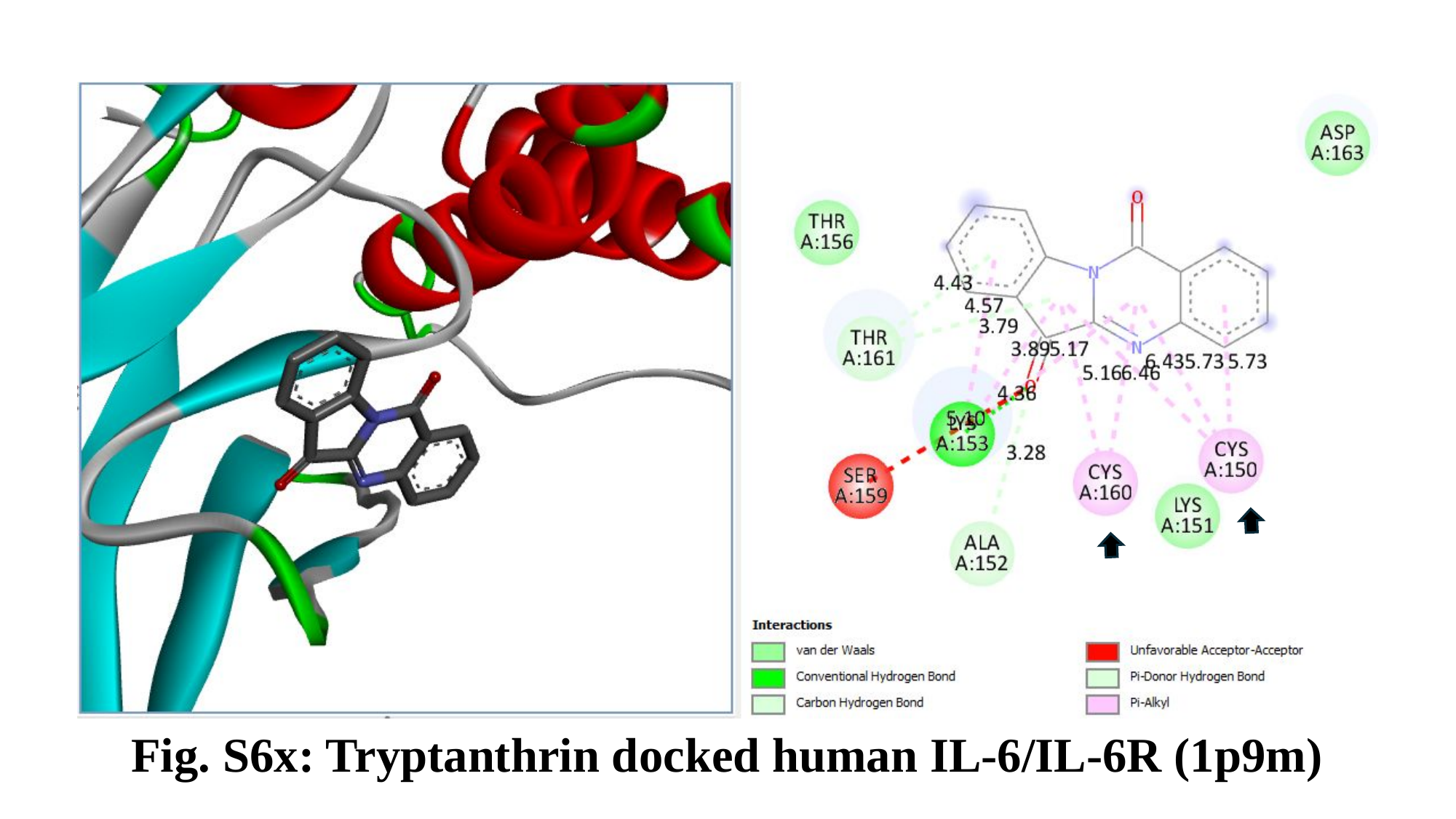

Fig. S6x: Tryptanthrin docked human IL-6/IL-6R (1p9m)
