# Supplementary material for: Targeting asparagine and cysteine in SARS-CoV-2 variants and human pro-inflammatory mediators to alleviate COVID-19 severity; a cross-section and in-silico study
Source: Sci Rep. 2025 Nov 3;15:38445. doi: 10.1038/s41598-025-19359-y (PMC12583749; doi:10.1038/s41598-025-19359-y)
Supplement: Supplementary file 14 — Supplementary Material 14 [file 41598_2025_19359_MOESM14_ESM.pptx]

## Slide 1
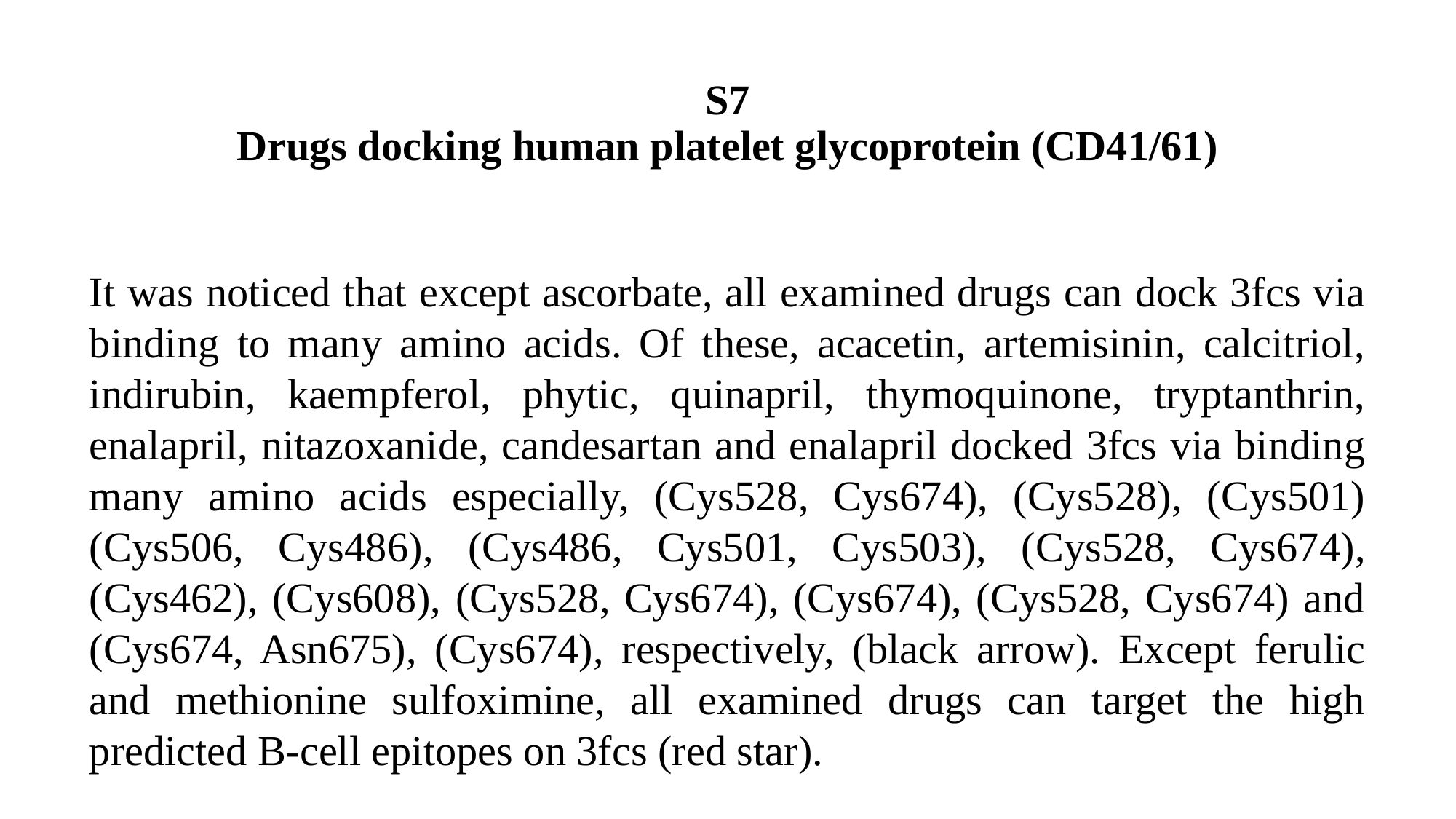

# S7 Drugs docking human platelet glycoprotein (CD41/61)
It was noticed that except ascorbate, all examined drugs can dock 3fcs via binding to many amino acids. Of these, acacetin, artemisinin, calcitriol, indirubin, kaempferol, phytic, quinapril, thymoquinone, tryptanthrin, enalapril, nitazoxanide, candesartan and enalapril docked 3fcs via binding many amino acids especially, (Cys528, Cys674), (Cys528), (Cys501) (Cys506, Cys486), (Cys486, Cys501, Cys503), (Cys528, Cys674), (Cys462), (Cys608), (Cys528, Cys674), (Cys674), (Cys528, Cys674) and (Cys674, Asn675), (Cys674), respectively, (black arrow). Except ferulic and methionine sulfoximine, all examined drugs can target the high predicted B-cell epitopes on 3fcs (red star).

## Slide 2
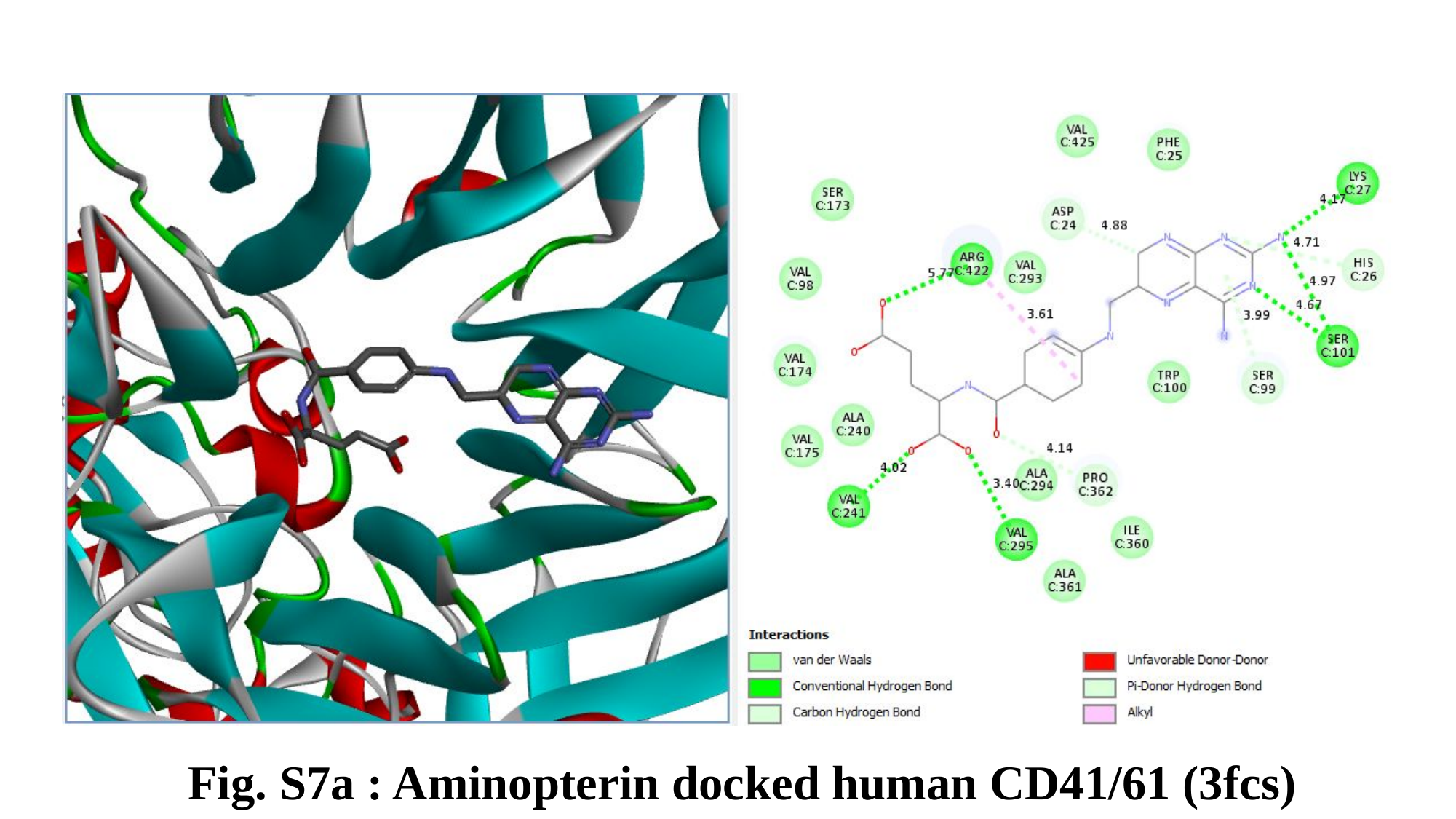

Fig. S7a : Aminopterin docked human CD41/61 (3fcs)

## Slide 3
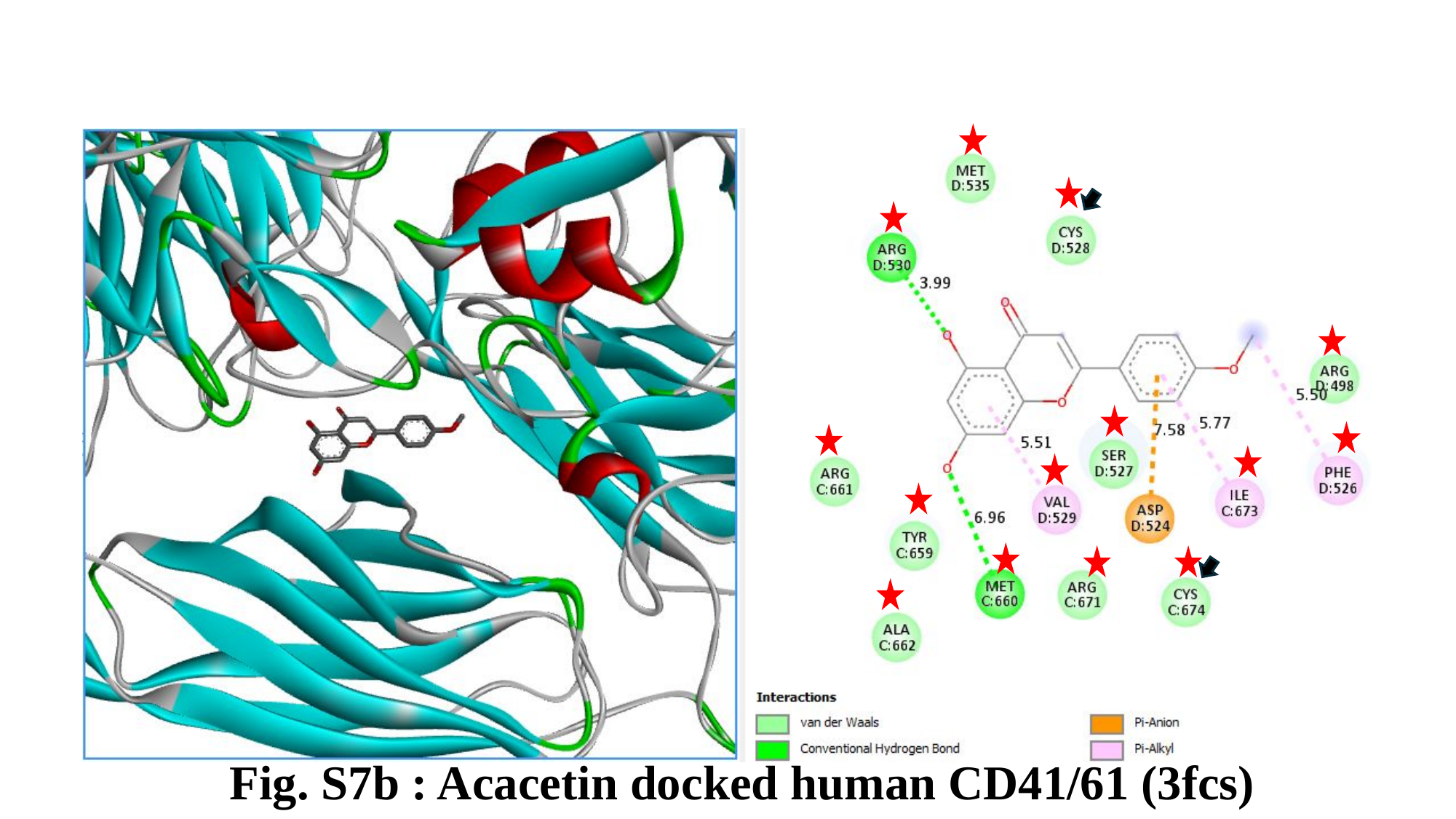

Fig. S7b : Acacetin docked human CD41/61 (3fcs)

## Slide 4
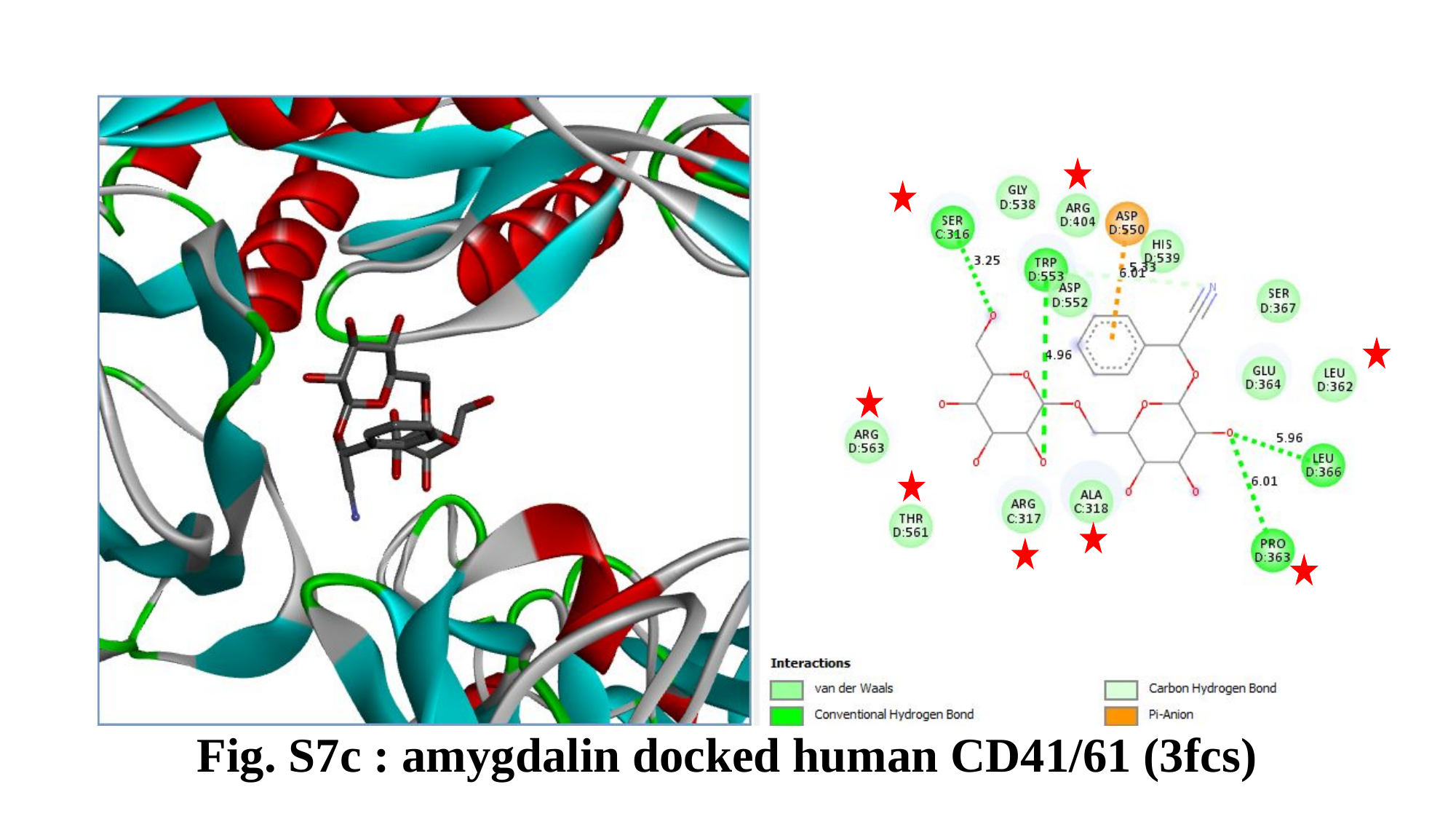

Fig. S7c : amygdalin docked human CD41/61 (3fcs)

## Slide 5
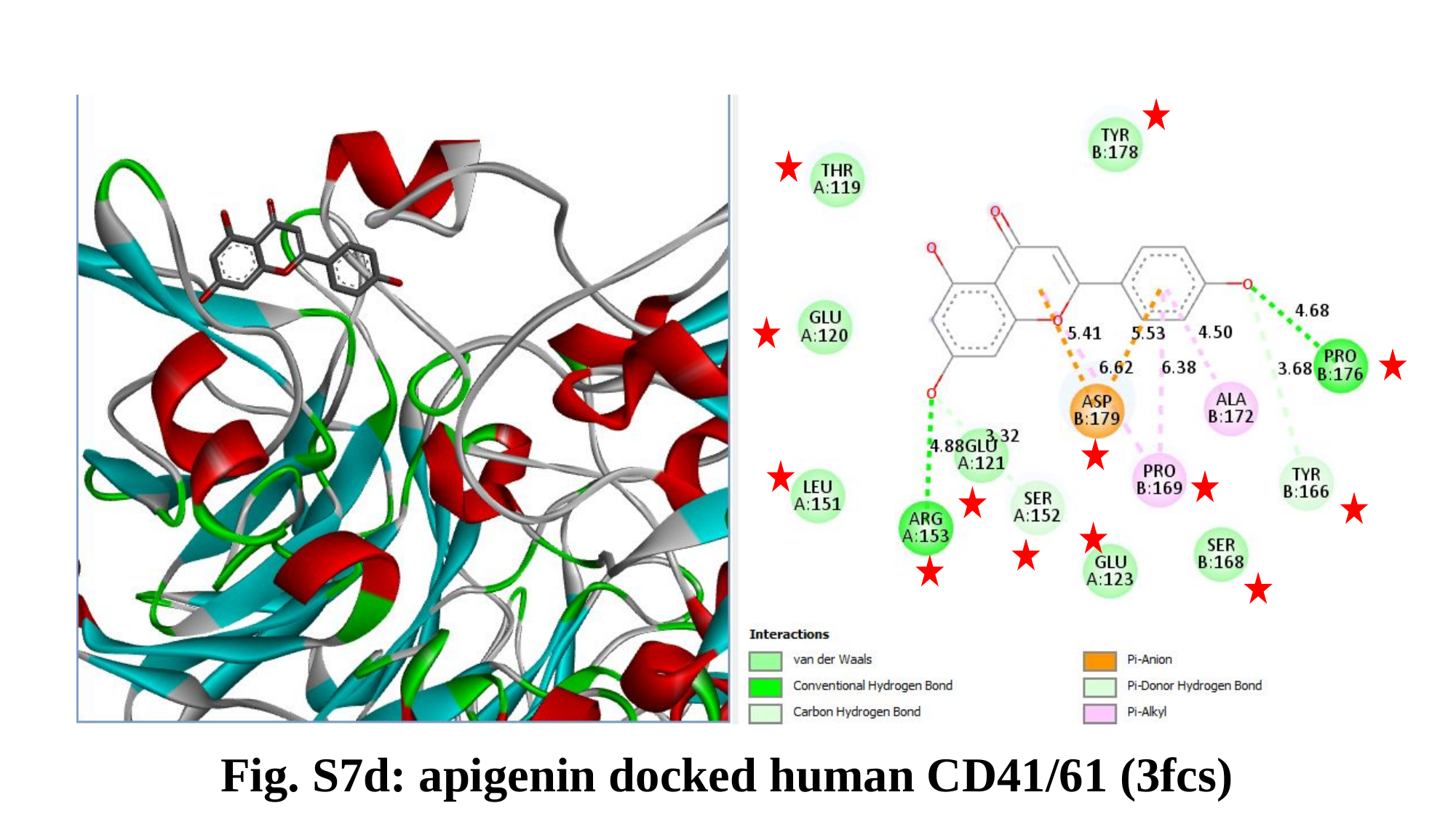

Fig. S7d: apigenin docked human CD41/61 (3fcs)

## Slide 6
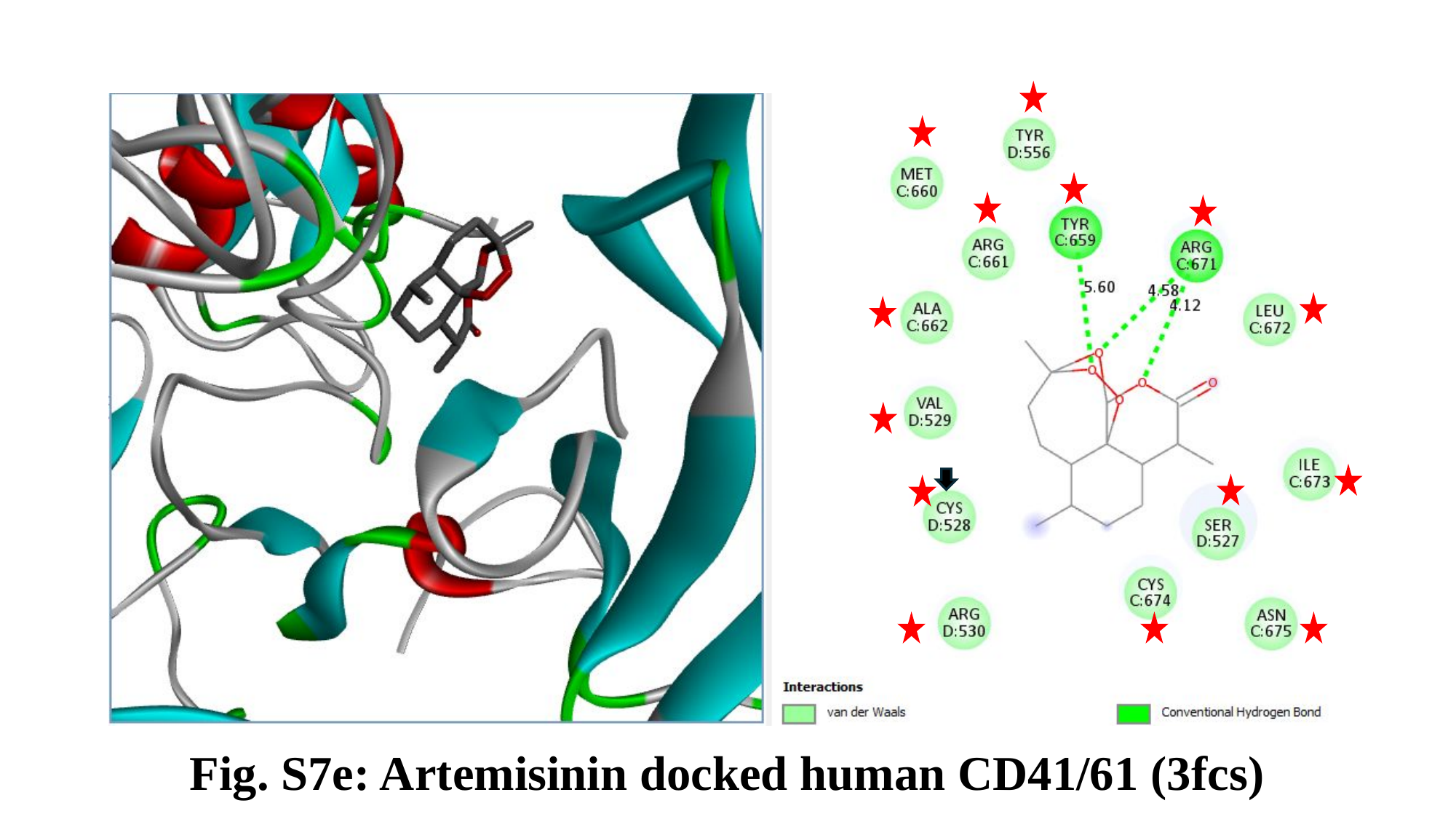

Fig. S7e: Artemisinin docked human CD41/61 (3fcs)

## Slide 7
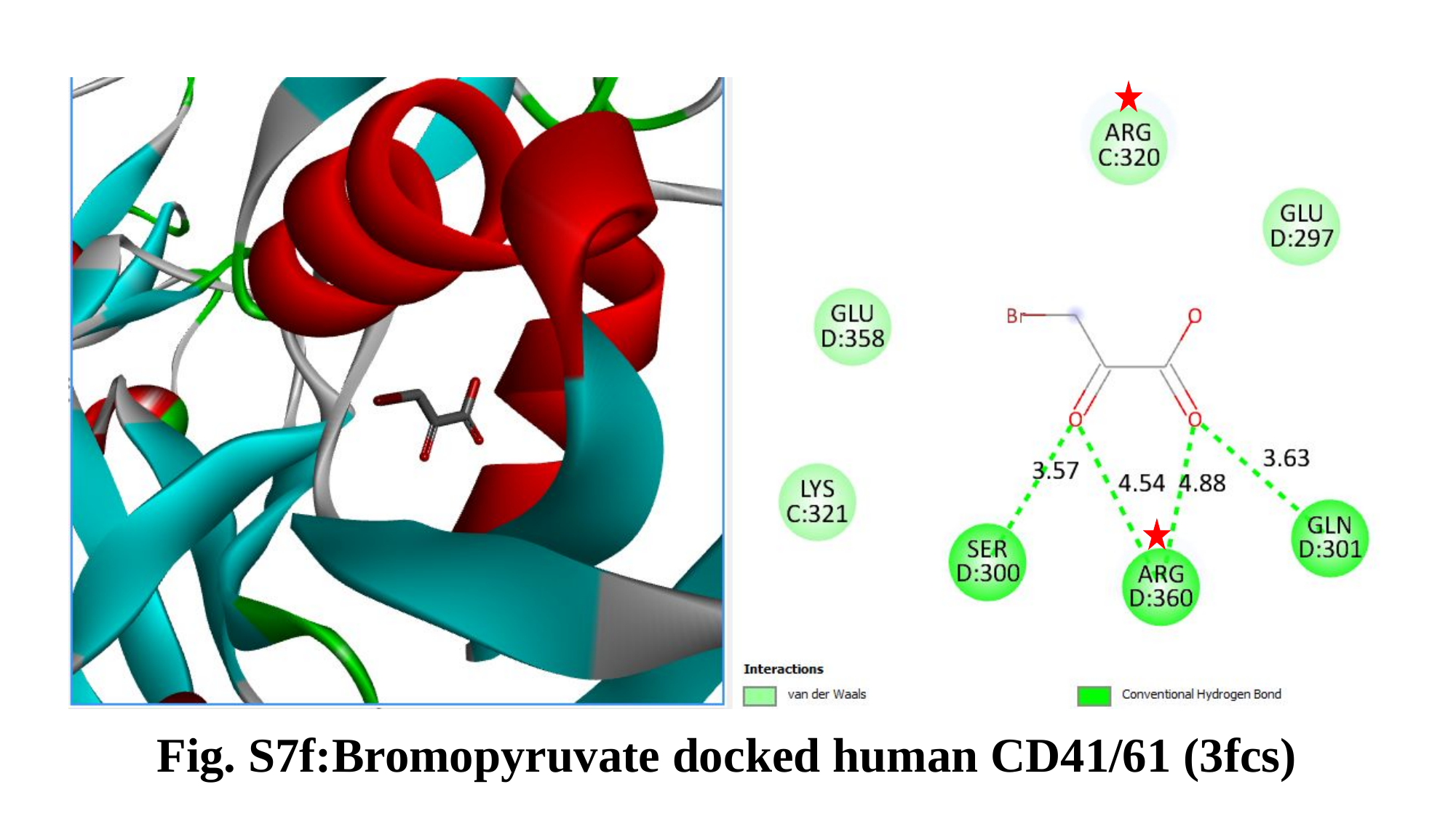

Fig. S7f:Bromopyruvate docked human CD41/61 (3fcs)

## Slide 8
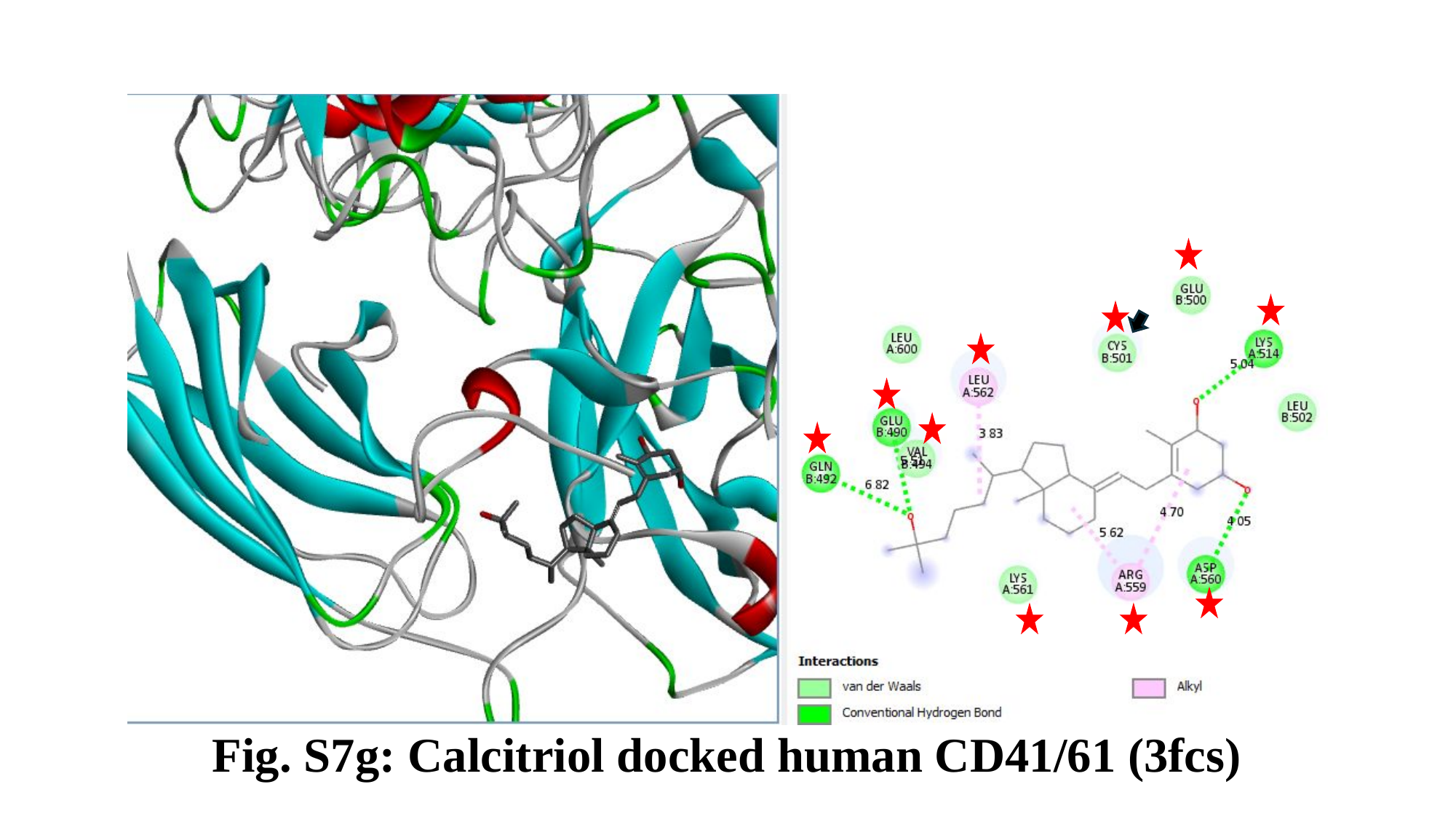

Fig. S7g: Calcitriol docked human CD41/61 (3fcs)

## Slide 9
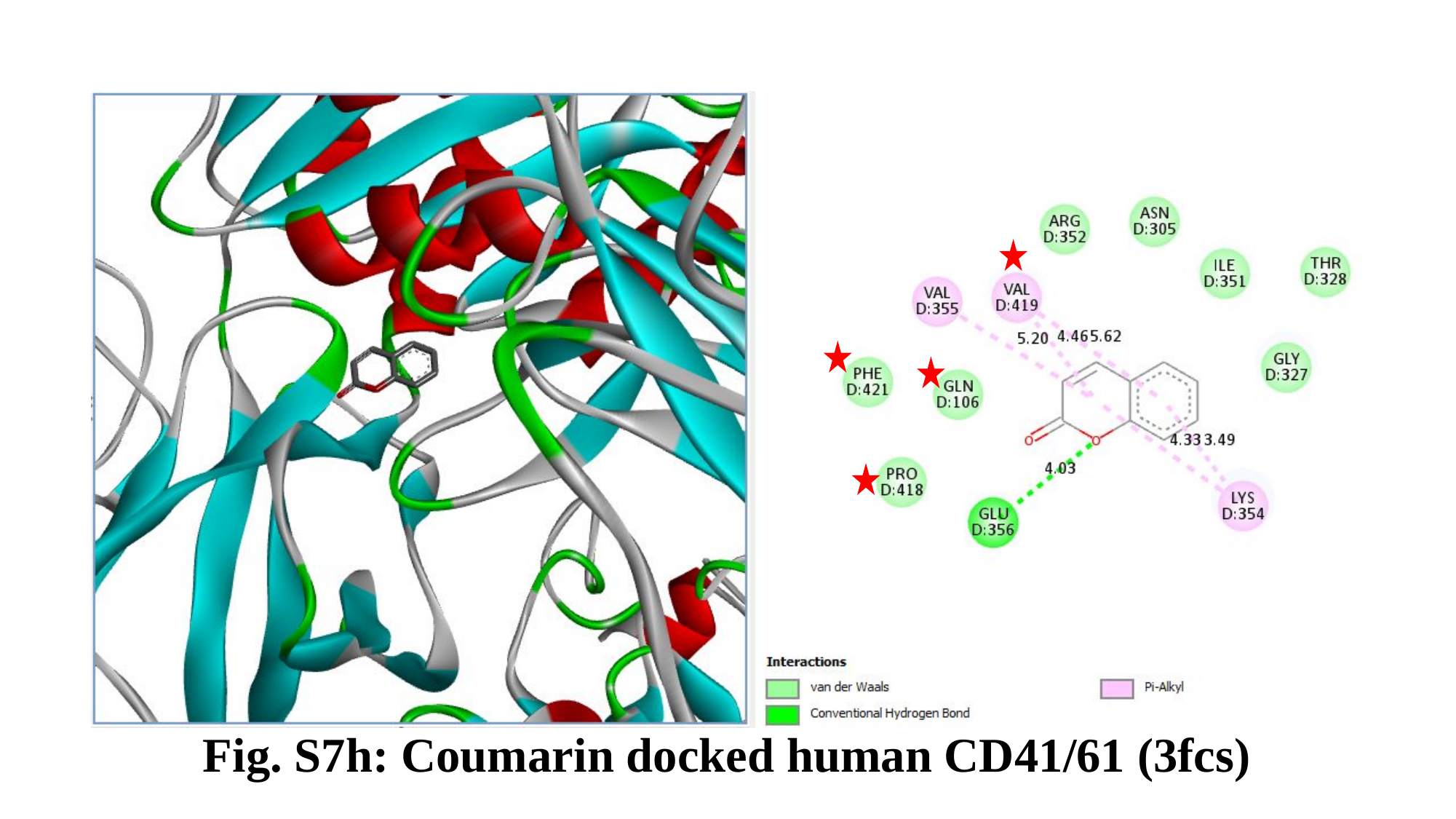

Fig. S7h: Coumarin docked human CD41/61 (3fcs)

## Slide 10
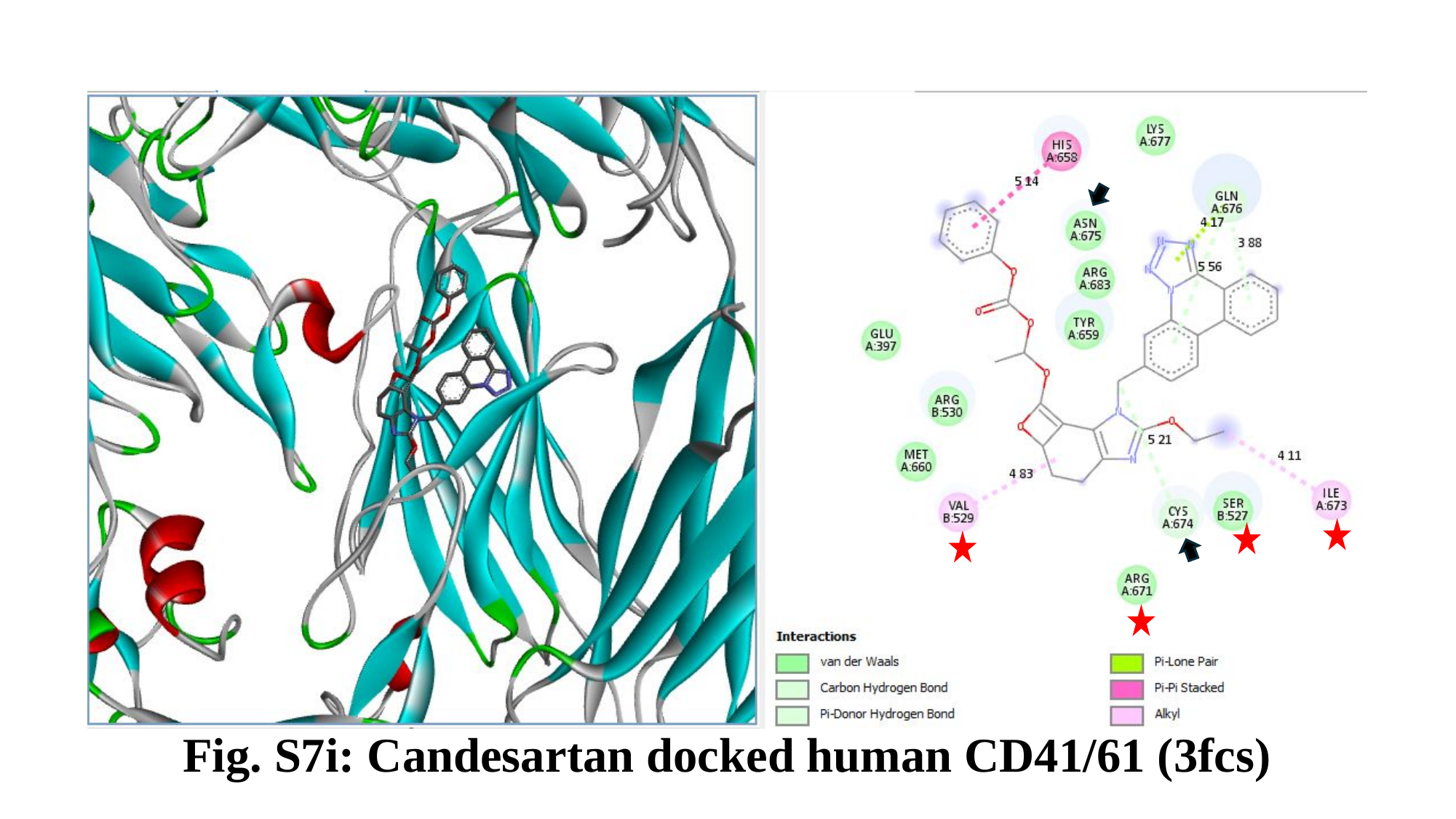

Fig. S7i: Candesartan docked human CD41/61 (3fcs)

## Slide 11
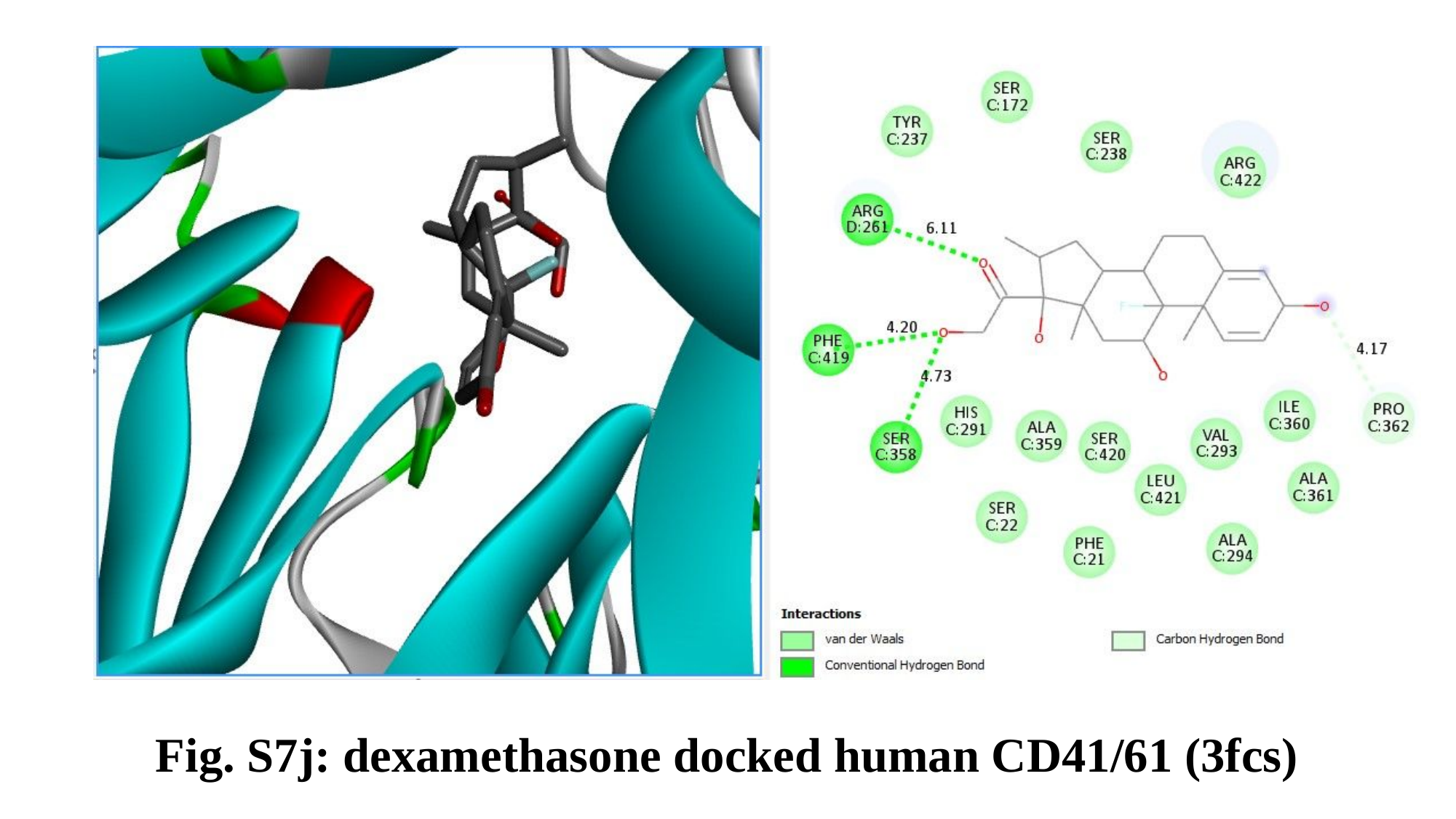

Fig. S7j: dexamethasone docked human CD41/61 (3fcs)

## Slide 12
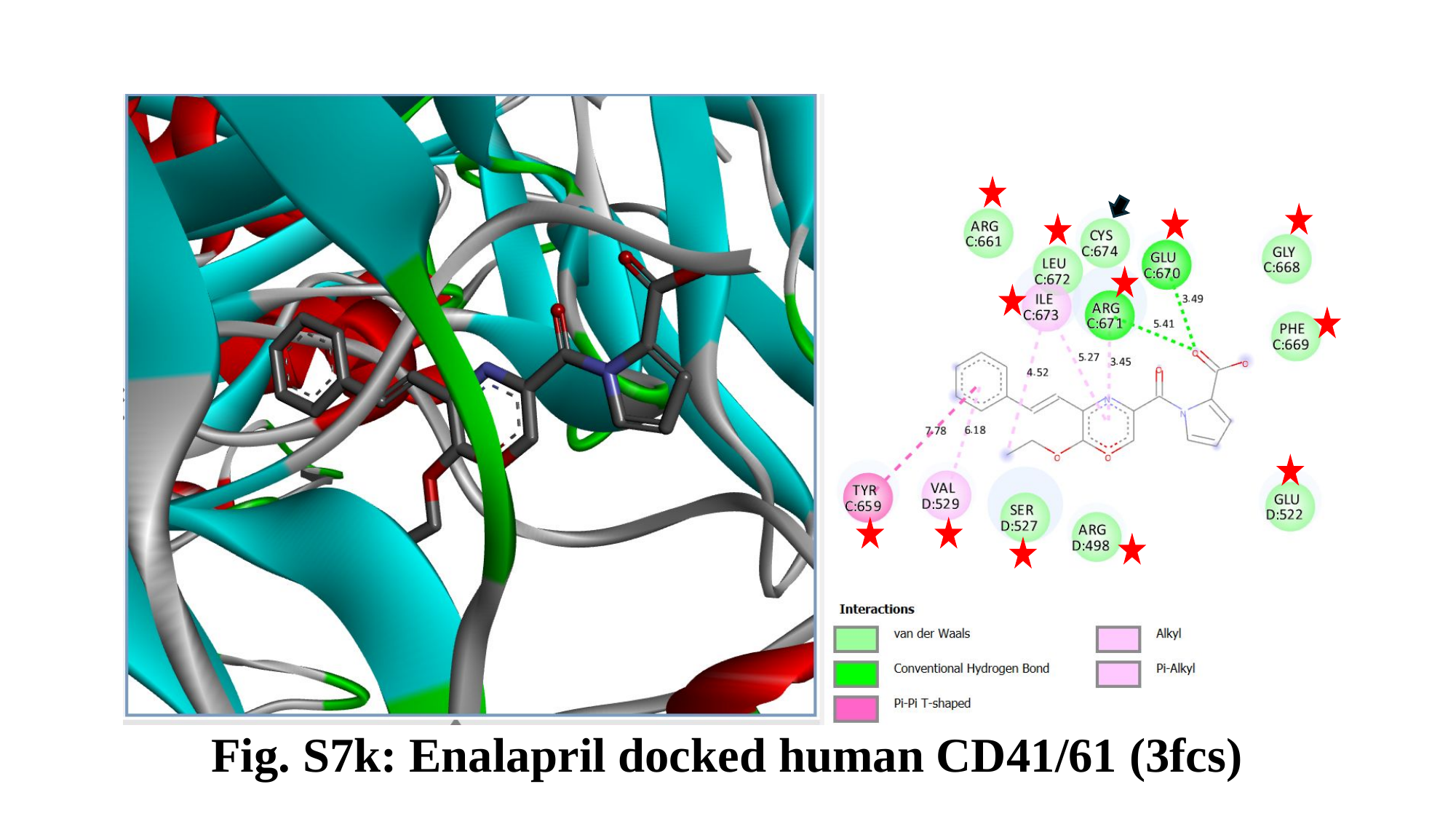

Fig. S7k: Enalapril docked human CD41/61 (3fcs)

## Slide 13
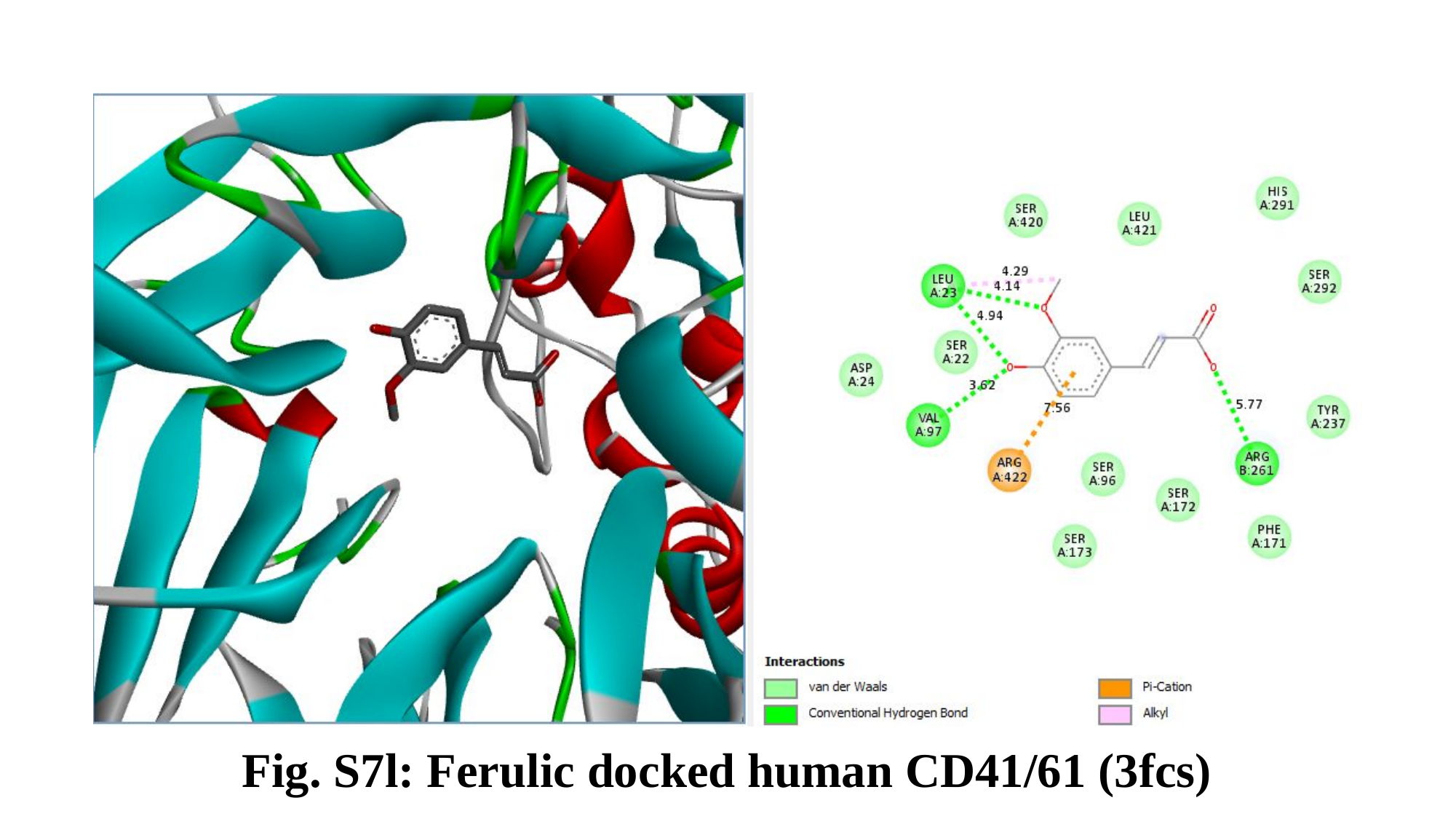

Fig. S7l: Ferulic docked human CD41/61 (3fcs)

## Slide 14
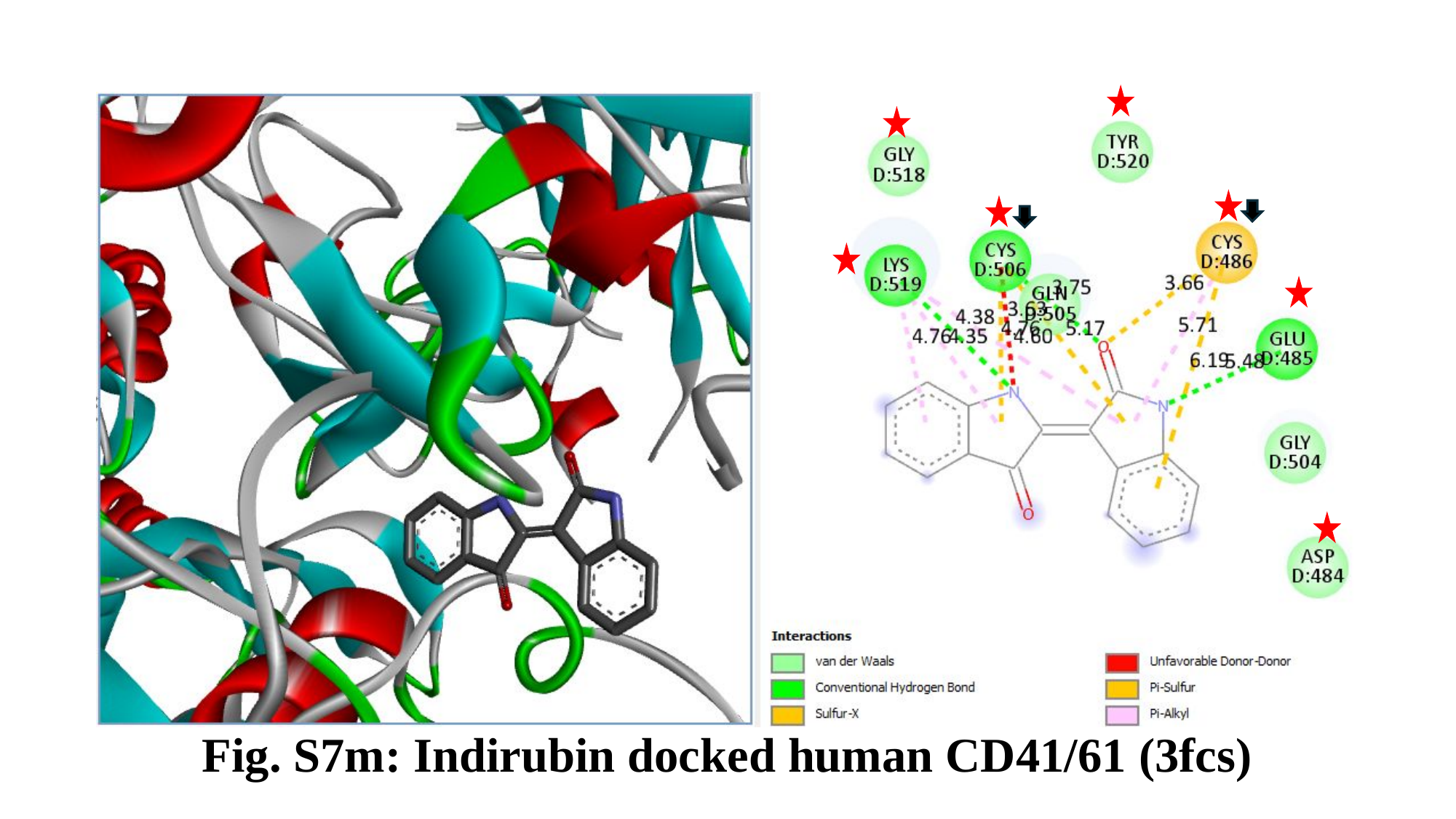

Fig. S7m: Indirubin docked human CD41/61 (3fcs)

## Slide 15
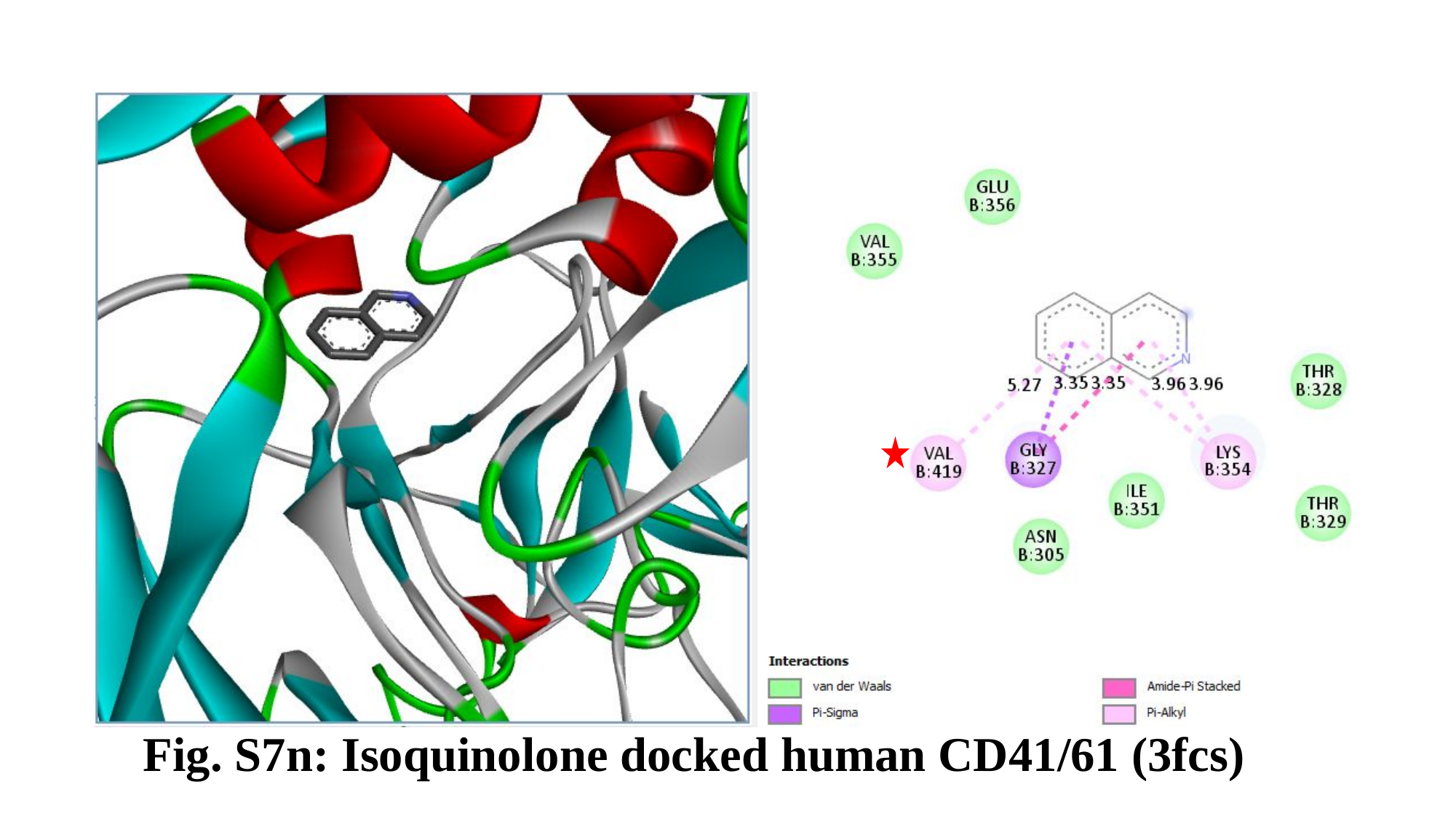

Fig. S7n: Isoquinolone docked human CD41/61 (3fcs)

## Slide 16
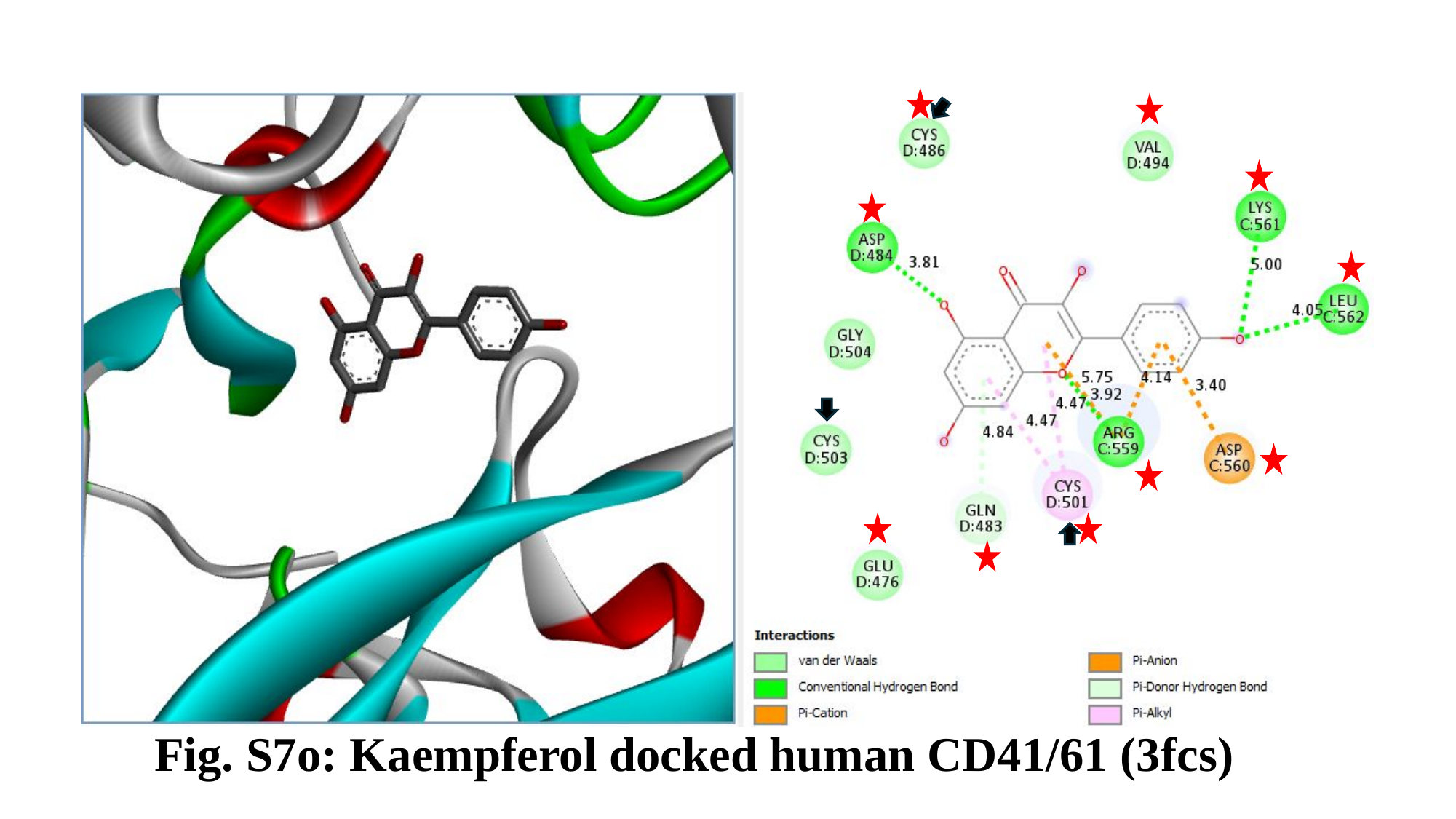

Fig. S7o: Kaempferol docked human CD41/61 (3fcs)

## Slide 17
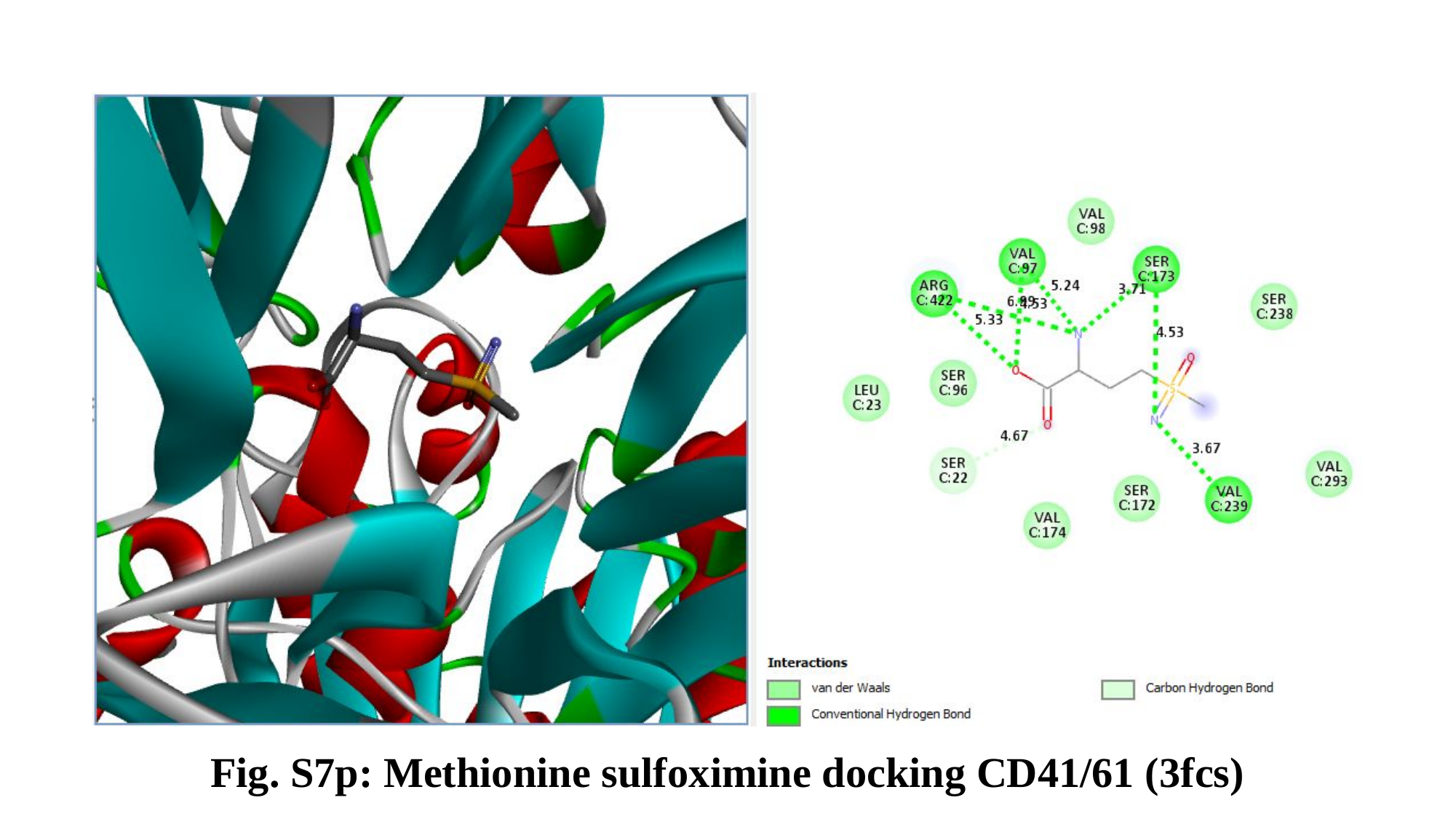

Fig. S7p: Methionine sulfoximine docking CD41/61 (3fcs)

## Slide 18
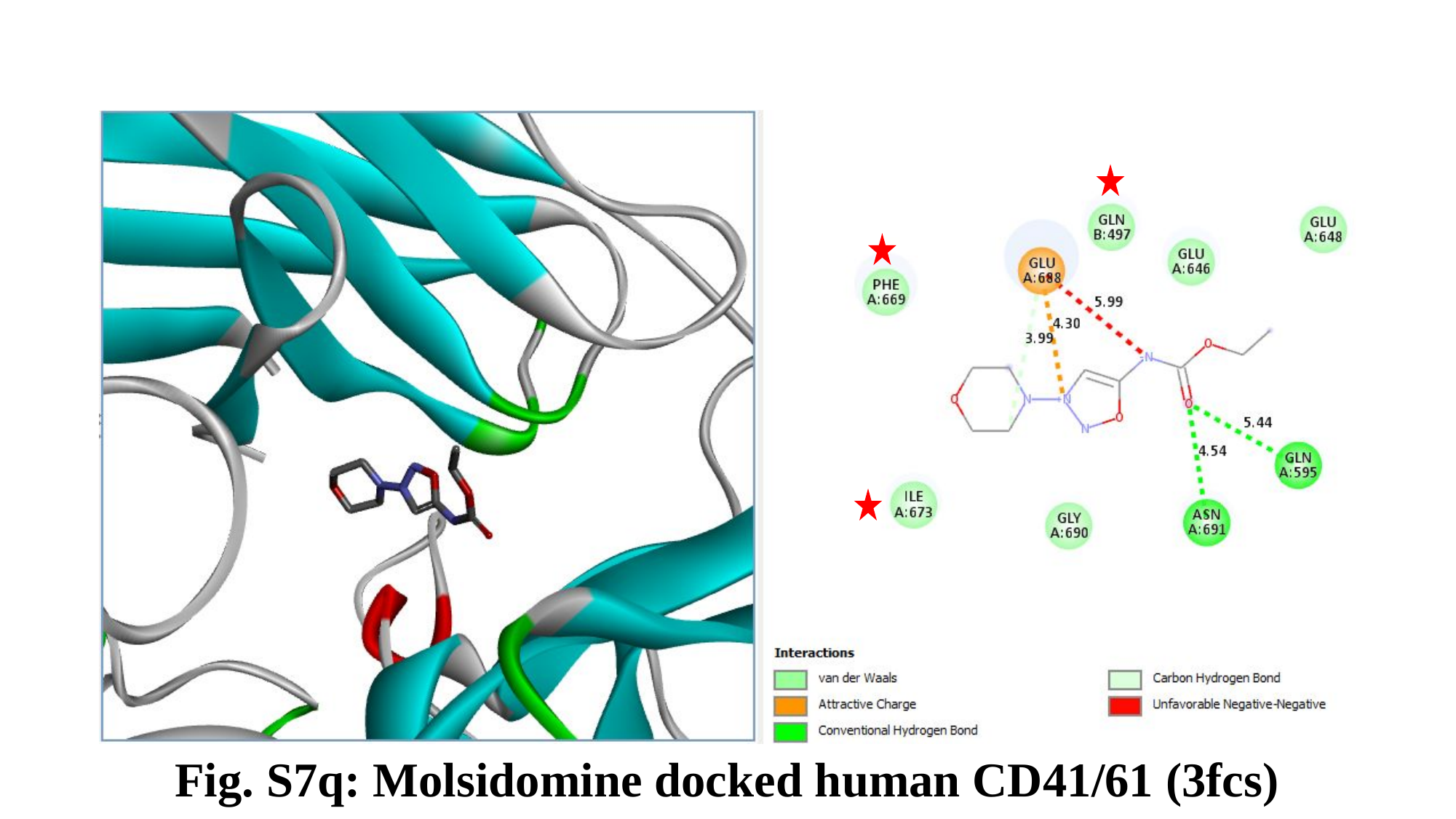

Fig. S7q: Molsidomine docked human CD41/61 (3fcs)

## Slide 19
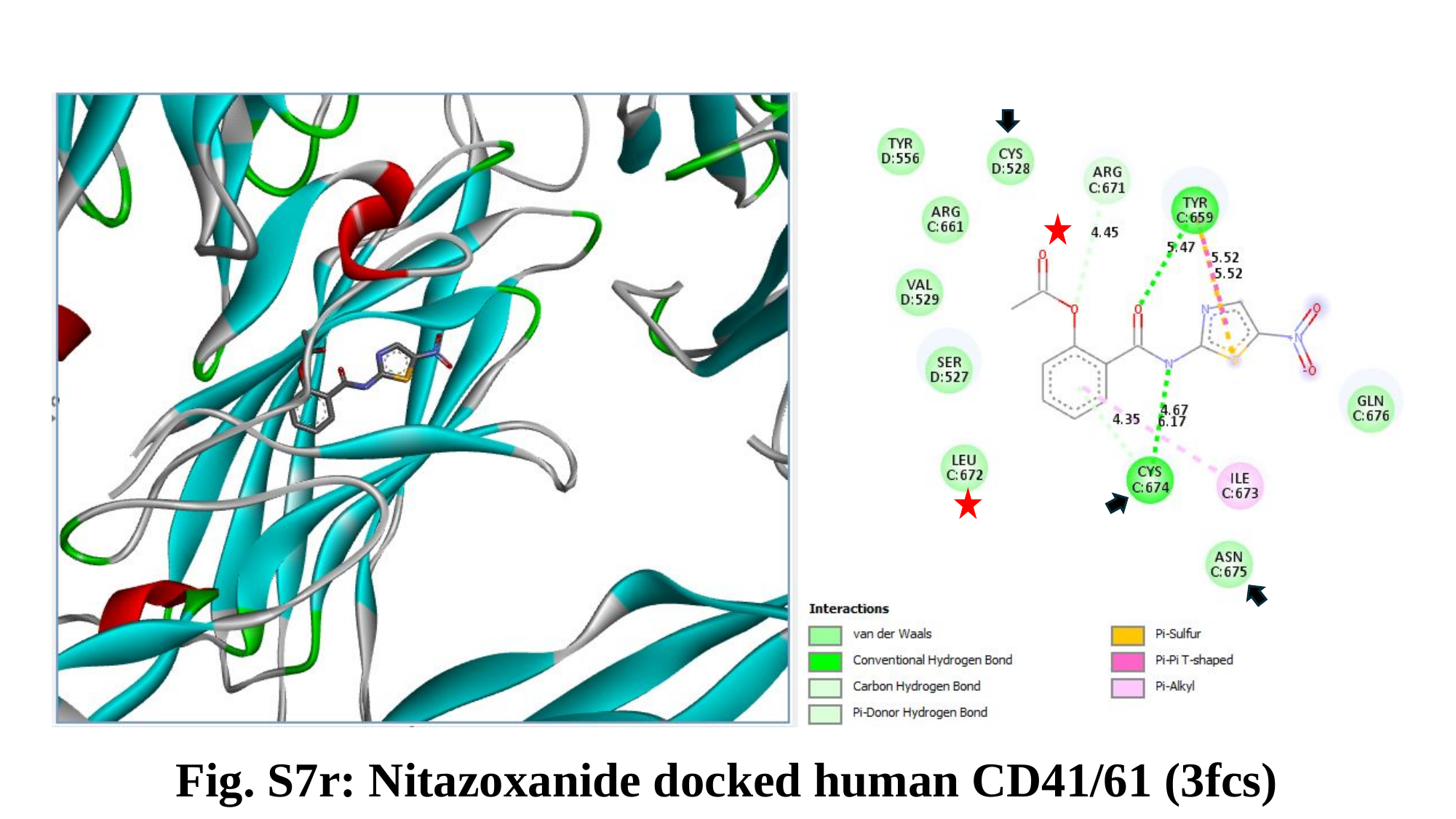

Fig. S7r: Nitazoxanide docked human CD41/61 (3fcs)

## Slide 20
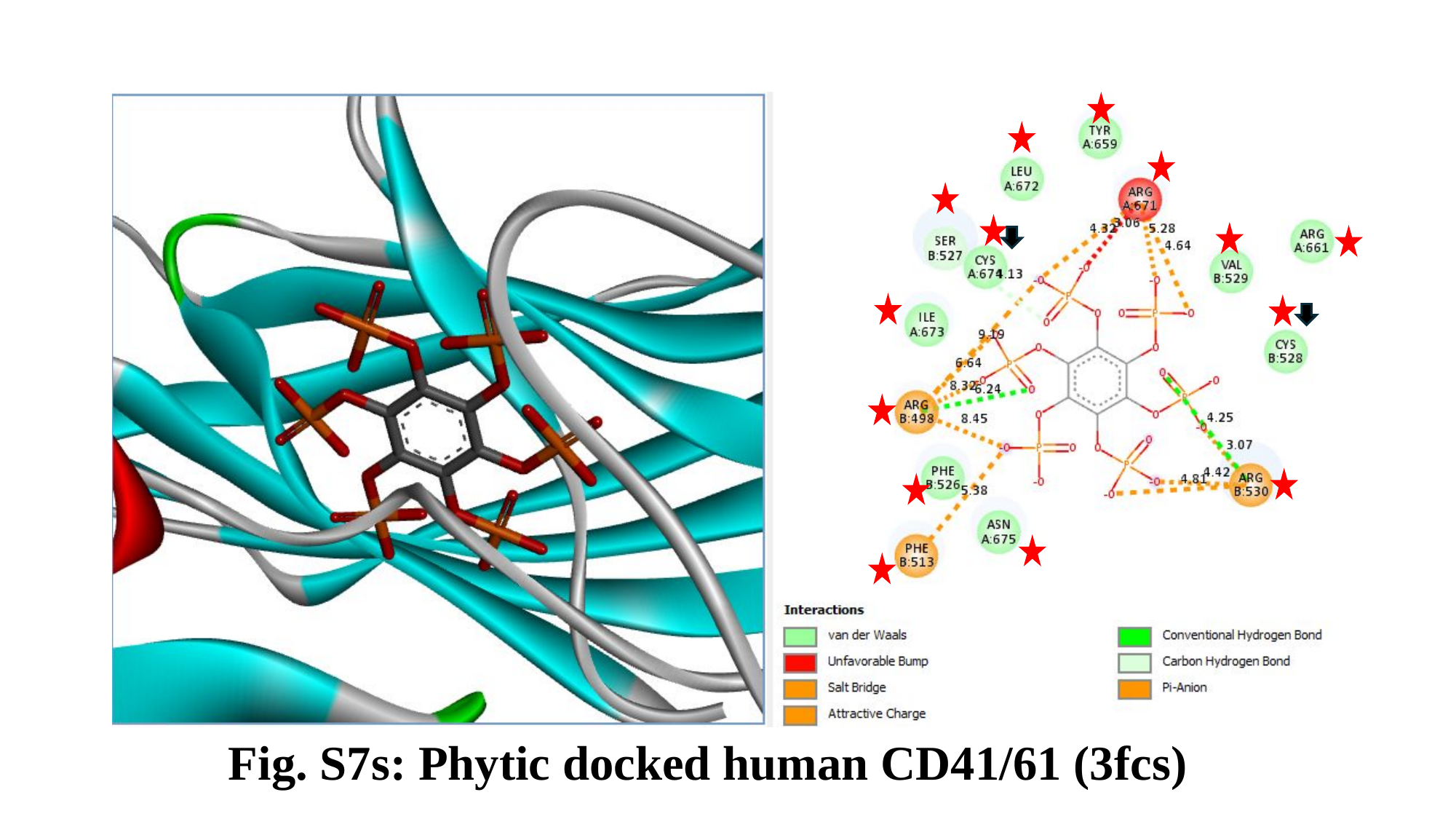

Fig. S7s: Phytic docked human CD41/61 (3fcs)

## Slide 21
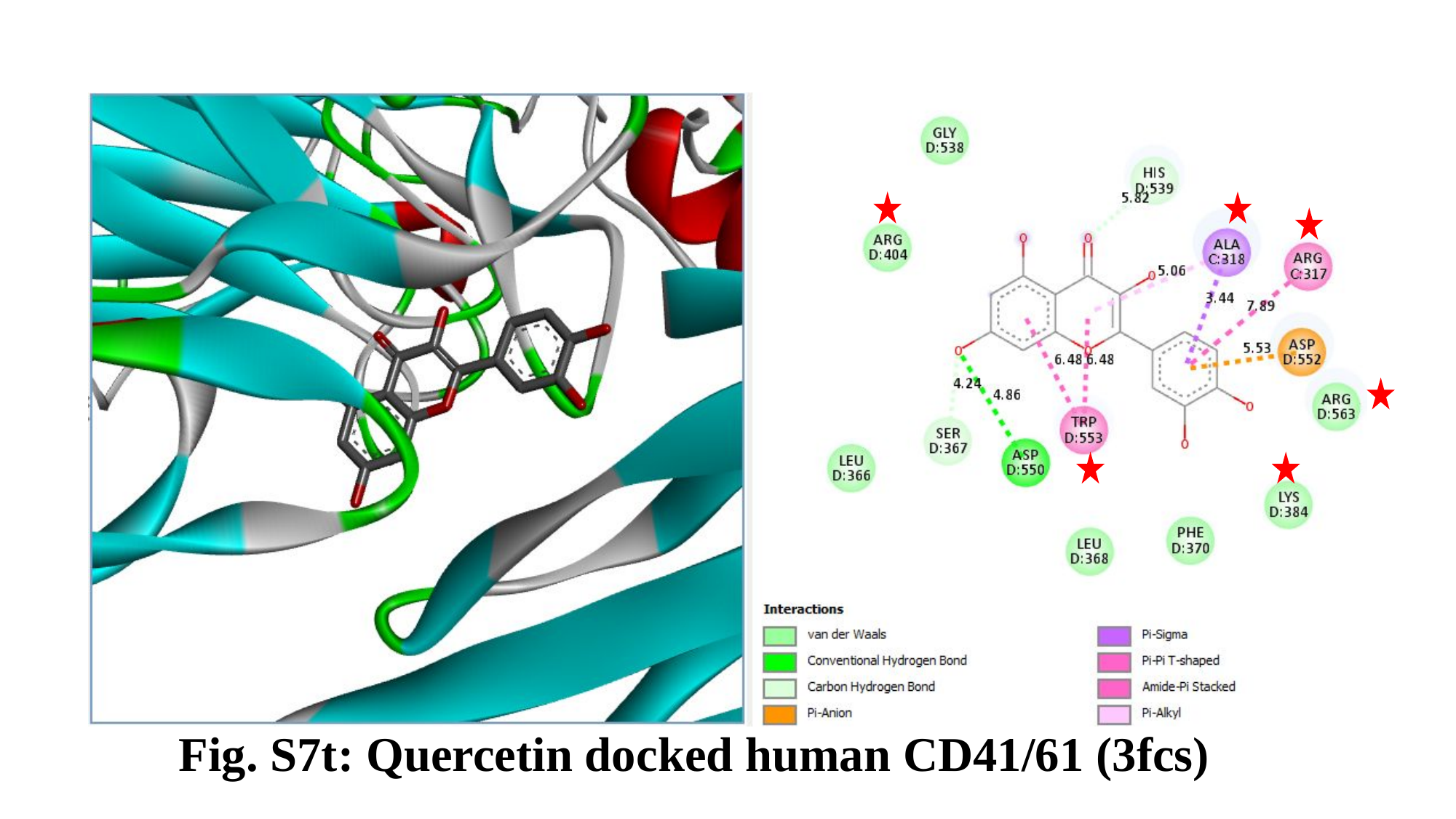

Fig. S7t: Quercetin docked human CD41/61 (3fcs)

## Slide 22
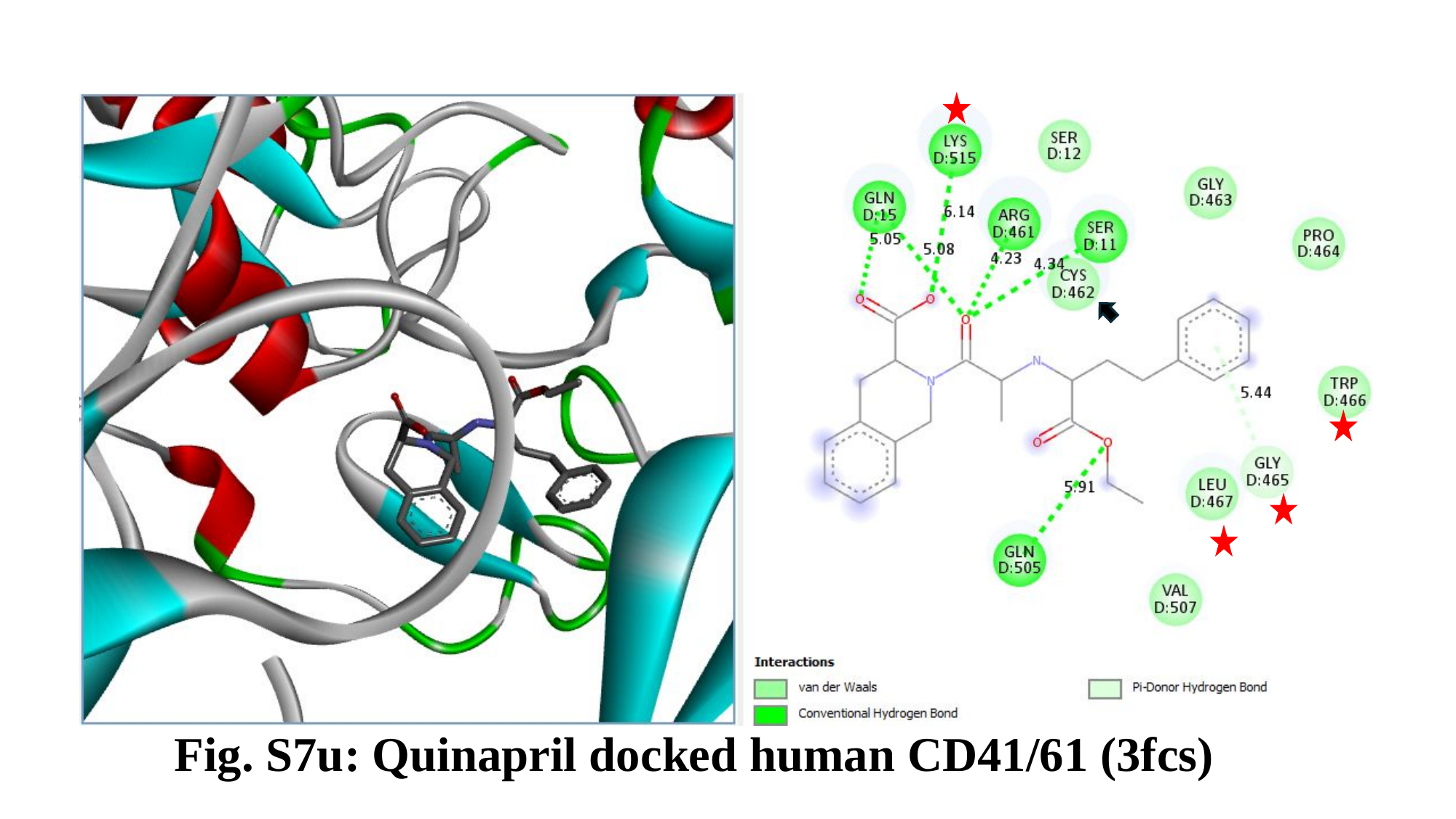

Fig. S7u: Quinapril docked human CD41/61 (3fcs)

## Slide 23
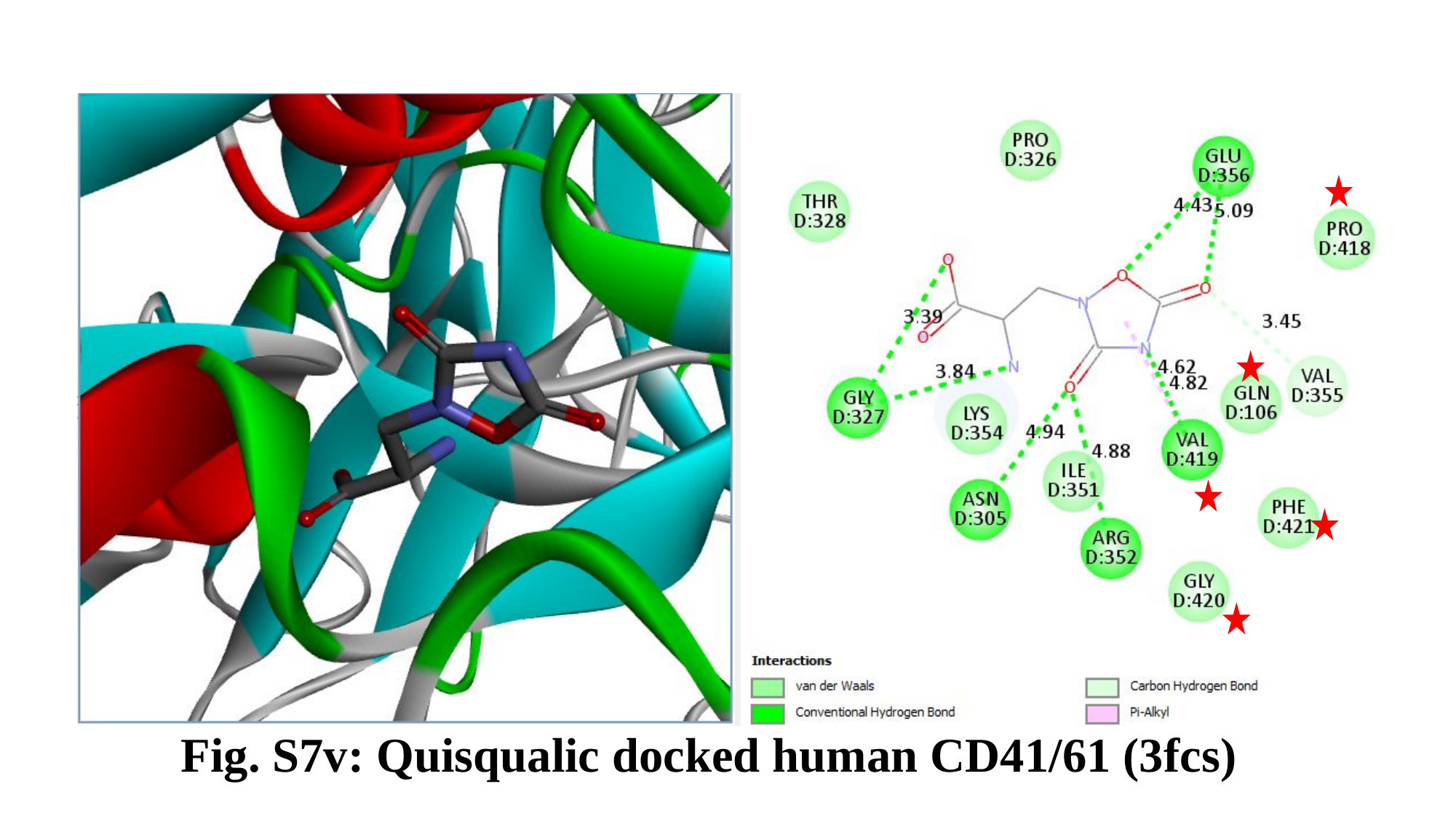

Fig. S7v: Quisqualic docked human CD41/61 (3fcs)

## Slide 24
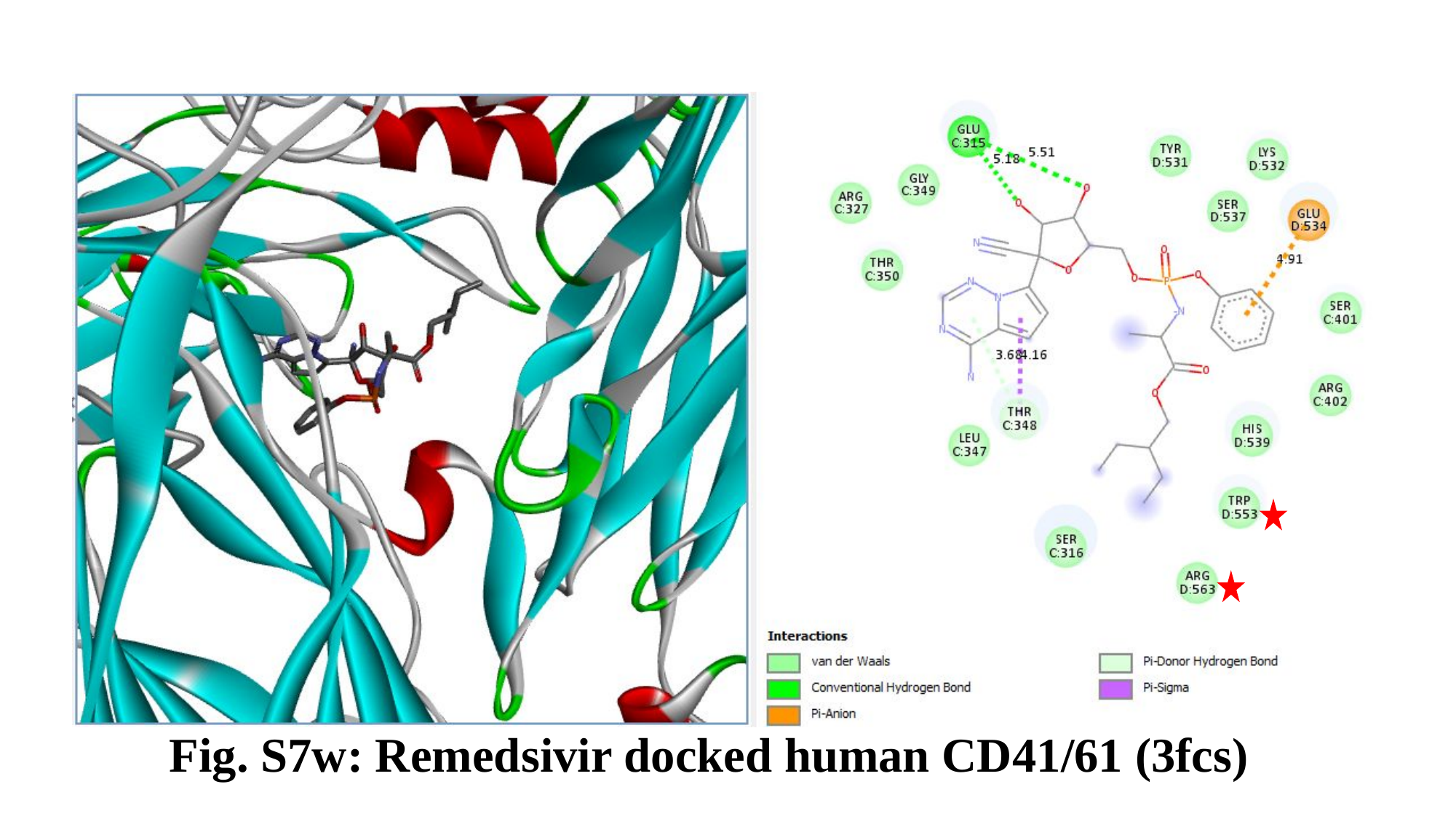

Fig. S7w: Remedsivir docked human CD41/61 (3fcs)

## Slide 25
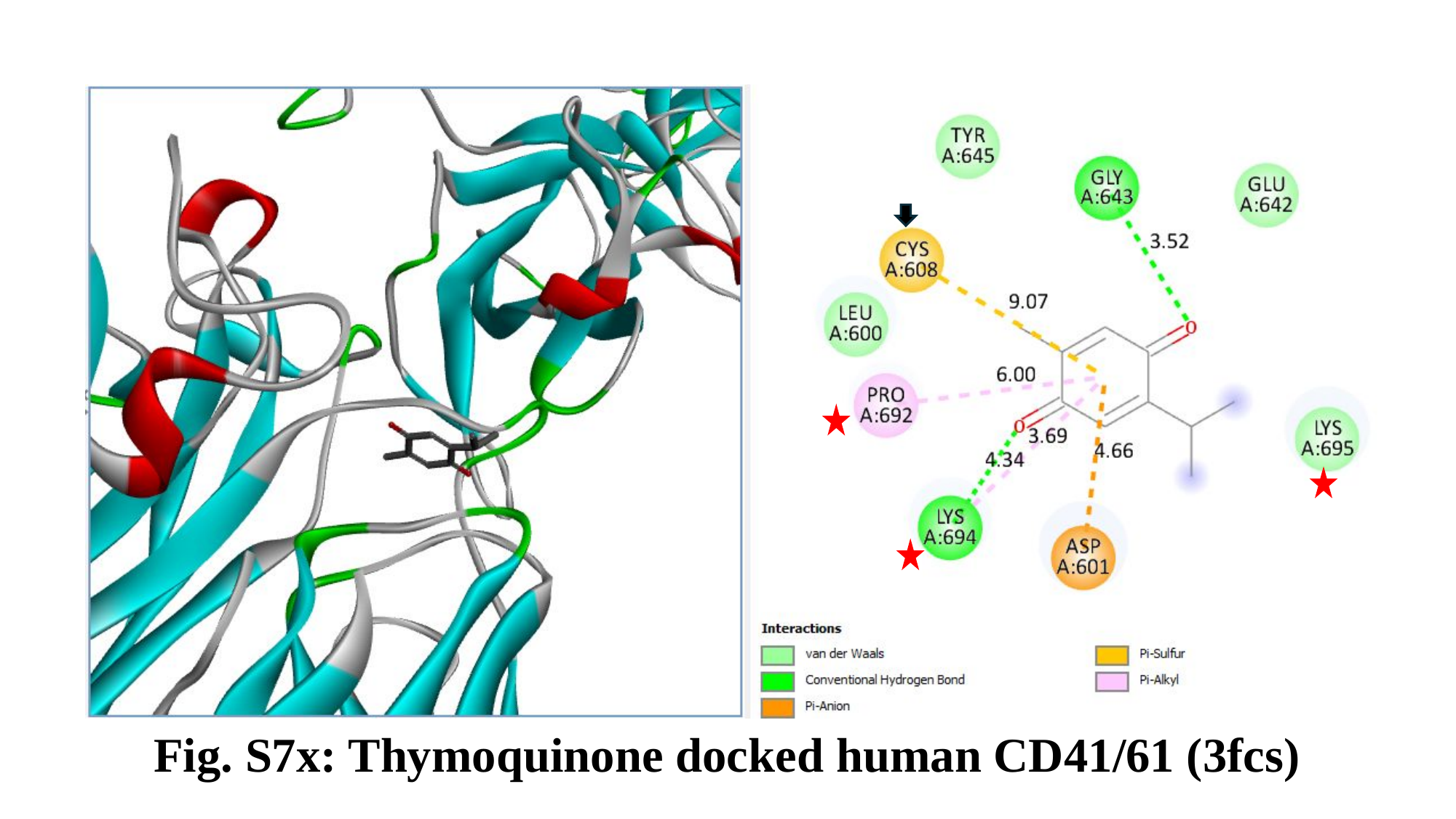

Fig. S7x: Thymoquinone docked human CD41/61 (3fcs)

## Slide 26
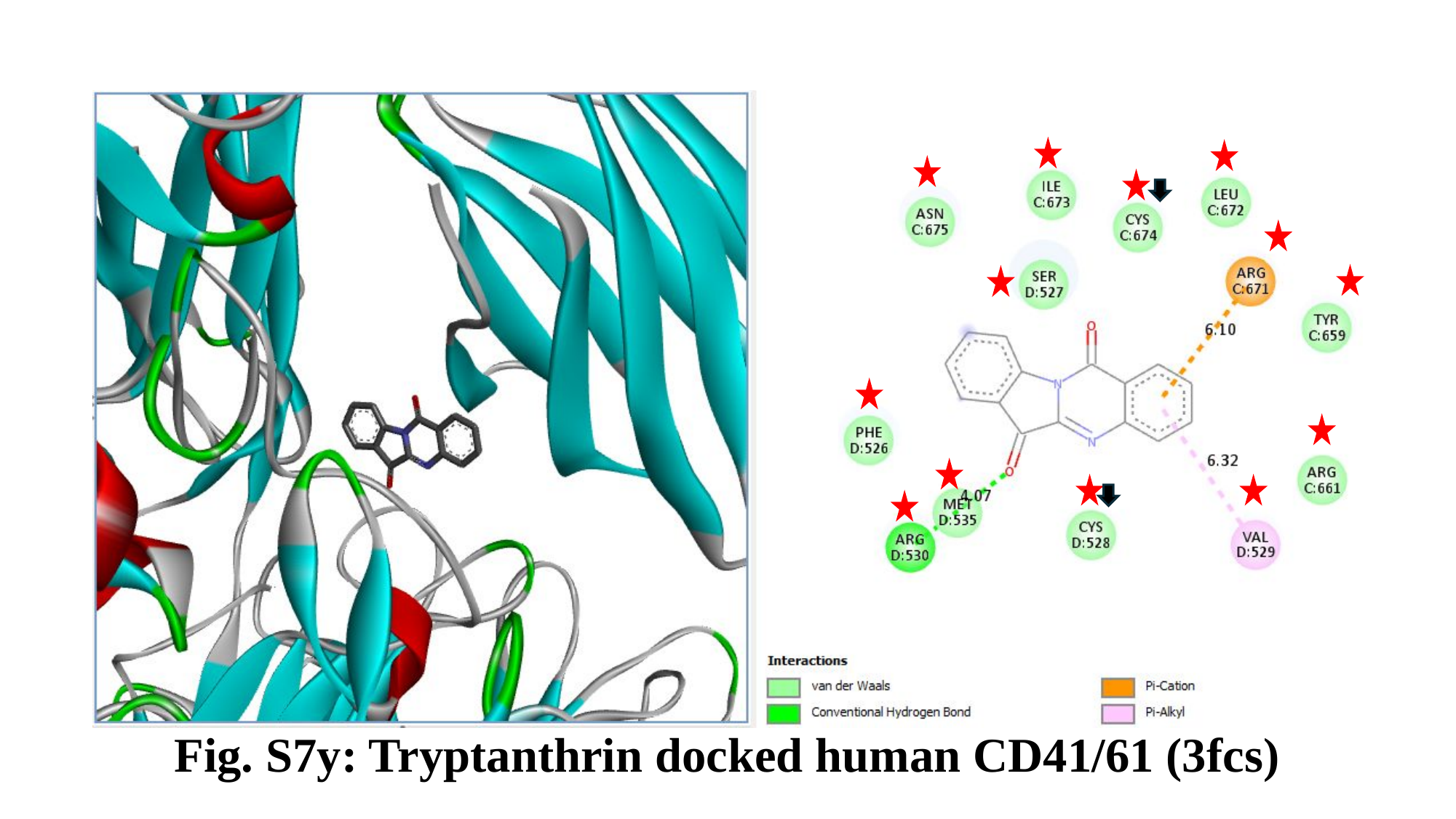

Fig. S7y: Tryptanthrin docked human CD41/61 (3fcs)
