# Supplementary material for: Targeting asparagine and cysteine in SARS-CoV-2 variants and human pro-inflammatory mediators to alleviate COVID-19 severity; a cross-section and in-silico study
Source: Sci Rep. 2025 Nov 3;15:38445. doi: 10.1038/s41598-025-19359-y (PMC12583749; doi:10.1038/s41598-025-19359-y)
Supplement: Supplementary file 15 — Supplementary Material 15 [file 41598_2025_19359_MOESM15_ESM.pptx]

## Slide 1
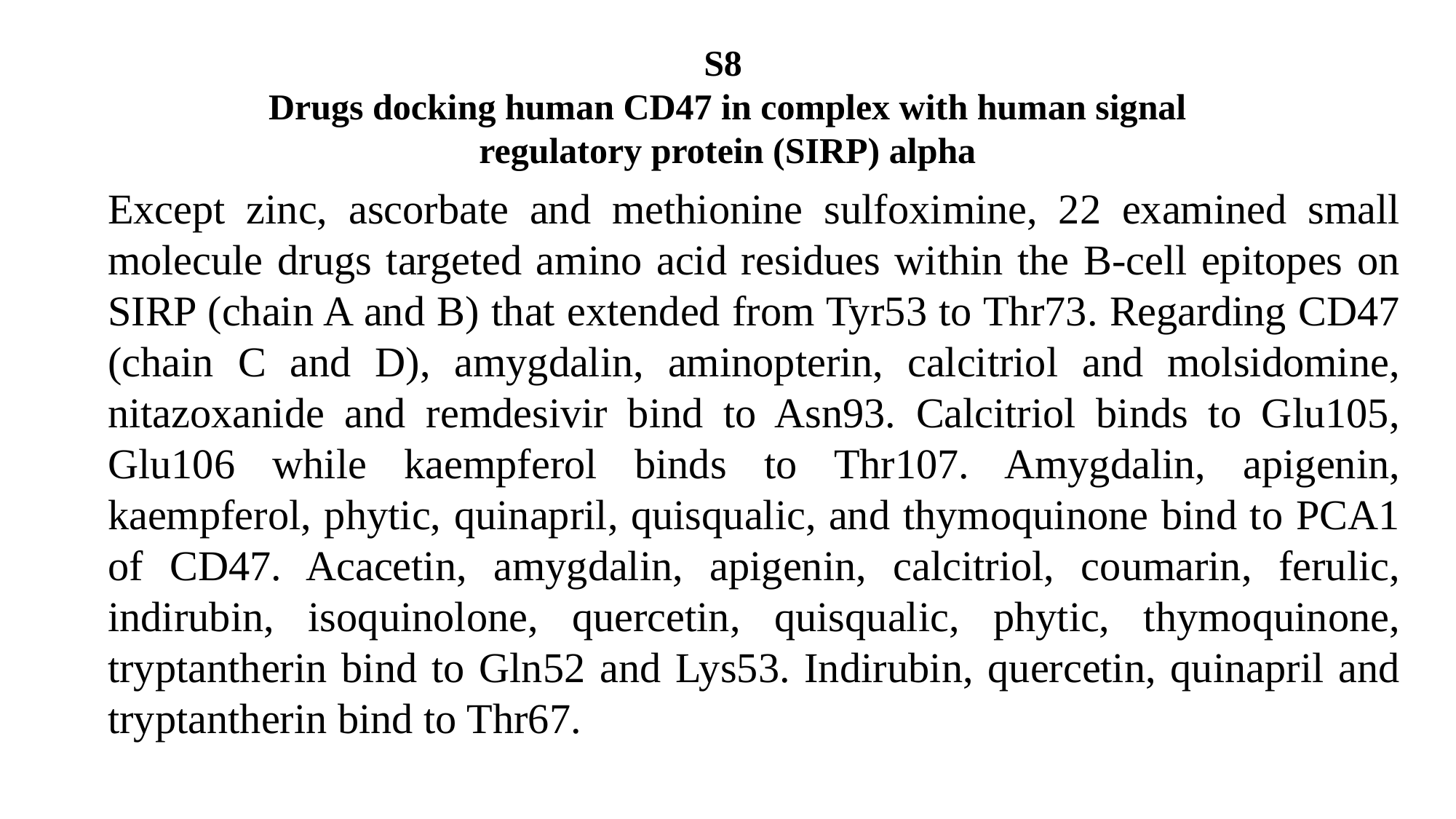

# S8 Drugs docking human CD47 in complex with human signal regulatory protein (SIRP) alpha
Except zinc, ascorbate and methionine sulfoximine, 22 examined small molecule drugs targeted amino acid residues within the B-cell epitopes on SIRP (chain A and B) that extended from Tyr53 to Thr73. Regarding CD47 (chain C and D), amygdalin, aminopterin, calcitriol and molsidomine, nitazoxanide and remdesivir bind to Asn93. Calcitriol binds to Glu105, Glu106 while kaempferol binds to Thr107. Amygdalin, apigenin, kaempferol, phytic, quinapril, quisqualic, and thymoquinone bind to PCA1 of CD47. Acacetin, amygdalin, apigenin, calcitriol, coumarin, ferulic, indirubin, isoquinolone, quercetin, quisqualic, phytic, thymoquinone, tryptantherin bind to Gln52 and Lys53. Indirubin, quercetin, quinapril and tryptantherin bind to Thr67.

## Slide 2
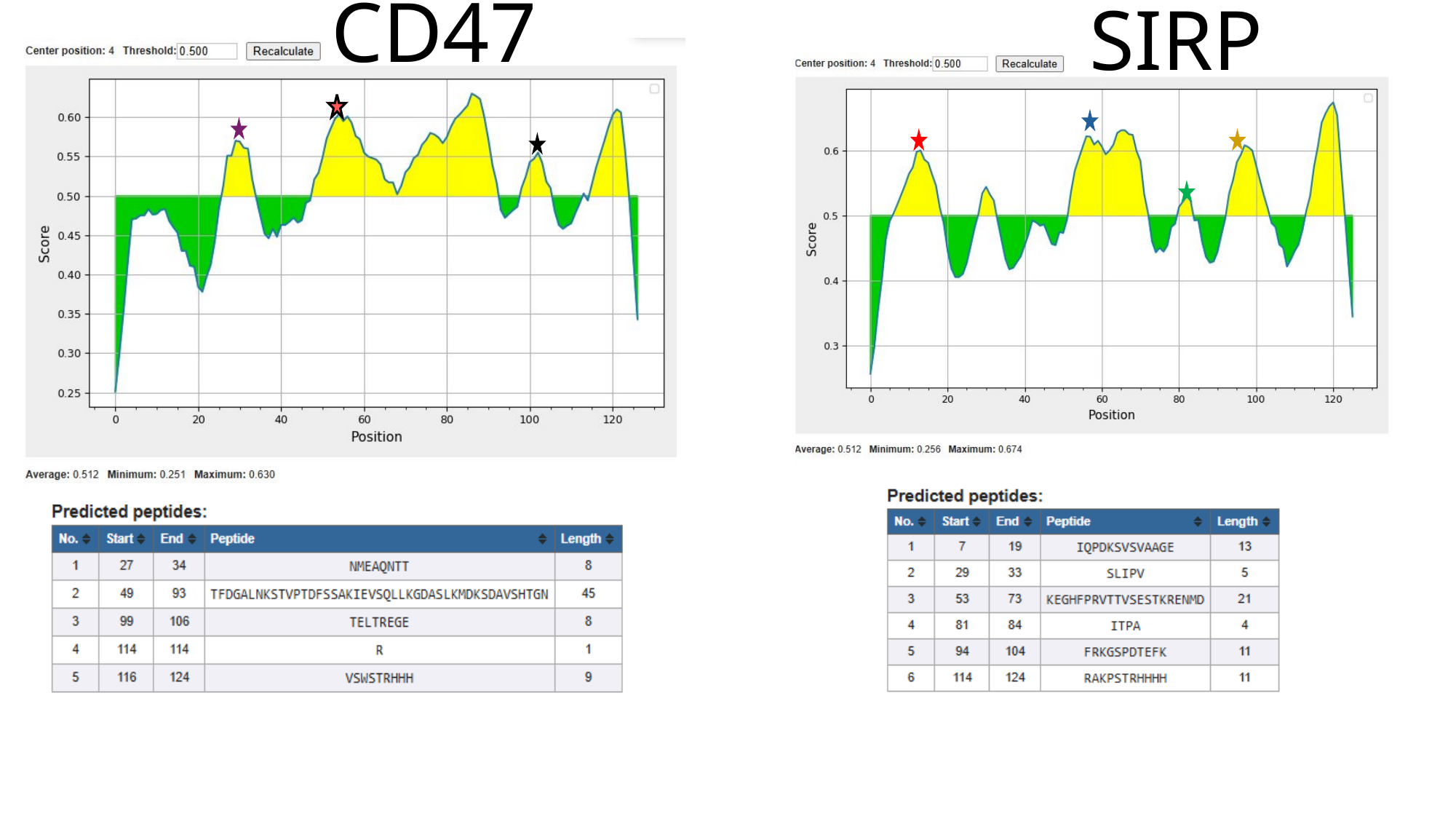

CD47
SIRP

## Slide 3
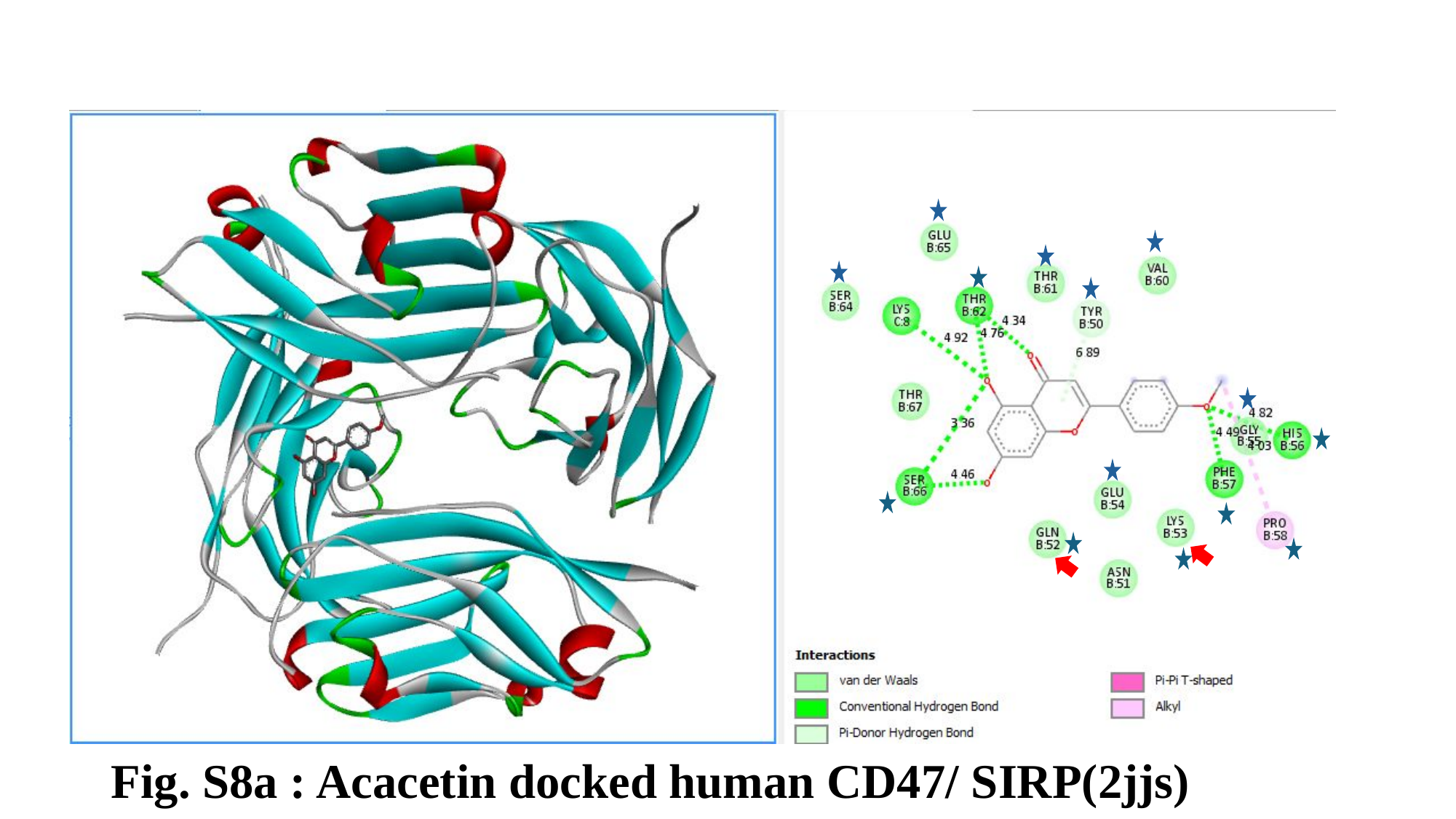

Fig. S8a : Acacetin docked human CD47/ SIRP(2jjs)

## Slide 4
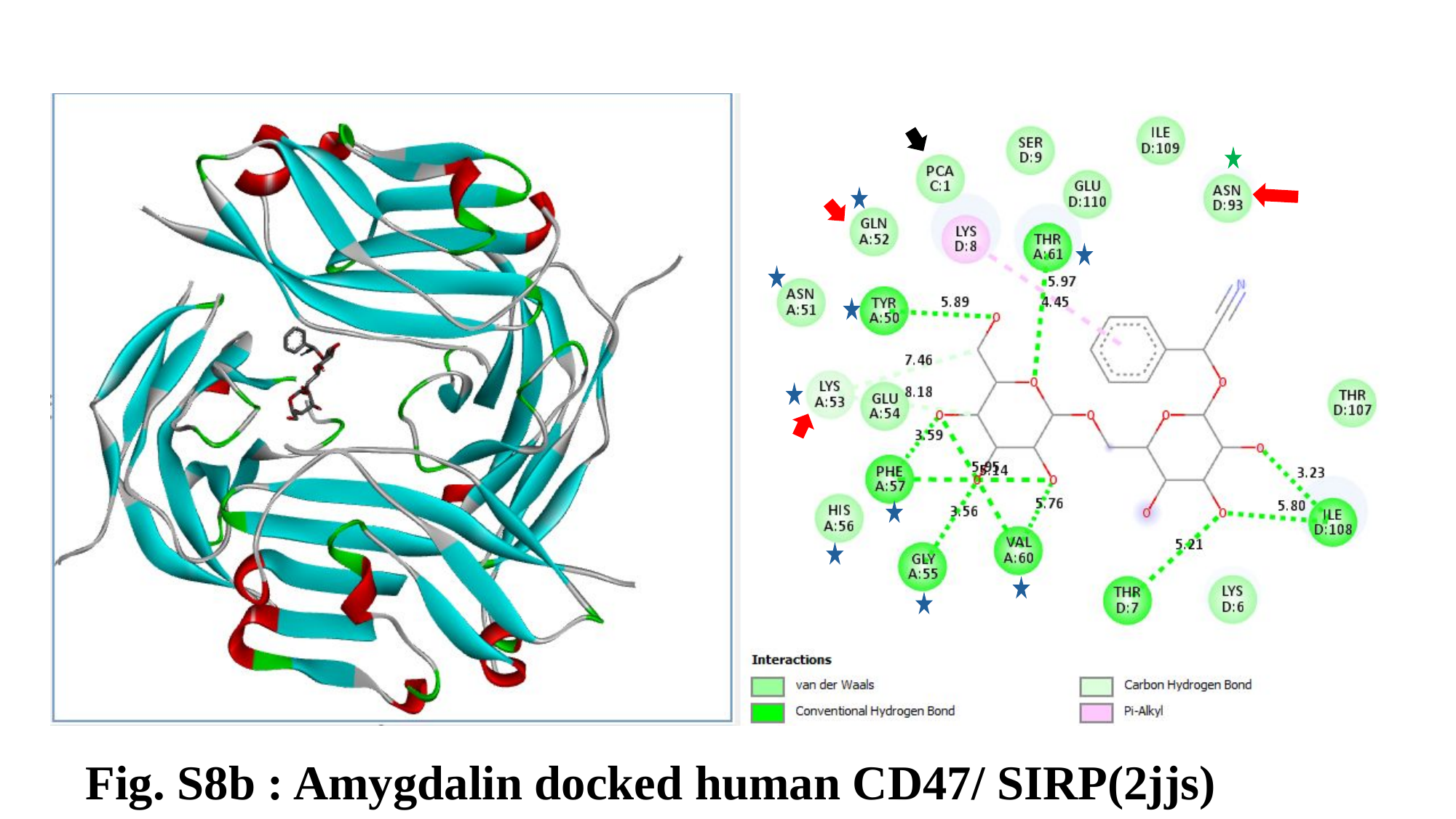

Fig. S8b : Amygdalin docked human CD47/ SIRP(2jjs)

## Slide 5
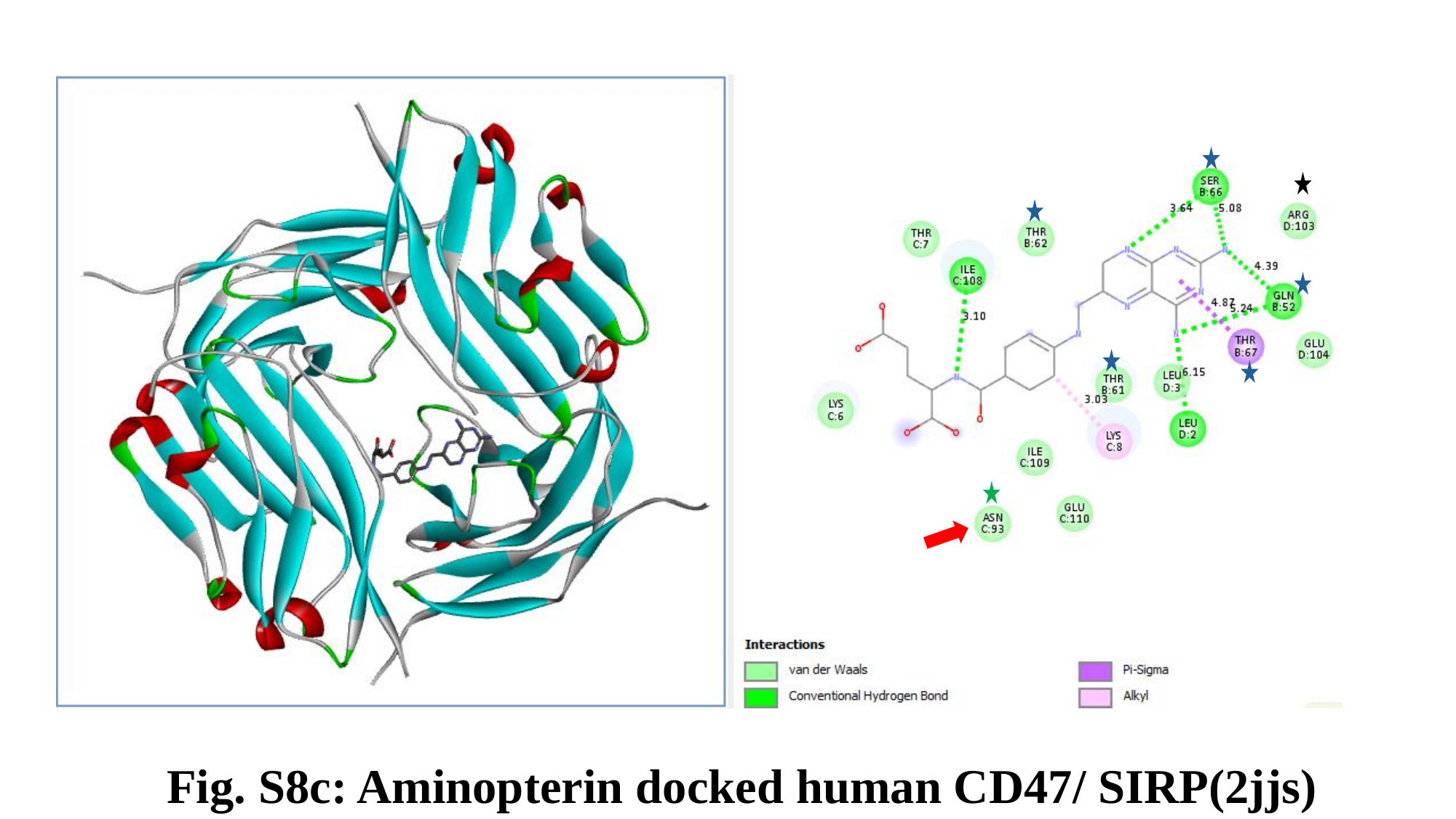

Fig. S8c: Aminopterin docked human CD47/ SIRP(2jjs)

## Slide 6
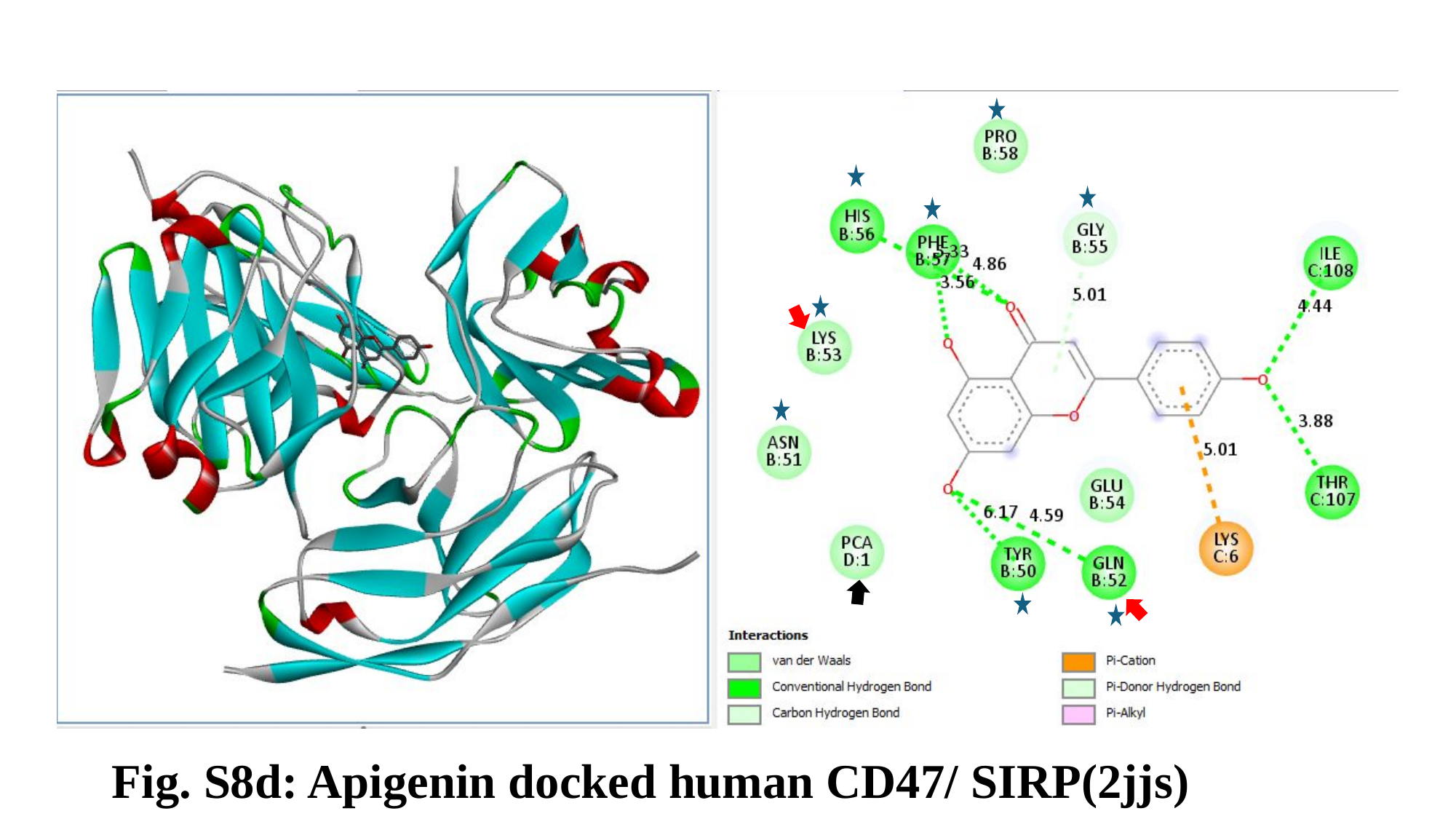

Fig. S8d: Apigenin docked human CD47/ SIRP(2jjs)

## Slide 7
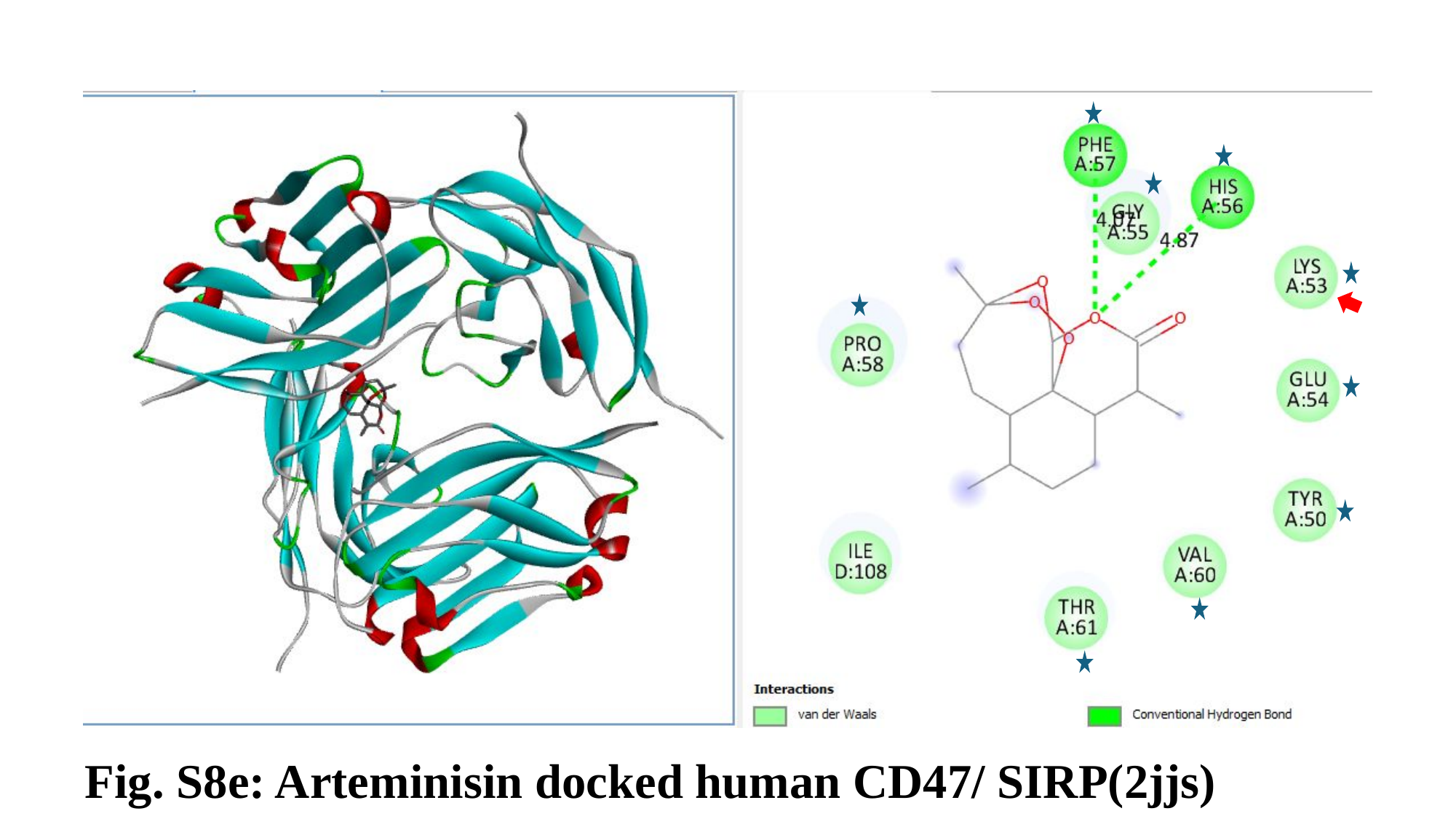

Fig. S8e: Arteminisin docked human CD47/ SIRP(2jjs)

## Slide 8
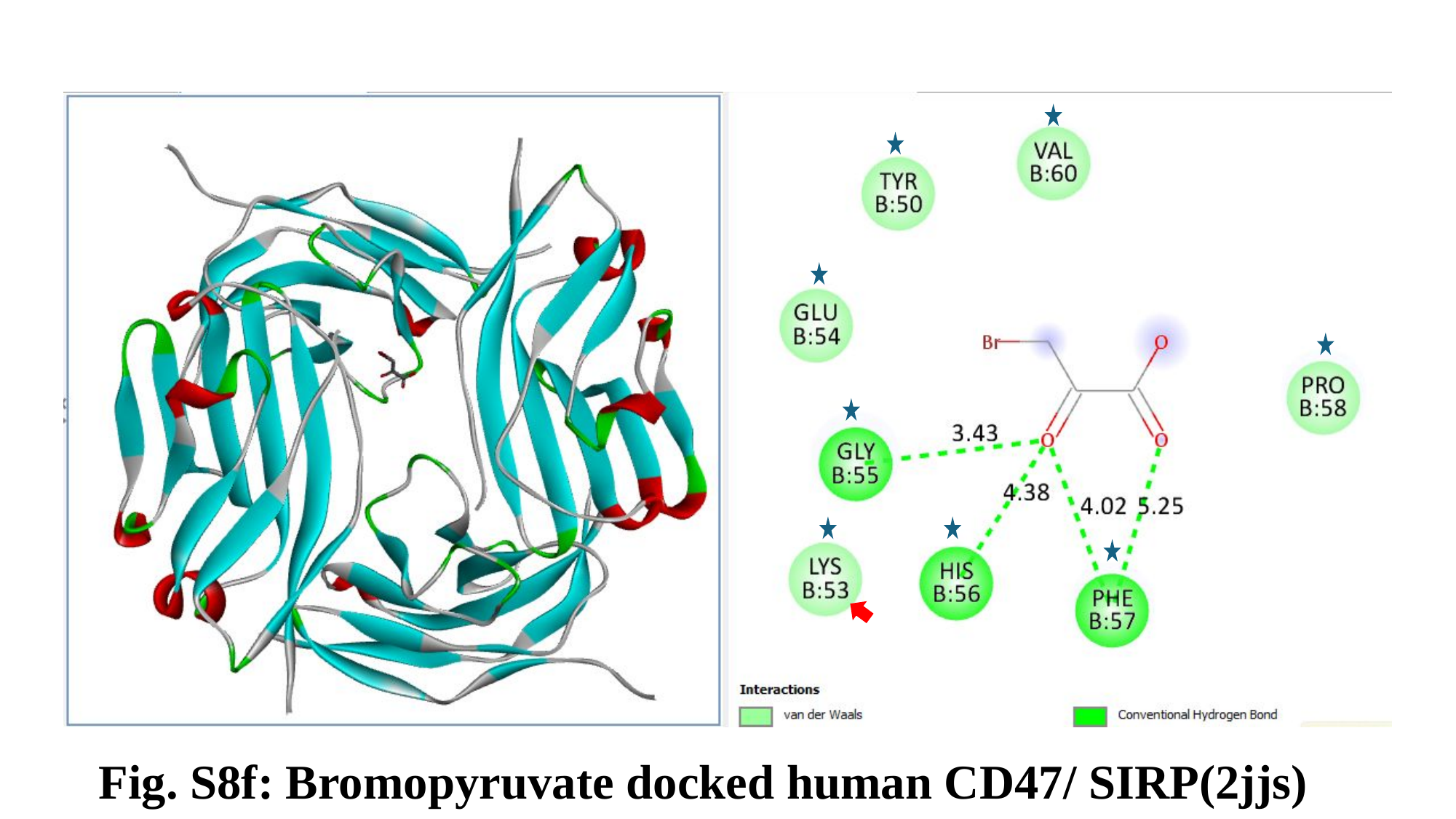

Fig. S8f: Bromopyruvate docked human CD47/ SIRP(2jjs)

## Slide 9
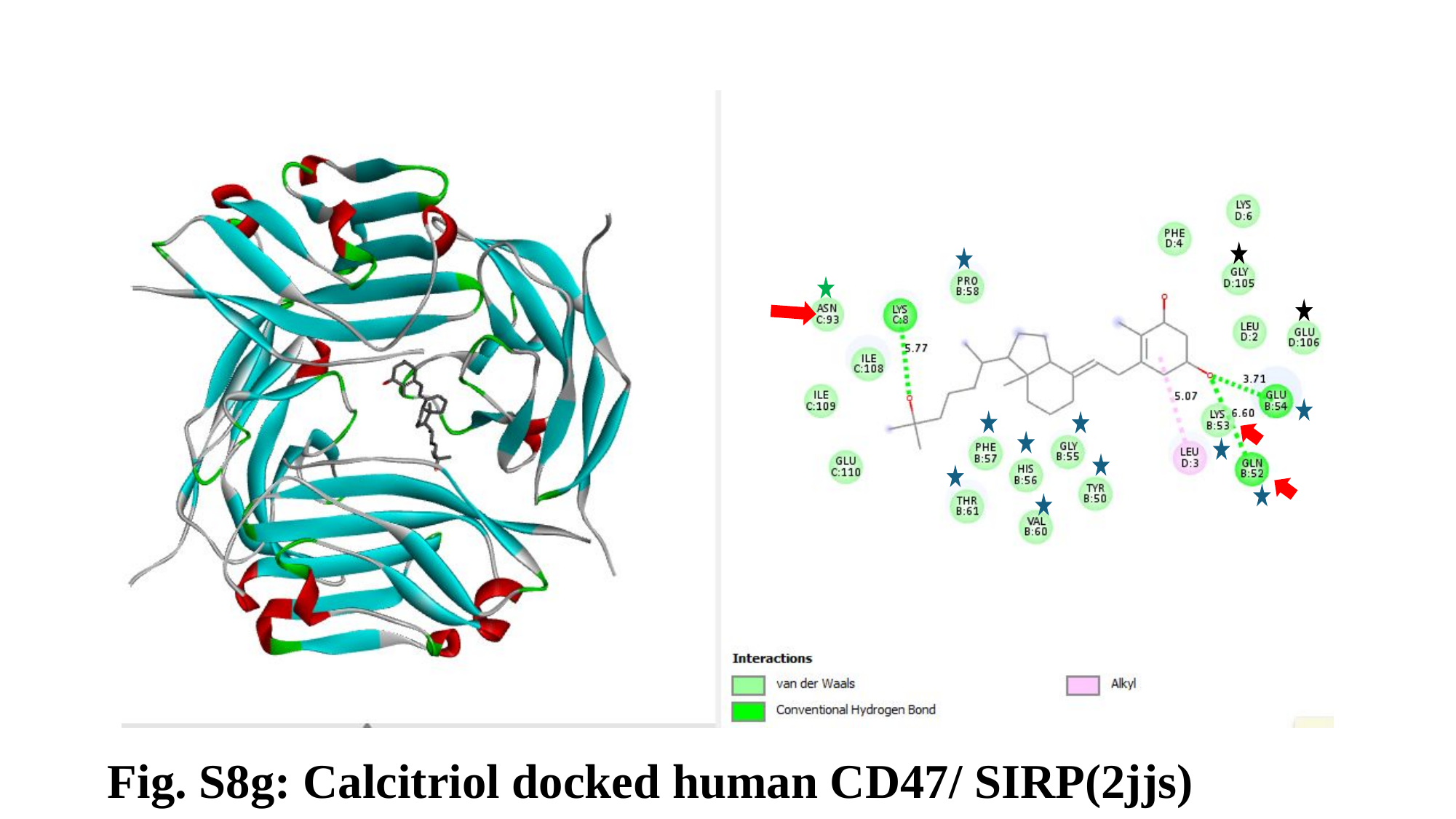

Fig. S8g: Calcitriol docked human CD47/ SIRP(2jjs)

## Slide 10
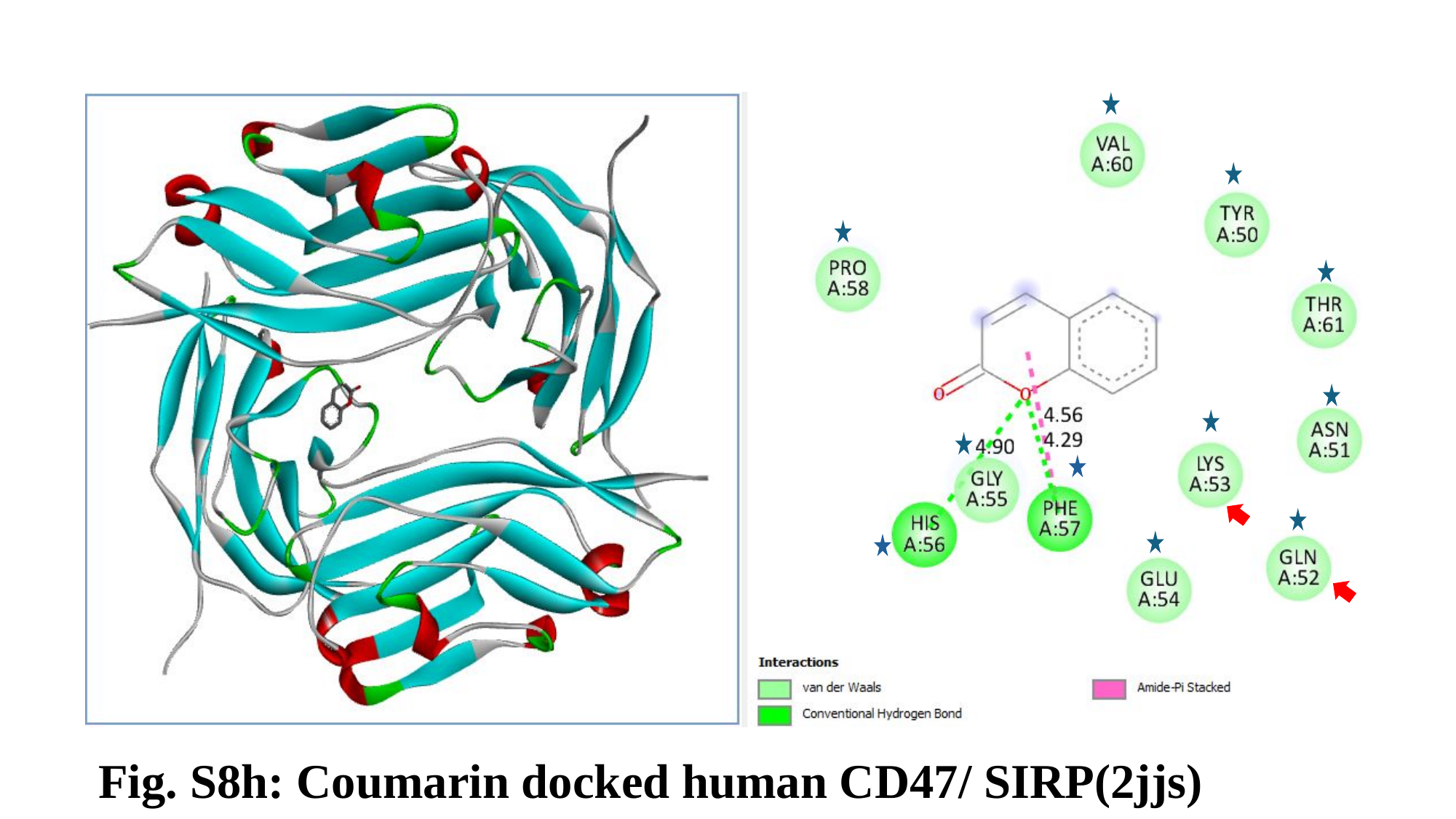

Fig. S8h: Coumarin docked human CD47/ SIRP(2jjs)

## Slide 11
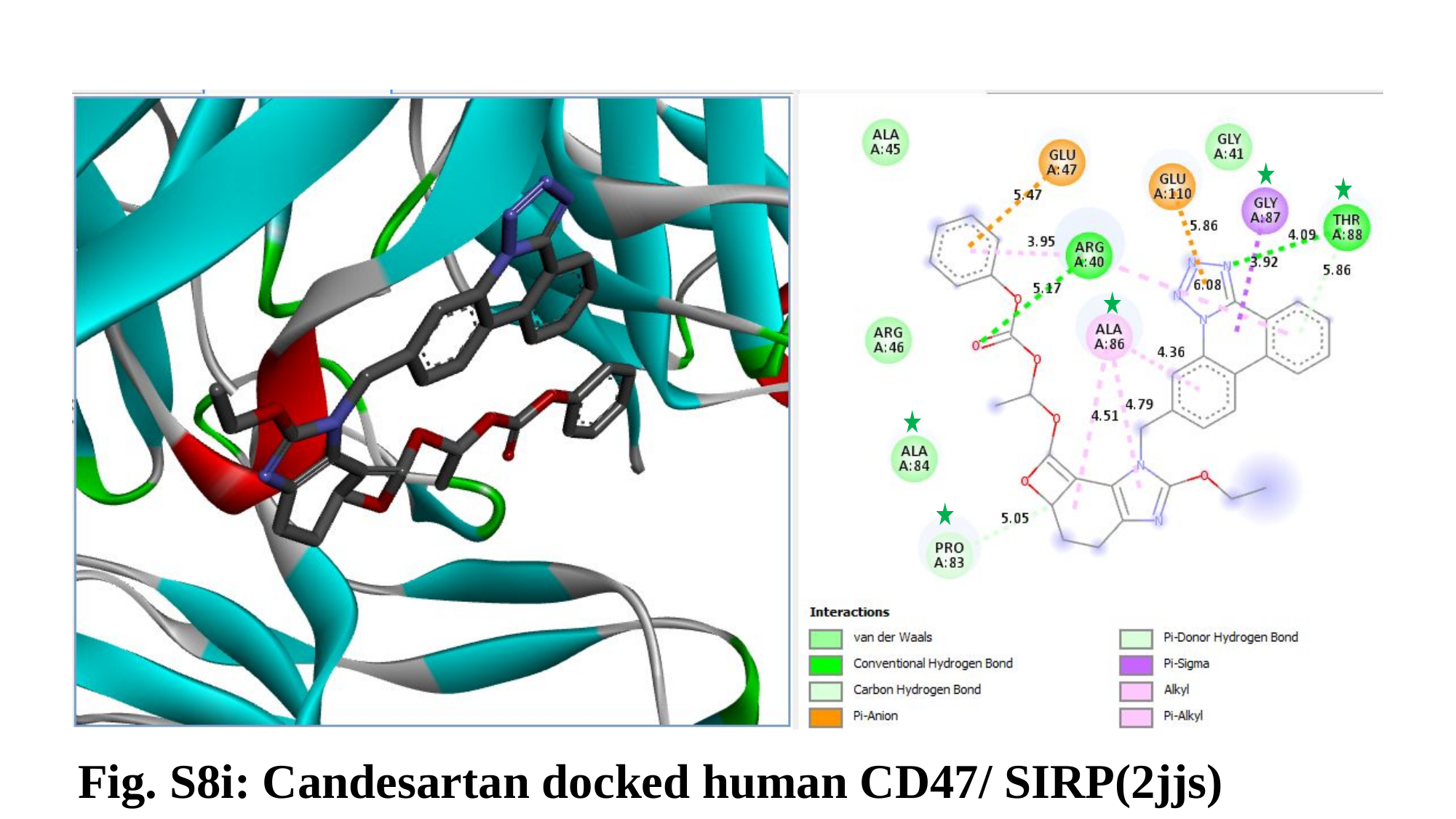

Fig. S8i: Candesartan docked human CD47/ SIRP(2jjs)

## Slide 12
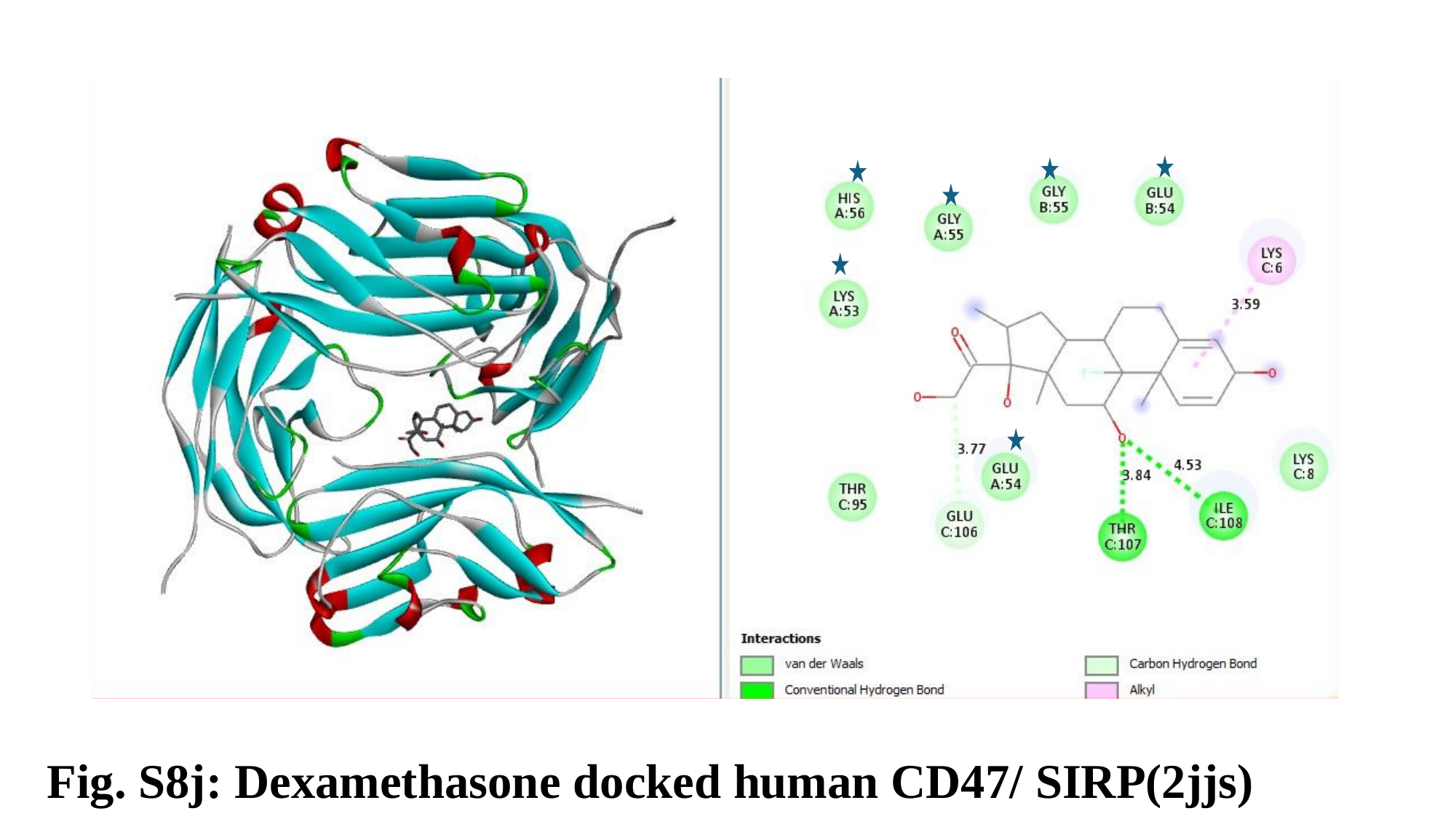

Fig. S8j: Dexamethasone docked human CD47/ SIRP(2jjs)

## Slide 13
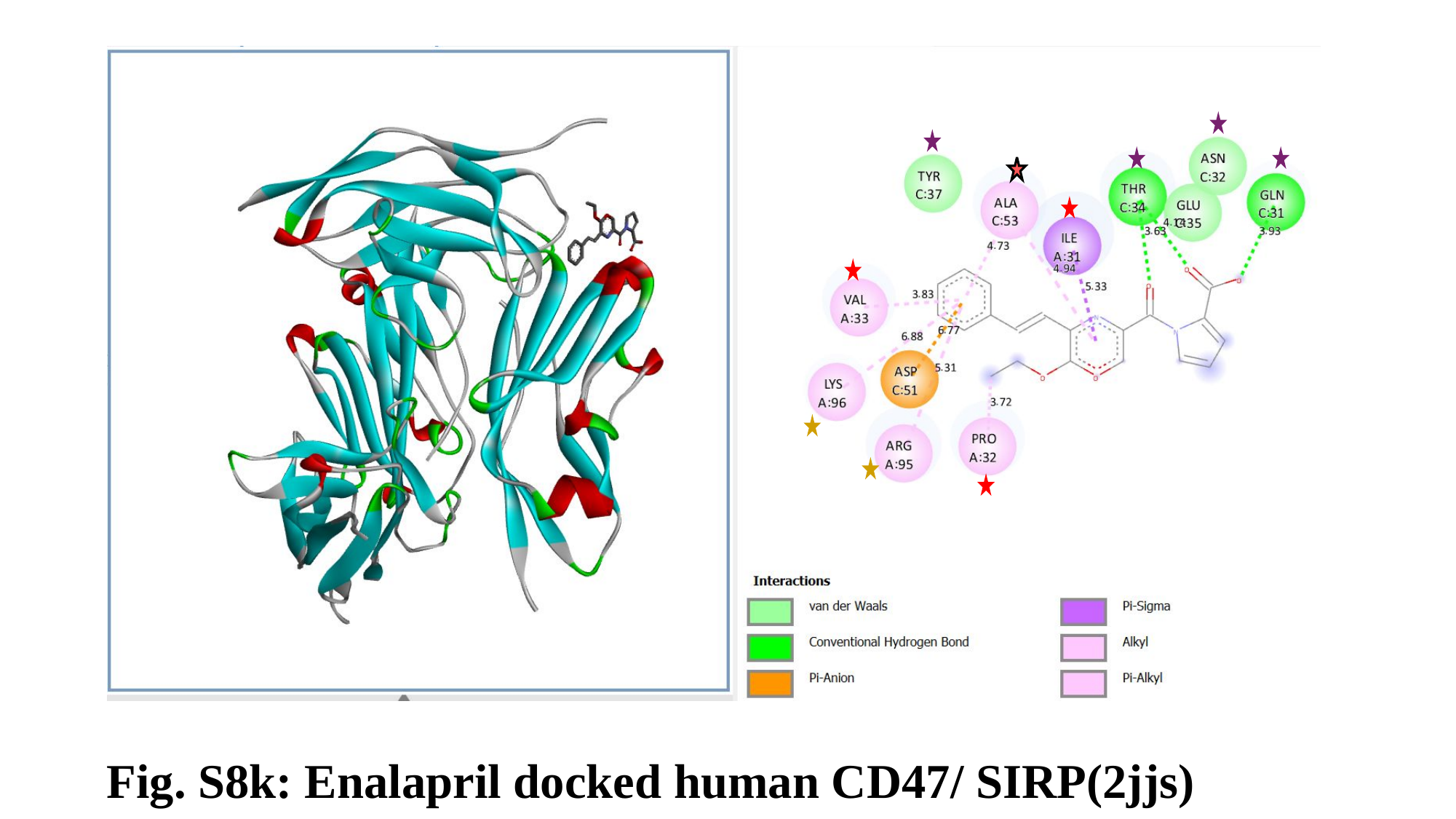

Fig. S8k: Enalapril docked human CD47/ SIRP(2jjs)

## Slide 14
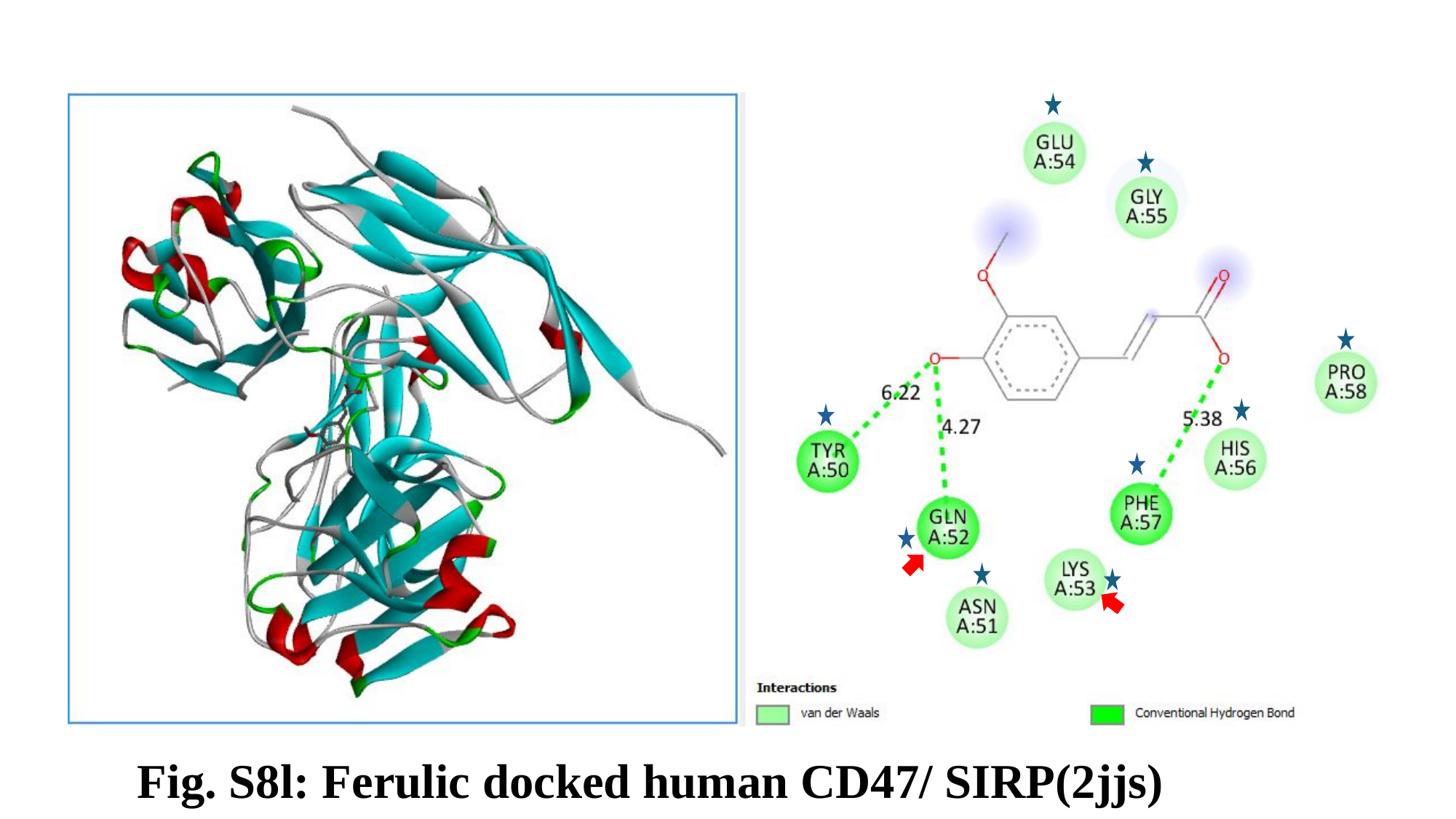

Fig. S8l: Ferulic docked human CD47/ SIRP(2jjs)

## Slide 15
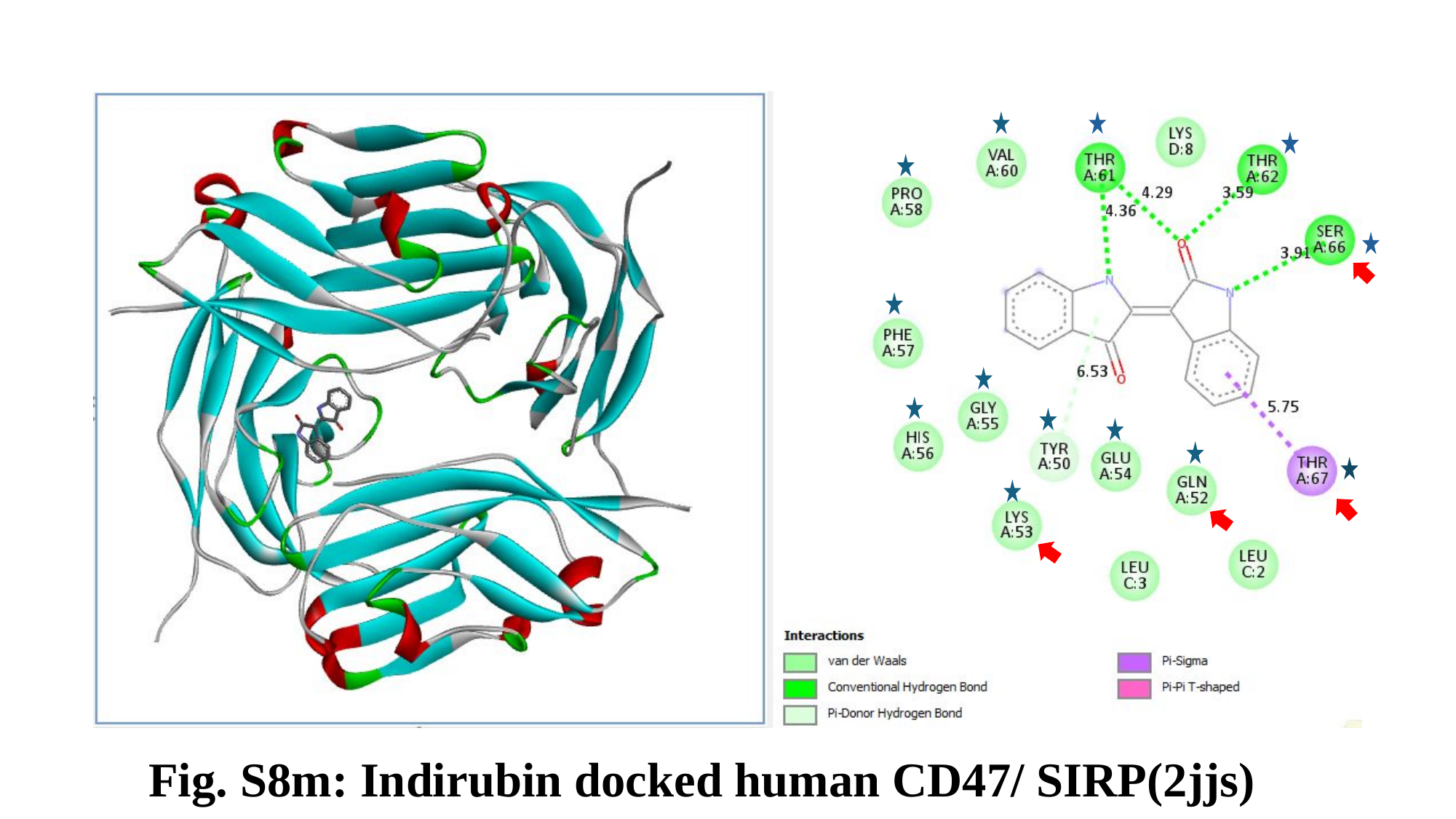

Fig. S8m: Indirubin docked human CD47/ SIRP(2jjs)

## Slide 16
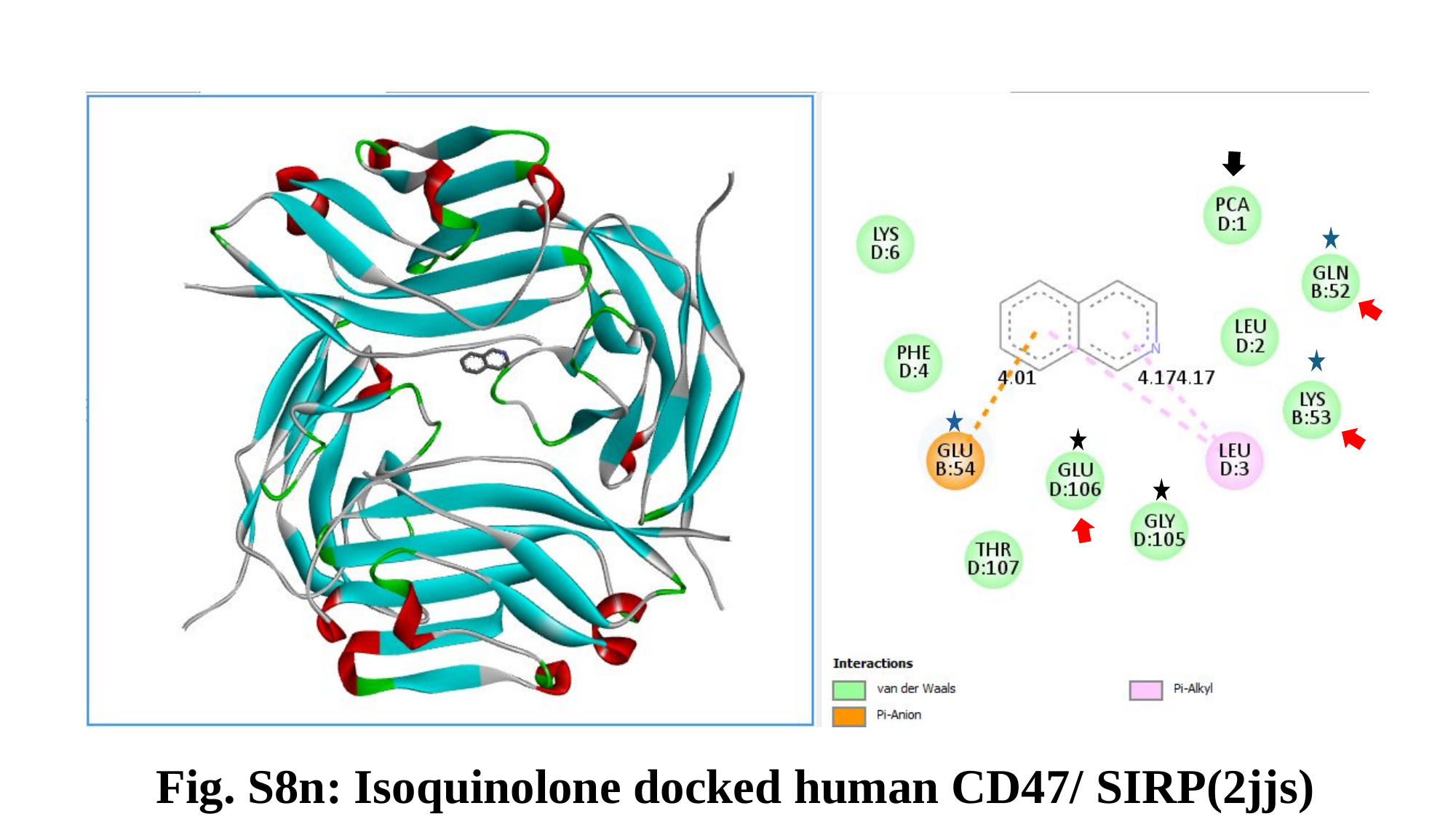

Fig. S8n: Isoquinolone docked human CD47/ SIRP(2jjs)

## Slide 17
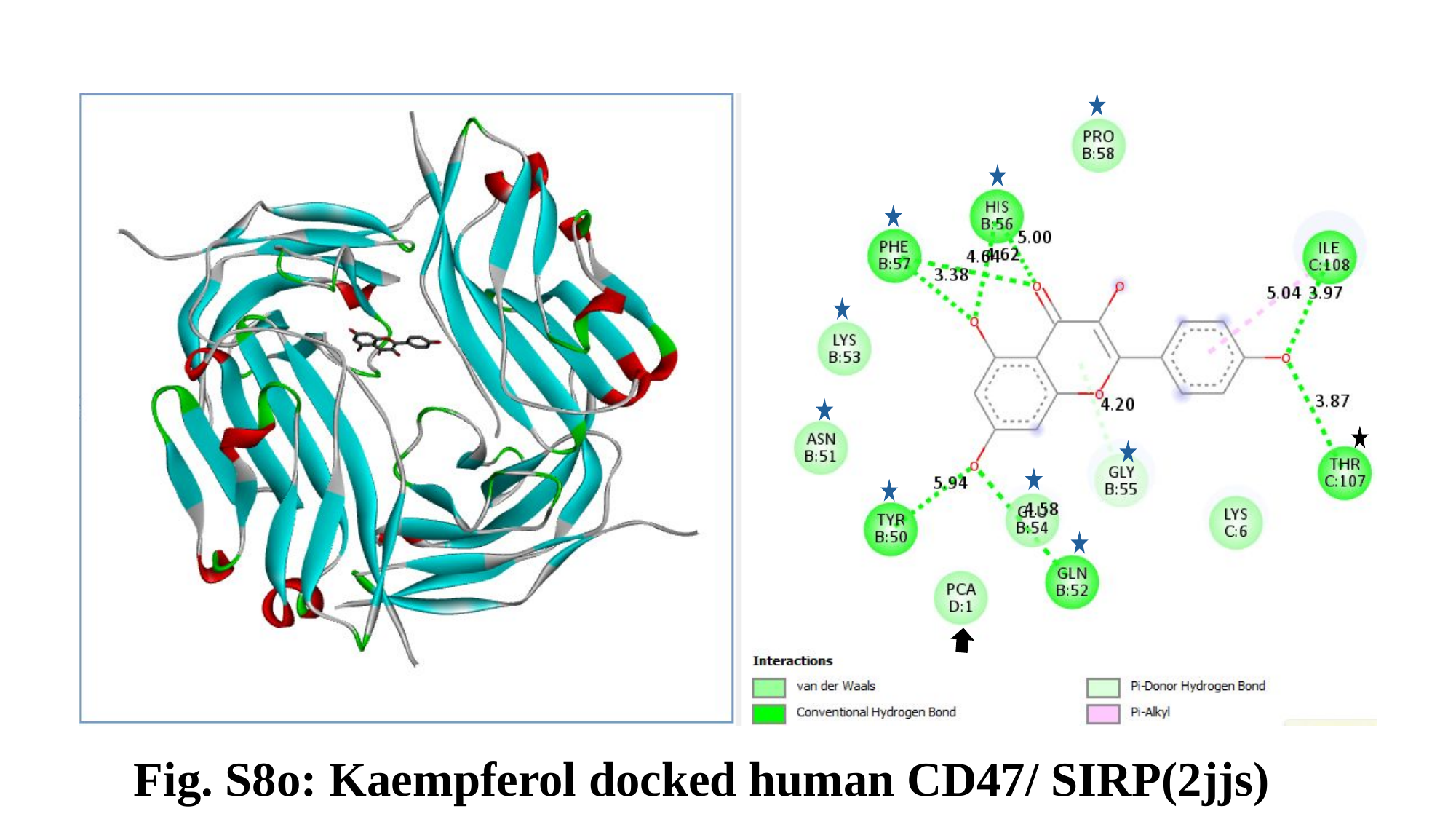

Fig. S8o: Kaempferol docked human CD47/ SIRP(2jjs)

## Slide 18
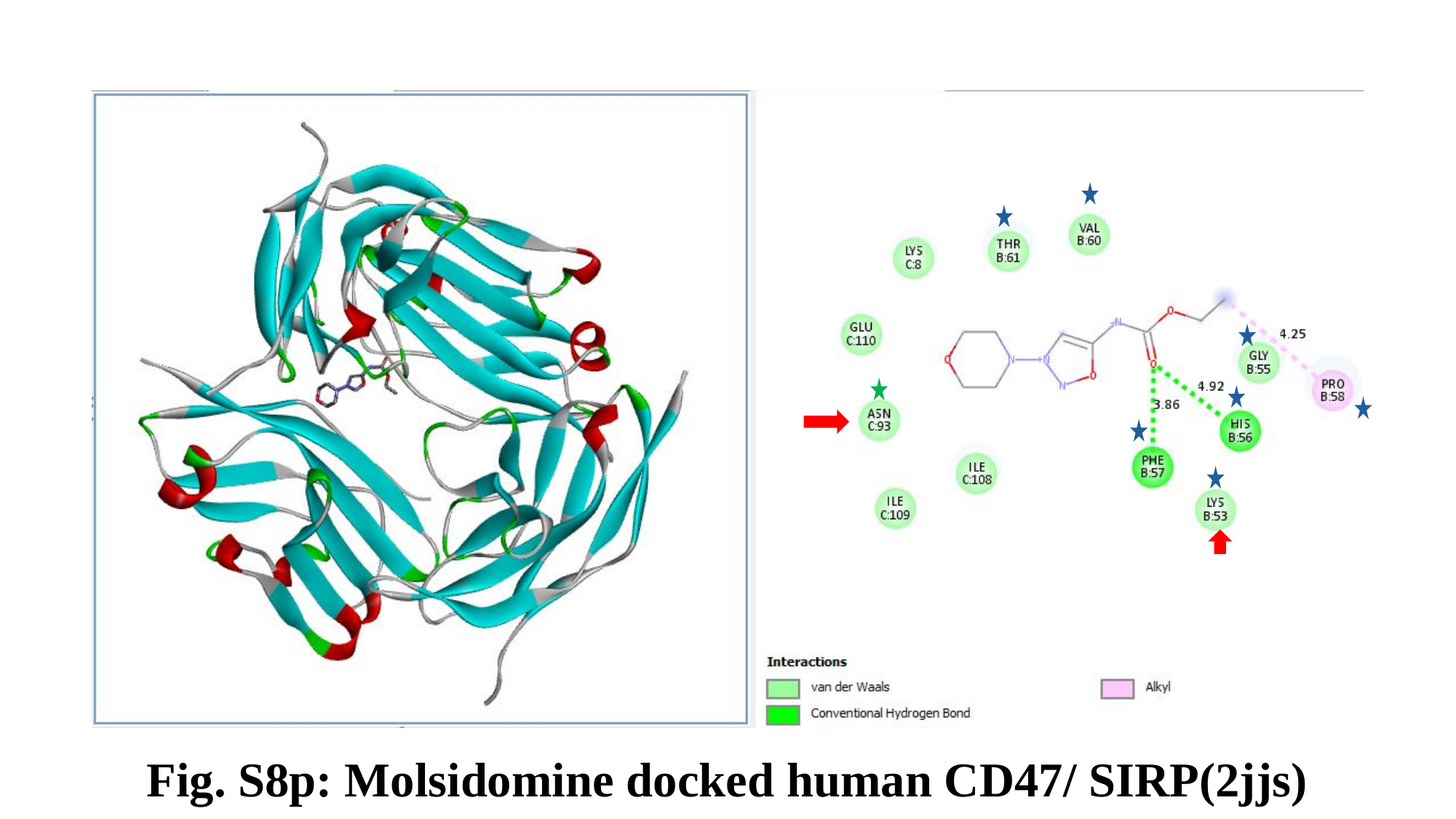

Fig. S8p: Molsidomine docked human CD47/ SIRP(2jjs)

## Slide 19
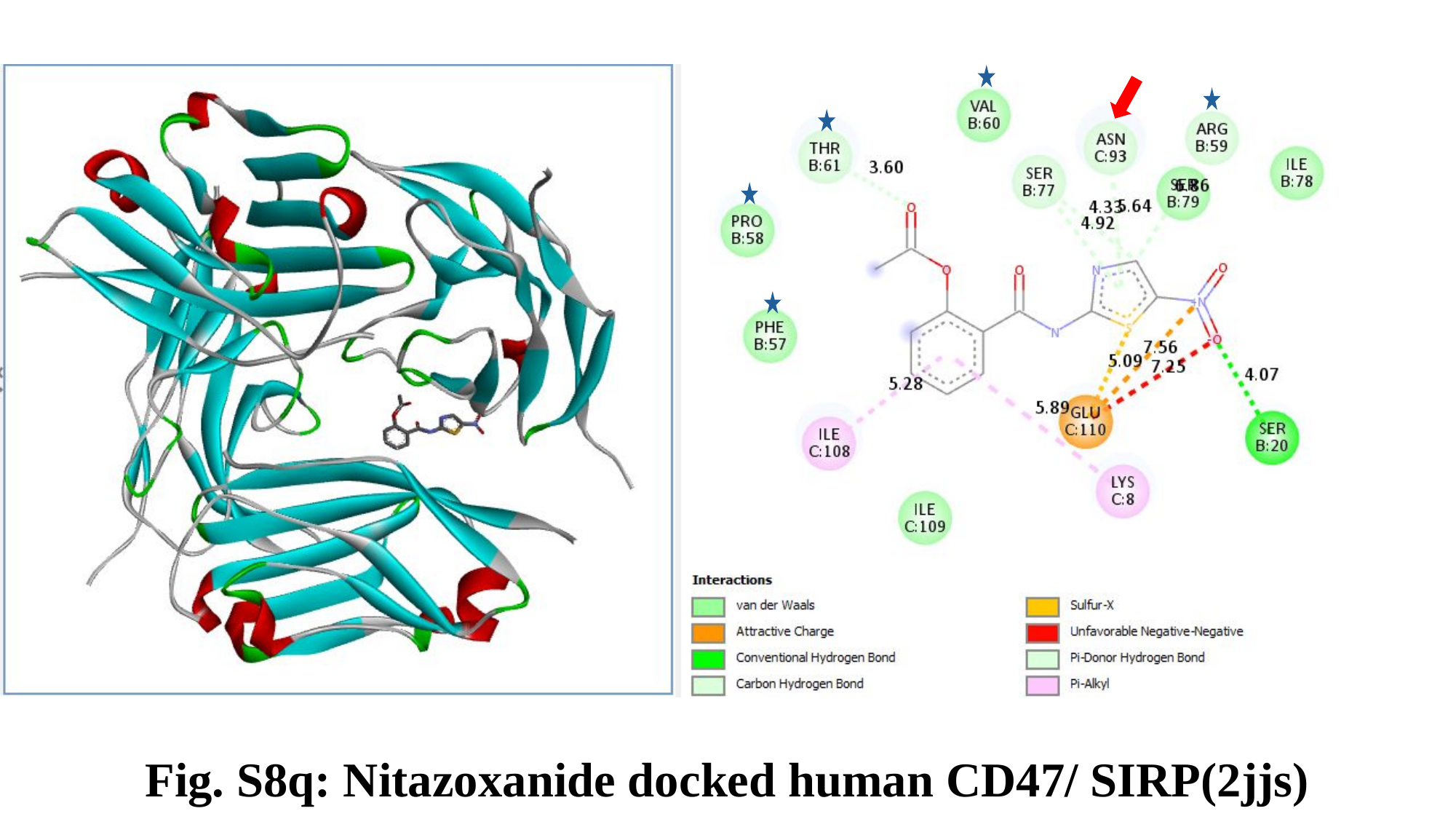

Fig. S8q: Nitazoxanide docked human CD47/ SIRP(2jjs)

## Slide 20
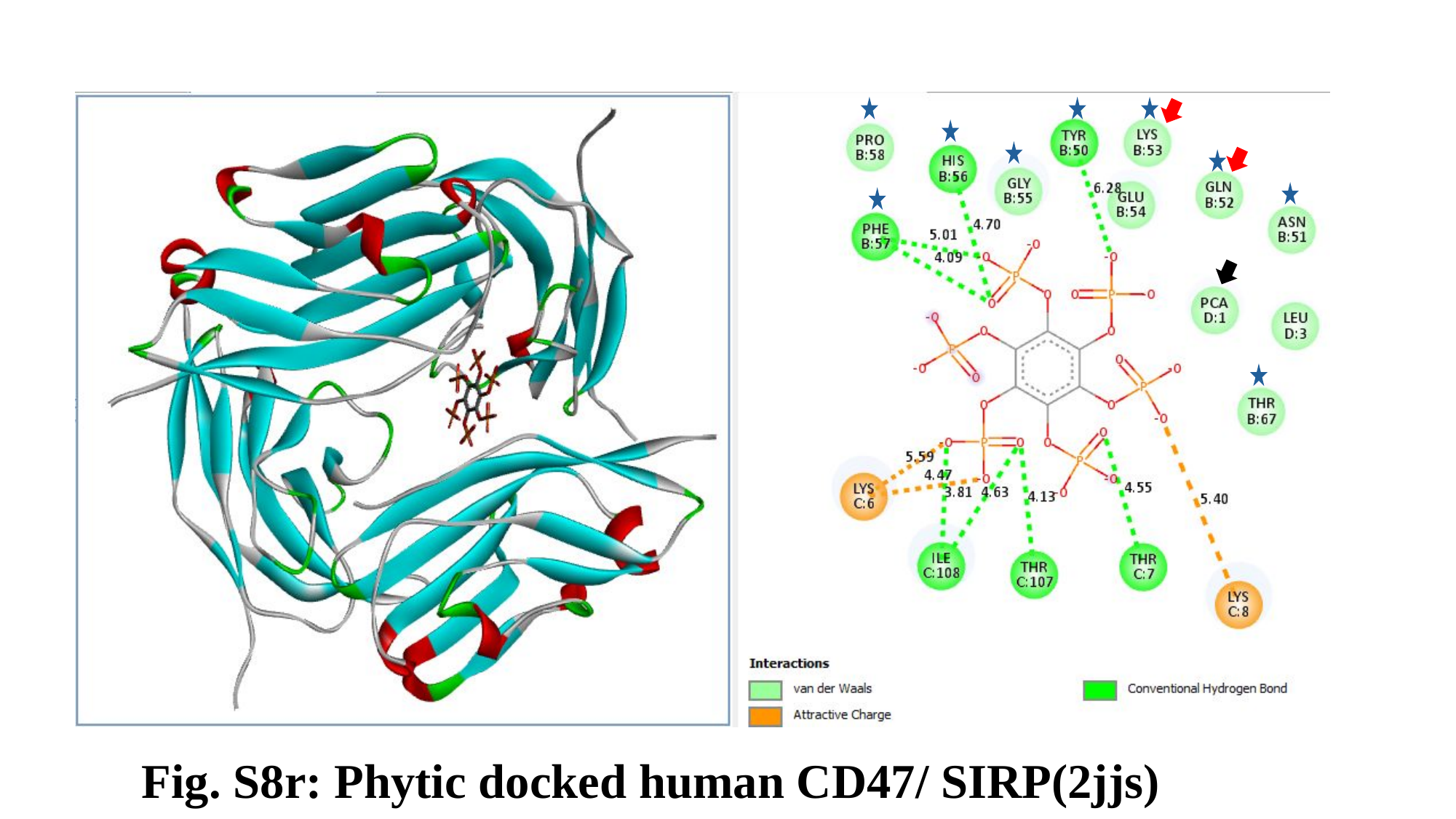

Fig. S8r: Phytic docked human CD47/ SIRP(2jjs)

## Slide 21
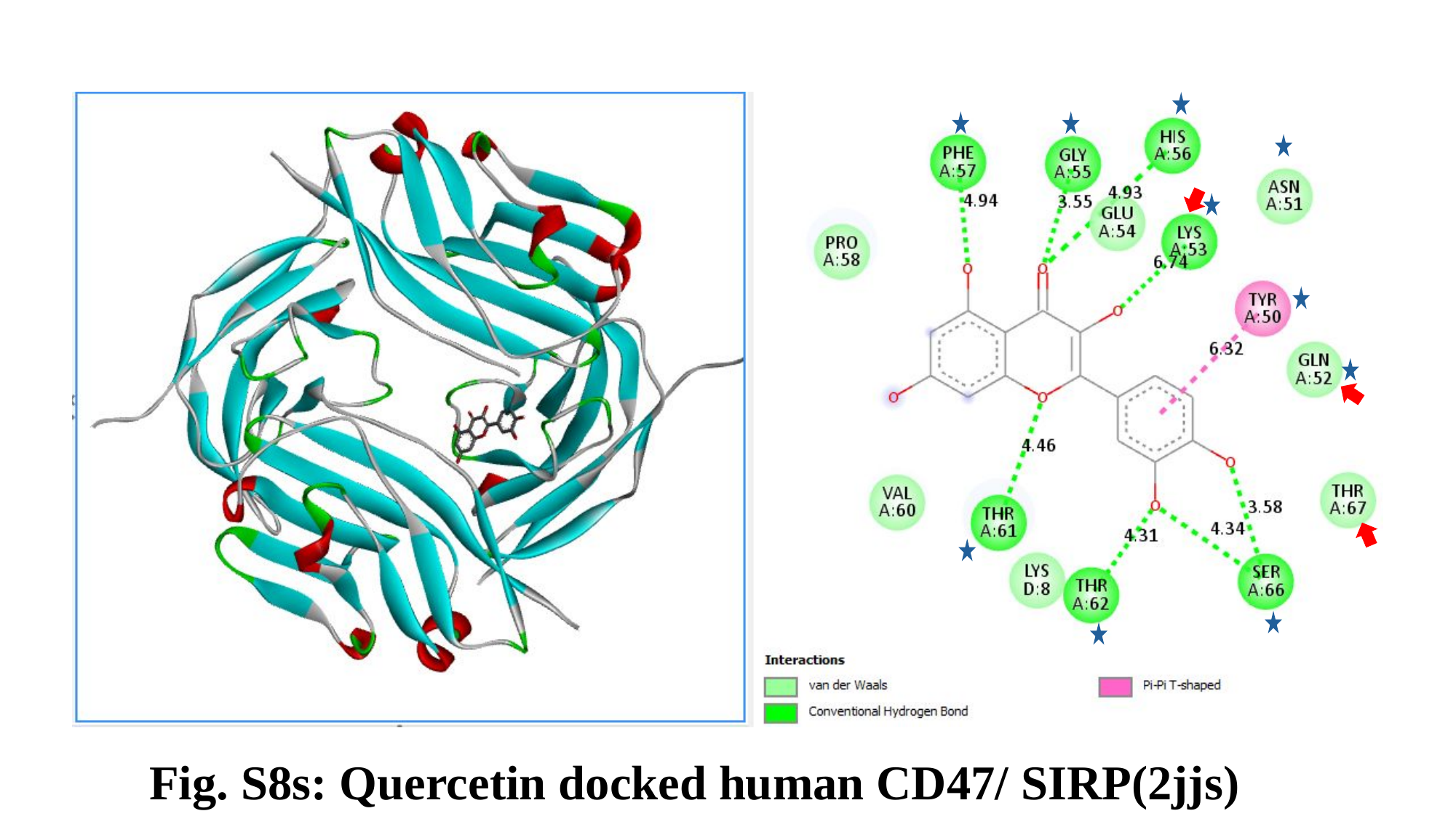

Fig. S8s: Quercetin docked human CD47/ SIRP(2jjs)

## Slide 22
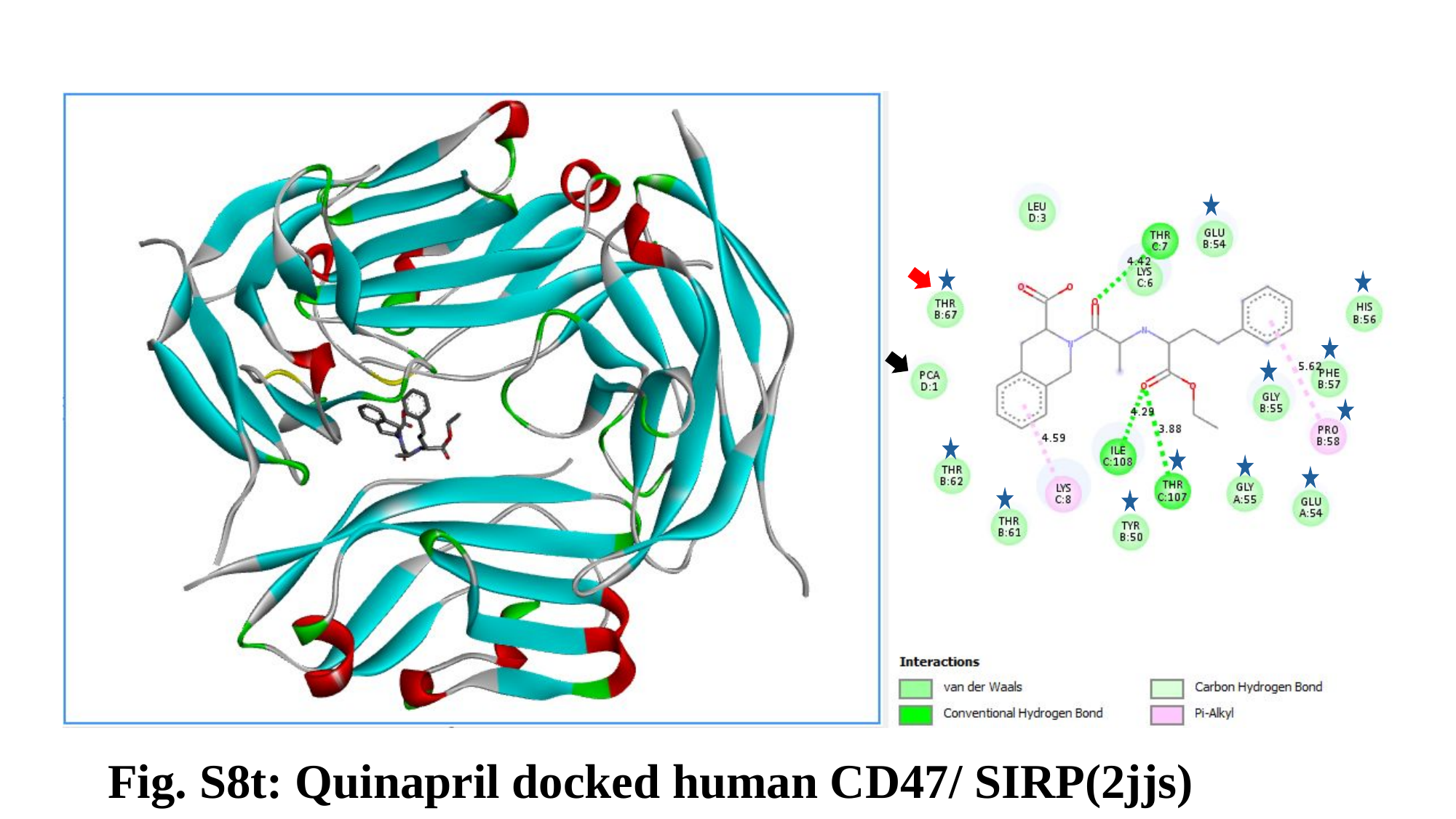

Fig. S8t: Quinapril docked human CD47/ SIRP(2jjs)

## Slide 23
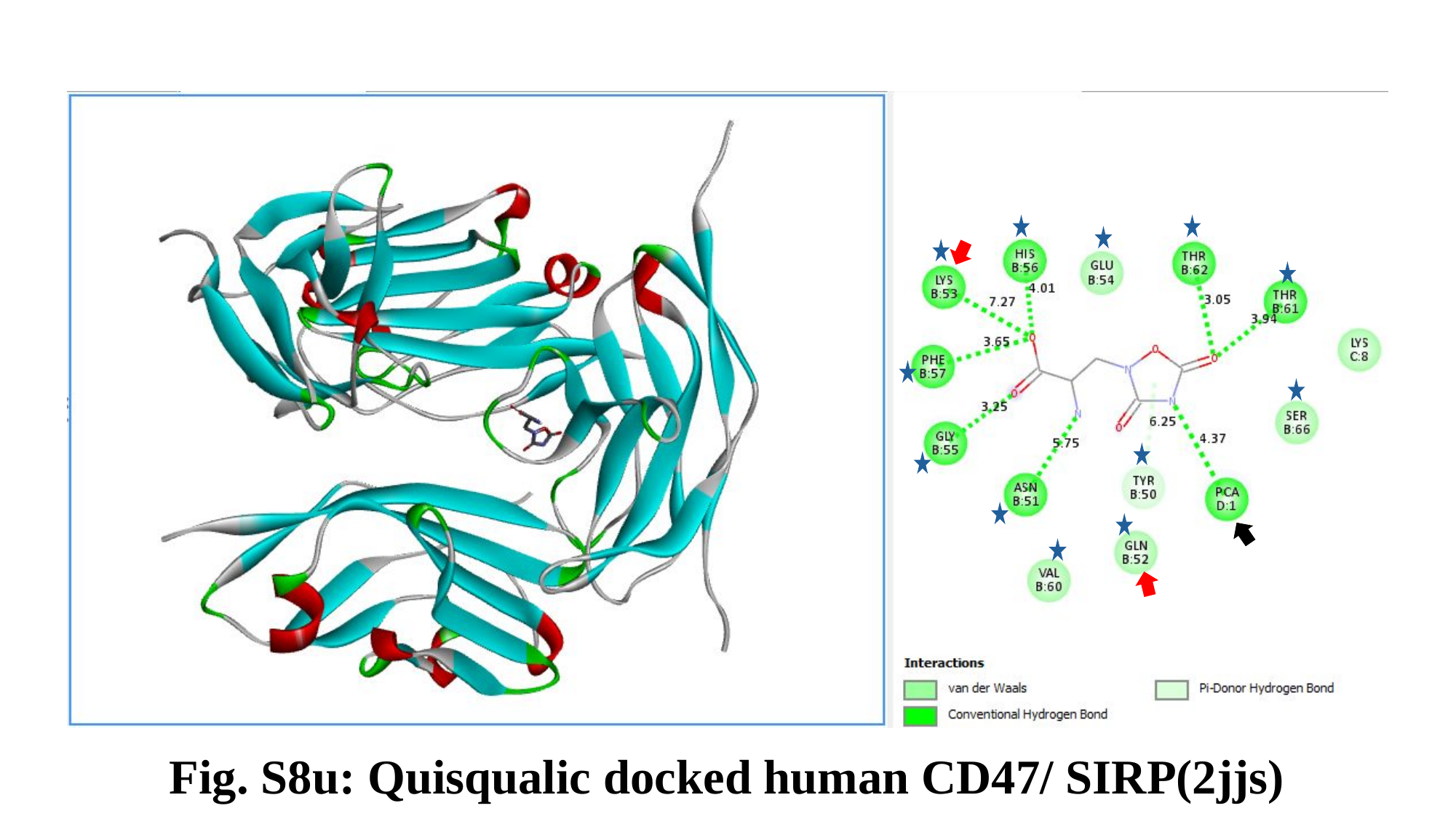

Fig. S8u: Quisqualic docked human CD47/ SIRP(2jjs)

## Slide 24
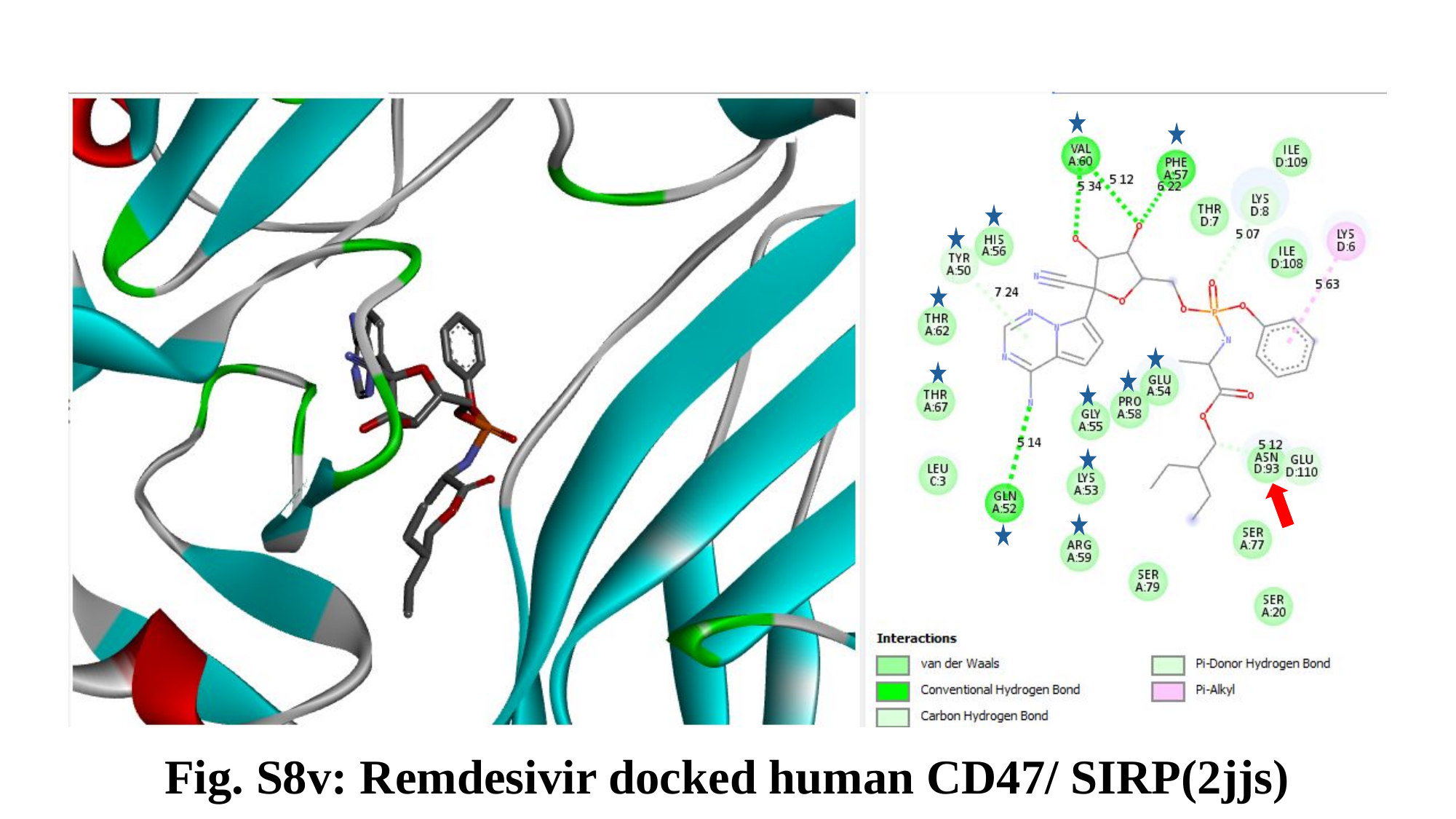

Fig. S8v: Remdesivir docked human CD47/ SIRP(2jjs)

## Slide 25
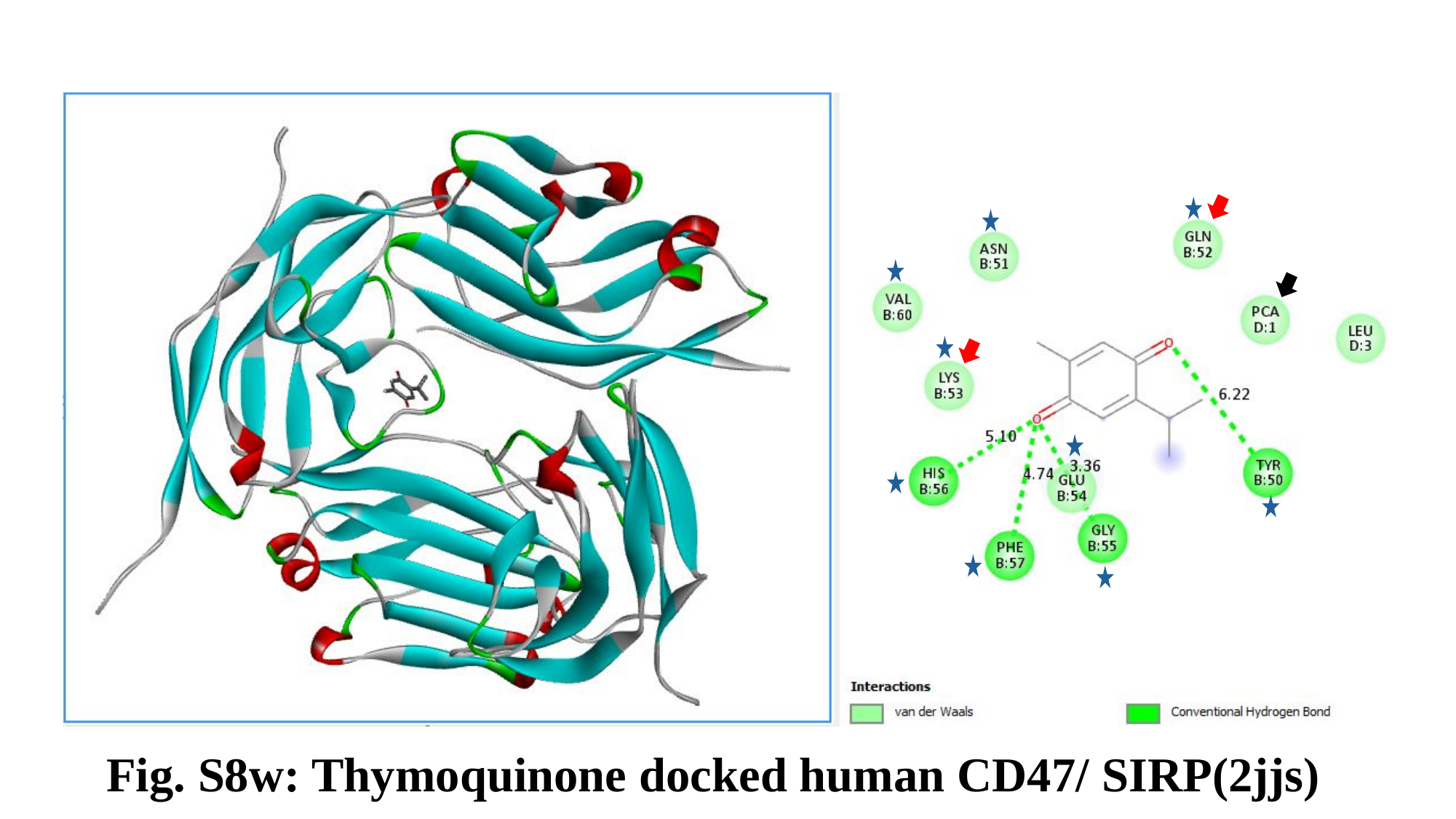

Fig. S8w: Thymoquinone docked human CD47/ SIRP(2jjs)

## Slide 26
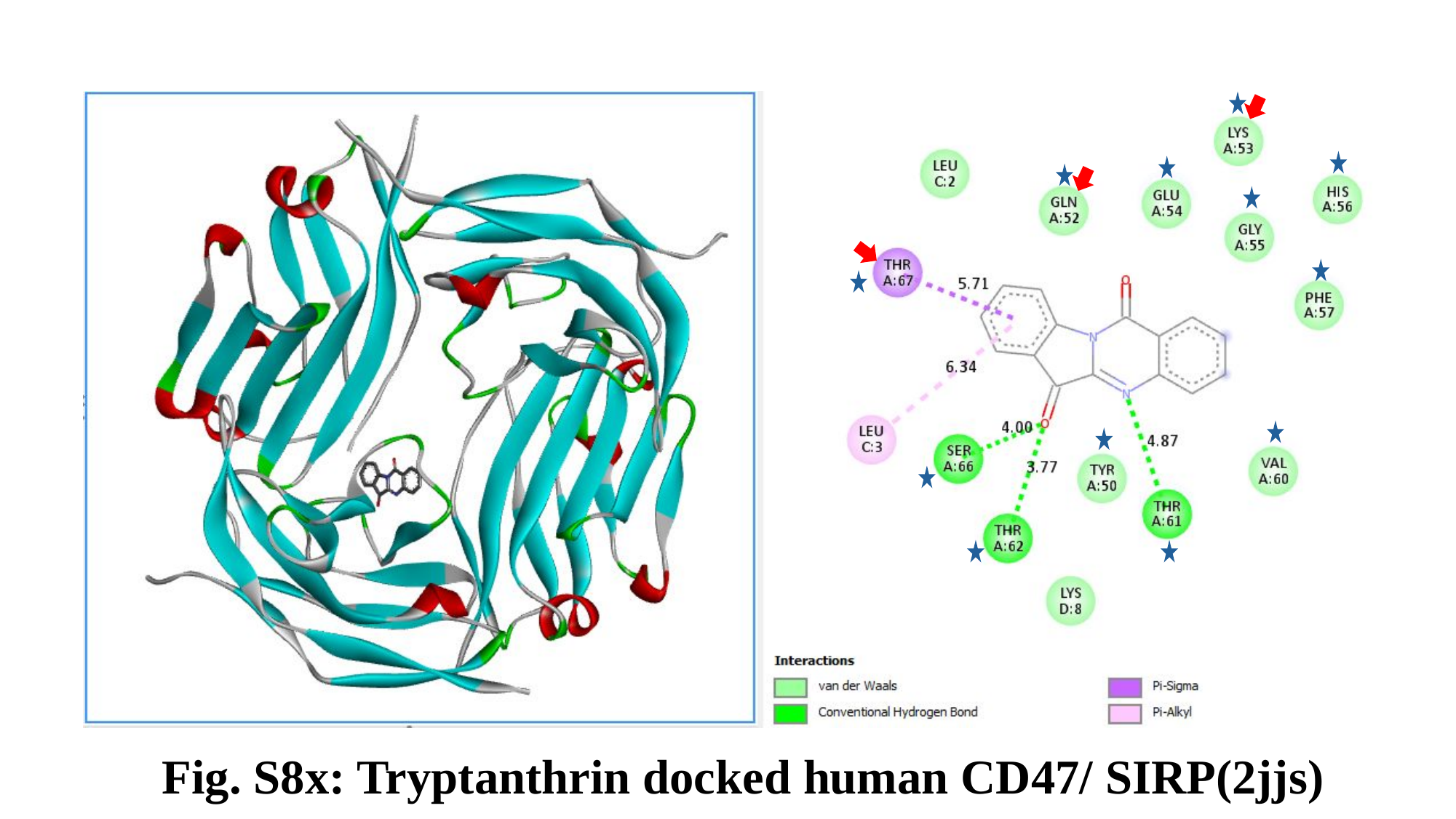

Fig. S8x: Tryptanthrin docked human CD47/ SIRP(2jjs)
