# Supplementary material for: Targeting asparagine and cysteine in SARS-CoV-2 variants and human pro-inflammatory mediators to alleviate COVID-19 severity; a cross-section and in-silico study
Source: Sci Rep. 2025 Nov 3;15:38445. doi: 10.1038/s41598-025-19359-y (PMC12583749; doi:10.1038/s41598-025-19359-y)
Supplement: Supplementary file 16 — Supplementary Material 16 [file 41598_2025_19359_MOESM16_ESM.pdf]

Supplementary file (W)

Percent Identity Matrix - created by Clustal2.1

1: 7cn8_	100.00	92.79	92.91	90.60	90.99	90.99	91.86	91.77	91.94	89.06	90.38	76.33	78.39	76.78
2: 7cn4_	92.79	100.00	97.44	96.87	95.76	95.76	96.72	96.87	96.95	93.58	94.69	76.76	77.86	76.84
3: 8hxj_	92.91	97.44	100.00	96.66	96.78	96.78	97.83	97.91	97.99	93.66	95.31	76.94	78.37	76.84
4: 7vxe_	90.60	96.87	96.66	100.00	97.51	97.51	98.55	98.87	98.71	94.60	94.59	76.24	75.92	75.80
5: 6vxx_	90.99	95.76	96.78	97.51	100.00	100.00	97.78	98.41	98.41	93.20	94.28	76.33	76.78	75.67
6: 6wpt_	90.99	95.76	96.78	97.51	100.00	100.00	97.78	98.41	98.41	93.20	94.28	76.33	76.78	75.67
7: 7qus_	91.86	96.72	97.83	98.55	97.78	97.78	100.00	98.81	98.96	94.43	95.65	76.51	76.74	76.43
8: 7bnn_	91.77	96.87	97.91	98.87	98.41	98.41	98.81	100.00	99.76	94.51	95.65	76.24	76.82	76.09
9: 6zgh_	91.94	96.95	97.99	98.71	98.41	98.41	98.96	99.76	100.00	94.51	95.48	76.42	76.99	76.18
10: 8x4h_	89.06	93.58	93.66	94.60	93.20	93.20	94.43	94.51	94.51	100.00	96.42	74.37	75.28	75.11
11: 8uir_	90.38	94.69	95.31	94.59	94.28	94.28	95.65	95.65	95.48	96.42	100.00	75.00	76.63	75.38
12: 8tc0_	76.33	76.76	76.94	76.24	76.33	76.33	76.51	76.24	76.42	74.37	75.00	100.00	92.06	92.25
13: 6crv_	78.39	77.86	78.37	75.92	76.78	76.78	76.74	76.82	76.99	75.28	76.63	92.06	100.00	99.24
14: 5wrg_	76.78	76.84	76.84	75.80	75.67	75.67	76.43	76.09	76.18	75.11	75.38	92.25	99.24	100.00

Figure W1: Identity percent between SARS-CoV-2 spike proteins for 14 variants showed that the identity percent between the selected Spike protein, (7bnn) which was published in 2021 and Spike protein (8x4h) which was published in 2024, is 94.51%.

Table W1: binding energy and dissociation constant for the studied protein-protein complexes

Protein-protein complex	ΔG (kcal mol^{-1})	K_d (M) at °C	ICs charged-charged	ICs charged-polar	ICs charged-apolar	ICs polar-polar	ICs polar-apolar	ICs apolar-apolar	NIS charged	NIS apolar
7bnn/1bz0	-33.0	5.9e-25	24	37	90	31	99	125	19.77	41.9
7bnn/ 5n9b	-22.6	2.5e-17	8	28	47	28	73	98	19.61	38.21
7bnn/ 1n26	-24.8	7e-19	9	33	57	19	71	101	18.74	40.34
7bnn/ 3fcs	-27.0	1.5e-20	25	41	103	25	62	115	22.95	40.62
7bnn/ 2jjs	-32.1	2.7e-24	17	31	79	30	101	148	20.0	40.65
ASNase/7bnn	-25.0	4.6e-19	13	28	57	20	72	102	19.47	40.66
ASNase/5n9b	-26.1	7.6e-20	11	42	60	30	83	99	19.15	38.64
ASNase/1n26	-8.5	5.4e-07	16	11	21	2	3	5	28.7	39.29
ASNase/1p9m	-5.6	7.4e-05	2	0	2	0	0	0	26.42	37.77
ASNase/3fcs	-19.9	2.6e-15	20	22	79	9	29	74	24.66	38.93
ASNase/2jjs	-29.8	1.3e-22	63	70	82	28	76	86	26.88	44.46
6wir/4hsa	-28.3	1.9e-21	23	41	60	36	92	94	23.7	34.29
6wir/3jvf	-10.9	1.1e-08	8	11	18	10	17	21	21.79	34.97
6wir/5n9b	-17.3	2.1e-13	12	21	46	10	32	52	21.36	36.14
6wir/5nan	-13.7	8.6e-11	14	29	29	12	25	26	21.61	36.31

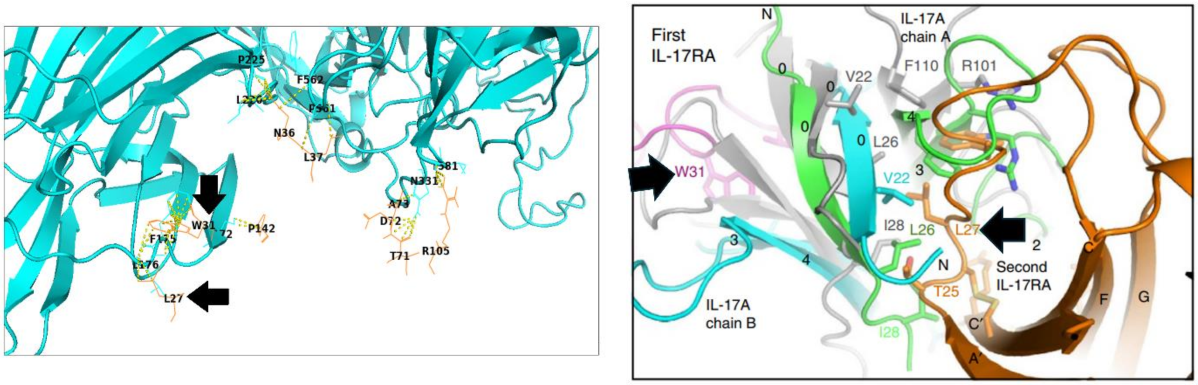

Figure W2: Binding interface between IL-17A and IL17RA in the current study (left), Binding interface between IL-17A and IL17RA published by Liu S et al., Nature Communication, 2013 (right).

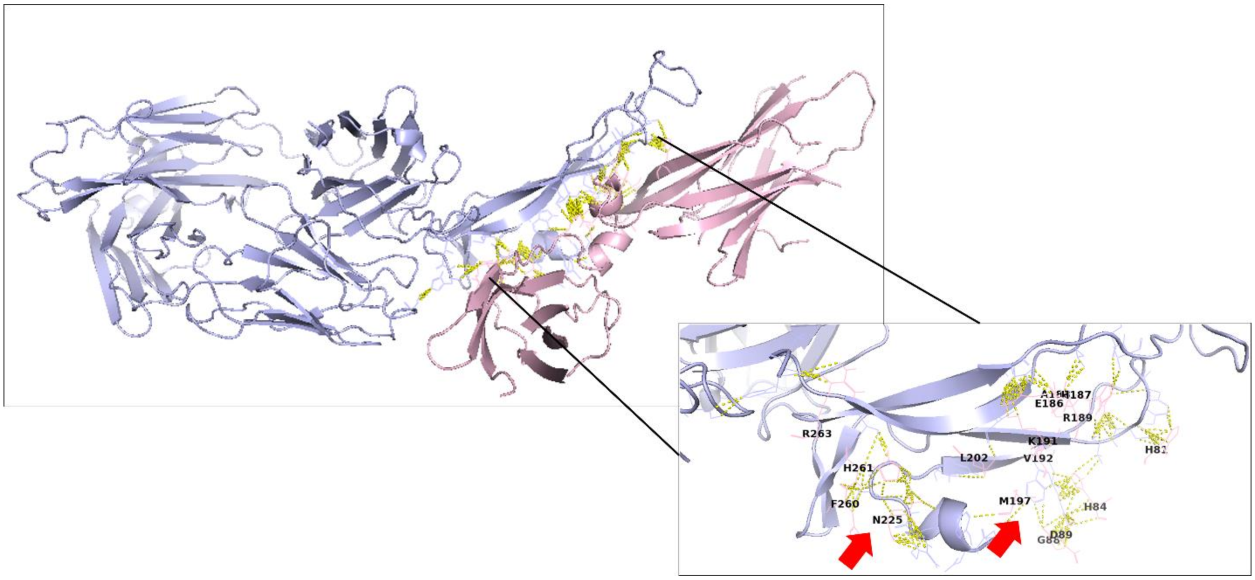

Figure W3: 3D image shows that Secukinumab (Cosentyx), a monoclonal antibody to IL-17, (light blue color) (pdb:6wir, Lieu R et al., 2020) can dock IL-17R (5n9b) at key residues M197, Asn N225 (red arrows).

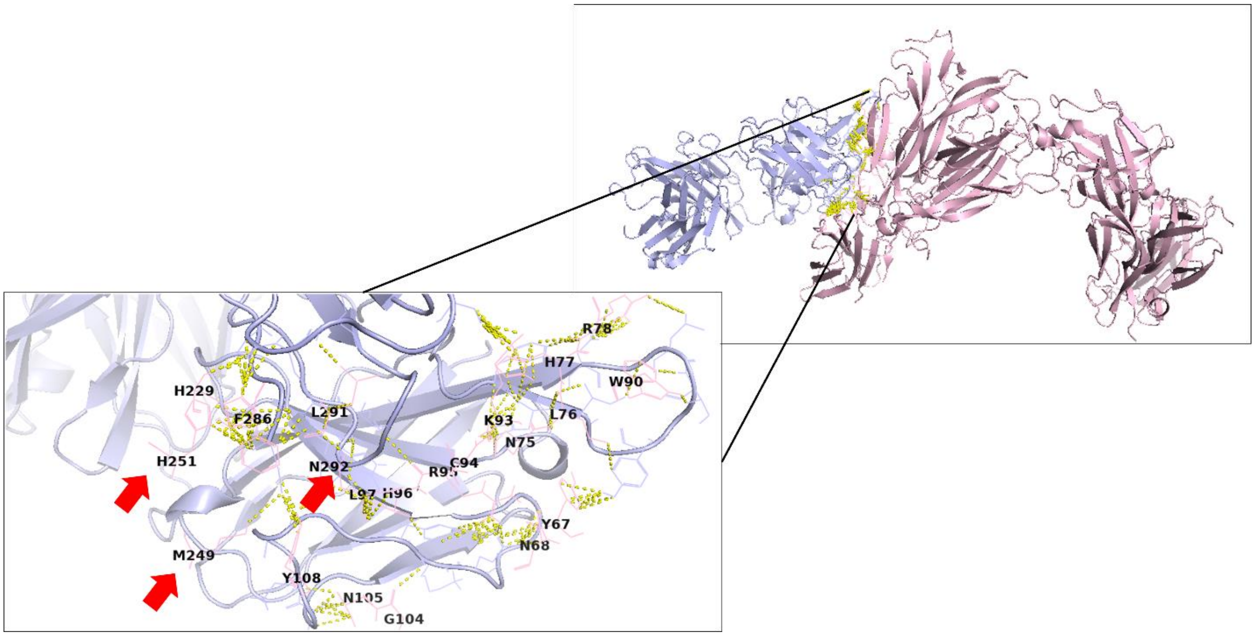

Figure W4: 3D image shows docking of Secukinumab (Cosentyx), a monoclonal antibody to IL-17, (light blue color) (pdb:6wir, Lieu R et al., 2020) to IL-17AF in complex with IL-17RA (5nan) (light pink color), as a reference to the docking of the selected drugs to the critical residues at the binding interface between IL-17 and IL-17R. Secukinumab can dock IL-17/IL-17R at the binding interface through binding to many residues in IL-17 and critical residues in IL-17R including M249 H251 N292 (red arrows).

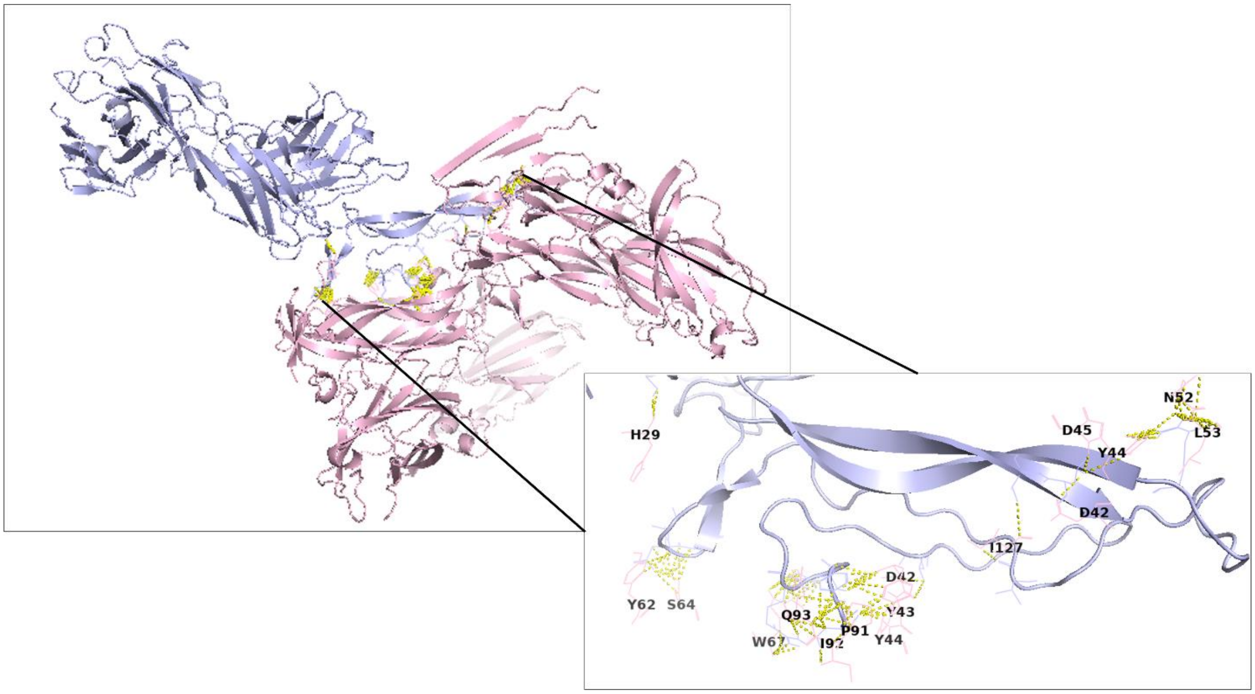

Figure W5:3D image shows docking of Secukinumab (Cosentyx), a monoclonal antibody to IL-17 (light blue color) (pdb:6wir, Lieu R et al., 2020) to IL-17A in complex with IL-17RA (4hsa) (light pink color) as a reference to the docking of the selected drugs to the critical residues at the binding interface between IL-17 and IL-17R.

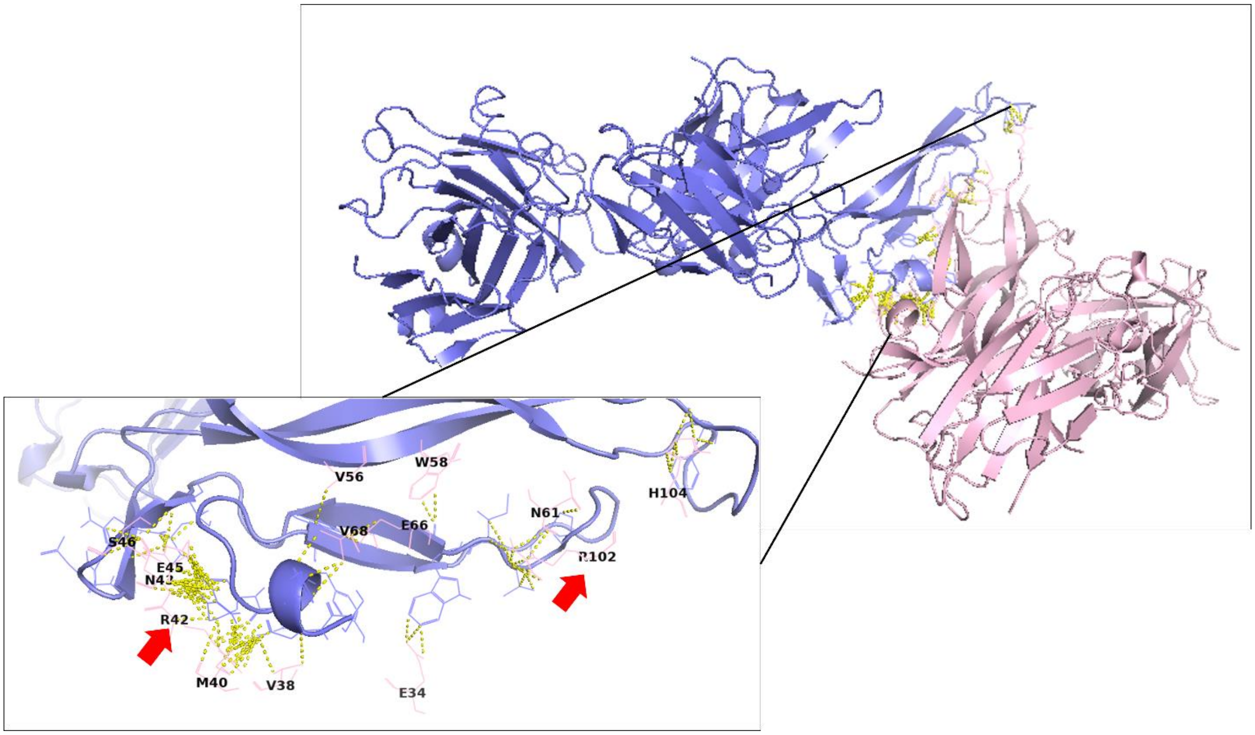

Figure W6: 3D image shows docking of Secukinumab (Cosentyx), a monoclonal antibody to IL-17 (light blue color) (pdb:6wir, Lieu R et al., 2020) to IL-17F in complex with IL-17RA (3jvf) (light pink color) as a reference to the docking of the selected drugs to the critical residues at the binding interface between IL-17 and IL-17R. Secukinumab can dock IL-17/IL-17R at the binding interface through binding to many residues in IL-17 R42, R102 (red arrows).
