# Supplementary material for: Targeting asparagine and cysteine in SARS-CoV-2 variants and human pro-inflammatory mediators to alleviate COVID-19 severity; a cross-section and in-silico study
Source: Sci Rep. 2025 Nov 3;15:38445. doi: 10.1038/s41598-025-19359-y (PMC12583749; doi:10.1038/s41598-025-19359-y)
Supplement: Supplementary file 17 — Supplementary Material 17 [file 41598_2025_19359_MOESM17_ESM.pdf]

<u>**Supplementary R**</u>

Supplementary table R1: Logistic regression analysis of factors associated with disease severity.

**Predictor**	**β (SE)**	**Adjusted OR (95% CI)**	**P value**
**Age (per year)**	0.127 (0.030)	1.14 (1.07–1.20)	<0.001
**Monocytes (per unit)**	0.0024 (0.001)	1.002 (1.001–1.004)	0.013
**Intercept**	−9.56 (2.16)	—	<0.001

Cohort: 85 patients (severe: 38 [44.7%]; non-severe: 47 [55.3%]).
Model fit: Likelihood ratio $\chi^2 = 35.20$ (2 df; $P < 0.001$).
Discrimination: AUC = 0.86 (95% CI: 0.77–0.92).
Variance explained: Nagelkerke's $R^2 = 0.45$
Calibration: Hosmer-Lemeshow $\chi^2 = 15.56$ ($P = 0.049$).

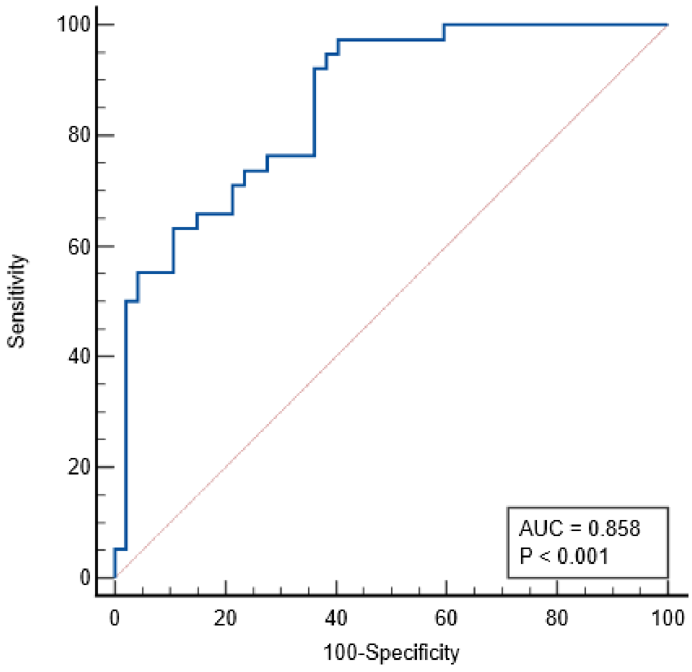

Supplementary Fig. R1. Receiver operating characteristic (ROC) curve of the logistic regression model for disease severity prediction. The model demonstrates strong discriminative ability with an area under the curve (AUC) of 0.858 (95% CI: 0.77–0.92, $P < 0.0001$). The dashed diagonal line represents random chance (AUC = 0.5), while the solid curve shows model performance. Optimal cutoff (>0.235, arrow) yields 97.4% sensitivity and 59.6% specificity (Youden's index = 0.57).

Supplementary table R2. Summary of binding energies (ΔG), origins of natural compounds, and mechanistic profiles of synthetic drugs across target proteins

				Energy (Kcal/ Mol)								
**Class**		**Name**	**Source**	**S**	**IL17R**	**IL17F/IL17RA**	**IL17AF/ IL17RA**	**IL17A/ IL17RA**	**IL6R**	**IL-6/IL6R**	**CD47**	**CD41**
				**(7bnn)**	**(5n9b)**	**(3jvf)**	**(5nan)**	**(4hsa)**	**(1n26)**	**(1p9m)**	**(2jjs)**	**(3fcs)**
Natural	1.	Phytic acid	cereals, oil seeds and nuts	-129.83	-107.12	-119.23	-126.4	-157.76	-122.57	-129.4	-118.63	-113.11
	2.	Amygdalin	bitter almonds, stone fruits, berries, apricots, plums, peaches, apples, papaya, cherries, rice, sugarcane, sorghum, nuts and Linamarin,	-92.97	-92.07	-103.41	-102.67	-109.06	-93.56	-130.9	-131.15	-106.28
	3.	Quercetin	onion, asparagus, and berries	-87.78	-96.78	-120.13	-90.8	-86.99	-81.94	-93.54	-103.02	-82.12
	4.	Apigenin	luteolin, parsley, chamomile tea, naringenin, celery, tangeritin, and baicalein, basil,	-86.98	-79.58	-107.75	-103.19	-95.41	-81.68	-85.14	-88.29	-87.26
	5.	Indirubin	indigo plant, Polygonum tinctorium, Isatis tinctoria and Strobilanthes cusia	-94.13	-88.3	-105.27	-109.12	-99.02	-91.96	-95.05	-89.31	-86.64
	6.	Kaempferol	tea, beans, broccoli, apples, and strawberries, Aloe vera, Delile, Euphorbia pekinensis Rupr, Ginkgo biloba L, and Rosmarinus officinalis	-96.1	-79.3	-102.71	-106.7	-103.33	-91.43	-89.82	-93.44	-92.56
	7.	Tryptanthrin	Polygonum tinctorium	-98.5	-82.01	-95.53	-97.26	-100.1	-85.67	-95.55	-81.45	-80.01

			and Isatis tincto ria									
	8.	Quisqualic	Quisqualis indica	-87.39	-65.71	-94.3	-91.91	-75.31	-74.17	-80.36	-81.66	-85.37
	9.	Ferulic	rice, wheat, oats, and pineapple, grains, beans, coffee, artichoke, peanut and nuts	-72.98	-66.6	-94.12	-93.2	-78.11	-60.66	-85	-68.13	-80.46
	10	Acacetin	Verbascum *lych nitis*, *Odontites viscosus* and Ro binia pseudoacacia	-83.58	-73.76	-88.51	-85.1	-91.88	-78.08	-80.34	-91.89	-86.99
	11	Methionine sulfoximine	Wheat	-87.78	-56.76	-83.3	-69.29	-	-	-	-	-68.87
	12	Artemisinin	Artemisia annua	-80.72	-72.6	-81.66	-95.3	-85.91	-78.18	-92.47	-84.19	-85.54
	13	Coumarin	Apiaceae and Rutaceae	-69.93	-58.8	-80.71	-78.16	-73.44	-78.01	-74.29	-69.62	-72.86
	14	Ascorbate	Citrus fruits, strawberries, cantaloupe, green peppers, tomatoes, broccoli, leafy greens, and potatoes	-76.15	-73.76	-79.18	-76.86	-76.47	-60.07	-85.09	-	-64.68
	15	Isoquinoline	Ranunculales, Menispermacea e, Berberidaceae, Papaveraceae, Hernandiaceae, and Monimiaceae.	-64.7	-57.49	-69.01	-68.1	-73.05	-73.74	-69.23	-62.57	-62.01
	16	Thymoquinon e	Nigella sativa	-63.8	-57.85	-69.56	-68.31	-64.46	-65.82	-60.75	-60.76	-63.87
**Synthe tic**	17	Candesartan	Block of Angiotensin II receptor	-109.01	-113.87	-137.29	-113.62	-130.09	-117.39	-118.59	-121.51	-127.62
	18	aminopterin	block tetrahydrofolate synthesis by binding to dihydrofolate reductase	-100.13	--98.1	-69.005	-95.46	-111.95	-118.9	-106.7	-119.69	-106.66

	19	Remdesivir	Adenosine-analogue nucleotide inhibits the RNA-dependent RNA polymerase (RdRp)	-102.9	-97.92	-97.00	-111.61	-107.17	-101.28	-111.65	-121.2	-101.47
	20	nitazoxanide	inhibits pyruvate:ferredoxin oxidoreductase enzyme-dependent electron transfer reactions	-97.65	-74.54	-82.13	-85.78	-101.21	-80.99	-85.61	-82.07	-82.97
	21	Calcitriol	active form of vitamin D	-91.61	-81.7	-90.77	-83.15	-78.92	-84.21	-97.05	-103.69	-95.65
	22	Dexamethasone	suppressing the migration of neutrophils and decreasing lymphocyte colony proliferation, promote apoptosis	-82.03	-81.9	-84.04	-86.04	-79.63	-94.56	-82.5	-96.92	-87.27
	23	Enalapril	inhibitor of ACE activity	-106.28	-96.01	-108.77	-95.73	-116.73	-87.9	-98.97	-99.05	-85.83
	24	Quinapril	inhibitor of ACE activity	-87.3	-82.04	-97.26	-88.29	-88.95	-76.71	-79.45	-98.3	-85.44
	25	Molsidomine	Nitric oxide donor	-74.59	-74.6	-93.46	-97.14	-92.45	-75.13	-77	-85.56	-79.9
	26	Bromopyruvate	induces apoptosis via enhancing endoplasmic reticulum stress and inhibits global protein synthesis	-52.98	-48.87	-66.15	-61.09	-61.14	-54.18	-64.74	-53.11	-52.17
	27	Zinc	anti-inflammatory effect, improves mucociliary clearance and barrier function of the respiratory epithelium, direct antibacterial	-	-	-	-	-	-	-	-	-

			effects against S.										

Supplementary table R3: Statistical Comparison of Mean Binding Energies (ΔG) of Natural vs. Synthetic Compounds Across Target Proteins (including ΔΔG, 95% CI, and *p* Values)

Target	Binding Affinity (ΔG) Natural Compounds	Synthetic Compounds	ΔΔG	95% CI	p	I^2 (%)	τ^2
**7bnn**	-85.83±15.3	-91.19 ± 19.03	-5.36	-17.91 to 7.19	0.39	68	0.32
**5n9b**	-75.62±14.6	-84.16 ± 18.89	-8.54	-19.68 to 2.60	0.13	72	0.41
**3jvf**	-93.4±15.23	-94.25 ± 21.71	-0.85	-15.09 to 13.39	0.91	65	0.29
**5nan**	-91.4±15.98	-98.02 ± 18.86	-6.62	-18.76 to 5.52	0.28	58	0.22
**4hsa**	-91.35±21.75	-96.92 ± 22.45	-5.57	-22.09 to 10.95	0.50	75	0.48
**1n26**	-81.17±14.93	-87.13 ± 19.97	-5.96	-18.25 to 6.33	0.33	70	0.35
**1p9m**	-89.87±18.42	-92.08 ± 17.88	-2.21	-15.33 to 10.91	0.74	62	0.27
**2jjs**	-87.44±99.75	-98.07 ± 19.77	-10.63	-59.39 to 38.13	0.66	80	0.63
**3fcs**	-84.71±16.21	-88.80 ± 22.31	-4.09	-18.36 to 10.18	0.57	78	0.57

ΔG (kcal/mol): Mean ± standard deviation of binding energies.
ΔΔG represents the difference in binding free energy (ΔG) between Natural and Synthetic compounds binding to target proteins.
95% CI : 95% confidence interval for ΔΔG (Natural − Synthetic).
***I^2*:** Percentage of total variation due to between-target heterogeneity ($I^2 > 50\%$ = substantial).
***τ^2*:** Estimated variance of true effect sizes (random-effects model applied where $I^2 > 50\%$).

Supplementary table R4: The mean of binding free energy (ΔG) to the studied proteins, half-maximal inhibitory concentration (IC_{50}), and corresponding negative logarithm (pIC_{50}) for natural and synthetic compounds, derived from molecular docking simulations.

Drug	ΔG (kcal/mol)	IC_{50} (nM)	pIC_{50}
**Phytic acid**	−124.89±13.57	2.8e-83	91.554
**Amygdalin**	−106.89±14.11	4.4e-70	78.359
**Quercetin**	−93.67±11.33	4.5e-59	66.378
**Apigenin**	−90.58± 9.04	3.9e-57	64.659
**Indirubin**	−95.42±7.28	1.3e-60	68.237
**Kaempferol**	−95.04±7.89	2.9e-60	67.968
**Tryptanthrin**	−90.67±7.74	3.8e-57	64.699
**Quisqualic acid**	−81.79±8.58	4.0e-50	56.375
**Ferulic acid**	−77.69±11.04	5.5e-47	52.98
**Acacetin**	−84.45±5.83	1.1e-51	59.119
**Methionine sulfoximine**	−73.20±11.25	9.2e-45	44.714
**Artemisinin**	−84.06±6.54	2.1e-51	58.796
**Coumarin**	−72.86±6.10	2.1e-44	44.46
**Ascorbate**	−74.03±7.49	4.6e-45	44.878
**Isoquinoline**	−66.65±5.07	1.5e-37	35.842
**Thymoquinone**	−63.90±3.52	2.8e-35	33.896
**Candesartan**	−120.99±8.56	1.1e-80	88.393
**Aminopterin**	-102.97±14.47	1.4 e-64	72.85
**Dexamethasone**	−86.09±5.61	2.7e-65	64.63
**Remdesivir**	−105.80±7.40	1.1e-70	76.086
**Nitazoxanide**	-85.88±7.91	4.3e-63	71.37
**Calcitriol**	−89.64±7.76	1.6e-56	65.637
**Enalapril**	−98.62±9.29	4.7e-64	71.572
**Quinapril**	−87.08±6.89	1.5e-54	62.837
**Molsidomine**	−83.31±8.51	8.5e-53	61.073
**Bromopyruvate**	−57.15±5.83	1.3e-33	41.896

ΔG values are expressed in kcal/mol, IC_{50} values in nanomolar (nM), and pIC_{50} calculated as $-\log_{10}(IC_{50}$ [M]).
